# Phylogenetic analyses of 5-hydroxytryptamine 3 (5-HT_3_) receptors in Metazoa

**DOI:** 10.1371/journal.pone.0281507

**Published:** 2023-03-01

**Authors:** Santosh T. R. B. Rao, Ilona Turek, Helen R. Irving

**Affiliations:** 1 La Trobe Institute for Molecular Science, La Trobe University, Bendigo, Victoria, Australia; 2 Department of Rural Clinical Sciences, La Trobe University, Bendigo, Victoria, Australia; Laboratoire de Biologie du Développement de Villefranche-sur-Mer, FRANCE

## Abstract

The 5-hydroxytrptamine 3 (5-HT_3_) receptor is a member of the ’Cys-loop’ family and the only pentameric ligand gated ion channel among the serotonin receptors. 5-HT_3_ receptors play an important role in controlling growth, development, and behaviour in animals. Several 5-HT_3_ receptor antagonists are used to treat diseases (e.g., irritable bowel syndrome, nausea and emesis). Humans express five different subunits (A-E) enabling a variety of heteromeric receptors to form but all contain 5HT3A subunits. However, the information available about the 5-HT_3_ receptor subunit occurrence among the metazoan lineages is minimal. In the present article we searched for 5-HT_3_ receptor subunit homologs from different phyla in Metazoa. We identified more than 1000 5-HT_3_ receptor subunits in Metazoa in different phyla and undertook simultaneous phylogenetic analysis of 526 5HT3A, 358 5HT3B, 239 5HT3C, 70 5HT3D, and 173 5HT3E sequences. 5-HT_3_ receptor subunits were present in species belonging to 11 phyla: Annelida, Arthropoda, Chordata, Cnidaria, Echinodermata, Mollusca, Nematoda, Orthonectida, Platyhelminthes, Rotifera and Tardigrada. All subunits were most often identified in Chordata phylum which was strongly represented in searches. Using multiple sequence alignment, we investigated variations in the ligand binding region of the 5HT3A subunit protein sequences in the metazoan lineage. Several critical amino acid residues important for ligand binding (common structural features) are commonly present in species from Nematoda and Platyhelminth gut parasites through to Chordata. Collectively, this better understanding of the 5-HT_3_ receptor evolutionary patterns raises possibilities of future pharmacological challenges facing Metazoa including effects on parasitic and other species in ecosystems that contain 5-HT_3_ receptor ligands.

## Introduction

Serotonin known as 5-hydroxytryptamine (5-HT) is an ancient molecule that acts as a neurotransmitter and is present in plants and animals. 5-HT from plants participates in plant metabolism and serves as a nutrient to animal species [1]. Animal species can produce their own 5-HT as they are equipped with enzymes such as tryptophan hydroxylase (TPH) and aromatic L-amino acid decarboxylase [2]. 5-HT influences animal behaviour and metabolism through neuromodulatory signalling with dysfunction of the 5-HT system affecting animal behaviours [3–5]. 5-HT is implicated in mobility, auditory and sexual behaviours. For example swimming behaviour in Nudipleura molluscs is strongly associated with neural synaptic strength generated by serotonergic neuromodulation [6]. 5-HT participates in auditory behaviour of cats, mice [7–10] and also in Mexican free tailed bats (*Tadarida brasiliensis mexicana*), where intraperitoneally applied 5-HT altered the relation between species-specific communication calls [11]. *Tph2* knock out mice lack 5-HT in the brain and lose gender preference suggesting that 5-HT and serotonergic neurons in the adult brain regulate mammalian sexual preference [12, 13].

To adapt to environmental risks, several species rely on regulating transcription of TPH [14] and other factors that influence 5-HT synthesis enabling modification of their basic behavioural processes (mobility, auditory and sexual). For instance, reactive oxygen species (ROS) generated by nonylphenol exposure disturbs 5-HT synthesis in *Caenorhabditis elegans* [15] condensing duration of forward movement [16] and decreasing learning ability [17]. In mammals including humans, 5-HT is present in central and peripheral nervous systems, the gastrointestinal tract, platelets and other tissues [18]. 5-HT plays important roles in cell division, differentiation, neuronal migration, emotion, cognition, memory, pain perception, and gastrointestinal functions including secretion and motility by binding to specific 5-HT receptors distributed throughout the body [11, 19, 20]. Serotonergic neurons which are equipped with 5-HT receptors contribute in acoustic, sexual, and social behaviour of animals [7, 11, 21–24].

In mammals, 15 distinct subtypes in the 5-HT_1_ to 5-HT_7_ receptor families exist [20, 25–28]. All but the 5-HT_3_ receptor belong to the G protein coupled receptor superfamily [28–31]. 5HT_1_, 5-HT_2,_ 5-HT_4_, 5-HT_6_ and 5-HT_7_ expression occurs in both vertebrates and invertebrates; 5-HT_7_R is notable for wide expression in phylum Platyhelminthes [15, 32–38]. The 5-HT_3_ receptors are ligand gated pentameric ion channels of the cysteine-loop family related to acetylcholine (ACh), glycine receptors (GlyRs) and γ-aminobutyric acid (GABA) receptors [39–41]. 5-HT activates 5-HT_3_ receptors by generating desensitising inward rectifying currents allowing sodium, potassium, or calcium ions to flow into the cell through the ion channel pore [39, 42].

Available information about 5-HT_3_ receptor gene or protein expression in metazoan phyla other than Chordata is limited. However, indirect evidence using 5-HT_3_ receptor specific ligands suggests that 5-HT_3_ receptors are present in Annelida, Arthropoda, and Mollusca. The 5-HT_3_ receptor agonist 1-m-chlorophenylbiguanide (mCPBG) successfully blocked release of intracellular calcium in the mollusc, *Ruditapes philippinarum* [43]. The 5-HT_3_ receptor agonists, m-CPBG, 1-phenylbiguanide (1-PBG), and alpha-methyl-5-hydroxytryptamine (2-CH3-5-HT) evoked concentration-dependent excitatory effects on isolated auricles from cuttlefish, *Sepia officinalis* [44]. The 5-HT_3_ receptor antagonist ondansetron prevents the larval settlement and metamorphosis of marine fouling invertebrate barnacle *Amphibalanus amphitrite* (Arthropoda) [45]. Another 5-HT_3_ receptor antagonist tropisetron decreased both the frequency and amplitude of the 5-HT-evoked contractions in the worms *Eisenia fetida* and *Lumbricus terrestris* (Annelida) [46].

5-HT_3_ receptor antagonists from the setron family (e.g., ondansetron, ramosetron, etc.) are common medications used to treat chemotherapy or operation-induced nausea, vomiting and diarrhoea in medical and veterinary practice [42, 47–50]. Several clinical trials and animal studies suggested that 5-HT_3_ receptor antagonists are effective in treating Alzheimer’s disease (AD), Parkinson’s disease (PD), antipsychotic-associated tardive dyskinesia, schizophrenia, depression, anxiety, addictions, cognitive disfunction, eating disorders, irritable bowel syndrome (IBS) and other gastrointestinal disorders [49, 51–69]. 5-HT_3_ receptor agonists and competitive antagonists bind to the ligand binding site in the extracellular domain to modulate receptor function, especially receptor pore opening for cations [70].

There are five different 5-HT_3_ receptor subunits encoded by the genes *HTR3A*, *HTR3B*, *HTR3C*, *HTR3D* and *HTR3E* in humans. 5-HT_3_ receptors can form homomeric (all A subunits) or heteromeric (mixture of A and some combination of B, C, D, or E subunits) functionally active channels in humans with the orthosteric ligand binding site occurring in the extracellular N terminal region at the interface between two A subunits [71–74]. Subunit A and the other subunits (B, C, D and E) are differentially expressed in human brain regions and gastrointestinal layers [75–79], where their biophysical characteristics contribute to receptor function [80–82]. For example, 5-HT_3_AB receptor heteromers showed altered conductance and calcium ion channel permeability compared with homomeric 5-HT_3_A receptor during ligand binding [75, 83, 84]. The human C and E subunits also influence the electrical properties in response to 5-HT_3_ receptor agonists and antagonists [81, 82]. In contrast to humans, rodents only express A and B subunits just forming 5HT_3_A receptor homomers or 5-HT_3_AB receptor heteromers [80, 85].

Since different subunit composition influences ligand responses and there are disparities in the number of 5-HT_3_ receptor subunits present in different species, we undertook a search to identify and compare 5-HT_3_ receptor subunits present in the Metazoa. We identified that all subunits are present in some members of Chordata, Mollusca, Platyhelminthes, and Cnidaria. As the A subunit is particularly important in forming the orthosteric binding site [85, 86], we investigated how ligand binding components (amino acid residues) in this subunit are altered across the phyla.

## Materials and methods

### Data sources

Protein sequences of human 5-HT_3_ receptor subunits were used to retrieve the 5-HT_3_ receptor subunit protein sequences in other species through protein-protein Basic Local Alignment Search Tool (BLASTp) at the National Centre for Biotechnology Information (NCBI) database (http://www.ncbi.nlm.nih.gov). The BLASTp search for 5-HT_3_ receptor subunits was performed using full-length protein sequences from human (*Homo sapiens*): 5HT3A - AAP35868.1, 5HT3B - EAW67236.1, 5HT3C - AAL66182.1, 5HT3D - NP_001138615.1, and 5HT3E - NP_938056.1.

### Sequence search parameters and sequence selection

The search was limited to Metazoa and included all phyla (Acanthocephala, Annelida, Arthropoda, Brachiopoda, Bryozoa, Chaetognatha, Chordata, Cnidaria, Ctenophora, Cycliophora, Echinodermata, Entoprocta, Gastrotricha, Gnathostomulida, Hemichordata, Kinorhyncha, Loricifera, Micrognathozoa, Mollusca, Nematoda, Nematomorpha, Nemertea, Onychophora, Orthonectida, Phoronida, Placozoa, Platyhelminthes, Porifera, Priapulida, Rhombozoa, Rotifera, Sipuncula, Tardigrada, and Xenacoelomorpha). Homolog protein sequence searches were performed through BLASTp algorithm with maximum number of target sequences set at 20,000 and by setting general parameters such as expected threshold (0.05), word size (6) (minimum number of characters required to seed a match between two sequences) and maximum matches in a query range (0) as default [87]. The scoring matrix BLOSUM62 and other scoring parameters such as gap costs and compositional adjustments were set as default in the search interface. Filters and masking parameters were not selected. Searches were performed independently at two different times by two authors (ST and IT) using the same search parameters to check search reproducibility. Initially protein sequences for each species identified by both authors from the BLASTp hits and annotated as 5-HT_3_ receptor subunit, 5-HT_3_ receptor-like subunit, unnamed, hypothetical, and uncharacterized were selected. Sequences were excluded based on Expect value (E-value), degree of sequence similarity, size of the protein sequence (Table 1) and only the best annotated sequence per species was selected. In the second step the selection was narrowed down so no more than 4 members per genus were selected from the sequences identified in the first step and the A subunit needed to have been identified for this species. This process was also done independently by two authors (ST and IT) and any differences were discussed with all three authors (ST, IT and HI) until a consensus was met. In the third step, sequences were selected for phylogenetic tree generation after performing the multiple sequence alignment using Clustal Omega as described below. Sequences were checked by the authors (ST and either HI or IT) to determine if the Cys-loop and each transmembrane domain were at least 20% homologous to the relevant human 5-HT_3_ receptor subunit. Sequences were checked to ensure that they were not mis-annotated 5HT3A subunits by aligning with human 5HT3A sequences. Any mis-annotated sequences were removed before further analysis (S2 Table). Sequences missing the entire Cys-loop (15 amino acids) and any transmembrane domain or sequences with less than 4 transmembrane domains were excluded (Table 1). A five-set Venn diagram for the number of A, B, C, D and E subunits present in the selected species was generated using interactive Venn tool [88].

**Table 1:** Values of parameters for sequence exclusion criteria.

Parameter	Value
**Exclusion criteria upon BLASTp search**	
E-value	>0.004
Percentage of sequence similarity	<40%
Size of the protein sequence	<100 aa
Maximum number of sequences per genus	4
**Exclusion criteria for phylogenetic analysis**	
Transmembrane domains	<4
Cys-loop and no transmembrane domains	0 and <1

### Sequence alignment and phylogenetic tree construction

The selected 5-HT_3_ receptor protein sequences from different species were aligned with Clustal Omega 1.2.2 [89] in Geneious Prime 2021 [90] last accessed 3 August 2022. The parameters for alignment with Clustal Omega 1.2.2, such as mBED cluster size was set as 100 with refinement iterations set as 0 and other settings as Auto. These alignments were exported to MEGA X software and subjected to phylogenetic tree construction using maximum likelihood and Neighbor-Join (NJ) and BioNJ algorithms to a matrix of pairwise distances estimated using a Jones-Taylor-Thornton (JTT) model. The other parameters such as substitution type (amino acid) rate among sites (uniform rates), site coverage cut-off (95%) with 500 bootstrap replicates were set as default [91]. The evolutionary history was inferred by using the Maxim Likelihood Heuristic method- Nearest Neighbour Interchange (NNI) and Branch swap filters were set to none. The trees were further edited and viewed with ITOL program [92] last accessed 7 September 2022.

### 5HT3A subunit sequence logos and protein structure modelling

Consensus sequence logo (graphical representation of an amino acid multiple sequence alignment) for 5HT3A subunit sequences for all selected species were generated by using WebLogo 3.7.4 web based application [93] last accessed 3 August 2022. The parameters for logo generation were as follows: 40 stacks per line, units were kept as bits. Scale stack widths, Error bars and Version fine print were selected, and colour scheme was changed to chemistry. Human 5HT3A (AAP35868.1) protein structure was modelled in Swiss model web-based application with default parameters [94] and last accessed 8 July 2022. Swiss model took the cryo-EM structure of mouse 5HT_3_A receptor [74] (PDB: 6np0.1) as a template to design the human 5HT3A (AAP35868.1) structure and the cryo-EM structure of mouse 5-HT_3_A receptor [95] (PDB: 6w1m.1) for 5HT3E (NP_938056.1) and the structures were captured using UCSF chimera X software [96].

## Phylogeny of 5-HT_3_ receptor subunits

### Identification of 5-HT_3_ receptor subunits in different species

To examine the evolution and divergence of the 5-HT_3_ receptors in different phyla of the animal kingdom, human 5-HT_3_ receptor A, B, C, D and E subunit sequences were used as query sequences to retrieve sequences of 5-HT_3_ receptor subunits through BLASTp searches. Although the 35 phyla in the metazoan lineage were included in the search, only species belonging to 11 phyla (Annelida, Arthropoda, Chordata, Cnidaria, Echinodermata, Mollusca, Nematoda, Orthonectida, Platyhelminthes, Rotifera, and Tardigrada) were identified to contain proteins at least 40% conserved with human 5-HT_3_ receptor subunits (S1 Table). The sequences were assessed to ensure that they met similarity criteria (Table 1) before being shortlisted as consensus sequences. Selection was further narrowed down, so no more than four members per genus were selected from the sequences identified to limit favouring of genera that have been more highly reported in the data bases. Since the A subunit can form functional receptor homomers and was initially thought to be the sole subunit and no other species have so far been observed to form functional pentamers without inclusion of an A subunit, species with annotated B to E subunits also needed to be on the A subunit list. Thus 526 5HT3A, 358 5HT3B, 239 5HT3C, 70 5HT3D, and 173 5HT3E subunit protein sequences were selected and downloaded (S2 Table). Clustal Omega 1.2.2 alignments of these sequences were undertaken to screen selected sequences for homology with the human subunit at the extracellular Cys loop and each of the transmembrane domains. Only sequences that contained >20% homology with the human subunit at transmembrane domains and the Cys loop were included in the final analyses (S2 Table). This reduced the number of orthologous sequences from different species analysed to 494 5HT3A, 299 5HT3B, 203 5HT3C, 36 5HT3D, and 147 5HT3E homologs (S2 Table).

**Fig 1:**
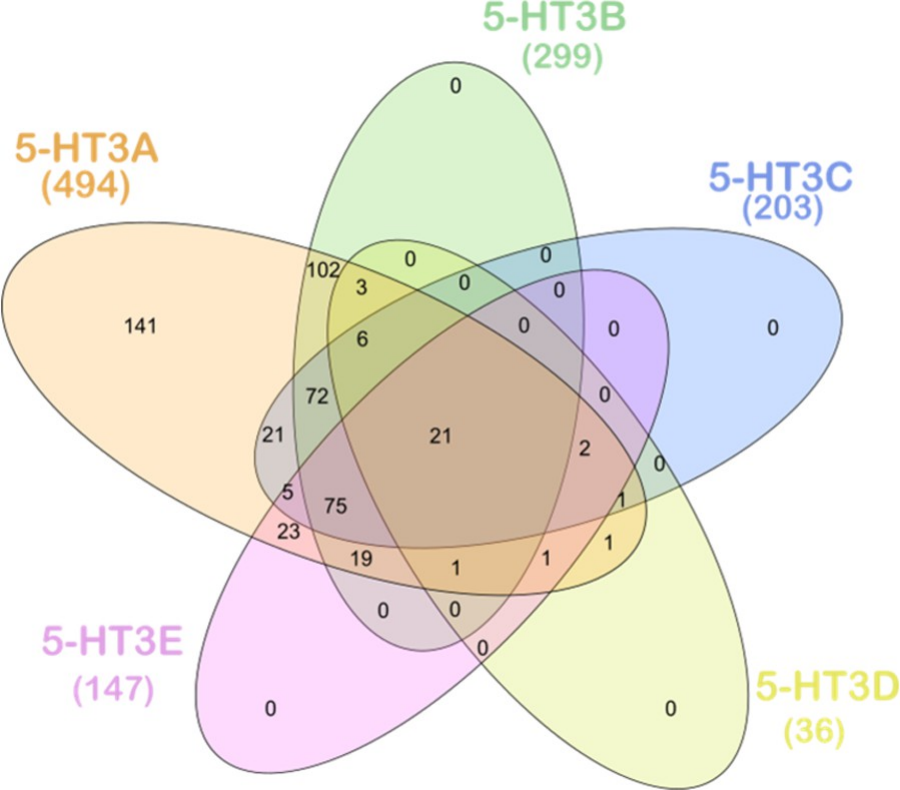
Venn diagram summarising the number of 5-HT_3_ receptor A, B, C, D and E subunit sequences identified in different phyla from the animal kingdom. A detailed breakdown of subunit distribution is listed in S2 Table. The figure contains the numbers of B, C, D and E subunits for those species that also contain subunit A, thus our dataset by definition contained zero (0) sequences with only B, C, D, or E subunits.

The greatest number of subunit homologs were found in Chordata phylum (S2 Table). Although some subunit homologs were annotated as uncharacterised or hypothetical or unnamed, they were kept for further analysis if they met the inclusion criteria described in Table 1. The distribution of the different 5-HT_3_ receptor subunits is represented in a Venn diagram (Fig 1) and highlights numbers of species where multiple subunits are present. Only those species with subunit A present were included in the B, C, D or E subunit datasets as described above. All five 5-HT_3_ receptor subunit homologs were detected in species from phyla Chordata, Mollusca, Platyhelminthes, Cnidaria; while Arthropoda, Annelida and Nematoda contain homologs of all subunits except the 5HT3D, and Hemichordata contain A and B receptor subunits (S2 Table). Only subunits A and E were identified in Echinodermata and Orthonectida, while Tardigrada contains A and D subunits, and A is the sole subunit found in Rotifera (S2 Table). A total of 20 species from phylum Chordata are predicted to have all subunits and, except for two species of the Panthera genus, these species are all from different genera (S3 Table).

Since we identified species with all subunits (S3 Table) from across Chordata with different dietary requirements and distinctive lineage splits [98], we were curious about the evolution of the 5-HT_3_ receptor subunits. We generated a phylogenetic tree for these species to see how the lineage and evolutionary pattern looks between the subunits (S1 Fig). Subunit A and B descended from the same ancestral lineage. Interestingly, the A and B genes are found on chromosome 11 (11q23) in humans. Similarly, primate species such as *Theropithecus gelada*, *Papio anubis*, *Pan troglodytes*, *Piliocolobus tephrosceles*, and *Rhinopithecus roxellana* have the A and B subunits on chromosomes 14, 12, 11, 13 or 15, respectively. Fish species *Perca flavescens* and *Etheostoma spectabile* contain A and B subunits on chromosome 13, *Camelus ferus* on chromosome 33, and carnivorous species such as *Felis catus*, *Ailuropoda melanoleuca*, and *Suricata suricatta* on chromosomes D1, 8, or 11 respectively. We could not obtain information about chromosomal location of the other species such as *Ceratotherium simum simum*, *Loxodonta africana*, *Trachypithecus francoisi*, *Hylobates moloch*, *Pongo abelii*, *Panthera pardus*, *Panthera tigris altaica*, *Vulpes vulpes*. Although it is inferred that the other subunits (C, D, or E) descended from same ancestral node, these subunits formed a separate sister branch with interruptions to the individual subunits. In humans, these subunits are found close together on chromosome 3 (3q27) and other primate species contain them on chromosomes 1, 2 or 3. These genes occur on chromosome 1 in *Camelus ferus* and on chromosome 5 in *Suricata suricatta*, while cat species *Felis catus* and *Panthera pardus* have them on C2 chromosome. However, the other species such as *Perca flavescens*, *Etheostoma spectabile* contain these 3 subunits on different chromosomes.

### Evolutionary relationship between 5-HT_3_ receptor subunits among metazoan linages

Homologs of each subunit were aligned using Clustal Omega and these alignments were used to generate phylogenetic trees where the reliability of each tree was estimated using the bootstrap method (500 bootstraps).

#### 5HT3A subunit

**Fig 2:**
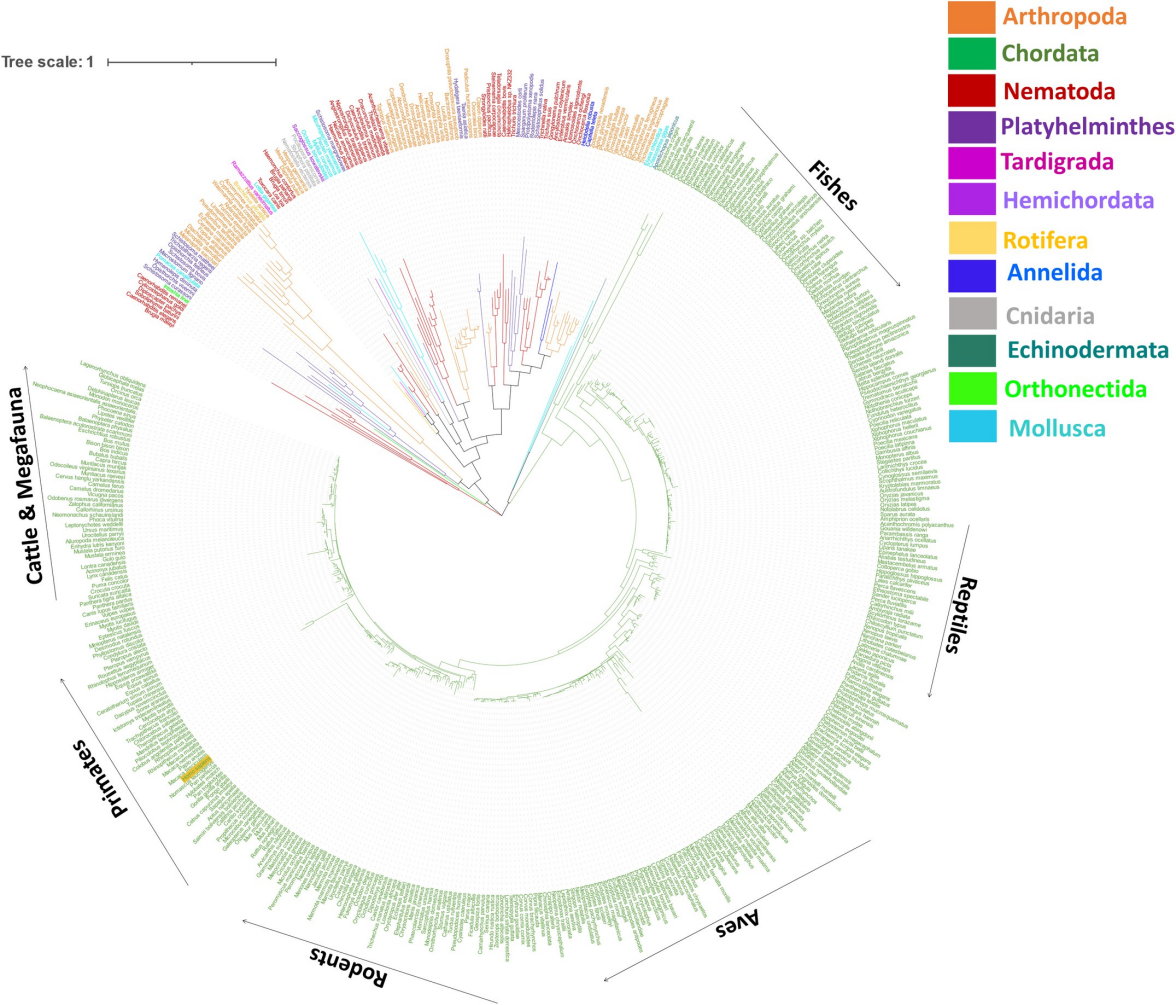
Phylogenetic tree of 5HT3A homolog proteins from animal phyla. The colours of the species in the tree correspond to the colours of the phyla in the figure legend: Chordata (green with the human sequence highlighted with amber background), Nematoda (dark red), Arthropoda (orange), Platyhelminthes (dark purple), Mollusca (cyan), Rotifera (yellow), Tardigrada (maroon), Hemichordata (purple), Orthonectida (pale green), Echinodermata (teal), Annelida (blue) and Cnidaria (grey). This analysis involved 494 amino acid sequences. There was a total of 292 alignment positions in the final dataset. Evolutionary analyses were conducted in MEGA X and tree editing was performed in iTOL. Tree scale represents the number of differences between sequences.

A total of 494 5HT3A homolog protein sequences were used to generate the phylogenetic tree based on 292 alignment positions (conserved regions) in the final dataset. The root of the tree divides into 3 major clades with bootstrap support ranging from 76 to 100 at the branches (Fig 2). The first clade contains species from Nematoda including two members of genus Caenorhabditis. The second clade divides into several sister groups of different phyla including Annelida, Arthropoda, Cnidaria, Mollusca, Nematoda, Orthonectida, Platyhelminthes, Rotifera, Hemichordata and Tardigrada. This clade includes many species from Arthropoda, Nematoda, and Platyhelminthes; frequently Arthropoda and Nematoda share the same ancestral node. Most of the species from the phylum Arthropoda are wasps and ants that live on plant saps [99]. Platyhelminthes and Nematoda also form sister clades and the representative species here are parasites of the gastrointestinal tract. This is an interesting observation as the gastrointestinal tract in humans, pigs, horses, and ruminants contains over 90% 5-HT present in the organism [100, 101]. The third clade mainly contains species from phylum Chordata supplemented with a few species from Mollusca and the single species from Echinodermata shares the same ancestral node. Human (primate) and rat (rodent) 5HT3A homologs share 84% sequence similarity reflecting their divergence from the same ancestral node in the tree. All the ancestral species were placed at the start of the tree which represents a possible evolutionary pattern of the 5HT3A subunits from species like *Apostichopus japonicus* (PIK58946.1—spiky sea cucumber) to *Homo sapiens* (AAP35868.1—human) and *Orcinus orca* (XP_004273432.1- killer whale) at the end of the tree. The pattern in the phylum Chordata (3^rd^ clade) starts at Fishes–Reptiles—Aves (nonflying birds to flying birds)—Rodents and then Primates. Most of the carnivorous species and large-bodied animals (megafauna) such as elephants, orcas, and cattle species (bison, cow) occur at the end of the tree after primates.

#### 5HT3B subunit

The phylogenetic tree of 5HT3B homologs contains 299 sequences with 324 alignment positions in the final dataset. Although it has three major clades with bootstrap support ranging from 78 to 99 at the branches, there are some surprising differences compared to the tree of 5HT3A homologs (S2 Fig). The first and third clades are monophyletic clades containing species from Chordata, while the second clade contains all the other phyla. The first clade contains only four species of fishes that have hypothetical or uncharacterized 5HT3B homologs. Only one species from Hemichordata is in the second clade, while species from Mollusca, Platyhelminthes, Cnidaria, Nematoda and Annelida form sister branches. Notably a few chordate species belonging to the class Aves share the same ancestral node with lower species other than Chordata and these Aves species were manually removed from the tree (S2 Fig). Similarities between the pattern of 5HT3B homologs and 5HT3A homologs is seen in the third clade, starting from Fishes–Reptiles—Aves (nonflying birds to flying birds)—Rodents and then Primates and carnivores to large-bodied animals (megafauna). No 5HT3B sequences from species in the phyla Tardigrada, Echinodermata, Rotifera and Orthonectida met the inclusion criteria.

#### 5HT3C subunit

Two major clades are seen in the phylogenetic tree of the 203 5HT3C homologs with 277 alignment positions in the final dataset with bootstrap support ranging from 76 to 100 at the branches. Three species sequences from Chordata phylum *Alligator mississippiensis* (KYO43969.1), *Melospiza melodia maxima* (KAF2983139.1) and *Scyliorhinus torazame* (GCB64712.1) were removed manually due to their placement inaccuracy (they were annotated as hypothetical and uncharacterised proteins from Chordata and were placed in the middle of the non-Chordata phyla). The first clade contains Annelida as the first diverging phyla followed by Nematoda, Mollusca, Cnidaria and Platyhelminthes, Arthropoda (S3 Fig). The second clade contains only members of Chordata where fishes descend from the same ancestral lineage as Reptiles, Aves, and Primates. Like the 5HT3A homologs tree, the Chordata phylum for 5HT3C homologs show a pattern from fishes, primates, and carnivores to large-bodied animals (megafauna). Phylum Hemichordata does not contain 5HT3B and 5HT3C subunits.

#### 5HT3D subunit

5HT3D has the smallest extracellular region and lacks half of the signature Cys-loop [39, 80, 81]. Although subunit D involvement in the structural and functional activities of the 5-HT_3_ receptor has been questioned due to the lack of a signal peptide and most of the extracellular domain [97], experimental studies have shown subunit D expression and function in the 5-HT3AD heteromeric receptors [80, 81, 102, 103]. We were able to download only 70 protein sequences in total with 36 sequences meeting the inclusion criteria (S4 Fig). The 5HT3D homolog tree forms 3 major clades with one clade containing species from several phyla, while the other two clades mainly contain species from phylum Chordata. The bootstrap support for the branches ranging from 72 to 99 in the tree. The first clade contains several fish species from Chordata. The second clade contains species from phyla Cnidaria, Tardigrada, Mollusca and Platyhelminthes, including some Chordata species and the third clade contains species from phylum Chordata. One molluscan species *Mytilus coruscus* (CAC5413744.1) and a species from Tardigrada *Ramazzottius varieornatus* (GAV02417.1) descends from the same ancestral node and forms a separate sister branch in the third clade. The first branch in the third major clade contains fishes and frogs while the other branch contains primates, carnivores, and megafauna sequences. Species from phyla Annelida, Nematoda and Arthropoda lack subunit D.

#### 5HT3E subunit

The 5HT3E homolog tree contains 144 sequences forming two major clades with bootstrap support ranging from 78 to 99 (S5 Fig). The first clade contains species from Orthonectida as the first diverging phyla; followed by Mollusca, Platyhelminthes, Echinodermata, Cnidaria, Nematoda, Arthropoda and Annelida. The second clade contains species from Chordata and the evolutionary pattern is similar to the 5HT3A homolog tree. Fishes and reptiles diverged first as a single branch while primates, carnivores and large-bodied animals descended as the second sister branch from the same ancestral node. Although *Etheostoma spectabile* (KAA8586554.1), *Erythrura gouldiae* (RLV82886.1) and *Phyllostomus discolor* (KAF6129353.1) meet inclusion criteria, they were excluded from the tree manually due to their placement inaccuracy (their taxonomic positions were placed in the clade with phyla Nematoda, and they are annotated as hypothetical or uncharacterized proteins in the data base).

### Variation in the 5-HT_3_ homomer receptor structure

**Fig 3:**
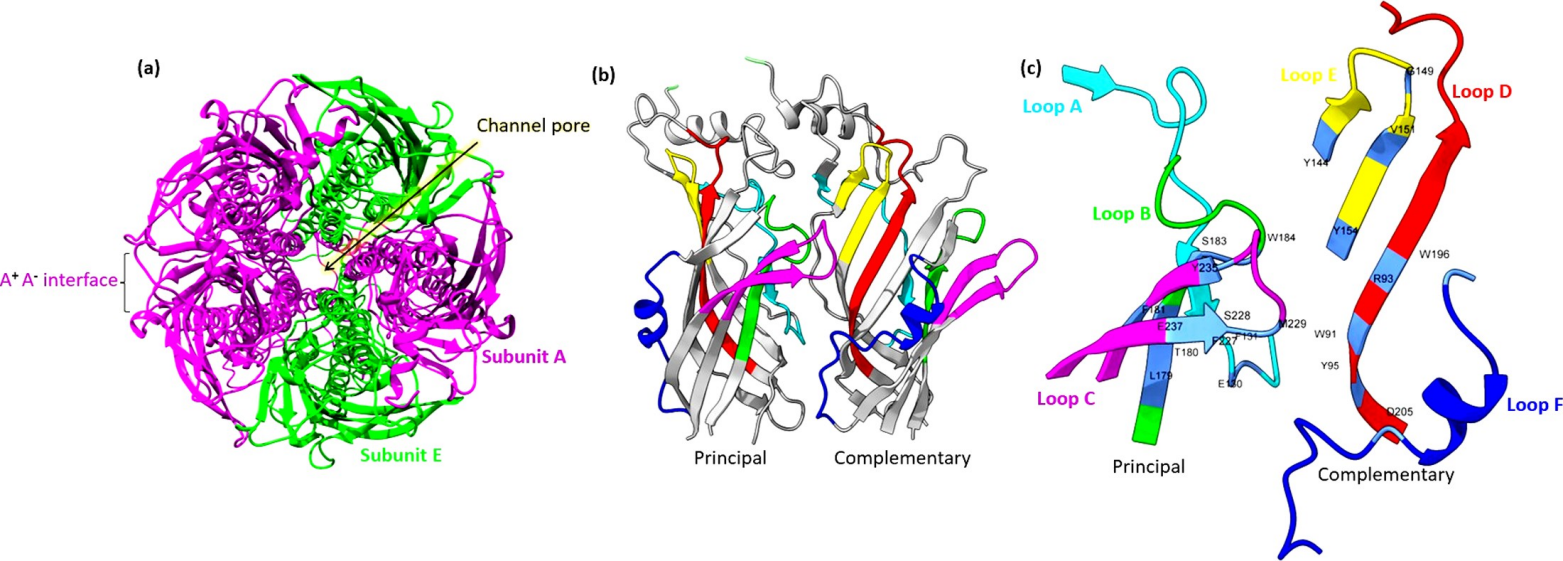
Homology models of heteromeric 5-HT_3_AE receptors **(a)** Extracellular view showing 5-HT_3_AE receptor heteromer formed with three A (maroon) subunits and two E (green) subunits with AAEAE stoichiometry (based on the AABAB stoichiometry [114]) where A+A- interface (ligand binding region) is indicated. **(b)** The A+A- extracellular interface of two adjacent subunits (principal subunit (A+) and complementary subunit (A-)) highlighting the six loops that converge to form the ligand binding site. Only two of the five subunits have been shown for ease of viewing. c) The loops in the principal subunit (loop A (cyan), B [115], C (pink)) and the complementary subunit (loops D (red), E (yellow), F (blue)) and the critical amino acid residues participating in the binding site are labelled. Human 5HT3A and 5HT3E subunit tertiary structures modelled using information from mouse 5HT_3_A receptor structures with PDB 6np0.1 [74] and PDB 6w1m.1 respectively by Swiss modelling software [94] and further edited in by UCSF Chimera software [116]. Residue numbers of the human 5HT3A (AAP35868.1) subunit (Fig 4) are used as the comparator in the following sections. Each 5HT3A subunit contains an extracellular N terminal helix (M1-P21 residues) signal peptide that helps in receptor translocation to the plasma membrane [117, 118]. The remainder of the extracellular region contains multiple beta strands with the orthosteric ligand binding site formed by β1 – β10 strands and six loops (T87-K239) followed by the signature Cys-loop (C163-C177) [97]. This sequence then leads into the four sequential transmembrane (TM) domains: TM1 (V252-L272), TM2 (R279-D299), TM3 (C318-K338) and TM4 (K457-W478). The intracellular loop (ICL) between TM3-TM4 (L341-L455) is involved in channel gating activities [40, 119, 120]. Resistance to inhibitors of cholinesterase 3 (RIC-3) a chaperone protein binds to the RIC-3 binding region present in between TM3 and TM4 domains to help in receptor translocation to cell membranes [121–123].

We used 5HT3A subunit homologs to examine the conservation of different protein domains and ligand binding sites between phyla. The mouse 5-HT_3_A receptor was first crystallised [104] and later single-particle cryo-electron microscopy (Cryo-EM) studies revealed resting conformation and details of setron binding that inhibit 5-HT_3_A homomeric receptors [73, 74]. 5HT3A subunit orthologs of mouse and human share 84% sequence identity [105]. In mammals, 5HT3A subunits can form a homomeric pentamer to create an active ligand gated ion channel [106, 107]. The orthosteric ligand binding site forms at the interface of two A subunits (A+ (principal subunit) and A- (complementary subunit)) and binds agonists and competitive antagonists (Fig 3B and 3C) [42, 71, 108–113]. Voltage-clamp fluorometry (VCF) studies with different classes of agonists and antagonists (setron-class) on human 5HT_3_ receptors have shown that loops C, D and E play important roles in ion channel conformation changes [111].

**Fig 4:**
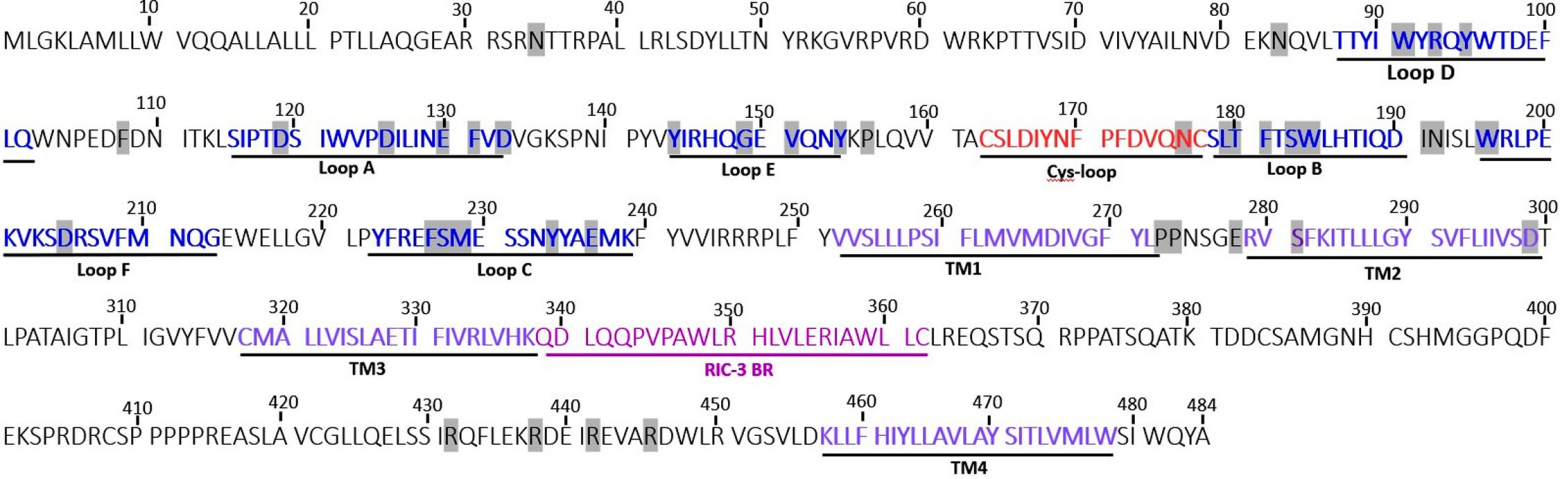
Amino acid sequence of the human 5HT3A subunit (AAP35868.1). The Cys-loop is marked in salmon, the extracellular loops are labelled in blue and transmembrane (TM) domains are depicted in purple, RIC-3 binding region (RIC-3 BR) depicted in pink. Functionally important amino acids as described in Table 2 are highlighted in grey.

**Table 2:** Human 5HT3A subunit (AAP35868.1) extracellular domain amino acid residues that participate in functional activity of the receptor.

Ligand	Critical residues *	Position	Functional significance	Reference
[3 H] granisetron	Y95	Loop D	Participate in forming hydrogen bond with ligand	[131–133]
granisetron	S228, M229	Loop C	Donates H‐bonds to the carbonyl groups of setrons	[85, 134]
[3 H] granisetron, granisetron, palonosetron, ondansetron	Y144, G149, V151 and Y154,	Loop E	Aromatic sidechains of these residues participate in π- π bond formation thus help planar rings of antagonists to intercalate between these residues	[134–137]
granisetron, tropisetron, ondansetron	Y235, E237	Loop C	Interact with tertiary ammonium ions of setrons and forms salt bridge (cation‐π interaction)	[85, 134, 138, 139]
[3 H] granisetron	W91, R93	Loop D	Indazole ring of granisetron interacts with R93 and the tropane ring interacts with W91	[72]
[3 H] granisetron	L179, T180 and F181, S183, W184, I191	Loop B	Contributes hydrophobic core that faces into the beta-sandwich which maintains the local structure of loop B	[72, 137]
[3 H] granisetron	W196 and D205	Loop F	Participate in forming hydrogen or ionic bond with ligand	[72]
[3 H] granisetron	P171	Cys-loop	Prolyl peptide bond (cis conformation) participates in pore opening	[126]
5-HT	E130, F131	Loop A	Ionic or hydrogen bond interaction with the primary ammonium group of 5-HT	[140, 141]
5-HT	F227, Y235	Loop C	Participate in forming hydrogen bond with ligand	[142]
5-HT	Y154	Loop E	Participate in forming hydrogen bond with ligand	[142]
mCPBG	W184	Loop B	Participate in forming hydrogen bond with ligand	[143]
5-HT	F108	Post loop D	Participate in forming hydrogen bond and recognition of the ligand	[141]

* Amino acid numbers given according to the sequence in Fig 4

**Fig 5:**
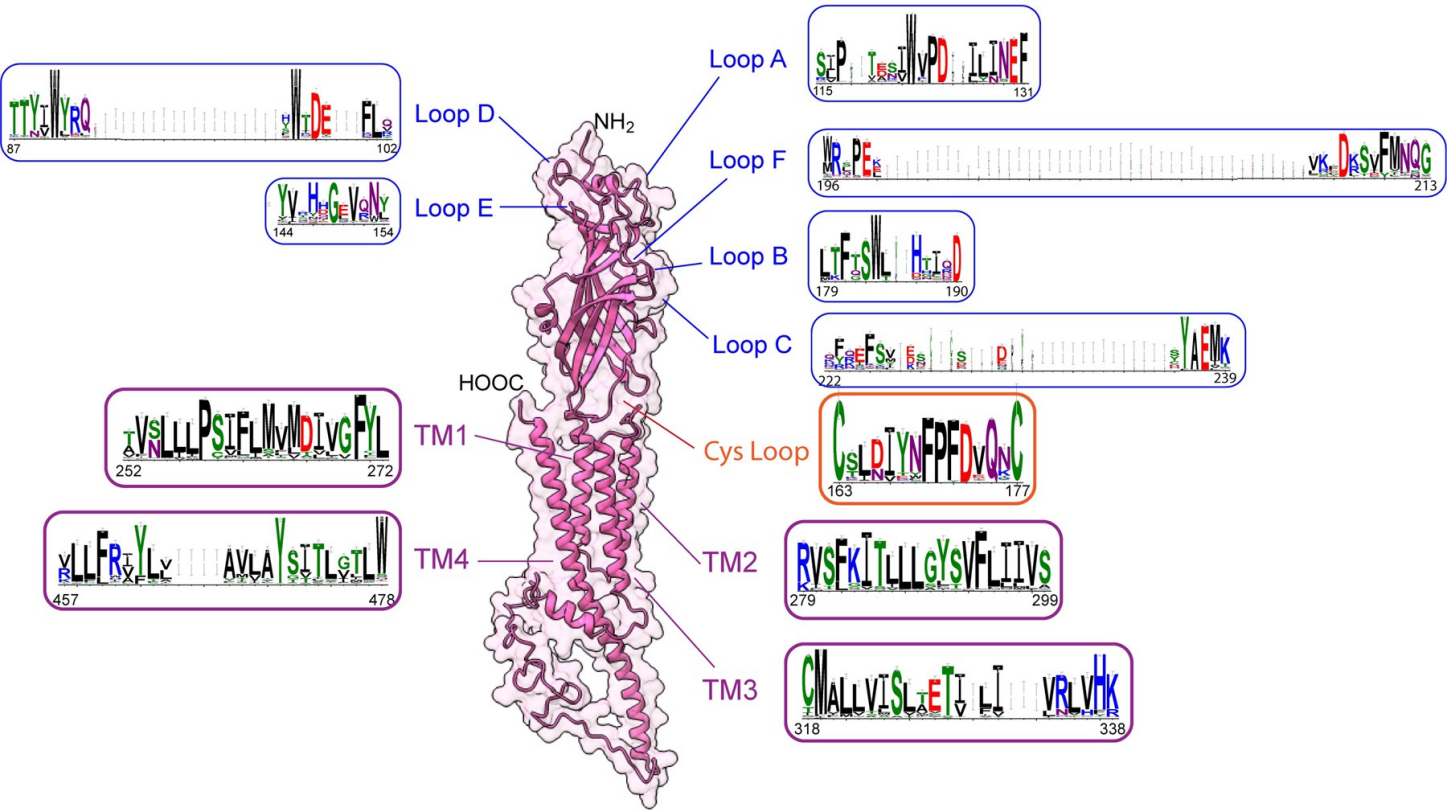
Consensus amino acid logos of 5HT3A subunit regions derived from 449 different animal species. Human 5HT3A subunit tertiary structure is modelled using information from PDB 6np0.1 using Swiss modelling [94] software and edited in UCSF Chimera software [116]. Sequence logos of loops A to F, TM domains and Cys-loop represented according to Table 2 and Figs 4 and S6 and S7.

Finally, the subunit ends with a short extracellular carboxy terminus. Conservation of different regions of the 5HT3A subunit across phyla is shown by consensus sequence logos using our final subset of 449 5HT3A subunits (Figs 5 and S7). Sequences with an extended N terminal (32 sequences) and C terminal (9 sequences) were removed from the 494 sequences used for phylogenetic analysis (details can be found in S1 Table) to generate Clustal Omega alignment with reduced gaps in the ligand binding domain (S6 Fig) used to create the consensus sequence logo (S7 Fig). The consensus sequence logos (Figs 5 and S7) provide a graphical representation of the degree of conservation. The sequence conservation is proportional to the overall height of each stack of letters (amino acid residues), measured in bits. The highest degree of conservation is shown by >4 or 4 bits while mid-range conservation by 2 bits, and low conservation by between 0 < 1 bits (S7 Fig).

Binding at the orthosteric site can cause conformational changes in the whole receptor [111, 124]. This affects how the loops at the lateral portals and TM domains align to form the aqueous pore with a constricted vestibule in the closed state (antagonist binding) and wide vestibule in the open state (agonist binding) [73, 97, 125]. TM2 faces the pore and is an essential player in moving ions through the pore [109, 115, 124, 126].

### Critical amino acids in the orthosteric ligand binding site

The ligand binding sites for 5-HT_3_ receptor are located near or at the interface of the two adjacent A subunits in the extra cellular domain (Fig 3) [85, 97]. Specific amino acid residues critical for binding competitive ligands at the A+A- interface in human 5-HT_3_A receptor homomer are summarised in Table 2. These residues occur in loops B, C, D, E and the Cys loop where they each make unique contributions to ligand binding by the receptor. These loops play critical roles in forming noncovalent interactions with ligands and participate in receptor pore opening and ion exchange (Fig 3) [85, 86]. In the present study we discuss amino acid substitutions in terms of the physico-chemical effects that are altered by amino acid residue substitutions between phyla [127–130].

For ease of comparison, we use residue numbers of the human 5HT3A (AAP35868.1) subunit (Fig 4) as the reference point for the remainder of this discussion. Residues equivalent to W91, G149, V151, F181, W184, D205 and E237 residues are highly preserved while R93, S228, Y144, Y154, T180, W196, and Y234 are less strongly preserved throughout 5HT3A homolog sequences (Fig 5). For example in loop D, W91 participates in interactions with the tropane ring while R93 interacts with the imidazole ring of setron antagonists [72]. F108 located just following loop D is important for serotonin recognition [141]. Y144, G149, V151, and Y154 (loop E) and Y235 (loop C) participate in π-π bond formation that helps the planar rings of antagonists to intercalate between these residues [134–137]. S228 and M229 (loop C) and E130 (loop A) interact with the carbonyl groups of the setron antagonists [85, 134]. P156 just beyond loop E is important for pore opening. Residues D119 and D133 (loop A) influence channel conductance [144]. W184 (loop B) and E237 (loop C) form cation−π interactions with ammonium groups [72, 138]. While L179, T180, F181 and S183 (loop B) are involved in shaping localised loop B structures [72, 137]. D205 (loop F) is another preserved residue across phyla that plays a critical role in ligand recognition and also participates in ligand binding via hydrogen bonding [72].

Several amino acids present in the extracellular domain assist in protein translocation and stability. For example, asparagine residues N34, N83, N176 and N192 are N-glycosylated during post-translational modification [145]. In addition, a 24 amino acid long segment in 5HT3A intracellular domain between TM3 and TM4 is important in binding the RIC-3 chaperone to help in trafficking the 5-HT_3_ receptor to the plasma membrane [77, 121–123, 146]. However, this appears to be a motif interaction as no specific individual amino acid residues have been clearly indicated to influence the chaperone binding.

Since we have observed high conservation of the critical residues in the phylum Chordata indicating conformity in ability to bind 5-HT and various 5-HT_3_ receptor ligands, we investigated if there were patterns occurring where the residues were less conserved (S4 Table). We discuss the loops in order from the N terminal sequence (Fig 4). Loop E is the only loop highly conserved and with no gaps throughout the alignment (Figs 5 and S7). Although loop E is the shortest loop, itis quite important as mutations at G149, V151 and Y154 abolish receptor ligand binding activity [147–149]. Most residues in loops A and B were also highly conserved although gaps were present that are omitted in Fig 5 as residues corresponding to V132 and D133 or S178 were not included in the logo. These gaps can be viewed in S7 Fig.

The gaps in loop D are generated by species from Chordata, *Ophiophagus hannah* (ETE72600.1) with 19 and *Limosa lapponica baueri* (PKU35975.1) with 6 amino acid long inserts. In loop D W91, R93 and Y95 are the important residues (Fig 5 and Table 2). The aromatic amino acid W91 in loop D participates in the binding pocket and is conserved throughout all the phyla. However, some species from Platyhelminthes, Nematoda, Mollusca have hydrophobic or aromatic amino acid substitutions such as L or Y and F, respectively and a species from Cnidaria has a C instead of W91. The basic amino acid R93 is present in species from phyla Chordata, Cnidaria, Mollusca, Tardigrada and Echinodermata (S4 Table). In Nematoda and Arthropoda a basic substituent of K occurs sometimes but residues T, Q and V can also occur, and in some cases, the acidic substituents D and E are present. While species from phyla Annelida and Rotifera have T and D respectively at the position of R93. Y95 has substitutional variation throughout the phylum Chordata with the residues Q, E, H, F and S present, while the other phyla lack the Y95 residue in their protein sequence (S4 Table).

The first part of loop A strongly aligns between the various homologs with a gap present before the final two residues V132 and D133 (Figs 5 and S6 and S7). This gap is due to amino acid inserts ranging from two to 18 residues in *Haemonchus placei* (3 residues), *Brugia pahangi* (18 residues), *Brugia timori* (7 residues) belonging to Nematoda and Camelus dromedarius (2 residues) and *Tetraodon nigroviridis* (7 residues) belonging to Chordata (S6 Fig). Loop A contains the functionally important acidic residues, E130 and D133, where D133 is highly conserved throughout the alignment. Only a few species from Chordata (T) and Nematoda (M) have variations with different physico-chemical properties at D133 position. E130 was only found in and is highly conserved throughout the phylum Chordata and species from phylum Nematoda contain residue Q as substituent for E130, while species from phylum Platyhelminthes, Orthonectida, Arthropoda, Mollusca, Cnidaria, Annelida, Tardigrada, and Echinodermata lack an equivalent or substitutional residue for E130. Residue F131 is conserved in phylum Chordata and species from Nematoda have a non-conservative substitution with residue N at F131 position while other phyla do not contain F131 in their protein sequence.

Loop E has the amino acids Y144, G149, V151 and Y154 that participate in antagonist intercalation [134–137]. G149 is highly conserved throughout the alignment, while Y144, V151 and Y154 are highly conserved in phylum Chordata but vary in other phyla. Notably, Y154 has conserved substitutions with S and T residues and non-conserved substitutions with K, V, L, G, I, Q, E, P, D residues in the lower phyla (S6 Fig).

Critical amino acids in loop B are L179, T180, F181, S183 and W184, as they maintain the local structure of loop B by contributing a hydrophobic core that faces into the beta-sandwich of the receptor structure. W184 (loop B) is highly conserved throughout the 5HT3A subunit alignment except for phyla Mollusca (T, M), Platyhelminthes (L) and Echinodermata (Q). Substitutions with similar physico-chemical properties for L179 occur in species from Arthropoda (M), Nematoda (F), Platyhelminthes (I), and Echinodermata (F), while some species from Chordata have R at this position. T180 exhibits a high number of variations amongst Rotifera, Cnidaria, Arthropoda and Tardigrada having K at this position, Platyhelminthes (D), Nematoda (I, Q), and Echinodermata (F). Whereas F181 and S183 are highly conserved in Chordata and are generally conserved throughout the other phyla (Fig 5). A species from phyla Nematoda *Ancylostoma caninum* (RCN52111.1) has a 19 amino acid long insert after S178 and is the reason for the gap in Loop B (S6 and S7 Figs) that is not shown in Fig 5 due to the high conservation of the remaining residues.

Residues W196 and D205 in loop F are important in ligand binding in humans, and D205 is conserved across all phyla (Fig 5). A species from phylum Arthropoda, *Hyalella azteca* (KAA0187152.1) has a 39 amino acid long insert in Loop F. Most species from phyla other than Chordata have 4 to 16 amino acid long inserts following W196 while one species from Chordata, *Phaethon lepturus* (XP_010287046.1), has a 5 amino acid insert in Loop F.

F227, S228, M229, Y235 and E237 are the functionally important amino acid residues in loop C; S228 shows mid-range conservation at 2 bits being somewhat conserved among all the phyla (S3 Table and Table 2 and S7 Fig). Among all the critical residues in loop C, M229 is the least conserved residue throughout the alignment. Notably, Y235 and E237, which are important for both agonist and antagonist binding, are highly conserved throughout the Chordata. Only one species from Nematoda *Caenorhabditis elegans* (NP_509270.1) contains these residues while the remaining phyla lack Y235 and E237 in our data set. Most species from phyla other than Chordata have 4 to 6 amino acid long inserts that create the first gap in the sequence logo of loop C. The second gap was created by a 17 amino acid long insert in loop C from species *Temnothorax longispinosus* (TGZ32403.1) that belongs to phylum Arthropoda.

### Evolutionary variation of Cys-loop and transmembrane domains

The Cys-loop and TM domains form part of the signature sequences for 5-HT_3_ receptors [109, 115, 126, 150], so not surprisingly show high conservation across the phyla (Fig 5).

### Variations in Cys-loop

Most species from Chordata, Nematoda, Arthropoda, Mollusca, Cnidaria, Tardigrada, Platyhelminthes, Echinodermata, and Hemichordata contain the key ingredients of the Cys-loop, C163 and C177 (numbers represent position of Cysteine residues in Cys loop of human 5HT3A sequence (Fig 4)). *Litomosoides sigmodontis* is an exception as it only contains residues from F170 to C177. Our search also included several species from Chordata without a Cys-loop, but these sequences did contain four TM sequences thereby meeting the selection criteria (*Ursus maritimus*, *Cercocebus atys*, *Grammomys surdaster*, *Melospiza melodia maxima*, *Gavia stellata*, *Pygoscelis adeliae*, and *Tauraco erythrolophus*). Interestingly, a Nematoda species, *Ancylostoma caninum*, contains a 19-residue long insert followed by Cys-loop which created a large gap in the alignment at loop B (S6 and S7 Figs). The Cys-loop in Chordata is highly conserved and invariably contains polar uncharged amino acids serine (S) or threonine (T) present in the second position while species from Arthropoda (E, K, P, G, D), Nematoda (I, P, Q), Mollusca (M) and Annelida (P) contain non-conserved substitutions. Amino acid residues F170, P171, F172 and D173 in the Cys-loop were highly conserved throughout the alignment in the Metazoa (Fig 5). The hydrophilic—acidic amino acid D166 was substituted by amino acids N and H in phylum Chordata and H in Mollusca. Platyhelminthes have a conservative acidic substituent E or a nonconservative substitution of Q while Echinodermata have the nonpolar amino acid L in this position. Amino acid I167 is conserved throughout Chordata phylum, however substituents in some species in phyla Nematoda, Arthropoda and Platyhelminths have residues with similar physico-chemical properties (V and M), and species from phylum Mollusca contain the polar residue T (Figs 5 and S6). Residue Y168 was substituted with amino acids with different physico-chemical properties in Nematoda, Arthropoda, Annelida, Mollusca and Chordata (residues W, F, H, K and N, respectively).

### Variations in transmembrane domains

To function, the receptor needs to assemble with five subunits, each containing four TM domains, embedded in the lipid membrane (Fig 5) [107]. The 5HT3A subunit TM1 domain (V252-L272) is highly conserved throughout homologs of Chordata phylum. Several homologs from phylum Arthropoda contain F substituents for V252 and V253. S254 is present in many Chordata species however, S254 is replaced by N in other phyla including some of Chordata bird and fish species. Notably, P258 is perfectly conserved throughout the metazoan lineage. The hydrophobic residues spanning F261 to M265 are conserved in Chordata phylum.

The 5HT3A TM2 domain (R279 to D299) lines the channel pore and shows strong alignment across the phyla and is highly conserved throughout the phylum Chordata. Polar charged amino residue E278 is located just before TM2 and contributes to the intermediate ring of the pore. Residues D299 and S281 form part of the channel lining. Annelida and Arthropoda have a conservative substitution with T in the position of S281. Nonconservative substitutions occur in Platyhelminthes (M), Mollusca (G), and Nematoda (A or G) in the position of S281. The TM2 domain also contributes to the extracellular ring and the polar central ring promoting cation conduction in the receptor [124]. L287, L288 and F293 are conserved throughout the alignment whereas other amino acid residues show variations (Fig 5).

The 5HT3A TM3 domain (C318—K338) shows reasonable alignment among all the species and is most highly conserved amongst the phylum Chordata. Species from phylum Chordata *Notothenia coriiceps*, *Takifugu bimaculatus*, *Cyprinodon variegatus* and a species from phylum Nematoda *Soboliphyme baturini* do not have TM3 in their protein sequence. However, they met inclusion criteria as they contain the Cys-loop and TM1 and TM2 in their protein sequence. A species from phylum Nematoda *Brugia malayi* (VIO86814.1) has a three amino acid long insert generating a gap in the alignment. Residues corresponding to C318 are mostly conserved in phylum Chordata. Residues at this position are substituted by I and T in Arthropoda and Annelida, while Platyhelminthes contain N, L, G residues. The basic R334 is conserved in homologs of the Chordata phylum while in other phyla it is replaced by C, H, N, or Q residues. M319 (F, I), H337 (F, Y) and K338 (R, G, Q, H) residues also show variation in the alignment (Figs 5 and S6).

TM4 (K457 to W478) is also conserved with the gap in the alignment due to a sequence from a bird species *Melospiza melodia maxima* containing a four residue insert. Residues F460 and Y470 are mostly conserved in the phylum Chordata (Fig 5). Some of the species from Arthropoda, Nematoda and Platyhelminthes have alternate hydrophobic amino acid residues, L or M, in the position of F460. A single species from Platyhelminthes, *Hymenolepis diminuta* (VUZ42516.1), contains Y470 and the remainder of Platyhelminthes in our data set lack Y470 (S6 Fig). L459 from TM4 was conserved throughout the metazoan lineage, however, a few species from Nematoda have S, Arthropoda has F and a species from Tardigrada has T instead of L459. The hydrophobic amino acid A466 shows some variations in Chordata (T), Arthropoda (F), and Nematoda (V and C). Overall, most residues in the transmembrane domains show a high degree of conservation between 3 and >4 bits. Together with the relatively small gaps of 3 and 4 residues long in TM3 and a 4 residue gap in TM4, this reflects the great conservation of transmembrane domains throughout species in the Metazoa.

### Intracellular loop between TM3 and TM4

5-HT_3_ receptor ion channel conductance is highly influenced by four arginine residues (R432, R438, R442, R446) in the large intracellular loop between TM3 and TM4 loop of the A subunit [119]. The R432and R438 residues were conserved throughout the A subunit homologs from Chordata phylum (S6 Fig). However, our alignment across the entire 5HT3A subunit (S6 Fig), revealed that only a few species from Nematoda, Platyhelminthes and Arthropoda contain these residues. For example, species from Arthropoda—*Daphnia pulex* and *Vespula pensylvanica* have an acidic E at R432 in their protein sequence. Species from Rotifera–*Brachionus plicatilis* are substituted by residue H at R432, and non-conservatively substituted by residue E at R438 and R442. Most species contain polar uncharged T or hydrophobic A at R432 position. In Chordata, R446 can be conservatively substituted with residue K, or in some species with the acidic amino acids E and D. Other phyla contain both conservative substitutions (K) and various non-conservative substitutions of acidic or hydrophobic amino acids at positions R432, R438, R442 or R446 (S6 Fig). The intracellular loop of the 5HT_3_ receptor shows multiple gaps throughout the alignment with most residues showing low degrees of conservation scoring between 0 and 1.5 bits. Therefore, it appears that the intracellular loop is one of the least conserved regions in Metazoa, like other ligand gated ion channels.

A 24 amino acid long segment at the beginning of the intracellular domain is involved in RIC-3 chaperone binding [121–123]. This segment is conserved in the phylum Chordata and shows strong alignment throughout the metazoan lineage. A few species have inserts in the segment. The Nematoda species, *Haemonchus contortus* (CDJ96026.1) and *Brugia malayi* (VIO86814.1) have two and five amino acid long inserts respectively, and another species from Platyhelminthes *Schistosoma curassoni* (VDP38785.1) has a 2 amino acid long insert generating gaps in the alignment. Species from Mollusca *Crassostrea gigas* (XP_034309618.1), Echinodermata *Apostichopus japonicus* (PIK58946.1), and Platyhelminthes *Hydatigera taeniaeformis* (VDM17286.1) have single amino acid inserts.

### Variations in other amino acid residues in 5-HT3A subunit important in receptor function

The extracellular asparagine residues N34, N83, N176 and N192 are glycosylated during post-translational modification and are conserved in the phylum Chordata [145]. The residue N83 is highly conserved throughout the alignment, although in some species from Nematoda it is substituted by D. The residues N176 and N192 were generally replaced by various polar residues in phyla other than Chordata. However, most of the species from metazoan linage have at least one extracellular site for potential glycosylation in their protein sequence (S6 Fig).

The aromatic residue F108 present after loop D is important for serotonin recognition [141] and this residue is mostly conserved throughout the metazoan lineage. One species from Chordata *Melospiza melodia maxima* (KAF2977017.1) does not contain F108. In addition, some of the species from Arthropoda, Nematoda, and Platyhelminthes are substituted with the aromatic amino acids tyrosine (Y) or tryptophan (W). P273 and P274 in the TM1-TM2 intracellular loop participate in pore opening [126] and both residues are conserved in phylum Chordata. The residue P273 is highly conserved throughout the Metazoan lineage. Although P274 is strictly conserved in phylum Chordata, some species from phyla Arthropoda (A, D), Nematoda (S, T, H, I), and Mollusca (V) exhibit substitutions. Only one species from phylum Nematoda, *Halicephalobus sp*. *NKZ332* (KAE9548540.1), does not contain either P273 or P274 (S6 Fig).

## Discussion

The 5-HT_3_ receptor is a therapeutic target in both human and veterinary medicine where antagonists are used predominantly to alleviate chemotherapy or operational induced nausea and vomiting [47, 48]. Potentially organisms present in the surroundings of human ecosystems including various parasites (tapeworms, round worms, insects) can be affected by the 5-HT_3_ receptor antagonists and their metabolites. Therefore, a need exists to understand the breadth of potential organisms that can be affected by 5-HT_3_ receptor ligands. Prior reports indicated that some species from Arthropoda, Mollusca and Annelida are responsive to 5-HT_3_ receptor antagonists, but molecular details are lacking [43–46]. We examined the evolutionary relationships of 5-HT_3_ receptor subunit proteins A, B, C, D, and E through multiple sequence alignment and phylogenetic analysis in the metazoan lineage. We identified homolog sequences of 5-HT_3_ receptor subunits throughout the animal kingdom in both vertebrates and invertebrates. Most homolog sequences were identified in phylum Chordata (subphylum Craniate) followed by Arthropoda and Nematoda. Only one species from the Chordata subphylum Cephalochordate *Branchiostoma floridae* (XP_002588410.1) was identified during the first search (BLASTp search hit), however it was excluded due to its position in the phylogenetic tree and no species from subphylum Urochordata (tunicate) were present in the first search hits.

Ancestral species were placed at the start of each subunit tree and similar evolutionary patterns were observed in phylogenetic trees for the subunits. The evolutionary pattern of the phylogenetic trees for 5HT3A, 5HT3B, 5HT3C and 5HT3E subunits within the phylum Chordata goes from Fishes–Reptiles—Aves (nonflying birds to flying birds)—Rodents -Primates followed by carnivorous and large-bodied animals (megafauna) such as elephants, orcas, and cattle species (bison, cow). Thus, many metazoan species living in lentic and terrestrial ecosystems are equipped with similar 5-HT_3_ receptors to those in humans. This is unsurprising as 5-HT is widespread in the animal kingdom, and it would be expected that receptors to detect 5-HT will be common. Notably, G protein coupled 5-HT receptors are prevalent [37, 151] while our study underscores the presence of 5-HT_3_ receptor subunits in vertebrates and importantly highlights the existence of 5-HT_3_ receptor subunits in invertebrates.

Our study reveals that several species from Nematoda, Platyhelminthes, Arthropoda, Mollusca, Annelida, Echinodermata, Rotifera and Orthonectida are predicted to have 5-HT_3_ receptor subunits identified through their signature Cys-loop and TM domains. It is thus likely that these sequences assemble into 5-HT_3_ receptors containing five subunits. Although we uncovered variation in the extracellular sequences across the phyla, the loops showed considerable conservation in the critical amino acids involved in ligand binding (Figs 5 and S7 and S4 Table). The above observations suggest that they are also likely to have the capacity to bind specific 5-HT_3_ receptor ligands as they contain signature ligand binding residues in A, B, C, D, E and F loops in the extracellular domain. Fascinatingly, proteins (subunits A and D) represented in databases as hypothetical proteins from *Ramazzottius varieornatus* (water bear or moss piglet) from phylum Tardigrada also fall into inclusion criteria with the residues present in TM domains and Cys-loop, while larvae of reef building coral, *Pocillopora damicornis* from phylum Cnidaria, are also predicted to contain all 5-HT_3_ receptor subunits except subunit B.

To gain some insight into the 5-HT_3_ receptor ligand binding sites across the phyla, we studied conservation of residues critical to binding 5-HT and 5-HT_3_ receptor ligands. The orthosteric 5-HT_3_ receptor ligand binding site occurs between two adjacent A subunits (A+ A-) in the receptor pentamer [71, 73, 74, 97]. Although there are variations in this ligand binding site, it is evident that the orthosteric 5-HT_3_ receptor ligand binding site is present across the phyla. The most marked variations do occur in the extracellular domain, and these can modify hydrogen bonds, and van der Waals interactions altering recognition of ligands (serotonin and setrons). Even so, conservation of critical subunits indicates that both vertebrates and invertebrates contain the orthosteric 5-HT_3_ receptor ligand binding site.

Channel pores of assembled 5-HT_3_ receptor pentamers are lined by TM2 regions of the five subunits and these TM2 domains influence channel gating and ion movement [124]. The residues involved in channel lining and participating in ion movement are highly conserved in Chordata, however the residues in other phyla were often substituted. For example, Annelida, Arthropoda and Nematoda have conservative substitutions while nonconservative substitutions were observed in Platyhelminthes, Mollusca and Nematoda.

Amongst all these different phyla, it is notable that several gastrointestinal parasites, flat worms (Platyhelminthes e.g., *Schistosoma bovis*) and round worms (Nematoda, e.g., *Enterobius vermicularis*, *Ancylostoma caninum*, *Necator americanus*), contain 5-HT_3_ receptors. The mammalian gastrointestinal tract is a rich source of 5-HT and other nutrients, and 5-HT helps in development of parasitic flatworms and nematodes [152–154]. Therefore, we performed a small multiple sequence alignment containing host and parasitic species. Interestingly, this alignment (S8 Fig) highlights that more residues are conserved in the ligand binding region compared to the large alignment (S6 Fig). The 5HT3A subunit of *Schistosoma bovis* (RTG88761.1) contains conservative substitutions in extracellular loops (S8 Fig). However, the large multiple sequence alignment indicates that most Platyhelminth species lack equivalent or substitutional residues at E130, F131, Y235, E237, and Y95 residues (S6 Fig). Another Platyhelminth species, the cryptic tape worm *Sparganum proliferum* (VZH96180.1) that can fatally but rarely infect humans, also contains critical amino acid residues essential for 5-HT_3_ receptor ligand binding [155]. The round worm *Toxocara canis* (VDM43573.1), a parasite from phylum Nematoda also contains most of the critical amino acid residues found in the human 5HT3A subunit (S8 Fig). These observations raise the possibility that 5-HT_3_ receptors in parasitic nematodes and platyhelminths can bind to the 5-HT_3_ receptor ligands due to the number of similarities in the ligand binding site. We speculate that these parasites rely on 5-HT_3_ receptors for their growth and development and will presumably be affected by exposure to 5-HT_3_ receptor ligands used to treat humans and animals.

Due to adverse side effects, 5-HT_3_ receptor agonists are rarely used clinically [156, 157], while 5-HT_3_ receptor antagonists are the main drugs used in human and veterinary medicine, particularly to reduce nausea and vomiting [47, 48]. Clinical administration of 5-HT_3_ receptor antagonists is incredibly valuable to human and animal patients, but these drugs are not all metabolised before removal from the body. Interestingly, human urine contains tropisetron, ondansetron, granisetron and various metabolites following oral administration [158, 159]. Therefore leakage of 5-HT_3_ receptor antagonists and metabolites into the environment through human and animal waste is likely even though they are not as common a pharmaceutical pollutant as G protein-coupled receptor ligands [160]. Once in aquatic environments, these ligands may interact with various species and influence development or behaviours.

### Limitations

A major limitation of this study is that the information available in NCBI data base for many species is incomplete and at times poorly annotated. Although this has improved dramatically in the last decade and will continue to do so, it potentially means that we have not identified the full range of phyla where 5-HT_3_ receptors are expressed. A further limitation is lack of whole genome data for many species that means it is difficult to determine chromosome locations of the different 5-HT_3_ receptor subunits. Another limitation is that we extrapolated data from human and rodent studies to other organisms where residue alignments were not always apparent. To gain a better insight into the relevance of the predicted 5-HT_3_ receptor protein sequences, phyla specific pharmacological and behavioural studies need to be carried out using specific 5-HT_3_ receptor drugs such as setron antagonists (tropisetron, granisetron or ondansetron).

## Conclusions

Phylogenetic and sequence variation analyses have been used to predict and identify distribution of 5-HT_3_, 5-HT_3_ like and potential 5-HT_3_ (uncharacterised, hypothetical, or unnamed) receptors from different phyla in Metazoa. Analysis of amino acid sequences further reveals that functionally important residues in the ligand-binding and other domains of these receptor proteins are conserved throughout the metazoan lineage. These findings may be a useful predictor of unexpected therapeutic responses to serotonin and several setron antagonists in gut parasites that infect humans and animals in Metazoa.

## Supporting information

S1 Table Details of the 5HT3A, 5HT3B, 5HT3C, 5HT3D and 5HT3E subunit protein sequences downloaded from the NCBI database. (XLS)

S2 Table Number of ortholog sequences of the human 5-HT3 receptor subunits A through to E downloaded. (PDF)

S3 Table Species containing each of the 5-HT_3_ receptor subunits. (PDF)

S4 Table Critical amino acids and their substitutions in the ligand binding region of 5HT3A subunit in the metazoan lineages. (PDF)

S1 Fig Phylogenetic tree of 5HT3 A, B, C, D and E subunit proteins from phylum Chordata. The subunits are coloured according to the legend (A (blue), B (crimson), C (teal), D (purple) and E (grey)) and the human sequence is on an amber background. This analysis involved 85 amino acid sequences. There was a total of 234 alignment positions in the final dataset. Evolutionary analyses were conducted in MEGA X and tree editing was performed in iTOL. Tree scale represents the number of differences between sequences. The species details are listed in S3 Table.
(PDF)

S2 Fig Phylogenetic tree of 5-HT_3_ receptor B subunit homologs. The subunits are coloured according to the legend (Chordata (green), Nematoda (dark red), Arthropoda (orange), Platyhelminthes (dark purple), Mollusca (cyan), Hemichordata (purple), Annelida (blue) and Cnidaria (grey)), where the human sequence is highlighted with amber background. There was a total of 324 alignment positions in the final dataset. Evolutionary analyses were conducted in MEGA X and tree editing was performed in iTOL. Tree scale represents the number of differences between sequences.
(PDF)

S3 Fig Phylogenetic tree of 5-HT_3_ receptor C subunit homologs. The subunits are coloured according to the legend (Chordata (green), Nematoda (dark red), Arthropoda (orange), Platyhelminthes (dark purple), Mollusca (cyan), Annelida (blue) and Cnidaria (grey)) with the human sequence highlighted with amber background. This analysis involved 203 amino acid sequences and there was a total of 277 alignment positions in the final dataset. Evolutionary analyses were conducted in MEGA X and tree editing was performed in iTOL. Tree scale which represents the number of differences between sequences.
(PDF)

S4 Fig Phylogenetic tree of 5-HT_3_ receptor D subunit homologs. The subunits are coloured according to the legend (Chordata (green), Platyhelminthes (dark purple), Mollusca (cyan), Cnidaria (grey) and Tardigrada (pink)) with the human sequence highlighted with amber background. This analysis involved 36 amino acid sequences. There was a total of 20 alignment positions in the final dataset. Evolutionary analyses were conducted in MEGA X tree editing was performed in iTOL. Tree scale which represents the number of differences between sequences.
(PDF)

S5 Fig Phylogenetic tree of 5-HT_3_ receptor E subunit homologs. The subunits are coloured according to the legend (Chordata (green with human sequence highlighted with amber background), Nematoda (dark red), Arthropoda (orange), Platyhelminthes (dark purple), Mollusca (cyan), Annelida (blue), Cnidaria (grey)) Orthonectida (pale green) and Echinodermata (teal)). This analysis involved 147 amino acid sequences and 3 sequences were removed due to their placement inaccuracy. There was a total of 164 alignment positions in the final dataset. Evolutionary analyses were conducted in MEGA X and tree editing was performed in iTOL. Tree scale which represents the number of differences between sequences.
(PDF)

S6 Fig Multiple sequence alignment of 449 sequences of 5HT3A subunit homologs. ClustalW alignment of sequences whose details can be found in S1 Table. Accession number represents the species. The colours of the accession numbers in the alignment correspond to the colours of the phyla: Chordata (green with the human sequence highlighted with yellow background), Nematoda (dark red), Arthropoda (orange), Platyhelminthes (dark purple), Mollusca (cyan), Rotifera (yellow), Tardigrada (maroon), Echinodermata (teal), Annelida (blue) and Cnidaria (grey). The Cys-loop and transmembrane (TM) domains are highlighted in yellow and A to E loops in the ligand binding region are highlighted in grey. The symbols asterisk (*), colon (:), and dot (.) indicate identical amino acid residues, conserved substitutions, and semi-conserved substitutions in all sequences used in the alignment respectively are present on pages 83 and 89.
(PDF)

S7 Fig Full length consensus sequence logo of multiple sequence alignment of 449 5HT3A subunit homologs. The height of each letter is proportional to the observed frequency of the corresponding amino acid. The overall height of each stack is proportional to the sequence conservation, measured in bits from 1 to 4 units, at that position. The frequency and the position of the amino acids are represented on the y and x axis, respectively. Amino acids are coloured according to their chemical properties; polar amino acids G, S, T, Y and C in green, neutral amino acids Q or N in purple, basic amino acids K, R and H in blue, acidic amino acids D and E in red, hydrophobic amino acids A, V, L, I, P, W, F, and M in black.
(PDF)

S8 Fig Multiple sequence alignment of 5HT_3_A subunit proteins from domestic animals and parasites. ClustalW alignment of sequences from phylum Chordata (human—*Homo sapiens* (AAP35868.1), cat—*Felis catus* (XP_023094886.1), dog—*Canis lupus familiaris* (NP_001041584.1), pig—*Sus scrofa* (XP_003357349.2)), parasitic round worm from Nematoda—*Toxocara canis* (VDM43573.1), and flat worms from Platyhelminthes: flatworm1—*Schistosoma bovis* (RTG88761.1) and flatworm2—*Sparganum proliferum* (VZH96180.1). Extracellular loops are highlighted in grey in order of appearance D, A, E, B, F and C; Cys-loop in blue; and transmembrane (TM) domains TM1, TM2, TM3 and TM4 labelled in orange. The asterisk (*), colon (:), and dot (.) indicate identical amino acid residues, conserved substitutions, and semi-conserved substitutions in all sequences used in the alignment respectively.
(PDF)
