# Supplementary material for: Phylogenetic analyses of 5-hydroxytryptamine 3 (5-HT3) receptors in Metazoa
Source: PLoS One. 2023 Mar 1;18(3):e0281507. doi: 10.1371/journal.pone.0281507 (PMC9977066; doi:10.1371/journal.pone.0281507)
Supplement: S2 Table — (PDF) [file pone.0281507.s002.pdf]

S2 Table. Number of ortholog sequences of the human 5-HT_3 receptor subunits A-E downloaded.

**Phyla**	**5HT3A**	**5HT3B**	**5HT3C**	**5HT3D**	**5HT3E**
**Chordata**	375	260	174	30	120
**Arthropoda**	51	8	7	-	5
**Nematoda**	37	19	11	-	6
**Platyhelminthes**	16	5	5	2	10
**Cnidaria**	3	1	1	1	1
**Mollusca**	8	3	3	2	2
**Echinodermata**	1	-	-	-	1
**Orthonectida**	1	-	-	-	1
**Hemichordata**	1	1	-	-	-
**Rotifera**	1	-	-	-	-
**Annelida**	2	2	2	-	1
**Tardigrada**	1	-	-	1	-
**Total sequences**	**494**	**299**	**203**	**36**	**147**
