# Supplementary material for: Phylogenetic analyses of 5-hydroxytryptamine 3 (5-HT3) receptors in Metazoa
Source: PLoS One. 2023 Mar 1;18(3):e0281507. doi: 10.1371/journal.pone.0281507 (PMC9977066; doi:10.1371/journal.pone.0281507)
Supplement: S3 Table — (PDF) [file pone.0281507.s003.pdf]

**S3 Table. Species containing each of the 5HT3 subunits.**

| Type of species | Species name | 5HT3A | 5HT3B | 5HT3C | 5HT3D | 5HT3E |
|---|---|---|---|---|---|---|
| **Fishes** | *Perca flavescens* | XP_028450365.1 | XP_028450629.1 | TDH08617.1 | TDH05242.1 | XP_028443533.1 |
| | *Etheostoma spectabile* | XP_032389084.1 | XP_032389083.1 | KAA8581361.1 | KAA8589506.1 | KAA8586554.1 |
| **Herbivores** | *Camelus ferus* | XP_006181802.1 | XP_014412279.2 | XP_006183319.1 | XP_014413475.2 | XP_006183318.2 |
| | *Ceratotherium simum simum* | XP_004427367.1 | XP_004427368.2 | XP_004424722.1 | XP_014638498.1 | XP_004424721.1 |
| | *Loxodonta africana* | XP_010596188.1 | XP_010596253.2 | XP_003412930.1 | XP_023407262.1 | XP_023407234.1 |
| **Primates** | *Homo sapiens* | AAP35868.1 | EAW67236.1 | AAL66182.1 | NP_001138615.1 | NP_938056.1 |
| | *Trachypithecus francoisi* | XP_033060210.1 | XP_033060423.1 | XP_033058016.1 | XP_033056869.1 | XP_033056879.1 |
| | *Theropithecus gelada* | XP_025212649.1 | XP_025213442.1 | XP_025233149.1 | XP_025233016.1 | XP_025232862.1 |
| | *Hylobates moloch* | XP_032024018.1 | XP_032024016.1 | XP_032027642.1 | XP_032027644.1 | XP_032027640.1 |
| | *Papio anubis* | XP_003910778.3 | XP_031508785.1 | XP_003894807.3 | XP_017810529.1 | XP_031519389.1 |
| | *Pan troglodytes* | XP_001149570.1 | XP_522188.2 | XP_001136647.3 | PNI54703.1 | PNI54711.1 |
| | *Piliocolobus tephrosceles* | XP_023063823.1 | XP_023063821.1 | XP_023043458.1 | XP_023043231.1 | XP_023043451.1 |
| | *Pongo abelii* | PNJ75904.1 | PNJ75907.1 | XP_002814391.2 | PNJ69107.1 | XP_009237875.1 |
| | *Rhinopithecus roxellana* | XP_010370669.1 | XP_010370671.1 | XP_010364540.1 | XP_030795227.1 | XP_010364501.2 |
| **Carnivores** | *Felis catus* | XP_023094886.1 | XP_023094887.1 | XP_003991915.1 | XP_023116117.1 | XP_003991914.1 |
| | *Panthera pardus* | XP_019324655.1 | XP_015390088.1 | XP_007077611.1 | XP_015390983.1 | XP_007083696.1 |
| | *Panthera tigris altaica* | XP_007075625.1 | XP_019324656.1 | XP_019318868.1 | XP_019319069.1 | XP_019318873.1 |
| | *Ailuropoda melanoleuca* | XP_034523598.1 | XP_019664705.1 | XP_002915741.1 | XP_034528184.1 | EFB29636.1 |
| | *Suricata suricatta* | XP_029812166.1 | XP_029771553.1 | XP_029796625.1 | XP_029796626.1 | XP_029794597.1 |
| | *Vulpes vulpes* | XP_025862501.1 | XP_025862672.1 | XP_025863040.1 | XP_025863039.1 | XP_025863036.1 |
