# Supplementary material for: Phylogenetic analyses of 5-hydroxytryptamine 3 (5-HT3) receptors in Metazoa
Source: PLoS One. 2023 Mar 1;18(3):e0281507. doi: 10.1371/journal.pone.0281507 (PMC9977066; doi:10.1371/journal.pone.0281507)
Supplement: S4 Table — (PDF) [file pone.0281507.s004.pdf]

S4 Table. Critical amino acids and their substitution in ligand binding region of 5HT3A subunit in the metazoan lineages.

Phylum ↓	Loop A				Loop B						loop C					Loop D			Loop E				Loop F	
**Amino acid residue →**	**D119**	**E130**	**F131**	**D133**	**L179**	**T180**	**F181**	**S183**	**W184**	**I191**	**Y235**	**E237**	**F227**	**S228**	**M229**	**W91**	**R93**	**Y95**	**Y144**	**G149**	**V151**	**Y154**	**W196**	**D205**
**Chordata**	E, D, S, Q	√	√	A, T, N	R	P, A	W, G	√	S, I, P, V, F, Y	V, I, S	F	√	√	T, R, N, V	L, V	V	√	H, Q, S, E, F	R	√	S, R	L, P	L, M, I	√
**Rotifera**	R	X	X	√	√	K	I	√	√	F	X	X	V	Y	Y	√	D	X	M	√	√	P	X	√
**Platyhelminthes**	Q, S	X	X	√	I	D	√	√	L	√	X	X	√		Y	L	N, M	X	Q	√	√	R	√	M
**Nematoda**	E, G, R	Q	N	M, E	F, I	I, Q	Y	√	√	L	√	√	√	R	Y	Y	T, Q, K, D	X	K	V	M	V, Q, E	√	F
**Cnidaria**	√	X	X	√	√	K	√	√	√	√	X	X	√	Y	Y	C	√	X	√	√	√	√	√	√
**Mollusca**	√	X	X	√	√		√	A, P	T, M	√	X	X	√		Y	F	√	X	√	√	√	√	√	I
**Arthropoda**	H	X	X	√	M	K	W	√	√	Y, F, A, N, D	X	X	√	F, Y, Q	G	√	E, V, K	X	I, M	√	K, E	K, T	L, M, I	√
**Tardigrada**	√	X	X	√	√	K	√	√	√	F	X	X	K	Y	Y	√	√	X	M	√	√	P	X	√
**Annelida**	N	X	X	√	√	S	L	√	√	Q	X	X	K	Y	Y	√	T	X	T, V	√	√	K	K	√
**Echinodermata**	K	X	X	√	F	F	√	P	Q	F	X	X	Y	N	Y	√	√	X	L	√	√	G	K	E

Same amino acid residue (√); lack equivalent or substitutional residues (X). Amino acid numbering based on Figure 4 human 5HT3A subunit.
