# Supplementary material for: Phylogenetic analyses of 5-hydroxytryptamine 3 (5-HT3) receptors in Metazoa
Source: PLoS One. 2023 Mar 1;18(3):e0281507. doi: 10.1371/journal.pone.0281507 (PMC9977066; doi:10.1371/journal.pone.0281507)
Supplement: S4 Fig — The subunits are coloured according to the legend (Chordata (green), Platyhelminthes (dark purple), Mollusca (cyan), Cnidaria (grey) and Tardigrada (pink)) with the human sequence highlighted with amber background. This analysis involved 36 amino acid sequences. There was a total of 20 alignment positions in the final dataset. Evolutionary analyses were conducted in MEGA X tree editing was performed in iTOL. Tree scale which represents the number of differences between sequences. (PDF) [file pone.0281507.s008.pdf]

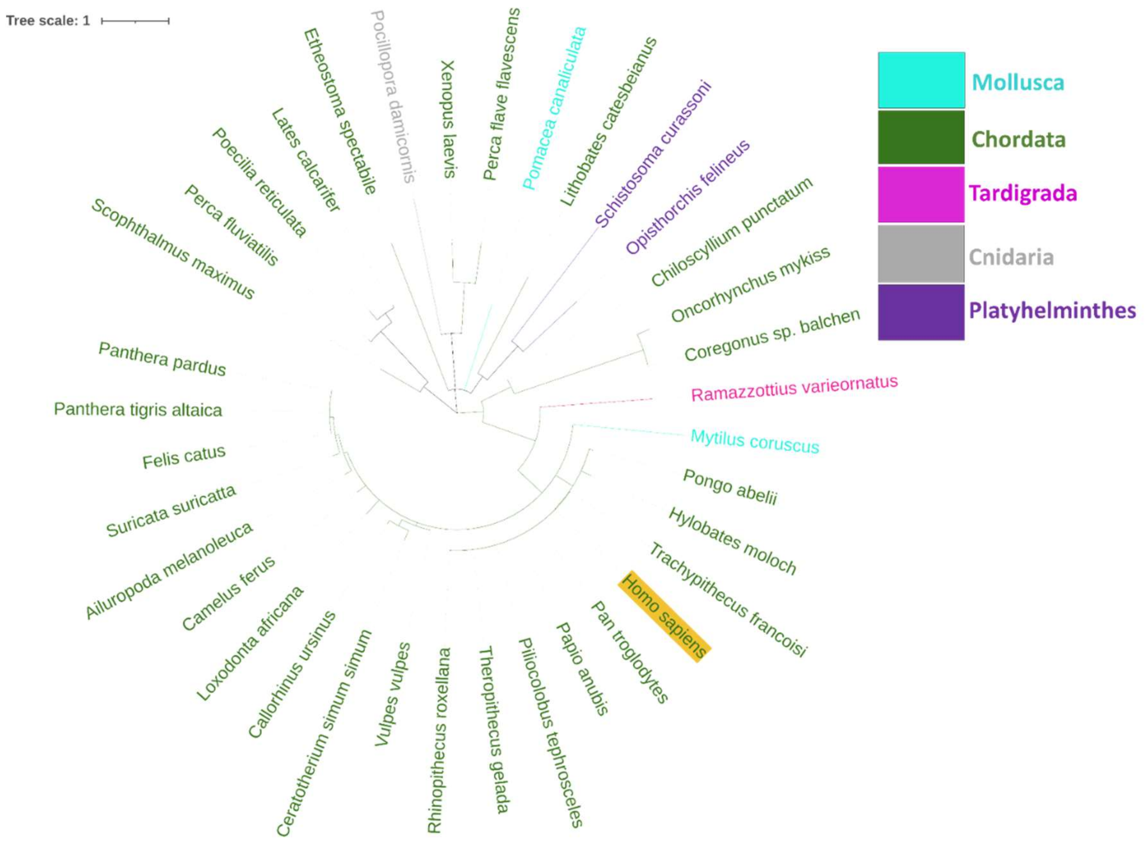

S4 Fig. Phylogenetic tree of 5-HT$_3$ receptor D subunit homologs. The subunits are coloured according to the legend (Chordata (green), Platyhelminthes (dark purple), Mollusca (cyan), Cnidaria (grey) and Tardigrada (pink)) with the human sequence highlighted with amber background. This analysis involved 36 amino acid sequences. There was a total of 20 alignment positions in the final dataset. Evolutionary analyses were conducted in MEGA X tree editing was performed in iTOL. Tree scale which represents the number of differences between sequences.
