# Supplementary material for: Phylogenetic analyses of 5-hydroxytryptamine 3 (5-HT3) receptors in Metazoa
Source: PLoS One. 2023 Mar 1;18(3):e0281507. doi: 10.1371/journal.pone.0281507 (PMC9977066; doi:10.1371/journal.pone.0281507)
Supplement: S6 Fig — ClustalW alignment of sequences whose details can be found in S1 Table. Accession number represents the species. The colours of the accession numbers in the alignment correspond to the colours of the phyla: Chordata (green with the human sequence highlighted with yellow background), Nematoda (dark red), Arthropoda (orange), Platyhelminthes (dark purple), Mollusca (cyan), Rotifera (yellow), Tardigrada (maroon), Echinodermata (teal), Annelida (blue) and Cnidaria (grey). The Cys-loop and transmembrane (TM) domains are highlighted in yellow and A to E loops in the ligand binding region are highlighted in grey. The symbols asterisk (*), colon (:), and dot (.) indicate identical amino acid residues, conserved substitutions, and semi-conserved substitutions in all sequences used in the alignment respectively are present on pages 83 and 89. (PDF) [file pone.0281507.s010.pdf]

CLUSTAL O(1.2.4) multiple sequence alignment

```
NP_509270.1         ------------------------------------------------------------	0
VDO93178.1          ------------------------------------------------------------	0
PAV91580.1          ------------------------------------------------------------	0
VDK46997.1          ------------------------------------------------------------	0
VIO86814.1          ------------------------------------------------------------	0
VDP38785.1          ------------------------------------------------------------	0
KOF68401.1          ------------------------------------------------------------	0
CDJ96026.1          ------------------------------------------------------------	0
EFX76216.1          ------------------------------------------------------------	0
KAF7403848.1        ------------------------------------------------------------	0
KAF7427032.1        ------------------------------------------------------------	0
KAE9417558.1        ------------------------------------------------------------	0
KJH51504.1          ------------------------------------------------------------	0
VDL69795.1          ------------------------------------------------------------	0
VDO32671.1          ------------------------------------------------------------	0
VBB32409.1          ------------------------------------------------------------	0
VDN54565.1          ------------------------------------------------------------	0
EGT55171.1          ------------------------------------------------------------	0
RCN52111.1          ------------------------------------------------------------	0
RMX54856.1          ------------------------------------------------------------	0
XP_022781674.1      MAVGRCSSVALAILFFTAVHFAI-----------------------TR---HDGT-----	29
RNA37099.1          ------------------------------------------------------------	0
KAA0187152.1        --------------------MLI-----------------------TY---IG-------	7
GAU96593.1          ------------------------------------------------------------	0
XP_009043980.1      ------------------------------------------------------------	0
VDM43573.1          ------------------------------------------------------------	0
XP_003140283.1      ------------------------------------------------------------	0
VDN82010.1          ------------------------------------------------------------	0
VDO31501.1          ------------------------------------------------------------	0
VDP19591.1          ------------------------------------------------------------	0
EDO32053.1          ------------------------------------------------------------	0
TRY67230.1          ------------------------------------------------------------	0
KAF7391312.1        ------------------------------------------------------------	0
GFG30449.1          ------------------------------------------------------------	0
KQS30083.1          ------------------------------------------------------------	0
EDW57583.2          --------------------MLS-----------------------AA---RA-------	7
KNC22799.1          ------------------------------------------------------------	0
RZF44856.1          ------------------------------------------------------------	0
ENN76856.1          ------------------------------------------------------------	0
KAF5300392.1        ------------------------------------------------------------	0
CAB3239999.1        ------------------------------------------------------------	0
PCG77624.1          ------------------------------------------------------------	0
PZC79131.1          ------------------------------------------------------------	0
KAF4083067.1        ------------------------------------------------------------	0
XP_009924659.1      ------------------------------------------------------------	0
XP_010123544.1      ------------------------------------------------------------	0
CBN81618.1          ------------------------------------------------------------	0
KAF3704230.1        ------------------------------------------------------------	0
RUS86578.1          ------------------------------------------------------------	0
XP_034309618.1      ------------------------------------------------------------	0
PIK58946.1          ------------------------------------------------------------	0
VUZ42516.1          ------------------------------------------------------------	0
PAA51166.1          ------------------------------------------------------------	0
TGZ55997.1          ------------------------------------------------------------	0
VDP48851.1          ------------------------------------------------------------	0
PVD38331.1          ------------------------------------------------------------	0
VDK73355.1          ------------------------------------------------------------	0
VDD84963.1          ------------------------------------------------------------	0
VDK42196.1          ------------------------------------------------------------	0
KFD58288.1          ------------------------------------------------------------	0
OUC49089.1          ------------------------------------------------------------	0
EYC26492.1          ------------------------------------------------------------	0
VDK27218.1          ------------------------------------------------------------	0
VDM17286.1          ------------------------------------------------------------	0
VDL91846.1          ------------------------------------------------------------	0
KAE9548540.1        ------------------------------------------------------------	0
PDM74087.1          ------------------------------------------------------------	0
XP_024504322.1      ------------------------------------------------------------	0
PIO52336.1          ------------------------------------------------------------	0
TKR73865.1          ------------------------------------------------------------	0
RLU23395.1          ------------------------------------------------------------	0
XP_002427906.1      ------------------------------------------------------------	0
TMW47392.1          ------------------------------------------------------------	0
VDD83956.1          ------------------------------------------------------------	0
```

```
VEL33078.1        ------------------------------------------------------------    0
XP_009019088.1    ------------------------------------------------------------    0
KAE9536378.1      ------------------------------------------------------------    0
KDR23473.1        ------------------------------------------------------------    0
KAF7266914.1      ------------------------------------------------------------    0
KAF4519445.1      ------------------------------------------------------------    0
OXU25983.1        ------------------------------------------------------------    0
TGZ32403.1        ------------------------------------------------------------    0
ELT94491.1        ------------------------------------------------------------    0
VVC39575.1        ------------------------------------------------------------    0
RVE49089.1        ------------------------------------------------------------    0
KAB0800277.1      ------------------------------------------------------------    0
TDG52197.1        ------------------------------------------------------------    0
TMW48669.1        ------------------------------------------------------------    0
XP_025896085.1    ------------------------------------------------------------    0
KAF2977017.1      ------------------------------------------------------------    0
XP_009321837.1    ------------------------------------------------------------    0
XP_009979985.1    ------------------------------------------------------------    0
XP_028942374.1    ------------------------------------------------------------    0
PKK19633.1        ------------------------------------------------------------    0
XP_009894240.1    ------------------------------------------------------------    0
XP_032820219.1    ------------------------------------------------------------    0
XP_029429480.1    ------------------------------------------------------------    0
XP_030077572.1    ------------------------------------------------------------    0
XP_033774596.1    ------------------------------------------------------------    0
ETE72600.1        ------------------------------------------------------------    0
XP_018082638.1    -------MSYLDIKFYVEGDTILTDLYRKPITRNTLLHAASCHPKSVLRGVPVGQ--FLR    51
XP_032905410.1    ------------------------------------------------------------    0
XP_020773490.1    ------------------------------------------------------------    0
XP_033833634.1    ------------------------------------------------------------    0
XP_030215795.1    ------------------------------------------------------------    0
XP_030602980.1    ------------------------------------------------------------    0
XP_004573543.1    ------------------------------------------------------------    0
XP_005916159.1    ------------------------------------------------------------    0
XP_013132089.1    ------------------------------------------------------------    0
XP_031603488.1    ------------------------------------------------------------    0
XP_005725536.1    ------------------------------------------------------------    0
XP_026038075.1    ------------------------------------------------------------    0
CAF96649.1        ------------------------------------------------------------    0
XP_023818570.1    ------------------------------------------------------------    0
RVE64289.1        ------------------------------------------------------------    0
XP_024121971.1    ------------------------------------------------------------    0
XP_015817559.1    ------------------------------------------------------------    0
XP_015225670.1    ------------------------------------------------------------    0
XP_012722063.1    ------------------------------------------------------------    0
XP_032431307.1    ------------------------------------------------------------    0
XP_014328329.1    ------------------------------------------------------------    0
XP_027886578.1    ------------------------------------------------------------    0
XP_008426791.1    ------------------------------------------------------------    0
XP_014838686.1    ------------------------------------------------------------    0
XP_014885967.1    ------------------------------------------------------------    0
XP_033954312.1    ------------------------------------------------------------    0
XP_034089244.1    ------------------------------------------------------------    0
XP_010780064.1    ------------------------------------------------------------    0
XP_033970261.1    ------------------------------------------------------------    0
XP_029375575.1    ------------------------------------------------------------    0
KAF0023022.1      ------------------------------------------------------------    0
XP_019952614.1    ------------------------------------------------------------    0
XP_034463117.1    ------------------------------------------------------------    0
XP_029924656.1    ------------------------------------------------------------    0
XP_028323228.1    ------------------------------------------------------------    0
TNM84480.1        ------------------------------------------------------------    0
XP_029703788.1    ------------------------------------------------------------    0
XP_030010368.1    ------------------------------------------------------------    0
XP_019725056.1    ------------------------------------------------------------    0
XP_034034934.1    ------------------------------------------------------------    0
XP_029956880.1    ------------------------------------------------------------    0
XP_008331307.3    ------------------------------------------------------------    0
XP_031724845.1    ------------------------------------------------------------    0
XP_013859395.1    ------------------------------------------------------------    0
XP_017275335.1    ------------------------------------------------------------    0
XP_020496197.1    ------------------------------------------------------------    0
XP_029030400.1    ------------------------------------------------------------    0
XP_022060538.1    ------------------------------------------------------------    0
XP_023150584.1    ------------------------------------------------------------    0
XP_029303892.1    ------------------------------------------------------------    0
XP_020454172.1    ------------------------------------------------------------    0
```

```
TKS83244.1         ------------------------------------------------------------      0
XP_022616583.1     ------------------------------------------------------------      0
XP_030293172.1     ------------------------------------------------------------      0
XP_027129539.1     ------------------------------------------------------------      0
XP_034539538.1     ------------------------------------------------------------      0
XP_008277330.1     ------------------------------------------------------------      0
XP_028276940.1     ------------------------------------------------------------      0
XP_023263433.1     ------------------------------------------------------------      0
XP_034406336.1     ------------------------------------------------------------      0
XP_026184460.1     ------------------------------------------------------------      0
KAF1378228.1       ------------------------------------------------------------      0
XP_028450365.1     ------------------------------------------------------------      0
XP_031163851.1     ------------------------------------------------------------      0
XP_032389084.1     ------------------------------------------------------------      0
XP_033494682.1     ------------------------------------------------------------      0
XP_018544782.1     ------------------------------------------------------------      0
XP_026228189.1     ------------------------------------------------------------      0
XP_028976606.1     ------------------------------------------------------------      0
CAB1352378.1       ------------------------------------------------------------      0
XP_023866849.1     ------------------------------------------------------------      0
XP_013992832.1     ------------------------------------------------------------      0
XP_029546688.1     ------------------------------------------------------------      0
XP_021481546.1     ------------------------------------------------------------      0
XP_020339889.1     ------------------------------------------------------------      0
XP_029481972.1     ------------------------------------------------------------      0
KPP68743.1         ------------------------------------------------------------      0
XP_023669383.1     ------------------------------------------------------------      0
KAA0720096.1       ------------------------------------------------------------      0
XP_009293684.1     ------------------------------------------------------------      0
XP_026090784.1     ------------------------------------------------------------      0
XP_018918715.1     ------------------------------------------------------------      0
KAF4098549.1       ------------------------------------------------------------      0
XP_016084173.1     ------------------------------------------------------------      0
XP_016332759.1     ------------------------------------------------------------      0
XP_016398039.1     ------------------------------------------------------------      0
XP_026989970.1     ------------------------------------------------------------      0
TSQ12698.1         ------------------------------------------------------------      0
XP_017347546.1     ------------------------------------------------------------      0
XP_026794616.2     ------------------------------------------------------------      0
XP_026861889.1     ------------------------------------------------------------      0
XP_017575347.1     ------------------------------------------------------------      0
XP_022531596.1     ------------------------------------------------------------      0
XP_012691019.2     ------------------------------------------------------------      0
XP_028839723.1     ------------------------------------------------------------      0
XP_030638860.1     ------------------------------------------------------------      0
XP_007882964.1     ------------------------------------------------------------      0
GCB70244.1         ------------------------------------------------------------      0
GCC26242.1         ------------------------------------------------------------      0
XP_020387034.1     ------------------------------------------------------------      0
XP_028931940.1     ------------------------------------------------------------      0
XP_031762490.1     ------------------------------------------------------------      0
PIO32240.1         ------------------------------------------------------------      0
XP_018425466.1     ------------------------------------------------------------      0
XP_006642351.1     ------------------------------------------------------------      0
XP_028665372.1     ------------------------------------------------------------      0
GCF49814.1         ------------------------------------------------------------      0
XP_015277816.1     ------------------------------------------------------------      0
XP_005987340.2     ------------------------------------------------------------      0
XP_019339534.1     ------------------------------------------------------------      0
XP_025063414.1     ------------------------------------------------------------      0
XP_019367488.1     ------------------------------------------------------------      0
XP_019412078.1     ------------------------------------------------------------      0
XP_014434314.1     ------------------------------------------------------------      0
XP_007060556.1     ------------------------------------------------------------      0
XP_024072175.1     ------------------------------------------------------------      0
XP_008170567.1     ------------------------------------------------------------      0
XP_034610543.1     ------------------------------------------------------------      0
XP_030394127.1     ------------------------------------------------------------      0
XP_032651960.1     ------------------------------------------------------------      0
XP_025020083.1     ------------------------------------------------------------      0
XP_026536833.1     ------------------------------------------------------------      0
XP_026564787.1     ------------------------------------------------------------      0
XP_032084675.1     ------------------------------------------------------------      0
XP_029139341.1     ------------------------------------------------------------      0
XP_034281006.1     ------------------------------------------------------------      0
XP_020649062.1     ------------------------------------------------------------      0
XP_008117087.1     ------------------------------------------------------------      0
```

```
XP_028564324.1      ------------------------------------------------------------     0
XP_033028155.1      ------------------------------------------------------------     0
XP_025963249.1      ------------------------------------------------------------     0
XP_009668348.1      ------------------------------------------------------------     0
XP_013798935.1      ------------------------------------------------------------     0
XP_025913685.1      ------------------------------------------------------------     0
XP_013042552.1      ------------------------------------------------------------     0
XP_005030458.2      ------------------------------------------------------------     0
XP_032057953.1      ------------------------------------------------------------     0
XP_021232050.1      ------------------------------------------------------------     0
OXB62403.1          ------------------------------------------------------------     0
OXB81319.1          ------------------------------------------------------------     0
XP_010722007.1      ------------------------------------------------------------     0
XP_015739349.1      ------------------------------------------------------------     0
XP_031455498.1      ------------------------------------------------------------     0
POI27435.1          ------------------------------------------------------------     0
XP_004948120.1      ------------------------------------------------------------     0
XP_032851190.1      ------------------------------------------------------------     0
XP_010007255.1      ------------------------------------------------------------     0
XP_030320702.1      ------------------------------------------------------------     0
XP_010191940.1      ------------------------------------------------------------     0
XP_027737112.1      ------------------------------------------------------------     0
XP_027555032.1      ------------------------------------------------------------     0
XP_032565370.1      ----------------------------------------------------------MQ     2
XP_027511217.1      ---------------------------------------------------------MSP     3
XP_027593499.1      ----------------------------------------------------------MQ     2
XP_017664924.1      ------------------------------------------------------------     0
XP_029817938.1      ------------------------------------------------------------     0
XP_005058641.1      ------------------------------------------------------------     0
XP_021385804.1      ------------------------------------------------------------     0
XP_030146687.2      ------------------------------------------------------------     0
KAF4796420.1        ------------------------------------------------------------     0
XP_032937581.1      ------------------------------------------------------------     0
XP_031989659.1      ------------------------------------------------------------     0
XP_010402086.1      ------------------------------------------------------------     0
XP_017594069.1      ------------------------------------------------------------     0
XP_014115268.1      ---------------------------MGPPAAAPSSHSAHARPLRFRPRRPPGP-----     28
XP_023797108.1      ------------------------------------------------------------     0
XP_033375614.1      ------------------------------------------------------------     0
XP_014740121.1      ---------------------------MGAAGSGAV-PAHRARAAAPVPAAPPAGAVSAV     32
RLV83430.1          ------------------------------------------------------------     0
XP_009096098.2      ---------------------------MGPPAAAPS-RTAHARPLRFRPRRPPGP-----     27
TRZ15870.1          ------------------------------------------------------------     0
RMB91935.1          ------------------------------------------------------------     0
XP_030820843.1      ------------------------------------------------------------     0
XP_014165179.1      ------------------------------------------------------------     0
XP_026653582.1      ------------------------------------------------------------     0
PKU35975.1          ------------------------------------------------------------     0
XP_014805072.1      ------------------------------------------------------------     0
XP_009818330.1      ------------------------------------------------------------     0
OPJ68307.1          ------------------------------------------------------------     0
XP_008936289.1      ------------------------------------------------------------     0
XP_010287046.1      ------------------------------------------------------------     0
XP_005240140.2      ------------------------------------------------------------     0
XP_005437752.2      ------------------------------------------------------------     0
KFV74811.1          ------------------------------------------------------------     0
XP_010018389.1      ------------------------------------------------------------     0
KQK78711.1          ------------------------------------------------------------     0
XP_009570162.1      ------------------------------------------------------------     0
KFP11268.1          ------------------------------------------------------------     0
KFQ98910.1          ------------------------------------------------------------     0
XP_012985202.3      ------------------------------------------------------------     0
XP_010573388.1      ------------------------------------------------------------     0
XP_029879496.1      ------------------------------------------------------------     0
XP_030361086.1      ------------------------------------------------------------     0
KFM00668.1          ------------------------------------------------------------     0
KAF1479074.1        ------------------------------------------------------------     0
KAF1651161.1        ------------------------------------------------------------     0
KAF1673648.1        ------------------------------------------------------------     0
KAF1493319.1        ------------------------------------------------------------     0
KAF1584157.1        ------------------------------------------------------------     0
KAF1571723.1        ------------------------------------------------------------     0
KAF1533169.1        ------------------------------------------------------------     0
KAF1638955.1        ------------------------------------------------------------     0
KAF1549972.1        ------------------------------------------------------------     0
KAF1606914.1        ------------------------------------------------------------     0
KAF1510957.1        ------------------------------------------------------------     0
```

```
KAF1498899.1        ------------------------------------------------------------      0
KAF1411525.1        ------------------------------------------------------------      0
KAF1429205.1        ------------------------------------------------------------      0
XP_005334318.1      ------------------------------------------------------------      0
XP_014395552.1      ------------------------------------------------------------      0
KAB0404854.1        ------------------------------------------------------------      0
XP_007494828.1      ------------------------------------------------------------      0
XP_003764254.1      ------------------------------------------------------------      0
XP_020845489.1      ------------------------------------------------------------      0
XP_027703160.1      ------------------------------------------------------------      0
XP_017523929.1      ------------------------------------------------------------      0
XP_008688428.1      ------------------------------------------------------------      0
XP_029812166.1      ------------------------------------------------------------      0
XP_025749781.1      ------------------------------------------------------------      0
XP_004416432.1      ------------------------------------------------------------      0
XP_027436262.1      ------------------------------------------------------------      0
XP_032284025.1      ------------------------------------------------------------      0
XP_006735421.1      ------------------------------------------------------------      0
XP_021552166.1      ------------------------------------------------------------      0
XP_026361066.1      ------------------------------------------------------------      0
XP_034523598.1      ------------------------------------------------------------      0
NP_001297113.1      ------------------------------------------------------------      0
XP_032215488.1      ------------------------------------------------------------      0
VCX31483.1          ------------------------------------------------------------      0
XP_022369003.1      ------------------------------------------------------------      0
XP_032694248.1      ------------------------------------------------------------      0
NP_001041584.1      ------------------------------------------------------------      0
XP_025862501.1      ------------------------------------------------------------      0
KAF0873564.1        ------------------------------------VSFPTLRPFRTS-AREPGDSLALF      23
XP_025784751.1      ------------------------------------------------------------      0
XP_007075625.1      ------------------------------------MPFLSLSPFKTS-AWTPWDSLVLL      23
XP_030189489.1      ------------------------------------------------------------      0
XP_019324655.1      ------------------------------------MPFLSLSPFKTS-AWTPGDSLVLL      23
XP_023094886.1      ------------------------------------MPFLSLSPFKTS-AWTPGDSLVLL      23
XP_026892357.1      ------------------------------------------------------------      0
XP_016004457.1      ------------------------------------------------------------      0
XP_006912860.1      ------------------------------------------------------------      0
XP_011363679.1      ------------------------------------------------------------      0
XP_016076060.1      ------------------------------------------------------------      0
XP_008148371.1      ------------------------------------------------------------      0
XP_006093568.1      ------------------------------------------------------------      0
XP_006761601.1      ------------------------------------------------------------      0
XP_024426743.1      ------------------------------------------------------------      0
XP_019520779.1      ------------------------------------------------------------      0
XP_032976539.1      ------------------------------------------------------------      0
ELW64270.1          ------------------------------------------------------------      0
XP_004427367.1      ------------------------------------------------------------      0
XP_008529353.1      ------------------------------------------------------------      0
NP_001288165.1      ------------------------------------------------------------      0
XP_014711213.1      ------------------------------------------------------------      0
XP_012514621.1      ------------------------------------------------------------      0
XP_008071525.1      ------------------------------------------------------------      0
XP_009005082.1      ------------------------------------------------------------      0
XP_010332832.1      ------------------------------------------------------------      0
XP_012326058.1      ------------------------------------------------------------      0
XP_017378796.1      ------------------------------------------------------------      0
XP_032141374.1      ------------------------------------------------------------      0
XP_032024018.1      ------------------------------------------------------------      0
XP_018891497.2      ------------------------------------------------------------      0
XP_030684894.1      ------------------------------------------------------------      0
PNJ75904.1          ------------------------------------------------------------      0
AAP35868.1          ------------------------------------------------------------      0
XP_001149570.1      ------------------------------------------------------------      0
XP_003805532.1      ------------------------------------------------------------      0
XP_023063823.1      ------------------------------------------------------------      0
XP_033060210.1      ------------------------------------------------------------      0
XP_010370669.1      ------------------------------------------------------------      0
XP_017738496.1      ------------------------------------------------------------      0
XP_011782168.1      ------------------------------------------------------------      0
EHH23440.1          ------------------------------------------------------------      0
XP_003910778.3      ------------------------------------------------------------      0
XP_025212649.1      ------------------------------------------------------------      0
XP_008019132.1      ------------------------------------------------------------      0
XP_011832421.1      ------------------------------------------------------------      0
XP_011759618.1      ------------------------------------------------------------      0
XP_005579749.1      ------------------------------------------------------------      0
XP_011921684.1      ------------------------------------------------------------      0
```

```
XP_008259391.1      ------------------------------------------------------------      0
XP_012782438.1      ------------------------------------------------------------      0
VTJ86076.1          ------------------------------------------------------------      0
XP_015345533.1      ------------------------------------------------------------      0
XP_027786432.1      ------------------------------------------------------------      0
XP_026248345.1      ------------------------------------------------------------      0
XP_005378209.1      ------------------------------------------------------------      0
XP_004466010.1      ------------------------------------------------------------      0
XP_020035707.1      ------------------------------------------------------------      0
XP_006181802.1      ------------------------------------------------------------      0
XP_006207862.1      ------------------------------------------------------------      0
XP_005891968.1      ------------------------------------------------------------      0
XP_010828662.1      ------------------------------------------------------------      0
XP_019830126.1      ------------------------------------------------------------      0
XP_017914486.1      ------------------------------------------------------------      0
XP_006060266.2      ------------------------------------------------------------      0
XP_020728447.1      ------------------------------------------------------------      0
KAF4008892.1        ------------------------------------------------------------      0
KAB0348059.1        ------------------------------------------------------------      0
KAB0371113.1        ------------------------------------------------------------      0
MBV96963.1          ------------------------------------------------------------      0
XP_007172048.1      ------------------------------------------------------------      0
XP_007452270.1      ------------------------------------------------------------      0
XP_023987108.1      ------------------------------------------------------------      0
XP_004273432.1      ------------------------------------------------------------      0
XP_004328646.1      ------------------------------------------------------------      0
XP_026937670.1      ------------------------------------------------------------      0
XP_030691031.1      ------------------------------------------------------------      0
XP_022414870.1      ------------------------------------------------------------      0
XP_029064651.1      ------------------------------------------------------------      0
XP_024607566.1      ------------------------------------------------------------      0
XP_032496907.1      ------------------------------------------------------------      0
XP_007535559.1      ------------------------------------------------------------      0
XP_031299536.1      ------------------------------------------------------------      0
XP_004666516.1      ------------------------------------------------------------      0
XP_008830963.1      ------------------------------------------------------------      0
XP_005347338.1      ------------------------------------------------------------      0
XP_005069548.1      ------------------------------------------------------------      0
XP_027267894.1      ------------------------------------------------------------      0
OBS80992.1          ------------------------------------------------------------      0
XP_006979812.1      ------------------------------------------------------------      0
XP_028720687.1      ------------------------------------------------------------      0
XP_021489005.1      ------------------------------------------------------------      0
XP_031199589.1      ------------------------------------------------------------      0
XP_028617944.1      ------------------------------------------------------------      0
XP_034347030.1      ------------------------------------------------------------      0
NP_077370.2         ------------------------------------------------------------      0
XP_032766961.1      ------------------------------------------------------------      0
XP_021063186.1      ------------------------------------------------------------      0
NP_001093114.1      ------------------------------------------------------------      0
XP_021028379.1      ------------------------------------------------------------      0
XP_004689305.1      ------------------------------------------------------------      0
XP_004712871.1      ------------------------------------------------------------      0
XP_004382575.1      ------------------------------------------------------------      0
XP_010596188.1      ------------------------------------------------------------      0
XP_006890855.1      ------------------------------------------------------------      0
XP_006834038.1      ------------------------------------------------------------      0
XP_007934716.1      ------------------------------------------------------------      0
XP_012603491.1      ------------------------------------------------------------      0
XP_012663606.1      ------------------------------------------------------------      0
XP_028372812.1      ------------------------------------------------------------      0
XP_008589608.1      ------------------------------------------------------------      0
XP_012865077.1      ------------------------------------------------------------      0
NP_001166178.1      ------------------------------------------------------------      0
XP_004636553.1      ------------------------------------------------------------      0
XP_004856670.1      ------------------------------------------------------------      0
XP_010627744.1      ------------------------------------------------------------      0

NP_509270.1         ------------------------------------------------------------      0
VDO93178.1          ------------------------------------------------------------      0
PAV91580.1          ------------------------------------------------------------      0
VDK46997.1          ------------------------------------------------------------      0
VIO86814.1          ------------------------------------------------------------      0
VDP38785.1          ------------------------------------------------------------      0
KOF68401.1          ------------------------------------------------------------      0
CDJ96026.1          -------------------------------------------------------MLLLL      5
```

```
EFX76216.1          ------------------------------------------------------------    0
KAF7403848.1        ------------------------------------------------------------    0
KAF7427032.1        ------------------------------------------------------------    0
KAE9417558.1        ------------------------------------------------------------    0
KJH51504.1          ------------------------------------------------------------    0
VDL69795.1          ------------------------------------------------------------    0
VDO32671.1          ------------------------------------------------------------    0
VBB32409.1          ------------------------------------------------------------    0
VDN54565.1          ------------------------------------------------------------    0
EGT55171.1          ------------------------------------------------------------    0
RCN52111.1          ------------------------------------------------------------    0
RMX54856.1          ------------------------------------------------------------    0
XP_022781674.1      -----QKFYSHLLYKINDFENAKEPGLNFWTPLSGLAGERCGSH------CCRLRLRLRL    78
RNA37099.1          ------------------------------------------------------------    0
KAA0187152.1        -------------------------GTV---VS-SVVSRVMGST----------NDLTTT    28
GAU96593.1          ------------------------------------------------------------    0
XP_009043980.1      ------------------------------------------------------------    0
VDM43573.1          ------------------------------------------------------------    0
XP_003140283.1      ------------------------------------------------------------    0
VDN82010.1          ------------------------------------------------------------    0
VDO31501.1          ------------------------------------------------------------    0
VDP19591.1          ------------------------------------------------------------    0
EDO32053.1          ------------------------------------------------------------    0
TRY67230.1          ------------------------------------------------------------    0
KAF7391312.1        ------------------------------------------------------------    0
GFG30449.1          ------------------------------------------------------------    0
KQS30083.1          ------------------------------------------------------------    0
EDW57583.2          -------------------------GRD---QATHTNIRLCARK------RQRLRRRRKR    33
KNC22799.1          ------------------------------------------------------------    0
RZF44856.1          ------------------------------------------------------------    0
ENN76856.1          ------------------------------------------------------------    0
KAF5300392.1        ------------------------------------------------------------    0
CAB3239999.1        ------------------------------------------------------------    0
PCG77624.1          ------------------------------------------------------------    0
PZC79131.1          ------------------------------------------------------------    0
KAF4083067.1        ------------------------------------------------------------    0
XP_009924659.1      ------------------------------------------------------------    0
XP_010123544.1      ------------------------------------------------------------    0
CBN81618.1          ------------------------------------------------------------    0
KAF3704230.1        ------------------------------------------------------------    0
RUS86578.1          ------------------------------------------------------------    0
XP_034309618.1      ------------------------------------------------------------    0
PIK58946.1          ------------------------------------------------------------    0
VUZ42516.1          ------------------------------------------------------------    0
PAA51166.1          ------------------------------------------------------------    0
TGZ55997.1          ------------------------------------------------------------    0
VDP48851.1          ------------------------------------------------------------    0
PVD38331.1          ------------------------------------------------------------    0
VDK73355.1          ------------------------------------------------------------    0
VDD84963.1          ------------------------------------------------------------    0
VDK42196.1          ------------------------------------------------------------    0
KFD58288.1          ------------------------------------------------------------    0
OUC49089.1          ------------------------------------------------------------    0
EYC26492.1          ------------------------------------------------------------    0
VDK27218.1          ------------------------------------------------------------    0
VDM17286.1          ------------------------------------------------------------    0
VDL91846.1          ------------------------------------------------------------    0
KAE9548540.1        ------------------------------------------------------------    0
PDM74087.1          ------------------------------------------------------------    0
XP_024504322.1      ------------------------------------------------------------    0
PIO52336.1          ------------------------------------------------------------    0
TKR73865.1          ------------------------------------------------------------    0
RLU23395.1          ------------------------------------------------------------    0
XP_002427906.1      ------------------------------------------------------------    0
TMW47392.1          ------------------------------------------------------------    0
VDD83956.1          ------------------------------------------------------------    0
VEL33078.1          ------------------------------------------------------------    0
XP_009019088.1      ------------------------------------------------------------    0
KAE9536378.1        ------------------------------------------------------------    0
KDR23473.1          ------------------------------------------------------------    0
KAF7266914.1        ------------------------------------------------------------    0
KAF4519445.1        ------------------------------------------------------------    0
OXU25983.1          ------------------------------------------------------------    0
TGZ32403.1          ------------------------------------------------------------    0
ELT94491.1          ------------------------------------------------------------    0
VVC39575.1          ------------------------------------------------------------    0
RVE49089.1          ------------------------------------------------------------    0
```

```
KAB0800277.1      ------------------------------------------------------------   0
TDG52197.1        ------------------------------------------------------------   0
TMW48669.1        ------------------------------------------------------------   0
XP_025896085.1    ------------------------------------------------------------   0
KAF2977017.1      ------------------------------------------------------------   0
XP_009321837.1    ------------------------------------------------------------   0
XP_009979985.1    ------------------------------------------------------------   0
XP_028942374.1    ------------------------------------------------------------   0
PKK19633.1        ----------------------------------------------MPVA----------   4
XP_009894240.1    ----------------MQRERLIAR------LIGRSQKPDGGGKSTLPCR----------   28
XP_032820219.1    ------------------------------------------------------------   0
XP_029429480.1    ------------------------------------------------------------   0
XP_030077572.1    ------------------------------------------------------------   0
XP_033774596.1    ------------------------------------------------------------   0
ETE72600.1        ------------------------------------------------------------   0
XP_018082638.1    LRRICTTWDSFNEQAMLLWDRFIQRGYDFWDVKSAYDRAVGTSRSDLLAS-QPKKAT--H   108
XP_032905410.1    ------------------------------------------------------------   0
XP_020773490.1    ------------------------------------------------------------   0
XP_033833634.1    ------------------------------------------------------------   0
XP_030215795.1    ------------------------------------------------------------   0
XP_030602980.1    ------------------------------------------------------------   0
XP_004573543.1    ------------------------------------------------------------   0
XP_005916159.1    ------------------------------------------------------------   0
XP_013132089.1    ------------------------------------------------------------   0
XP_031603488.1    ------------------------------------------------------------   0
XP_005725536.1    ------------------------------------------------------------   0
XP_026038075.1    ------------------------------------------------------------   0
CAF96649.1        ------------------------------------------------------------   0
XP_023818570.1    ------------------------------------------------------------   0
RVE64289.1        ------------------------------------------------------------   0
XP_024121971.1    ------------------------------------------------------------   0
XP_015817559.1    ------------------------------------------------------------   0
XP_015225670.1    ------------------------------------------------------------   0
XP_012722063.1    ------------------------------------------------------------   0
XP_032431307.1    ------------------------------------------------------------   0
XP_014328329.1    ------------------------------------------------------------   0
XP_027886578.1    ------------------------------------------------------------   0
XP_008426791.1    ------------------------------------------------------------   0
XP_014838686.1    ------------------------------------------------------------   0
XP_014885967.1    ------------------------------------------------------------   0
XP_033954312.1    ------------------------------------------------------------   0
XP_034089244.1    ------------------------------------------------------------   0
XP_010780064.1    ------------------------------------------------------------   0
XP_033970261.1    ------------------------------------------------------------   0
XP_029375575.1    ------------------------------------------------------------   0
KAF0023022.1      ------------------------------------------------------------   0
XP_019952614.1    ------------------------------------------------------------   0
XP_034463117.1    ------------------------------------------------------------   0
XP_029924656.1    ------------------------------------------------------------   0
XP_028323228.1    ------------------------------------------------------------   0
TNM84480.1        ------------------------------------------------------------   0
XP_029703788.1    ------------------------------------------------------------   0
XP_030010368.1    ------------------------------------------------------------   0
XP_019725056.1    ------------------------------------------------------------   0
XP_034034934.1    ------------------------------------------------------------   0
XP_029956880.1    ------------------------------------------------------------   0
XP_008331307.3    ------------------------------------------------------------   0
XP_031724845.1    ------------------------------------------------------------   0
XP_013859395.1    ------------------------------------------------------------   0
XP_017275335.1    ------------------------------------------------------------   0
XP_020496197.1    ------------------------------------------------------------   0
XP_029030400.1    ------------------------------------------------------------   0
XP_022060538.1    ------------------------------------------------------------   0
XP_023150584.1    ------------------------------------------------------------   0
XP_029303892.1    ------------------------------------------------------------   0
XP_020454172.1    ------------------------------------------------------------   0
TKS83244.1        ------------------------------------------------------------   0
XP_022616583.1    ------------------------------------------------------------   0
XP_030293172.1    ------------------------------------------------------------   0
XP_027129539.1    ------------------------------------------------------------   0
XP_034539538.1    ------------------------------------------------------------   0
XP_008277330.1    ------------------------------------------------------------   0
XP_028276940.1    ------------------------------------------------------------   0
XP_023263433.1    ------------------------------------------------------------   0
XP_034406336.1    ------------------------------------------------------------   0
XP_026184460.1    ------------------------------------------------------------   0
KAF1378228.1      ------------------------------------------------------------   0
```

```
XP_028450365.1      ------------------------------------------------------------      0
XP_031163851.1      ------------------------------------------------------------      0
XP_032389084.1      ------------------------------------------------------------      0
XP_033494682.1      ------------------------------------------------------------      0
XP_018544782.1      ------------------------------------------------------------      0
XP_026228189.1      ------------------------------------------------------------      0
XP_028976606.1      ------------------------------------------------------------      0
CAB1352378.1        ------------------------------------------------------------      0
XP_023866849.1      ------------------------------------------------------------      0
XP_013992832.1      ------------------------------------------------------------      0
XP_029546688.1      ------------------------------------------------------------      0
XP_021481546.1      ------------------------------------------------------------      0
XP_020339889.1      ------------------------------------------------------------      0
XP_029481972.1      ------------------------------------------------------------      0
KPP68743.1          ------------------------------------------------------------      0
XP_023669383.1      ------------------------------------------------------------      0
KAA0720096.1        ------------------------------------------------------------      0
XP_009293684.1      ------------------------------------------------------------      0
XP_026090784.1      ------------------------------------------------------------      0
XP_018918715.1      ------------------------------------------------------------      0
KAF4098549.1        ------------------------------------------------------------      0
XP_016084173.1      ------------------------------------------------------------      0
XP_016332759.1      ------------------------------------------------------------      0
XP_016398039.1      ------------------------------------------------------------      0
XP_026989970.1      ------------------------------------------------------------      0
TSQ12698.1          ------------------------------------------------------------      0
XP_017347546.1      ------------------------------------------------------------      0
XP_026794616.2      ------------------------------------------------------------      0
XP_026861889.1      ------------------------------------------------------------      0
XP_017575347.1      ------------------------------------------------------------      0
XP_022531596.1      ------------------------------------------------------------      0
XP_012691019.2      ------------------------------------------------------------      0
XP_028839723.1      ------------------------------------------------------------      0
XP_030638860.1      ------------------------------------------------------------      0
XP_007882964.1      ------------------------------------------------------------      0
GCB70244.1          ------------------------------------------------------------      0
GCC26242.1          ------------------------------------------------------------      0
XP_020387034.1      ------------------------------------------------------------      0
XP_028931940.1      ------------------------------------------------------------      0
XP_031762490.1      ------------------------------------------------------------      0
PIO32240.1          ------------------------------------------------------------      0
XP_018425466.1      ------------------------------------------------------------      0
XP_006642351.1      ------------------------------------------------------------      0
XP_028665372.1      ------------------------------------------------------------      0
GCF49814.1          ------------------------------------------------------------      0
XP_015277816.1      ------------------------------------------------------------      0
XP_005987340.2      ------------------------------------------------------------      0
XP_019339534.1      ------------------------------------------------------------      0
XP_025063414.1      ------------------------------------------------------------      0
XP_019367488.1      ------------------------------------------------------------      0
XP_019412078.1      ------------------------------------------------------------      0
XP_014434314.1      ------------------------------------------------------------      0
XP_007060556.1      ------------------------------------------------------------      0
XP_024072175.1      ------------------------------------------------------------      0
XP_008170567.1      ------------------------------------------------------------      0
XP_034610543.1      ------------------------------------------------------------      0
XP_030394127.1      ------------------------------------------------------------      0
XP_032651960.1      ------------------------------------------------------------      0
XP_025020083.1      ------------------------------------------------------------      0
XP_026536833.1      ------------------------------------------------------------      0
XP_026564787.1      ------------------------------------------------------------      0
XP_032084675.1      ------------------------------------------------------------      0
XP_029139341.1      ------------------------------------------------------------      0
XP_034281006.1      ------------------------------------------------------------      0
XP_020649062.1      ------------------------------------------------------------      0
XP_008117087.1      ------------------------------------------------------------      0
XP_028564324.1      ------------------------------------------------------------      0
XP_033028155.1      ------------------------------------------------------------      0
XP_025963249.1      ------------------------------------------------------------      0
XP_009668348.1      ------------------------------------------------------------      0
XP_013798935.1      ------------------------------------------------------------      0
XP_025913685.1      ------------------------------------------------------------      0
XP_013042552.1      ------------------------------------------------------------      0
XP_005030458.2      ------------------------------------------------------------      0
XP_032057953.1      ------------------------------------------------------------      0
XP_021232050.1      ------------------------------------------------------------      0
OXB62403.1          ------------------------------------------------------------      0
```

```
OXB81319.1          ------------------------------------------------------------      0
XP_010722007.1      ------------------------------------------------------------      0
XP_015739349.1      ------------------------------------------------------------      0
XP_031455498.1      ------------------------------------------------------------      0
POI27435.1          ------------------------------------------------------------      0
XP_004948120.1      ------------------------------------------------------------      0
XP_032851190.1      ------------------------------------------------------------      0
XP_010007255.1      ------------------------------------------------------------      0
XP_030320702.1      ------------------------------------------------------------      0
XP_010191940.1      ------------------------------------------------------------      0
XP_027737112.1      ------------------------------------------------------------      0
XP_027555032.1      ------------------------------------------------------------      0
XP_032565370.1      RKKLI----AR--------------------LTGRSQKPDGG--GKATRLPP-VSL----      31
XP_027511217.1      CVRLCVRVSIR--------------------MSVRVSVCVPV--RPCVRVSDPVCMCPSA      41
XP_027593499.1      RKRLIA----R--------------------LTGRSQKPDGG--RKATRL-PPVSLLHSS      35
XP_017664924.1      ------------------------------------------------------------      0
XP_029817938.1      ------------------------------------------------------------      0
XP_005058641.1      ------------------------------------------------------------      0
XP_021385804.1      ------------------------------------------------------------      0
XP_030146687.2      ------------------------------------------------------------      0
KAF4796420.1        ------------------------------------------------------------      0
XP_032937581.1      ------------------------------------------------------------      0
XP_031989659.1      ------------------------------------------------------------      0
XP_010402086.1      ------------------------------------------------------------      0
XP_017594069.1      ------------------------------------------------------------      0
XP_014115268.1      ------------------------------------------------------------      28
XP_023797108.1      ------------------------------------------------------------      0
XP_033375614.1      ------------------------------------------------------------      0
XP_014740121.1      CLSVCPSVCPA--------------------MPVCVSVCVSGHARPCACLC--VRLCVRP      70
RLV83430.1          ------------------------------------------------------------      0
XP_009096098.2      ------------------------------------------------------------      27
TRZ15870.1          ------------------------------------------------------------      0
RMB91935.1          ------------------------------------------------------------      0
XP_030820843.1      ------------------------------------------------------------      0
XP_014165179.1      ------------------------------------------------------------      0
XP_026653582.1      ------------------------------------------------------------      0
PKU35975.1          ------------------------------------------------------------      0
XP_014805072.1      ------------------------------------------------------------      0
XP_009818330.1      ------------------------------------------------------------      0
OPJ68307.1          ------------------------------------------------------------      0
XP_008936289.1      ------------------------------------------------------------      0
XP_010287046.1      ------------------------------------------------------------      0
XP_005240140.2      ------------------------------------------------------------      0
XP_005437752.2      ------------------------------------------------------------      0
KFV74811.1          ------------------------------------------------------------      0
XP_010018389.1      ------------------------------------------------------------      0
KQK78711.1          ------------------------------------------------------------      0
XP_009570162.1      ------------------------------------------------------------      0
KFP11268.1          ------------------------------------------------------------      0
KFQ98910.1          ------------------------------------------------------------      0
XP_012985202.3      ------------------------------------------------------------      0
XP_010573388.1      ------------------------------------------------------------      0
XP_029879496.1      ------------------------------------------------------------      0
XP_030361086.1      ------------------------------------------------------------      0
KFM00668.1          ------------------------------------------------------------      0
KAF1479074.1        ------------------------------------------------------------      0
KAF1651161.1        ------------------------------------------------------------      0
KAF1673648.1        ------------------------------------------------------------      0
KAF1493319.1        ------------------------------------------------------------      0
KAF1584157.1        ------------------------------------------------------------      0
KAF1571723.1        ------------------------------------------------------------      0
KAF1533169.1        ------------------------------------------------------------      0
KAF1638955.1        ------------------------------------------------------------      0
KAF1549972.1        ------------------------------------------------------------      0
KAF1606914.1        ------------------------------------------------------------      0
KAF1510957.1        ------------------------------------------------------------      0
KAF1498899.1        ------------------------------------------------------------      0
KAF1411525.1        ------------------------------------------------------------      0
KAF1429205.1        ------------------------------------------------------------      0
XP_005334318.1      ------------------------------------------------------------      0
XP_014395552.1      ------------------------------------------------------------      0
KAB0404854.1        ------------------------------------------------------------      0
XP_007494828.1      ------------------------------------------------------------      0
XP_003764254.1      ------------------------------------------------------------      0
XP_020845489.1      ------------------------------------------------------------      0
XP_027703160.1      ------------------------------------------------------------      0
XP_017523929.1      ------------------------------------------------------------      0
```

XP_008688428.1	--	0
XP_029812166.1	--	0
XP_025749781.1	--	0
XP_004416432.1	--	0
XP_027436262.1	--	0
XP_032284025.1	--	0
XP_006735421.1	--	0
XP_021552166.1	--	0
XP_026361066.1	--	0
XP_034523598.1	--	0
NP_001297113.1	--	0
XP_032215488.1	--	0
VCX31483.1	--	0
XP_022369003.1	--	0
XP_032694248.1	--	0
NP_001041584.1	--	0
XP_025862501.1	--	0
KAF0873564.1	ALSFCQAWMYS---	34
XP_025784751.1	--	0
XP_007075625.1	ALSCCQAWMYS---	34
XP_030189489.1	--	0
XP_019324655.1	ALSCCQAWMYS---	34
XP_023094886.1	ALSCCQAWMYS---	34
XP_026892357.1	--	0
XP_016004457.1	--	0
XP_006912860.1	--	0
XP_011363679.1	--	0
XP_016076060.1	--	0
XP_008148371.1	--	0
XP_006093568.1	--	0
XP_006761601.1	--	0
XP_024426743.1	--	0
XP_019520779.1	--	0
XP_032976539.1	--	0
ELW64270.1	--	0
XP_004427367.1	--	0
XP_008529353.1	--	0
NP_001288165.1	--	0
XP_014711213.1	--	0
XP_012514621.1	--	0
XP_008071525.1	--	0
XP_009005082.1	--	0
XP_010332832.1	--	0
XP_012326058.1	--	0
XP_017378796.1	--	0
XP_032141374.1	--	0
XP_032024018.1	--	0
XP_018891497.2	--	0
XP_030684894.1	--	0
PNJ75904.1	--	0
AAP35868.1	--	0
XP_001149570.1	--	0
XP_003805532.1	--	0
XP_023063823.1	--	0
XP_033060210.1	--	0
XP_010370669.1	--	0
XP_017738496.1	--	0
XP_011782168.1	--	0
EHH23440.1	--	0
XP_003910778.3	--	0
XP_025212649.1	--	0
XP_008019132.1	--	0
XP_011832421.1	--	0
XP_011759618.1	--	0
XP_005579749.1	--	0
XP_011921684.1	--	0
XP_008259391.1	--	0
XP_012782438.1	--	0
VTJ86076.1	--	0
XP_015345533.1	--	0
XP_027786432.1	--	0
XP_026248345.1	--	0
XP_005378209.1	--	0
XP_004466010.1	--	0
XP_020035707.1	--	0
XP_006181802.1	--	0
XP_006207862.1	--	0

```
XP_005891968.1      ------------------------------------------------------------      0
XP_010828662.1      ------------------------------------------------------------      0
XP_019830126.1      ------------------------------------------------------------      0
XP_017914486.1      ------------------------------------------------------------      0
XP_006060266.2      ------------------------------------------------------------      0
XP_020728447.1      ------------------------------------------------------------      0
KAF4008892.1        ------------------------------------------------------------      0
KAB0348059.1        ------------------------------------------------------------      0
KAB0371113.1        ------------------------------------------------------------      0
MBV96963.1          ------------------------------------------------------------      0
XP_007172048.1      ------------------------------------------------------------      0
XP_007452270.1      ------------------------------------------------------------      0
XP_023987108.1      ------------------------------------------------------------      0
XP_004273432.1      ------------------------------------------------------------      0
XP_004328646.1      ------------------------------------------------------------      0
XP_026937670.1      ------------------------------------------------------------      0
XP_030691031.1      ------------------------------------------------------------      0
XP_022414870.1      ------------------------------------------------------------      0
XP_029064651.1      ------------------------------------------------------------      0
XP_024607566.1      ------------------------------------------------------------      0
XP_032496907.1      ------------------------------------------------------------      0
XP_007535559.1      ------------------------------------------------------------      0
XP_031299536.1      ------------------------------------------------------------      0
XP_004666516.1      ------------------------------------------------------------      0
XP_008830963.1      ------------------------------------------------------------      0
XP_005347338.1      ------------------------------------------------------------      0
XP_005069548.1      ------------------------------------------------------------      0
XP_027267894.1      ------------------------------------------------------------      0
OBS80992.1          ------------------------------------------------------------      0
XP_006979812.1      ------------------------------------------------------------      0
XP_028720687.1      ------------------------------------------------------------      0
XP_021489005.1      ------------------------------------------------------------      0
XP_031199589.1      ------------------------------------------------------------      0
XP_028617944.1      ------------------------------------------------------------      0
XP_034347030.1      ------------------------------------------------------------      0
NP_077370.2         ------------------------------------------------------------      0
XP_032766961.1      ------------------------------------------------------------      0
XP_021063186.1      ------------------------------------------------------------      0
NP_001093114.1      ------------------------------------------------------------      0
XP_021028379.1      ------------------------------------------------------------      0
XP_004689305.1      ------------------------------------------------------------      0
XP_004712871.1      ------------------------------------------------------------      0
XP_004382575.1      ------------------------------------------------------------      0
XP_010596188.1      ------------------------------------------------------------      0
XP_006890855.1      ------------------------------------------------------------      0
XP_006834038.1      ------------------------------------------------------------      0
XP_007934716.1      ------------------------------------------------------------      0
XP_012603491.1      ------------------------------------------------------------      0
XP_012663606.1      ------------------------------------------------------------      0
XP_028372812.1      ------------------------------------------------------------      0
XP_008589608.1      ------------------------------------------------------------      0
XP_012865077.1      ------------------------------------------------------------      0
NP_001166178.1      ------------------------------------------------------------      0
XP_004636553.1      ------------------------------------------------------------      0
XP_004856670.1      ------------------------------------------------------------      0
XP_010627744.1      ------------------------------------------------------------      0


NP_509270.1         ------------------------------------------------------------      0
VDO93178.1          ------------------------------------------------------------      0
PAV91580.1          ------------------------------------------------------------      0
VDK46997.1          ------------------------------------------------------------      0
VIO86814.1          ------------------------------------------------------------      0
VDP38785.1          ------------------------------------------------------------      0
KOF68401.1          ------------------------------------------------------------      0
CDJ96026.1          LP-----TTLSLLLLRS----------------TLGHLGHERNTH--ST-----TKKASV      37
EFX76216.1          ------------------------------------------------------------      0
KAF7403848.1        ------------------------------------------------------------      0
KAF7427032.1        ------------------------------------------------------------      0
KAE9417558.1        ------------------------------------------------------------      0
KJH51504.1          ------------------------------------------------------------      0
VDL69795.1          ------------------------------------------------------------      0
VDO32671.1          ------------------------------------------------------------      0
VBB32409.1          ------------------------------------------------------------      0
VDN54565.1          ------------------------------------------------------------      0
EGT55171.1          ------------------------------------------------------------      0
RCN52111.1          ------------------------------------------------------------      0
```

```
RMX54856.1          ------------------------------------------------------------      0
XP_022781674.1      RP-----RGKGYFASRINRYTNSTSSFELVRLVISGSISPNQGPDKTSVSPKPDKRNRRC      133
RNA37099.1          ------------------------------------------------------------      0
KAA0187152.1        AP-----DG---------GYDNYNP---YEE-----LYQPSHNDDGDSN----------V      56
GAU96593.1          ------------------------------------------------------------      0
XP_009043980.1      ------------------------------------------------------------      0
VDM43573.1          MP-----NS---------ALR--------------------------SV----------M      10
XP_003140283.1      ------------------------------------------------------------      0
VDN82010.1          ------------------------------------------------------------      0
VDO31501.1          ------------------------------------------------------------      0
VDP19591.1          ------------------------------------------------------------      0
EDO32053.1          ------------------------------------------------------------      0
TRY67230.1          ------------------------------------------------------------      0
KAF7391312.1        ------------------------------------------------------------      0
GFG30449.1          ------------------------------------------------------------      0
KQS30083.1          ------------------------------------------------------------      0
EDW57583.2          KP-----TAAAAAVTAAATFSNSNSNQSTTA-----TYWLERSSN---------------      68
KNC22799.1          ------------------------------------------------------------      0
RZF44856.1          ------------------------------------------------------------      0
ENN76856.1          ------------------------------------------------------------      0
KAF5300392.1        ------------------------------------------------------------      0
CAB3239999.1        ------------------------------------------------------------      0
PCG77624.1          ------------------------------------------------------------      0
PZC79131.1          ------------------------------------------------------------      0
KAF4083067.1        ------------------------------------------------------------      0
XP_009924659.1      ------------------------------------------------------------      0
XP_010123544.1      ------------------------------------------------------------      0
CBN81618.1          ------------------------------------------------------------      0
KAF3704230.1        ------------------------------------------------------------      0
RUS86578.1          ------------------------------------------------------------      0
XP_034309618.1      ------------------------------------------------------------      0
PIK58946.1          ------------------------------------------------------------      0
VUZ42516.1          ------------------------------------------------------------      0
PAA51166.1          ------------------------------------------------------------      0
TGZ55997.1          ------------------------------------------------------------      0
VDP48851.1          ------------------------------------------------------------      0
PVD38331.1          ------------------------------------------------------------      0
VDK73355.1          ------------------------------------------------------------      0
VDD84963.1          ------------------------------------------------------------      0
VDK42196.1          ------------------------------------------------------------      0
KFD58288.1          ------------------------------------------------------------      0
OUC49089.1          ------------------------------------------------------------      0
EYC26492.1          ------------------------------------------------------------      0
VDK27218.1          ------------------------------------------------------------      0
VDM17286.1          ------------------------------------------------------------      0
VDL91846.1          ------------------------------------------------------------      0
KAE9548540.1        ------------------------------------------------------------      0
PDM74087.1          ------------------------------------------------------------      0
XP_024504322.1      ------------------------------------------------------------      0
PIO52336.1          ------------------------------------------------------------      0
TKR73865.1          ------------------------------------------------------------      0
RLU23395.1          ------------------------------------------------------------      0
XP_002427906.1      ------------------------------------------------------------      0
TMW47392.1          ------------------------------------------------------------      0
VDD83956.1          ------------------------------------------------------------      0
VEL33078.1          ------------------------------------------------------------      0
XP_009019088.1      ------------------------------------------------------------      0
KAE9536378.1        ------------------------------------------------------------      0
KDR23473.1          ------------------------------------------------------------      0
KAF7266914.1        ------------------------------------------------------------      0
KAF4519445.1        ------------------------------------------------------------      0
OXU25983.1          ------------------------------------------------------------      0
TGZ32403.1          ------------------------------------------------------------      0
ELT94491.1          ------------------------------------------------------------      0
VVC39575.1          ----------------------------------------------------------MC      2
RVE49089.1          ------------------------------------------------------------      0
KAB0800277.1        ------------------------------------------------------------      0
TDG52197.1          ------------------------------------------------------------      0
TMW48669.1          ------------------------------------------------------------      0
XP_025896085.1      ------------------------------------------------------------      0
KAF2977017.1        ------------------------------------------------------------      0
XP_009321837.1      ------------------------------------------------------------      0
XP_009979985.1      ------------------------------------------------------------      0
XP_028942374.1      ------------------------------------------------------------      0
PKK19633.1          -----APPR-----PRAPQPQNG-------------------------------------      17
XP_009894240.1      -----LPPCSGSLRSRQPGELER-------------------------------------      46
XP_032820219.1      ------------------------------------------------------------      0
```

XP_029429480.1	--	0
XP_030077572.1	--	0
XP_033774596.1	--	0
ETE72600.1	--	0
XP_018082638.1	LGSNRNSRCQGRNRNECPRIITS--------------YS-----------------RNSA	137
XP_032905410.1	--	0
XP_020773490.1	--	0
XP_033833634.1	--	0
XP_030215795.1	--	0
XP_030602980.1	--	0
XP_004573543.1	--	0
XP_005916159.1	--	0
XP_013132089.1	--	0
XP_031603488.1	--	0
XP_005725536.1	--	0
XP_026038075.1	--	0
CAF96649.1	--	0
XP_023818570.1	--	0
RVE64289.1	--	0
XP_024121971.1	--	0
XP_015817559.1	--	0
XP_015225670.1	--	0
XP_012722063.1	--	0
XP_032431307.1	--	0
XP_014328329.1	--	0
XP_027886578.1	--	0
XP_008426791.1	--	0
XP_014838686.1	--	0
XP_014885967.1	--	0
XP_033954312.1	--	0
XP_034089244.1	--	0
XP_010780064.1	--	0
XP_033970261.1	--	0
XP_029375575.1	--	0
KAF0023022.1	--	0
XP_019952614.1	--	0
XP_034463117.1	--	0
XP_029924656.1	--	0
XP_028323228.1	--	0
TNM84480.1	--	0
XP_029703788.1	--	0
XP_030010368.1	--	0
XP_019725056.1	--	0
XP_034034934.1	--	0
XP_029956880.1	--	0
XP_008331307.3	--	0
XP_031724845.1	--	0
XP_013859395.1	--	0
XP_017275335.1	--	0
XP_020496197.1	--	0
XP_029030400.1	--	0
XP_022060538.1	--	0
XP_023150584.1	--	0
XP_029303892.1	--	0
XP_020454172.1	--	0
TKS83244.1	--	0
XP_022616583.1	--	0
XP_030293172.1	--	0
XP_027129539.1	--	0
XP_034539538.1	--	0
XP_008277330.1	--	0
XP_028276940.1	--	0
XP_023263433.1	--	0
XP_034406336.1	--	0
XP_026184460.1	--	0
KAF1378228.1	--	0
XP_028450365.1	--	0
XP_031163851.1	--	0
XP_032389084.1	--	0
XP_033494682.1	--	0
XP_018544782.1	--	0
XP_026228189.1	--	0
XP_028976606.1	--	0
CAB1352378.1	--	0
XP_023866849.1	--	0
XP_013992832.1	--	0
XP_029546688.1	--	0

XP_021481546.1	--	0
XP_020339889.1	--	0
XP_029481972.1	--	0
KPP68743.1	--	0
XP_023669383.1	--	0
KAA0720096.1	--	0
XP_009293684.1	--	0
XP_026090784.1	--	0
XP_018918715.1	--	0
KAF4098549.1	--	0
XP_016084173.1	--	0
XP_016332759.1	--	0
XP_016398039.1	--	0
XP_026989970.1	--	0
TSQ12698.1	--	0
XP_017347546.1	--	0
XP_026794616.2	--	0
XP_026861889.1	--	0
XP_017575347.1	--	0
XP_022531596.1	--	0
XP_012691019.2	--	0
XP_028839723.1	--	0
XP_030638860.1	--	0
XP_007882964.1	--	0
GCB70244.1	--	0
GCC26242.1	--	0
XP_020387034.1	--	0
XP_028931940.1	--	0
XP_031762490.1	--	0
PIO32240.1	--	0
XP_018425466.1	--	0
XP_006642351.1	--	0
XP_028665372.1	--	0
GCF49814.1	--	0
XP_015277816.1	--	0
XP_005987340.2	--	0
XP_019339534.1	--	0
XP_025063414.1	--	0
XP_019367488.1	--	0
XP_019412078.1	--	0
XP_014434314.1	--	0
XP_007060556.1	--	0
XP_024072175.1	--	0
XP_008170567.1	--	0
XP_034610543.1	--	0
XP_030394127.1	--	0
XP_032651960.1	--	0
XP_025020083.1	--	0
XP_026536833.1	--	0
XP_026564787.1	--	0
XP_032084675.1	--	0
XP_029139341.1	--	0
XP_034281006.1	--	0
XP_020649062.1	--	0
XP_008117087.1	--	0
XP_028564324.1	--	0
XP_033028155.1	--	0
XP_025963249.1	--	0
XP_009668348.1	--	0
XP_013798935.1	--	0
XP_025913685.1	--	0
XP_013042552.1	--	0
XP_005030458.2	--	0
XP_032057953.1	--	0
XP_021232050.1	--	0
OXB62403.1	--	0
OXB81319.1	--	0
XP_010722007.1	--	0
XP_015739349.1	--	0
XP_031455498.1	--	0
POI27435.1	--	0
XP_004948120.1	--	0
XP_032851190.1	--	0
XP_010007255.1	--	0
XP_030320702.1	--	0
XP_010191940.1	--	0
XP_027737112.1	--	0

```
XP_027555032.1      ------------------------------------------------------------      0
XP_032565370.1      ------------------------------------------------------------      31
XP_027511217.1      CPSVRPSVCLAMCLSVCPSVCPS--------------VC-----------------PAMS      70
XP_027593499.1      ---------------AEP-AAAG--------------AS-----------------RAMT      48
XP_017664924.1      ------------------------------------------------------------      0
XP_029817938.1      ------------------------------------------------------------      0
XP_005058641.1      ----------------------------------------------------------MI      2
XP_021385804.1      ------------------------------------------------------------      0
XP_030146687.2      ------------------------------------------------------------      0
KAF4796420.1        ------------------------------------------------------------      0
XP_032937581.1      ------------------------------------------------------------      0
XP_031989659.1      ------------------------------------------------------------      0
XP_010402086.1      ------------------------------------------------------------      0
XP_017594069.1      ------------------------------------------------------------      0
XP_014115268.1      CPAVRVRPC---------------------------------------------------      37
XP_023797108.1      ------------------------------------------------------------      0
XP_033375614.1      ------------------------------------------------------------      0
XP_014740121.1      CPSVRLSVCPSVRLSVCPSVCPA-----------------------------------MP      95
RLV83430.1          ------------------------------------------------------------      0
XP_009096098.2      ----R--PPVPGRVSVCPAVPPS-----------------------------------MS      46
TRZ15870.1          ------------------------------------------------------------      0
RMB91935.1          ------------------------------------------------------------      0
XP_030820843.1      ------------------------------------------------------------      0
XP_014165179.1      ------------------------------------------------------------      0
XP_026653582.1      ------------------------------------------------------------      0
PKU35975.1          ------------------------------------------------------------      0
XP_014805072.1      ------------------------------------------------------------      0
XP_009818330.1      ------------------------------------------------------------      0
OPJ68307.1          ------------------------------------------------------------      0
XP_008936289.1      ------------------------------------------------------------      0
XP_010287046.1      ------------------------------------------------------------      0
XP_005240140.2      ---MK-QERP--------------------------------------------------      6
XP_005437752.2      ---MK-QERP--------------------------------------------------      6
KFV74811.1          ------------------------------------------------------------      0
XP_010018389.1      ------------------------------------------------------------      0
KQK78711.1          ------------------------------------------------------------      0
XP_009570162.1      ------------------------------------------------------------      0
KFP11268.1          ------------------------------------------------------------      0
KFQ98910.1          ------------------------------------------------------------      0
XP_012985202.3      ------------------------------------------------------------      0
XP_010573388.1      ------------------------------------------------------------      0
XP_029879496.1      ------------------------------------------------------------      0
XP_030361086.1      --MAKLQSPP--------------------------------------------------      8
KFM00668.1          ------------------------------------------------------------      0
KAF1479074.1        ------------------------------------------------------------      0
KAF1651161.1        ------------------------------------------------------------      0
KAF1673648.1        ------------------------------------------------------------      0
KAF1493319.1        ------------------------------------------------------------      0
KAF1584157.1        ------------------------------------------------------------      0
KAF1571723.1        ------------------------------------------------------------      0
KAF1533169.1        ------------------------------------------------------------      0
KAF1638955.1        ------------------------------------------------------------      0
KAF1549972.1        ------------------------------------------------------------      0
KAF1606914.1        ------------------------------------------------------------      0
KAF1510957.1        ------------------------------------------------------------      0
KAF1498899.1        ------------------------------------------------------------      0
KAF1411525.1        ------------------------------------------------------------      0
KAF1429205.1        ------------------------------------------------------------      0
XP_005334318.1      ------------------------------------------------------------      0
XP_014395552.1      ------------------------------------------------------------      0
KAB0404854.1        ------------------------------------------------------------      0
XP_007494828.1      ------------------------------------------------------------      0
XP_003764254.1      ------------------------------------------------------------      0
XP_020845489.1      ------------------------------------------------------------      0
XP_027703160.1      ------------------------------------------------------------      0
XP_017523929.1      ------------------------------------------------------------      0
XP_008688428.1      ------------------------------------------------------------      0
XP_029812166.1      ------------------------------------------------------------      0
XP_025749781.1      ------------------------------------------------------------      0
XP_004416432.1      ------------------------------------------------------------      0
XP_027436262.1      ------------------------------------------------------------      0
XP_032284025.1      ------------------------------------------------------------      0
XP_006735421.1      ------------------------------------------------------------      0
XP_021552166.1      ------------------------------------------------------------      0
XP_026361066.1      ------------------------------------------------------------      0
XP_034523598.1      ------------------------------------------------------------      0
NP_001297113.1      ------------------------------------------------------------      0
```

```
XP_032215488.1      ------------------------------------------------------------      0
VCX31483.1          ------------------------------------------------------------      0
XP_022369003.1      ------------------------------------------------------------      0
XP_032694248.1      ------------------------------------------------------------      0
NP_001041584.1      ------------------------------------------------------------      0
XP_025862501.1      ------------------------------------------------------------      0
KAF0873564.1        -------------------FRGG--------------VS-----------------KHMT      44
XP_025784751.1      ------------------------------------------------------------      0
XP_007075625.1      -------------------FWSG--------------VR-----------------KHMT      44
XP_030189489.1      ------------------------------------------------------------      0
XP_019324655.1      -------------------FWSG-------------------------------------      38
XP_023094886.1      -------------------FWSG--------------VR-----------------KHMT      44
XP_026892357.1      ------------------------------------------------------------      0
XP_016004457.1      ------------------------------------------------------------      0
XP_006912860.1      ------------------------------------------------------------      0
XP_011363679.1      ------------------------------------------------------------      0
XP_016076060.1      ------------------------------------------------------------      0
XP_008148371.1      ------------------------------------------------------------      0
XP_006093568.1      ------------------------------------------------------------      0
XP_006761601.1      ------------------------------------------------------------      0
XP_024426743.1      ------------------------------------------------------------      0
XP_019520779.1      ------------------------------------------------------------      0
XP_032976539.1      ------------------------------------------------------------      0
ELW64270.1          ------------------------------------------------------------      0
XP_004427367.1      ------------------------------------------------------------      0
XP_008529353.1      ------------------------------------------------------------      0
NP_001288165.1      ------------------------------------------------------------      0
XP_014711213.1      ------------------------------------------------------------      0
XP_012514621.1      ------------------------------------------------------------      0
XP_008071525.1      ------------------------------------------------------------      0
XP_009005082.1      ------------------------------------------------------------      0
XP_010332832.1      ------------------------------------------------------------      0
XP_012326058.1      ------------------------------------------------------------      0
XP_017378796.1      ------------------------------------------------------------      0
XP_032141374.1      ------------------------------------------------------------      0
XP_032024018.1      ------------------------------------------------------------      0
XP_018891497.2      ------------------------------------------------------------      0
XP_030684894.1      ------------------------------------------------------------      0
PNJ75904.1          ------------------------------------------------------------      0
AAP35868.1          ------------------------------------------------------------      0
XP_001149570.1      ------------------------------------------------------------      0
XP_003805532.1      ------------------------------------------------------------      0
XP_023063823.1      ------------------------------------------------------------      0
XP_033060210.1      ------------------------------------------------------------      0
XP_010370669.1      ------------------------------------------------------------      0
XP_017738496.1      ------------------------------------------------------------      0
XP_011782168.1      ------------------------------------------------------------      0
EHH23440.1          ------------------------------------------------------------      0
XP_003910778.3      ------------------------------------------------------------      0
XP_025212649.1      ------------------------------------------------------------      0
XP_008019132.1      ------------------------------------------------------------      0
XP_011832421.1      ------------------------------------------------------------      0
XP_011759618.1      ------------------------------------------------------------      0
XP_005579749.1      ------------------------------------------------------------      0
XP_011921684.1      ------------------------------------------------------------      0
XP_008259391.1      ------------------------------------------------------------      0
XP_012782438.1      ------------------------------------------------------------      0
VTJ86076.1          ------------------------------------------------------------      0
XP_015345533.1      ------------------------------------------------------------      0
XP_027786432.1      ------------------------------------------------------------      0
XP_026248345.1      ------------------------------------------------------------      0
XP_005378209.1      ------------------------------------------------------------      0
XP_004466010.1      ------------------------------------------------------------      0
XP_020035707.1      ------------------------------------------------------------      0
XP_006181802.1      ------------------------------------------------------------      0
XP_006207862.1      ------------------------------------------------------------      0
XP_005891968.1      ------------------------------------------------------------      0
XP_010828662.1      ------------------------------------------------------------      0
XP_019830126.1      ------------------------------------------------------------      0
XP_017914486.1      ------------------------------------------------------------      0
XP_006060266.2      ------------------------------------------------------------      0
XP_020728447.1      ------------------------------------------------------------      0
KAF4008892.1        ------------------------------------------------------------      0
KAB0348059.1        ------------------------------------------------------------      0
KAB0371113.1        ------------------------------------------------------------      0
MBV96963.1          ------------------------------------------------------------      0
XP_007172048.1      ------------------------------------------------------------      0
```

```
XP_007452270.1      ------------------------------------------------------------      0
XP_023987108.1      ------------------------------------------------------------      0
XP_004273432.1      ------------------------------------------------------------      0
XP_004328646.1      ------------------------------------------------------------      0
XP_026937670.1      ------------------------------------------------------------      0
XP_030691031.1      ------------------------------------------------------------      0
XP_022414870.1      ------------------------------------------------------------      0
XP_029064651.1      ------------------------------------------------------------      0
XP_024607566.1      ------------------------------------------------------------      0
XP_032496907.1      ------------------------------------------------------------      0
XP_007535559.1      ------------------------------------------------------------      0
XP_031299536.1      ------------------------------------------------------------      0
XP_004666516.1      ------------------------------------------------------------      0
XP_008830963.1      ------------------------------------------------------------      0
XP_005347338.1      ------------------------------------------------------------      0
XP_005069548.1      ------------------------------------------------------------      0
XP_027267894.1      ------------------------------------------------------------      0
OBS80992.1          ------------------------------------------------------------      0
XP_006979812.1      ------------------------------------------------------------      0
XP_028720687.1      ------------------------------------------------------------      0
XP_021489005.1      ------------------------------------------------------------      0
XP_031199589.1      ------------------------------------------------------------      0
XP_028617944.1      ------------------------------------------------------------      0
XP_034347030.1      ------------------------------------------------------------      0
NP_077370.2         ------------------------------------------------------------      0
XP_032766961.1      ------------------------------------------------------------      0
XP_021063186.1      ------------------------------------------------------------      0
NP_001093114.1      ------------------------------------------------------------      0
XP_021028379.1      ------------------------------------------------------------      0
XP_004689305.1      ------------------------------------------------------------      0
XP_004712871.1      ------------------------------------------------------------      0
XP_004382575.1      ------------------------------------------------------------      0
XP_010596188.1      ------------------------------------------------------------      0
XP_006890855.1      ------------------------------------------------------------      0
XP_006834038.1      ------------------------------------------------------------      0
XP_007934716.1      ------------------------------------------------------------      0
XP_012603491.1      ------------------------------------------------------------      0
XP_012663606.1      ------------------------------------------------------------      0
XP_028372812.1      ------------------------------------------------------------      0
XP_008589608.1      ------------------------------------------------------------      0
XP_012865077.1      ------------------------------------------------------------      0
NP_001166178.1      ------------------------------------------------------------      0
XP_004636553.1      ------------------------------------------------------------      0
XP_004856670.1      ------------------------------------------------------------      0
XP_010627744.1      ------------------------------------------------------------      0

NP_509270.1         ------------------------------------------------------------      0
VDO93178.1          ------------------------------------------------------------      0
PAV91580.1          ------------------------------------------------------------      0
VDK46997.1          ------------------------------------------------------------      0
VIO86814.1          ------------------------------------------------------------      0
VDP38785.1          ------------------------------------------------------------      0
KOF68401.1          ------------------------------------------------------------      0
CDJ96026.1          PTDQSPTPSN------AQENRSVYISDSDV------------------------------      61
EFX76216.1          ------------------------------------------------------------      0
KAF7403848.1        ------------------------------------------------------------      0
KAF7427032.1        ------------------------------------------------------------      0
KAE9417558.1        ------------------------------------------------------------      0
KJH51504.1          ------------------------------------------------------------      0
VDL69795.1          ------------------------------------------------------------      0
VDO32671.1          ------------------------------------------------------------      0
VBB32409.1          ------------------------------------------------------------      0
VDN54565.1          ------------------------------------------------------------      0
EGT55171.1          ------------------------------------------------------------      0
RCN52111.1          ------------------------------------------------------------      0
RMX54856.1          ------------------------------------------------------------      0
XP_022781674.1      QTDPLYNINIAHLDIRSLKNHKHYILAKEAVRANNIDIFAVSETWLDSTVSDIEVEFLGF      193
RNA37099.1          ------------------------------------------------------------      0
KAA0187152.1        PYDPAHPYQLYASNFPNIKNIKNA------------------------------------      80
GAU96593.1          ------------------------------------------------------------      0
XP_009043980.1      ------------------------------------------------------------      0
VDM43573.1          AAKQTKRVCVVAGRLPRDRERRLV------------------------------------      34
XP_003140283.1      ------------------------------------------------------------      0
VDN82010.1          ------------------------------------------------------------      0
VDO31501.1          ------------------------------------------------------------      0
VDP19591.1          ------------------------------------------------------------      0
```

EDO32053.1	--	0
TRY67230.1	--	0
KAF7391312.1	--	0
GFG30449.1	--	0
KQS30083.1	--	0
EDW57583.2	----SNSINLFHGLWPISVGIVLQL------------LLPALRLWQM-------------	99
KNC22799.1	--	0
RZF44856.1	--	0
ENN76856.1	--	0
KAF5300392.1	--	0
CAB3239999.1	--	0
PCG77624.1	--	0
PZC79131.1	--	0
KAF4083067.1	--	0
XP_009924659.1	--	0
XP_010123544.1	--	0
CBN81618.1	--	0
KAF3704230.1	--	0
RUS86578.1	--	0
XP_034309618.1	--	0
PIK58946.1	--	0
VUZ42516.1	--	0
PAA51166.1	--	0
TGZ55997.1	--	0
VDP48851.1	--	0
PVD38331.1	--	0
VDK73355.1	--	0
VDD84963.1	--	0
VDK42196.1	--	0
KFD58288.1	--	0
OUC49089.1	--	0
EYC26492.1	--	0
VDK27218.1	--	0
VDM17286.1	--	0
VDL91846.1	--	0
KAE9548540.1	--	0
PDM74087.1	--	0
XP_024504322.1	--	0
PIO52336.1	--	0
TKR73865.1	--	0
RLU23395.1	--	0
XP_002427906.1	--	0
TMW47392.1	--	0
VDD83956.1	--	0
VEL33078.1	--	0
XP_009019088.1	--	0
KAE9536378.1	--	0
KDR23473.1	--	0
KAF7266914.1	--	0
KAF4519445.1	--	0
OXU25983.1	--	0
TGZ32403.1	--	0
ELT94491.1	--	0
VVC39575.1	KTTEATKLKLREMTVTQEVDIVKRISKK---------------------VRNY-------	34
RVE49089.1	--	0
KAB0800277.1	--	0
TDG52197.1	--	0
TMW48669.1	--	0
XP_025896085.1	--	0
KAF2977017.1	--	0
XP_009321837.1	--	0
XP_009979985.1	--	0
XP_028942374.1	--	0
PKK19633.1	--	17
XP_009894240.1	--	46
XP_032820219.1	--	0
XP_029429480.1	--	0
XP_030077572.1	--	0
XP_033774596.1	--	0
ETE72600.1	--	0
XP_018082638.1	Q-----------------LRGIIQ------------------K---------------YW	147
XP_032905410.1	--	0
XP_020773490.1	--	0
XP_033833634.1	--	0
XP_030215795.1	--	0
XP_030602980.1	--	0
XP_004573543.1	--	0

```
XP_005916159.1      ------------------------------------------------------------      0
XP_013132089.1      ------------------------------------------------------------      0
XP_031603488.1      ------------------------------------------------------------      0
XP_005725536.1      ------------------------------------------------------------      0
XP_026038075.1      ------------------------------------------------------------      0
CAF96649.1          ------------------------------------------------------------      0
XP_023818570.1      ------------------------------------------------------------      0
RVE64289.1          ------------------------------------------------------------      0
XP_024121971.1      ------------------------------------------------------------      0
XP_015817559.1      ------------------------------------------------------------      0
XP_015225670.1      ------------------------------------------------------------      0
XP_012722063.1      ------------------------------------------------------------      0
XP_032431307.1      ------------------------------------------------------------      0
XP_014328329.1      ------------------------------------------------------------      0
XP_027886578.1      ------------------------------------------------------------      0
XP_008426791.1      ------------------------------------------------------------      0
XP_014838686.1      ------------------------------------------------------------      0
XP_014885967.1      ------------------------------------------------------------      0
XP_033954312.1      --------------------------------------------------------M-R-      2
XP_034089244.1      --------------------------------------------------------M-R-      2
XP_010780064.1      --------------------------------------------------------M-D-      2
XP_033970261.1      --------------------------------------------------------M-D-      2
XP_029375575.1      -------------------------------------------MALQRHPLTPFSHS-T-      15
KAF0023022.1        ----------------------------------------------MHTPTMSLSDT-N-      12
XP_019952614.1      ------------------------------------------------------------      0
XP_034463117.1      ------------------------------------------------------------      0
XP_029924656.1      ------------------------------------------------------------      0
XP_028323228.1      ------------------------------------------------------------      0
TNM84480.1          ------------------------------------------------------------      0
XP_029703788.1      ------------------------------------------------------------      0
XP_030010368.1      ------------------------------------------------------------      0
XP_019725056.1      ------------------------------------------------------------      0
XP_034034934.1      ------------------------------------------------------------      0
XP_029956880.1      ------------------------------------------------------------      0
XP_008331307.3      ------------------------------------------------------------      0
XP_031724845.1      ------------------------------------------------------------      0
XP_013859395.1      ------------------------------------------------------------      0
XP_017275335.1      ------------------------------------------------------------      0
XP_020496197.1      ------------------------------------------------------------      0
XP_029030400.1      ------------------------------------------------------------      0
XP_022060538.1      ------------------------------------------------------------      0
XP_023150584.1      ------------------------------------------------------------      0
XP_029303892.1      -----------------------------------------------MDI----------      3
XP_020454172.1      ------------------------------------------------------------      0
TKS83244.1          ------------------------------------------------------------      0
XP_022616583.1      ---------------------------------------------MIYDP----------      5
XP_030293172.1      ------------------------------------------------------------      0
XP_027129539.1      ------------------------------------------------------------      0
XP_034539538.1      ------------------------------------------------------------      0
XP_008277330.1      ------------------------------------------------------------      0
XP_028276940.1      ------------------------------------------------------------      0
XP_023263433.1      ------------------------------------------------------------      0
XP_034406336.1      ------------------------------------------------------------      0
XP_026184460.1      ------------------------------------------------------------      0
KAF1378228.1        ------------------------------------------------------------      0
XP_028450365.1      ------------------------------------------------------------      0
XP_031163851.1      ------------------------------------------------------------      0
XP_032389084.1      ------------------------------------------------------------      0
XP_033494682.1      ------------------------------------------------------------      0
XP_018544782.1      ------------------------------------------------------------      0
XP_026228189.1      ------------------------------------------------------------      0
XP_028976606.1      ------------------------------------------------------------      0
CAB1352378.1        ------------------------------------------------------------      0
XP_023866849.1      ------------------------------------------------------------      0
XP_013992832.1      ------------------------------------------------------------      0
XP_029546688.1      ------------------------------------------------------------      0
XP_021481546.1      ------------------------------------------------------------      0
XP_020339889.1      ------------------------------------------------------------      0
XP_029481972.1      ------------------------------------------------------------      0
KPP68743.1          ------------------------------------------------------------      0
XP_023669383.1      ------------------------------------------------------------      0
KAA0720096.1        ------------------------------------------------------------      0
XP_009293684.1      ------------------------------------------------------------      0
XP_026090784.1      ------------------------------------------------------------      0
XP_018918715.1      ------------------------------------------------------------      0
KAF4098549.1        ------------------------------------------------------------      0
XP_016084173.1      ------------------------------------------------------------      0
```

```
XP_016332759.1      ------------------------------------------------------------      0
XP_016398039.1      ------------------------------------------------------------      0
XP_026989970.1      ------------------------------------------------------------      0
TSQ12698.1          ------------------------------------------------------------      0
XP_017347546.1      ------------------------------------------------------------      0
XP_026794616.2      ------------------------------------------------------------      0
XP_026861889.1      ------------------------------------------------------------      0
XP_017575347.1      ------------------------------------------------------------      0
XP_022531596.1      ------------------------------------------------------------      0
XP_012691019.2      ------------------------------------------------------------      0
XP_028839723.1      ------------------------------------------------------------      0
XP_030638860.1      ------------------------------------------------------------      0
XP_007882964.1      ------------------------------------------------------------      0
GCB70244.1          ------------------------------------------------------------      0
GCC26242.1          ------------------------------------------------------------      0
XP_020387034.1      ------------------------------------------------------------      0
XP_028931940.1      ------------------------------------------------------------      0
XP_031762490.1      ------------------------------------------------------------      0
PIO32240.1          ------------------------------------------------------------      0
XP_018425466.1      ------------------------------------------------------------      0
XP_006642351.1      ------------------------------------------------------------      0
XP_028665372.1      ------------------------------------------------------------      0
GCF49814.1          ------------------------------------------------------------      0
XP_015277816.1      ------------------------------------------------------------      0
XP_005987340.2      ------------------------------------------------------------      0
XP_019339534.1      ------------------------------------------------------------      0
XP_025063414.1      ------------------------------------------------------------      0
XP_019367488.1      ------------------------------------------------------------      0
XP_019412078.1      ------------------------------------------------------------      0
XP_014434314.1      ------------------------------------------------------------      0
XP_007060556.1      ------------------------------------------------------------      0
XP_024072175.1      ------------------------------------------------------------      0
XP_008170567.1      ------------------------------------------------------------      0
XP_034610543.1      ------------------------------------------------------------      0
XP_030394127.1      ------------------------------------------------------------      0
XP_032651960.1      ------------------------------------------------------------      0
XP_025020083.1      ------------------------------------------------------------      0
XP_026536833.1      ------------------------------------------------------------      0
XP_026564787.1      ------------------------------------------------------------      0
XP_032084675.1      ------------------------------------------------------------      0
XP_029139341.1      ------------------------------------------------------------      0
XP_034281006.1      ------------------------------------------------------------      0
XP_020649062.1      ------------------------------------------------------------      0
XP_008117087.1      ------------------------------------------------------------      0
XP_028564324.1      ------------------------------------------------------------      0
XP_033028155.1      ------------------------------------------------------------      0
XP_025963249.1      ------------------------------------------------------------      0
XP_009668348.1      ------------------------------------------------------------      0
XP_013798935.1      ------------------------------------------------------------      0
XP_025913685.1      ------------------------------------------------------------      0
XP_013042552.1      ------------------------------------------------------------      0
XP_005030458.2      ------------------------------------------------------------      0
XP_032057953.1      ------------------------------------------------------------      0
XP_021232050.1      ------------------------------------------------------------      0
OXB62403.1          ------------------------------------------------------------      0
OXB81319.1          ------------------------------------------------------------      0
XP_010722007.1      ------------------------------------------------------------      0
XP_015739349.1      ------------------------------------------------------------      0
XP_031455498.1      ------------------------------------------------------------      0
POI27435.1          ------------------------------------------------------------      0
XP_004948120.1      ------------------------------------------------------------      0
XP_032851190.1      ------------------------------------------------------------      0
XP_010007255.1      ------------------------------------------------------------      0
XP_030320702.1      ------------------------------------------------------------      0
XP_010191940.1      ------------------------------------------------------------      0
XP_027737112.1      ------------------------------------------------------------      0
XP_027555032.1      ------------------------------------------------------------      0
XP_032565370.1      ------------------------------------------------------------      31
XP_027511217.1      PHKQP--LGI----P-----------WP--------------RDPLSLAPTPAAGH--TG      97
XP_027593499.1      PT--A--LGA----L-----------LA--------------LLPLALML----------      65
XP_017664924.1      ------------------------------------------------------------      0
XP_029817938.1      ------------------------------------------------------------      0
XP_005058641.1      AR---------------------------------------------LIGRSQKPD--GG      15
XP_021385804.1      ------------------------------------------------------------      0
XP_030146687.2      ------------------------MQRE--------------RLIAR-LTGRSQKP--DG      19
KAF4796420.1        ------------------------------------------------------------      0
XP_032937581.1      ------------------------MQRE--------------RLIARLTGRSQKPD--GG      20
```

```
XP_031989659.1      ------------------------------------------------------------      0
XP_010402086.1      ------------------------------------------------------------      0
XP_017594069.1      ------------------------------------------------------------      0
XP_014115268.1      ------------------------------------------------------------      37
XP_023797108.1      ------------------------------------------------------------      0
XP_033375614.1      ------------------------------------------------------------      0
XP_014740121.1      VRV-P--VCV----SV----------------------------CLSVCPAMPDCV--SV      118
RLV83430.1          ------------------------------------------------------------      0
XP_009096098.2      VRT-P--DEE----FRRQEGGGSGLDKV--------------KLWKLVFANFPD-----V      80
TRZ15870.1          ------------------------------------------------------------      0
RMB91935.1          ------------------------------------------------------------      0
XP_030820843.1      -MQ-R--ERL----IARLIGR----------------------------SQKPDVV---G      21
XP_014165179.1      -ME----------------MS----------------------------QQGPRGC---A      12
XP_026653582.1      ------------------------------------------------------------      0
PKU35975.1          ------------------------------------------------------------      0
XP_014805072.1      ------------------------------------------------------------      0
XP_009818330.1      ------------------------------------------------------------      0
OPJ68307.1          ------------------------------------------------------------      0
XP_008936289.1      ------------------------------------------------------------      0
XP_010287046.1      ------------------------------------------------------------      0
XP_005240140.2      ------------------------------------------------IV---RLI--GR      13
XP_005437752.2      ------------------------------------------------IV---RLI--GR      13
KFV74811.1          ------------------------------------------------------------      0
XP_010018389.1      ------------------------------------------------------------      0
KQK78711.1          ------------------------------------------------------------      0
XP_009570162.1      ------------------------------------------------------------      0
KFP11268.1          ------------------------------------------------------------      0
KFQ98910.1          ------------------------------------------------------------      0
XP_012985202.3      ------------------------------------------------------------      0
XP_010573388.1      ------------------------------------------------------------      0
XP_029879496.1      ------------------------------------------------------------      0
XP_030361086.1      ------------------------------------------------APRRKHCI--GR      18
KFM00668.1          ------------------------------------------------------------      0
KAF1479074.1        ------------------------------------------------------------      0
KAF1651161.1        ------------------------------------------------------------      0
KAF1673648.1        ------------------------------------------------------------      0
KAF1493319.1        ------------------------------------------------------------      0
KAF1584157.1        ------------------------------------------------------------      0
KAF1571723.1        ------------------------------------------------------------      0
KAF1533169.1        ------------------------------------------------------------      0
KAF1638955.1        ------------------------------------------------------------      0
KAF1549972.1        ------------------------------------------------------------      0
KAF1606914.1        ------------------------------------------------------------      0
KAF1510957.1        ------------------------------------------------------------      0
KAF1498899.1        ------------------------------------------------------------      0
KAF1411525.1        ------------------------------------------------------------      0
KAF1429205.1        ------------------------------------------------------------      0
XP_005334318.1      ------------------------------------------------------------      0
XP_014395552.1      ------------------------------------------------------------      0
KAB0404854.1        ------------------------------------------------------------      0
XP_007494828.1      ------------------------------------------------------------      0
XP_003764254.1      ------------------------------------------------------------      0
XP_020845489.1      ------------------------------------------------------------      0
XP_027703160.1      ------------------------------------------------------------      0
XP_017523929.1      ------------------------------------------------------------      0
XP_008688428.1      ------------------------------------------------------------      0
XP_029812166.1      ------------------------------------------------------------      0
XP_025749781.1      ------------------------------------------------------------      0
XP_004416432.1      ------------------------------------------------------------      0
XP_027436262.1      ------------------------------------------------------------      0
XP_032284025.1      ------------------------------------------------------------      0
XP_006735421.1      ------------------------------------------------------------      0
XP_021552166.1      ------------------------------------------------------------      0
XP_026361066.1      ------------------------------------------------------------      0
XP_034523598.1      ------------------------------------------------------------      0
NP_001297113.1      ------------------------------------------------------------      0
XP_032215488.1      ------------------------------------------------------------      0
VCX31483.1          ------------------------------------------------------------      0
XP_022369003.1      ------------------------------------------------------------      0
XP_032694248.1      ------------------------------------------------------------      0
NP_001041584.1      ------------------------------------------------------------      0
XP_025862501.1      ------------------------------------------------------------      0
KAF0873564.1        S----------------EKGSPISLSSTE---------HSKTKLRLTAIRRGSIAHQ-IW      78
XP_025784751.1      ------------------------------------------------------------      0
XP_007075625.1      S----------------QKGSPISLSWTK---------HSRTKLWLIAIQRDSIR---TW      76
XP_030189489.1      ------------------------------------------------------------      0
XP_019324655.1      ------------------------------------------------------G---TW      41
```

```
XP_023094886.1      S----------------EKGSPISLSWTK---------PSGTKLWLIAIQRDSIR---TW      76
XP_026892357.1      ------------------------------------------------------------      0
XP_016004457.1      ------------------------------------------------------------      0
XP_006912860.1      ------------------------------------------------------------      0
XP_011363679.1      ------------------------------------------------------------      0
XP_016076060.1      ------------------------------------------------------------      0
XP_008148371.1      ------------------------------------------------------------      0
XP_006093568.1      ------------------------------------------------------------      0
XP_006761601.1      ------------------------------------------------------------      0
XP_024426743.1      ------------------------------------------------------------      0
XP_019520779.1      ------------------------------------------------------------      0
XP_032976539.1      ------------------------------------------------------------      0
ELW64270.1          ------------------------------------------------------------      0
XP_004427367.1      ------------------------------------------------------------      0
XP_008529353.1      ------------------------------------------------------------      0
NP_001288165.1      ------------------------------------------------------------      0
XP_014711213.1      ------------------------------------------------------------      0
XP_012514621.1      ------------------------------------------------------------      0
XP_008071525.1      ------------------------------------------------------------      0
XP_009005082.1      ------------------------------------------------------------      0
XP_010332832.1      ------------------------------------------------------------      0
XP_012326058.1      ------------------------------------------------------------      0
XP_017378796.1      ------------------------------------------------------------      0
XP_032141374.1      ------------------------------------------------------------      0
XP_032024018.1      ------------------------------------------------------------      0
XP_018891497.2      ------------------------------------------------------------      0
XP_030684894.1      ------------------------------------------------------------      0
PNJ75904.1          ------------------------------------------------------------      0
AAP35868.1          ------------------------------------------------------------      0
XP_001149570.1      ------------------------------------------------------------      0
XP_003805532.1      ------------------------------------------------------------      0
XP_023063823.1      ------------------------------------------------------------      0
XP_033060210.1      ------------------------------------------------------------      0
XP_010370669.1      ------------------------------------------------------------      0
XP_017738496.1      ------------------------------------------------------------      0
XP_011782168.1      ------------------------------------------------------------      0
EHH23440.1          ------------------------------------------------------------      0
XP_003910778.3      ------------------------------------------------------------      0
XP_025212649.1      ------------------------------------------------------------      0
XP_008019132.1      ------------------------------------------------------------      0
XP_011832421.1      ------------------------------------------------------------      0
XP_011759618.1      ------------------------------------------------------------      0
XP_005579749.1      ------------------------------------------------------------      0
XP_011921684.1      ------------------------------------------------------------      0
XP_008259391.1      ------------------------------------------------------------      0
XP_012782438.1      ------------------------------------------------------------      0
VTJ86076.1          ------------------------------------------------------------      0
XP_015345533.1      ------------------------------------------------------------      0
XP_027786432.1      ------------------------------------------------------------      0
XP_026248345.1      ------------------------------------------------------------      0
XP_005378209.1      ------------------------------------------------------------      0
XP_004466010.1      ------------------------------------------------------------      0
XP_020035707.1      ------------------------------------------------------------      0
XP_006181802.1      ------------------------------------------------------------      0
XP_006207862.1      ------------------------------------------------------------      0
XP_005891968.1      ------------------------------------------------------------      0
XP_010828662.1      ------------------------------------------------------------      0
XP_019830126.1      ------------------------------------------------------------      0
XP_017914486.1      ------------------------------------------------------------      0
XP_006060266.2      ------------------------------------------------------------      0
XP_020728447.1      ------------------------------------------------------------      0
KAF4008892.1        ------------------------------------------------------------      0
KAB0348059.1        ------------------------------------------------------------      0
KAB0371113.1        ------------------------------------------------------------      0
MBV96963.1          ------------------------------------------------------------      0
XP_007172048.1      ------------------------------------------------------------      0
XP_007452270.1      ------------------------------------------------------------      0
XP_023987108.1      ------------------------------------------------------------      0
XP_004273432.1      ------------------------------------------------------------      0
XP_004328646.1      ------------------------------------------------------------      0
XP_026937670.1      ------------------------------------------------------------      0
XP_030691031.1      ------------------------------------------------------------      0
XP_022414870.1      ------------------------------------------------------------      0
XP_029064651.1      ------------------------------------------------------------      0
XP_024607566.1      ------------------------------------------------------------      0
XP_032496907.1      ------------------------------------------------------------      0
XP_007535559.1      ------------------------------------------------------------      0
```

XP_031299536.1	--	0
XP_004666516.1	--	0
XP_008830963.1	--	0
XP_005347338.1	--	0
XP_005069548.1	--	0
XP_027267894.1	--	0
OBS80992.1	--	0
XP_006979812.1	--	0
XP_028720687.1	--	0
XP_021489005.1	--	0
XP_031199589.1	--	0
XP_028617944.1	--	0
XP_034347030.1	--	0
NP_077370.2	--	0
XP_032766961.1	--	0
XP_021063186.1	--	0
NP_001093114.1	--	0
XP_021028379.1	--	0
XP_004689305.1	--	0
XP_004712871.1	--	0
XP_004382575.1	--	0
XP_010596188.1	--	0
XP_006890855.1	--	0
XP_006834038.1	--	0
XP_007934716.1	--	0
XP_012603491.1	--	0
XP_012663606.1	--	0
XP_028372812.1	--	0
XP_008589608.1	--	0
XP_012865077.1	--	0
NP_001166178.1	--	0
XP_004636553.1	--	0
XP_004856670.1	--	0
XP_010627744.1	--	0

NP_509270.1	---MYYKISYLLSI--------	11
VDO93178.1	--MVNWRLRDAWSM--HLVAML	18
PAV91580.1	--	0
VDK46997.1	--	0
VIO86814.1	---MLLEYVITQ--NFLQII	15
VDP38785.1	---M--QFDDLR	7
KOF68401.1	--	0
CDJ96026.1	------STGPSANASDLLKERTS-------N--------DQLVYSL----LS--NQSQV-	93
EFX76216.1	--	0
KAF7403848.1	--	0
KAF7427032.1	--	0
KAE9417558.1	--	0
KJH51504.1	--	0
VDL69795.1	--	0
VDO32671.1	--	0
VBB32409.1	--	0
VDN54565.1	--	0
EGT55171.1	--	0
RCN52111.1	--	0
RMX54856.1	--	0
XP_022781674.1	HLHRLDTSIQTGAGVCIFTKQYF-------K--------VECLYQLSYIAAS--GLHMLW	236
RNA37099.1	--	0
KAA0187152.1	-----KIPG-SESGKPIFSEINS-------D---------ESVT--SDDINS--GHRDE-	113
GAU96593.1	--	0
XP_009043980.1	--	0
VDM43573.1	-----RLVQ-QCSGR-------------------------------APALGR--AGRQFE	55
XP_003140283.1	--	0
VDN82010.1	--	0
VDO31501.1	--	0
VDP19591.1	--	0
EDO32053.1	--	0
TRY67230.1	--	0
KAF7391312.1	--	0
GFG30449.1	--	0
KQS30083.1	---M--K-KPSR	6
EDW57583.2	-----QLQL-QQRAATVLRAIAN-------S--------TTAIISYANAIGM--QLLHSW	136
KNC22799.1	--	0
RZF44856.1	--	0
ENN76856.1	--	0
KAF5300392.1	--	0
CAB3239999.1	--	0

PCG77624.1	--	0
PZC79131.1	--	0
KAF4083067.1	--	0
XP_009924659.1	--	0
XP_010123544.1	--	0
CBN81618.1	--	0
KAF3704230.1	--	0
RUS86578.1	--MGLG-------R	5
XP_034309618.1	--	0
PIK58946.1	---MKYFLRYLLLVVVTILA--	17
VUZ42516.1	--	0
PAA51166.1	--	0
TGZ55997.1	--	0
VDP48851.1	--	0
PVD38331.1	--	0
VDK73355.1	--	0
VDD84963.1	--	0
VDK42196.1	--	0
KFD58288.1	---M	1
OUC49089.1	------------------MPEFD-------I-----------------LSDISER-GELK	17
EYC26492.1	----------------MLFLLFS-------I-----------------VAK-----STSD	15
VDK27218.1	--	0
VDM17286.1	--	0
VDL91846.1	--	0
KAE9548540.1	--	0
PDM74087.1	--	0
XP_024504322.1	--	0
PIO52336.1	--	0
TKR73865.1	--	0
RLU23395.1	--	0
XP_002427906.1	--	0
TMW47392.1	--	0
VDD83956.1	--	0
VEL33078.1	--	0
XP_009019088.1	--	0
KAE9536378.1	------------------MPSID-----------------------PVAGDGDDCHNNNN	19
KDR23473.1	--	0
KAF7266914.1	----------------MK------------N--SL------------------------T	6
KAF4519445.1	--------METMEQKTMR------------R--CKV---------------LSACVSALH	23
OXU25983.1	----------------MR------------Q--VLA---------------L-------L	8
TGZ32403.1	----------------MR------------T--TV-------------------------	5
ELT94491.1	--	0
VVC39575.1	-----KKEFDKKKKKCLKRQAIN-------N--VITENAFAGGYWLRVTAGGRGTISAVH	80
RVE49089.1	--	0
KAB0800277.1	--	0
TDG52197.1	--	0
TMW48669.1	--	0
XP_025896085.1	--MA---HAALRVLL-VLLPLA	16
KAF2977017.1	--	0
XP_009321837.1	--	0
XP_009979985.1	--	0
XP_028942374.1	--	0
PKK19633.1	------RGARAR---------AV---------------R---------------------	26
XP_009894240.1	------KELERR---------EL-----------AAS-RARP---PATFGALL-ALLPLA	75
XP_032820219.1	------------------------------------MEDRETVTKGGSGGALL-------	17
XP_029429480.1	--	0
XP_030077572.1	--	0
XP_033774596.1	--	0
ETE72600.1	--MAFNRLPHQ---------LA	11
XP_018082638.1	NI--LGQDPRLR---------DS-----------LPSQPSFAFKRGPCLGSIL-S-PSLF	183
XP_032905410.1	--	0
XP_020773490.1	--	0
XP_033833634.1	---MP-------	2
XP_030215795.1	--	0
XP_030602980.1	--	0
XP_004573543.1	--	0
XP_005916159.1	--	0
XP_013132089.1	--	0
XP_031603488.1	--	0
XP_005725536.1	--	0
XP_026038075.1	--	0
CAF96649.1	--	0
XP_023818570.1	--	0
RVE64289.1	------------------------------------MDEGGSDAWWLCRSVRH-QSHF--	21
XP_024121971.1	--	0
XP_015817559.1	--	0
XP_015225670.1	--	0

```
XP_012722063.1      ------------------------------------------------------------      0
XP_032431307.1      ------------------------------------------------------------      0
XP_014328329.1      ------------------------------------------------------------      0
XP_027886578.1      ------------------------------------------------------------      0
XP_008426791.1      ------------------------------------------------------------      0
XP_014838686.1      ------------------------------------------------------------      0
XP_014885967.1      ------------------------------------------------------------      0
XP_033954312.1      ----------------------------------ASS-----------------------      5
XP_034089244.1      ----------------------------------ASS-----------------------      5
XP_010780064.1      ----------------------------------LVEGYGG--YVS--------------      12
XP_033970261.1      ----------------------------------LVEGYGG--YVS--------------      12
XP_029375575.1      ----------------------------------SRISQGTESYAFS----------R--      29
KAF0023022.1        ----------------------------------Q-V--------LM----------A--      17
XP_019952614.1      ------------------------------------------------------------      0
XP_034463117.1      ------------------------------------------------------------      0
XP_029924656.1      ------------------------------------------------------------      0
XP_028323228.1      ------------------------------------------------------------      0
TNM84480.1          ------------------------------------------------------------      0
XP_029703788.1      ------------------------------------------------------------      0
XP_030010368.1      ------------------------------------------------------------      0
XP_019725056.1      ------------------------------------------------------------      0
XP_034034934.1      ------------------------------------------------------------      0
XP_029956880.1      ------------------------------------------------------------      0
XP_008331307.3      ----------------------------------MRWTCKGGSWNRRVSAFLR-SFIR--      23
XP_031724845.1      ---------------------------------------MGVRHPV--------------      7
XP_013859395.1      ------------------------------------------------------------      0
XP_017275335.1      ------------------------------------------------------------      0
XP_020496197.1      ------------------------------------------------------------      0
XP_029030400.1      ------------------------------------------------------------      0
XP_022060538.1      --------------------------------------------------MRP-------      3
XP_023150584.1      --------------------------------------------------MRP-------      3
XP_029303892.1      ----------------------------------V-DGYGG----YGCEASR---GLP--      19
XP_020454172.1      ------------------------------------------------------------      0
TKS83244.1          ---------------------------------------------------------M--      1
XP_022616583.1      ----------------------------------VTESQRGVSTVFQLHASQP-AAAL--      28
XP_030293172.1      ----------------------------------MRLSSAGTALIF--------------      12
XP_027129539.1      ----------------------------------MKLSSAWTAFIL--------------      12
XP_034539538.1      ----------------------------------MRLSAAWTTLLF--------------      12
XP_008277330.1      ----------------------------------MRLSAAWTALAF--------------      12
XP_028276940.1      ----------------------------------MRISPGWTALVS--------------      12
XP_023263433.1      ----------------------------------MRLSPAWTTLVF--------------      12
XP_034406336.1      ----------------------------------MRLSSAWSALIF--------------      12
XP_026184460.1      ----------------------------------MRFSPACTVLVF--------------      12
KAF1378228.1        ----------------------------------MRLSSARTALIF--------------      12
XP_028450365.1      ----------------------------------MRLSSARTALIF--------------      12
XP_031163851.1      ----------------------------------MRLSSAGTALIF--------------      12
XP_032389084.1      ----------------------------------MRLSPARTALIF--------------      12
XP_033494682.1      ----------------------------------MRVSSAWKALVF--------------      12
XP_018544782.1      ----------------------------------MRLSSAWTALVL--------------      12
XP_026228189.1      ----------------------------------MRLSSAWMVLVF--------------      12
XP_028976606.1      ------------------------------------------------------------      0
CAB1352378.1        ------------------------------------------------------------      0
XP_023866849.1      ------------------------------------------------------------      0
XP_013992832.1      ------------------------------------------------------------      0
XP_029546688.1      ------------------------------------------------------------      0
XP_021481546.1      ------------------------------------------------------------      0
XP_020339889.1      ------------------------------------------------------------      0
XP_029481972.1      ------------------------------------------------------------      0
KPP68743.1          ------------------------------------------------------------      0
XP_023669383.1      ------------------------------------------------------------      0
KAA0720096.1        ------------------------------------------------------------      0
XP_009293684.1      ------------------------------------------------------------      0
XP_026090784.1      ------------------------------------------------------------      0
XP_018918715.1      ------------------------------------------------------------      0
KAF4098549.1        ------------------------------------------------------------      0
XP_016084173.1      ------------------------------------------------------------      0
XP_016332759.1      ------------------------------------------------------------      0
XP_016398039.1      ------------------------------------------------------------      0
XP_026989970.1      ------------------------------------------------------------      0
TSQ12698.1          ------------------------------------------------------------      0
XP_017347546.1      ------------------------------------------------------------      0
XP_026794616.2      ------------------------------------------------------------      0
XP_026861889.1      ------------------------------------------------------------      0
XP_017575347.1      ------------------------------------------------------------      0
XP_022531596.1      ------------------------------------------------------------      0
XP_012691019.2      ------------------------------------------------------------      0
XP_028839723.1      ------------------------------------------------------------      0
```

```
XP_030638860.1      ------------------------------------------------------------      0
XP_007882964.1      ------------------------------------------------------------      0
GCB70244.1          ------------------------------------------------------------      0
GCC26242.1          ------------------------------------------------------------      0
XP_020387034.1      ------------------------------------------------------------      0
XP_028931940.1      ------------------------------------------------------------      0
XP_031762490.1      ------------------------------------------------------------      0
PIO32240.1          ------------------------------------------------------------      0
XP_018425466.1      ------------------------------------------------------------      0
XP_006642351.1      ---------------------------------------------------MS-QDFG--      6
XP_028665372.1      ------------------------------------------------------------      0
GCF49814.1          ------------------------------------------------------------      0
XP_015277816.1      ------------------------------------------------------------      0
XP_005987340.2      ------------------------------------------------------------      0
XP_019339534.1      ------------------------------------------------------------      0
XP_025063414.1      ------------------------------------------------------------      0
XP_019367488.1      ------------------------------------------------------------      0
XP_019412078.1      ------------------------------------------------------------      0
XP_014434314.1      ------------------------------------------------------------      0
XP_007060556.1      ------------------------------------------------------------      0
XP_024072175.1      ------------------------------------------------------------      0
XP_008170567.1      ------------------------------------------------------------      0
XP_034610543.1      ------------------------------------------------------------      0
XP_030394127.1      ------------------------------------------------------------      0
XP_032651960.1      ------------------------------------------------------------      0
XP_025020083.1      ------------------------------------------------------------      0
XP_026536833.1      ------------------------------------------------------------      0
XP_026564787.1      ------------------------------------------------------------      0
XP_032084675.1      ------------------------------------------------------------      0
XP_029139341.1      ------------------------------------------------------------      0
XP_034281006.1      ------------------------------------------------------------      0
XP_020649062.1      ------------------------------------------------------------      0
XP_008117087.1      ------------------------------------------------------------      0
XP_028564324.1      ------------------------------------------------------------      0
XP_033028155.1      ------------------------------------------------------------      0
XP_025963249.1      ------------------------------------------------------------      0
XP_009668348.1      ------------------------------------------------------------      0
XP_013798935.1      ------------------------------------------------------------      0
XP_025913685.1      ------------------------------------------------------------      0
XP_013042552.1      ------------------------------------------------------------      0
XP_005030458.2      ------------------------------------------------------------      0
XP_032057953.1      ------------------------------------------------------------      0
XP_021232050.1      ------------------------------------------------------------      0
OXB62403.1          ------------------------------------------------------------      0
OXB81319.1          ------------------------------------------------------------      0
XP_010722007.1      ------------------------------------------------------------      0
XP_015739349.1      ------------------------------------------------------------      0
XP_031455498.1      ------------------------------------------------------------      0
POI27435.1          ------------------------------------------------------------      0
XP_004948120.1      ------------------------------------------------------------      0
XP_032851190.1      ------------------------------------------------------------      0
XP_010007255.1      ------------------------------------------------------------      0
XP_030320702.1      ------------------------------------------------------------      0
XP_010191940.1      ------------------------------------------------------------      0
XP_027737112.1      ------------------------------------------------------------      0
XP_027555032.1      ------------------------------------------------------------      0
XP_032565370.1      ------------------------------------------LRSLLCRAGSS-GA----      44
XP_027511217.1      R---SQRSAPAV--PLWTDKNELFS-FNYNNNEKRPFPPFWPKLRLCCPGAAR-AP----      146
XP_027593499.1      -------QSPGK--GCFLERSSS-----YSEVVHLGEAGAWESSGVE----PK-GT----      102
XP_017664924.1      ----------------------------------------------------M-LL----      3
XP_029817938.1      ------------------------------------------------------------      0
XP_005058641.1      GK--ATRVLPTSLLRSVLR------------------------------------R----      33
XP_021385804.1      ------------------------------------------------------------      0
XP_030146687.2      GG--SQKVPPASLLRAV-------------------------------------LRQA--      38
KAF4796420.1        ------------------------------------------------------------      0
XP_032937581.1      GK--ATRVPPASLLRSVL------------------------------------RRAG--      40
XP_031989659.1      ------------------------------------------------------------      0
XP_010402086.1      ------------------------------------------------------------      0
XP_017594069.1      ------------------------------------------------------------      0
XP_014115268.1      ------------------------------------------------------------      37
XP_023797108.1      ------------------------------------------------------------      0
XP_033375614.1      ------------------------------------------------------------      0
XP_014740121.1      RV--SGRVPPPEEPPIIAPAAE---------------------P------VLP-SPAP--      146
RLV83430.1          ------------------------------------------------------------      0
XP_009096098.2      -R--TTRVPPASLLRSVL------------------------------------RRAG--      99
TRZ15870.1          ------------------------------------------------------------      0
RMB91935.1          ------------------------------------------------------------      0
```

```
XP_030820843.1      -K--ATRVPPASPLRS----------------------------------VPA-E-RA--      40
XP_014165179.1      -G--SRCVCADSTLSAVQQLDL---------------------LMGCGSGTPD-EEFG--      45
XP_026653582.1      ------------------------------------------------------------      0
PKU35975.1          ------------------------------------------------------------      0
XP_014805072.1      ------------------------------------------------------------      0
XP_009818330.1      ------------------------------------------------------------      0
OPJ68307.1          ------------------------------------------------------------      0
XP_008936289.1      ------------------------------------------------------------      0
XP_010287046.1      ------------------------------------------------------------      0
XP_005240140.2      SH--KPDGGGKVSKE------------------CCLPPCSGSFLRGGSRLGRR-QAAA--      50
XP_005437752.2      SH--KPDGGGKVSKE------------------CCLPPCSGSFLRGGSRLGRR-QAAA--      50
KFV74811.1          ------------------------------------------------------------      0
XP_010018389.1      -----------------------------------------------MA--WK-ERAV--      8
KQK78711.1          ------------------------------------------------------------      0
XP_009570162.1      ------------------------------------------------------------      0
KFP11268.1          ------------------------------------------------------------      0
KFQ98910.1          ------------------------------------------------------------      0
XP_012985202.3      ------------------------------------------------------------      0
XP_010573388.1      ------------------------------------------------------------      0
XP_029879496.1      ------------------------------------------------------------      0
XP_030361086.1      GA--S----EHRERA------------------ACL-------LLGATA--AA-EADS--      42
KFM00668.1          ------------------------------------------------------------      0
KAF1479074.1        ------------------------------------------------------------      0
KAF1651161.1        ------------------------------------------------------------      0
KAF1673648.1        ------------------------------------------------------------      0
KAF1493319.1        ------------------------------------------------------------      0
KAF1584157.1        ------------------------------------------------------------      0
KAF1571723.1        ------------------------------------------------------------      0
KAF1533169.1        ------------------------------------------------------------      0
KAF1638955.1        ------------------------------------------------------------      0
KAF1549972.1        ------------------------------------------------------------      0
KAF1606914.1        ------------------------------------------------------------      0
KAF1510957.1        ------------------------------------------------------------      0
KAF1498899.1        ------------------------------------------------------------      0
KAF1411525.1        ------------------------------------------------------------      0
KAF1429205.1        ------------------------------------------------------------      0
XP_005334318.1      ------------------------------------------------------------      0
XP_014395552.1      ------------------------------------------------------------      0
KAB0404854.1        ------------------------------------------------------------      0
XP_007494828.1      ------------------------------------------------------------      0
XP_003764254.1      ------------------------------------------------------------      0
XP_020845489.1      ------------------------------------------------------------      0
XP_027703160.1      ------------------------------------------------------------      0
XP_017523929.1      ------------------------------------------------------------      0
XP_008688428.1      ------------------------------------------------------------      0
XP_029812166.1      ---------------------------------------------------MS-HPEA--      6
XP_025749781.1      ------------------------------------------------------------      0
XP_004416432.1      ------------------------------------------------------------      0
XP_027436262.1      ------------------------------------------------------------      0
XP_032284025.1      ------------------------------------------------------------      0
XP_006735421.1      ------------------------------------------------------------      0
XP_021552166.1      ------------------------------------------------------------      0
XP_026361066.1      ------------------------------------------------------------      0
XP_034523598.1      ------------------------------------------------------------      0
NP_001297113.1      ------------------------------------------------------------      0
XP_032215488.1      ------------------------------------------------------------      0
VCX31483.1          ------------------------------------------------------------      0
XP_022369003.1      ------------------------------------------------------------      0
XP_032694248.1      ------------------------------------------------------------      0
NP_001041584.1      ------------------------------------------------------------      0
XP_025862501.1      ------------------------------------------------------------      0
KAF0873564.1        DI--NEGSSPGPLPAHRLHPCSRGPCSALHPHLKLLPGX-GAAWA-GCLSARA-ALRP--      131
XP_025784751.1      ------------------------------------------------------------      0
XP_007075625.1      RL--SSAGGPSAVGGCSLRGVEGGPEE----AGRLGQEA-GRVWA-ACFSARL-TLRP--      125
XP_030189489.1      ------------------------------------------------------------      0
XP_019324655.1      RL--SSAGGPSAVGGCSLRGVEGGPEE----AGRLGHEA-GRVWA-ACFSARL-TLRP--      90
XP_023094886.1      RL--SSAGGPSAVGGCSLRGVEGGPEE----AGRLGREA-GRVWA-ACLSARL-TLRP--      125
XP_026892357.1      ------------------------------------------------------------      0
XP_016004457.1      ------------------------------------------------------------      0
XP_006912860.1      ------------------------------------------------------------      0
XP_011363679.1      ------------------------------------------------------------      0
XP_016076060.1      ------------------------------------------------------------      0
XP_008148371.1      ------------------------------------------------------------      0
XP_006093568.1      ------------------------------------------------------------      0
XP_006761601.1      ------------------------------------------------------------      0
XP_024426743.1      ------------------------------------------------------------      0
XP_019520779.1      ------------------------------------------------------------      0
```

XP_032976539.1	--	0
ELW64270.1	--	0
XP_004427367.1	--	0
XP_008529353.1	--	0
NP_001288165.1	--	0
XP_014711213.1	--	0
XP_012514621.1	--	0
XP_008071525.1	--	0
XP_009005082.1	--	0
XP_010332832.1	--	0
XP_012326058.1	--	0
XP_017378796.1	--------------------------------MRLAEGRQAGPW-------RA-SPWA--	18
XP_032141374.1	--------------------------------MRLAEGRQAGPW-------RA-SPWA--	18
XP_032024018.1	--	0
XP_018891497.2	--	0
XP_030684894.1	--	0
PNJ75904.1	--	0
AAP35868.1	--	0
XP_001149570.1	--	0
XP_003805532.1	--	0
XP_023063823.1	--	0
XP_033060210.1	--	0
XP_010370669.1	--	0
XP_017738496.1	--	0
XP_011782168.1	--	0
EHH23440.1	--	0
XP_003910778.3	--	0
XP_025212649.1	--	0
XP_008019132.1	--	0
XP_011832421.1	--	0
XP_011759618.1	--	0
XP_005579749.1	--	0
XP_011921684.1	--	0
XP_008259391.1	--	0
XP_012782438.1	--	0
VTJ86076.1	--	0
XP_015345533.1	--	0
XP_027786432.1	--	0
XP_026248345.1	--	0
XP_005378209.1	--	0
XP_004466010.1	--	0
XP_020035707.1	--	0
XP_006181802.1	--	0
XP_006207862.1	--	0
XP_005891968.1	--	0
XP_010828662.1	--	0
XP_019830126.1	--	0
XP_017914486.1	--	0
XP_006060266.2	--	0
XP_020728447.1	--	0
KAF4008892.1	--	0
KAB0348059.1	--	0
KAB0371113.1	--	0
MBV96963.1	--	0
XP_007172048.1	--	0
XP_007452270.1	--	0
XP_023987108.1	--	0
XP_004273432.1	--	0
XP_004328646.1	--	0
XP_026937670.1	--	0
XP_030691031.1	--	0
XP_022414870.1	--	0
XP_029064651.1	--	0
XP_024607566.1	--	0
XP_032496907.1	--	0
XP_007535559.1	--	0
XP_031299536.1	--	0
XP_004666516.1	--	0
XP_008830963.1	--	0
XP_005347338.1	--	0
XP_005069548.1	--	0
XP_027267894.1	--	0
OBS80992.1	--	0
XP_006979812.1	--	0
XP_028720687.1	--	0
XP_021489005.1	--	0
XP_031199589.1	-------------------------------------MCSLAVW------GTH-PEWL--	14

```
XP_028617944.1      ------------------------------------------------------------      0
XP_034347030.1      ------------------------------------------------------------      0
NP_077370.2         ------------------------------------------------------------      0
XP_032766961.1      ------------------------------------------------------------      0
XP_021063186.1      ------------------------------------------------------------      0
NP_001093114.1      ------------------------------------------------------------      0
XP_021028379.1      ------------------------------------------------------------      0
XP_004689305.1      ------------------------------------------------------------      0
XP_004712871.1      ------------------------------------------------------------      0
XP_004382575.1      ------------------------------------------------------------      0
XP_010596188.1      ------------------------------------------------------------      0
XP_006890855.1      ------------------------------------------------------------      0
XP_006834038.1      ------------------------------------------------------------      0
XP_007934716.1      ------------------------------------------------------------      0
XP_012603491.1      ------------------------------------------------------------      0
XP_012663606.1      ------------------------------------------------------------      0
XP_028372812.1      ------------------------------------------------------------      0
XP_008589608.1      ------------------------------------------------------------      0
XP_012865077.1      ------------------------------------------------------------      0
NP_001166178.1      ------------------------------------------------------------      0
XP_004636553.1      ------------------------------------------------------------      0
XP_004856670.1      ------------------------------------------------------------      0
XP_010627744.1      ------------------------------------------------------------      0


NP_509270.1         -----------------------------SILLFFAKC----------EK-V--------      23
VDO93178.1          C-----------------------LCVESHLVCFPLGC----------QL-AA-------      37
PAV91580.1          -----------------------------------MGT----------QV-EL-------      7
VDK46997.1          ------------------------------------------------------------      0
VIO86814.1          A-VQI--SHGV--TSKARIGSKQNEVL---FDTFGKSN----------ML-ANG------      50
VDP38785.1          NVCPI--RFHR-LLLISSSAGSWNAVLAFPIVDFTSAS----------DP-PRSLMI---      50
KOF68401.1          ------------------------------------------------------------      0
CDJ96026.1          --SPR--PNGR-KRSTTTSLTTQNTRLSRFGTEFSSGG----------LP-PE---I---      131
EFX76216.1          ------------------------------------------------------------      0
KAF7403848.1        ------------------------------------------------------------      0
KAF7427032.1        ------------------------------------------------MS-PS---L---      5
KAE9417558.1        ------------------------------------------------------------      0
KJH51504.1          ------------------------------------------------------------      0
VDL69795.1          ------------------------------------------------------------      0
VDO32671.1          ------------------------------------------------------------      0
VBB32409.1          ------------------------------------------------------------      0
VDN54565.1          ---------------------------------------------------------MAL      3
EGT55171.1          ------------------------------------------------------------      0
RCN52111.1          ----------------------------------------------------------MR      2
RMX54856.1          -------------------M----PSGDPNITGFEHD------------V-QQ---NK--      19
XP_022781674.1      VKIQI--RNSR-SFLVCTVY----KPPDASTLCFDTD------------Q-QN---SRSG      273
RNA37099.1          ------------------MF----LTYSAV---FN--T----------YV-LN---V-SE      18
KAA0187152.1        ---EK--PEP---QPVTEVY----GDMLPH---FD---------------------S-SN      136
GAU96593.1          ------------------MT----ASTTLF---FL---------------------V-TL      13
XP_009043980.1      ------------------------------------------------------------      0
VDM43573.1          RSLER--CLT---PPVLPMM----ATRRRC---FL---------------------I-LL      81
XP_003140283.1      ------------------------------------------------------------      0
VDN82010.1          -----------------------------M---YL---------------------I---      4
VDO31501.1          -----------------------------M---YL---------------------I---      4
VDP19591.1          ------------------------------------------------------------      0
EDO32053.1          ------------------------------------------------------------      0
TRY67230.1          ------------------------------------------------------------      0
KAF7391312.1        ------------------------------------------------------------      0
GFG30449.1          -----------------------------MTVSFRY-------------------VCGRP      12
KQS30083.1          TRLEN--PF---------CS----GQERTRTMSFPQPH----------SL-PEATANGGK      40
EDW57583.2          KRIRN--TNSS--NNNNSSS----GNSSNNNNSSSSAT----------LL-INGLNK-HS      176
KNC22799.1          ------------------------------------------------------------      0
RZF44856.1          ---------------------------------------------------ME--AV-SS      6
ENN76856.1          --------------------------M--------YFS----------YL-PT--KK-ET      12
KAF5300392.1        ---------------MTEVK----INM--------KGM----------SL------I-AP      16
CAB3239999.1        -------------------------------------M----------HF--------PP      5
PCG77624.1          ------------------------------------------------------------      0
PZC79131.1          ------------------MG----GRA--------RRS----------HL------A-AP      13
KAF4083067.1        ---------------------------------------------------------MCR      3
XP_009924659.1      ------------------------------VEIFPDPF----------L-------IQVE      13
XP_010123544.1      ---------------------------------MQRAF----------I-------AILT      10
CBN81618.1          -----------------------------------MPM----------M-------IMCL      8
KAF3704230.1        ---------------------------------------------------------MCP      3
RUS86578.1          YSKQN--KRDK-----TRIS----HNENEIDAF-NSNK----------L-----------      32
XP_034309618.1      -----------------------------ML--YHLLT----------T-------KAAL      12
PIK58946.1          FN----------------------KA--SCKVF-KSRI----------T-------SLSH      35
VUZ42516.1          -----------------------------MRDLWIGKF----------V---I---LLAI      15
```

PAA51166.1	-----------------------------MPNIFLAVL----------L---------LL	12
TGZ55997.1	---------------------------MNATVWWITVL----------LC-AV---HFPE	19
VDP48851.1	--	0
PVD38331.1	--	0
VDK73355.1	------------------------------MENNMVSR----------A-------ELVV	13
VDD84963.1	---------------------------------MLLAS----------L---S---FLVV	11
VDK42196.1	--	0
KFD58288.1	WLSA--------------------KE-DEPISYFPVFV----------R---F---LIII	24
OUC49089.1	HNLQ--------------------KA-NKPNNNKS-----------------N---ASTL	36
EYC26492.1	YPAN--------------------RSPTDRSGWGRMLT----------R---Q---VFVI	39
VDK27218.1	-----------------------------MNEWTKISG-------------------ILL	12
VDM17286.1	--	0
VDL91846.1	-----------------------------MADWSKTLK----------Y-------LP--	12
KAE9548540.1	---M-KF---LLFW	7
PDM74087.1	-----------------------------------MIL----------RS-PL---LACI	11
XP_024504322.1	--------------------------MLCII---KIFF----------F--IL---IL--	14
PIO52336.1	-----------------------------------MYF----------Q--LL---I--A	8
TKR73865.1	-----------------------------MT---GMSL----------R--IL---ITLS	13
RLU23395.1	------------------------------------MY----------FC-LR---LVRL	10
XP_002427906.1	-----------------------------MRLIDVKGK----------KK-KK---KKNF	17
TMW47392.1	-----------------------------MDFLRYSGM-------------GV---TLAI	15
VDD83956.1	-----------------------------CNK-----H----------NC-IL---KPNL	12
VEL33078.1	--	0
XP_009019088.1	-----------------------------MIHIFH----------------VI---LKIY	12
KAE9536378.1	NSSSI--K--Q-----FRVR----RRRRGCAQ--------------------L---TSSL	43
KDR23473.1	--	0
KAF7266914.1	NNIDD--T--E-----------------IDHGILGALA----------NI-TF---LLNY	31
KAF4519445.1	HGVQR--E--A-----------------HDGSWVAGTG----------A---------AS	43
OXU25983.1	FSV---------------------------------VV----------A---------VT	16
TGZ32403.1	LTV---------------------------------LL----------N---------VV	13
ELT94491.1	-----------------------------MDSWLNSV--------------------VVA	11
VVC39575.1	CTIAR--R--G-----KRLN----ETMRRCNLTLAGA--------------------IIL	107
RVE49089.1	--------------------------MPRRAAPPA-----------------------LA	11
KAB0800277.1	---------------------------M--ELVIA-----------------------CI	8
TDG52197.1	--------------------------MG--SVLFA-----------------------AV	9
TMW48669.1	--------------------------MG--SLLFA-----------------------AV	9
XP_025896085.1	TTL---	19
KAF2977017.1	--	0
XP_009321837.1	--	0
XP_009979985.1	--	0
XP_028942374.1	--	0
PKK19633.1	--LSA---------PG--RS--ETSFCTTAT---APL-----------------------	45
XP_009894240.1	WTLQG---------AGWYGM--RTRLCGETL---TLL-----KTD--------------V	102
XP_032820219.1	-------------------L--QHEVFHLKL---HLF-----LLL-KERAAQRALRN-L-	45
XP_029429480.1	------MYFHLEAFQGCIKL--GDKTMYMGL---DFE-----NYFGAKATDKEATDF-LP	43
XP_030077572.1	--	0
XP_033774596.1	------------------------MRMFIII---T-L-----RYEGMHRVENRSVQ----	23
ETE72600.1	AKLNPSGSK---TF----------SRDKLC-------------------------RL-SV	32
XP_018082638.1	SESESPGPTHWLTFKGCYRC--GASRCGTC-------------------------RY-MK	215
XP_032905410.1	--	0
XP_020773490.1	------------------------MRTSLICMVL--LSVAL--YG--------------A	18
XP_033833634.1	--S-----------C---------LVYSCLCMWDVLLSDTVSIAEESTPE-----QT-RA	34
XP_030215795.1	--------------------------MRLSVPWTALVF------------------L-LV	15
XP_030602980.1	-----------------MDV--XDMGAGHAQAFHQ-S------------------DR-HT	21
XP_004573543.1	--------------------------MRFSSAWTALC------------------FL-LI	15
XP_005916159.1	--------------------------MRFSSAWTALC------------------FL-LI	15
XP_013132089.1	--------------------------MRFSSAWTALC------------------FL-LI	15
XP_031603488.1	-----------------MDV--EDIGARHPEAFRQ-S------------------DR-QT	21
XP_005725536.1	-----------------MDV--EDIGARHPEAFHQ-S------------------DR-QT	21
XP_026038075.1	-----------------MDV--EDIGARHPEAFHQ-S------------------DR-QT	21
CAF96649.1	--	0
XP_023818570.1	---MI-HS	4
RVE64289.1	--E-----------Q--KPS--RTRTMRASPARTALL------------------FL-LI	45
XP_024121971.1	--------------------------MRASSAWAALL------------------FI-LI	15
XP_015817559.1	---R	1
XP_015225670.1	--------------------------MRLSSGWTILL------------------YL-LI	15
XP_012722063.1	--------------------------MRLSSGWTILL------------------HL-LI	15
XP_032431307.1	--------------------------MRLSSGWTTLI------------------YL-LL	15
XP_014328329.1	--------------------------MRLSSGWTTLI------------------YL-LL	15
XP_027886578.1	--------------------------MRLSSGWTTLI------------------YL-LL	15
XP_008426791.1	--------------------------MRLSSGWTALL------------------YL-LL	15
XP_014838686.1	--------------------------MRLSSGWTALL------------------YL-LL	15
XP_014885967.1	--------------------------MRLSSGWTALL------------------YL-LL	15
XP_033954312.1	-------------------------------AWTALI------------------LL-FI	15
XP_034089244.1	-------------------------------AWTALL------------------LL-FI	15
XP_010780064.1	--------------E--------ASRGLSSERQEDFQ------------------VK-RH	31
XP_033970261.1	--------------E--------ASRGLSSERQEGFQ------------------VK-RH	31

```
XP_029375575.1      --S-----------T--KHM--KDQIEDQIGAWDIVLPGPIKD------------PC---      57
KAF0023022.1        --D-----------E-------SGLMDIVARRWSTTVSGVPRPFI---RA-----AG-RF      48
XP_019952614.1      -------------------------------------MFA-------------------N      4
XP_034463117.1      --------------------------MRLSSAWTTLVFLL-------------------I      15
XP_029924656.1      --------------------------MRLSSGCVAFVVL-----------------L-LI      16
XP_028323228.1      --------------------------MRHSTLW-AALTL-----------------V-FI      15
TNM84480.1          --------------------------MRASPAWT-IFIS-----------------L-VL      15
XP_029703788.1      --------------------------MRASPAWT-IFIS-----------------L-VL      15
XP_030010368.1      -----------------------MKASSSSSAW-AVLIL-----------------L-LI      18
XP_019725056.1      --------------------------MRPTSAWTTLVLL-----------------V-VI      16
XP_034034934.1      --------------------------------------M-----------------G-EV      4
XP_029956880.1      ---------------------------MRRSSVWAALLF-----------------L-LI      15
XP_008331307.3      --T-----------D--RLH--SRSSRMRASSAAAAMIL-----------------L-LI      48
XP_031724845.1      ---------------------------------AFHQSS-----------------S-QP      16
XP_013859395.1      ---------------------------MRLSSGWTALLS-----------------L-LI      15
XP_017275335.1      ---------------------------MRLSSGWTALLY-----------------L-LI      15
XP_020496197.1      ---------------------------MRVASAWTALLF-----------------L-LI      15
XP_029030400.1      ---------------------------MRLSAAGAVLAC-----------------L-LI      15
XP_022060538.1      ----------------------------SAAAWT-ALVL-----------------L-LL      16
XP_023150584.1      ----------------------------SAAAWT-ALVL-----------------L-LL      16
XP_029303892.1      --S-----------E--R-------QAHFHSRRRKPLNC-----------------H-HE      39
XP_020454172.1      ----------------------------MRLSACTALVF-----------------L-LI      14
TKS83244.1          --E-----------D-----------MGVMH--AEAFHQ-----------------N-GT      17
XP_022616583.1      --S-----------D--K-------EEGRRSGWTLPHEL-----------------S-DT      48
XP_030293172.1      ---------------------------------LL------------------------I      15
XP_027129539.1      ---------------------------------LL------------------------I      15
XP_034539538.1      ---------------------------------LL------------------------M      15
XP_008277330.1      ---------------------------------LL------------------------L      15
XP_028276940.1      ---------------------------------LL------------------------I      15
XP_023263433.1      ---------------------------------LL------------------------I      15
XP_034406336.1      ---------------------------------LL------------------------I      15
XP_026184460.1      ---------------------------------LL------------------------I      15
KAF1378228.1        ---------------------------------LL------------------------I      15
XP_028450365.1      ---------------------------------LL------------------------I      15
XP_031163851.1      ---------------------------------LL------------------------I      15
XP_032389084.1      ---------------------------------LL------------------------I      15
XP_033494682.1      ---------------------------------LL------------------------I      15
XP_018544782.1      ---------------------------------LL------------------------I      15
XP_026228189.1      ---------------------------------LL------------------------I      15
XP_028976606.1      --------------------------MRISALWIGVIW------------------L-LI      15
CAB1352378.1        --------------------------MRVSALWTALVL------------------L-LI      15
XP_023866849.1      --------------------------MRVSALWTALVL------------------L-LI      15
XP_013992832.1      --------------------------MRVSALWTALVL------------------L-LI      15
XP_029546688.1      --------------------------MRVSALWTALVL------------------L-LI      15
XP_021481546.1      --------------------------MRVSALWTALVL------------------L-LI      15
XP_020339889.1      --------------------------MRVSALWTALVL------------------L-LI      15
XP_029481972.1      --------------------------MRVSALWTALVL------------------L-LI      15
KPP68743.1          ------------------------------------------------------------      0
XP_023669383.1      --------------------------MKLSALWTALG-------------------A-LI      14
KAA0720096.1        ------------------------------------------------------------      0
XP_009293684.1      --------------------------MRSSALWTALC-------------------I-CM      14
XP_026090784.1      --------------------------MKCSALWTALF-------------------I-CL      14
XP_018918715.1      --------------------------MKCSALWTALC-------------------V-CV      14
KAF4098549.1        --------------------------MKCSALWTALC-------------------V-CM      14
XP_016084173.1      --------------------------MKCSALWTALC-------------------V-CM      14
XP_016332759.1      --------------------------MKCSAPWTALC-------------------V-CM      14
XP_016398039.1      --------------------------MKCSALWTALC-------------------V-CM      14
XP_026989970.1      --------------------------MRTLVVWTAIS-------------------V-CV      14
TSQ12698.1          --------------------------MRTLVVWTALS-------------------V-YV      14
XP_017347546.1      -------------------------------MWMALS-------------------V-CA      9
XP_026794616.2      --------------------------MRTLVVWTALS-------------------V-CV      14
XP_026861889.1      --------------------------MKTLALWTALS-------------------V-CM      14
XP_017575347.1      --------------------------MQPLTLWTALS-------------------V-CM      14
XP_022531596.1      --------------------------MQPLALWTALS-------------------V-CT      14
XP_012691019.2      ------------------------------------------------------------      0
XP_028839723.1      --------------------------MRILALWMALI-------------------M-FL      14
XP_030638860.1      ------------------------------------------------------------      0
XP_007882964.1      ------------------------------------------------------------      0
GCB70244.1          ------------------------------------------------------------      0
GCC26242.1          --------------------------MNFYS-FWASVP------------------L-IF      14
XP_020387034.1      ------------------------------------MA------------------N-LR      5
XP_028931940.1      --------------------------MPHPG-HRPLLA------------------L-LL      14
XP_031762490.1      ------------------MF--PAHYSIHCV-VLIL-S--------------------L-      17
PIO32240.1          ------------------------------------------------------------      0
XP_018425466.1      ------------------MV--PRKFCIQPN-VIVP-A------------------L-F-      18
XP_006642351.1      --R-----------ILLAKI--LQCKMRYPA-LWTTLA------------------L-LL      31
XP_028665372.1      --------------------------MRSSA-LWTALG------------------L-FL      14
```

```
GCF49814.1          ------------------MI--P-----QEV----------------------------T    7
XP_015277816.1      ------------------MA--P-----KET-LLLFLI------------------C-LP    15
XP_005987340.2      ------------------------------------------------------------    0
XP_019339534.1      --------------------------MVLPA-WWALLT------------------L-AL    14
XP_025063414.1      --------------------------MVLSA-WWALLT------------------L-AL    14
XP_019367488.1      --------------------------MVLSA-WWALLT------------------L-AL    14
XP_019412078.1      --------------------------MVLSA-RWALLT------------------L-AL    14
XP_014434314.1      --------------------------MTSSV-PWAFLA------------------L-LL    14
XP_007060556.1      ---------------------------------MQQIT------------------E-LI    8
XP_024072175.1      ------------------------------------------------------------    0
XP_008170567.1      --------------------------MTPSA-LRAFLA------------------L-LL    14
XP_034610543.1      --------------------------MTPSA-LRAFLA------------------L-LL    14
XP_030394127.1      --------------------------MIPSA-LWAFLA------------------L-LL    14
XP_032651960.1      --------------------------MTPSA-LWAFLA------------------L-LL    14
XP_025020083.1      --------------------------MARSA-LWVSLF------------------L-LA    14
XP_026536833.1      --------------------------MACSA-LRVSLL------------------L-LA    14
XP_026564787.1      --------------------------MACSA-LRVFLI------------------L-LA    14
XP_032084675.1      ------------------------------------------------------------    0
XP_029139341.1      --------------------------MARSA-LGVSLL------------------L-LA    14
XP_034281006.1      --------------------------MARSA-LGVSLL------------------L-LA    14
XP_020649062.1      --------------------------MAPSV-LWIPLL------------------L-LL    14
XP_008117087.1      --------------------------MAPSA-LWISLL------------------L-LL    14
XP_028564324.1      ------------------ML--PKVLMAPSA-LRVSLL------------------L-LF    20
XP_033028155.1      -------------------L--PKVLMAPSA-LGVSLL------------------L-LF    19
XP_025963249.1      ------------------------------------------------------------    0
XP_009668348.1      --------------------------MAG-----AVLR----------------------    7
XP_013798935.1      ------------------------------------------------------------    0
XP_025913685.1      --------------------------MRPRRLIARLIR------------------H-FQ    15
XP_013042552.1      --------------------------MVLAA-LTALLS------------------L-LP    14
XP_005030458.2      --------------------------MVLVA-LMALLS------------------L-LP    14
XP_032057953.1      ------------------------------------------------------------    0
XP_021232050.1      -------------------------MALVAL-VSLLLA------------------L-LL    15
OXB62403.1          -------------------------MVLVAL-MSLLLA------------------P-LP    15
OXB81319.1          ------------------------------------------------------------    0
XP_010722007.1      ------------------------MVLVVAV-MLLLLA------------------L-LP    16
XP_015739349.1      ------------------------MVLVVPI-VLLLLA------------------L-LP    16
XP_031455498.1      ------------------------MVLMVAV-MLLLLA------------------L-LP    16
POI27435.1          ------------------------------------------------------------    0
XP_004948120.1      ------------------------MVLVVAV-VLLLLA------------------L-LP    16
XP_032851190.1      --------------------------MTPAA-LGVLLA------------------L-LP    14
XP_010007255.1      --------------------------MVPMP-FGVLLA------------------L-LP    14
XP_030320702.1      ------------------------------------------------------------    0
XP_010191940.1      ----------------------------MAA-LGVLLA------------------L-LP    12
XP_027737112.1      --------------------------------MCKLLP------------------E-CE    9
XP_027555032.1      --------------------------MLLLN-LDGKPR------------------L-CE    14
XP_032565370.1      -----------------------SRAMTLTA-L-GALL------------------A-LL    60
XP_027511217.1      -----------------------SCTIGNVS-L-QA----------------------LA    159
XP_027593499.1      -----------------------SCL------D-NT----------------------SS    110
XP_017664924.1      -----------------------FNL------D-GK----------------------LR    11
XP_029817938.1      ------------------------------------------------------------    0
XP_005058641.1      ------------------AG--ASASMVPTA-LGALLA------------------L-LP    53
XP_021385804.1      --------------------------MVPTA-LGALLA------------------L-LP    14
XP_030146687.2      -----------------GGA--ASASMVPAA-LGALLA------------------L-LP    59
KAF4796420.1        --------------------------MVPTA-LGALLA------------------L-LP    14
XP_032937581.1      -----------------GGW--SQGSMLPTA-LGALLA------------------L-LP    61
XP_031989659.1      --------------------------MVPTA-LGALLA------------------L-LP    14
XP_010402086.1      --------------------------MVPTA-LGALLA------------------L-LP    14
XP_017594069.1      ------------------------------------------------------------    0
XP_014115268.1      -----------------------------------------------------------P    38
XP_023797108.1      ------------------------------------------------------------    0
XP_033375614.1      ------------------------------------------------------------    0
XP_014740121.1      -----------------AAV--I---------TGPS-R------------------R-SA    158
RLV83430.1          ------------------------------------ML------------------L-FK    5
XP_009096098.2      -----------------GGC--AGGSMVPRA-LGPLLA------------------L-LP    120
TRZ15870.1          --------------------------MVPTA-LGALLA------------------L-LP    14
RMB91935.1          ------------------------------------------------------------    0
XP_030820843.1      -----------------AAA--GSGPMVPAA-LGALLA------------------L-LP    61
XP_014165179.1      -----------------RQE--GGGSSVDK----VKLR------------------K-LV    63
XP_026653582.1      ------------------------------------------------------------    0
PKU35975.1          --------------------------MVPIA-LGALLA------------------L-LP    14
XP_014805072.1      --------------------------MVPIA-LGALLA------------------L-LP    14
XP_009818330.1      ------------------------------------------------------------    0
OPJ68307.1          --------------------------MVPAA-LGALLA------------------L-LP    14
XP_008936289.1      -------------------------MTPPII-VGTLLA------------------L-LP    15
XP_010287046.1      ------------------------------------------------------------    0
XP_005240140.2      --G-----------SQ-QSG--GRRAMTPTT-LGAFLA------------------L-LP    74
XP_005437752.2      --G-----------SQ-QSG--GRRAMTPTT-LGAFLA------------------L-LP    74
```

KFV74811.1	--	0
XP_010018389.1	--G-----------NL-SGG--SES-----------LV------------------V-PA	22
KQK78711.1	--------------------------MTPTA-LGVLLA------------------L-LP	14
XP_009570162.1	---------------------------MFTA-FGALLA------------------L-LP	13
KFP11268.1	--	0
KFQ98910.1	--	0
XP_012985202.3	--------------------------MTPTA-LGVLLA------------------L-LP	14
XP_010573388.1	---------------------------MPAA-LGVLLA------------------L-LP	13
XP_029879496.1	--	0
XP_030361086.1	--G-----------GC-SQR--SSTAMTPAT-LSVLLA------------------L-LP	66
KFM00668.1	--	0
KAF1479074.1	--	0
KAF1651161.1	--	0
KAF1673648.1	--	0
KAF1493319.1	--	0
KAF1584157.1	--	0
KAF1571723.1	--	0
KAF1533169.1	--	0
KAF1638955.1	--	0
KAF1549972.1	--	0
KAF1606914.1	--	0
KAF1510957.1	--	0
KAF1498899.1	--	0
KAF1411525.1	--	0
KAF1429205.1	--	0
XP_005334318.1	------------------------------------MA------------------L-LW	5
XP_014395552.1	------------------------------------MA------------------P-LW	5
KAB0404854.1	--------------------------MLPWV-PQALLA------------------L-LL	14
XP_007494828.1	--------------------------MPLSL-RQVLLS------------------L-LF	14
XP_003764254.1	--------------------------MPLSL-RQVLLA------------------L-LL	14
XP_020845489.1	--------------------------MPLSL-RQVLLA------------------L-LL	14
XP_027703160.1	--------------------------MPLSL-RQVLLA------------------L-LL	14
XP_017523929.1	--------------------------MLSWA-PS-LLA------------------F-LL	13
XP_008688428.1	--------------------------MLLWV-PQALLA------------------L-LL	14
XP_029812166.1	--T-----------PRHPTP--RKVTMLSWA-PRTLLA------------------L-LL	31
XP_025749781.1	--------------------------MLLWV-PQALLV------------------L-LL	14
XP_004416432.1	--------------------------MLLWV-PQALLV------------------L-LL	14
XP_027436262.1	--------------------------MLLWV-PQALLV------------------L-LL	14
XP_032284025.1	--------------------------MLLWV-PQALLA------------------L-LL	14
XP_006735421.1	--------------------------MLLWV-PEALLA------------------L-LL	14
XP_021552166.1	--------------------------MLLWV-PEALLA------------------L-LL	14
XP_026361066.1	--------------------------MLLWV-PQALLA------------------L-LL	14
XP_034523598.1	--------------------------MLLWV-PQALLA------------------L-LL	14
NP_001297113.1	--------------------------MLLWV-PRALLA------------------L-LL	14
XP_032215488.1	--------------------------MLLWV-PRALLA------------------L-LL	14
VCX31483.1	--------------------------MLLWV-PQALLA------------------L-LL	14
XP_022369003.1	--------------------------MLLWV-PQALLA------------------L-LL	14
XP_032694248.1	--------------------------MLLWV-PQALLA------------------L-LL	14
NP_001041584.1	--------------------------MLLWV-PQALLA------------------L-LL	14
XP_025862501.1	--------------------------MLLWV-PQALLA------------------L-LL	14
KAF0873564.1	--L-----------QVTPSP--ERVTMLSWA-PRVLLA------------------L-LL	156
XP_025784751.1	--------------------------MLSWV-PRVLLA------------------L-LL	14
XP_007075625.1	--L-----------QGSSRP--GKVTMLSWV-PRVLLA------------------L-LL	150
XP_030189489.1	--------------------------MLSWV-PRVLLA------------------L-LL	14
XP_019324655.1	--L-----------QGSSRP--GKVTMLSWV-PRVLLA------------------L-LL	115
XP_023094886.1	--L-----------QGSSRP--GKVTMLSWV-PRVLLA------------------L-LL	150
XP_026892357.1	--------------------------MLSWV-PRVLLA------------------L-LL	14
XP_016004457.1	--------------------------MQLWV-PHALLA------------------L-LL	14
XP_006912860.1	--------------------------MLLWV-PHALLA------------------L-LL	14
XP_011363679.1	--------------------------MLLWV-PHALLA------------------L-LL	14
XP_016076060.1	--------------------------MLQWV-LQALLA------------------L-LL	14
XP_008148371.1	--------------------------MLLWA-PQALLA------------------L-LL	14
XP_006093568.1	--------------------------MLPWA-PPALLA------------------L-LL	14
XP_006761601.1	--------------------------MLPWA-PPALLA------------------L-LL	14
XP_024426743.1	--------------------------MLLKV-PQALLA------------------L-LL	14
XP_019520779.1	--------------------------MLLWV-PQALLA------------------W-LL	14
XP_032976539.1	--------------------------MLLWV-PQMLLT------------------L-LL	14
ELW64270.1	----------------------MPPAMPQWV-RQALLA------------------L-LL	18
XP_004427367.1	--------------------------MLLWV-PQALLA------------------L-LL	14
XP_008529353.1	--------------------------MLLWV-PRALLA------------------L-LL	14
NP_001288165.1	--------------------------MLLWV-PRALLA------------------L-LL	14
XP_014711213.1	--------------------------MLLWV-PRALLA------------------L-LL	14
XP_012514621.1	--------------------------MLLWA-PQALLT------------------L-LL	14
XP_008071525.1	------------------MR--VKLAMLPWV----LLV------------------L-LL	17
XP_009005082.1	--------------------------MLLWV-QQTLLA------------------L-LL	14
XP_010332832.1	--------------------------MLLWV-QQALLA------------------L-LL	14
XP_012326058.1	------------------MR--GKLAMLLWV-QQALLA------------------L-LL	20

XP_017378796.1	--L-----------GGTPMC--GKLSMLLWV-QQALLA------------------L-LL	43
XP_032141374.1	--L-----------GGTPMR--GKLAMLLWV-QQALLA------------------L-LL	43
XP_032024018.1	--------------------------MLLWV-QQALLA------------------L-LL	14
XP_018891497.2	------------------MS--GKLTMPLWV-QQALLA------------------L-LL	20
XP_030684894.1	--------------------------MPLWV-QQALLA------------------L-LL	14
PNJ75904.1	------------------TR--GKLAMLLWV-QQALLA------------------L-LL	20
AAP35868.1	------------------ML--GKLAMLLWV-QQALLA------------------L-LL	20
XP_001149570.1	------------------MR--GKLAMLLWV-QQALLA------------------L-LL	20
XP_003805532.1	------------------MR--GKLAMLLWV-QQALLA------------------L-LL	20
XP_023063823.1	--------------------------MLLWV-QQALLA------------------L-LL	14
XP_033060210.1	--------------------------MLLWV-QQALLA------------------L-LL	14
XP_010370669.1	--------------------------MLLWV-QQALLA------------------L-LL	14
XP_017738496.1	--------------------------MLLWV-QQALLA------------------L-LL	14
XP_011782168.1	--------------------------MLLWV-QQALLA------------------L-LL	14
EHH23440.1	--------------------------MLLWV-QQALLA------------------L-LL	14
XP_003910778.3	--------------------------MLLWV-QQALLA------------------L-LL	14
XP_025212649.1	------------------MR--RKLAMLLWV-QQALLA------------------L-LL	20
XP_008019132.1	--------------------------MLLWV-QQALLA------------------L-LL	14
XP_011832421.1	--	0
XP_011759618.1	--------------------------MLLWV-QQALLA------------------L-LL	14
XP_005579749.1	--------------------------MLLWV-QQALLA------------------L-LL	14
XP_011921684.1	--	0
XP_008259391.1	--------------------------MQPWL-WQALLA------------------L-L-	13
XP_012782438.1	--------------------------MPSWF-WQALLL------------------A-LL	14
VTJ86076.1	------------------MC--GKLTMKQWI-LQALLA------------------W-LL	20
XP_015345533.1	------------------MC--GKLTMKQWI-LQALLA------------------W-LL	20
XP_027786432.1	------------------MC--GKLTMKQWI-LQALLA------------------W-LL	20
XP_026248345.1	--------------------------MKQWI-LQALLA------------------W-LL	14
XP_005378209.1	--------------------------MLLWL-QLALLA------------------L-LL	14
XP_004466010.1	--------------------------MPLSA-WRALLA------------------L-LL	14
XP_020035707.1	--------------------------MLLWV-RQVLPA------------------L-VL	14
XP_006181802.1	--------------------------MLLWV-PWALLA------------------L-LL	14
XP_006207862.1	--------------------------MLPWA-PWALLA------------------L-LL	14
XP_005891968.1	--------------------------MLPWL-PRALLG------------------L-LL	14
XP_010828662.1	--------------------------MLPWL-PRALLG------------------L-LL	14
XP_019830126.1	--------------------------MLPWL-PRALLG------------------L-LL	14
XP_017914486.1	--------------------------MLPWV-PRALLG------------------L-LL	14
XP_006060266.2	--------------------------MLPWV-PRALLG------------------L-LL	14
XP_020728447.1	--------------------------MLPWV-PRALLG------------------L-LL	14
KAF4008892.1	--------------------------MLPWV-PRALLG------------------L-LL	14
KAB0348059.1	--	0
KAB0371113.1	--	0
MBV96963.1	--------------------------MLPWV-PRALLA------------------L-LL	14
XP_007172048.1	--------------------------MLPWV-PQVLLA------------------L-LL	14
XP_007452270.1	--------------------------MLLWV-PQVLLA------------------L-FL	14
XP_023987108.1	--------------------------MLLWV-PQALLA------------------L-LL	14
XP_004273432.1	--------------------------MLLWV-PQALLA------------------L-LL	14
XP_004328646.1	--------------------------MLLWV-PQALLA------------------L-LL	14
XP_026937670.1	--------------------------MLLWV-PQALLA------------------L-LL	14
XP_030691031.1	--------------------------MLLWV-PQALLA------------------L-LL	14
XP_022414870.1	--------------------------MLLWV-PQALLA------------------L-LL	14
XP_029064651.1	--------------------------MLLWV-PQALLA------------------L-LL	14
XP_024607566.1	--------------------------MLLWV-PQALLA------------------L-LL	14
XP_032496907.1	--------------------------MLLWV-PQALLA------------------L-LL	14
XP_007535559.1	--------------------------MLLWV-PL--LA------------------L-LL	12
XP_031299536.1	--------------------------MLLWV-PWALLA------------------L-LL	14
XP_004666516.1	--------------------------MLLWV-PQALLV------------------L-LL	14
XP_008830963.1	--------------------------MLLWV-LQALLA------------------L-FL	14
XP_005347338.1	--------------------------MKLCA-PQVLLA------------------L-FL	14
XP_005069548.1	--------------------------MQLCV-PQVPLA------------------L-FL	14
XP_027267894.1	--------------------------MLLCV-PQVLLA------------------P-LL	14
OBS80992.1	---S	1
XP_006979812.1	------------------ML--GKLAMLLCM-PQVLLA------------------L-VL	20
XP_028720687.1	--------------------------MLLCM-PQVLLA------------------L-VL	14
XP_021489005.1	--------------------------MLPCI-PQVLLA------------------L-FL	14
XP_031199589.1	--H-----------RGPPTS--GKLAMLLRI-PQVLLA------------------L-FL	39
XP_028617944.1	--	0
XP_034347030.1	--------------------------MLLCI-PQVLLA------------------L-LL	14
NP_077370.2	--------------------------MPLCI-PQVLLA------------------L-FL	14
XP_032766961.1	--------------------------MPLCI-PQVLLA------------------L-FL	14
XP_021063186.1	--	0
NP_001093114.1	--------------------------MRLCI-PQVLLA------------------L-FL	14
XP_021028379.1	--------------------------MWLCI-PQVLLA------------------L-FL	14
XP_004689305.1	--------------------------MRLWG-LQALLP------------------L-LL	14
XP_004712871.1	--------------------------MLLPL-PKVLLA------------------L-LL	14
XP_004382575.1	--------------------------MSLPV-WQALLA------------------L-LL	14
XP_010596188.1	--------------------------MPLPV-QQVLLA------------------L-LL	14

XP_006890855.1	--------------------------MLLSF-QQALLV------------------L-LL	14
XP_006834038.1	--------------------------MLLPF-RQMLLV------------------L-LL	14
XP_007934716.1	--------------------------MLLLI-RQALLV------------------L-FL	14
XP_012603491.1	--------------------------MLPWA-RQALLT------------------L-LL	14
XP_012663606.1	--------------------------MLLWA-PRALLT------------------L-LL	14
XP_028372812.1	--------------------------MLLKV-PQTLLA------------------L-LL	14
XP_008589608.1	--------------------------MLLGV-WQALLA------------------L-LL	14
XP_012865077.1	--------------------------MMSWI-QQALLV------------------L-LL	14
NP_001166178.1	--------------------------MVLWL-QLALLA------------------L-LL	14
XP_004636553.1	--------------------------MSLWP-QRALLA------------------L-LL	14
XP_004856670.1	--------------------------MSLWL-QQALLA------------------L-LL	14
XP_010627744.1	--------------------------MSLWL-QQALLA------------------L-LL	14

NP_509270.1	---------KNKA---V------EKKDDR-------NERELAKHLL---------DDY--	47
VDO93178.1	--------VQN---------------------DTEATSKRLVRDLF-------ENSHY--	59
PAV91580.1	--------SNSTK---ISDPRTFNSTEDKDIAMMNIPLIRLIRDLL-------DPARY--	47
VDK46997.1	--------------------------------------MKCHQNPC-------YDFRY--	13
VIO86814.1	--------------------QSIGNQDYSDERKAANAHTRLLNQIK---------TAA--	79
VDP38785.1	--L---------P---SI--QLIFLLYNYDCQCYELSEQLFIKQLL---------SNY--	83
KOF68401.1	----------------------------TAITKLQKARLALRKHIF---------TNY--	21
CDJ96026.1	HQEVHL-------------NERRLYHALQTKHFADGTEERLYRTLL-------SEERY--	169
EFX76216.1	--------------------------------------HRQIQHL---------MANY--	11
KAF7403848.1	--------------------------MVRADGFCDEHEYRLTKYL---------LDGY--	23
KAF7427032.1	GLFL----------------RYGILAVIFKNGFCDEHEYRLTKYL---------LDGY--	38
KAE9417558.1	----------------------------MDSISGSEDEFRLIQDLR---------NNY--	21
KJH51504.1	--------------------MIVLYLILLPSLNCSPAEQRLMNDLM---------AGY--	29
VDL69795.1	--MVNQ-------------YL-LLLILGIPSVAASFRESQLYSKLL---------EEY--	33
VDO32671.1	--MVHA-------------FLALIVGAIFVGTSASLAETRLYNELL---------NGY--	34
VBB32409.1	------------------------------MITGSYHERRLYDDLM---------KNY--	19
VDN54565.1	QLIIFC-------------DIII-FMTFIYCISGSYHERRLYEDLM---------RDY--	38
EGT55171.1	--MSVC-------------ALLLTCALFAAPAFGSLQERRLYEDLM---------RNY--	34
RCN52111.1	SLVVCC-------------SLLATCIRC---TVASYHERRLYEDLM---------RDY--	35
RMX54856.1	VVP-----GKTAP---VMRID-------------DDDEQRLLKYLF---------ANY--	47
XP_022781674.1	TLN-----SLNKP---IFILGDLNC----YMLNENDLACQTLTSFY---------SSF--	310
RNA37099.1	YINRLCDK-------------NLPSYSLMDCRKVNSDEQRLLRYLM---------RNY--	54
KAA0187152.1	FISSNTEKSTTDK---KS-VLAKNKYFKRKLKLTENDEKRLLDELM---------WGY--	181
GAU96593.1	-LMFLL-----TD---SF-MLPSNISGYMRPKAEQTDEQRLLYTLL---------RGY--	52
XP_009043980.1	------------------------------------DEQKLINKLS---------ENY--	13
VDM43573.1	QLFHKL-----VA---VV-LADVTITDISDIEGAATDEQKLLYHLL---------RQY--	121
XP_003140283.1	---------------------------MHDDSNQITDEQRLLRHLL---------SQY--	22
VDN82010.1	FLITAC-----IA---NT-VGGKEI-RDDNDDNQVTDEQRLLRHLF---------GQY--	43
VDO31501.1	FLITAC-----IA---NT-VGGKEI-RDDNDDNQVTDEQRLLRHLF---------GQY--	43
VDP19591.1	---------------------MRIAEKSHDIVLSGLYEKRLLKYLFD----SSRPDAH--	33
EDO32053.1	-------------------------------------EPALLKYLL---------QNY--	12
TRY67230.1	-----------------------------RAHFQEHHERRLIDDLLPGGSDVSIARKY--	29
KAF7391312.1	-MERRLG----ED---VI-KCLWECLGVTMKSLGGQHEKRLLNNLL---------LNY--	40
GFG30449.1	IILRHFS----EP-------KIQVYPFVFAESLQGPHEKRLLHTLL---------DHY--	50
KQS30083.1	MLV-----------------YGLGLLIMIPACTAGPHEKRLLHALL---------DNY--	72
EDW57583.2	WIF-----------------LLIYLNLSAKVCLAGYHEKRLLHDLL---------DPY--	208
KNC22799.1	-------------------------------CLAGFNEKRLLHDLL---------DTY--	18
RZF44856.1	LIV-----------------LVACIASLPHESLQGPHEKRLLHNLL---------DHY--	38
ENN76856.1	QIL-----------------KFCRMLKMHEKSWAGYYEKKLLHKLL---------DTY--	44
KAF5300392.1	QVK-----------------ILHRVQEINNESSAGNNEKRLLHKLL---------DHY--	48
CAB3239999.1	CGR-------------------QLKTSFPQWARGGYHEKKLLHHLL---------DHY--	35
PCG77624.1	--	0
PZC79131.1	AGL-----------------LLLLCLLWPRGARCGYHEKRLLHHLL---------DHY--	45
KAF4083067.1	-------------------------------PGQSGPTYESLQEVFD-------------	19
XP_009924659.1	F---------------------------FLLAGAAPRYLCTYYDVIE----YLNISSYSK	42
XP_010123544.1	F--------------------------SLGTAGAAPNYVCTYYDVVE----YLNISSHSK	40
CBN81618.1	D--------------------------VLAVDGESSEQICSYQDVLD----HLNFTQNKD	38
KAF3704230.1	D--------------------------NFSADAACAQENCSYQDVLK----YLNLSMNNE	33
RUS86578.1	---------------------DPDQYKDLSYGQTYSDALTLFDTVL---------GNSSA	62
XP_034309618.1	LIIS----------------ATRVPYAMCLNNETRSPEFALQEELF---------KTY--	45
PIK58946.1	FVTF-----------------R-----CTEARYPLSNEARIRDYLL-------NQANF--	64
VUZ42516.1	IYIG-----------------N-----CLEEFKFHSIEKILIKKLL---------EKYEQ	44
PAA51166.1	NLLG----------------NGCQGSRLASVASNRSNEKLLIEELI---------SRYRE	47
TGZ55997.1	DVHG----------------RAPAG--HPKETKVKSVEKSLIRMLL---------NRYEQ	52
VDP48851.1	---MLL---------NRYEQ	8
PVD38331.1	-MFL-----------------HS---PRPHLRDVKSTEKQLVKQLL---------DRYER	30
VDK73355.1	LFAF-----------------L-----SLSMVHCNEDAKRLHDDLM---------ANY--	40
VDD84963.1	TIFS-----------------Q-----SPTKTLANEDAKRLYDDLM---------MSY--	38
VDK42196.1	--	0
KFD58288.1	FSLF-----------------T-----DCVQCSRNNNARRLFQDIL---------TGY--	51
OUC49089.1	FMIT-----------------L-----YVVRCSRSEHARRLFQDIL---------TGY--	63
EYC26492.1	AWAV-----------------A-----MYAISHANRDANRLFEDLL---------ADY--	66
VDK27218.1	FW-------------------------LLPLSQCSRDASRLFEDLL---------ADY--	36

```
VDM17286.1        ------------------------------------------------------------     0
VDL91846.1        ---------------------------------------------F---------TRF--     16
KAE9548540.1      TIFL-----------------YSTNEIRSKVISHKSYEEALYEELM---------FDY--     39
PDM74087.1        TAFI-----------------Y-----FTGPIGCSEEEERLMVDIF---------RGY--     38
XP_024504322.1    --FF-----------------F-----IPKNLNGSEDEERLMQDIF---------KGY--     39
PIO52336.1        AIAS-----------------L-----LMAPALASDDEERLMMDVF---------RGY--     35
TKR73865.1        LCIA-----------------L-----QVGQNEASDYEERLMVEVF---------RGY--     40
RLU23395.1        FLIS-----------------A-----ILCVGLCSEDEERLVRDLF---------RGY--     37
XP_002427906.1    FVVV-----------------V-----IVVVSRCSEDEERLVRDLF---------RGY--     44
TMW47392.1        LLLV-----------------L-----GLCSVNASEDEERLVRDLF---------RGY--     42
VDD83956.1        PL--------------------------FLELLPSGSDWKLYKYLT-------TKKNY--     37
VEL33078.1        ------------------------------------------------------------     0
XP_009019088.1    LILW-----------------I-----SFNVTRSNPYAKKLYDQLI-------RKKGY--     41
KAE9536378.1      PLLL-----------------L-----SAVGCLCNPDAKRLYDDLL---------SNY--     70
KDR23473.1        --MG-----------------F-----IVTPSQANPDAKRLYDDLL---------SNY--     25
KAF7266914.1      CYVS-----------------A-----EVKQIEANPDAKRLYDDLL---------SNY--     58
KAF4519445.1      LLPG-----------------P-----GMRTVEANPDAKRLYDDLL---------SNY--     70
OXU25983.1        DGFH-----------------P-----EINSFEANPDTKRLYDDLL---------SNY--     43
TGZ32403.1        SIVY-----------------G-----NRVVFEANPDTKRLYDDLL---------SNY--     40
ELT94491.1        WQFV-----------------L-----LLTPCDASPQAKELYDDLL-------RRSDY--     40
VVC39575.1        CIML-----------------V-----IISGCSMNPDAKRLYDDLL---------SNY--     134
RVE49089.1        LLFA-----------------L-----LLAGCGGNPDAKRLYDDLL---------SNY--     38
KAB0800277.1      MLGG-----------------F-----GLPAVFGNPDAKRLYDDLL---------SNY--     35
TDG52197.1        FIAL-----------------H-----FATGGLANPDAKRLYDDLL---------SNY--     36
TMW48669.1        FIAL-----------------H-----FVTGGLANPDAKRLYDDLL---------SNY--     36
XP_025896085.1    ----------QH----------PGAPRDS-GAHGEPVLLRLAEQLL---------ARY--     47
KAF2977017.1      ------------------------------------------------------------     0
XP_009321837.1    -----------------MGNPNRYAARGP-ETRDRPALRRLSHYLL---------AHY--     31
XP_009979985.1    ----------------------TQKGGQM-PEPTEPALRRLSRYLL---------AHY--     26
XP_028942374.1    ----------------------------T-QRGAEPALHRLSHYLL---------SHY--     20
PKK19633.1        ---LALGGFVSHPI-PPSAGTGPRGRRRP-PEPAQPALRRLSRYLL---------AHY--     89
XP_009894240.1    PGRSPSVGFSD-------AGGTPVSGGGA-GGPAAPPLPRLSHCLL---------AHY--     143
XP_032820219.1    ---------AR----NPSC----FAAHST-NAHKKTPLRNLSSRLS---------SGY--     76
XP_029429480.1    LPPEHKSSVKQ----TGQST--GEEIGGT-ANTTEPTFIRLYKHLM---------KDY--     85
XP_030077572.1    ----------M----RPWTT--DEEIYDS-ANTINSTYIQLFNHLM---------TGY--     32
XP_033774596.1    --HITCGTLHE----AQGAT--GEEICDP-ANTNESTYIQLFNHLM---------TGY--     63
ETE72600.1        ---------------S------QKIPNKA-SGSSKAALRQLSDYLF---------ANY--     59
XP_018082638.1    PSKTFCGRLDR----RSHNINFYANCADV-NKSPKSALLQLSDHLM---------EGY--     259
XP_032905410.1    --MSFHISWAV----VPIVLNALLSSGVQ-TKGNHSTTLELSNYLM---------ANY--     42
XP_020773490.1    ATGR---------------AGKRAGAGSA-GRFANATLVRLSEYLS---------SGY--     51
XP_033833634.1    QHGH-----EDCTHLHGAAFSSTVWSNGT-DWFANATLVRLSEYLS---------SGY--     77
XP_030215795.1    QGVL-----PASP--------VKKIGNSM-GRFANATLVRLSEFLS---------AGY--     50
XP_030602980.1    STAT-----RGNA-A-------KKLGGGT-GRFANATLVRLSEFLS---------TGY--     56
XP_004573543.1    NEAS-----KECT-A-------KKLGSST-GRFANATLVRLAEFLS---------VGY--     50
XP_005916159.1    NEAS-----KECT-A-------KKLGSST-GRFANATLVRLAEFLS---------VGY--     50
XP_013132089.1    NEAS-----KECT-A-------KKPGSST-GRFANATLVRLAEFLS---------AGY--     50
XP_031603488.1    SAAT-----RENT-A-------KKPGSST-GRFANATLVRLAEFLS---------AGY--     56
XP_005725536.1    SAAT-----RENT-A-------KKLGSST-GRFANATLVRLAEFLS---------VGY--     56
XP_026038075.1    SAAT-----RENT-A-------KKLGSST-GRFANATLVRLAEFLS---------VGY--     56
CAF96649.1        -------------------------------------LVRLSEFLS---------AGY--     12
XP_023818570.1    QQPW-----ESVC-AKKLGSSSSSSSSSS-GRFANATLVRLSEFLS---------SGY--     46
RVE64289.1        HGGF-----RASA-AKK-----L-GSSSS-GRFANATLVRLSEFLS---------SGY--     81
XP_024121971.1    HGGF-----RAST-AKK-----LAGGGSS-GRFANATLVRLSEFLS---------SGY--     52
XP_015817559.1    PVNV-----LQPS-VKK-------LGSNS-GRFANATLVRLAEFLS---------AGY--     36
XP_015225670.1    HGAA-----VFCT-VKK-------PSSHP-GRFANATLVRLAEFLS---------TGY--     50
XP_012722063.1    HGAA-----TVCT-VKK-------LGGNP-GRFANATLVRLSEFLS---------AGY--     50
XP_032431307.1    HGAA-----TVCT-VKK-------PPSNP-GRFANATLVQLADFLS---------AGY--     50
XP_014328329.1    HGAA-----TVCT-VKK-------PPSNP-GRFANATLVRLADFLS---------AGY--     50
XP_027886578.1    HGAA-----TVCT-VKK-------PPSSP-GRFANATLVRLADFLS---------AGY--     50
XP_008426791.1    HGAA-----TFCT-VKK-------PPSNP-GRFANATLVRLADFLS---------AGY--     50
XP_014838686.1    HGAA-----TFCT-VKK-------PPSNP-GRFANATLVRLADFLS---------AGY--     50
XP_014885967.1    HGAA-----TFCT-VKK-------PPSNP-GRFANATLVRLADFLS---------TGY--     50
XP_033954312.1    QGAT-----RACT-V-------KKSGSST-GRFANATLVRLSEFLS---------AGY--     50
XP_034089244.1    QGAT-----RACT-V-------KKSGSST-GRFANATLVRLSEFLS---------AGY--     50
XP_010780064.1    HESL-----ISVD-L-------KKPGSST-GRFANATLVRLSEFLS---------AGY--     66
XP_033970261.1    HESL-----ISVD-L-------KKPGSST-GRFANATLVRLSEFLS---------AGY--     66
XP_029375575.1    ----------KIP-G-------KKVSSST-GRFANATLVRLSEFLS---------GGY--     87
KAF0023022.1      PRRE-----EENA-L-------KRSGGSA-GRFSNATLVRLSEFLS---------AGY--     83
XP_019952614.1    EVSS-----LSRD-V-------KRQGSST-GRFANATLVRLSEFLS---------AGY--     39
XP_034463117.1    QGAA-----RACT-V-------KRQGSST-GRFANATLVRLSEFLS---------GGY--     50
XP_029924656.1    QEAV-----RACT-V-------RKLGSSS-GRFSNATLVRLAEFLS---------AGY--     51
XP_028323228.1    HGAT-----KACT-V-------KKLGSST-GRFANATLVRLSEFLS---------AGY--     50
TNM84480.1        LAAT-----RGCT-V-------KKPSSSS-GRFSNATLVRLSDFLS---------AGY--     50
XP_029703788.1    LAAT-----RGCT-V-------KKSSSSS-GRFSNATLVRLSDFLS---------AGY--     50
XP_030010368.1    QGAS-----RACT-V-------KKLGSST-GRFANATLVRLSEFLS---------AGY--     53
XP_019725056.1    QGTA-----RSCT-V-------RKLGSST-GRFANATLVRLSEFLS---------AGY--     51
XP_034034934.1    LMSV-----LHPV-V-------KKINSST-GRFANATLVRLSEFLS---------AGY--     39
```

```
XP_029956880.1      DGAS-----RACT-V-------RRSGSSA-GRFANATLVRLSEFLS---------AGY--      50
XP_008331307.3      QGAA-----PICT-A-------KRLGSST-GRFANATLVRLSEFLS---------AGY--      83
XP_031724845.1      STVG-----EENT-V-------KKLGSSP-GRFANATLVRLSEFLN---------AGY--      51
XP_013859395.1      HGAS-----TACT-V-------KKVGSST-GRFANATLVRLSEFLS---------AGY--      50
XP_017275335.1      HGAS-----TACT-V-------KKLGSST-GRFANATLVRLSEFLS---------AGY--      50
XP_020496197.1      QGAT-----RACT-V-------KKVGGSP-GRFANATLVRLSEFLS---------AGY--      50
XP_029030400.1      PGAS-----RACT-V-------KKAGGST-GRFANATLVRLSEFLS---------AGY--      50
XP_022060538.1      QGAS-----SACT-V-------KKASSSS-GRFANATLVRLSEFLS---------AGY--      51
XP_023150584.1      QGAS-----SACT-V-------KKLGSST-GRFANATLVRLSEFLS---------AGY--      51
XP_029303892.1      TLIS-----LDST-L-------KKPGSSP-GRFANATLVRLSEFLS---------AGY--      74
XP_020454172.1      QGTL-----RACT-V-------KKLGSST-GRFANATLVRLSDFLS---------AGY--      49
TKS83244.1          STAG-----EENT-V-------KKLGSST-GRFANATLVRLSEFLS---------AGY--      52
XP_022616583.1      DEVL-----IVDV-V-------KKLGSST-GRFANATLVRLSDFLS---------AGY--      83
XP_030293172.1      QGAS-----RACT-V-------KKPGGSS-GRFANATLVRLSEFLS---------GGY--      50
XP_027129539.1      QGAS-----RACT-V-------KKLGSST-GRFANATLVRLSEFLS---------AGY--      50
XP_034539538.1      QRGS-----RACT-V-------KKPGSSA-GRFGNATLVRLSEFLS---------AGY--      50
XP_008277330.1      HGAA-----RACT-A-------KKAGGST-GRFANATLVRLSDFLS---------GGY--      50
XP_028276940.1      YGAS-----RACT-V-------KKLGSNT-GRFGNATLVRLSEFLS---------AGY--      50
XP_023263433.1      QGAS-----NACT-V-------KKLGSST-GRFANATLVRLSDFLS---------AGY--      50
XP_034406336.1      QGAS-----RACT-V-------KKLGGSS-GRFANATLVRLSEFLS---------AGY--      50
XP_026184460.1      QGAS-----RACT-V-------KKLSSST-GRFANATLVRLSEFLS---------AGY--      50
KAF1378228.1        QGAS-----RACT-V-------KKLGSST-GRFANATLVRLSEFLS---------AGY--      50
XP_028450365.1      QGAS-----RACT-V-------KKLGSST-GRFANATLVRLSEFLS---------AGY--      50
XP_031163851.1      QGAS-----RACT-V-------KKLGSST-GRFANATLVRLSEFLS---------AGY--      50
XP_032389084.1      QGAS-----KACT-V-------KKLGSST-GRFANATLVRLSEFLS---------AGY--      50
XP_033494682.1      QGAS-----RACT-V-------KKLGSST-GRFANATLVRLSEFLS---------AGY--      50
XP_018544782.1      QGAS-----RACT-V-------KKLGTST-GRFANATLVRLSEFLS---------AGY--      50
XP_026228189.1      QGAS-----RACT-V-------KKLGSST-GRFANATLVRLSEFLS---------AGY--      50
XP_028976606.1      QGTA-------------QACTVKKLGNNT-GRFANATLVHLSEYLS---------AGY--      50
CAB1352378.1        QGTA-------------RACTVKKLSNNT-GRFANATLVRLSEYLS---------AGY--      50
XP_023866849.1      QGTA-------------RACTVKKLSNNT-GRFANATLVRLSEYLS---------AGY--      50
XP_013992832.1      QATS-------------RACTVKKLSNNT-GRFANATLVRLSEYLS---------AGY--      50
XP_029546688.1      PGTA-------------RACTVKKLSNNT-GRFANATLVRLSEYLS---------AGY--      50
XP_021481546.1      QGTA-------------RACTVKKLSNNT-GRFANATLVRLSEYLS---------AGY--      50
XP_020339889.1      QGTA-------------RACTVKKLSNNT-GRFANATLVRLSEYLS---------AGY--      50
XP_029481972.1      QGTA-------------RACTVKKLSNNT-GRFANATLVRLSEYLS---------AGY--      50
KPP68743.1          ---------------------MTRTEHSP-GRFSNATLVRLSEYLS---------AGY--      27
XP_023669383.1      YGTQ-------------QVRPVRRTGNGS-GRFSNATLVRLSEYLS---------AGY--      49
KAA0720096.1        ------------------------------------------------------------      0
XP_009293684.1      YGTV-------------VDCSVKKLGNNT-GRFANATLVRLNEFLS---------AGY--      49
XP_026090784.1      YGAV-------------ETCSVKKLSNNT-GRFANATLVRLNEFLS---------AGY--      49
XP_018918715.1      YGAV-------------VTCSVKKLSNNT-GRFANATLVRLNEFLS---------AGY--      49
KAF4098549.1        CGAV-------------VTCSVKKLGNNT-GRFANATLVRLNEFLS---------AGY--      49
XP_016084173.1      YVAV-------------VVCSVKKLSNNT-GRFANATLVRLNEFLS---------AGY--      49
XP_016332759.1      YVAV-------------VACSVKKLSNNT-GRFANATLVRLNEFLS---------AGY--      49
XP_016398039.1      YVAV-------------VACSVKKLSNNT-GRFANATLVRLNEFLS---------AGY--      49
XP_026989970.1      HIAV-------------TACSVKRQGSNP-GRFANATLVRLSEHLS---------TGY--      49
TSQ12698.1          HIAV-------------TACSVKRQGSNP-SRFANATLVRLSEYLS---------AGY--      49
XP_017347546.1      NIAV-------------TACSVKRLGSNP-GRFANATLVRLSEYLS---------AGY--      44
XP_026794616.2      HISV-------------TACSVKRLGSNP-WRFANATLVRLSEYLS---------AGY--      49
XP_026861889.1      CGAV-------------KACSVKKLGSNT-GRFANATLVRLSEFLS---------AGY--      49
XP_017575347.1      HGAV-------------RACSVKKLGSNT-GRFANVTLVRLSEFLS---------AGY--      49
XP_022531596.1      YATV-------------QTCSVKKLGSNT-GRFANATLVRLSEFLS---------AGY--      49
XP_012691019.2      ---M-------------ACGRVKKIGNNP-GRFANATLVRLSEYLS---------TGY--      32
XP_028839723.1      DTSG-------------CTCSVKKIGNNT-GRFANATLVRISEYLS---------AGY--      49
XP_030638860.1      -----------------MSALVRKLGTST-GRFANATLVRLSEFLS---------AGY--      31
XP_007882964.1      -----------------------KKGDKR-SPNTRPALLRLHEYLM---------ANY--      25
GCB70244.1          ----------------------MKKSGNQ-TAGPTPGLLQLSDYLM---------ANY--      26
GCC26242.1          TALL-----LS----------EGWRSGNQ-TAGSRPSLLQLSNYLM---------TNY--      47
XP_020387034.1      DDYQ-----QA----------SGQRSGNQ-TESPRPGLLQLSDYLM---------TNY--      38
XP_028931940.1      LLAS-----LGSSQ--GSGSPGLWRHLQA-RNATRSALLELSDYLL---------THY--      55
XP_031762490.1      ---------------------LIPCYANV-NRSSKPTLLRLSDHLM---------EGY--      44
PIO32240.1          ------------------------------------------------------------      0
XP_018425466.1      ---------------------MISAVCGI-ENVTKPVLIRLSDELM---------EGY--      45
XP_006642351.1      LCAQ-----SAR-------S-GRRIGNNP-GRFANATLVRLSDYLM---------TGY--      66
XP_028665372.1      FNTT-----RSS-------E-AFRLGNNP-NRFANSTLVRLSDYLM---------TGY--      49
GCF49814.1          LGWG-----WVG-------DASPKVANRT-ARSSKATFHLLYDYLL---------SNY--      43
XP_015277816.1      FTCV-----LPS-------KASQKVANRT-ASSSQATFHQLYDYLL---------TSY--      51
XP_005987340.2      ---M-----GEL-------EPARKPGNQT-SKMTKPTLFRLSEYLM---------THY--      33
XP_019339534.1      AAFE-----LQS-------EAARRGKNRT-LDTAKPALLQLSEHLL---------THY--      50
XP_025063414.1      AAFE-----LQS-------EAARRGKNRT-LDTAKPALLQLSEHLL---------THY--      50
XP_019367488.1      AAFE-----LQS-------EAVRRGKNRT-LDTTKPALLQLSEHLL---------THY--      50
XP_019412078.1      AAFE-----LQS-------EAARRGKNKT-MGTAKPALLQLSEHLL---------THY--      50
XP_014434314.1      FTFK-----FQS-------EASRKGNKTT-WNGTKPALLQLSDYLL---------THY--      50
XP_007060556.1      KSHY-----TIS-------ETARKGNNRT-LNSAKPALLQLSDYLL---------THY--      44
XP_024072175.1      -------------------MAARKGNNRT-LNSAKPALLQLSDYLL---------THY--      29
XP_008170567.1      FTVM-----FQS-------EAARKGNNRT-LNSAKPALLQLSDYLL---------THY--      50
```

```
XP_034610543.1      FTVM-----FQS-------EAARKGNNRT-LNSAKPALLQLSDYLL---------THY--      50
XP_030394127.1      FTFR-----FQS-------EAARKGNNRT-LNSAKPALLQLSDYLL---------THY--      50
XP_032651960.1      FTFR-----FQS-------EAARKGNNRT-LNSAKPALLQLSDYLL---------THY--      50
XP_025020083.1      FASR-----VQS-------KVSQKTPSKA-SSSNKAALRQLSDYLF---------ASY--      50
XP_026536833.1      VAST-----VPS-------KVSQKTPTKA-SGSSKAALRQLSDYLF---------ASY--      50
XP_026564787.1      VAST-----VPS-------KVSQKTPTKA-SGSSKAALRQLSDYLF---------ASY--      50
XP_032084675.1      -MPM-----VHS-------KVSQKIPNKA-LGSSKAALRQLSDYLF---------ANY--      35
XP_029139341.1      VASM-----VHS-------KVSQKIPSKA-SSSSKAALRQLSDYLF---------ANY--      50
XP_034281006.1      VAST-----VHC-------KVSQKIPNKA-SSSSKAALRQLSDYLF---------ANY--      50
XP_020649062.1      FAAL-----LES-------KAYQKISNKA-INPTKPALRHLSDYLL---------ANY--      50
XP_008117087.1      FAPR-----LQS-------KAPPKIPSKI-TNVSKPALRMLSDYLF---------TGY--      50
XP_028564324.1      FTSV-----LQC-------KAYHKTASKQ-ANLTKPALRLLSDYLF---------SNY--      56
XP_033028155.1      FTSV-----LQC-------KAFHKTASKP-ENLTKPALRLLSDYLF---------SNY--      55
XP_025963249.1      -----------MTKAFSFSLSAPQAGSGM-PAPGKPTLLRLAHHLL---------AHY--      37
XP_009668348.1      APFT-----L-LLLTAVLQNEATLAGSRT-PGPSKPALLRLSDYLL---------AHY--      49
XP_013798935.1      ---------------MGAAKMATHAGSRT-SGPGKPTLLRLSDHLL---------AHY--      33
XP_025913685.1      KPNG-----WGKANKKGWLLSSTHAGSRT-SGPGKPTLLRLSDHLL---------AHY--      58
XP_013042552.1      LASM-----LQ-DTAPFPALAGTPRASWV-PDSDAPALLRLSDSLL---------AHY--      56
XP_005030458.2      LVSM-----LQ-DT-------GTPKASGV-PDPDTPALLRLSDSLL---------AHY--      49
XP_032057953.1      ---M-----LQ-DT-------GTPKASWV-PDPDTPALLRLSDSLL---------AHY--      32
XP_021232050.1      PVSM-----LQ-DA------AGALRGSQE-PDPTTPALLRLSDTLL---------AHY--      51
OXB62403.1          PVLM-----LQ-D-------TGVLQGRQE-PDPTTPALLRLSDTLL---------AHY--      50
OXB81319.1          ---M-----LQ-D-------TGVLQGRQE-PDPTTPALLRLSDTLL---------AHY--      32
XP_010722007.1      PVSM-----LQ-D-------AGALQGSPV-PDPSTPALLRLSDTLL---------AHY--      51
XP_015739349.1      PVWM-----LQ-D-------AGALQGSTE-PDPSTPALLRLSDTLL---------THY--      51
XP_031455498.1      PVSM-----LQ-DA------VGALQGSPE-PDTSTPALLRLSDTLL---------AHY--      52
POI27435.1          --------------------GALRGSPE-ADPSTPALLRLSDTLL---------AHY--      27
XP_004948120.1      PVSM-----LQ-DA------VGALQGNPE-PDPSTPALLRLSDTLL---------AHY--      52
XP_032851190.1      LTST-----LQ-SQ------AG---THRT-PEPAEPALRRLAHYLL---------AHY--      47
XP_010007255.1      LAGT-----LQ-NS------GT-RGGSQT-PEPAEPALRRLSHHLL---------AHY--      49
XP_030320702.1      ----------------------------M-LEPTEPALRRLSHHLL---------AHY--      20
XP_010191940.1      LSST-----LQ-SP------GT-HRRGRM-LEPTEPALRRLSHSLL---------ERY--      47
XP_027737112.1      QPPT-----LP-LL------AGTQRGGRT-PEPSEPALRRLSHYLL---------AHY--      45
XP_027555032.1      QPPS-----LP-LL------AGTQRGGRT-PEPSEPALHRLSHYLL---------AHY--      50
XP_032565370.1      PLAL-----ML-QS------PGTQRGGRT-PEPSEPALRRLSHYLL---------AHY--      96
XP_027511217.1      WPPS-----LP-LL------AGTQRGGRT-PEPSEPALRRLSHYLL---------AHY--      195
XP_027593499.1      KPPS-----LP-LL------AGTQRGGRT-PEPSEPALRRLSHYLL---------AHY--      146
XP_017664924.1      LPPS-----LP-LL------AGTQRGGRT-PEPSEPALRRLSHYLL---------AHY--      47
XP_029817938.1      -------------M------AGTQRGGRT-PEPSEPALRRLSHYLL---------AHY--      29
XP_005058641.1      LASA-----LQ-SP------GAQ----------HEPALRRLAQHLL---------ARY--      80
XP_021385804.1      LAST-----LQ-SP------GA-QRGGRV-PEPSEPALRRLSHHLL---------AHY--      49
XP_030146687.2      LAST-----LQ-SP------GA-QRGGRV-PEPSEPALRRLSHYLL---------AHY--      94
KAF4796420.1        LAST-----LQ-SP------GT-QHGGRV-LEPSEPALRRLAHHLL---------AHY--      49
XP_032937581.1      LAST-----LQ-SP------GT-QHGGRV-LEPSEPALRRLAHHLL---------AHY--      96
XP_031989659.1      LAST-----LQ-SP------AGTQHRGRM-PEPSEPTLRRLSHYLL---------AHY--      50
XP_010402086.1      LAST-----LQ-SP-------GTQHRGRM-PEPSEPTLRRLSHYLL---------AHY--      49
XP_017594069.1      ----------------------------M-PEPSEPTLRRLSHYLL---------AHY--      20
XP_014115268.1      PPSH-----KV-SH------KSPLRARRT-REPSEPALQRLAHYLL---------AHY--      74
XP_023797108.1      ----------------------------M-PEPSEPALQRLAHYLL---------AHY--      20
XP_033375614.1      ----------------------------M-PEPSEPALQRLAHHLL---------AHY--      20
XP_014740121.1      LAAP-----LH-PD------KSSQRGGRV-PEPSEPALRRLCHHLL---------AHY--      194
RLV83430.1          LDGK-----PQ-MV------G-TQHGARV-PEPSEPALRRLSHHLL---------ARY--      40
XP_009096098.2      LAAT-----LQ-SP------GPG-RGGGM-PEPSEPALRRLSHHLL---------AHY--      155
TRZ15870.1          LAST-----LQ-SP------ADAQHGGWM-PEPPEPALRRLSHSLL---------AHY--      50
RMB91935.1          ----------------------------M-PEPSEPALRRLSHDLL---------ARY--      20
XP_030820843.1      LAAT-----LQ-SP------GTR-PGGGT-PEPSEPALRRLSHHLL---------AHY--      96
XP_014165179.1      FANS-----PD-VQ------STR-PGGGT-PEPSEPALRRLSHHLL---------AHY--      98
XP_026653582.1      ------------------------------------------------------------      0
PKU35975.1          LARA-----PQ-SQ------GS-RHGGQT-PE---SALRRLSHSLL---------AHY--      46
XP_014805072.1      LAWA-----PQ-RQ------GS-WRGGQT-PE---PALRRLSHSLL---------VHY--      46
XP_009818330.1      ------------------------------------------------------------      0
OPJ68307.1          LAAP-----LQ-ST------GS-RRGGRM-LEPAQPALRRLSRYLL---------AHY--      49
XP_008936289.1      LAVM-----LQ-SQ------GT-WRGDWT-PEPAEPALRRLSHYLL---------SHY--      50
XP_010287046.1      ------------------------------------------------------------      0
XP_005240140.2      LIST-----LQ-SQ------GT-WQGGQT-PEPAEPALRRLSHYLL---------SHY--      109
XP_005437752.2      LIST-----LQ-SQ------GT-WQGGQT-PEPAEPALRRLSHYLL---------SHY--      109
KFV74811.1          -----------------------------------PALPWLSHYLL---------AHY--      14
XP_010018389.1      ATVL-----VS-VQ------GT-RQQGRT-PEPTEPALRRLSHYLL---------SHY--      57
KQK78711.1          LPSM-----LQ-SQ------GT-RRQGWT-PEPMEPALRRLSHYLL---------AHY--      49
XP_009570162.1      LALT-----LQ-SQ------GT-QQRGQK-PDPAEPALRRLSHYLL---------SHY--      48
KFP11268.1          ------------------------------PAPPAPALRRLAHYLL---------ARY--      19
KFQ98910.1          --PL-----SL-PA------GT-RQGGRP-PEPDEPALRRLSHYLL---------ARY--      33
XP_012985202.3      LTST-----LQ-SQ------GT-WRQGKT-PDPMEPALHRLSHYLL---------THY--      49
XP_010573388.1      LALM-----LQ-SQ------GT-RRGGWT-PEPTEPALHRLSHYLL---------AHY--      48
XP_029879496.1      ----------------------------M-PEPTEPALHRLSHYLL---------AHY--      20
XP_030361086.1      LTST-----LQ-SQ------GT-WQQGRT-PEPTEPALHRLSHYLL---------AHY--      101
KFM00668.1          --PF-----PL-PA------GM-RHGGRT-PETAETALRRLSHYLL---------AHY--      33
```

```
KAF1479074.1        -------------------------------------LRRLSHYLL---------AHY--     12
KAF1651161.1        ------------------------------------ALRRLSHYLL---------AHY--     13
KAF1673648.1        ------------------------------------ALRRLSHYLL---------AHY--     13
KAF1493319.1        ------------------------------------ALRRLSHYLL---------AHY--     13
KAF1584157.1        ------------------------------------ALRRLSHYLL---------AHY--     13
KAF1571723.1        ------------------------------------ALRRLSHYLL---------AHY--     13
KAF1533169.1        ------------------------------------ALRRLSHYLL---------AHY--     13
KAF1638955.1        ------------------------------------ALRRLSHYLL---------AHY--     13
KAF1549972.1        ------------------------------------ALRRLSHYLL---------AHY--     13
KAF1606914.1        ------------------------------------ALRRLSHYLL---------AHY--     13
KAF1510957.1        ------------------------------------ALRRLSHYLL---------AHY--     13
KAF1498899.1        ------------------------------------ALRRLSHYLL---------AHY--     13
KAF1411525.1        ------------------------------------ALRRLSHYLL---------AHY--     13
KAF1429205.1        ------------------------------------ALRRLSHYLL---------AHY--     13
XP_005334318.1      PCIL-----V-------AATGIL--ATDTP-HPRNSTLHHLTQYLL---------EQY--     39
XP_014395552.1      PFIL-----V-------AASGIL--AIDTP-HPRNSALYHLTKQLL---------QRY--     39
KAB0404854.1        PTFL-----A-------QGEAKHRRGPQAR-DASRPALLRLSDHLL---------ANY--     50
XP_007494828.1      LIFL-----G-------PSEAKRKQSPKVL-NNTKPALLQLSDYLL---------THY--     50
XP_003764254.1      LIVL-----G-------PSEARKKQGTKAP-NNTKPALLQLSDYLL---------THY--     50
XP_020845489.1      LIFL-----G-------PCEGRRKQGTKAA-NNTKPALLQLSDHLL---------THY--     50
XP_027703160.1      LIFL-----G-------PCEGRRKQGIKAA-NNTKPALLQLSDHLL---------THY--     50
XP_017523929.1      PTLL-----V-------QGEARHHGGLW-T-HSTRPALLRLSDYLL---------AGY--     48
XP_008688428.1      PMLL-----A-------QGEARRWRPLQAQ-NASRPALRRLSDYLM---------ANY--     50
XP_029812166.1      PALL-----A-------PGQARLREHPEAQ-NASRPALLRLSEYLL---------ANY--     67
XP_025749781.1      PTLL-----A-------QGEARRWRHRQSQ-NASRPALLRLSDHLL---------ANY--     50
XP_004416432.1      PTLL-----A-------QGEARRWRHRQSQ-NASRPALLRLSDHLL---------ANY--     50
XP_027436262.1      PTLL-----A-------QGEARRWRHRQPQ-NASRPALLRLSDHLL---------ANY--     50
XP_032284025.1      PTLL-----A-------QGEARRWRHHQAQ-KAARPALLRLSDHLL---------ANY--     50
XP_006735421.1      PTLL-----A-------QGEARRWRHHQAQ-KASRPALLRLSDHLL---------ANY--     50
XP_021552166.1      PTLL-----A-------QGEARRWRHHQAQ-EASRPALLRLSDHLL---------ANY--     50
XP_026361066.1      PMLL-----A-------QGEARRWRPLQAQ-NASRPALRRLSDYLM---------ANY--     50
XP_034523598.1      PTLL-----A-------QGEARRWRPLQAQ-NASRPALRRLSDYLM---------ANY--     50
NP_001297113.1      PMLL-----A-------QGEAKHWRPLQAQ-NSSRPALLRLSEHLM---------ANY--     50
XP_032215488.1      PMLL-----A-------QGEAKHWRPLQAQ-NSSRPALLRLSEHLM---------ANY--     50
VCX31483.1          PTLL-----T-------QGEARNWRPLQAQ-NTSRPALLRLSDHLM---------ANY--     50
XP_022369003.1      PTLL-----A-------QGEARHWRRLQAQ-NTSRPALLRLSDHLM---------ANY--     50
XP_032694248.1      PTLL-----A-------QGEARHWRPLQAQ-NTSRPALLRLSDHLM---------ANY--     50
NP_001041584.1      PTLL-----V-------QGEARHWRHLQAQ-NTSRPALLRLSNYLL---------ANY--     50
XP_025862501.1      PTLL-----V-------QGEARHWRHLQAQ-NTSRPALLRLSNYLL---------ANY--     50
KAF0873564.1        PAFL-----T-------QGQARLREHPGAQ-NASRPALLRLSDHLL---------ANY--     192
XP_025784751.1      PTLL-----A-------HGQARRREHPQAQ-NTSRPALLRLSDYLL---------ANY--     50
XP_007075625.1      PTLL-----A-------HGQARRREHPQAQ-NTSRPALLRLSDYLL---------ANY--     186
XP_030189489.1      PPLL-----T-------HGQARRREHPQAQ-NTSRPALLRLSDYLL---------ANY--     50
XP_019324655.1      PTLL-----A-------HGQARRREHPQAQ-NTSRPALLRLSDYLL---------ANY--     151
XP_023094886.1      PTLL-----A-------HGQARRREHPQAQ-NTSRPALLRLSDYLL---------ANY--     186
XP_026892357.1      PTLL-----A-------HGQARRREHPQAQ-NTSRPALLRLSDYLL---------ANY--     50
XP_016004457.1      PTFL-----T-------QGEG-----LQAH-NLSEPALGRLSDYLL---------RNY--     45
XP_006912860.1      PTLL-----T-------QGEG-----LQAH-NLSDPALGRLSDYLL---------SNY--     45
XP_011363679.1      PTLL-----T-------QGEG-----LQAH-NLSDPALGRLSDYLL---------SNY--     45
XP_016076060.1      PMFL-----T-------QGEARRRGGPQAH-NTSGPALRRLSDHLL---------TNY--     50
XP_008148371.1      PGLL-----T-------QGEARRRAGLQAH-NTSRPALWRLSDHLL---------TNY--     50
XP_006093568.1      PVLL-----T-------QGEARRRGGLRAQ-NTSGPALRQLSDHLL---------TNY--     50
XP_006761601.1      PVLL-----T-------QGDARRRGGLPAQ-NTSGPALRRLSDHLL---------ANY--     50
XP_024426743.1      PTLL-----T-------QGEGK-----AAH-NTSRPTLHRLSDHLL---------TNY--     45
XP_019520779.1      PTLL-----T-------QGEASHRGSFQAH-NTSRPALGRLSDYLL---------TNY--     50
XP_032976539.1      GMLL-----T-------QGEARHSGGLQAH-NTSRPALGRLSDYLL---------TNY--     50
ELW64270.1          PTLL-----A-------QGEARRRGDPQAR-NSSRPALIRLSDYLL---------ANY--     54
XP_004427367.1      PTLL-----A-------QGEARQRGGPQAR-NTSRPALLRLSDYLL---------ANY--     50
XP_008529353.1      PMLL-----A-------QGEARLRGVPHTH-NTSRPALLRLSDYLL---------ANY--     50
NP_001288165.1      PMLL-----A-------QGEARLRGVPHTH-NTSRPALLRLSDYLL---------ANY--     50
XP_014711213.1      PMLL-----A-------QGEARLRGVPHTH-NTSRPALLRLSDYLL---------ANY--     50
XP_012514621.1      PTLL-----A-------QGEARRRGGLQGR-NTSRPALLRLSDHLL---------ANY--     50
XP_008071525.1      PTFL-----A-------QGE-----GKRGR-NTTRPALLRLSDYLL---------ANY--     48
XP_009005082.1      PTFL-----A-------QGEARRNRGLQGR-NTTKPALLRLSDYLL---------TNY--     50
XP_010332832.1      PTFL-----A-------QGEGK-----RGR-NTTKPALLRLSDYLL---------TNY--     45
XP_012326058.1      PMFL-----A-------QGEARRSGGLQGR-NTTKPALLRLSDYLL---------TNY--     56
XP_017378796.1      PTFL-----A-------QGEARRSGGLQGR-NTTKPALLRLSDYLL---------TNY--     79
XP_032141374.1      PTFL-----A-------QGEARRSGGLQGR-NTTKPALLRLSDYLL---------TNY--     79
XP_032024018.1      PTLL-----A-------QGEARRS-----R-NTTRPALLRLSDYLL---------TNY--     45
XP_018891497.2      PTLL-----A-------QGEARRS-----R-NTTRPALLRLSDYLL---------TNY--     51
XP_030684894.1      PTLL-----A-------QGEARRS-----R-NTTRPALLRLSDYLL---------TNY--     45
PNJ75904.1          PTLL-----A-------QGEARRS-----R-NTTRPALLRLSDYLL---------TNY--     51
AAP35868.1          PTLL-----A-------QGEARRS-----R-NTTRPALLRLSDYLL---------TNY--     51
XP_001149570.1      PTLL-----A-------QGEARRS-----R-NTTRPALLRLSDYLL---------TNY--     51
XP_003805532.1      PTLL-----A-------QGEARRS-----R-NTTRPALLRLSDYLL---------TNY--     51
XP_023063823.1      PMLL-----A-------QGEARRSGGLQGR-NTTRPALLRLSDYLL---------TNY--     50
XP_033060210.1      PMLL-----A-------QGEARRSGGLQGR-NTTRPALLRLSDYLL---------TNY--     50
```

```
XP_010370669.1      PMLL-----A-------QGEARRSGGLQGR-NTTRPALLRLSDYLL---------TNY--      50
XP_017738496.1      PMLL-----A-------QGEARRSGGLQGR-NTTRPALLRLSDYLL---------TNY--      50
XP_011782168.1      PMLL-----A-------QGEARRSGGLQGR-NTTRPALLRLSDYLL---------TNY--      50
EHH23440.1          PTLL-----A-------QGE-----GLQGR-NTTRPALLRLSDYLL---------TNY--      45
XP_003910778.3      PRLL-----A-------QGEARRSAGLQGR-NTTRPALLRLSDYLL---------TNY--      50
XP_025212649.1      PTLL-----A-------QGEARRSAGLQGR-NTTRPALLRLSDYLL---------TNY--      56
XP_008019132.1      PTLL-----A-------QGEARRSPGLQGR-NTTRPALLRLSDYLL---------TNY--      50
XP_011832421.1      -MHR-----S-------FLQARRSAGLQGR-NTTRPALLRLSDYLL---------TNY--      35
XP_011759618.1      PTLL-----A-------QGEARRSAGLQGR-NTTRPALLRLSDYLL---------TNY--      50
XP_005579749.1      PMLL-----A-------QGEARRSAGLQGR-NTTRPALLRLSDYLL---------TNY--      50
XP_011921684.1      ------------------------------------------------------------      0
XP_008259391.1      LATL-----LT------QGEARQRAGAQAW-NSSRPALLRLSDHLL---------GNY--      50
XP_012782438.1      LPTL-----LA-----------QSEGAQNQ-NSSRPALLRLSDHLL---------ADY--      46
VTJ86076.1          PTLL-----A-------QGEARQRGISQAQ-NSTRPALLRLSDHLL---------ANY--      56
XP_015345533.1      PTLL-----A-------QGEARQRGISQAQ-NSTRPALLRLSDHLL---------ANY--      56
XP_027786432.1      PTLL-----A-------QGEARQRGISQAQ-NSTRPALLRLSDHLL---------ANY--      56
XP_026248345.1      PTLL-----A-------QGEARQRGISQAQ-NSTRPALLRLSDHLL---------ANY--      50
XP_005378209.1      PTLL-----A-------RGEARGKGTAHAR-NSSRPALQRLSDHLL---------ADY--      50
XP_004466010.1      PTLL-----A-------QGEARRREATRAG-AATGPALLRLSHHLL---------ENY--      50
XP_020035707.1      PVLL-----AQ------------GKAPQTP-NSTMPALLRLSDHLL---------AHY--      45
XP_006181802.1      PTLL-----AP-------GEARHGGGLPAQ-NSSRPALLRLSDHLL---------ADY--      50
XP_006207862.1      PTLL-----AP-------GEARHGRGLPAR-NSSRPALLRLSDHLL---------ADY--      50
XP_005891968.1      PTLL-----AP------RGEGK----ETPP-NASRPALLRLSDHLL---------ANY--      47
XP_010828662.1      PTLL-----AP------RGEGK----ETPP-NASRPALLRLSDHLL---------ANY--      47
XP_019830126.1      PTLL-----AP------WGEGR----R-PP-NASRPALLRLSDHLL---------ANY--      46
XP_017914486.1      PTLL-----AP------RGEAR-----RPP-NASRPALLRLSDHLL---------ANY--      46
XP_006060266.2      PTLL-----AP------RGEAKHRGDPQPP-NASRPALLRLSDHLL---------ANY--      51
XP_020728447.1      PTLL-----AP------RGEAKHRGDAQPP-NVSRPALLRLSDHLL---------ANY--      51
KAF4008892.1        PTLL-----AP------RGEAKHRGDPQPP-NASRPALLRLSDHLL---------ANY--      51
KAB0348059.1        ---M-----VL------ACLTKHRGDPQPP-NASRPALLRLSDHLL---------ANY--      34
KAB0371113.1        ---------------------KHRGDPQPP-NASRPALLRLSDHLL---------ANY--      27
MBV96963.1          PTLL-----A-------QGEAKHRRGPQAR-DTSRPALLRLSDHLL---------ANY--      50
XP_007172048.1      PTLL-----A-------QGEAKHRRGPQAR-DASRPALLRLSDHLL---------ANY--      50
XP_007452270.1      PTLL-----A-------QGEAKHRRGPQAR-NASRPALLRLSDHLL---------ANY--      50
XP_023987108.1      SGLL-----A-------QGEGK-----EAR-NASRPALLRLSDHLL---------ANY--      45
XP_004273432.1      PTLL-----A-------QGEGK-----EAR-NASRPALLRLSDHLL---------ANY--      45
XP_004328646.1      PTLL-----A-------QGEGK-----EAR-NASRPALLRLSDHLL---------ANY--      45
XP_026937670.1      PTLL-----A-------QGEGK-----EAR-NASRPALLRLSDHLL---------ANY--      45
XP_030691031.1      PTLL-----A-------QGEGK-----EAR-NASRPALLRLSDHLL---------ANY--      45
XP_022414870.1      PTLL-----A-------QGEAKHRRGPQAR-NASRPALLRLSDHLL---------ANY--      50
XP_029064651.1      PTLL-----A-------QGEAKHRRGPQAR-NASRPALLRLSDHLL---------ANY--      50
XP_024607566.1      PTLL-----A-------QGEAKHRRGPQAQ-NASRPALLRLSDHLL---------ANY--      50
XP_032496907.1      PTLL-----A-------QGEAKHRRGPQAQ-NASRPALLRLSDHLL---------ANY--      50
XP_007535559.1      PKPL-----A-------QGGGKGA------RNTPQPSLQRLAAHLL---------AHY--      43
XP_031299536.1      PTLL-----A-------PGEARHGGGLPAQ-NSSRPALLRLSDHLL---------ADY--      50
XP_004666516.1      PMLL-----A-------QGEGPQAS------NTSRPALLRLSDHLL---------AHY--      45
XP_008830963.1      PMLL-----A-------QGEARWRGTPQAHN-TTQPALLRLSDHLL---------THY--      50
XP_005347338.1      STLL-----A-------QAEASRRAATQARN-TTLPALLRLSDHLL---------TNY--      50
XP_005069548.1      SMLL-----A-------QAEASQRAATQAHN-TTRPALLRLSDHLL---------ANY--      50
XP_027267894.1      SMLL-----A-------QAEASQRAATQAHD-TTRPALLRLSDHLL---------ANY--      50
OBS80992.1          CVKL-----F-------SFPASRRAATQAHN-TTLPALLRLSDHLL---------VNY--      37
XP_006979812.1      SLLL-----A-------QAEASRRAATRAGN-TTLPALLRLSDHLL---------ANY--      56
XP_028720687.1      SLLL-----A-------QAEASRRAATRAHN-TTLPALLRLSDHLL---------ANY--      50
XP_021489005.1      STLL-----A-------QGEGSRRKATQARSSATRPTLLRLSDHLL---------ADY--      51
XP_031199589.1      STLT-----A-------QGEGSRRRAKQARG-TTQPALLRLSDHLL---------SNY--      75
XP_028617944.1      ------------------------------------------------------------      0
XP_034347030.1      SMLT-----A-------QGEGK--EATQARG-TTQPALLRLSDHLL---------ANY--      48
NP_077370.2         SVLI-----A-------QGEGSRRRATQAHS-TTQPALLRLSDHLL---------ANY--      50
XP_032766961.1      SVLI-----A-------QGEGSRRRATQAHS-ATQPALLRLSDHLL---------ANY--      50
XP_021063186.1      ---T-----A-------PGEGSRRRATQARD-TTQPALLRLSDHLL---------ANY--      33
NP_001093114.1      SMLT-----A-------PGEGSRRRATQARD-TTQPALLRLSDHLL---------ANY--      50
XP_021028379.1      SMLT-----A-------RGEGSRRRATQARD-TTQPALLRLSDHLL---------ANY--      50
XP_004689305.1      PTLL-----T-------QGEARGRRDLQPG-NAARPAMRKLYDYLL---------SDY--      50
XP_004712871.1      PTFL-----A-------VGGARPKVCSRDH-NTTLPALLRLSSCLL---------ANY--      50
XP_004382575.1      PTLL-----V-------QGQARWRGCPQDH-STTKPALLRLSDCLL---------SNY--      50
XP_010596188.1      PTLL-----A-------QGQAKWKGCPQAH-STTKPALLRLSECLL---------SNY--      50
XP_006890855.1      PTLL-----A-------QGQARQRECPQSP-NTTKPAFLRLSDCLL---------TNY--      50
XP_006834038.1      LTLL-----A-------LGHDRQRDCSKAH-NTTKPALLRLSDCLL---------TNY--      50
XP_007934716.1      PTLL-----A-------QEQARQRGCSQAQ-NITKPALLRLSDCLL---------ANY--      50
XP_012603491.1      PTLL-----A-------QGEARRQGSPQGR-NASRPTLLRLSDHLL---------ANY--      50
XP_012663606.1      PTLL-----A-------QGEAQ-----QGR-NTTKPTLLRLSDHLL---------ANY--      45
XP_028372812.1      PVLL-----T-------QGEARHRGGLQAH-NTSRPTLHKLSDHLL---------TNY--      50
XP_008589608.1      PMLL-----A-------RGEARVRGRLQAR-NATRPALLRLSDYLL---------ANY--      50
XP_012865077.1      PTLL-----A-------QGEARRKGAPQAR-NNTLPALLRLSDHLL---------AHY--      50
NP_001166178.1      PTSL-----A-------QGEVRGKGTAQAH-NSTRPALQRLSDHLL---------ADY--      50
XP_004636553.1      PTVL-----A-------QGEARGRETAQAH-SSTRPALQRLSDHLL---------ADY--      50
XP_004856670.1      PTLL-----A-------QGEARGRGTAQAH-SFTRPALQRLSDHLL---------ADY--      50
```

```
XP_010627744.1       PTFL-----A-------QGEARERGTAQAH-NFNGPALQRLADHLL---------ADY--       50

                                                                  Loop-D
NP_509270.1          -YQYTRPVRNYSSV-LNVTV--QPQIYNLVEV----------NEQNEQIKILLWFPQS--       91
VDO93178.1           -DNRVRPVRHHSTP-I---------------N----------DERAQNIELYMWIVQM--       90
PAV91580.1           -DVRVRPILDHRKS-LKVHI--KISLYQIIEV----------DEPAQNIKLNVWMIQK--       91
VDK46997.1           -DTRVRPIIDHTKT-LKVHI--SISLYQIIEV----------DEPSQNIKLNVWMIQK--       57
VIO86814.1           -RPFQRPALNFHTP-TEIHV--RAALYQIFDL----------DYRNNIVTISGYFHLW--       123
VDP38785.1           -EIASRPIQYGNET-LNIFL--CIILKQIIDL----------DEKNQILHTSLLIQLK--       127
KOF68401.1           -QYYLRPHCGN-SS-VNITL--NLALRQIIDL----------NDSLQILHTSIWIRMQ--       64
CDJ96026.1           -EKDVRPTGHHSQP-TNVTF--GFLLNQIVEM----------DERNQVLTTRSWLNIN--       213
EFX76216.1           -DRSVRPSRNAAEP-LNITF--GLALTQIIDV----------DERNQILTTNCWMNQM--       55
KAF7403848.1         -DAAVRPAKNSSQP-LAVVF--GLSLHHIIDV----------DEKNQILTTNCWVTQI--       67
KAF7427032.1         -DAAVRPAKNSSQP-LAVVF--GLSLHHIIDV----------DEKNQILTTNCWVTQI--       82
KAE9417558.1         -DPIERPVRSHSEP-IRVNL--RILLQQLVDT--------FSDEKNQVITLVLWTQYT--       67
KJH51504.1           -VRDERPVLDSNKP-IVVSL--GVSMQQIINL----------NEKEEQLEVSAWLKFQ--       73
VDL69795.1           -EPLERPVANSSEP-VRVKM--GLILQQIVSV----------DEKNQLVDVNAWLKLS--       77
VDO32671.1           -NPLERPVENASQP-LVVKI--KMFLQQILDV----------DEKNQLISVNAWLSYS--       78
VBB32409.1           -NNLERPVENHSQP-VVIYL--KVSLQQIIDV----------DEKNQIVYVNAWLDFA--       63
VDN54565.1           -NNLERPVANHSLP-VIVYL--KVSLQQIIDV----------DEKNQIVYVNAWLDYS--       82
EGT55171.1           -NNLERPVANHSEP-VTVHL--KVALQQIIDV----------DEKNQVVYVNAWLDYVRF       80
RCN52111.1           -NNLERPVANHSKP-VTVYL--KVSLQQIIDV----------DEKNQIVYVNAWLDYI--       79
RMX54856.1           -NPQLRPVENKSDR-VTVTF--GISLHQIINV--------DE--KNQLLQTSVWVRQV--       91
XP_022781674.1       -NLLQ--LVAQPTR-ITETS------ETLIDV--------LATNGNLVIETKCCKSRT--       350
RNA37099.1           -EKDVRPVHNHSKT-VTVFV--GLTLTQIFDL----------EEKNQFLISNVWLDNE--       98
KAA0187152.1         -DRNVRPVLNASHA-VVIQL--GITLTQIFDM----------DEKNQVLTTNVWLDQE--       225
GAU96593.1           -EKSVRPVRNASTP-IVVRL--GITLTHLFDL----------DERNQVLTTMIWLDQE--       96
XP_009043980.1       -DILARPIFNASDI-VTIKF--GMALIQLLDM----------DEVNQVLTINVWLEQE--       57
VDM43573.1           -EKAVRPVRNASHT-VTVKL--GMTMTNIFEMVIASQIVTFQDEKNQVLTINVWLDQE--       175
XP_003140283.1       -EKAVRPVRNASNT-VVVRM--GMTMTNIFDM----------DEKNQVLTINVWLDQE--       66
VDN82010.1           -EKAVRPVRNASNT-VV-------------------------DEKNQVLTINVWLDQE--       74
VDO31501.1           -EKAVRPVRNASNT-VVVRM--GMTMTNIFDM----------DEKNQVLTINVWLDQE--       87
VDP19591.1           -NPIERPSANDTET-LNVSV--KFFLNQVMDV----------DEKNQVLTTIIWMDLI--       77
EDO32053.1           -SKDARPVLNVNDP-VQLKL--GIMLRQVIDL----------NERDQILTINVWIRQY--       56
TRY67230.1           -TIEERPVVNELDV-LELKF--GLELQQIIDI----------NEKDQVISTNMWLNLK--       73
KAF7391312.1         -NTLERPVANESEP-LEVKF--GITLQQIIDV----------DEKNQILTTNAWLKLS--       84
GFG30449.1           -NVLERPVMNESDP-LQLSF--GLTLMQIIDV----------DEKNQLLITNIWLKLE--       94
KQS30083.1           -NSLERPVVNESDP-LQLSF--GLTLMQIIDV----------DEKNQLLITNIWLKLE--       116
EDW57583.2           -NTLERPVLNESDP-LQLSF--GLTLMQIIDV----------DEKNQLLVTNVWLKLE--       252
KNC22799.1           -NTLERPVLNESDP-LQLSF--GLTLMQIIDV----------DEKNQLLVTNVWLKLE--       62
RZF44856.1           -NVLERPVANESDP-LQLSF--GLTLMQIIDV----------DEKNQLLITNIWLKLE--       82
ENN76856.1           -NVLERPVANESDP-LQLSF--GITLMQIIDV----------DEKNQLLITNIWLKLE--       88
KAF5300392.1         -NVLERPVANESDP-LQLSF--GLTLMQIIDV----------DEKNQLLITNIWLKLE--       92
CAB3239999.1         -NVLERPVVNESDP-LQLSF--GLTLMQIIDV----------DEKNQLLITNIWLKLE--       79
PCG77624.1           --------------------------MQIIDV----------DEKNQLLITNIWLKLE--       22
PZC79131.1           -NVLERPVVNESDP-LQLSF--GLTLMQIIDV----------DEKNQLLITNIWLKLE--       89
KAF4083067.1         -RKPFRPAVNLSNP-TITNI--SFTLYAVLGV----------NEKTQILTTFLWLRLY--       63
XP_009924659.1       LHAHILPKTDLKEP-VEVKM--DFMLVAILSV----------VEKLQTVTFYIVLNLE--       87
XP_010123544.1       VHAHILPKTNWKEP-LEVKM--DFMLVAILSV----------VEKLQTVTFYFVLNME--       85
CBN81618.1           LFSMIRPVKNYRKP-TNVTL--DVQLYAILDV----------NEKNQQFVSYIWIVMF--       83
KAF3704230.1         LFVMTRPVKNYKEP-TYVSL--ELLLYAILDV----------VEKDQKFIPYVWTVTR--       78
RUS86578.1           YNPRIIPTLDQGSI-INVSV--EFSLISILAL----------DDKRQELITNGFLGLE--       107
XP_034309618.1       -NRHIKPVKNYSKI-IPLGI--KLYLLSLQEL----------DMKSQTLHTATWLEVF--       89
PIK58946.1           -SARERPVLDSDES-VHVEI--MLQFYAMLDL----------NERDQVITTASWLNLR--       108
VUZ42516.1           VGKIGRPVRNTSET-VNVEF--GLSLFQLTDL----------DEAEQLFTVNVWNKYA--       89
PAA51166.1           KGLLGRPVKEYDNL-VQIKF--GLAFVQILDL----------DENKQLLRTNCWLRYQ--       92
TGZ55997.1           FGVVGRPVNDSKIQ-VVVAY--GLQLFQILDL----------DENKQILRTNCWSMYK--       97
VDP48851.1           FGIIGRPVNDSKIK-VDVRY--GLQLFQILDL----------DENKQILRTNCWCMHK--       53
PVD38331.1           QGKEGRPVVNTSDI-ISVHF--GLYLIQILDV----------DEKDQILKTNIWYQYE--       75
VDK73355.1           -NKHRRPAPAANKP-ITIKL--KLRLSQIIDV----------HEIDQIMTCSVWLKQV--       84
VDD84963.1           -NRLRRPAKSPQEP-VVIKL--KLRLSQIIDL----------HEIDQIMTCSVWLKQV--       82
VDK42196.1           ------------------------------------------------MTCSVWLKQV--       10
KFD58288.1           -NKLLRPVQNTSEA-VTVKV--KLRLSQLLDV----------HEKNQIITTNIWIKQI--       95
OUC49089.1           -NKLLRPVQNTSDA-VTVKV--KLRLSQLLDV-------------------------V--       92
EYC26492.1           -NKLVRPVDNNSDT-LVVRF--KLKLSQLLDV----------HEKNQIMTTNVWLQHS--       110
VDK27218.1           -NKLVRPVENDSDT-LIVRF--KLKLSQLLDV----------VCIFSVIEAYV-AFKH--       79
VDM17286.1           ------------------------------------------------MKSNVWLTMS--       10
VDL91846.1           -IGFLPPCCGKDCAIVFLVMEKTLNCSNPCVCL----LRFRQDERKEVITTKVWVEQE--       69
KAE9548540.1         -NKIPRPVKNSTEV-LLIHV--GSSLIRIIDV----------DEKNQVLTTNLWLEMQ--       83
PDM74087.1           -NPLIPPVHNASALPIVVRV--SLQLVLLIDV----------NEKDQVMHTNVWLTLV--       83
XP_024504322.1       -NSLIRPIQNDSDGPLVVRA--SLQLVLLINV----------DEKDQIMQTNVWLTLK--       84
PIO52336.1           -NSLIQPVKNISDTPIIVKI--ALQLVLLINV----------DEKDQVMHTNVWLTLK--       80
TKR73865.1           -NPLIPPVRNHSDTPIIVKM--ALQLVLLINV----------DEKDQVMHTNVWLTLK--       85
RLU23395.1           -NKLIRPVQNMTEK-VHVRF--GLAFVQLINVA----SISCFNEKNQIMKSNVWLRFV--       87
XP_002427906.1       -NKLIRPVQNMTQT-VDVRF--GLAFVQLINV----------NEKNQIMKSNVWLRLV--       88
```

```
TMW47392.1          -NKLIRPVQNMTQK-VGVRF--GLAFVQLINV----------NEKNQIMKSNVWLRLV--     86
VDD83956.1          -SPFVRPVTRHESA-VLLFM--NLSLIQILDV----------DEKRQIIKMNAWVNLD--     81
VEL33078.1          ------------------------------------------DERNEVITSNIWLEQE--     16
XP_009019088.1      -SKVIRPVYNTSDK-VIVEI--SLKLSQLIDL----------DERNQIMTTNVWVTQT--     85
KAE9536378.1        -NRLIRPVSNNTDT-VLVKL--GLRLSQLIELL----ENEFINLKDQILTTNVWLEHE--     120
KDR23473.1          -NRLIRPVSNNTDT-VLVKL--GLRLSQLIDL----------NLKDQILTTNVWLEHE--     69
KAF7266914.1        -NRLIRPVINHTET-LTVWL--SLKLSQLIEM----------NLKNQVMTTNLWVVQK--     102
KAF4519445.1        -NRLIRPVGNNSET-LTVWL--GLKLSQLIE-------------------------VK--     99
OXU25983.1          -NRLIRPVVNNTET-LTVWL--GLKLSQLIEM----------NLKNQVMTTNVWVEQK--     87
TGZ32403.1          -NRLIRPVINNTET-LTVWL--GLKLSQLIEM----------NLKNQVMTTNVWVEQK--     84
ELT94491.1          -NRLIRPVGNITET-LTVKL--GLRLTQIIDV----------DEKNQIMTTNVWLEQE--     84
VVC39575.1          -NKLVRPVLNNTDP-LPVRI--KLKLSQLIDI----------NLKNQIMTTNLWVEQY--     178
RVE49089.1          -NKLVRPVLNVSDA-LTVRI--KLKLSQLIDV----------NLKNQIMTTNLWVEQS--     82
KAB0800277.1        -NRLIRPVGNNSDR-LTVKM--GLRLSQLIDV----------NLKNQIMTTNVWVLQE--     79
TDG52197.1          -NRLIRPVGNNSDR-LTVKM--GLRLSQLID-------------------------VE--     65
TMW48669.1          -NRLIRPVGNNSDR-LTVKM--GLRLSQLIDV----------NLKNQIMTTNVWVEQE--     80
XP_025896085.1      -RRGVRPVRDWRTT-TTVAL--DVMVYAILSV----------DEKNQVVTTYIWYRQ---     90
KAF2977017.1        ------------------------------------------------------------     0
XP_009321837.1      -QKGTRPVRDWRTT-TNVAI--DLMVYAILSV----------DEKNQVLTTYIWYRQ---     74
XP_009979985.1      -QKGTRPVRDWRTT-TNVAI--DLMVYAILSV----------DEKNQVLTTYIWYRQ---     69
XP_028942374.1      -QKGTRPVRDWRTT-TNVAI--DLMVYAILSV----------DEKNQVLTTYIWYRQ---     63
PKK19633.1          -QKGTRPVRDWRTT-TNVAI--DLMVYAILGV----------DEKNQVLTTYIWYRQ---     132
XP_009894240.1      -QKGTRPVRDWRTT-TNVAI--DLMVYAILSV----------DEKNQVLTTYVWYRQ---     186
XP_032820219.1      -DKGVRPVKNWRQP-IIVAI--DVMLYAILNV----------DEKNQVLTAYIWYRQ---     119
XP_029429480.1      -KKEVRPVRNWTNP-TIVEI--DILVRKILNV----------DEKYETLKTYLWYRQ---     128
XP_030077572.1      -KKDVRPVHDWRNT-TIVEI--SIIVRAILDV----------DEKNEILRSYIWYRQ---     75
XP_033774596.1      -KKDVRPVRNWRNT-TIVEI--GIIVRAILDV----------DEKNEILRSYIWYRQ---     106
ETE72600.1          -KKDVRPVWNWRKT-TNVAI--DVMIYAILSV----------DEKNQVLTTYIWYRQHLH     105
XP_018082638.1      -KKGVRPVQDWRQT-TTVYI--DVMVYAILGV----------DEKNQVLTTYIWYRQ---     302
XP_032905410.1      -SKAVRPVRDWRRT-TTVAI--DLMVYAILNV----------DEKNQVLTTYIWYRQ---     85
XP_020773490.1      -KKGVRPVKDWRSS-TVVAI--DLMVYSILNV----------DEKNQVLTTYVWYRQ---     94
XP_033833634.1      -KKGVRPVKDWRSS-TVVAI--DLMVYSILNV----------DEKNQVLTTYVWYRQ---     120
XP_030215795.1      -KKGVRPVKDWRDS-TSVAI--DLMIYSILNV----------DEKNQVLTTYIWYRQ---     93
XP_030602980.1      -KKGVRPVKDWRTS-TNVEI--DLMVYSILNV----------DEKNQVLTTYVWYRQ---     99
XP_004573543.1      -KKGVRPVKDWNTP-TNVEI--DLMVYSILNV----------DEKNQVLTTYVWYRQ---     93
XP_005916159.1      -KKGVRPVKDWNTP-TNVEI--DLMVYSILNV----------DEKNQVLTTYVWYRQ---     93
XP_013132089.1      -KKGVRPVKDWNTP-TNVEI--DLMVYSILNV----------DEKNQVLTTYVWYRQ---     93
XP_031603488.1      -KKGVRPVKDWNTP-TNVEI--DLMVYSILNV----------DEKNQVLTTYVWYRQ---     99
XP_005725536.1      -KKGVRPVKDWNTP-TNVEI--DLMVYSILNV----------DEKNQVLTTYVWYRQ---     99
XP_026038075.1      -KKGVRPVKDWNTP-TNVEI--DLMVYSILNV----------DEKNQVLTTYVWYRQ---     99
CAF96649.1          -KKGVRPVKDWRTS-TSVAI--DLMVYSILNV----------DEKNQVLTTYVWYRQ---     55
XP_023818570.1      -KKGVRPVKDWRTS-TIVAI--DLMVYSILNV----------DEKNQVLTTYVWYRQ---     89
RVE64289.1          -KKGVRPVKDWRTS-TIVAI--DLMVYSILNV----------DEKNQVLTTYVWYRQ---     124
XP_024121971.1      -KKGVRPVKDWRTS-TIVAI--DLMVYSILNV----------DEKNQVLTTYVWYRQ---     95
XP_015817559.1      -KKGVRPVKDWRTS-TIVSI--DLMVYSILNV----------DEKNQVLTTYVWYRQ---     79
XP_015225670.1      -KKGVRPVKDWRTS-TLVAI--DLMVYSILNV----------------------------     78
XP_012722063.1      -KKGVRPVKDWRTS-TIVAI--DLMVYSILNV----------DEKNQVLTTYVWYRQ---     93
XP_032431307.1      -KKGVRPVKDWRTS-TIVAI--DLMVYSILNV----------DEKNQVLTTYVWYRQ---     93
XP_014328329.1      -KKGVRPVKDWRTS-TIVAI--DLMVYSILNV----------DEKNQVLTTYVWYRQ---     93
XP_027886578.1      -KKGVRPVKDWRTS-TIVAI--DLMVYSILNV----------DEKNQVLTTYVWYRQ---     93
XP_008426791.1      -KKGVRPVKDWRTS-TIVAI--DLMVYSILSV----------DEKNQVLTTYVWYRQ---     93
XP_014838686.1      -KKGVRPVKDWRTS-TIVAI--DLMVYSILNV----------DEKNQVLTTYVWYRQ---     93
XP_014885967.1      -KKGVRPVKDWRTS-TIVAI--DLMVYSILNV----------DEKNQVLTTYVWYRQ---     93
XP_033954312.1      -KKGVRPVKDWRTS-TIVAI--DLMVYSILNV----------DEKNQVLTTYVWYRQ---     93
XP_034089244.1      -KKGVRPVKDWRTS-TIVAI--DLMVYSILNV----------DEKNQVLTTYVWYRQ---     93
XP_010780064.1      -KKGVRPVKDWRTS-TIVAI--DLMVYSILNV----------DEKNQVLTTYVWYRQ---     109
XP_033970261.1      -KKGVRPVKDWRTS-TFVAI--DLMVYAILNV----------DEKNQVLTTYVWYRQ---     109
XP_029375575.1      -KKGVRPVKDWRTS-TIVAI--DLMVYSILNV----------DEKNQVLTTYVWYRQ---     130
KAF0023022.1        -KKGVRPVKDWRTS-TIVAI--DLMVYSILNV----------DEKNQVLTTYVWYRQ---     126
XP_019952614.1      -KKGVRPVKDWRTS-TIVAI--DLMVYSILNV----------DEKNQVLTTYVWYRQ---     82
XP_034463117.1      -KKGVRPVKDWRTS-TIVAI--DLMVYSILNV----------DEKNQVLTTYVWYRQ---     93
XP_029924656.1      -KKGVRPVKDWRKS-TTVAI--DLMVYSILNV----------DEKNQVLTTYVWYRQ---     94
XP_028323228.1      -KKGVRPVKDWRTS-TIVAI--DLMVYSILSV----------DEKNQVLTTYVWYRQ---     93
TNM84480.1          -KKGVRPVKDWRTS-TTVAI--DLMVYSILNV----------DEKNQVLTTYVWYRQ---     93
XP_029703788.1      -KKGVRPVKDWRTS-TTVAI--DLMVYSILNV----------DEKNQVLTTYVWYRQ---     93
XP_030010368.1      -KKGVRPVKDWRTS-TTVSI--DLMVYSILSV----------DEKNQVLTTYVWYRQ---     96
XP_019725056.1      -KKGVRPVKDWRTS-TLVAI--DLMVYSILNV----------DEKNQVLTTYVWYRQ---     94
XP_034034934.1      -KKGVRPVRDWRTS-TIVAI--DLMVYSILNV----------DEKNQVLTTYVWYRQ---     82
XP_029956880.1      -KKGVRPVKDWRTS-TIVAI--DLMVYSILNV----------DEKNQVLTTYVWYRQ---     93
XP_008331307.3      -KKGVRPVKDWRTS-TIVAI--DLMVYSILNV----------DEKNQVLTTYVWYRQ---     126
XP_031724845.1      -KKGVRPVKDWRTS-TLVAI--DLMVYSILNV----------DEKNQVLTTYVWYRQ---     94
XP_013859395.1      -KKGVRPVKDWRTS-TIVAI--DLMVYSILNV----------DEKNQVLTTYVWYRQ---     93
XP_017275335.1      -KKGVRPVKDWRTS-TIVAI--DLMVYSILNV----------DEKNQVLTTYVWYRQ---     93
XP_020496197.1      -KKGVRPVKDWRTS-TIVAI--DLMVYSILNV----------DEKNQVLTTYVWYRQ---     93
XP_029030400.1      -KKGVRPVKDWRTS-TLVAI--DLMVYSILNV----------DEKNQVLTTYVWYRQ---     93
XP_022060538.1      -KKGVRPVKDWRTS-TIVAI--DLMVYSILSV----------DEKNQVLTTYVWYRQ---     94
XP_023150584.1      -KKGVRPVKDWRTS-TIVAI--DLMVYSILNV----------DEKNQVLTTYVWYRQ---     94
```

XP_029303892.1	-KKGVRPVKDWRTS-TIVAI--DLMVYSILNV----------DEKNQVLTTYVWYRQ---	117
XP_020454172.1	-KKGVRPVKDWRTS-TIVAI--DLMVYSILSV----------DEKNQVLTTYVWYRQ---	92
TKS83244.1	-KKGVRPVKDWRTS-TIVAI--DLMVYSILNV----------------------------	80
XP_022616583.1	-KKGVRPVKDWRTS-TIVAI--DLMVYSILNV----------DEKNQVLTTYVWYRQ---	126
XP_030293172.1	-KKGVRPVKDWRTS-TIVAI--DLMVYSILNV----------DEKNQVLTTYVWYRQ---	93
XP_027129539.1	-KKGVRPVKDWRTS-TIVAI--DLMVYSILNV----------DEKNQVLTTYVWYRQ---	93
XP_034539538.1	-KKGVRPVKDWRTS-TIVAI--DLMVYSILNV----------DEKNQVLTTYVWYRQ---	93
XP_008277330.1	-KKGVRPVKDWRTS-TIVAI--DLMVYSILSV----------DEKNQVLTTYVWYRQ---	93
XP_028276940.1	-KKGVRPVKDWRTS-TIVAI--DLMVYSILNV----------DEKNQVLTTYVWYRQ---	93
XP_023263433.1	-KKGVRPVKDWRTS-TIVAI--DLMVYSILSV----------DEKNQVLTTYVWYRQ---	93
XP_034406336.1	-KKGVRPVKDWRTS-TIVAI--DLMVYSILNV----------DEKNQVLTTYVWYRQ---	93
XP_026184460.1	-KKGVRPVKDWRTS-TIVAI--DLMVYSILNV----------DEKNQVLTTYVWYRQ---	93
KAF1378228.1	-KKGVRPVKDWRTS-TIVAI--DLMVYSILNV----------------------------	78
XP_028450365.1	-KKGVRPVKDWRTS-TIVAI--DLMVYSILNV----------DEKNQVLTTYVWYRQ---	93
XP_031163851.1	-KKGVRPVKDWRTS-TIVAI--DLMVYSILNV----------DEKNQVLTTYVWYRQ---	93
XP_032389084.1	-KKGVRPVKDWRTS-TIVAI--DLMVYSILNV----------DEKNQVLTTYVWYRQ---	93
XP_033494682.1	-KKGVRPVKDWRTS-TIVAI--DLMVYSILNV----------DEKNQVLTTYVWYRQ---	93
XP_018544782.1	-KKGVRPVKDWRTS-TIVAI--DLMVYSILNV----------DEKNQVLTTYVWYRQ---	93
XP_026228189.1	-KKGVRPVKDWRTS-TIVAI--DLMVYSILNV----------DEKNQVLTTYVWYRQ---	93
XP_028976606.1	-KKGVRPVRNWRNC-TMVAI--DLMVYSILSV----------DEKNQVLTTYVWYRQ---	93
CAB1352378.1	-KKGVRPVRNWRNC-TMVAI--DLMVYSILSV----------DEKNQVLTTYVWYRQ---	93
XP_023866849.1	-KKGVRPVRNWRNC-TMVAI--DLMVYSILSV----------DEKNQVLTTYIWYRQ---	93
XP_013992832.1	-KKGVRPVRNWRNC-TMVAI--DLMVYSILSV----------DEKNQVLTTYIWYRQ---	93
XP_029546688.1	-KKGVRPVRNWRNC-TMVAI--DLMVYSILSV----------DEKNQVLTTYIWYRQ---	93
XP_021481546.1	-KKGVRPVRNWRNC-TMVAI--DLMVYSILSV----------DEKNQVLTTYIWYRQ---	93
XP_020339889.1	-KKGVRPVRNWRNC-TMVAI--DLMVYSILSV----------DEKNQVLTTYIWYRQ---	93
XP_029481972.1	-KKGVRPVRNWRNC-TMVAI--DLMVYSILSV----------DEKNQVLTTYIWYRQ---	93
KPP68743.1	-KKGVRPVRDWRQT-TMVAI--DLMVYSILSV----------DEKNQVLTTYIWYRQ---	70
XP_023669383.1	-KKGVRPVRDWRQT-TMVAI--DLMVYSILSV----------DEKNQVLTTYVWYRQ---	92
KAA0720096.1	--	0
XP_009293684.1	-KKGVRPVRDWRQS-TTVAI--DLMVYAILNV----------DEKNQVLTTYVWYRQ---	92
XP_026090784.1	-KKGVRPVRDWRQS-TTVAI--DLMVYAILNV----------DEKNQVLTTYVWYRQ---	92
XP_018918715.1	-KKGVRPVRDWRQS-TTVAI--DLMVYAILNV----------DEKNQVLTTYVWYRQ---	92
KAF4098549.1	-KKGVRPVRDWRQS-TTVAI--DLMVYAILNV----------DEKNQVLTTYVWYRQ---	92
XP_016084173.1	-KKGVRPVRDWRQS-TTVAI--DLMVYAILNV----------DEKNQVLTTYVWYRQ---	92
XP_016332759.1	-KKGVRPVRDWRQS-TTVAI--DLMVYAILNV----------DEKNQVLTTYVWYRQ---	92
XP_016398039.1	-KKGVRPVRDWRQS-TTVAI--DLMVYAILNV----------DEKNQVLTTYVWYRQ---	92
XP_026989970.1	-KKGVRPVKDWRDS-TMVAI--DLMVYSILNV----------DEKNQVLTTYVWYRQ---	92
TSQ12698.1	-KKGVRPVKDWRDS-TMVAI--DLMVYSILNV----------DEKNQVLTTYVWYRQ---	92
XP_017347546.1	-KKGVRPVKDWRDS-TMVAI--DLMVYSILNV----------DEKNQVLTTYVWYRQ---	87
XP_026794616.2	-KKGVRPVKDWRDS-TMVAI--DLMVYSILNV----------DEKNQVLTTYVWYRQ---	92
XP_026861889.1	-KKGVRPVKDWRYS-TMVSI--DLMVYSILNV----------DEKNQVLTTYVWYRQ---	92
XP_017575347.1	-KKGVRPVKDWRDS-TMVAI--DLMVYSILNV----------DEKNQVLTTYVWYRQ---	92
XP_022531596.1	-KKGVRPVKDWRDS-TMVAI--DLMVYSILNV----------DEKNQVLTTYVWYRQ---	92
XP_012691019.2	-KKGVRPVKNWRQS-TMVAI--DLMVYSILNV----------DEKNQVLTTYVWYRQ---	75
XP_028839723.1	-KKGVRPVRDWRQS-TMVAI--DLMVYSILNV----------DEKNQVLTTYVWYRQ---	92
XP_030638860.1	-KKAVRPVRDWRQS-TTVAI--DLMVYSILNV----------DEKNQVLTTYVWYRQ---	74
XP_007882964.1	-SKGARPVLDWRKT-TTVAI--DVMVYAILNV----------DEKNQVLTTYIWYRQ---	68
GCB70244.1	-SKGVRPVRDWRKT-TTVAI--DVMVYAILNV----------DEKNQVLTTYIWYRQ---	69
GCC26242.1	-SKGVRPVRDWRKT-TTVAI--DVMVYAILNV----------DEKNQVLTTYIWYRQ---	90
XP_020387034.1	-SKGVRPVRDWRKT-TTVAI--DVMVYAILDV----------DEKNQVLTTYIWYRQ---	81
XP_028931940.1	-KKGVRPVYDWRRP-TNVSI--DVMVYAVLSV----------DEKNQVLTTYIWYRQ---	98
XP_031762490.1	-KKGVRPVLDWRQT-TTVYI--DVMVYAILGV----------DEKNQVLTTYIWYRQ---	87
PIO32240.1	--	0
XP_018425466.1	-KKGVRPVHNWRQT-TTVFI--DVMVYAILGV----------DEKNQVLTTYIWYRQ---	88
XP_006642351.1	-KKGVRPVRDWRNS-TMVAI--DFMVYAILSV----------DEKNQVLTTYIWYRQ---	109
XP_028665372.1	-KKGVRPVRNWRNS-TLVSI--DFMVYAILSV----------DEKNQVLTTYIWYRQ---	92
GCF49814.1	-KKGVRPVKDWRRT-TNVAI--DLMVYAILS-------------------------V---	71
XP_015277816.1	-KKGVRPVKDWRRT-TNVAI--DIMVYAILSV----------DEKNQVLTTYIWYRQ---	94
XP_005987340.2	-KKGIRPVRDWRTA-TTVSI--DVIVYAILSV----------DEKNQVLTTYIWYRQ---	76
XP_019339534.1	-NKGVRPVRDWRTT-TNVAI--DVMVYAILNV----------DEKNQVLTTYIWYRQ---	93
XP_025063414.1	-NKGVRPVRDWRTT-TNVAI--DVMVYAILNV----------DEKNQVLTTYIWYRQ---	93
XP_019367488.1	-NKGVRPVWDWRTT-TNVAI--DVMVYAILNV----------DEKNQVLTTYIWYRQ---	93
XP_019412078.1	-NKGVRPVRDWRTT-TNVAI--DVMVYAILNV----------DEKNQVLTTYIWYRQ---	93
XP_014434314.1	-KKGVRPVQDWRRT-TNVAI--DVMVYAILSV----------DEKNQVLTTYIWYQQ---	93
XP_007060556.1	-RKGVRPVQDWRRT-TNVAI--DVMVYAILSV----------DEKNQVLTTYLWYRQ---	87
XP_024072175.1	-RKGVRPVQDWRRT-TNVAI--DVMVYAILSV----------DEKNQVLTTYIWYRQ---	72
XP_008170567.1	-RKGVRPVQDWRRT-TNVAI--DVMVYAILSV----------DEKNQVLTTYIWYRQ---	93
XP_034610543.1	-RKGVRPVQDWRRT-TNVAI--DVMVYAILSV----------DEKNQVLTTYIWYRQ---	93
XP_030394127.1	-RKGVRPVQDWRRT-TNVAI--DVMVYAILSV----------DEKNQVLTTYIWYRQ---	93
XP_032651960.1	-RKGVRPVQDWRRT-TNVAI--DVMVYAILSV----------DEKNQVLTTYIWYRQ---	93
XP_025020083.1	-KKDVRPVWNWRKT-TNVAI--DVMIYAILSV----------DEKNQVLTTYIWYRQ---	93
XP_026536833.1	-KKDVRPVWNWRKT-TNVAI--DVMIYAILSV----------DEKNQVLTTYIWYRQ---	93
XP_026564787.1	-KKDVRPVWNWRKT-TIVAI--DVMIYAILSV----------DEKNQVLTTYIWYRQ---	93
XP_032084675.1	-KKDVRPVWNWRKT-TNVAI--DVMIYAILSV----------DEKNQVLTTYIWYRQ---	78
XP_029139341.1	-KKDVRPVWNWRKT-TNVAI--DVMIYAILSV----------DEKNQVLTTYIWYRQ---	93
XP_034281006.1	-KKDVRPVWNWRKT-TNVAI--DVMIYAILSV----------DEKNQVLTTYIWYRQ---	93

XP_020649062.1	-TKGVRPVRNWRKA-TNVAI--DFMVYAILSV----------DEKNQVLTTYIWYRQ---	93
XP_008117087.1	-KKGVRPVRNWRKT-TNVAI--DLMIYAILSV----------DEKNQVLTTYIWYRQ---	93
XP_028564324.1	-KKDVRPVRNWRKT-TNVAI--DVMVYAILSV----------DEKNQVLTTYIWYRQ---	99
XP_033028155.1	-KKDVRPVRNWRKT-TNVAI--DVMVYAILSV----------DEKNQVLTTYIWYRQ---	98
XP_025963249.1	-EKGVRPVRDWRTT-TNVAI--DVMVYAVLSV----------DEKNQVLTTYIWYRQ---	80
XP_009668348.1	-EKGVRPVRDWRTT-TNVAI--DVMVYAILSV----------DEKNQVLTTYIWYRQ---	92
XP_013798935.1	-EKGVRPVRDWRMA-TNVAI--DVMVYAILSV----------DEKNQVLTTYIWYRQ---	76
XP_025913685.1	-XKGVRPVRDWRMA-TNVAI--DVMVYAILSV----------DEKNQVLTTYIWYRQ---	101
XP_013042552.1	-RKGTRPVRDWRTT-TTVAI--DVMVYAILSV----------DEKNQVLTTYIWYRQ---	99
XP_005030458.2	-KKSTRPVRDWRTT-TTVAI--DVMVYAILSV----------DEKNQVLTTYIWYRQ---	92
XP_032057953.1	-KKGTRPVRDWRTT-TTVAI--DVMVYAILSV----------DEKNQVLTTYIWYRQ---	75
XP_021232050.1	-RRGVRPVRDWRTT-TTVAI--DVMVYAILSV----------DEKNQVLTTYIWYRQ---	94
OXB62403.1	-RRGVRPVRDWRTT-TTVAI--DVMVYAILSV----------DEKNQVLTTYIWYRQ---	93
OXB81319.1	-RRGVRPVRDWRTT-TTVAI--DVMVYAILSV----------DEKNQVLTTYIWYRQ---	75
XP_010722007.1	-RRGVRPVRDWRTT-TTVAI--DVMVYAILSV----------DEKNQVLTTYIWYRQ---	94
XP_015739349.1	-RRGVRPVRDWRTT-TTVAI--DVMVYAILSV----------DEKNQVLTTYIWYRQ---	94
XP_031455498.1	-RRGVRPVRDWRTT-TTVAI--DVMVYAILSV----------DEKNQVLTTYIWYRQ---	95
POI27435.1	-RRGVRPVRDWRTT-TTVAI--DVMVYAILSV----------DEKNQVLTTYIWYRQ---	70
XP_004948120.1	-RRGVRPVRDWRTT-TTVAI--DVMVYAILSV----------DEKNQVLTTYIWYRQ---	95
XP_032851190.1	-QKGTRPVRDWRTT-TNVAI--DLMVYAILSV----------DEKNQVLTTYIWYRQ---	90
XP_010007255.1	-QKGTRPVRDWRTT-TNVSI--DLMVYAILSV----------DEKNQVLTTYIWYRQ---	92
XP_030320702.1	-QKGTRPVRDWRTT-TTVAI--DLMVYAILSV----------DEKNQVLTTYIWYRQ---	63
XP_010191940.1	-QKDTRPVRDWRTT-TTVAI--DLMVYAILSV----------DEKNQVLTTYIWYRQ---	90
XP_027737112.1	-QKGTRPVRDWRTT-TTVAI--DLMVYAILSV----------DEKNQVLTTYIWYRQ---	88
XP_027555032.1	-QKGTRPVRDWRTT-TTVAI--DLMVYAILSV----------DEKNQVLTTYIWYRQ---	93
XP_032565370.1	-QKGTRPVRDWRTT-TNVAI--DLMVYAILSV----------DEKNQVLTTYIWYRQ---	139
XP_027511217.1	-QKGTRPVRDWRTT-TNVAI--DLMVYAILSV----------DEKNQVLTTYIWYRQ---	238
XP_027593499.1	-QKGTRPVRDWRTT-TNVAI--DLMVYAILSV----------DEKNQVLTTYIWYRQ---	189
XP_017664924.1	-QKGTRPVRDWRTT-TNVAI--DLMVYAILSV----------DEKNQVLTTYIWYRQ---	90
XP_029817938.1	-QKGTRPVRDWRTT-TNVAI--DLMVYAILSV----------DEKNQVLTTYIWYRQ---	72
XP_005058641.1	-QKGTRPVRDWRTT-TNVAI--DLMVYAILSV----------DEKNQVLTTYIWYRQ---	123
XP_021385804.1	-QKGARPVRDWRTT-TNVAI--DLMVYAILSV----------DEKNQVLTTYIWYRQ---	92
XP_030146687.2	-QKGARPVRDWRTT-TNVAI--DLMVYAILSV----------DEKNQVLTTYIWYRQ---	137
KAF4796420.1	-QKGTRPVRDWRTT-TNVAI--DLMVYAILSV----------DEKNQVLTTYVWYRQ---	92
XP_032937581.1	-QKGTRPVRDWRTT-TNVAL--DLMVYAILSV----------DEKNQVLTTYIWYRQ---	139
XP_031989659.1	-QKGSRPVRDWRTT-TNVAI--DLMVYAILSV----------DEKNQVLTTYIWYRQ---	93
XP_010402086.1	-QKGSRPVRDWRTT-TNVAI--DLMVYAILSV----------DEKNQVLTTYIWYRQ---	92
XP_017594069.1	-QKGSRPVRDWRTT-TNVAI--DLMVYAILSV----------DEKNQVLTTYIWYRQ---	63
XP_014115268.1	-QKGTRPVRDWRTT-TNVAI--DLMVYAILSV----------DEKNQVLTTYIWYRQ---	117
XP_023797108.1	-QKGTRPVRDWRTT-TNVAI--DLMVYAILSV----------DEKNQVLTTYIWYRQ---	63
XP_033375614.1	-QKGTRPVRDWRTT-TNVAI--DLMVYAILSV----------DEKNQVLTTYIWYRQ---	63
XP_014740121.1	-QKGTRPVRDWRTT-TNVAI--DLMVYAILSV----------DEKNQVLTTYIWYRQ---	237
RLV83430.1	-QKGTRPVRDWRTT-TNVAI--DLMVYAILSV----------DEKNQVLTTYIWYRQ---	83
XP_009096098.2	-QKGTRPVRDWRTT-TNVAI--DLMVYAILSV----------DEKDQVLTTYIWYRQ---	198
TRZ15870.1	-QKGTRPVRDWRTT-TNVAI--DLMVYAILSV----------DEKNQVLTTYIWYRQ---	93
RMB91935.1	-QKGTRPVRDWRTT-TNVAI--DLMVYAILSV----------DEKNQVLTTYIWYRQ---	63
XP_030820843.1	-QKGTRPVRDWRTT-TNVAI--DLMVYAILSV----------DEKDQVLTTYIWYRQ---	139
XP_014165179.1	-QKGTRPVRDWRTT-TNVAI--DLMVYAILSV----------DEKDQVLTTYIWYRQ---	141
XP_026653582.1	------------------------MVYAILSV----------DEKDQVLTTYIWYRQ---	23
PKU35975.1	-QKDTRPVQDWRTT-TNVAI--DLMVYAILSV----------DEKNQVLTTYIWYRQVS-	91
XP_014805072.1	-QKDTRPVRDWRTT-TNVAI--DLMVYAILSV----------DEKNQVLTTYIWYRQ---	89
XP_009818330.1	--	0
OPJ68307.1	-QKGTRPVRDWRTT-TNVAI--DLMVYAILGV----------DEKNQVLTTYIWYRQ---	92
XP_008936289.1	-QKGTRPVRDWRTT-TNVAI--DLMVYAILSV----------DEKNQVLTTYIWYRQ---	93
XP_010287046.1	--DEKNQVLTTYIWYRQ---	15
XP_005240140.2	-QKSTRPVQDWRTT-TNVAI--DLMVYAILSV----------DEKNQVLTTYIWYRQ---	152
XP_005437752.2	-QKSTRPVQDWRTT-TNVAI--DLMVYAILSV----------DEKNQVLTTYIWYRQ---	152
KFV74811.1	-QKGTRPVQDWRKT-TNVAI--DLMVYAILSV----------DEKNQVLTTYIWYRQ---	57
XP_010018389.1	-QKGTRPVRDWRTT-TNVAI--DLMVYAILSV----------DEKNQVLTTYIWYRQ---	100
KQK78711.1	-QKGTRPVRDWRTT-TNVAI--DLMVYAILNV----------DEKNQVLTTYIWYRQ---	92
XP_009570162.1	-QKGTRPVRDWRTT-TNVAI--DLMVYAILGV----------DEKNQVLTTYIWYRQ---	91
KFP11268.1	-QKGTRPVRDWRTT-TNVAI--DLMVYAILSV----------DEKNQVLTTYIWYRQ---	62
KFQ98910.1	-QKGTRPVRDWRTT-TNVAI--DLMVYAILSV----------DEKNQVLTTYIWYRQ---	76
XP_012985202.3	-QKGTRPVRDWRTT-TNVAI--DLMVYAILSV----------DEKNQVLTTYIWYRQ---	92
XP_010573388.1	-QKGTRPVRDWRTT-TNVAI--DLMVYAILSV----------DEKNQVLTTYIWYRQ---	91
XP_029879496.1	-QKGTRPVRDWRTT-TNVAI--DLMVYAILSV----------DEKNQVLTTYIWYRQ---	63
XP_030361086.1	-QKGTRPVRDWRTT-TNVAI--DLMVYAILSV----------DEKNQVLTTYIWYRQ---	144
KFM00668.1	-QKGTRPVRDWRTT-TNVAI--DLMVYAILSV----------DEKNQVLTTYIWYRQ---	76
KAF1479074.1	-QKGTRPVRDWRTT-TNVAI--DLMVYAILSV----------DEKNQVLTTYIWYRQ---	55
KAF1651161.1	-QKGTRPVRDWRTT-TNVAI--DLMVYAILSV----------DEKNQVLTTYIWYRQ---	56
KAF1673648.1	-QKGTRPVRDWRTT-TNVAI--DLMVYAILSV----------DEKNQVLTTYIWYRQ---	56
KAF1493319.1	-QKGTRPVRDWRTT-TNVAI--DLMVYAILSV----------DEKNQVLTTYIWYRQ---	56
KAF1584157.1	-QKGTRPVRDWRTT-TNVAI--DLMVYAILSV----------DEKNQVLTTYIWYRQ---	56
KAF1571723.1	-QKGTRPVRDWRTT-TNVAI--DLMVYAILSV----------DEKNQVLTTYIWYRQ---	56
KAF1533169.1	-QKGTRPVRDWRTT-TNVAI--DLMVYAILSV----------DEKNQVLTTYIWYRQ---	56
KAF1638955.1	-QKGTRPVRDWRTT-TNVAI--DLMVYAILSV----------DEKNQVLTTYIWYRQ---	56
KAF1549972.1	-QKGTRPVRDWRTT-TNVAI--DLMVYAILSV----------DEKNQVLTTYIWYRQ---	56

KAF1606914.1	-QKGTRPVRDWRTT-TNVAI--DLMVYAILSV----------DEKNQVLTTYIWYRQ---	56
KAF1510957.1	-QKGTRPVRDWRTT-TNVAI--DLMVYAILSV----------DEKNQVLTTYIWYRQ---	56
KAF1498899.1	-QKGTRPVRDWRTT-TNVAI--DLMVYAILSV----------DEKNQVLTTYIWYRQ---	56
KAF1411525.1	-QKGTRPVRDWRTT-TNVAI--DLMVYAILSV----------DEKNQVLTTYIWYRQ---	56
KAF1429205.1	-QKGTRPVRDWRTT-TNVAI--DLMVYAILSV----------DEKNQVLTTYIWYRQ---	56
XP_005334318.1	-HKEVRPVHNWHDA-TTVYL--DLFVQAVLDV----------DIQNQKLKTSVWYRE---	82
XP_014395552.1	-QKDVRPVHDWTEA-TTVYL--DVFVRAVLDV----------DAQNQKLKTNVWYSE---	82
KAB0404854.1	-KKGVRPVRDWRTP-TTVSI--DVIVYAILN-------------------------V---	78
XP_007494828.1	-KKSVRPVRDWRKP-TTVSI--DVMIYAILSV----------DEKNQVLTTYIWYRQ---	93
XP_003764254.1	-KKSVRPVRDWRKP-TTVSI--DVMIYAILNV----------DEKNQVLTTYIWYRQ---	93
XP_020845489.1	-KKSVRPVLDWRKP-TTVSI--DVMIYAILSV----------DEKNQVLTTYIWYRQ---	93
XP_027703160.1	-KKSVRPVRDWRKP-TTVSI--DVMIYAILSV----------DEKNQVLTTYIWYRQ---	93
XP_017523929.1	-NKGVRPVRDWRRP-TTVSI--DVIVYAILSV----------DEKNQVLTTYIWYRQ---	91
XP_008688428.1	-EKGVRPVRDWRKP-TTVAI--DVIIYAILSV----------DEKNQVLTTYIWYRQ---	93
XP_029812166.1	-QKGVRPVRDWRKP-TTVSI--DVIVYAILSV----------DEKNQVLTTYIWYRQ---	110
XP_025749781.1	-EKGVRPVRDWRKP-TTVAI--DVIIYAILSV----------DEKNQVLTTYIWYRQ---	93
XP_004416432.1	-EKGVRPVRDWRKP-TTVAI--DVIIYAILSV----------DEKNQVLTTYIWYRQ---	93
XP_027436262.1	-EKGVRPVRDWRKP-TTVAI--DVIIYAILSV----------DEKNQVLTTYIWYRQ---	93
XP_032284025.1	-EKGVRPVRDWRKP-TTVAI--DVIIYAILSV----------DEKNQVLTTYIWYRQ---	93
XP_006735421.1	-EKGVRPVRDWRKP-TTVAI--DVIIYAILSV----------DEKNQVLTTYIWYRQ---	93
XP_021552166.1	-EKGVRPVQDWRKP-TTVAI--DVIIYAILSV----------DEKNQVLTTYIWYRQ---	93
XP_026361066.1	-EKGVRPVRDWRKP-TTVAI--DVIIYAILSV----------DEKNQVLTTYIWYRQ---	93
XP_034523598.1	-EKGVRPVRDWRKP-TTVAI--DVIIYAILSV----------DEKNQVLTTYIWYRQ---	93
NP_001297113.1	-EKGVRPVRDWRKP-TTVAI--DVIIYAILSV----------DEKNQVLTTYIWYRQ---	93
XP_032215488.1	-EKGVRPVRDWRKP-TTVAI--DVIIYAILSV----------DEKNQVLTTYIWYRQ---	93
VCX31483.1	-EKGVRPVRDWRKP-TTVAI--DVIIYAILSV----------DEKNQVLTTYIWYRQ---	93
XP_022369003.1	-EKGVRPVRDWRKP-TTVAI--DVIIYAILSV----------DEKNQVLTTYIWYRQ---	93
XP_032694248.1	-EKGVRPVRDWRKP-TTVAI--DVIIYAILSV----------DEKNQVLTTYIWYRQ---	93
NP_001041584.1	-QKGVRPVRDWRKP-TTVSI--DVIVYAILSV----------DEKNQVLTTYIWYRQ---	93
XP_025862501.1	-QKGVRPVRDWRKP-TMVSI--DVIVYAILSV----------DEKNQVLTTYIWYRQ---	93
KAF0873564.1	-QKGVRPVRDWRKP-TTVSI--DVIVYAILSV----------DEKNQVLTTYIWYRQ---	235
XP_025784751.1	-QKGVRPVRDWRKP-TTVSI--DVIVYAILSV----------DEKNQVLTTYIWYRQ---	93
XP_007075625.1	-QKGVRPVRDWRKP-TTVSI--DVIVYAILSV----------DEKNQVLTTYIWYRQ---	229
XP_030189489.1	-QKGVRPVRDWRKP-TTVSI--DVIVYAILSV----------DEKNQVLTTYIWYRQ---	93
XP_019324655.1	-QKGVRPVRDWRKP-TTVSI--DVIVYAILSV----------DEKNQVLTTYIWYRQ---	194
XP_023094886.1	-QKGVRPVRDWRKP-TTVSI--DVIVYAILSV----------DEKNQVLTTYIWYRQ---	229
XP_026892357.1	-QKGVRPVRDWRKP-TTVSI--DVIVYAILSV----------DEKNQVLTTYIWYRQ---	93
XP_016004457.1	-KKGVRPVRDWRTP-TAVSI--DVIVYAILGV----------DEKNQVLTTYIWYRQ---	88
XP_006912860.1	-KKGVRPVRDWRKP-TAVSI--DVIVYAILSV----------DEKNQVLTTYIWYRQ---	88
XP_011363679.1	-KKGVRPVWDWRKP-TAVSI--DVIVYAILSV----------DEKNQVLTTYIWYRQ---	88
XP_016076060.1	-KKGVRPVRDWRKP-TTVSI--DVIIYAILSV----------DEKNQVLTTYIWYRQ---	93
XP_008148371.1	-KKGVRPVRDWRRP-TTVSI--DVIVYAILNV----------DEKNQVLTTYIWYRQ---	93
XP_006093568.1	-KKGVRPVRDWRRP-TTVSI--DVIVYAILNV----------DEKNQVLTTYIWYRQ---	93
XP_006761601.1	-KKGVRPVRDWRRP-TTVSI--DVIVYAILNV----------DEKNQVLTTYIWYRQ---	93
XP_024426743.1	-KKGVRPVLDWRKP-TTVSI--DVIVYAILSV----------DEKNQVLTTYIWYRQ---	88
XP_019520779.1	-KKGVRPVRDWRKP-TTVAI--DVIVYAILSV----------DEKNQVLTTYIWYRQ---	93
XP_032976539.1	-KKGVRPVRDWRKP-TTVSI--DVIVYAILSV----------DEKNQVLTTYIWYRQ---	93
ELW64270.1	-EKGVRPVRDWRKP-TTVSI--DVMVYAILNV----------DEKNQVLTTYIWYRQ---	97
XP_004427367.1	-KKGVRPVRDWRKP-TTVSI--DVMVYAILSV----------DEKNQVLTTYIWYRQ---	93
XP_008529353.1	-EKGVRPVRDWRKP-TTVSI--DVIVYAILSV----------DEKNQVLTTYIWYRQ---	93
NP_001288165.1	-EKGVRPVRDWRKP-TTVSI--DVIVYAILSV----------DEKNQVLTTYIWYRQ---	93
XP_014711213.1	-EKGVRPVRDWRKP-TTVSI--DVIVYAILSV----------DEKNQVLTTYIWYRQ---	93
XP_012514621.1	-KKGVRPVWDWRKP-TTVSI--DVIVYAILSV----------DEKNQVLTTYIWYRQ---	93
XP_008071525.1	-KKSVRPVRDWRKP-TTVSI--DVIVYAILNV----------DEKNQVLTTYIWYRQ---	91
XP_009005082.1	-KKGVRPVRNWRKP-TTVSI--DVIVYAILNV----------DEKNQVLTTYIWYRQ---	93
XP_010332832.1	-KKGVRPVRDWRKP-TTISI--DVIVYAILNV----------DEKNQVLTTYIWYRQ---	88
XP_012326058.1	-KKGVRPVRDWRKP-TTISI--DVIVYAILNV----------DEKNQVLTTYIWYRQ---	99
XP_017378796.1	-KKGVRPVRDWRKP-TTISI--DVIVYAILNV----------DEKNQVLTTYIWYRQ---	122
XP_032141374.1	-KKGVRPVRDWRKP-TTISI--DVIVYAILNV----------DEKNQVLTTYIWYRQ---	122
XP_032024018.1	-RKGVRPVRDWRKP-TTVSI--DVIVYAILNV----------DEKNQVLTTYIWYRQ---	88
XP_018891497.2	-RKGVRPVRDWRKP-TTVSI--DVIVYAILNV----------DEKNQVLTTYIWYRQ---	94
XP_030684894.1	-RKGVRPVRDWRKP-TTVSI--DVIVYAILNV----------DEKNQVLTTYIWYRQ---	88
PNJ75904.1	-RKGVRPVRDWRKP-TTVSI--DVIVYAILNV----------DEKNQVLTTYIWYRQ---	94
AAP35868.1	-RKGVRPVRDWRKP-TTVSI--DVIVYAILNV----------DEKNQVLTTYIWYRQ---	94
XP_001149570.1	-RKGVRPVRDWRKP-TTISI--DVIVYAILNV----------DEKNQVLTTYIWYRQ---	94
XP_003805532.1	-RKGVRPVRDWRKP-TTVSI--DVIVYAILNV----------DEKNQVLTTYIWYRQ---	94
XP_023063823.1	-RKGVRPVRDWRKP-TTVSI--DVIVYAILDV----------DEKNQVLTTYIWYRQ---	93
XP_033060210.1	-RKGVRPVRDWRKP-TTVSI--DIIVYAILNV----------DEKNQVLTTYIWYRQ---	93
XP_010370669.1	-RKGVRPVRDWRKP-TTVSI--DVIVYAILNV----------DEKNQVLTTYIWYRQ---	93
XP_017738496.1	-RKGVRPVRDWRKP-TTVSI--DVIVYAILNV----------DEKNQVLTTYIWYRQ---	93
XP_011782168.1	-RKGVRPVRDWRKP-TTVSI--DVIVYAILNV----------DEKNQVLTTYIWYRQ---	93
EHH23440.1	-RKGVRPVRDWRKP-TTVSI--DVIVYAILNV----------DEKNQVLTTYIWYRQ---	88
XP_003910778.3	-RKGVRPVRDWRKP-TTVSI--DVIVYAILNV----------DEKNQVLTTYIWYRQ---	93
XP_025212649.1	-RKGVRPVRDWRKP-TTVSI--DVIVYAILNV----------DEKNQVLTTYIWYRQ---	99
XP_008019132.1	-RKGVRPVKDWRKP-TTVSI--DVIVYAILNV----------DEKNQVLTTYIWYRQ---	93
XP_011832421.1	-RKGVRPVRDWRKP-TTVSI--DVIVYAILNV----------DEKNQVLTTYIWYRQ---	78
XP_011759618.1	-RKGVRPVRDWRKP-TTVSI--DVIVYAILNV----------DEKNQVLTTYIWYRQ---	93

XP_005579749.1	-RKGVRPVRDWRKP-TTVSI--DVIVYAILNV----------DEKNQVLTTYIWYRQ---	93
XP_011921684.1	--	0
XP_008259391.1	-KKSVRPVRDWRKP-TTVSI--DVIIYAILSV----------DEKNQVLTTYIWYRQ---	93
XP_012782438.1	-NKSVRPVRDWRKP-TTVAI--DVIIYAILSV----------DEKNQVLTTYIWYRQ---	89
VTJ86076.1	-RKGVRPVRDWRRP-TTISI--DVIVYAILNV----------DEKNQVLTTYIWYRQ---	99
XP_015345533.1	-RKGVRPVRDWRRP-TTISI--DVIVYAILNV----------DEKNQVLTTYIWYRQ---	99
XP_027786432.1	-RKGVRPVRDWRRP-TTISI--DVIVYAILNV----------DEKNQVLTTYIWYRQ---	99
XP_026248345.1	-RKGVRPVRDWRRP-TTISI--DVIVYAILNV----------DEKNQVLTTYIWYRQ---	93
XP_005378209.1	-RKGVRPVRDWRRP-TTISI--DVIVYAILSV----------DEKNQVLTTYIWYRQ---	93
XP_004466010.1	-RKGVRPVRDWRTP-TTVSI--DVIVYAILSV----------DEKNQVLTTYIWYRQ---	93
XP_020035707.1	-KKAVRPVRDWRKP-TTVSI--DVIIYAILSV----------DEKNQVLTTYIWYRQ---	88
XP_006181802.1	-KKGVRPVRDWRKP-TTVSI--DVIVYAILNV----------DEKNQVLTTYIWYRQ---	93
XP_006207862.1	-KKGVRPVRDWRKP-TTVSI--DVIVYAILNV----------DEKNQVLTTYIWYRQ---	93
XP_005891968.1	-KKGVRPVRDWRTP-TTVSI--DVIVYAILSV----------DEKNQVLTTYIWYRQ---	90
XP_010828662.1	-KKGVRPVRDWRTP-TTVSI--DVIIYAILSV----------DEKNQVLTTYIWYRQ---	90
XP_019830126.1	-KKGVRPVRDWRTP-TTVSI--DVIVYAILSV----------DEKNQVLTTYIWYRQ---	89
XP_017914486.1	-KKGVRPVRDWRTP-TTVSI--DVIVYAILSV----------DEKNQVLTTYIWYRQ---	89
XP_006060266.2	-KKGVRPVRDWRTP-TTVSI--DVIVYAILSV----------DEKNQILTTYIWYRQ---	94
XP_020728447.1	-KKGVRPVRDWRTP-TTVSI--DVILYAILSV----------DEKNQVLTTYIWYRQ---	94
KAF4008892.1	-KKGVRPVRDWRTP-TTVSI--DVILYAILSV----------DEKNQVLTTYIWYRQ---	94
KAB0348059.1	-KKGVRPVRDWRKP-TTVSI--DVILYAILSV----------DEKNQVLTTYIWYRQ---	77
KAB0371113.1	-KKGVRPVRDWRTP-TTVSI--DVILYAILSV----------DEKNQVLTTYIWYRQ---	70
MBV96963.1	-KKGVRPVRDWRTP-TTVSI--DVIVYAILNV----------DEKNQVLTTYIWYRQ---	93
XP_007172048.1	-KKGVRPVRDWRTP-TTVSI--DVIVYAILNV----------DEKNQVLTTYIWYRQ---	93
XP_007452270.1	-KKGVRPVRDWRTP-TMVSI--DVIVYAILSV----------DEKNQVLTTYIWYRQ---	93
XP_023987108.1	-KKGVRPVRDWRTP-TTVSI--DVIVYAILSV----------DEKNQVLTTYIWYRQ---	88
XP_004273432.1	-KKGVRPVRDWRTP-TTVSI--DVIVYAILSV----------DEKNQVLTTYIWYRQ---	88
XP_004328646.1	-KKGVRPVRDWRTP-TTVSI--DVIVYAILSV----------DEKNQVLTTYIWYRQ---	88
XP_026937670.1	-KKGVRPVRDWRTP-TTVSI--DVIVYAILSV----------DEKNQVLTTYIWYRQ---	88
XP_030691031.1	-KKGVRPVRDWRTP-TTVSI--DVIVYAILSV----------DEKNQVLTTYIWYRQ---	88
XP_022414870.1	-KKGVRPVRDWRTP-TTVSI--DVIVYAILSV----------DEKNQVLTTYIWYRQ---	93
XP_029064651.1	-KKGVRPVRDWRTP-TTVSI--DVIVYAILSV----------DEKNQVLTTYIWYRQ---	93
XP_024607566.1	-KKGVRPVRDWRTP-TTVSI--DVIVYAILSV----------DEKNQVLTTYIWYRQ---	93
XP_032496907.1	-KKGVRPVRDWRTP-TTVSI--DVIVYAILSV----------DEKNQVLTTYIWYRQ---	93
XP_007535559.1	-EKGVRPVRDWRKP-TTVSI--DVIVYAILNV----------DEKNQVLTTYIWYRQ---	86
XP_031299536.1	-KKGVRPVRDWRKP-TTVSI--DVIVYAILNV----------DEKNQVLTTYIWYRQ---	93
XP_004666516.1	-RKGVRPVLDWRKP-TTISI--DVIIYAILSV----------DEKNQVLTTYIWYRQ---	88
XP_008830963.1	-RKGVRPVRDWRQP-TTIFI--DVIMYAILSV----------DEKNQVLTTYIWYRQ---	93
XP_005347338.1	-KKAVRPVRDWRKP-TTVSI--DVIMYAILSV----------DEKNQVLTTYIWYRQ---	93
XP_005069548.1	-RKGVRPVRDWRKP-TTVSI--DVIMYAILSV----------DEKNQVLTTYIWYRQ---	93
XP_027267894.1	-RKGVRPVRDWRNP-TTVSI--DVIMYAILSV----------DEKNQVLTTYIWYRQ---	93
OBS80992.1	-RKGVRPVRDWRKP-TTVSI--DVIMYAILSV----------DEKNQVLTTYIWYRQ---	80
XP_006979812.1	-KKGVRPVRDWRKP-TTVSI--DVIMYAILNV----------DEKNQVLTTYIWYRQ---	99
XP_028720687.1	-KKDVRPVRDWRKP-TTVSI--DVIMYAILNV----------DEKNQVLTTYIWYRQ---	93
XP_021489005.1	-RKGVRPVQDWRKP-TTISI--DVIMYAILSV----------DEKNQVLTTYIWYRQ---	94
XP_031199589.1	-KKGVRPVRDWRKP-TTVSI--DVIMYAILNV----------DEKNQVLTTYIWYRQ---	118
XP_028617944.1	--	0
XP_034347030.1	-RKGVRPVRDWRKP-TTVSI--DVIMYAILNV----------DEKNQVLTTYIWYRQ---	91
NP_077370.2	-KKGVRPVRDWRKP-TLVSI--DVIMYAILNV----------DEKNQVLTTYIWYRQ---	93
XP_032766961.1	-KKGVRPVRDWRKP-TLVSI--DVIMYAILNV----------DEKNQVLTTYIWYRQ---	93
XP_021063186.1	-KKGVRPVRDWRKP-TTVSI--DVIMYAILNV----------DEKNQVLTTYIWYRQ---	76
NP_001093114.1	-KKGVRPVRDWRKP-TTVSI--DVIMYAILNV----------DEKNQVLTTYIWYRQ---	93
XP_021028379.1	-KKGVRPVRDWRKP-TTVSI--DVIMYAILNV----------DEKNQVLTTYIWYRQ---	93
XP_004689305.1	-KKGVRPVRDWRKP-TTVSI--DVIMYAILSV----------DEKNQVLTTYIWYRQ---	93
XP_004712871.1	-NKGVRPVQDWRKP-TTVFI--DVMVYSIINV----------DEKNQVLTTYIWYRQ---	93
XP_004382575.1	-TKGVRPVRDWRKS-TTVLV--DVIVYAILNV----------DEKNQVLTTYIWYRQ---	93
XP_010596188.1	-TKGVRPVWDWRKP-TTVLI--DVIVYAILNV----------DEKNQVLTTYIWYRQ---	93
XP_006890855.1	-TKGVRPVRDWRKP-TTVFI--DVIVYAILSV----------DEKNQVLTTYIWYRQ---	93
XP_006834038.1	-TKGVRPVLDWRKP-TTVFI--DVIVYAILSV----------DEKNQVLTTYIWYRQ---	93
XP_007934716.1	-TKGVRPVRDWRKP-TTVFI--DVIVYAILNV----------DEKNQVLTTYIWYRQ---	93
XP_012603491.1	-KKGVRPVWNWRKP-TTVSI--DVIVYAILSV----------DEKNQVLTTYIWYRQ---	93
XP_012663606.1	-KKGVRPVWDWRKP-TTVAI--DVIVYAILSV----------DEKNQVLTTYIWYRQ---	88
XP_028372812.1	-KKGVRPVRDWRKP-TTVSI--DVIVYAILSM----------DEKNQVLTTYIWYRQ---	93
XP_008589608.1	-RKGVRPVRDWRKP-TTVAV--DVIVYAILNV----------DEKNQVLTTYIWYRQ---	93
XP_012865077.1	-KKGVRPVRDWRKP-TTISI--DVIIYAILSV----------DEKNQVLTTYIWYRQ---	93
NP_001166178.1	-RKSVRPVRDWRKP-TTVSI--DAIVYAILSV----------DEKNQVLTTYIWYRQ---	93
XP_004636553.1	-RKGVRPVRDWRKP-TTIAI--DVIVYAILNV----------DEKNQVLTTYIWYRQ---	93
XP_004856670.1	-RKGVRPVRDWRKP-TTISI--DVIVYAILSV----------DEKNQVLTTYIWYRQ---	93
XP_010627744.1	-RKGVRPVRDWRKP-TTISI--DVIVYAILSV----------DEKNQVLTTYIWYRQ---	93

Loop-D

NP_509270.1	-----------------WKDD---YLTW---D---P-----------KEWNGIERIIIPK	114
VDO93178.1	-----------------WFDE---FLYW---N---P-----------KQYEGLENLVIPY	113
PAV91580.1	-----------------WTDE---YLSW---N---P-----------REYGMINSTNIPF	114
VDK46997.1	-----------------WKDE---YLTW---D---P-----------RDYGMINSTIIPY	80

VIO86814.1	-----------------WIDP---SLRW---N---A-----------SEFNNITRTFIPS	146
VDP38785.1	-----------------WIDE---TIQL---RMLQLYDLYHRNNTTNKLKTYFPSLVLPS	164
KOF68401.1	-----------------WRDC---RLSW---N---K-----------TDFEGIDQILVSN	87
CDJ96026.1	-----------------WMDK---RLTW---N---Y-----------TEWGGIKTIYIPY	236
EFX76216.1	-----------------WTDY---SLVW---N---V-----------SEFGGIRVVRIPA	78
KAF7403848.1	-----------------WTDH---HLKW---N---I-----------SDFAGIRVIRVPY	90
KAF7427032.1	-----------------WTDH---HLKW---N---I-----------SDFAGIRVIRVPY	105
KAE9417558.1	-----------------WNDY---KMHW---D---P-----------KEYGNITNIQLPN	90
KJH51504.1	-----------------WRDE---NLRW---Q---P-----------NEYDNVTDLRHPA	96
VDL69795.1	-----------------WHDY---SLEW---D---P-----------AKYDGVADLRFNA	100
VDO32671.1	-----------------WQDY---KLTW---D---P-----------AKYDGIQNVRFPG	101
VBB32409.1	-----------------WNDY---KLRW---D---K-----------AKYGNITDVRFPA	86
VDN54565.1	-----------------WNDY---KLKW---D---K-----------SEYGNISDVRFPA	105
EGT55171.1	STP----QSSVTLFFQTWKDY---NLVW---D---Q-----------AEYGNITDVRFPA	116
RCN52111.1	-----------------WYDY---KLSW---D---M-----------SEYGNITDVRFPA	102
RMX54856.1	-----------------WN---NPFLVW---N---Q-----------TDFGGIKSVNVHS	114
XP_022781674.1	-----------------YVDDNQLYITF---PVQQR-----------ENVVEDMNKDLTK	379
RNA37099.1	-----------------WYDE---FLTW---D---P-----------DKFNGLKSIRIPS	121
KAA0187152.1	-----------------WVDQ---LLSW---D---P-----------ADYNGLETVRLPC	248
GAU96593.1	-----------------WYDE---LLTW---N---P-----------SDYNGLTVLRTPC	119
XP_009043980.1	-----------------WQDE---RLLW---N---P-----------EDYNGLNVLRIPC	80
VDM43573.1	-----------------WKDE---LLRW---D---P-----------KQFGGIESIRIPC	198
XP_003140283.1	-----------------WKDE---LLVW---D---P-----------NEFGGIESVRVPC	89
VDN82010.1	-----------------WKDE---LLVW---D---P-----------KKFGGIKSVRVPC	97
VDO31501.1	-----------------WKDE---LLVW---D---P-----------KKFGGIKSVRVPC	110
VDP19591.1	-----------------WNDY---HFLW---N---P-----------KEFGNITTLNLPY	100
EDO32053.1	-----------------WKDH---LLKW---N---P-----------DDFGGIKSINIAP	79
TRY67230.1	-----------------WQDY---SLMW---S---P-----------TEYGNISDIRLPP	96
KAF7391312.1	-----------------WNDY---NLQW---N---Q-----------SDYGGVKDVHITP	107
GFG30449.1	-----------------WNDV---NLRW---N---S-----------SEYGGVKDLRIPP	117
KQS30083.1	-----------------WNDM---NLRW---N---S-----------SEFGGVRDLRIPP	139
EDW57583.2	-----------------WNDM---NLRW---N---T-----------SDYGGVKDLRIPP	275
KNC22799.1	-----------------WNDM---NLRW---N---T-----------SDYGGIKDLRIPP	85
RZF44856.1	-----------------WNDV---NLRW---N---S-----------TEYGGVKDLRIPP	105
ENN76856.1	-----------------WNDV---NLRW---N---S-----------SEFGGVKDLRIPP	111
KAF5300392.1	-----------------WNDV---NLRW---N---T-----------SEYGGVRDLRIPP	115
CAB3239999.1	-----------------WNDM---NLRW---N---T-----------SDFGGVKDLRVPP	102
PCG77624.1	-----------------WNDM---NLRW---N---T-----------SDFGGVKDLRVPP	45
PZC79131.1	-----------------WNDM---NLRW---N---T-----------SDFGGVKDLRVPP	112
KAF4083067.1	-----------------WFHE---FLIW---D---P-----------EECDGISKISLPV	86
XP_009924659.1	-----------------WENT---FVTW---D---P-----------QDFCNISEIVLPT	110
XP_010123544.1	-----------------WKNT---FATW---D---P-----------QDFCNISKVVLPT	108
CBN81618.1	-----------------WENE---FMSW---N---P-----------KEFCGIYCLILPS	106
KAF3704230.1	-----------------WHND---YISW---D---P-----------NQFCGIDNISVPT	101
RUS86578.1	-----------------WEDE---IVQW---D---P-----------QEYG-LTVMYVYP	129
XP_034309618.1	-----------------WKDE---YLAW---N---S-----------SEFSDIQYLRLPA	112
PIK58946.1	-----------------WTDE---RLSW---N---S-----------SEFGGEDVVVIFI	131
VUZ42516.1	-----------------WYDE---VLTW---N---P-----------KEYNDIKHVRIPS	112
PAA51166.1	-----------------WRDH---HLNWRNWS---H-----------PLFKNLTQIRVFP	118
TGZ55997.1	-----------------WQDS---LLTW---N---H-----------SEYGDIRSIRIFP	120
VDP48851.1	-----------------WTDS---LLRW---N---E-----------SEYGNIKTVRIFP	76
PVD38331.1	-----------------WRDE---LLTW---N---P-----------EDYDNISDVRLPS	98
VDK73355.1	-----------------WIDK---KLSW---N---P-----------KNYGSVSVLYVPY	107
VDD84963.1	-----------------WTDS---RLSW---D---P-----------NRYGDVNVLYIPY	105
VDK42196.1	-----------------WIDK---KLSW---D---P-----------KNYGGVSVLYVPY	33
KFD58288.1	-----------------WTDC---NLRW---N---P-----------SEYGGLDVLHVPA	118
OUC49089.1	-----------------WTDS---NINW---D---P-----------NDYGGVDVLYVPA	115
EYC26492.1	-----------------WTDY---KLRW---N---P-----------DDYGGVDVLYVPS	133
VDK27218.1	-----------------WTDY---KLKW---N---P-----------ADYGGVNVLYVPS	102
VDM17286.1	-----------------WYDY---RIAW---N---P-----------SEFADIKSINIAA	33
VDL91846.1	-----------------WTDS---KFTW---N---P-----------ADYGGVRMLHVPA	92
KAE9548540.1	-----------------WTDA---KLTW---D---P-----------KKYGGITALHIPS	106
PDM74087.1	-----------------WHDF---QMRW---N---P-----------TNYGGISQIRVSP	106
XP_024504322.1	-----------------WHDF---QMKW---N---P-----------IDYGNIQDIRVSP	107
PIO52336.1	-----------------WHDF---QMRW---N---P-----------VKYGEIRQIRVQP	103
TKR73865.1	-----------------WYDF---QMKW---N---P-----------VDYGEIKNIRVAP	108
RLU23395.1	-----------------WMDY---QLQW---D---E-----------ADYGGIGVLRLPP	110
XP_002427906.1	-----------------WSDY---QLQW---D---E-----------ADYGGIGVLRLPP	111
TMW47392.1	-----------------WYDY---QLQW---D---E-----------ADYGGIGVLRLPP	109
VDD83956.1	-----------------WKDH---NFMW---D---P-----------KDHGNVTSLILPS	104
VEL33078.1	-----------------WSDN---NLRW---D---P-----------NDYNGTTQIYVPS	39
XP_009019088.1	-----------------WFDP---ALVW---K---P-----------KEFGNVDMIYVPA	108
KAE9536378.1	-----------------WADH---KFIW---E---P-----------LEYGGVKELYVPS	143
KDR23473.1	-----------------WQDH---KFRW---D---P-----------QEYGGVTELYVPS	92
KAF7266914.1	-----------------WNDY---KLRW---D---P-----------EEYGGIEMLYVPS	125
KAF4519445.1	-----------------WIDY---KLRW---D---P-----------EEYGGVEMLYVPS	122
OXU25983.1	-----------------WFDY---KLRW---D---P-----------EEYGGIEMLYVPS	110

```
TGZ32403.1       -----------------WFDY---KLQW---D---P-----------QEYGGIDMLYVPS   107
ELT94491.1       -----------------WYDY---KLQW---D---P-----------AEYGDIKHLYVPS   107
VVC39575.1       -----------------WYDY---KLTW---N---P-----------SEYGGVEGLHVPS   201
RVE49089.1       -----------------WYDY---KLSW---E---P-----------REYGGVEMLHVPS   105
KAB0800277.1     -----------------WNDY---KLKW---N---P-----------DDYGGVETLHVPS   102
TDG52197.1       -----------------WNDY---KLKW---N---P-----------DDYGGVDTLHVPS   88
TMW48669.1       -----------------WNDY---KLKW---N---P-----------DDYGGVDTLHVPS   103
XP_025896085.1   ----------------HWTDE---FLQW---D---P-----------ARFDNVTQISLP-   113
KAF2977017.1     ------------------------------------------------------------   0
XP_009321837.1   ----------------HWTDE---FLRW---D---P-----------TRFDNLTQISLP-   97
XP_009979985.1   ----------------HWTDE---FLRW---D---P-----------ARFDNLTQISLP-   92
XP_028942374.1   ----------------HWTDE---FLQW---D---P-----------ARFDNLTPGLFP-   86
PKK19633.1       ----------------HWTDE---FLRW---D---P-----------ARFDNLTQISLP-   155
XP_009894240.1   ----------------HWTDE---FLRW---D---P-----------ERFDNLTQISLP-   209
XP_032820219.1   ----------------RWTDE---FLQW---D---P-----------VQYDGIEQISIP-   142
XP_029429480.1   ----------------FWKDE---FLTW---V---P-----------EAFDNVKNISIP-   151
XP_030077572.1   ----------------FWRDE---FLTW---D---P-----------QNFDCIENISIP-   98
XP_033774596.1   ----------------FWKDE---FLTW---D---P-----------EKFDCIENISIP-   129
ETE72600.1       VGGKHDTCPCFLSTLQHWVDE---FLKW---N---P-----------QDFDNITQMSVP-   144
XP_018082638.1   ----------------FWVDE---FLTW---D---P-----------KEFENVSQISIP-   325
XP_032905410.1   ----------------FWTDE---FLQW---D---P-----------QDFDNILQLSIP-   108
XP_020773490.1   ----------------SWTDE---FLVW---D---P-----------DDFDDVKQVSLP-   117
XP_033833634.1   ----------------SWTDE---FLVW---D---P-----------EDFDDVKQVSLP-   143
XP_030215795.1   ----------------AWTDE---FMVW---N---P-----------EDFDEVKQVSIP-   116
XP_030602980.1   ----------------SWKDE---FLVW---N---S-----------EEFDDVKQISIP-   122
XP_004573543.1   ----------------SWKDE---FLVW---D---S-----------QDFDGVKKISIP-   116
XP_005916159.1   ----------------SWKDE---FLVW---D---S-----------QDFDGVKKISIP-   116
XP_013132089.1   ----------------SWKDE---FLVW---D---S-----------QDFDGVKKISIP-   116
XP_031603488.1   ----------------SWKDE---FLMW---D---S-----------QDFDGVKKISIP-   122
XP_005725536.1   ----------------SWKDE---FLVW---D---S-----------QDFDGVKKISIP-   122
XP_026038075.1   ----------------SWKDE---FLVW---D---S-----------QDFDGVKKISIP-   122
CAF96649.1       ----------------SWTDE---FLVW---N---P-----------EDFDEVKQVSMP-   78
XP_023818570.1   ----------------SWTDE---FLVW---N---P-----------EDFEEVKQVSLP-   112
RVE64289.1       ----------------SWTDE---FLVW---N---P-----------EDFDEVKQVSLP-   147
XP_024121971.1   ----------------SWTDE---FLVW---N---P-----------EDFDEVKQVSLP-   118
XP_015817559.1   ----------------SWTDE---FLVW---D---P-----------EDFDEVKQISIP-   102
XP_015225670.1   ----------------SWTDE---FLVW---N---P-----------EDFDEVKQVSLP-   101
XP_012722063.1   ----------------SWTDE---FLVW---D---P-----------EDFDEVKQVSMP-   116
XP_032431307.1   ----------------SWTDE---FLVW---N---P-----------EDFDEVKQVSLP-   116
XP_014328329.1   ----------------SWTDE---FLVW---N---P-----------EDFDEVKQVSLP-   116
XP_027886578.1   ----------------SWTDE---FLVW---N---P-----------EDFDEVKQVSLP-   116
XP_008426791.1   ----------------SWTDE---FLVW---N---P-----------EDFDEVKQVSLP-   116
XP_014838686.1   ----------------SWTDE---FLVW---N---P-----------EDFDEVKQVSLP-   116
XP_014885967.1   ----------------SWTDE---FLVW---N---P-----------EDFDEVKQVSLP-   116
XP_033954312.1   ----------------SWTDE---FLVW---N---P-----------EDFDEVKQVSLP-   116
XP_034089244.1   ----------------SWTDE---FLVW---N---P-----------EDFDEVKQVSLP-   116
XP_010780064.1   ----------------SWTDE---FLVW---N---P-----------EDFDEVKQVSLP-   132
XP_033970261.1   ----------------SWTDE---FLVW---N---P-----------EDFDEVKQLSLP-   132
XP_029375575.1   ----------------SWKDE---FLVW---N---P-----------EDFDEVKQVSIP-   153
KAF0023022.1     ----------------SWTDE---FLVW---N---P-----------EDFDEVKQVSLP-   149
XP_019952614.1   ----------------SWTDE---FLVW---N---P-----------EDFDEVKQVSLP-   105
XP_034463117.1   ----------------SWTDE---FLVW---N---P-----------EDFDEVKQVSLP-   116
XP_029924656.1   ----------------AWTDE---FLVW---D---P-----------EDFDKVKQVSIP-   117
XP_028323228.1   ----------------SWTDE---FLVW---N---P-----------EDFDEVKQVSMP-   116
TNM84480.1       ----------------SWTDE---FLVW---N---P-----------EDFDEVKQVSIP-   116
XP_029703788.1   ----------------SWTDE---FLVW---N---P-----------EDFDEVKQVSIP-   116
XP_030010368.1   ----------------SWTDE---FLVW---D---P-----------EDFDEVKQVSLP-   119
XP_019725056.1   ----------------SWTDE---FLVW---N---P-----------EDFDEVKQISLP-   117
XP_034034934.1   ----------------SWTDE---FLVW---N---P-----------EDFDEVKQISVP-   105
XP_029956880.1   ----------------SWTDE---FLVW---D---P-----------EDFDEVKQVSLP-   116
XP_008331307.3   ----------------SWTDE---FLVW---D---P-----------EDFDEVKQLSLP-   149
XP_031724845.1   ----------------SWTDE---FLVW---N---P-----------EDFDEVKQVSIP-   117
XP_013859395.1   ----------------SWTDE---FLVW---D---P-----------EDFDEVKQVSIP-   116
XP_017275335.1   ----------------SWTDE---FLVW---N---P-----------EDFDEVKQVSIP-   116
XP_020496197.1   ----------------SWKDE---FLVW---D---P-----------EDFDEVKQVSLP-   116
XP_029030400.1   ----------------MWRDE---FLVW---N---P-----------EDFDEVKQVSLP-   116
XP_022060538.1   ----------------SWTDE---FLVW---N---P-----------EDFDEVKQVSIP-   117
XP_023150584.1   ----------------SWTDE---FLVW---N---P-----------EDFDEVKQVSIP-   117
XP_029303892.1   ----------------SWTDE---FLVW---N---P-----------EDFDEVKQVSLP-   140
XP_020454172.1   ----------------SWKDE---FLVW---N---P-----------EDFDEVKQVSIP-   115
TKS83244.1       ----------------AWTDE---FLVW---D---P-----------EDFDEVKQVSIP-   103
XP_022616583.1   ----------------SWTDE---FLVW---N---P-----------EDFDEVKQVSIP-   149
XP_030293172.1   ----------------SWTDE---FLVW---N---P-----------EDFDEVKQVSIP-   116
XP_027129539.1   ----------------AWTDE---FLVW---D---P-----------EDFDEVKQVSIP-   116
XP_034539538.1   ----------------SWTDE---FLVW---N---P-----------EDFDEVKQVSIP-   116
XP_008277330.1   ----------------SWTDE---FLVW---N---P-----------EDFDEVKQVSIP-   116
XP_028276940.1   ----------------SWTDE---FLVW---N---P-----------EDFDEVKQVSIP-   116
```

```
XP_023263433.1    ----------------SWTDE---FLVW---N---P-----------EDFDEVKQVSIP-    116
XP_034406336.1    ----------------SWTDE---FLVW---D---P-----------EDFDEVKQVSIP-    116
XP_026184460.1    ----------------SWTDE---FLVW---N---P-----------EDFDEVKQVSIP-    116
KAF1378228.1      ----------------SWTDE---FLVW---N---P-----------EDFDEVKQVSLP-    101
XP_028450365.1    ----------------SWTDE---FLVW---N---P-----------EDFDEVKQVSLP-    116
XP_031163851.1    ----------------SWTDE---FLVW---N---P-----------EDFDEVKQVSLP-    116
XP_032389084.1    ----------------SWTDE---FLVW---N---P-----------EDFDEVKQVSLP-    116
XP_033494682.1    ----------------SWTDE---FLVW---N---P-----------EDFDEVKQVSIP-    116
XP_018544782.1    ----------------SWTDE---FLVW---N---P-----------EDFDEVKQVSIP-    116
XP_026228189.1    ----------------AWTDE---FLVW---N---P-----------EDFDEVKQVSIP-    116
XP_028976606.1    ----------------EWTDE---FLVW---N---P-----------KDFDDVNQTSIP-    116
CAB1352378.1      ----------------EWTDE---FLVW---N---P-----------EDFDDVNQMSIP-    116
XP_023866849.1    ----------------EWTDE---XLVW---N---P-----------EDFDDVNQMSIP-    116
XP_013992832.1    ----------------EWTDE---FLVW---N---P-----------EDFDDVNQMSIP-    116
XP_029546688.1    ----------------EWTDE---FLVW---N---P-----------EDFDDVNQMSIP-    116
XP_021481546.1    ----------------EWTDE---FLVW---N---P-----------EDFDDVNQMSIP-    116
XP_020339889.1    ----------------EWTDE---FLVW---N---P-----------EDFDDVNQMSIP-    116
XP_029481972.1    ----------------EWTDE---FLVW---N---P-----------EDFDDVNQMSIP-    116
KPP68743.1        ----------------QWVDE---FLVW---N---P-----------EDFDDVKQVSIP-    93
XP_023669383.1    ----------------QWVDE---FLVW---N---P-----------EDFDDVKQVSIP-    115
KAA0720096.1      ------------------MDE---FLVW---N---P-----------EEFDDVKQISIP-    21
XP_009293684.1    ----------------QWTDE---FLMW---D---P-----------EEFDEVKKISIP-    115
XP_026090784.1    ----------------QWTDE---FLVW---D---P-----------EKFDDVEQISIP-    115
XP_018918715.1    ----------------QWTDE---FLAW---D---P-----------EEFDEVKQISIP-    115
KAF4098549.1      ----------------QWIDE---FLAW---D---P-----------EEFDDVKQISIP-    115
XP_016084173.1    ----------------QWTDE---FLAW---D---P-----------EEFDDVKQISIP-    115
XP_016332759.1    ----------------QWIDE---FLAW---D---P-----------EEFDDVKQISIP-    115
XP_016398039.1    ----------------QWIDE---FLAW---D---P-----------EEFDDVKQISIP-    115
XP_026989970.1    ----------------EWTDE---FLVW---N---P-----------EDFDDVRQISVP-    115
TSQ12698.1        ----------------QWTDE---FLVW---N---P-----------EEFDDVLKISVP-    115
XP_017347546.1    ----------------QWIDE---FLVW---N---P-----------EDFDDVRQISIP-    110
XP_026794616.2    ----------------QWIDE---FLVW---N---P-----------EDFDDVRKISIP-    115
XP_026861889.1    ----------------QWTDE---FLVW---N---P-----------EDFDDVKQISMP-    115
XP_017575347.1    ----------------QWTDE---FLVW---N---P-----------EDFDDVKKISMP-    115
XP_022531596.1    ----------------QWIDE---FLVW---N---P-----------EDFDDVKKISMP-    115
XP_012691019.2    ----------------QWTDE---FLVW---N---P-----------EDFDEAKKASIP-    98
XP_028839723.1    ----------------QWTDE---FLVW---S---P-----------EDFDKVKQVSIP-    115
XP_030638860.1    ----------------QWFDE---FLVW---N---T-----------SDFDGVKQVSIP-    97
XP_007882964.1    ----------------FWKDE---FLVW---D---P-----------ADFDELRQISVP-    91
GCB70244.1        ----------------VWTDE---FLMW---N---P-----------KDFDDIKQISIP-    92
GCC26242.1        ----------------IWTDE---FLMW---N---P-----------KDFDDIKQISIP-    113
XP_020387034.1    ----------------IWTDE---FLMW---N---P-----------KNFDDIKQISIP-    104
XP_028931940.1    ----------------YWIDE---FLMW---N---P-----------EDFDSITQLSIP-    121
XP_031762490.1    ----------------FWVDE---FLTW---D---P-----------KKFENVSQISIP-    110
PIO32240.1        ------------------------------------------------------------    0
XP_018425466.1    ----------------FWIDE---FLTW---D---P-----------KEYENLSQISIP-    111
XP_006642351.1    ----------------KWVDE---FLVW---N---P-----------EDFDDIKQVSIP-    132
XP_028665372.1    ----------------QWKDE---FLVW---N---P-----------EDFDNLKQISIP-    115
GCF49814.1        ----------------CWTDE---FLQW---N---P-----------EEFDNLTQMSIP-    94
XP_015277816.1    ----------------CWTDE---FLTW---N---P-----------EDFDNLTQMSIR-    117
XP_005987340.2    ----------------RWMDE---FLLW---N---P-----------EEFDNVTQISVP-    99
XP_019339534.1    ----------------HWIDE---FLKW---D---P-----------EDFDNITQVSLP-    116
XP_025063414.1    ----------------HWIDE---FLKW---D---P-----------DNFDNITQVSLP-    116
XP_019367488.1    ----------------HWIDE---FLKW---H---P-----------EDFDNITQVSLP-    116
XP_019412078.1    ----------------HWIDE---FLKW---H---P-----------EDFDNITQVSLP-    116
XP_014434314.1    ----------------HWIDE---FLRW---N---P-----------EDFDNITQLSLP-    116
XP_007060556.1    ----------------HWIDE---FLRW---N---P-----------EDFDNITQMSIP-    110
XP_024072175.1    ----------------HWIDE---FLRW---N---P-----------EDFDNITQMSLP-    95
XP_008170567.1    ----------------HWIDE---FLRW---N---P-----------EDFDNITQMSLP-    116
XP_034610543.1    ----------------HWIDE---FLRW---N---P-----------EDFDNITQMSLP-    116
XP_030394127.1    ----------------HWIDE---FLRW---N---P-----------EDFDNITQMSLP-    116
XP_032651960.1    ----------------HWIDE---FLRW---N---P-----------EDFDNITQMSLP-    116
XP_025020083.1    ----------------HWIDE---FLKW---N---P-----------EDFDNITQMSVP-    116
XP_026536833.1    ----------------HWVDE---FLKW---N---P-----------QDFDNITQMSVL-    116
XP_026564787.1    ----------------HWVDE---FLKW---N---P-----------QDFDNITQMSVP-    116
XP_032084675.1    ----------------HWIDE---FLKW---N---P-----------QEFDNISQMSVP-    101
XP_029139341.1    ----------------HWIDE---FLKW---N---P-----------EDFDNITQMSVP-    116
XP_034281006.1    ----------------HWIDE---FLKW---N---P-----------QDFDNITQMSVP-    116
XP_020649062.1    ----------------HWIDE---FLQW---N---P-----------KDFDNITQMSVP-    116
XP_008117087.1    ----------------QWVDE---FLQW---K---P-----------EEFDNITQMSVP-    116
XP_028564324.1    ----------------HWIDE---FLQW---N---P-----------RDFDNITQMSVP-    122
XP_033028155.1    ----------------HWIDE---FLQW---N---P-----------REFDNITQMSVP-    121
XP_025963249.1    ----------------HWIDE---FLQW---D---P-----------AHFDNVTQISLP-    103
XP_009668348.1    ----------------HWIDE---FLQW---D---P-----------AHFDNVTHISLP-    115
XP_013798935.1    ----------------HWIDE---FLQW---D---P-----------AHFDNVTRISLP-    99
XP_025913685.1    ----------------HWIDE---FLQW---D---P-----------AHFDNVTRISLP-    124
XP_013042552.1    ----------------HWTDE---FLQW---D---P-----------ACFDNITQISLP-    122
```

XP_005030458.2	----------------HWTDE---FLQW---D---P-----------ACFDNITQISLP-	115
XP_032057953.1	----------------HWTDE---FLQW---D---P-----------ACFDNITQISLL-	98
XP_021232050.1	----------------HWTDE---FLQW---D---P-----------AHFDNITQISLP-	117
OXB62403.1	----------------HWKDE---FLQW---D---P-----------AHFDNITQISLP-	116
OXB81319.1	----------------HWKDE---FLQW---D---P-----------AHFDNITQISLP-	98
XP_010722007.1	----------------HWTDE---FLQW---D---P-----------AHFDNITQISLP-	117
XP_015739349.1	----------------HWTDE---FLQW---D---P-----------AHFDNITQISLP-	117
XP_031455498.1	----------------HWTDE---FLQW---D---P-----------AHFDNITQISLP-	118
POI27435.1	----------------HWTDE---FLQW---D---P-----------AHFDNITQISLP-	93
XP_004948120.1	----------------HWTDE---FLQW---D---P-----------AHFDNITQISLP-	118
XP_032851190.1	----------------HWMDE---FLRW---D---P-----------AHFDNLTHISLP-	113
XP_010007255.1	----------------HWTDE---FLRW---D---P-----------AHFDNLMQISLP-	115
XP_030320702.1	----------------HWTDE---FLQW---D---P-----------AHFDNLTQISLP-	86
XP_010191940.1	----------------HWTDE---FLRW---D---P-----------TRFDNLTQISLP-	113
XP_027737112.1	----------------HWTDE---FLSW---D---P-----------ARFDNLTQISLP-	111
XP_027555032.1	----------------HWTDE---FLRW---D---P-----------ARFDNLTQISLP-	116
XP_032565370.1	----------------HWTDE---FLRW---D---P-----------ARFDNLTQISLP-	162
XP_027511217.1	----------------HWTDE---FLRW---D---P-----------ARFDNLTQISLP-	261
XP_027593499.1	----------------HWTDE---FLRW---D---P-----------ARFDNLTQISLP-	212
XP_017664924.1	----------------HWTDE---FLRW---D---P-----------AHFDNLTQISLP-	113
XP_029817938.1	----------------HWTDE---FLRW---D---P-----------ARFDNLTQISLP-	95
XP_005058641.1	----------------HWTDE---FLKW---D---P-----------ARFDNLTQISLP-	146
XP_021385804.1	----------------HWTDE---FLKW---D---P-----------ARFDNLTQISLP-	115
XP_030146687.2	----------------HWTDE---FLKW---D---P-----------ARFDNLTQISLP-	160
KAF4796420.1	----------------HWTDE---FLKW---D---P-----------ARFDNLTQISLP-	115
XP_032937581.1	----------------HWTDE---FLKW---D---P-----------AHFDNLTEISLP-	162
XP_031989659.1	----------------HWTDE---FLRW---D---P-----------ARFDNLTQISLP-	116
XP_010402086.1	----------------HWTDE---FLRW---D---P-----------ARFDNLTQISLP-	115
XP_017594069.1	----------------HWTDE---FLRW---D---P-----------ARFDNLTQISLP-	86
XP_014115268.1	----------------HWTDE---FLKW---D---P-----------AHFDNLTQISLP-	140
XP_023797108.1	----------------HWTDE---FLKW---D---P-----------ARFDNLTQISLP-	86
XP_033375614.1	----------------HWTDE---FLKW---D---P-----------ARFDNLTQISLP-	86
XP_014740121.1	----------------HWTDE---FLKW---D---P-----------AHFDNLTQISLP-	260
RLV83430.1	----------------HWTDE---FLQW---D---P-----------ARFDNLTQISLP-	106
XP_009096098.2	----------------HWTDE---FLKW---D---P-----------ARFDNLTQISLP-	221
TRZ15870.1	----------------HWTDE---FLKW---D---P-----------THFDNLTQISLP-	116
RMB91935.1	----------------HWTDE---FLKW---D---P-----------ARFDNLTQISLP-	86
XP_030820843.1	----------------HWTDE---FLKW---D---P-----------ARFDNLTQISLP-	162
XP_014165179.1	----------------HWTDE---FLKW---D---P-----------ARFDNLTQISLP-	164
XP_026653582.1	----------------HWTDE---FLKW---D---P-----------DRFDNLTQISLP-	46
PKU35975.1	------------RWDRHWTDE---FLRW---D---P-----------ERFDNLTQISLP-	118
XP_014805072.1	----------------HWTDE---FLRW---D---P-----------ARFDNLTQISLP-	112
XP_009818330.1	--	0
OPJ68307.1	----------------HWTDE---FLRW---D---P-----------ARFDNLTQISLP-	115
XP_008936289.1	----------------HWIDE---FLQW---D---P-----------ERFDNLTQISLP-	116
XP_010287046.1	----------------HWTDE---FLRW---D---P-----------ARFDNLTQISLP-	38
XP_005240140.2	----------------HWTDE---FLRW---D---P-----------ALFDNLTQISLP-	175
XP_005437752.2	----------------HWTDE---FLRW---D---P-----------ARFDNLTQISLP-	175
KFV74811.1	----------------HWTDE---FLRW---D---P-----------AHFDNVTQLSLP-	80
XP_010018389.1	----------------HWTDE---FLRW---D---P-----------GRFDNLTQISLP-	123
KQK78711.1	----------------HWTXE---FLRW---D---P-----------ARFDNLTQISLP-	115
XP_009570162.1	----------------HWTDE---FLRW---D---P-----------ARFDNLTQISLP-	114
KFP11268.1	----------------HWTDE---FLRW---D---P-----------ARFDNLTQISLP-	85
KFQ98910.1	----------------HWTDE---FLRW---E---P-----------ERFDNLTQISLP-	99
XP_012985202.3	----------------HWTDE---FLRW---D---P-----------AHFDNLTQISLP-	115
XP_010573388.1	----------------HWTDE---FLRW---D---P-----------ARFDNLTQISLP-	114
XP_029879496.1	----------------HWTDE---FLRW---D---P-----------ARFDNLTQISLP-	86
XP_030361086.1	----------------HWTDE---FLRW---D---P-----------ARFDNLTQISLP-	167
KFM00668.1	----------------HWTDE---FLRW---D---P-----------TRFDNLTQISLP-	99
KAF1479074.1	----------------HWTDE---FLRW---D---P-----------TRFDNLTQISLP-	78
KAF1651161.1	----------------HWTDE---FLRW---D---P-----------MRFDNLTQISLP-	79
KAF1673648.1	----------------HWTDE---FLRW---D---P-----------TRFDNLTQISLP-	79
KAF1493319.1	----------------HWTDE---FLRW---D---P-----------TRFDNLTQISLP-	79
KAF1584157.1	----------------HWTDE---FLRW---D---P-----------TRFDNLTQISLP-	79
KAF1571723.1	----------------HWTDE---FLRW---D---P-----------TRFDNLTQISLP-	79
KAF1533169.1	----------------HWTDE---FLRW---D---P-----------TRFDNLTQISLP-	79
KAF1638955.1	----------------HWTDE---FLRW---D---P-----------TRFDNLTQISLP-	79
KAF1549972.1	----------------HWTDE---FLRW---D---P-----------TRFDNLTQISLP-	79
KAF1606914.1	----------------HWTDE---FLRW---D---P-----------TRFDNLTQISLP-	79
KAF1510957.1	----------------HWTDE---FLRW---D---P-----------TRFDNLTQISLP-	79
KAF1498899.1	----------------HWTDE---FLRW---D---P-----------TRFDNLTQISLP-	79
KAF1411525.1	----------------HWTDE---FLRW---D---P-----------TRFDNLTQISLP-	79
KAF1429205.1	----------------HWTDE---FLRW---D---P-----------TRFDNLTQISLP-	79
XP_005334318.1	----------------VWDDE---FLSW---N---S-----------SMFDEIREISLP-	105
XP_014395552.1	----------------VWNDE---FLSW---N---S-----------SMFDDISQISLP-	105
KAB0404854.1	----------------YWTDE---FLQW---N---P-----------EDFDNITKLSIP-	101
XP_007494828.1	----------------YWIDE---FLKW---N---P-----------EDFDNITQLSIP-	116

```
XP_003764254.1      ----------------YWIDE---FLKW---N---P-----------EDFDNITQLSIP-     116
XP_020845489.1      ----------------YWVDE---FLKW---N---P-----------EDFDNITQLSIP-     116
XP_027703160.1      ----------------YWVDE---FLKW---N---P-----------EDFDNITQLSIP-     116
XP_017523929.1      ----------------YWTDE---FLQW---D---P-----------GDFDNITKLSIP-     114
XP_008688428.1      ----------------YWTDE---FLQW---N---P-----------EDFDNITKLSIP-     116
XP_029812166.1      ----------------YWTDE---FLQW---N---P-----------EDFDNITKLSIP-     133
XP_025749781.1      ----------------YWTDE---FLQW---N---P-----------EDFDNITKLSIP-     116
XP_004416432.1      ----------------YWTDE---FLQW---N---P-----------EDFDNITKLSIP-     116
XP_027436262.1      ----------------YWTDE---FLQW---N---P-----------EDFDNITKLSIP-     116
XP_032284025.1      ----------------YWTDE---FLQW---N---P-----------EDFDNITKLSIP-     116
XP_006735421.1      ----------------YWTDE---FLQW---N---P-----------EDFDNITKLSIP-     116
XP_021552166.1      ----------------YWTDE---FLQW---N---P-----------EDFDNITKLSIP-     116
XP_026361066.1      ----------------YWTDE---FLQW---N---P-----------EDFDNITKLSIP-     116
XP_034523598.1      ----------------YWTDE---FLQW---N---P-----------EDFDNITKLSIP-     116
NP_001297113.1      ----------------YWTDE---FLQW---N---P-----------EDFDNITKLSVP-     116
XP_032215488.1      ----------------YWTDE---FLQW---N---P-----------EDFDNITKLSVP-     116
VCX31483.1          ----------------YWTDE---FLQW---N---P-----------EDFDNITKLSIP-     116
XP_022369003.1      ----------------YWTDE---FLQW---N---P-----------EDFDNITKLSIP-     116
XP_032694248.1      ----------------YWTDE---FLQW---N---P-----------EDFDNITKLSIP-     116
NP_001041584.1      ----------------YWTDE---FLQW---N---P-----------EDFDNITKLSIP-     116
XP_025862501.1      ----------------YWTDE---FLQW---N---P-----------EDFDNITKLSIP-     116
KAF0873564.1        ----------------YWTDE---FLQW---N---P-----------EDFDNITKLSIP-     258
XP_025784751.1      ----------------YWTDE---FLQW---N---P-----------EDFDNITKLSIP-     116
XP_007075625.1      ----------------YWTDE---FLQW---N---P-----------EDFDNITKLSLP-     252
XP_030189489.1      ----------------YWTDE---FLQW---N---P-----------EDFDNITKLSIP-     116
XP_019324655.1      ----------------YWTDE---FLQW---N---P-----------EDFDNITKLSIP-     217
XP_023094886.1      ----------------YWTDE---FLQW---N---P-----------EDFDNITKLSIP-     252
XP_026892357.1      ----------------YWTDE---FLQW---N---P-----------EDFDNITKLSIP-     116
XP_016004457.1      ----------------WWTDE---FLQW---N---P-----------EDFDNITKLSIL-     111
XP_006912860.1      ----------------WWMDE---FLQW---N---P-----------EDFDNITKLSIL-     111
XP_011363679.1      ----------------WWMDE---FLQW---N---P-----------EDFDNITKLSIL-     111
XP_016076060.1      ----------------YWTDE---FLQW---N---P-----------EDFDNITKLSIP-     116
XP_008148371.1      ----------------YWTDE---FLQW---N---P-----------EDFDNITKLSIP-     116
XP_006093568.1      ----------------YWTDE---FLQW---N---P-----------EDFDNITKLSIP-     116
XP_006761601.1      ----------------YWTDE---FLQW---N---P-----------EDFDNITKLSIP-     116
XP_024426743.1      ----------------YWTDE---FLRW---N---P-----------KDFDNITKLSIP-     111
XP_019520779.1      ----------------YWTDE---FLQW---N---P-----------EDFDNITKLSIP-     116
XP_032976539.1      ----------------YWTDE---FLQW---N---P-----------EDFDNITKLSIP-     116
ELW64270.1          ----------------YWTDE---FLQW---N---P-----------KDFDNITKLSIP-     120
XP_004427367.1      ----------------YWTDE---FLQW---N---P-----------EDFDNITKLSIP-     116
XP_008529353.1      ----------------YWTDE---FLQW---N---P-----------EDFDNITQLSIP-     116
NP_001288165.1      ----------------YWTDE---FLQW---N---P-----------EDFDNITQLSIP-     116
XP_014711213.1      ----------------YWTDE---FLQW---N---P-----------EDFDNITQLSIP-     116
XP_012514621.1      ----------------HWTDE---FLRW---N---P-----------EDFDNITKLSIP-     116
XP_008071525.1      ----------------YWTDE---FLQW---S---P-----------EDFDNITKLSIP-     114
XP_009005082.1      ----------------YWTDE---FLQW---N---P-----------EDFDNITKLSIP-     116
XP_010332832.1      ----------------YWTDE---FLQW---N---P-----------EDFDNITKLSIP-     111
XP_012326058.1      ----------------YWTDE---FLQW---N---P-----------EDFDNITKLSIP-     122
XP_017378796.1      ----------------YWTDE---FLQW---N---P-----------EDFDNITKLSIP-     145
XP_032141374.1      ----------------YWTDE---FLQW---N---P-----------EDFDNITKLSIP-     145
XP_032024018.1      ----------------YWTDE---FLQW---N---P-----------EDFDNITKLSIP-     111
XP_018891497.2      ----------------YWTDE---FLQW---N---P-----------EDFDNITKLSIP-     117
XP_030684894.1      ----------------YWTDE---FLQW---N---P-----------EDFDNITKLSIP-     111
PNJ75904.1          ----------------YWTDE---FLQW---N---P-----------EDFDNITKLSIP-     117
AAP35868.1          ----------------YWTDE---FLQW---N---P-----------EDFDNITKLSIP-     117
XP_001149570.1      ----------------HWTDE---FLQW---N---P-----------EDFDNITKLSIP-     117
XP_003805532.1      ----------------HWTDE---FLQW---N---P-----------EDFDNITKLSIP-     117
XP_023063823.1      ----------------YWTDE---FLQW---N---P-----------EDFDNITKLSIP-     116
XP_033060210.1      ----------------YWTDE---FLQW---N---P-----------EDFDNITKLSIP-     116
XP_010370669.1      ----------------YWTDE---FLQW---N---P-----------EDFDNITKLSIP-     116
XP_017738496.1      ----------------YWTDE---FLQW---N---P-----------EDFDNITKLSIP-     116
XP_011782168.1      ----------------YWTDE---FLQW---N---P-----------EDFDNITKLSIP-     116
EHH23440.1          ----------------YWTDE---FLQW---N---P-----------EDFDNITKLSIP-     111
XP_003910778.3      ----------------YWTDE---FLQW---N---P-----------EDFDNITKLSIP-     116
XP_025212649.1      ----------------YWTDE---FLQW---N---P-----------EDFDNITKLSIP-     122
XP_008019132.1      ----------------YWTDE---FLQW---N---P-----------EDFDNITKLSIP-     116
XP_011832421.1      ----------------YWTDE---FLQW---N---P-----------EDFDNITKLSIP-     101
XP_011759618.1      ----------------YWTDE---FLQW---N---P-----------EDFDNITKLSIP-     116
XP_005579749.1      ----------------YWTDE---FLQW---N---P-----------EDFDNITKLSIP-     116
XP_011921684.1      ------------------------------------------------------------     0
XP_008259391.1      ----------------CWTDE---FLQW---N---P-----------EDFDNITKLSIP-     116
XP_012782438.1      ----------------FWTDE---FLQW---N---P-----------EDFDNITKLSIP-     112
VTJ86076.1          ----------------FWTDE---FLQW---N---P-----------EDFDNITKLSVP-     122
XP_015345533.1      ----------------FWTDE---FLQW---N---P-----------EDFDNITKLSVP-     122
XP_027786432.1      ----------------FWTDE---FLQW---N---P-----------EDFDNITKLSVP-     122
XP_026248345.1      ----------------FWTDE---FLQW---N---P-----------EDFDNITKLSVP-     116
XP_005378209.1      ----------------FWTDE---FLQW---N---P-----------EDFDNITKLSIP-     116
```

```
XP_004466010.1     ----------------YWNDE---FLQW---N---P-----------EDFDNITKLSIP-     116
XP_020035707.1     ----------------FWTDE---FLQW---N---P-----------EDFDNITKLSIP-     111
XP_006181802.1     ----------------FWADE---FLQW---N---P-----------EDFDNITKLSIP-     116
XP_006207862.1     ----------------FWTDE---FLQW---N---P-----------EDFDNITKLSIP-     116
XP_005891968.1     ----------------YWTDE---FLQW---N---P-----------EDFDNITKLSIP-     113
XP_010828662.1     ----------------YWTDE---FLQW---N---P-----------EDFDNITKLSIP-     113
XP_019830126.1     ----------------YWTDE---FLQW---D---P-----------EDFDNITKLSIP-     112
XP_017914486.1     ----------------YWTDE---FLQW---N---P-----------EDFDNITKLSIP-     112
XP_006060266.2     ----------------YWTDE---FLQW---N---P-----------EDFDNITKLSIP-     117
XP_020728447.1     ----------------YWTDE---FLQW---D---P-----------EDFDNITKLSIP-     117
KAF4008892.1       ----------------YWTDE---FLQW---N---P-----------EDFDNITKLSIP-     117
KAB0348059.1       ----------------YWTDE---FLQW---N---P-----------EDFDNITKLSIP-     100
KAB0371113.1       ----------------YWTDE---FLQW---N---P-----------EDFDNITKLSIP-     93
MBV96963.1         ----------------YWTDE---FLQW---N---P-----------EDFDNITKLSIP-     116
XP_007172048.1     ----------------YWTDE---FLQW---N---P-----------EDFDNITKLSIP-     116
XP_007452270.1     ----------------YWTDE---FLQW---N---P-----------EDFDNITKLSIP-     116
XP_023987108.1     ----------------YWTDE---FLQW---N---P-----------EDFDNITKLSIP-     111
XP_004273432.1     ----------------YWTDE---FLQW---N---P-----------EDFDNITKLSIP-     111
XP_004328646.1     ----------------YWTDE---FLQW---N---P-----------EDFDNITKLSIP-     111
XP_026937670.1     ----------------YWTDE---FLQW---N---P-----------EDFDNITKLSIP-     111
XP_030691031.1     ----------------YWTDE---FLQW---N---P-----------EDFDNITKLSIP-     111
XP_022414870.1     ----------------YWTDE---FLQW---N---P-----------EDFDNITKLSIP-     116
XP_029064651.1     ----------------YWTDE---FLQW---N---P-----------QDFDNITKLSIP-     116
XP_024607566.1     ----------------YWTDE---FLQW---N---P-----------EDFDNITKLSIP-     116
XP_032496907.1     ----------------YWTDE---FLQW---N---P-----------EDFDNITKLSIP-     116
XP_007535559.1     ----------------FWTDE---FLQW---N---P-----------EDFDNITRLSIP-     109
XP_031299536.1     ----------------FLS------IQW---N---P-----------XXLXQLSSPSVHP     114
XP_004666516.1     ----------------FWTDE---FLQW---N---P-----------EDFDNITKLSIP-     111
XP_008830963.1     ----------------FWTDE---FLQW---N---P-----------EDFDNITKFSIP-     116
XP_005347338.1     ----------------FWTDE---FLQW---T---P-----------EDFDNITKLSVP-     116
XP_005069548.1     ----------------FWTDE---FLQW---T---P-----------EDFDNITKLSIP-     116
XP_027267894.1     ----------------FWTDE---FLQW---T---P-----------EDFDNITKLSIP-     116
OBS80992.1         ----------------FWTDE---FLRW---T---P-----------EDFDNITKLSIP-     103
XP_006979812.1     ----------------FWTDE---FLQW---T---P-----------EDFDNITKLSIP-     122
XP_028720687.1     ----------------FWTDE---FLQW---T---P-----------EDFDNITKLSIP-     116
XP_021489005.1     ----------------FWTDE---FLQW---T---P-----------EDFDNVTKLSIP-     117
XP_031199589.1     ----------------YWTDE---FLQW---T---P-----------EDFDNVTKLSIL-     141
XP_028617944.1     ------------------------------------------------------------     0
XP_034347030.1     ----------------FWTDE---FLQW---T---P-----------EDFDNVTKLSIP-     114
NP_077370.2        ----------------FWTDE---FLQW---T---P-----------EDFDNVTKLSIP-     116
XP_032766961.1     ----------------FWTDE---FLQW---T---P-----------EDFDNVTKLSIP-     116
XP_021063186.1     ----------------FWTDE---FLQW---T---P-----------EDFDNVTKLSIP-     99
NP_001093114.1     ----------------YWTDE---FLQW---T---P-----------EDFDNVTKLSIP-     116
XP_021028379.1     ----------------FWTDE---FLQW---T---P-----------EDFDNVTKLSIP-     116
XP_004689305.1     ----------------FWTDE---FLQW---N---P-----------EDFDNITKLSIP-     116
XP_004712871.1     ----------------CWKDE---LLQW---D---P-----------EDFDNVTKLSIP-     116
XP_004382575.1     ----------------CWTDE---FLQW---N---P-----------EDFDNITRLSIP-     116
XP_010596188.1     ----------------CWMDE---FLQW---H---P-----------EDFDNITKLSIP-     116
XP_006890855.1     ----------------CWIDE---FLQW---N---P-----------EDFDNVTKLSIP-     116
XP_006834038.1     ----------------CWTDE---FLQW---S---P-----------EDFDNITKLSIP-     116
XP_007934716.1     ----------------CWTDE---FLQW---N---P-----------EDFDNITKLSIP-     116
XP_012603491.1     ----------------HWTDE---FLQW---N---P-----------KDFDNITKLSIP-     116
XP_012663606.1     ----------------HWTDE---FLQW---N---P-----------KDFDNISKLSIP-     111
XP_028372812.1     ----------------YWTDE---FLLW---N---P-----------EDFDNITKLSIP-     116
XP_008589608.1     ----------------FWIDE---FLQW---N---P-----------EDFDNITKLSIP-     116
XP_012865077.1     ----------------FWTDE---FLQW---N---P-----------EDFDNITKLSIP-     116
NP_001166178.1     ----------------FWTDE---FLQW---N---P-----------EDFDNITKLSIP-     116
XP_004636553.1     ----------------FWTDE---FLQW---N---P-----------EDFDNITKLSIP-     116
XP_004856670.1     ----------------FWADE---FLQW---N---P-----------EDFDNITKLSIP-     116
XP_010627744.1     ----------------FWADE---FLQW---N---P-----------EDFDNITKLSIP-     116
```

Loop-A

```
NP_509270.1        --SQIWIPDGYIFNT------------------VEETE-PLE---NH----NAR------     140
VDO93178.1         --NLIWLPDTYLYNG------------------LVMER-EKS---ERWLSVILSMNRSNL     149
PAV91580.1         --NHLWIPDTYLYNS------------------VTMSR-DET---ERYMNIQVQSNY---     147
VDK46997.1         --KYLWIPDTYLYNS------------------VKMSR-DET---ERYMNIQVESNH---     113
VIO86814.1         --KWFWKPELYLYHS------------------AQDRV-MD-----YAPDAVA-------     173
VDP38785.1         --SRIWTPDLYVYNN------------------ADDGK-NGL---LDVSHSRV-------     193
KOF68401.1         --KEVWIPDIALYEN------------------VDYVL-KGI---LEYY--NV-------     114
CDJ96026.1         --RRLWKPDIILVNN------------------AIREY-HA-----SLVSTDI-------     263
EFX76216.1         --DKVWKSDIILYNN------------------ADSEY-NS-----ALLSTNV-------     105
KAF7403848.1       --NRVWRPDIILYNN------------------ADPQY-SS-----AIINTNV-------     117
KAF7427032.1       --NRVWRPDIILYNN------------------ADPQY-SS-----AIINTNV-------     132
KAE9417558.1       --NFLWKPDILLFNS------------------ADEHF-D------ASFPVNF-------     116
KJH51504.1         --GTLWQPDILLYNS------------------VDPAF-D------SMYKVNL-------     122
```

```
VDL69795.1         ----------------------------------DPQF-D------STFQSNL-------     112
VDO32671.1         SADHIWKPDILLYNS------------------AAEDF-D------STYKSNL-------     129
VBB32409.1         --GKIWKPDVLLYNS------------------VDANF-D------STYPTNM-------     112
VDN54565.1         --GRIWKPDVLLYNS------------------VDANF-D------STYPTNM-------     131
EGT55171.1         --GKIWKPDVLLYNS------------------VDTNF-D------STYQTNM-------     142
RCN52111.1         --GRIWKPDVLLYNS------------------VDANF-D------STYQTNM-------     128
RMX54856.1         --SQVWIPDIYLYNN------------------ADHER-EGG---MDQFHTQI-------     143
XP_022781674.1     --IGNW---MFSKTS------------------ADHER-QSG---MDQFHTQI-------     405
RNA37099.1         --RRIWLPDLVLYNN------------------ADDYF-S------YKKDTYA-------     147
KAA0187152.1       --ERIWLPDIVLYNN------------------ADDYT-R------GYMNSKA-------     274
GAU96593.1         --ELLWLPDIVLYNN------------------ADDYT-S------GYMKSKA-------     145
XP_009043980.1     --EKIWLPDIVLYNS------------------ADDFT-T------GYMKSKA-------     106
VDM43573.1         --DLIWLPDIVLYNN------------------ADDYT-A------GYMRSRA-------     224
XP_003140283.1     --DLIWLPDIVLYNN------------------ADDYT-I------GSMNSRA-------     115
VDN82010.1         --DLIWLPDIVLYNKQNFSVLLTFSYQSHIRFSADDYA-V------GSMHSRA-------     141
VDO31501.1         --DLIWLPDIVLYNKQ-----------SHIRFSADDYT-V------GSMHSRA-------     143
VDP19591.1         --TAVWRPDILLYNC------------------ADEKF-D------RTFPTNT-------     126
EDO32053.1         --DRVWKPDVLLYNR------------------TDLRF-GSL---NNKIDTNV-------     108
TRY67230.1         --HKIWTPDILLTNS------------------ASQVF-N------PTHPTNI-------     122
KAF7391312.1       --NKVWRPDILMYNS------------------ADEGF-D------GTFQTNV-------     133
GFG30449.1         --HRIWKPDVLMYNS------------------ADEGF-D------GTYPTNV-------     143
KQS30083.1         --HRLWKPDVLMYNS------------------ADEGF-D------GTYATNV-------     165
EDW57583.2         --HRIWKPDVLMYNS------------------ADEGF-D------GTYQTNV-------     301
KNC22799.1         --HRIWKPDVLMYNS------------------ADEGF-D------GTYQTNV-------     111
RZF44856.1         --HRIWKPDVLMYNS------------------ADEGF-D------GTYPTNV-------     131
ENN76856.1         --HRLWKPDVLMYNS------------------ADEGF-D------GTYPTNV-------     137
KAF5300392.1       --HRIWKPDVLMYNS------------------ADEGF-D------GTYPTNV-------     141
CAB3239999.1       --HRLWKPDVLMYNS------------------ADEGF-D------STYPTNV-------     128
PCG77624.1         --HRLWKPDVLMYNS------------------ADEGF-D------STYPTNV-------     71
PZC79131.1         --HRLWKPDVLMYNS------------------ADEGF-D------STYPTNV-------     138
KAF4083067.1       --NNLWSPDILIYEF------------------VDDDV--------SQACPYV-------     111
XP_009924659.1     --DTYWSPTIFILER------------------VNGQ---------NSNLDYM-------     134
XP_010123544.1     --DTYWSPPIFIFER------------------VNGQ---------NSNLDYM-------     132
CBN81618.1         --ELLWKPDLTIIEM------------------TEKDK--------APPTPYL-------     131
KAF3704230.1       --EILWKPDLTIEEM------------------TEKDK--------APPSPYL-------     126
RUS86578.1         --KDVWRPRLILWNT------------------LGERDLFKD------DYSPT-------     156
XP_034309618.1     --DKVWVPSICNIQE------------------ISGKR-CVTYGSVADTSSEV-------     144
PIK58946.1         --DEIWTPKLFISNA------------------LDIDSLNII----SPERGSI-------     160
VUZ42516.1         --KHVWTPDIVLLNY------------------ADKRL-QEL------REVML-------     138
PAA51166.1         --DLIWTPDIQLLNF------------------ADERL-SER------REARI-------     144
TGZ55997.1         --SNIWTPDIKLYNF------------------ADERL-KEH------REARV-------     146
VDP48851.1         --SQIWTPDIKLYNF------------------ADERL-REY------RDARV-------     102
PVD38331.1         --DKIWIPDILLYNF------------------ADDRL-KEQ------RNALV-------     124
VDK73355.1         --EMIWVPDIVLYNK---------------------------------------------     120
VDD84963.1         --EMIWVPDIVLYNN------------------ADSAY-NIT------ISTKA-------     131
VDK42196.1         --EMIWVPDIVLYNN------------------ADSNY-NIT------ISTKA-------     59
KFD58288.1         --DQLWTPNIVLYNN------------------ADGKY-QVN------AITKA-------     144
OUC49089.1         --DQLWTPNVVLYNN------------------ADGNY-QVS------AVTKA-------     141
EYC26492.1         --DTIWLPDIVLYNN------------------ADGNY-QVT------IMTKA-------     159
VDK27218.1         --DMIWLPDIVLYNN------------------ADGNY-QVS------IMTKA-------     128
VDM17286.1         --HKLWKPDIVVMNN------------------VDGEF-EAV------WKPNT-------     59
VDL91846.1         --EEIWKPDIVLYNN------------------ADGKY-EIT------TLTKA-------     118
KAE9548540.1       --DMIWVPDFVLYNN------------------AAGDP-DIT------IYTDA-------     132
PDM74087.1         --DKVWLPDIVLFNN------------------ADGNF-EVS------FMCNV-------     132
XP_024504322.1     --DKVWVPDIVLFNN------------------ADGNY-EVS------FMCNV-------     133
PIO52336.1         --DKVWLPDIVLFNN------------------ADGNY-EVS------FMCNV-------     129
TKR73865.1         --DKVWLPDIVLFNN------------------ADGNY-EVS------FMCNV-------     134
RLU23395.1         --DKVWKPDIVLFNN------------------ADGNY-EVR------YKSNV-------     136
XP_002427906.1     --DKVWKPDIVLFNN------------------ADGNY-EVR------YKSNV-------     137
TMW47392.1         --DKVWKPDIVLFNN------------------ADGNY-EVR------YKSNV-------     135
VDD83956.1         --QDIWTPDLALYNN------------------AGGDW-DLA----SSSSTKV-------     132
VEL33078.1         --QMIWKPDIVLFNN------------------ADGKY-EIT------VMTKA-------     65
XP_009019088.1     --NSIWVPDIVLYNT------------------ADGVY-EMT------SGTKA-------     134
KAE9536378.1       --EHIWLPDIVLYNN------------------ADGEY-VVT------TMTKA-------     169
KDR23473.1         --EHIWLPDIVLYNN------------------ADGEY-VVT------TMTKA-------     118
KAF7266914.1       --EHIWLPDIVLFNN------------------ADGNY-EVT------LMTKA-------     151
KAF4519445.1       --EHIWLPDIVLFNN------------------ADGNY-EVT------LMTKA-------     148
OXU25983.1         --ENIWLPDIVMYNN------------------ADGNY-EVT------LMTKA-------     136
TGZ32403.1         --ENIWLPDIVLYNN------------------ADGNY-EVT------LMTKA-------     133
ELT94491.1         --NEIWLPDIVLYNS------------------ADGNF-TVK------LMTKA-------     133
VVC39575.1         --EHVWRPDIVLYNN------------------ADGNF-EVT------LATKA-------     227
RVE49089.1         --DHIWRPDIVLYNN------------------ADGNF-EVT------LATKA-------     131
KAB0800277.1       --EHIWLPDIVLYNN------------------ADGNY-EVT------IMTKA-------     128
TDG52197.1         --EHIWLPDIVLYNN------------------ADGNY-EVT------IMTKA-------     114
TMW48669.1         --EHIWLPDIVLYNN------------------ADGNY-EVT------IMTKA-------     129
XP_025896085.1     -AESIWVPD--ILINEF----------------VDVGR--------SPEIPYV-------     139
KAF2977017.1       ------------------------------------------------------------     0
```

```
XP_009321837.1      -VESIWVPDILIN--EL----------------XV-RX--------N---PWG-------      119
XP_009979985.1      -MESIWALPTS----EL----------------G--------------------------      105
XP_028942374.1      -AQSTPQPWSLSLVSFS----------------VDVGK--------SPDVPYV-------      114
PKK19633.1          -AESIWVPD--ILINEF----------------VDVGK--------SPPVPYV-------      181
XP_009894240.1      -VESIWVPD--ILINEF----------------VDVGK--------SPHVPYV-------      235
XP_032820219.1      -TNNIWVPD--IMISEF----------------VDVGK--------SPDIGYV-------      168
XP_029429480.1      -TTDIWIPD--ILINEF----------------VETET--------SPHIPYV-------      177
XP_030077572.1      -IERIWVPD--VVINEF----------------VDTKM--------TPNIPYV-------      124
XP_033774596.1      -IERIWVPD--ILINEF----------------VDSKM--------SPSIPYV-------      155
ETE72600.1          -TEAIWVPD--ILINEF----------------VDVGK--------SPEIPYV-------      170
XP_018082638.1      -TEKIWVPD--ILINEF----------------VDVGK--------SPDIPYV-------      351
XP_032905410.1      -TENIWVPD--ILINEF----------------VDVGK--------SPDIPYV-------      134
XP_020773490.1      -TANVWVPD--ILINEF----------------VDVGK--------SPDIPYV-------      143
XP_033833634.1      -TANVWVPD--ILINEF----------------VDVGK--------SPDIPYV-------      169
XP_030215795.1      -TANVWVPD--ILINEF----------------VDVGK--------SPDIPYV-------      142
XP_030602980.1      -TANVWVPD--ILINEF----------------VDVGK--------SPDIPYV-------      148
XP_004573543.1      -TANVWVPD--ILINEF----------------VDVGK--------SPDIPYV-------      142
XP_005916159.1      -TANVWVPD--ILINEF----------------VDVGK--------SPDIPYV-------      142
XP_013132089.1      -TANVWVPD--ILINEF----------------VDVGK--------SPDIPYV-------      142
XP_031603488.1      -TANVWVPD--ILINEF----------------VDVGK--------SPDIPYV-------      148
XP_005725536.1      -TANVWVPD--ILINEF----------------VDVGK--------SPDIPYV-------      148
XP_026038075.1      -TANVWVPD--ILINEF----------------VDVGK--------SPDIPYV-------      148
CAF96649.1          -TANVWVPD--ILINELLS---------PVTCSVDVGK--------SPDIPYV-------      111
XP_023818570.1      -TANVWVPD--ILINEF----------------VDVGK--------SPDIPYV-------      138
RVE64289.1          -TANVWVPD--ILINEF----------------VDVGK--------SPDIPYV-------      173
XP_024121971.1      -TANVWVPD--ILINEF----------------VDVGK--------SPDIPYV-------      144
XP_015817559.1      -TANVWVPD--ILINEF----------------VDVGK--------SPDIPYV-------      128
XP_015225670.1      -TANVWVPD--ILINEF----------------VDVGK--------SPDIPYV-------      127
XP_012722063.1      -TANVWVPD--ILINEF----------------VDVGK--------SPDIPYV-------      142
XP_032431307.1      -TANVWVPD--ILINEF----------------VDVGK--------SPDIPYV-------      142
XP_014328329.1      -TANVWVPD--ILINEF----------------VDVGK--------SPDIPYV-------      142
XP_027886578.1      -TANVWVPD--ILINEF----------------VDVGK--------SPDIPYV-------      142
XP_008426791.1      -TANVWVPD--ILINEF----------------VDVGK--------SPDIPYV-------      142
XP_014838686.1      -TANVWVPD--ILINEF----------------VDVGK--------SPDIPYV-------      142
XP_014885967.1      -TANVWVPD--ILINEF----------------VDVGK--------SPDIPYV-------      142
XP_033954312.1      -TANVWVPD--ILINEF----------------VDVGK--------SPDIPYV-------      142
XP_034089244.1      -TANVWVPD--ILINEF----------------VDVGK--------SPDIPYV-------      142
XP_010780064.1      -TANVWVPD--ILINEF----------------VDVGK--------SPDIPYV-------      158
XP_033970261.1      -TANVWVPD--ILINEF----------------VDVGK--------SPDIPYV-------      158
XP_029375575.1      -TANVWVPD--ILINEF----------------VDVGK--------SPDIPYV-------      179
KAF0023022.1        -TANVWVPD--ILINEF----------------VDVGK--------SPDIPYV-------      175
XP_019952614.1      -TANVWVPD--ILINEF----------------VDVGK--------SPDIPYV-------      131
XP_034463117.1      -TANVWVPD--ILINEF----------------VDVGK--------SPDIPYV-------      142
XP_029924656.1      -TANVWVPD--ILINEF----------------VDVGK--------SPDIPYV-------      143
XP_028323228.1      -TSNVWVPD--ILINEF----------------VDVGK--------SPDIPYV-------      142
TNM84480.1          -TANVWVPD--ILINEF----------------VDVGK--------SPDIPYV-------      142
XP_029703788.1      -TANVWVPD--ILINEF----------------VDVGK--------SPDIPYV-------      142
XP_030010368.1      -TANVWVPD--ILINEF----------------VDVGK--------SPDIPYV-------      145
XP_019725056.1      -TANVWVPD--ILINEF----------------VDVGK--------SPDIPYV-------      143
XP_034034934.1      -TANVWVPD--ILINEF----------------VDVGK--------SPNIPYV-------      131
XP_029956880.1      -TANVWVPD--ILINEF----------------VDVGK--------SPDIPYV-------      142
XP_008331307.3      -TANVWVPD--ILINEF----------------VDVGK--------SPDIPYV-------      175
XP_031724845.1      -TTNVWVPD--ILINEF----------------VDVGK--------SPDIPYV-------      143
XP_013859395.1      -TANVWVPD--ILINEF----------------VDVGK--------SPDIPYV-------      142
XP_017275335.1      -TANVWVPD--ILINEF----------------VDVGK--------SPDIPYV-------      142
XP_020496197.1      -TANVWVPD--ILINEF----------------VDVGK--------SPDIPYV-------      142
XP_029030400.1      -TANVWVPD--ILINEF----------------VDVGK--------SPDIPYV-------      142
XP_022060538.1      -TANVWVPD--ILINEF----------------VDVGK--------SPDIPYV-------      143
XP_023150584.1      -TANVWVPD--ILINEF----------------VDVGK--------SPDIPYV-------      143
XP_029303892.1      -TANVWVPD--ILINEF----------------VDVGK--------SPDIPYV-------      166
XP_020454172.1      -TANVWVPD--ILINEF----------------VDVGK--------SPDIPYV-------      141
TKS83244.1          -TANVWVPD--ILINEF----------------VDVGK--------SPDIPYV-------      129
XP_022616583.1      -TANVWVPD--ILINEF----------------VDVGK--------SPDIPYV-------      175
XP_030293172.1      -TANVWVPD--ILINEF----------------VDVGK--------SPDIPYV-------      142
XP_027129539.1      -TANVWVPD--ILINEF----------------VDVGK--------SPDIPYV-------      142
XP_034539538.1      -TANVWVPD--ILINEF----------------VDVGK--------SPDIPYV-------      142
XP_008277330.1      -TANVWVPD--ILINEF----------------VDVGK--------SPDIPYV-------      142
XP_028276940.1      -TANVWVPD--ILINEF----------------VDVGK--------SPDIPYV-------      142
XP_023263433.1      -TANVWVPD--ILINEF----------------VDVGK--------SPDIPYV-------      142
XP_034406336.1      -TTNVWVPD--ILINEF----------------VDVGK--------SPDIPYV-------      142
XP_026184460.1      -TANVWVPD--ILINEF----------------VDVGK--------SPDIPYV-------      142
KAF1378228.1        -TNNVWVPD--ILINEF----------------VDVGK--------SPDIPYV-------      127
XP_028450365.1      -TNNVWVPD--ILINEF----------------VDVGK--------SPDIPYV-------      142
XP_031163851.1      -TNNVWVPD--ILINEF----------------VDVGK--------SPDIPYV-------      142
XP_032389084.1      -TNNVWVPD--ILINEF----------------VDVGK--------SPDIPYV-------      142
XP_033494682.1      -TANVWVPD--ILINEF----------------VDVGK--------SPDIPYV-------      142
XP_018544782.1      -TANVWVPD--ILINEF----------------VDVGK--------SPDIPYV-------      142
```

```
XP_026228189.1      -TANVWVPD--ILINEF----------------VDVGK--------SPDIPYV-------     142
XP_028976606.1      -TANVWVPD--ILINEF----------------VDVGK--------SPDIPYV-------     142
CAB1352378.1        -TANVWVPD--ILINEF----------------VDVGK--------SPDIPYV-------     142
XP_023866849.1      -TANVWVPD--ILINEF----------------VDVGK--------SPDIPYV-------     142
XP_013992832.1      -TANVWVPD--ILINEF----------------VDVGK--------SPDIPYV-------     142
XP_029546688.1      -TANVWVPD--ILINEF----------------VDVGK--------SPDIPYV-------     142
XP_021481546.1      -TANVWVPD--ILINEF----------------VDVGK--------SPDIPYV-------     142
XP_020339889.1      -TANVWVPD--ILINEF----------------VDVGK--------SPDIPYV-------     142
XP_029481972.1      -TANVWVPD--ILINEF----------------VDVGK--------SPDIPYV-------     142
KPP68743.1          -TTNVWVPD--ILINEF----------------VDVGK--------SPDIPYV-------     119
XP_023669383.1      -TTNIWVPD--ILINEF----------------VDVGK--------SPDIPYV-------     141
KAA0720096.1        -TANVWVPD--ILINEF----------------VDVGK--------SPDIPYV-------     47
XP_009293684.1      -TANIWIPD--ILINEF----------------VDVGK--------SPDIPYV-------     141
XP_026090784.1      -TANIWVPD--ILINEF----------------VDVGK--------SPDIPYV-------     141
XP_018918715.1      -TANIWVPD--ILINEF----------------VDVGK--------SPEIPYV-------     141
KAF4098549.1        -TANIWVPD--ILINEF----------------VDVGK--------SPEIPYV-------     141
XP_016084173.1      -TANIWVPD--ILINEF----------------VDVGK--------SPEIPYV-------     141
XP_016332759.1      -TANIWVPD--ILINEF----------------VDVGK--------SPEIPYV-------     141
XP_016398039.1      -TANIWVPD--ILINEF----------------VDVGK--------SPEIPYV-------     141
XP_026989970.1      -SANIWVPD--ILINEF----------------VDVGK--------SPDIPYV-------     141
TSQ12698.1          -SANIWVPD--ILINEF----------------VDVGK--------SPDIPYV-------     141
XP_017347546.1      -TANVWVPD--ILINEF----------------VDVGK--------SPDIPYV-------     136
XP_026794616.2      -TANVWVPD--ILINEF----------------VDVGK--------SPDIPYV-------     141
XP_026861889.1      -TANVWVPD--IVINEF----------------VDVGK--------SPDIPYV-------     141
XP_017575347.1      -TANVWVPD--IVINEF----------------VDVGK--------SPDIPYV-------     141
XP_022531596.1      -TANVWVPD--IVINEF----------------VDVGK--------SPDIPYV-------     141
XP_012691019.2      -TANVWVPD--IVINEF----------------VDVGK--------SPDIPYV-------     124
XP_028839723.1      -TANVWVPD--IVINEF----------------VDVGK--------SPDIPYV-------     141
XP_030638860.1      -TANVWVPD--ILINEF----------------VDVGK--------SPDIPYV-------     123
XP_007882964.1      -TEKIWVPD--IMINEF----------------VELGK--------SPDIPYV-------     117
GCB70244.1          -TANIWVPD--ILINEF----------------VDVGK--------SPEIPYV-------     118
GCC26242.1          -TANVWMPD--ILINEF----------------VDVGK--------SPDIPYV-------     139
XP_020387034.1      -TANIWVPD--ILINEF----------------VDVGK--------SPDIPYV-------     130
XP_028931940.1      -TNCIWIPD--ILINEF----------------VDVGK--------SPDIPYV-------     147
XP_031762490.1      -TEKIWVPD--ILINEF----------------VDVGK--------SPDIPYV-------     136
PIO32240.1          ------------------------------------------------------------     0
XP_018425466.1      -TEKIWVPD--ILINEF----------------VDVGK--------SPDIPYV-------     137
XP_006642351.1      -TSNIWVPD--ILINEF----------------VDVGK--------SPDIPYV-------     158
XP_028665372.1      -TTNVWVPD--ILINEF----------------VDVGK--------SPDIPYV-------     141
GCF49814.1          -TQAIWVPD--ILINEF----------------VDVGK--------SPDIPYV-------     120
XP_015277816.1      -TEAIWVPD--ILINEF----------------VDVGK--------SPDIPYV-------     143
XP_005987340.2      -TESIWVPD--ILINEF----------------VDVGK--------SPNIPYV-------     125
XP_019339534.1      -TESIWVPD--ILINEF----------------VDVGK--------SPDIPYV-------     142
XP_025063414.1      -TESIWVPD--ILINEF----------------VDVGK--------SPDIPYV-------     142
XP_019367488.1      -TESIWVPD--ILINEF----------------VDVGK--------SPDIPYV-------     142
XP_019412078.1      -TESIWVPD--ILINEF----------------VDVGK--------SPDIPYV-------     142
XP_014434314.1      -TESIWVPD--ILINEF----------------VDMGK--------SPDIPYV-------     142
XP_007060556.1      -TESIWVPD--ILINEF----------------VDVGK--------SPDIPYV-------     136
XP_024072175.1      -TDSIWVPD--ILINEF----------------VDVGK--------SPDIPYV-------     121
XP_008170567.1      -TDSIWVPD--ILINEF----------------VDVGK--------SPDIPYV-------     142
XP_034610543.1      -TDSIWVPD--ILINEF----------------VDVGK--------SPDIPYV-------     142
XP_030394127.1      -TQSIWVPD--ILINEF----------------VDVGK--------SPDIPYV-------     142
XP_032651960.1      -TQSIWVPD--ILINEF----------------VDVGK--------SPDIPYV-------     142
XP_025020083.1      -TEAIWVPD--ILINEF----------------VDVGK--------SPEIPYV-------     142
XP_026536833.1      -TEAIWVPD--ILINEF----------------VDVGK--------SPEIPYV-------     142
XP_026564787.1      -TEAIWIPD--ILINEF----------------VDVGK--------SPEIPYV-------     142
XP_032084675.1      -TEAIWVPD--ILINEF----------------VDVGK--------SPEIPYV-------     127
XP_029139341.1      -TEAIWVPD--ILINEF----------------VDVGK--------SPEIPYV-------     142
XP_034281006.1      -TEAIWIPD--ILINEF----------------VDVGK--------SPEIPYV-------     142
XP_020649062.1      -TDAIWVPD--ILINEF----------------VDVGK--------SPDIPYV-------     142
XP_008117087.1      -TDAIWVPD--ILINEF----------------VDVGK--------SPDIPYV-------     142
XP_028564324.1      -TEAIWVPD--ILINEF----------------VDVGK--------SPDIPYV-------     148
XP_033028155.1      -TEAIWVPD--ILINEF----------------VDVGK--------SPDIPYV-------     147
XP_025963249.1      -TESIWVPD--ILISEF----------------VDVGK--------SPDIPYV-------     129
XP_009668348.1      -TESIWVPD--ILINEF----------------VDVGK--------SPDIPYV-------     141
XP_013798935.1      -TESIWVPD--ININEF----------------VDVGK--------SPNIPYV-------     125
XP_025913685.1      -TESIWVPD--ININEF----------------VDVGK--------SPNIPYV-------     150
XP_013042552.1      -AESIWVPD--ILINEF----------------VDVGK--------SPDVPYV-------     148
XP_005030458.2      -AESIWVPD--ILINEF----------------VDVGK--------SPDVPYV-------     141
XP_032057953.1      -AESIWVPD--ILINEF----------------VDVGK--------SPDVPYV-------     124
XP_021232050.1      -TESVWVPD--ILINEF----------------VDVGK--------SPDVPYV-------     143
OXB62403.1          -AESIWVPD--ILINEF----------------VDVGK--------SPDVPYV-------     142
OXB81319.1          -AESIWVPD--ILINEF----------------VDVGK--------SPDVPYV-------     124
XP_010722007.1      -AENIWVPD--ILINEF----------------VDVGK--------SPDVPYV-------     143
XP_015739349.1      -AESIWVPD--ILINEF----------------VDVGK--------SPDVPYV-------     143
XP_031455498.1      -AESIWVPD--ILINEF----------------VDVGK--------SPDVPYV-------     144
POI27435.1          -AESIWVPD--ILINEF----------------VDVGK--------SPDVPYV-------     119
```

```
XP_004948120.1      -AESIWVPD--ILINEF----------------VDVGK--------SPDVPYV-------      144
XP_032851190.1      -VESIWVPD--ILINEF----------------VDVGK--------SPHVPYV-------      139
XP_010007255.1      -AESIWVPD--ILINEF----------------VDVGR--------SPRVPYV-------      141
XP_030320702.1      -AESIWVPD--ILINEF----------------VDVGR--------SPRVPYV-------      112
XP_010191940.1      -AESIWVPD--ILINEF----------------VDVGR--------SPHVPYV-------      139
XP_027737112.1      -MESIWVPD--ILINEF----------------VDVGK--------SPHVPYV-------      137
XP_027555032.1      -MESIWVPD--ILINEF----------------VDVGK--------SPHVPYV-------      142
XP_032565370.1      -VESIWVPD--ILINEF----------------VDVGK--------SPHVPYV-------      188
XP_027511217.1      -VESIWVPD--ILINEF----------------VDVGK--------SPHVPYV-------      287
XP_027593499.1      -VESIWVPD--ILINEF----------------VDVGK--------SPHVPYV-------      238
XP_017664924.1      -VESIWVPD--ILINEF----------------VDVGK--------SPHVPYV-------      139
XP_029817938.1      -VESIWVPD--ILINEF----------------VDVGK--------SPHVPYV-------      121
XP_005058641.1      -VESIWVPD--ILINEF----------------VDVGK--------SPHVPYV-------      172
XP_021385804.1      -VESIWVPD--ILINEF----------------VDVGK--------SPHVPYV-------      141
XP_030146687.2      -VESIWVPD--ILINEF----------------VDVGK--------SPHVPYV-------      186
KAF4796420.1        -VESIWVPD--ILINEF----------------VDVGK--------SPHVPYV-------      141
XP_032937581.1      -VESIWVPD--ILINEF----------------VDVGK--------SPHIPYV-------      188
XP_031989659.1      -VESIWVPD--ILINEF----------------VDVGK--------SPHVPYV-------      142
XP_010402086.1      -VESIWVPD--ILINEF----------------VDVGK--------SPHVPYV-------      141
XP_017594069.1      -VESIWVPD--ILINEF----------------VDVGK--------SPHVPYV-------      112
XP_014115268.1      -VESIWVPD--ILINEF----------------VDVGK--------SPHVPYV-------      166
XP_023797108.1      -VESIWVPD--ILINEF----------------VDVGK--------SPHVPYV-------      112
XP_033375614.1      -VESIWVPD--ILINEF----------------VDVGK--------SPHVPYV-------      112
XP_014740121.1      -VESIWVPD--IHINEF----------------VDVGK--------SPHVPYV-------      286
RLV83430.1          -VESIWVPD--ILINEF----------------VDVGK--------SPHVPYV-------      132
XP_009096098.2      -VESIWVPD--ILINEF----------------VDVGK--------SPHVPYV-------      247
TRZ15870.1          -VESIWVPD--ILINEF----------------VDVGK--------SPHVPYV-------      142
RMB91935.1          -VESIWVPD--ILINEF----------------VDVGK--------SPHVPYV-------      112
XP_030820843.1      -VESIWVPD--ILINEF----------------VDVGK--------SPHVPYV-------      188
XP_014165179.1      -VESIWVPD--ILINEF----------------VDVGK--------SPHVPYV-------      190
XP_026653582.1      -VESIWVPD--ILINEF----------------VDVGK--------SPHVPYV-------      72
PKU35975.1          -VESIWVPD--ILINEF----------------VDVGK--------SPHVPYV-------      144
XP_014805072.1      -VESIWVPD--ILINEF----------------VDVGK--------SPHVPYV-------      138
XP_009818330.1      ------------------------------------------------------------      0
OPJ68307.1          -AESIWVPD--ILINEF----------------VDVGK--------SPPVPYV-------      141
XP_008936289.1      -VESIWVPD--ILINEF----------------VDVGK--------SPHVPYV-------      142
XP_010287046.1      -VESIWVPD--ILINEF----------------VDVGK--------SPHVPYV-------      64
XP_005240140.2      -MESIWVPD--ILINEF----------------VDVGK--------SPHVPYV-------      201
XP_005437752.2      -MESIWVPD--ILINEF----------------VDVGK--------SPHVPYV-------      201
KFV74811.1          -VESIWVPD--ILINEF----------------VDVGK--------SPHVPYV-------      106
XP_010018389.1      -VESIWVPD--ILINEF----------------VDVGK--------SPHVPYV-------      149
KQK78711.1          -MESIWVPD--ILINEF----------------VDVGK--------SPHVPYV-------      141
XP_009570162.1      -MENIWVPD--ILINEF----------------VDVGK--------SPHVPYV-------      140
KFP11268.1          -VESIWVPD--ILINEF----------------VDVGK--------SPHVPYV-------      111
KFQ98910.1          -AESIWVPD--ILINEF----------------VDVGK--------SPHVPYV-------      125
XP_012985202.3      -VESIWVPD--ILINEF----------------VDVGK--------SPHVPYV-------      141
XP_010573388.1      -AESIWVPD--ILINEF----------------VDVGK--------SPHVPYV-------      140
XP_029879496.1      -AESIWVPD--ILINEF----------------VDVGK--------SPHVPYV-------      112
XP_030361086.1      -MESIWVPD--ILINEF----------------VDVGK--------SPHVPYV-------      193
KFM00668.1          -VESIWVPD--ILINEF----------------VDVGK--------SPHVPYV-------      125
KAF1479074.1        -VESIWVPD--ILINEF----------------VDVGK--------SPHVPYV-------      104
KAF1651161.1        -VESIWVPD--ILINEF----------------VDVGK--------SPHVPYV-------      105
KAF1673648.1        -MESIWVPD--ILINEF----------------VDVGK--------SPHVPYV-------      105
KAF1493319.1        -VESIWVPD--ILINEF----------------VDVGK--------SPHVPYV-------      105
KAF1584157.1        -VESIWVPD--ILINEF----------------VDVGK--------SPHVPYV-------      105
KAF1571723.1        -VESIWVPD--ILINEF----------------VDVGK--------SPHVPYI-------      105
KAF1533169.1        -VESIWVPD--ILINEF----------------VDVGK--------SPHVPYV-------      105
KAF1638955.1        -VESIWVPD--ILINEF----------------VDVGK--------SPHVPYV-------      105
KAF1549972.1        -VESIWVPD--ILINEF----------------VDVGK--------SPHVPYV-------      105
KAF1606914.1        -VESIWVPD--ILINEF----------------VDVGK--------SPHVPYV-------      105
KAF1510957.1        -VESIWVPD--ILINEF----------------VDVGK--------SPHVPYV-------      105
KAF1498899.1        -VESIWVPD--ILINEF----------------VDVGK--------SPHVPYV-------      105
KAF1411525.1        -VESIWVPD--ILINEF----------------VDVGK--------SPHVPYV-------      105
KAF1429205.1        -VESIWVPD--ILINEF----------------VDVGK--------SPHVPYV-------      105
XP_005334318.1      -LSAIWAPD--IIINEF----------------VDIER--------SPDIPYV-------      131
XP_014395552.1      -LSAIWAPD--IIINEF----------------VDIER--------LPELHYV-------      131
KAB0404854.1        -TDSIWVPD--ILINEF----------------VDVGK--------SPNIPYV-------      127
XP_007494828.1      -TDYIWVPD--ILINEF----------------VDVGK--------SPNIPYV-------      142
XP_003764254.1      -TDYIWVPD--ILINEF----------------VDVGK--------SPNIPYV-------      142
XP_020845489.1      -TDYIWVPD--ILINEF----------------VDVGK--------SPNIPYV-------      142
XP_027703160.1      -TDYIWVPD--ILINEF----------------VDVGK--------SPNIPYV-------      142
XP_017523929.1      -TDSIWVPD--ILINEF----------------VDVGK--------SPNIPYV-------      140
XP_008688428.1      -TESIWVPD--ILINEL----------------A--------------------------      131
XP_029812166.1      -TERIWVPD--ILINEF----------------VDVGK--------SPSIPYV-------      159
XP_025749781.1      -TESIWVPD--ILINEF----------------VDVGK--------SPSIPYV-------      142
XP_004416432.1      -TESIWVPD--ILINEF----------------VDVGK--------SPSIPYV-------      142
XP_027436262.1      -TESIWVPD--ILINEF----------------VDVGK--------SPSIPYV-------      142
```

XP_032284025.1	-TESIWVPD--ILINEF----------------VDVGK--------SPSIPYV-------	142
XP_006735421.1	-TESIWVPD--ILINEF----------------VDVGK--------SPSIPYV-------	142
XP_021552166.1	-TESIWVPD--ILINEF----------------VDVGK--------SPSIPYV-------	142
XP_026361066.1	-TESIWVPD--ILINEF----------------VDVGK--------SPSIPYV-------	142
XP_034523598.1	-TESIWVPD--ILINEF----------------VDVGK--------SPSIPYV-------	142
NP_001297113.1	-TDNIWVPD--ILINEF----------------VDVGK--------SPSIPYV-------	142
XP_032215488.1	-TDNIWVPD--ILINEF----------------VDVGK--------SPSIPYV-------	142
VCX31483.1	-TDNIWVPD--ILINEF----------------VDVGK--------SPSIPYV-------	142
XP_022369003.1	-TDNIWVPD--ILINEF----------------VDVGK--------SPSIPYV-------	142
XP_032694248.1	-TDNIWVPD--ILINEF----------------VDVGK--------SPSIPYV-------	142
NP_001041584.1	-TESIWVPD--ILINEF----------------VDVGK--------SPSIPYV-------	142
XP_025862501.1	-TESIWVPD--ILINEF----------------VDVGK--------SPSIPYV-------	142
KAF0873564.1	-TESIWVPD--ILINEF----------------VDVGK--------SPSIPYV-------	284
XP_025784751.1	-TESIWVPD--ILINEF----------------VDVGK--------SPSIPYV-------	142
XP_007075625.1	-TESIWVPD--ILINEF----------------VDVGK--------SPSIPYV-------	278
XP_030189489.1	-TESIWVPD--ILINEF----------------VDVGK--------SPSIPYV-------	142
XP_019324655.1	-TESIWVPD--ILINEF----------------VDVGK--------SPSIPYV-------	243
XP_023094886.1	-TESIWVPD--ILINEF----------------VDVGK--------SPSIPYV-------	278
XP_026892357.1	-TESIWVPD--ILINEF----------------VDVGK--------SPSIPYV-------	142
XP_016004457.1	-KDSIWVPD--ILINEF----------------VDVGK--------SPDMPYV-------	137
XP_006912860.1	-KDSIWVPD--ILINEF----------------VDVGK--------SPDMPYV-------	137
XP_011363679.1	-KDSIWVPD--ILINEF----------------VDVGK--------SPDMPYV-------	137
XP_016076060.1	-TDSIWVPD--ILINEF----------------VDVGK--------SPDIPYV-------	142
XP_008148371.1	-TDSIWVPD--ILINEF----------------VDVGK--------SPDIPYV-------	142
XP_006093568.1	-TDSIWVPD--ILINEF----------------VDVGK--------SPDIPYV-------	142
XP_006761601.1	-TDSIWVPD--ILINEF----------------VDVGK--------SPDIPYV-------	142
XP_024426743.1	-TDSIWVPD--ILINEF----------------VDVGK--------SPDIPYV-------	137
XP_019520779.1	-TDSIWVPD--ILINEF----------------VDVGK--------SPDIPYV-------	142
XP_032976539.1	-TDSIWVPD--ILINEF----------------VDVGK--------SPDIPYV-------	142
ELW64270.1	-TDSIWVPD--ILINEF----------------VDVGK--------SPNIPYV-------	146
XP_004427367.1	-TDSIWVPD--ILINEF----------------VDVGK--------SPNIPYV-------	142
XP_008529353.1	-TDSIWVPD--ILINEF----------------VDVGK--------SPNIPYV-------	142
NP_001288165.1	-TDSIWVPD--ILINEF----------------VDVGK--------SPNIPYV-------	142
XP_014711213.1	-TDSIWVPD--ILINEF----------------VDVGK--------SPNIPYV-------	142
XP_012514621.1	-TDSVWVPD--ILINEF----------------VDVGK--------SPNIPYV-------	142
XP_008071525.1	-TDSIWVPD--ILINEF----------------VDVGK--------SPDIPYV-------	140
XP_009005082.1	-TDSIWVPD--ILINEF----------------VDVGK--------SPNIPYV-------	142
XP_010332832.1	-TDSIWVPD--ILINEF----------------VDVGK--------SPNIPYV-------	137
XP_012326058.1	-TDSIWVPD--ILINEF----------------VDVGK--------SPNIPYV-------	148
XP_017378796.1	-TESIWVPD--ILINEF----------------VDVGK--------SPNIPYV-------	171
XP_032141374.1	-TESIWVPD--ILINEF----------------VDVGK--------SPNIPYV-------	171
XP_032024018.1	-TDSIWVPD--ILINEF----------------VDVGK--------SPNIPYV-------	137
XP_018891497.2	-TDSIWVPD--ILINEF----------------VDVGK--------SPNIPYV-------	143
XP_030684894.1	-TDSIWVPD--ILINEF----------------VDVGK--------SPNIPYV-------	137
PNJ75904.1	-TDSIWVPD--ILINEF----------------VDVGK--------SPNIPYV-------	143
AAP35868.1	-TDSIWVPD--ILINEF----------------VDVGK--------SPNIPYV-------	143
XP_001149570.1	-TDSIWVPD--ILINEF----------------VDVGK--------SPNIPYV-------	143
XP_003805532.1	-TDSIWVPD--ILINEF----------------VDVGK--------SPNIPYV-------	143
XP_023063823.1	-TDSIWVPD--ILINEF----------------VDVGK--------SPNIPYV-------	142
XP_033060210.1	-TDSIWVPD--ILINEF----------------VDVGK--------SPNIPYV-------	142
XP_010370669.1	-TDSIWVPD--ILINEF----------------VDVGK--------SPNIPYV-------	142
XP_017738496.1	-TDSIWVPD--ILINEF----------------VDVGK--------SPNIPYV-------	142
XP_011782168.1	-TDSIWVPD--ILINEF----------------VDVGK--------SPNIPYV-------	142
EHH23440.1	-TDSIWVPD--ILINEF----------------VDVGK--------SPNIPYV-------	137
XP_003910778.3	-TDSIWVPD--ILINEF----------------VDVGK--------SPNIPYV-------	142
XP_025212649.1	-TDSIWVPD--ILINEF----------------VDVGK--------SPNIPYV-------	148
XP_008019132.1	-TDSIWVPD--ILINEF----------------VDVGK--------SPNIPYV-------	142
XP_011832421.1	-TDSIWVPD--ILINEF----------------VDVGK--------SPNIPYV-------	127
XP_011759618.1	-TDSIWVPD--ILINEF----------------VDVGK--------SPNIPYV-------	142
XP_005579749.1	-TDSIWVPD--ILINEF----------------VDVGK--------SPNIPYV-------	142
XP_011921684.1	--	0
XP_008259391.1	-TDSIWIPD--ILINEF----------------VDVGK--------SPNIPYV-------	142
XP_012782438.1	-TDSVWVPD--ILINEF----------------VDVGK--------SPYIPYV-------	138
VTJ86076.1	-TDSVWVPD--ILINEF----------------VDVGK--------SPNIPYV-------	148
XP_015345533.1	-TDSVWVPD--ILINEF----------------VDVGK--------SPNIPYV-------	148
XP_027786432.1	-TDSVWVPD--ILINEF----------------VDVGK--------SPNIPYV-------	148
XP_026248345.1	-TDSVWVPD--ILINEF----------------VDVGK--------SPNIPYV-------	142
XP_005378209.1	-TDSIWVPD--ILINEF----------------VDVGK--------SPSIPYV-------	142
XP_004466010.1	-TDSIWVPD--ILINEF----------------VDVGK--------SPNIPYV-------	142
XP_020035707.1	-TDSIWVPD--ILINEF----------------VDVGK--------SPSIPYV-------	137
XP_006181802.1	-TDSIWVPD--ILINEF----------------VDVGK--------SPYIPYV-------	142
XP_006207862.1	-TDSIWVPD--ILINEF----------------VDVGK--------SPYIPYV-------	142
XP_005891968.1	-TDSIWVPD--ILINEF----------------VDVGK--------SPNIPYV-------	139
XP_010828662.1	-TDSIWVPD--ILINEF----------------VDVGK--------SPNIPYV-------	139
XP_019830126.1	-TDSIWVPD--ILINEF----------------VDVGK--------SPNIPYV-------	138
XP_017914486.1	-TDSIWVPD--ILINEF----------------VDVGK--------SPNIPYV-------	138
XP_006060266.2	-TDSIWVPD--ILINEF----------------VDVGK--------SPNIPYV-------	143

```
XP_020728447.1      -TDSIWVPD--ILINEF----------------VDVGK--------SPNIPYV-------    143
KAF4008892.1        -TDSIWVPD--ILINEF----------------VDVGK--------SPNIPYV-------    143
KAB0348059.1        -TDSIWVPD--ILINEF----------------VDVGK--------SPNIPYV-------    126
KAB0371113.1        -TDSIWVPD--ILINEF----------------VDVGK--------SPNIPYV-------    119
MBV96963.1          -TDSIWVPD--ILINEF----------------VDVGK--------SPNIPYV-------    142
XP_007172048.1      -TDSIWVPD--ILINEF----------------VDVGK--------SPNIPYV-------    142
XP_007452270.1      -TDSIWVPD--ILINEF----------------VDVGK--------SPNIPYV-------    142
XP_023987108.1      -TDSIWVPD--ILINEF----------------VDVGK--------SPNIPYV-------    137
XP_004273432.1      -TDSIWVPD--ILINEF----------------VDVGK--------SPNIPYV-------    137
XP_004328646.1      -TDSIWVPD--ILINEF----------------VDVGK--------SPNIPYV-------    137
XP_026937670.1      -TDSIWVPD--ILINEF----------------VDVGK--------SPNIPYV-------    137
XP_030691031.1      -TDSIWVPD--ILINEF----------------VDVGK--------SPNIPYV-------    137
XP_022414870.1      -TDSIWVPD--ILINEF----------------VDVGK--------SPNIPYV-------    142
XP_029064651.1      -TDSIWVPD--ILINEF----------------VDVGK--------SPNIPYV-------    142
XP_024607566.1      -TDSIWVPD--ILINEF----------------VDVGK--------SPNIPYV-------    142
XP_032496907.1      -TDSIWVPD--ILINEF----------------VDVGK--------SPNIPYV-------    142
XP_007535559.1      -TESIWVPD--ILINEF----------------VDVGK--------SPEIPYV-------    135
XP_031299536.1      TDTSGSSSD--ILINEF----------------VDVGK--------SPYIPYV-------    141
XP_004666516.1      -TDSIWVPD--ILINEF----------------VDVGK--------SPSIPYV-------    137
XP_008830963.1      -TDSIWVPD--ILINEF----------------VDVGK--------SPSIPYV-------    142
XP_005347338.1      -TDSVWVPD--ILINEF----------------VDVGK--------SPNIPYV-------    142
XP_005069548.1      -TDSIWVPD--ILINEF----------------VDVGK--------SPNIPYV-------    142
XP_027267894.1      -TDSIWVPD--ILINEF----------------VDVGK--------SPNIPYV-------    142
OBS80992.1          -TDSIWVPD--ILINEF----------------VDVGK--------SPSIPYV-------    129
XP_006979812.1      -TDSIWVPD--ILINEF----------------VDVGK--------SPSIPYV-------    148
XP_028720687.1      -TDSIWVPD--ILINEF----------------VDVGK--------SPSIPYV-------    142
XP_021489005.1      -TDSIWVPD--ILINEF----------------VDVGK--------SPSIPYV-------    143
XP_031199589.1      -TDSIWLPD--ILINEF----------------VDVGK--------SPSIPYV-------    167
XP_028617944.1      ------------------------------------------------------------    0
XP_034347030.1      -TDSIWVPD--ILINEF----------------VDVGK--------SPSIPYV-------    140
NP_077370.2         -TDSIWVPD--ILINEF----------------VDVGK--------SPSIPYV-------    142
XP_032766961.1      -TDSIWVPD--ILINEF----------------VDVGK--------SPSIPYV-------    142
XP_021063186.1      -TDSIWVPD--ILINEF----------------VDVGK--------SPSIPYV-------    125
NP_001093114.1      -TDSIWVPD--ILINEF----------------VDVGK--------SPNIPYV-------    142
XP_021028379.1      -TDSIWVPD--ILINEF----------------VDVGK--------SPSIPYV-------    142
XP_004689305.1      -TDSIWVPD--ILINEF----------------VDVGK--------SPEIPYV-------    142
XP_004712871.1      -TDSIWVPD--ILINEF----------------VDVGK--------SPYIPYV-------    142
XP_004382575.1      -TDSIWVPD--ILINEF----------------VDVGK--------SPNIPYV-------    142
XP_010596188.1      -TESIWVPD--ILINEF----------------VDVGK--------SPNIPYV-------    142
XP_006890855.1      -TDSIWVPD--ILINEF----------------VDVGK--------SPNIPYV-------    142
XP_006834038.1      -TDSIWVPD--ILINEF----------------VDVGK--------SPNIPYV-------    142
XP_007934716.1      -TDSIWVPD--ILINEF----------------VDVGK--------SPNIPYV-------    142
XP_012603491.1      -TDSIWVPD--ILINEF----------------VDVGK--------SPNIPYV-------    142
XP_012663606.1      -TDSIWVPD--ILINEF----------------VDVGK--------SPNIPYV-------    137
XP_028372812.1      -TDSIWVPD--ILINEF----------------VDVGK--------SPDIPYV-------    142
XP_008589608.1      -TDSIWVPD--ILINEF----------------VDVGK--------SPDIPYV-------    142
XP_012865077.1      -TDSIWVPD--ILINEF----------------VDMGK--------SPSIPYV-------    142
NP_001166178.1      -TDSIWVPD--ILINEF----------------VDVGK--------SPNIPYV-------    142
XP_004636553.1      -TESIWVPD--ILINEF----------------VDVGK--------SPSIPYV-------    142
XP_004856670.1      -TDSIWVPD--ILINEF----------------VDVGK--------SPSIPYV-------    142
XP_010627744.1      -TDSIWVPD--ILINEF----------------VDVEK--------SPSIPYV-------    142

                          Loop-E           Cys-loop          Loop-B

NP_509270.1         -------VRYDGRVEVDFNKLVDLTCPMSVLSFPFDVQLCA-------------------    174
VDO93178.1          LSIDSSNETNKVFVTFRYPAIYKFVCNMNIFYYPFDVQKCR-------------------    190
PAV91580.1          -----WEGRNGSDLSFLYPAIYTVTCRLNIRFFPYDRQNCT-------------------    183
VDK46997.1          -----WKGENGSQMSFLYPAIYTITCRLNIRFFPYDRQNCT-------------------    149
VIO86814.1          ------EISANGRLRVFIPITVRTLCPINVKHFPFDIQNCT-------------------    208
VDP38785.1          ------RISQKGEVTWNLPVSIRSSCNVDVLYFPFDHQLCN-------------------    228
KOF68401.1          ------RISSNGTMYYRFPSVVKSSCMLQTKNFPFDKQFCN-------------------    149
CDJ96026.1          ------MVTSNGNVTWLFSALFRSSCPIRVRYYPFDDQECD-------------------    298
EFX76216.1          ------IVTHDGNLTWLSSAIFKSSCGINVEYFPFDEQKCS-------------------    140
KAF7403848.1        ------IVSHTGEVVWLSHGIFRSSCDIDVEFFPFDEQRCA-------------------    152
KAF7427032.1        ------IVSHTGEVVWLSHGIFRSSCDIDVEFFPFDEQRCA-------------------    167
KAE9417558.1        ------VVSSNGDVLLAPPGIVRVSCELSMTWFPFDEQICF-------------------    151
KJH51504.1          ------LNYHDGVINWVPPGIFKISCKLDIYWFPFDEQICY-------------------    157
VDL69795.1          ------LVYPDGMVNWMPPGIFRISCKIAVAWFPFDIQQCF-------------------    147
VDO32671.1          ------LVYSSGDVNWIPPGVLKFVCKLDVTWFPFDDQICE-------------------    164
VBB32409.1          ------IVYNTGDISWIPPAIFKISCKINIEWFPFDEQRCF-------------------    147
VDN54565.1          ------LVYNTGDISWIPPGIFKISCKIDIRWFPFDEQRCF-------------------    166
EGT55171.1          ------IVYSSGLVHWVPPGIFKISCKIDIQWFPFDEQKCF-------------------    177
RCN52111.1          ------VVYSDGKVHWVPPGIFKISCKINIEWFPFDEQQCKFKVKEPNLFGPWENFHGDL    182
RMX54856.1          ------VINSTGWNMWLTPVILYSSCKMDVKYFPFDTQACD-------------------    178
XP_022781674.1      ------VINSTGWNMWLTPVILYSSCKMDVKYFPFDAQACD-------------------    440
RNA37099.1          ------MIYNNGKVFWPVPTKLQSTCKFDVTFFPFDIQRCF-------------------    182
```

```
KAA0187152.1      ------MVSHNGKVFWPPPTKFRSTCPVDVTYFPFDDQTCI-------------------   309
GAU96593.1        ------MVHSDGNVFWPPPTKLRSTCKVEITFFPFDSQTCT-------------------   180
XP_009043980.1    ------MVSYDGNVFWAPPAKFRSSCKIDITYFPFDDQRCE-------------------   141
VDM43573.1        ------MVFYDGTVFWPPPTQLRSTCKIDVTYFPFDSQHCA-------------------   259
XP_003140283.1    ------ILFYDGTVFWPPPTQLRSTCKTDVTYFPFDSQHCT-------------------   150
VDN82010.1        ------ILFYDGTVFWPPPTQLRSTCKTDVTYFPFDSQHCS-------------------   176
VDO31501.1        ------ILFYDGTVFWPPPTQLRSTCKTDVTYFPFDSQHCS-------------------   178
VDP19591.1        ------IIKHDGTVQWMPPGLFKSTCNIDILWFPFDEQSCI-------------------   161
EDO32053.1        ------IVNHDGSTTWLAPAIIKSECKIDVRFFPFDVQSCE-------------------   143
TRY67230.1        ------VVYNNGDCVFIPPGLFQSTCKINILWFPFDDQECV-------------------   157
KAF7391312.1      ------VVAHNGSCLYVPPGIFKSTCKIDITWFPFDDQHCD-------------------   168
GFG30449.1        ------VVRNNGSCLYVPPGIFKSTCKIDITWFPFDDQRCE-------------------   178
KQS30083.1        ------VVRNNGSCLYVPPGIFKSTCKIDITWFPFDDQRCE-------------------   200
EDW57583.2        ------VVRNNGSCLYVPPGIFKSTCKIDITWFPFDDQRCE-------------------   336
KNC22799.1        ------VVRNNGSCLYVPPGIFKSTCKIDITWFPFDDQRCE-------------------   146
RZF44856.1        ------VVRNNGSCLYVPPGIFKSTCKIDITWFPFDDQRCE-------------------   166
ENN76856.1        ------VVRNNGSCFYLPPGIFKSTCKIDITWFPFDDQRCE-------------------   172
KAF5300392.1      ------VVRNNGSCLYVPPGIFKSTCKIDITWFPFDDQRCE-------------------   176
CAB3239999.1      ------VVRNNGSCLYVPPGIFKSTCKIDITWFPFDDQRCE-------------------   163
PCG77624.1        ------VVRNNGSCLYVPPGIFKSTCKIDITWFPFDDQRCE-------------------   106
PZC79131.1        ------VVRNNGSCLYVPPGIFKSTCKIDITWFPFDDQRCE-------------------   173
KAF4083067.1      ------YVNHTGHIRYDRMLRLVTACNLQIFSFPFDVQNCT-------------------   146
XP_009924659.1    ------AITHNGSFKSTQPFQVTLTCSLMIFKFPFDTQTCN-------------------   169
XP_010123544.1    ------AVMHNGSFNATQPFQVTLTCSLIILKFPFDTQTCN-------------------   167
CBN81618.1        ------HITANGFVESINNQVIISTCRMQVYKFPFDIQSCN-------------------   166
KAF3704230.1      ------TINDKGDVEVQNDQVLVSTCRMHTYNFPFDIQSCN-------------------   161
RUS86578.1        ------LVKFNGHVNWYPGSLFPVSCKLNLEKFPFDRQTCV-------------------   191
XP_034309618.1    ------FVNSTGQVVLGEGIQSIIMCAFNTQKFPFDTQTCE-------------------   179
PIK58946.1        ------LLTSDGSVSLGTPLVLSTQCPILIYYFPFDTQVCP-------------------   195
VUZ42516.1        ------TVDYTGHIFWSPPAIYKSMCKIQMEHFPFDHQICY-------------------   173
PAA51166.1        ------VVYEDGSVLWVPQSLFKSTCPVEILHFPFDTQLCQ-------------------   179
TGZ55997.1        ------VIEKDGSVLWIPQALYKSTCEVEITYFPFDTQVCM-------------------   181
VDP48851.1        ------VVSSNGETLWLPQALFKSTCEVEITYFPFDTQICM-------------------   137
PVD38331.1        ------VTYSTGDLLWMPQAILRSSCSFDTKFFPFDEQECI-------------------   159
VDK73355.1        --------------------------------FPFDEQRCH-------------------   129
VDD84963.1        ------TIRYTGEITWEPPAIFKSNCPIDVQWFPFDEQKCE-------------------   166
VDK42196.1        ------TLHHSGEVTWEPPAIFKSMCQIDVRWFPFDEQKCH-------------------   94
KFD58288.1        ------LVHHNGTVVWEPPAFYKSMCPIDIEWFPFDEQLCD-------------------   179
OUC49089.1        ------AVYYNGTVVWEPPAIYKSMCPIDIEWFPFDEQLCD-------------------   176
EYC26492.1        ------KLSYNGTVEWAPPAIYKSMCQIDVEFFPFDRQQCE-------------------   194
VDK27218.1        ------KLSPNGTVEWSPPAIYKSMCQIDVEWFPFDVQTCE-------------------   163
VDM17286.1        ------ILHSDGRVLWIPPAIFKTACEIDVRYFPFDQQTCH-------------------   94
VDL91846.1        ------QVFYNGLVRWMPPALYLTSCRINVEFFPYDEQECS-------------------   153
KAE9548540.1      ------LVTYDGRVNWQPPAIFKSFCPIDVTWFPYDTQNCT-------------------   167
PDM74087.1        ------VIESTGDMLWVPPALYKSSCIIDVEYFPFDQQSCY-------------------   167
XP_024504322.1    ------VINHKGDVLWVPPAIYKSSCIIDVEYFPMDQQICH-------------------   168
PIO52336.1        ------VINHLGDMLWVPPAIYKSSCIIDVEFFPFDEQVCT-------------------   164
TKR73865.1        ------VIDHKGEMLWVPPAIYKSSCIIDVEFFPFDEQTCH-------------------   169
RLU23395.1        ------LIYPNGEVLWVPPAIYQSSCTIDVTYFPFDQQTCI-------------------   171
XP_002427906.1    ------LIYPNGEVLWVPPAIYQSSCTIDVTYFPFDQQTCI-------------------   172
TMW47392.1        ------LIYPTGEVLWVPPAIYQSSCTIDVTYFPFDQQTCI-------------------   170
VDD83956.1        ------IVHYDGTVIWRPPIIFKSQCGINVEYFPFDMQNCS-------------------   167
VEL33078.1        ------TVYHDGQVIWIPPAIYKSSCQIDVEFFPYDEQVCS-------------------   100
XP_009019088.1    ------VVMNTGKIKWKPPASFRSSCLIDVTYFPFDEQMCG-------------------   169
KAE9536378.1      ------VLHYSGKVMWTPPAIFKSSCEIDVRYFPFDQQTCF-------------------   204
KDR23473.1        ------ILNYTGKVTWTPPAIFKSSCEIDVRYFPFDQQTCF-------------------   153
KAF7266914.1      ------TLKYTGEVIWKPPSIYKSSCEINVQYFPFDEQSCL-------------------   186
KAF4519445.1      ------TLKYTGEVVWKPPAIYKSSCEINVEYFPFDEQSCN-------------------   183
OXU25983.1        ------TLKYTGEVYWKPPAIYKSSCEIDVEYFPFDEQSCN-------------------   171
TGZ32403.1        ------TLKYTGEVFWKPPAIYKSSCEINVEYFPFDEQSCI-------------------   168
ELT94491.1        ------TLHHDGRVVWKPPAIYKSLCPINVMFFPFDEQLCT-------------------   168
VVC39575.1        ------MLHYSGRVEWKPPAIYKSSCEIDVEFFPFDEQTCV-------------------   262
RVE49089.1        ------TLNYTGRVEWRPPAIYKSSCEIDVEYFPFDQQTCV-------------------   166
KAB0800277.1      ------ILHHTGKVVWKPPAIYKSFCEIDVEYFPFDEQTCF-------------------   163
TDG52197.1        ------ILHHTGKVVWKPPAIYKSFCEIDVEYFPFDEQTCF-------------------   149
TMW48669.1        ------ILHHTGKVVWKPPAIYKSFCEIDVEYFPFDEQTCF-------------------   164
XP_025896085.1    ------YVQHHGEVRNLKPIQVMTACSLDIYSFPFDVQNCS-------------------   174
KAF2977017.1      ------------------------------------------------------------   0
XP_009321837.1    ------RGRQPRGSDPPRP---PRARAAPRFVFPLPVLGAG-------------------   151
XP_009979985.1    ------------GRWNPRP---PLARHGH--VLPCALYSRH-------------------   129
XP_028942374.1    ------YVGHHGEVQNLKPIQVMTACSLDIYNFPFDVQNCS-------------------   149
PKK19633.1        ------YVGHHGEVQNLKPIQVVTACSLDIYNFPFDVQNCS-------------------   216
XP_009894240.1    ------YVSHHGEVQNLKPIQVMTACSLDIYNFPFDVQNCS-------------------   270
XP_032820219.1    ------YLSYNGTVMNYKPLQVVSACSLDIYSFPFDVQNCT-------------------   203
XP_029429480.1    ------YLDYEGRVRNYKPVQAVTTCSLRIYSFPFDWQNCT-------------------   212
XP_030077572.1    ------YVTSEGKVRNYKPIQAVTSCSLNIYNFPFDLQICN-------------------   159
XP_033774596.1    ------YVTSDGKVRNYKPIQAVTSCSLNIYNFPFDLQICS-------------------   190
```

ETE72600.1	------YILYNGEVRNLKPVQVVTACSLDIYNFPFDVQNCS-------------------	205
XP_018082638.1	------YVNHEGRVQNYKPIQVVTACSLNIYNFPFDVQNCS-------------------	386
XP_032905410.1	------YVNYEGQVKNYKPIQVVTACSLEIYSFPFDVQNCT-------------------	169
XP_020773490.1	------YVTHDGQVRNYKPIQVVTACTLNIYNFPFDVQKCS-------------------	178
XP_033833634.1	------YVTHDGQVRNYKPIQVVTACTLNIYNFPFDVQKCS-------------------	204
XP_030215795.1	------YVTHEGLVSNYKPIQVVTACTLNIYNFPFDVQNCS-------------------	177
XP_030602980.1	------YVTNDGFVRNYKPIQVVTACTLNIYNFPFDVQNCS-------------------	183
XP_004573543.1	------YVTDDGVVRNYKPIQVVTACTLNIYNFPFDVQNCS-------------------	177
XP_005916159.1	------YVTDDGVVRNYKPIQVVTACTLNIYNFPFDVQNCS-------------------	177
XP_013132089.1	------YVTDDGVVRNYKPIQVVTACTLNIYNFPFDVQNCS-------------------	177
XP_031603488.1	------YVTDDGVVRNYKPIQVVTACTLNIYNFPFDVQNCS-------------------	183
XP_005725536.1	------YVTDDGVVRNYKPIQVVTACTLNIYNFPFDVQNCS-------------------	183
XP_026038075.1	------YVTDDGVVRNYKPIQVVTACTLNIYNFPFDVQNCS-------------------	183
CAF96649.1	------YVSHDGLVRNYKPIQVVTACTLNIYNFPFDVQKCS-------------------	146
XP_023818570.1	------YVTHDGLVRNYKPIQVVTACTLNIYNFPFDVQKCS-------------------	173
RVE64289.1	------YVTHDGLVRNYKPIQVVTACTLNIYNFPFDVQKCS-------------------	208
XP_024121971.1	------YVTHEGLVRNYKPIQVVTACTLNIYNFPFDVQKCS-------------------	179
XP_015817559.1	------YVTHEGLVQNYKPIQVVTACTLNIYNFPFDVQKCS-------------------	163
XP_015225670.1	------YVTHEGLVQNYKPIQVVTACTLNIYNFPFDVQKCS-------------------	162
XP_012722063.1	------YVTHEGLVQNYKPIQVVTACTLNIYNFPFDVQKCS-------------------	177
XP_032431307.1	------YVTHEGLVQNYKPIQVVTACTLNIYNFPFDVQKCS-------------------	177
XP_014328329.1	------YVTHEGLVQNYKPIQVVTACTLNIYNFPFDVQKCS-------------------	177
XP_027886578.1	------YVTHEGLVQNYKPIQVVTACTLNIYNFPFDVQKCS-------------------	177
XP_008426791.1	------YVTHEGLVQNYKPIQVVTACTLNIYNFPFDVQKCS-------------------	177
XP_014838686.1	------YVTHEGLVQNYKPIQVVTACTLNIYNFPFDVQKCS-------------------	177
XP_014885967.1	------YVTHEGLVQNYKPIQVVTACTLNIYNFPFDVQKCS-------------------	177
XP_033954312.1	------YVTHDGLVRNYKPIQVVTACTLNIYNFPFDVQKCS-------------------	177
XP_034089244.1	------YVTHDGLVRNYKPIQVVTACTLNIYNFPFDVQKCS-------------------	177
XP_010780064.1	------YVTHDGLVRNYKPIQVVTACTLNIYNFPFDVQKCS-------------------	193
XP_033970261.1	------YVTHDGLVRNYKPIQVVTACTLNIYNFPFDVQKCS-------------------	193
XP_029375575.1	------YVTHDGLVRNYKPIQVVTACTLNIYNFPFDVQKCS-------------------	214
KAF0023022.1	------YVTHDGLVRNYKPIQVVTACTLNIYNFPFDVQKCS-------------------	210
XP_019952614.1	------YVTHDGLVRNYKPIQVVTACTLNIYNFPFDVQKCS-------------------	166
XP_034463117.1	------YVTHEGLVRNYKPIQVVTACTLNIYNFPFDVQKCS-------------------	177
XP_029924656.1	------YVTHDGLVRNYKPIQVVTACTLNIYNFPFDVQNCS-------------------	178
XP_028323228.1	------YVTNNGLVRNYKPIQVVTACTLNIYNFPFDAQKCS-------------------	177
TNM84480.1	------YVTHDGLVRNYKPIQVVTACTLNIYNFPFDVQKCS-------------------	177
XP_029703788.1	------YVTHDGLVRNYKPIQVVTACTLNIYNFPFDVQKCS-------------------	177
XP_030010368.1	------YVRYNGMVRNYKPLQVVTACTLNIYNFPFDVQKCS-------------------	180
XP_019725056.1	------YVTHDGLVRNYKPIQVVTACTLNIYNFPFDVQKCS-------------------	178
XP_034034934.1	------YVTHDGLVRNYKPIQVVTACTLNIYNFPFDVQKCS-------------------	166
XP_029956880.1	------YVTHNGLVRNYKPIQVVTACTLNIYNFPFDVQKCS-------------------	177
XP_008331307.3	------YVTHDGLVRNYKPIQVVTACTLNIYNFPFDVQKCS-------------------	210
XP_031724845.1	------YVTHDGLVRNYKPIQVVTACTLNIYNFPFDVQKCS-------------------	178
XP_013859395.1	------YVTHDGLVRNYKPIQVVTACTLNIYNFPFDVQKCS-------------------	177
XP_017275335.1	------YVTHDGLVRNYKPIQVVTACTLNIYNFPFDVQKCS-------------------	177
XP_020496197.1	------YVTHDGLVRNYKPIQVVTACTLNIYNFPFDVQKCS-------------------	177
XP_029030400.1	------YVTNDGVVRNYKPIQVVTACTLNIYNFPFDVQKCS-------------------	177
XP_022060538.1	------YVTHDGLVRNYKPIQVVTACTLNIYNFPFDVQKCS-------------------	178
XP_023150584.1	------YVTHDGLVRNYKPIQVVTACTLNIYNFPFDVQKCS-------------------	178
XP_029303892.1	------YVTHDGLVRNYKPIQVVTACTLNIYNFPFDVQKCS-------------------	201
XP_020454172.1	------YVTHEGLVRNYKPIQVVTACTLNIYNFPFDVQKCS-------------------	176
TKS83244.1	------YVTYDGLVRNYKPIQVVTACTLNIYNFPFDVQKCS-------------------	164
XP_022616583.1	------YVTHDGLVRNYKPIQVVTACTLNIYNFPFDVQKCS-------------------	210
XP_030293172.1	------YVTHDGLVRNYKPIQVVTACTLNIYNFPFDVQKCS-------------------	177
XP_027129539.1	------YVTYDGLVRNYKPIQVVTACTLNIYNFPFDVQKCS-------------------	177
XP_034539538.1	------YVTHDGLVRNYKPIQVVTACTLNIYNFPFDVQKCS-------------------	177
XP_008277330.1	------YVTHDGLVRNYKPIQVVTACTLNIYNFPFDVQKCS-------------------	177
XP_028276940.1	------YVTHNGLVRNYKPIQVVTACTLNIYNFPFDVQKCS-------------------	177
XP_023263433.1	------YVTHDGLVRNYKPIQVVTACTLNIYNFPFDVQKCS-------------------	177
XP_034406336.1	------YVTHDGLVRNYKPIQVVTACTLNIYNFPFDVQKCS-------------------	177
XP_026184460.1	------YVTHEGLVRNYKPIQVVTACTLNISNFPFDVQKCS-------------------	177
KAF1378228.1	------YVTHDGLVRNYKPIQVVTACTLNIYNFPFDVQKCS-------------------	162
XP_028450365.1	------YVTHDGLVRNYKPIQVVTACTLNIYNFPFDVQKCS-------------------	177
XP_031163851.1	------YVTHDGLVRNYKPIQVVTACTLNIYNFPFDVQKCS-------------------	177
XP_032389084.1	------YVTHDGLVRNYKPIQVVTACTLNIYNFPFDVQKCS-------------------	177
XP_033494682.1	------YVTHDGLVRNYKPIQVVTACTLNIYNFPFDVQKCS-------------------	177
XP_018544782.1	------YVTHDGLVRNYKPIQVVTACTLNIYNFPFDVQKCS-------------------	177
XP_026228189.1	------YVTHDGLVRNYKPIQVVTACTLNIYNFPFDVQKCS-------------------	177
XP_028976606.1	------YVTHYGLVRNYKPIQVVTACTLNIYNFPFDVQNCS-------------------	177
CAB1352378.1	------YVTHYGLVRNYKPIQVVTACTLNIYNFPFDVQNCS-------------------	177
XP_023866849.1	------YVTHYGLVRNYKPIQVVTACTLNIYNFPFDVQNCS-------------------	177
XP_013992832.1	------YVTHYGLVRNYKPIQVVTACTLNIYNFPFDVQNCS-------------------	177
XP_029546688.1	------YVTHYGLVRNYKPIQVVTACTLNIYNFPFDVQNCS-------------------	177
XP_021481546.1	------YVTHYGLVRNYKPIQVVTACTLNIYNFPFDVQNCS-------------------	177
XP_020339889.1	------YVTHYGLVRNYKPIQVVTACTLNIYNFPFDVQNCS-------------------	177
XP_029481972.1	------YVTHYGLVRNYKPIQVVTACTLNIYNFPFDVQNCS-------------------	177

KPP68743.1	------YVRHNGLVRNYKPIQVVTACSLNIYNFPFDVQNCS-------------------	154
XP_023669383.1	------YVGHNGLVRNYKPIQVVTACSLNIYNFPFDVQNCS-------------------	176
KAA0720096.1	------YVGNDGLVRNYKPIQVVTACSLNIYNFPFDVQNCS-------------------	82
XP_009293684.1	------YVGHDGLVSNYKPIQVVTACSLNIYNFPYDVQKCS-------------------	176
XP_026090784.1	------YVGHTGLVRNYKPIQVVTACSLNIYNFPYDVQKCS-------------------	176
XP_018918715.1	------YVGNTGLVRNYKPIQVVTACSLNIYNFPYDVQKCS-------------------	176
KAF4098549.1	------YVGHTGLVRNYKPIQVVTACSLNIYNFPYDVQKCS-------------------	176
XP_016084173.1	------YVGHTGLVRNYKPIQVVTACSLNIYNFPYDVQKCS-------------------	176
XP_016332759.1	------YVGHTGLVRNYKPIQVVTACSLNIYNFPYDVQKCS-------------------	176
XP_016398039.1	------YVGHTGLVRNYKPIQVVTACSLNIYNFPYDVQKCS-------------------	176
XP_026989970.1	------YVNNEGLVQNYKPIQVVTACSLNIYNFPFDVQKCS-------------------	176
TSQ12698.1	------YVNNEGLVQNYKPIQVVTACSLNIYNFPFDVQKCS-------------------	176
XP_017347546.1	------YVSNEGLVQNYKPIQVVTACSLNIYNFPFDVQKCS-------------------	171
XP_026794616.2	------YVSNEGLVQNYKPIQVVTACSLNIYNFPFDVQKCS-------------------	176
XP_026861889.1	------YVRSDGLVQNYKPIQVVTACSLNIYNFPFDVQNCS-------------------	176
XP_017575347.1	------YVSNDGLVQNYKPIQVVTACSLNIYNFPFDVQKCS-------------------	176
XP_022531596.1	------YVTSDGLVQNYKPIQVVTACSLNIYNFPFDIQNCS-------------------	176
XP_012691019.2	------YVSHDGLVRNYKPIQVVTACTLNIYNFPFDVQNCS-------------------	159
XP_028839723.1	------YVRHDGLVRNYKPIQVVTACTLNIYNFPFDVQNCS-------------------	176
XP_030638860.1	------YVRHDGLVRNYKPIQVVTACTLNIYNFPFDVQNCS-------------------	158
XP_007882964.1	------YVSHDGRVQNYKPIQVVAACSLDIYNFPFDIQNCT-------------------	152
GCB70244.1	------YVDNGGRVKNYKPIQVVTACSLDIYNFPFDVQNCT-------------------	153
GCC26242.1	------YVDHEGRVKNYKPIQVVTACSLDIYNFPFDVQNCT-------------------	174
XP_020387034.1	------YVDYEGRVKNYKPIQVVTACSLDIYNFPFDVQNCT-------------------	165
XP_028931940.1	------YVRHHGEVQNYKPIQVVTACSLDIYNFPFDVQNCS-------------------	182
XP_031762490.1	------YVNHEGRVQNYKPIQVVTACSLNIYNFPFDVQNCS-------------------	171
PIO32240.1	--	0
XP_018425466.1	------YVNHEGRVLNYKPIQVVTACSLNIYNFPFDLQNCS-------------------	172
XP_006642351.1	------YVGYDGKVQNYKPIQVVTACSLDIYNFPFDIQNCT-------------------	193
XP_028665372.1	------YVRSDGLVRNYKPIQVVTACSLDIYNFPFDVQNCT-------------------	176
GCF49814.1	------YIFYTGEVQNLKPVQVVTACSLDIYNFPFDVQNCS-------------------	155
XP_015277816.1	------YILHTGEVQNLKPVQVVTACSLDIYNFPFDVQNCS-------------------	178
XP_005987340.2	------YVGHHGEVRNYKPIQVVTACSLDIYNFPFDVQNCS-------------------	160
XP_019339534.1	------YVLHQGEVQNLKPIQVVTACSLDIYNFPFDVQNCS-------------------	177
XP_025063414.1	------YVLHQGEVQNLKPIQVVTACSLDIYNFPFDVQNCS-------------------	177
XP_019367488.1	------YVRHQGEVQNLKPIQVVTACSLDIYNFPFDVQNCS-------------------	177
XP_019412078.1	------YVRHQGEVQNLKPIQVVTACSLDIYNFPFDVQNCS-------------------	177
XP_014434314.1	------YVRSHGEVKNLKPIQVVTACSLDIYNFPFDVQNCS-------------------	177
XP_007060556.1	------YIRYHGEVQNLKPIQVVTACSLDIYNFPFDVQNCS-------------------	171
XP_024072175.1	------YVRYHGEVQNLKPIQVVTACSLDIYNFPFDVQNCS-------------------	156
XP_008170567.1	------YVRYHGEVQNLKPIQVVTACSLDIYNFPFDVQNCS-------------------	177
XP_034610543.1	------YVRYHGEVQNLKPIQVVTACSLDIYNFPFDVQNCS-------------------	177
XP_030394127.1	------YVRYHGEVQNLKPIQVMTACSLDIYNFPFDVQNCS-------------------	177
XP_032651960.1	------YVSYHGEVQNLKPIQVMTACSLDIYNFPFDVQNCS-------------------	177
XP_025020083.1	------YILYNGEVRNLKPVQVVTACSLDIYNFPFDVQNCS-------------------	177
XP_026536833.1	------YILYNGEVRNLKPVQVVTACSLDIYNFPFDVQNCS-------------------	177
XP_026564787.1	------YILYNGEVRNLKPVQVVTACSLDIYNFPFDVQNCS-------------------	177
XP_032084675.1	------YILYDGEVRNLKPVQVVTACSLDIYNFPFDVQNCS-------------------	162
XP_029139341.1	------YILYNGEVRNLKPVQVVTACSLDIYNFPFDVQNCS-------------------	177
XP_034281006.1	------YILYNGEVRNLKPVQVVTACSLDIYNFPFDVQNCS-------------------	177
XP_020649062.1	------YILYDGEVRNLKPVQVVTACSLDIYNFPFDVQNCS-------------------	177
XP_008117087.1	------YILHDGEVRNLKPVQVVTACSLDIYNFPFDVQNCS-------------------	177
XP_028564324.1	------YILHHGEVRNLKPVQVVTACSLDIYNFPFDVQNCS-------------------	183
XP_033028155.1	------YILHHGEVRNLKPVQVVTACSLDIYNFPFDVQNCS-------------------	182
XP_025963249.1	------YVHHHGEVWNLKPIQVMTACSLDIYNFPFDVQNCS-------------------	164
XP_009668348.1	------YVHHYGEVRNLKPIQVMTACSLDIYNFPFDVQNCS-------------------	176
XP_013798935.1	------YVHHYGEVWNLKPIQVMTACSLDIYNFPFDVQNCS-------------------	160
XP_025913685.1	------YVHHYGEVWNLKPIQVMTACSLDIYNFPFDVQNCS-------------------	185
XP_013042552.1	------YVRHHGEVQNLKPIQVVTACSLDIYNFPFDVQNCS-------------------	183
XP_005030458.2	------YVRHHGEVQNLKPIQVVTACSLDIYNFPFDVQNCS-------------------	176
XP_032057953.1	------YVRHHGEVQNLKPIQVVTACSLDIYNFPFDVQNCS-------------------	159
XP_021232050.1	------YVRHHGEVQNLKPIQVVTACSLDIYNFPFDVQNCS-------------------	178
OXB62403.1	------YIRHHGEVQNLKPIQVVTACSLDIYNFPFDVQNCS-------------------	177
OXB81319.1	------YIRHHGEVQNLKPIQVVTACSLDIYNFPFDVQNCS-------------------	159
XP_010722007.1	------YIRHHGEVQNLKPIQVVTACSLDIYNFPFDVQNCS-------------------	178
XP_015739349.1	------YVRHHGEVQNLKPIQVVTACSLDIYNFPFDVQNCS-------------------	178
XP_031455498.1	------YVRHHGEVQNLKPIQVVTACSLDIYNFPFDVQNCS-------------------	179
POI27435.1	------YVRHHGEVQNLKPIQVVTACSLDIYNFPFDVQNCS-------------------	154
XP_004948120.1	------YVRHHGEVQNLKPIQVVTACSLDIYNFPFDVQNCS-------------------	179
XP_032851190.1	------YVGHHGEVQNLKPIQVMTACSLDIYNFPFDVQNCS-------------------	174
XP_010007255.1	------YVGHHGEVQNLKPIQVMTACSLDIYNFPFDVQNCS-------------------	176
XP_030320702.1	------YVSHHGEVQNLKPIQVMTACSLDIYNFPFDVQNCS-------------------	147
XP_010191940.1	------YVSHHGEVQNLKPIQVMTACSLDIYNFPFDVQNCS-------------------	174
XP_027737112.1	------YVSHHGEVQNLKPIQAMTTCSLDIYNFPFDVQNCS-------------------	172
XP_027555032.1	------YVSHHGEVQNLKPIQVMTACSLDIYNFPFDVQNCS-------------------	177
XP_032565370.1	------YVSHHGEVQNLKPIQVMTACSLDIYNFPFDVQNCS-------------------	223
XP_027511217.1	------YVSHHGEVQNLKPIQVMTACSLDIYNFPFDVQNCS-------------------	322

```
XP_027593499.1      ------YVSHHGEVQNLKPIQVMTACSLDIYNFPFDVQNCS-------------------   273
XP_017664924.1      ------YVSHHGEVQNLKPIQVMTACSLDIYNFPFDVQNCS-------------------   174
XP_029817938.1      ------YVSHHGEVQNLKPIQVMTACSLDIYNFPFDVQNCS-------------------   156
XP_005058641.1      ------YVSHHGEVQNLKPIQVMTACSLDIYNFPFDVQNCS-------------------   207
XP_021385804.1      ------YVSHHGEVQNLKPIQVMTACSLDIYNFPFDVQNCS-------------------   176
XP_030146687.2      ------YVSHHGEVQNLKPIQVMTACSLDIYNFPFDVQNCS-------------------   221
KAF4796420.1        ------YVSHHGEVQNLKPIQVMTACSLDIYNFPFDVQNCS-------------------   176
XP_032937581.1      ------YVSHHGEVQNLKPIQVMTACSLDIYNFPFDVQNCS-------------------   223
XP_031989659.1      ------YVSHHGEVQNLKPIQVMTACSLDIYSFPFDVQNCS-------------------   177
XP_010402086.1      ------YVSHHGEVQNLKPIQVMTACSLDIYSFPFDVQNCS-------------------   176
XP_017594069.1      ------YVSHHGEVQNLKPIQVMTACSLDIYSFPFDVQNCS-------------------   147
XP_014115268.1      ------YVSHHGEVQNLKPIQVMTACSLDIYNFPFDVQNCS-------------------   201
XP_023797108.1      ------YVSHHGEVQNLKPIQVMTACSLDIYNFPFDVQNCS-------------------   147
XP_033375614.1      ------YVSHHGEVQNLKPIQVMTACSLDIYNFPFDVQNCS-------------------   147
XP_014740121.1      ------YVSHHGEVQNLKPIQVMTACSLDIYNFPFDVQNCS-------------------   321
RLV83430.1          ------YVSHHGEVQNLKPIQVMTACSLDIYNFPFDVQNCS-------------------   167
XP_009096098.2      ------YVSHHGEVQNLKPIQVMTACSLDIYNFPFDVQNCS-------------------   282
TRZ15870.1          ------YVSHHGEVQNLKPIQVMTACSLDIYNFPFDVQNCS-------------------   177
RMB91935.1          ------YVSHHGEVQNLKPIQVMTACSLDIYNFPFDVQNCS-------------------   147
XP_030820843.1      ------YVSHHGEVQNLKPIQVMTACSLDIYNFPFDVQNCS-------------------   223
XP_014165179.1      ------YVSHHGEVQNLKPIQVMTACSLDIYNFPFDVQNCS-------------------   225
XP_026653582.1      ------YVSHHGEVQNLKPIQVMTACSLDIYNFPFDVQNCS-------------------   107
PKU35975.1          ------YVSHHGEVQNLKPIQVMTACSLDIYNFPFDVQNCS-------------------   179
XP_014805072.1      ------YVSHHGEVQNLKPIQVMTACSLDIYNFPFDVQNCS-------------------   173
XP_009818330.1      ------------------------------------------------------------   0
OPJ68307.1          ------YVGHHGEVQNLKPIQVVTACSLDIYNFPFDVQNCS-------------------   176
XP_008936289.1      ------YVGYHGEVQNLKPIQVMTACSLDIYNFPFDVQNCS-------------------   177
XP_010287046.1      ------YVSHHGEVQNLKPIQVMTACSLDIYNFPFDVQNCS-------------------   99
XP_005240140.2      ------YVGHHGEVQNLKPIQVMTACSLDIYNFPFDVQNCS-------------------   236
XP_005437752.2      ------YVGHHGEVQNLKPIQVMTACSLDIYNFPFDVQNCS-------------------   236
KFV74811.1          ------YVDHHGEVQNLKPIQVMTACSLDIYNFPFDVQNCS-------------------   141
XP_010018389.1      ------YVGHHGEVQNLKPIQVMTACSLDIYNFPFDVQNCS-------------------   184
KQK78711.1          ------YVGQHGEVQNLKPIQVMTACSLDIYNFPFXVQNCS-------------------   176
XP_009570162.1      ------YVDHHGEVQNLKPIQVMTACSLDIYNFPFDVQNCS-------------------   175
KFP11268.1          ------YVGHHGEVQNLKPIQVMTACSLDIYNFPFDVQNCS-------------------   146
KFQ98910.1          ------YVGHHGEVQNLKPIQVMTACSLDIYNFPFDVQNCS-------------------   160
XP_012985202.3      ------YVDHHGEVQNLKPIQVMTACSLDIYNFPFDVQNCS-------------------   176
XP_010573388.1      ------YVGHHGEVQNLKPIQVMTACSLDIYNFPFDVQNCS-------------------   175
XP_029879496.1      ------YVGHHGEVQNLKPIQVMTACSLDIYNFPFDVQNCS-------------------   147
XP_030361086.1      ------YVGHHGEVQNLKPIQVMTACSLDIYSFPFDVQNCS-------------------   228
KFM00668.1          ------YVSHHGEVQNLKPIQVMTACSLDIYNFPFDVQNCS-------------------   160
KAF1479074.1        ------YVSHHGEVQNLKPIQVMTACSLDIYNFPFDVQNCS-------------------   139
KAF1651161.1        ------YVSHHGEVQNLKPIQVMTACSLDIYNFPFDVQNCS-------------------   140
KAF1673648.1        ------YVSHHGEVQNLKPIQVMTACSLDIYNFPFDVQNCS-------------------   140
KAF1493319.1        ------YVSHHGEVQNLKPIQVMTACSLDIYNFPFDVQNCS-------------------   140
KAF1584157.1        ------YVSHHGEVQNLKPIQVMTACSLDIYNFPFDVQNCS-------------------   140
KAF1571723.1        ------YVSHHGEVQNLKPIQVMTACSLDIYNFPFDVQNCS-------------------   140
KAF1533169.1        ------YVSHHGEVQNLKPIQVMTACSLDIYNFPFDVQNCS-------------------   140
KAF1638955.1        ------YVSHHGEVQNLKPIQVMTACSLDIYNFPFDVQNCS-------------------   140
KAF1549972.1        ------YVSHHGEVQNLKPIQVMTACSLDIYNFPFDVQNCS-------------------   140
KAF1606914.1        ------YVSHHGEVQNLKPIQVMTACSLDIYNFPFDVQNCS-------------------   140
KAF1510957.1        ------YVSHHGEVQNLKPIQVMTACSLDIYNFPFDVQNCS-------------------   140
KAF1498899.1        ------YVSHHGEVQNLKPIQVMTACSLDIYNFPFDVQNCS-------------------   140
KAF1411525.1        ------YVSHHGEVQNLKPIQVMTACSLDIYNFPFDVQNCS-------------------   140
KAF1429205.1        ------YVSHHGEVQNLKPIQVMTACSLDIYNFPFDVQNCS-------------------   140
XP_005334318.1      ------YVTSSGSIKNFKPIQVVSACSLQTYAFPFDIQNCS-------------------   166
XP_014395552.1      ------YVNSSGTIRNSKSIQVVSACSLETYAFPFDIQNCS-------------------   166
KAB0404854.1        ------YVQHHGEVQNYKPLQVVTACSLDIYNFPFDVQNCS-------------------   162
XP_007494828.1      ------YVRHQGEVQNYKPIQVVTACSLDIYNFPFDVQNCS-------------------   177
XP_003764254.1      ------YVRHQGEVQNYKPIQVVTACSLDIYNFPFDVQNCS-------------------   177
XP_020845489.1      ------YIRHQGEVRNYKPIQVVTACSLDIYNFPFDVQNCS-------------------   177
XP_027703160.1      ------YVRHQGEVQNYKPIQVVTACSLDIYNFPFDVQNCS-------------------   177
XP_017523929.1      ------YVRHHGEVQNYKPLHVVTACSLDIYNFPFDVQNCS-------------------   175
XP_008688428.1      ----------------------------------------G-------------------   132
XP_029812166.1      ------YVRHXXXXXXXXXXXXXTPPSQRLVHPPGPRLRAG-------------------   194
XP_025749781.1      ------YVGHHGEVQNYKPLQVMTACSLDIYNFPFDVQNCS-------------------   177
XP_004416432.1      ------YVGHHGEVQNYKPLQVMTACSLDIYNFPFDVQNCS-------------------   177
XP_027436262.1      ------YVGHHGEVQNYKPLQVMTACSLDIYNFPFDVQNCS-------------------   177
XP_032284025.1      ------YVGHHGEVQNYKPLQVMTACSLDIYNFPFDVQNCS-------------------   177
XP_006735421.1      ------YVGHHGEVQNYKPLQVMTACSLDIYNFPFDVQNCS-------------------   177
XP_021552166.1      ------YVGHHGEVQNYKPLQVMTACSLDIYNFPFDVQNCS-------------------   177
XP_026361066.1      ------YVGHHGEVQNYKPLQVMTACSLDIYNFPFDVQNCS-------------------   177
XP_034523598.1      ------YVGHHGEVQNYKPLQVMTACSLDIYNFPFDVQNCS-------------------   177
NP_001297113.1      ------YVGHHGEVQNYKPLQVMTACSLDIYNFPFDVQNCS-------------------   177
XP_032215488.1      ------YVGHHGEVQNYKPLQVMTACSLDIYNFPFDVQNCS-------------------   177
VCX31483.1          ------YVGHHGEVQNYKPLQVMTACSLDIYNFPFDVQNCS-------------------   177
XP_022369003.1      ------YVGHHGEVQNYKPLQVMTACSLDIYNFPFDVQNCS-------------------   177
```

XP_032694248.1	------YVGHHGEVQNYKPLQVMTACSLDIYNFPFDVQNCS-------------------	177
NP_001041584.1	------YVGHHGEVQNYKPLQVVTACSLDIYNFPFDVQNCS-------------------	177
XP_025862501.1	------YVGHHGEVQNYKPLQVVTACSLDIYNFPFDVQNCS-------------------	177
KAF0873564.1	------YVGHHGEVENYKPLQVVTACSLDIYNFPFDVQNCS-------------------	319
XP_025784751.1	------YVGHRGEVQNYKPLQVVTACSLDIYNFPFDVQNCS-------------------	177
XP_007075625.1	------YVGHHGEVQNYKPLQVVTACSLDIYNFPFDVQNCS-------------------	313
XP_030189489.1	------YVGHHGEVQNYKPLQVVTACSLDIYNFPFDVQNCS-------------------	177
XP_019324655.1	------YVGHHGEVQNYKPLQVVTACSLDIYNFPFDVQNCS-------------------	278
XP_023094886.1	------YVGHHGEVQNYKPLQVVTACSLDIYNFPFDVQNCS-------------------	313
XP_026892357.1	------YVGHHGEVQNYKPLQVVTACSLDIYNFPFDVQNCS-------------------	177
XP_016004457.1	------YVQHHGEVQNYKPLQVVTACSLNIYNFPFDVQNCS-------------------	172
XP_006912860.1	------YVRHHGEVQNYKPLQVVTACSLDIYNFPFDVQNCS-------------------	172
XP_011363679.1	------YVRHHGEVQNYKPLQVVTACSLDIYNFPFDVQNCS-------------------	172
XP_016076060.1	------YVRHHGEVQNYKPLQVVTACSLDIYNFPFDVQNCS-------------------	177
XP_008148371.1	------YVRHHGEVQNYKPLQVVTACSLDIYNFPFDVQNCS-------------------	177
XP_006093568.1	------YVGHQGEVQNYKPLQVVTACSLDIYNFPFDVQNCS-------------------	177
XP_006761601.1	------YVGHQGEVQNYKPLQVVTACSLDIYNFPFDVQNCS-------------------	177
XP_024426743.1	------YVQHHGEVQNYKPLQVVTACSLDIYNFPFDVQNCS-------------------	172
XP_019520779.1	------YVRHHGEVQNYKPLQVVTACSLDIYNFPFDVQNCS-------------------	177
XP_032976539.1	------YVRHHGEVQNYKPLQVVTACSLDIYNFPFDVQNCS-------------------	177
ELW64270.1	------YVRHHGEVQNYKPLQVVTACSLDIYNFPFDVQNCS-------------------	181
XP_004427367.1	------YVRHGGEVQNYKPLQVVTACSLDIYNFPFDVQNCS-------------------	177
XP_008529353.1	------YVRHCGEVQNYKPLQVVTACSLDIYNFPFDVQNCS-------------------	177
NP_001288165.1	------YVRHCGEVQNYKPLQVVTACSLDIYNFPFDVQNCS-------------------	177
XP_014711213.1	------YVRHCGEVQNYKPLQVVTACSLDIYNFPFDVQNCS-------------------	177
XP_012514621.1	------YVQHYGEVQNYKPLQVVTACSLDIYNFPFDVQNCS-------------------	177
XP_008071525.1	------YVRHHGEVQNYKPLQVVTACSLDIYNFPFDVQNCS-------------------	175
XP_009005082.1	------YIQHQGEVQNYKPLQVVTACSLDIYNFPFDVQNCS-------------------	177
XP_010332832.1	------YIQHQGEVQNYKPLQIVTACSLDIYNFPFDVQNCS-------------------	172
XP_012326058.1	------YIQHQGEVQNYKPLQVVTACSLDIYNFPFDVQNCS-------------------	183
XP_017378796.1	------YVQHQGEVQNYKPLQVVTACSLDIYNFPFDVQNCS-------------------	206
XP_032141374.1	------YIQHQGEVQNYKPLQVVTACSLDIYNFPFDVQNCS-------------------	206
XP_032024018.1	------YIRHQGEVQNYKPLQVVTACSLDIYNFPFDVQNCS-------------------	172
XP_018891497.2	------YIQHQGEVQNYKPLQVVTACSLDIYNFPFDVQNCS-------------------	178
XP_030684894.1	------YIRHQGEVQNYKPLQVVTACSLDIYNFPFDVQNCS-------------------	172
PNJ75904.1	------YIRHQGEVQNYKPLQVVTACSLDIYNFPFDVQNCS-------------------	178
AAP35868.1	------YIRHQGEVQNYKPLQVVTACSLDIYNFPFDVQNCS-------------------	178
XP_001149570.1	------YIQHQGEVQNYKPLQVVTACSLDIYNFPFDVQNCS-------------------	178
XP_003805532.1	------YIRHQGEVQNYKPLQVVTACSLDIYNFPFDVQNCS-------------------	178
XP_023063823.1	------YIRHQGEVQNYKPLQVVTACSLDIYNFPFDVQNCS-------------------	177
XP_033060210.1	------YIRHQGEVQNYKPLQVVTACSLDIYNFPFDVQNCS-------------------	177
XP_010370669.1	------YIRHQGEVQNYKPLQVVTACSLDIYNFPFDVQNCS-------------------	177
XP_017738496.1	------YIRHQGEVQNYKPLQVVTACSLDIYNFPFDVQNCS-------------------	177
XP_011782168.1	------YIRHQGEVQNYKPLQVVTACSLDIYNFPFDVQNCS-------------------	177
EHH23440.1	------YIRHQGEVQNYKPLQVVTACSLDIYNFPFDVQNCS-------------------	172
XP_003910778.3	------YIRHQGEVQNYKPLQVVTACSLDIYNFPFDVQNCS-------------------	177
XP_025212649.1	------YIRHQGEVQNYKPLQVVTACSLDIYNFPFDVQNCS-------------------	183
XP_008019132.1	------YIRHQGEVQNYKPLQVVTACSLDIYNFPFDVQNCS-------------------	177
XP_011832421.1	------YIRHQGEVQNYKPLQVVTACSLDIYNFPFDVQNCS-------------------	162
XP_011759618.1	------YIRHQGEVQNYKPLQVVTACSLDIYNFPFDVQNCS-------------------	177
XP_005579749.1	------YIRHQGEVQNYKPLQVVTACSLDIYNFPFDVQNCS-------------------	177
XP_011921684.1	--	0
XP_008259391.1	------YVQHQGEVQNYKPLQVVTACSLDIYNFPFDVQNCS-------------------	177
XP_012782438.1	------YVRHQGEVQNYKPLQVVTACSLDIYNFPFDVQNCS-------------------	173
VTJ86076.1	------YVQHQGEVQNYKPLQVVTACSLDIYNFPFDVQNCS-------------------	183
XP_015345533.1	------YVQHQGEVQNYKPLQVVTACSLDIYNFPFDVQNCS-------------------	183
XP_027786432.1	------YVQHQGEVQNYKPLQVVTACSLDIYNFPFDVQNCS-------------------	183
XP_026248345.1	------YVQHQGEVQNYKPLQVVTACSLDIYNFPFDVQNCS-------------------	177
XP_005378209.1	------YVRHQGEVQNYKPLQVVTACSLDIYNFPFDVQNCS-------------------	177
XP_004466010.1	------YVRHHGEVQNYKPLQVVTACSLDIYNFPFDVQNCS-------------------	177
XP_020035707.1	------YVRHQGEVQNYKPFQVVTACSLNIYNFPFDVQNCS-------------------	172
XP_006181802.1	------YVRHDGEVQNYKPLQVVTACSLDIYNFPFDVQNCS-------------------	177
XP_006207862.1	------YVRHDGEVQNYKPLQVVTACSLDIYNFPFDVQNCS-------------------	177
XP_005891968.1	------YVRHHGEVQNYKPLQVMTACTLDIYNFPFDVQNCS-------------------	174
XP_010828662.1	------YVRHHGEVQNYKPLQVMTACTLDIYNFPFDVQNCS-------------------	174
XP_019830126.1	------YVRHHGEVQNYKPLQVMTACTLDIYNFPFDIQNCS-------------------	173
XP_017914486.1	------YVRHHGEVQNYKPLQVMTACTLDIYNFPFDVQNCS-------------------	173
XP_006060266.2	------YVRHHGEVQNYKPLQVMTACTLDIYNFPFDVQNCS-------------------	178
XP_020728447.1	------YVRHHGEVQNYKPLQVMTACSLDIYNFPFDVQNCS-------------------	178
KAF4008892.1	------YVRHHGEVQNYKPLQVMTACSLDIYNFPFDVQNCS-------------------	178
KAB0348059.1	------YVRHHGEVQNYKPLQVMTACSLDIYNFPFDVQNCS-------------------	161
KAB0371113.1	------YVRHHGEVQNYKPLQVMTACSLDIYNFPFDVQNCS-------------------	154
MBV96963.1	------YVQHHGEVQNYKPLQVVTACSLDIYNFPFDVQNCS-------------------	177
XP_007172048.1	------YVQHHGEVQNYKPLQVVTACSLDIYNFPFDVQNCS-------------------	177
XP_007452270.1	------YVRHHGEVQNYKPLQVVTACSLDIYNFPFDVQNCS-------------------	177
XP_023987108.1	------YVRHHGEVQNYKPLQVVTACSLDIYNFPFDVQNCS-------------------	172
XP_004273432.1	------YVRHHGEVQNYKPLQVVTACSLDIYNFPFDVQNCS-------------------	172

```
XP_004328646.1      ------YVRHHGEVQNYKPLQVVTACSLDIYNFPFDVQNCS-----------------      172
XP_026937670.1      ------YVRHHGEVQNYKPLQVVTACSLDIYNFPFDVQNCS-----------------      172
XP_030691031.1      ------YVRHHGEVQNYKPLQVVTACSLDIYNFPFDVQNCS-----------------      172
XP_022414870.1      ------YVRHHGEVQNYKPLQVVTACSLDIYNFPFDVQNCS-----------------      177
XP_029064651.1      ------YVRHHGEVQNYKPLQVVTACSLDIYNFPFDVQNCS-----------------      177
XP_024607566.1      ------YVRHHGEVQNYKPLQVVTACSLDIYNFPFDVQNCS-----------------      177
XP_032496907.1      ------YVRHHGEVQNYKPLQVVTACSLDIYNFPFDVQNCS-----------------      177
XP_007535559.1      ------YVRHHGEVQNYKPLQVVTACSLDIYNFPFDVQNCT-----------------      170
XP_031299536.1      ------YVRHDGEVQNYKPLQVVTACSLDIYNFPFDVQNCS-----------------      176
XP_004666516.1      ------YVQHQGKVQNYKPLQVVTACSLDIYNFPFDVQNCS-----------------      172
XP_008830963.1      ------YVQHQGKVHNYKPLQVVTACSLDIYNFPFDVQNCS-----------------      177
XP_005347338.1      ------YVHHHGEVQNYKPLQVVTACTLDIYNFPFDVQNCS-----------------      177
XP_005069548.1      ------YVRHRGEVQNYKPLQVVTACSLDIYNFPFDVQNCS-----------------      177
XP_027267894.1      ------YVHHRGEVQNYKPLQVVTACSLDIYNFPFDVQNCS-----------------      177
OBS80992.1          ------YVHHQGEVQNYKPLQVVTACSLDIYNFPFDVQNCS-----------------      164
XP_006979812.1      ------YVHHQGEVQNYKPLQVVTACSLDIYNFPFDVQNCS-----------------      183
XP_028720687.1      ------YVHHQGEVQNYKPLQVVTACSLDIYNFPFDVQNCS-----------------      177
XP_021489005.1      ------YVNHQGEVQNYKPLQVVTACSLDIYNFPFDVQNCS-----------------      178
XP_031199589.1      ------YVDHRGQVQNYKPLQVVTSCSLDIYNFPFDVQNCS-----------------      202
XP_028617944.1      ------------------------------------------------------------      0
XP_034347030.1      ------YVHHRGKVQNYKPLQVVSACSLDIYNFPFDVQNCS-----------------      175
NP_077370.2         ------YVHHQGEVQNYKPLQLVTACSLDIYNFPFDVQNCS-----------------      177
XP_032766961.1      ------YVHHQGEVQNYKPLQLVTACSLDIYNFPFDVQNCS-----------------      177
XP_021063186.1      ------YVHHRGEVQNYKPLQLVTACSLDIYNFPFDVQNCS-----------------      160
NP_001093114.1      ------YVHHRGEVQNYKPLQLVTACSLDIYNFPFDVQNCS-----------------      177
XP_021028379.1      ------YVHHRGEVQNYKPLQLVTACSLDIYNFPFDVQNCS-----------------      177
XP_004689305.1      ------YVQQHGKVQNYKPLQVVTACSLDIYNFPFDVQNCS-----------------      177
XP_004712871.1      ------YVQQDGDIHNYKPLQVVTACSLDIYNFPFDVQNCS-----------------      177
XP_004382575.1      ------YVKQDGNVQNYKPLQVVTACSLDIYNFPFDVQNCS-----------------      177
XP_010596188.1      ------YVQQNGNVQNYKPLQVVTACSLDIYNFPFDVQNCS-----------------      177
XP_006890855.1      ------YVQQDGSVHNYKPLQVVTSCSLDIYNFPFDVQNCS-----------------      177
XP_006834038.1      ------YVHQNGNVQNYKPLQVVTACSLEIYNFPFDVQNCS-----------------      177
XP_007934716.1      ------YVQQDGNVQNYKPLQVVTACSLDIYNFPFDVQNCS-----------------      177
XP_012603491.1      ------YVQHHGEVQNYKPLQVVTACSLDIYNFPFDVQNCS-----------------      177
XP_012663606.1      ------YVRHHGEVQNYKPLQVVTACSLDIYNFPFDVQNCS-----------------      172
XP_028372812.1      ------YVRHHGEVQNYKPLQVVTACSLDIYNFPFDVQNCS-----------------      177
XP_008589608.1      ------YVRHDGEVRNYKPLQVVTACSLDIYNFPFDVQNCS-----------------      177
XP_012865077.1      ------YVQHKGEVQNYKPFQVVTACSLNIYNFPFDVQNCS-----------------      177
NP_001166178.1      ------YVRHQGEVQNYKPLQVVTACSLDIYNFPFDVQNCS-----------------      177
XP_004636553.1      ------YVQHQGEVQNYKPLQVVTACSLDIYNFPFDVQNCS-----------------      177
XP_004856670.1      ------YVQHQGEVQNYKPLQVVTACSLDIYNFPFDVQNCS-----------------      177
XP_010627744.1      ------YVQHQGEVQNYKPLQVVTACSLDIYNFPFDVQNCS-----------------      177
```

Loop-B **Loop-F**

```
NP_509270.1         LQFGSWSY-QAHA-ISFNV-----------------------------------------      191
VDO93178.1          MTFGSWMY-DSAD-INYDT-----------------------------------------      207
PAV91580.1          LTISSWTN-SKAA-LDYYG-----------------------------------------      200
VDK46997.1          LTISSWTN-SKSA-LDYYA-----------------------------------------      166
VIO86814.1          FACGSWSY-QYEY-VSLVV-----------------------------------------      225
VDP38785.1          IQLASWIY-DQSQ-LQLNII----------------------------------------      246
KOF68401.1          MTFSSWAY-LVNE-LDLY------------------------------------------      165
CDJ96026.1          LKFASWSH-DASE-IDLG------------------------------------------      314
EFX76216.1          MKFSSWTY-DGLQ-VNLL------------------------------------------      156
KAF7403848.1        LKWASWTY-DGYQ-LELE------------------------------------------      168
KAF7427032.1        LKWASWTY-DGYQ-LELE------------------------------------------      183
KAE9417558.1        IKYGSWTY-TGSK-LDLHID------------------------------D---------      170
KJH51504.1          FKFGSWSY-SRDK-IELAVG----------------------------------------      175
VDL69795.1          MKFGSWTF-DGSK-LDLDVD------------------------------D---------      166
VDO32671.1          MKFGSWTF-HGYA-LDLQIE------------------------------T---------      183
VBB32409.1          FKFGSWTY-GGDK-LDLQPG------------------------------K---------      166
VDN54565.1          FKFGSWTY-DGFK-LDLQPG------------------------------K---------      185
EGT55171.1          FKFGSWTY-DGYK-LDLQPA------------------------------T---------      196
RCN52111.1          FQFGSWTY-DGYK-LDLQPA------------------------------E---------      201
RMX54856.1          LKFGSWTY-DGFR-VDM-------------------------------------------      193
XP_022781674.1      LKFGSWTY-DGFR-MDM-------------------------------------------      455
RNA37099.1          LKIGSWTY-DGFQ-----------------------------------------------      194
KAA0187152.1        LKLGSWIY-DGFQ-AKCCVCAACEGVAQAKCCVCAACEGVAQAKGCVHVACEGVAQAKCC      367
GAU96593.1          MKFGSWTY-NGFQ-----------------------------------------------      192
XP_009043980.1      LKFGSWTY-DGFQ-----------------------------------------------      153
VDM43573.1          LKFGSWTY-HGFQ-----------------------------------------------      271
XP_003140283.1      IKFGSWTY-HGLQ-----------------------------------------------      162
VDN82010.1          IKFGSWTY-HGLQ-----------------------------------------------      188
VDO31501.1          IKFGSWTY-HGLQ-----------------------------------------------      190
VDP19591.1          LKFGSWTY-YGDQ-INFQ------------------------------LQCINASQPDC-      188
EDO32053.1          LTFGSWTH-DGLK-IDIH------------------------------LKQE--------      163
```

TRY67230.1	FKFISWTY-DASK-LNIS------------------------------LD-G--------	176
KAF7391312.1	MKFGSWTY-DGSQ-LDLV------------------------------LESE--------	188
GFG30449.1	MKFGSWTY-DGFQ-LDLQ------------------------------LQDE--------	198
KQS30083.1	MKFGSWTY-DGFQ-LDLQ------------------------------LQDE--------	220
EDW57583.2	MKFGSWTY-DGFQ-LDLQ------------------------------LQDE--------	356
KNC22799.1	MKFGSWTY-DGFQ-LDLQ------------------------------LQDE--------	166
RZF44856.1	MKFGSWTY-DGFQ-LDLQ------------------------------LQDE--------	186
ENN76856.1	MKFGSWTY-DGLQ-LDLQ------------------------------LQDD--------	192
KAF5300392.1	MKFGSWTY-DGLQ-LDLQ------------------------------LQDD--------	196
CAB3239999.1	MKFGSWTY-DGHQ-LDLQ------------------------------LQDE--------	183
PCG77624.1	MKFGSWTY-DGYQ-LDLQ------------------------------LQDE--------	126
PZC79131.1	MKFGSWTY-DGYQ-LDLQ------------------------------LQDE--------	193
KAF4083067.1	FTFGSYMH-TIRD-VRVKPALPFDE-----------------------------------	169
XP_009924659.1	LSVASFLY-PV-TDLVMKTKRTPAE-----------------------------------	192
XP_010123544.1	LSIASFLH-PAVEDLVMKTRRTPAE-----------------------------------	191
CBN81618.1	ISVKSVIH-SDWE-MDLLVFNNKSK-----------------------------------	189
KAF3704230.1	LSFKSVIH-TVKD-IRLQPSDNSSE-----------------------------------	184
RUS86578.1	LELFAMGW-TKSI---LRFLI---------------------------------------	208
XP_034309618.1	FEFFPTNY-FIHG-IHIIYNQSKF------------------------------------	201
PIK58946.1	FFFFPQNQ-HSEK-LILTTKTSP-------------------------------------	216
VUZ42516.1	LRFASWTL-HGKK-LNLTFLDG--------------------------------------	193
PAA51166.1	LEFGSWTY-DMTQ-MNITWYDYEITYDN-----N--------------------------	206
TGZ55997.1	LEFGSWTY-DRTQ-MDIIWWT------G-----S--------------------------	202
VDP48851.1	LEFGSLTY-DRTQ-MDISWWI------P-----D--------------------------	158
PVD38331.1	LKFSSWSY-NGFK-LDIHFLPN--------------------------------------	179
VDK73355.1	FEFGSWTY-SDDL-LDLKVLGGEPRYEL------EMNA------------------NGEM	163
VDD84963.1	LKFGPWSY-SGNL-VVLELFDSDKSYNK-----TEINE------------------KGIE	201
VDK42196.1	LKFGSWTY-SEDL-LVLDLLDGDARYEL------ETNE------------------FGEI	128
KFD58288.1	LKFGSWTY-DGFE-VDLIHYDKDKEEIT-----L-----------------------ENG	209
OUC49089.1	LKFGSWTY-DGFE-VDLIHYDKDDVQIT-----R-----------------------ENG	206
EYC26492.1	MKFGSWTY-GGLE-VDLIHKDEHLQEET-----IEMVEGVD---------------GPVQ	232
VDK27218.1	MKFGSWTY-GGLE-VDLQHKDSHIERTE-----TETVLGFD---------------GEYD	201
VDM17286.1	MDLGSWTY-TNKE-VELQFYVN--------------------------------------	114
VDL91846.1	MRFGSWTY-DGSK-VELRHHLQKYVPEM--------------------------------	179
KAE9548540.1	MKFGLWSF-TGHY-VDMKQLPAEKVKIA-----Q----------------------DEDG	198
PDM74087.1	LIFGSWTY-DEKE-LILDYV--	185
XP_024504322.1	LIFGSWTY-NENE-ITLDFDQ---------------------------------------	187
PIO52336.1	LVFGSWTY-NENE-IKLEFEQ---------------------------------------	183
TKR73865.1	LIFGSWTY-NENE-IKLEFVQ---------------------------------------	188
RLU23395.1	MKFGSWTF-NGDQ-VSLALYND--------------------------------------	191
XP_002427906.1	MKFGSWTF-NGDQ-VSLALYNN--------------------------------------	192
TMW47392.1	MKFGSWTF-NGDQ-VSLALYNN--------------------------------------	190
VDD83956.1	MKIGCWTH-HGLL-IDLRHTSQKANS-------Q----------------------PTFD	196
VEL33078.1	MKFGSWTY-DGGK-VDLKHITEKYAN-------G----------------------P---	126
XP_009019088.1	FQFGSWTY-NIQE-VDIQHAWQ----------------------------------P---	190
KAE9536378.1	MKFGSWSY-DGNQ-INLKHIGQ---L-------V----------------------G---	227
KDR23473.1	MKFGSWTY-DGFQ-IDLKHINQ---R-------F----------------------N---	176
KAF7266914.1	MKFASWTY-NGLQ-VDLKHMDQ---Q-------P----------------------G---	209
KAF4519445.1	MKFGSWTY-NGIQ-VDLKHMDQ---V-------L----------------------G---	206
OXU25983.1	MKFGSWTY-NGAQ-VDLKHMEQ---V-------P----------------------G---	194
TGZ32403.1	MKFGSWTY-NGAQ-VDLKHMKQ---E-------P----------------------G---	191
ELT94491.1	LKIGSWSY-DGFS-VDIRHKDLPMVN-------T----------------------D---	194
VVC39575.1	MKFGSWTY-DGFQ-VDLRHASE---I-------N----------------------G---	285
RVE49089.1	MKFGSWTY-DGFQ-VDLRHIDE---T-------R----------------------G---	189
KAB0800277.1	MKFGSWTY-DGYM-VDLRHLSQ---G-------Q----------------------D---	186
TDG52197.1	MKFGSWTY-DGYM-VDLRHLKQ---T-------A----------------------D---	172
TMW48669.1	MKFGSWTY-DGYM-VDLRHLKQ---T-------P----------------------D---	187
XP_025896085.1	LTFTSWL--HHIHDINLSLWRPPEL-----------------------------------	197
KAF2977017.1	------------------------L-----------------------------------	1
XP_009321837.1	RPWSSPLLHPPVRDINLSLWRQPEL-----------------------------------	176
XP_009979985.1	RAGSLP-----VPGKLQMGNGLPEL-----------------------------------	149
XP_028942374.1	LTFTSWL--HHSECRDLXXXXXRDL-----------------------------------	172
PKK19633.1	LTFTSWL--HHIRDLNLSLWRQPEL-----------------------------------	239
XP_009894240.1	LTFTSWL--HHSE-CAVARGRXPEL-----------------------------------	292
XP_032820219.1	LTFTSWL--HTSNDINLTLWRTTEE-----------------------------------	226
XP_029429480.1	FTFTSWL--HIIQDINLTVWREPQL-----------------------------------	235
XP_030077572.1	LTFVSWL--HVKQDIDLKLWNEPVH-----------------------------------	182
XP_033774596.1	LTFVSWL--HVKEDIDLKLWNESLY-----------------------------------	213
ETE72600.1	LTFTSWL--HNIRDINLSLWRTPEE-----------------------------------	228
XP_018082638.1	LTFTSWL--HTIKDINVSLWRTPEA-----------------------------------	409
XP_032905410.1	FTFTSWL--HNIRDINISLLRSPEA-----------------------------------	192
XP_020773490.1	LTFQSWL--HTIDDINITLMRTPEE-----------------------------------	201
XP_033833634.1	LTFQSWL--HTIDDINITLMRSPEE-----------------------------------	227
XP_030215795.1	LTFQSWL--HTIKDINITLMRSPQE-----------------------------------	200
XP_030602980.1	LTFQSWL--HTINDINITLMRNPED-----------------------------------	206
XP_004573543.1	LTFQSWL--HTIDDINITLMREPEE-----------------------------------	200
XP_005916159.1	LTFQSWL--HTIDDINITLMREPEE-----------------------------------	200

XP_013132089.1	LTFQSWL--HTIDDINITLMREPEE-----------------------------------	200
XP_031603488.1	LTFQSWL--HTIDDINITLMREPEE-----------------------------------	206
XP_005725536.1	LTFQSWL--HTIDDINITLMREPEE-----------------------------------	206
XP_026038075.1	LTFQSWL--HTIDDINITLMREPEE-----------------------------------	206
CAF96649.1	LTFQSWL--HTIDDINITLLRSPEE-----------------------------------	169
XP_023818570.1	LTFQSWL--HTIDDINITLMRSPEK-----------------------------------	196
RVE64289.1	LTFQSWL--HTIDDINITLMRSPEM-----------------------------------	231
XP_024121971.1	LTFQSWL--HTIDDINITLMRSPEM-----------------------------------	202
XP_015817559.1	LTFQSWL--HTIDDINITLMRSPEK-----------------------------------	186
XP_015225670.1	LTFQSWL--HTINDINITLMRSPEK-----------------------------------	185
XP_012722063.1	LTFQSWL--HTIDDINITLMRSPEM-----------------------------------	200
XP_032431307.1	LTFQSWL--HTIDDINITLMRSPEK-----------------------------------	200
XP_014328329.1	LTFQSWL--HTIDDINITLMRSPEK-----------------------------------	200
XP_027886578.1	LTFQSWL--HTIDDINITLMRSPEK-----------------------------------	200
XP_008426791.1	LTFQSWL--HTIDDINITLMRSPEK-----------------------------------	200
XP_014838686.1	LTFQSWL--HTIDDINITLMRSPEK-----------------------------------	200
XP_014885967.1	LTFQSWL--HTIDDINITLMRSPEK-----------------------------------	200
XP_033954312.1	LTFQSWL--HTIDDINITLMRSPEE-----------------------------------	200
XP_034089244.1	LTFQSWL--HTIDDINITLMRSPEE-----------------------------------	200
XP_010780064.1	LTFQSWL--HTIDDINITLMRSPEE-----------------------------------	216
XP_033970261.1	LTFQSWL--HTIDDINITLMRSPEE-----------------------------------	216
XP_029375575.1	LTFQSWL--HTIDDINITLIRSPEE-----------------------------------	237
KAF0023022.1	LTFQSWL--HTIDDINITLMRSPEE-----------------------------------	233
XP_019952614.1	LTFQSWL--HTIDDINITLMRSPEE-----------------------------------	189
XP_034463117.1	LTFQSWL--HTIDDINITLMRSPEE-----------------------------------	200
XP_029924656.1	LTFQSWL--HTIDDINITLMRSPEE-----------------------------------	201
XP_028323228.1	LTFQSWL--HTVDDINITLMRSPEE-----------------------------------	200
TNM84480.1	LTFQSWL--HTIDDINITLMRSPEE-----------------------------------	200
XP_029703788.1	LTFQSWL--HTIDDINITLMRSPEE-----------------------------------	200
XP_030010368.1	LTFQSWL--HTINDINITLMRSPEE-----------------------------------	203
XP_019725056.1	LTFQSWL--HTIDDINITLMRSPEE-----------------------------------	201
XP_034034934.1	LTFQSWL--HTINDINITLMRSPEE-----------------------------------	189
XP_029956880.1	LTFQSWL--HTIDDINITLIRSPEE-----------------------------------	200
XP_008331307.3	LTFQSWL--HTINDINITLMRSPEE-----------------------------------	233
XP_031724845.1	LTFQSWL--HTIDDINITLMRSPEE-----------------------------------	201
XP_013859395.1	LTFQSWL--HTIDDINITLMRSPEK-----------------------------------	200
XP_017275335.1	LTFQSWL--HTIDDINITLMRSPEK-----------------------------------	200
XP_020496197.1	LTFQSWL--HTIDDINITLMRSPEE-----------------------------------	200
XP_029030400.1	LTFQSWL--HTINDINITLIRSPEE-----------------------------------	200
XP_022060538.1	LTFQSWL--HTIDDINITLMRSPEE-----------------------------------	201
XP_023150584.1	LTFQSWL--HTIDDINITLMRSPEE-----------------------------------	201
XP_029303892.1	LTFQSWL--HTIDDINITLMRSPEE-----------------------------------	224
XP_020454172.1	LTFQSWL--HTIDDINITLMRSPEE-----------------------------------	199
TKS83244.1	LTFQSWL--HTVDDINITLMRSPEE-----------------------------------	187
XP_022616583.1	LTFQSWL--HTIDDINITLMRSPEE-----------------------------------	233
XP_030293172.1	LTFQSWL--HTIDDINITLMRTPEE-----------------------------------	200
XP_027129539.1	LTFQSWL--HTVDDINITLMRSPEE-----------------------------------	200
XP_034539538.1	LTFQSWL--HTIDDINITLMRSPEE-----------------------------------	200
XP_008277330.1	LTFQSWL--HTIDDINITLMRSPEE-----------------------------------	200
XP_028276940.1	LTFQSWL--HTIDDINITLMRSPEE-----------------------------------	200
XP_023263433.1	LTFQSWL--HTIDDINITLMRSPEE-----------------------------------	200
XP_034406336.1	LTFQSWL--HTIDDINITLMRSPEE-----------------------------------	200
XP_026184460.1	LTFQSWL--HTIDDINITLMRSPEE-----------------------------------	200
KAF1378228.1	LTFQSWL--HTIDDINITLMRSPEE-----------------------------------	185
XP_028450365.1	LTFQSWL--HTIDDINITLMRSPEE-----------------------------------	200
XP_031163851.1	LTFQSWL--HTIDDINITLMRSPEE-----------------------------------	200
XP_032389084.1	LTFQSWL--HTIDDINITLMRSPEE-----------------------------------	200
XP_033494682.1	LTFQSWL--HTIDDINITLMRSPEE-----------------------------------	200
XP_018544782.1	LTFQSWL--HTIDDINITLMRRPEE-----------------------------------	200
XP_026228189.1	LTFQSWL--HTIDDINITLMRSPEE-----------------------------------	200
XP_028976606.1	LTFQSWL--HTLNDIDLKLIRSADE-----------------------------------	200
CAB1352378.1	LTFQSWL--HTLNDIDLKLMRSAEE-----------------------------------	200
XP_023866849.1	LTFQSWL--HTLNDIDLKLMRSAEE-----------------------------------	200
XP_013992832.1	LTFQSWL--HTLNDIDLKLMRSAEE-----------------------------------	200
XP_029546688.1	LTFQSWL--HTLNDIDLKLMRSAEE-----------------------------------	200
XP_021481546.1	LTFQSWL--HTLNDIDLKLMRSAEE-----------------------------------	200
XP_020339889.1	LTFQSWL--HTLNDIDLKLMRSAEE-----------------------------------	200
XP_029481972.1	LTFQSWL--HTLNDIDLKLMRSAEE-----------------------------------	200
KPP68743.1	LTFQSWL--HIINDINITLIRTPDE-----------------------------------	177
XP_023669383.1	LTFQSWL--HIINDINITLMRTPEE-----------------------------------	199
KAA0720096.1	LTFQSWL--HTTKDINITLIRTPDE-----------------------------------	105
XP_009293684.1	LTFQSWL--HTTKDINITLMRSPEA-----------------------------------	199
XP_026090784.1	LTFQSWL--HTTKDINITLMRTPEE-----------------------------------	199
XP_018918715.1	LTFQSWL--HTTKDINITLMRTPEE-----------------------------------	199
KAF4098549.1	LTFQSWL--HTTKDINITLMRTPEE-----------------------------------	199
XP_016084173.1	LTFQSWL--HTTKDINITLMRTPEE-----------------------------------	199
XP_016332759.1	LTFQSWL--HTTKDINITLMRTPEE-----------------------------------	199

```
XP_016398039.1      LTFQSWL--HTTKDINITLMRTPEE-----------------------------------      199
XP_026989970.1      LTFQSWL--HTINDINITLMRTPQA-----------------------------------      199
TSQ12698.1          LTFQSWL--HTINDINITLMRTPEK-----------------------------------      199
XP_017347546.1      LTFQSWL--HTINDINITLMRTPQE-----------------------------------      194
XP_026794616.2      LTFQSWL--HTINDINITLMRTPQE-----------------------------------      199
XP_026861889.1      LTFQSWL--HTINDINITLMRSPEE-----------------------------------      199
XP_017575347.1      LTFQSWL--HTINDINITLMRSPEE-----------------------------------      199
XP_022531596.1      LTFQSWL--HTINDINITLMRSPEE-----------------------------------      199
XP_012691019.2      LTFQSWL--HTINDINITLMRSPED-----------------------------------      182
XP_028839723.1      LTFQSWL--HTINDINITLMRDPED-----------------------------------      199
XP_030638860.1      LTFQSWL--HTINDINITLMRSPEK-----------------------------------      181
XP_007882964.1      LTFTSWL--HHIQDINISLWRSPEL-----------------------------------      175
GCB70244.1          FTFTSWL--HNIRDINISLWRSPEV-----------------------------------      176
GCC26242.1          FTFTSWL--HNIQDINISLWRSAEL-----------------------------------      197
XP_020387034.1      FTFTSWL--HNIQDINISLWRSAEL-----------------------------------      188
XP_028931940.1      LTFTSWL--HTIRDINISLLRKPEN-----------------------------------      205
XP_031762490.1      LTFTSWL--HTIQDINVSLWRTPEE-----------------------------------      194
PIO32240.1          ------------------------------------------------------------      0
XP_018425466.1      LTFTSWL--HTIQDINVSLWRTPEE-----------------------------------      195
XP_006642351.1      LTFTSWL--HTTADINISLMRTPEE-----------------------------------      216
XP_028665372.1      LTFTSWL--HTISDINISLMRTPEE-----------------------------------      199
GCF49814.1          LTFTSWL--HNIRDINISLWRSEEA-----------------------------------      178
XP_015277816.1      LTFTSWL--HNIRDINISLWRSQEA-----------------------------------      201
XP_005987340.2      LTFTSWL--HIIQDINISLWRSPEQ-----------------------------------      183
XP_019339534.1      LTFTSWL--HNIQDINLSLWRHPDQ-----------------------------------      200
XP_025063414.1      LTFTSWL--HNIQDINLSLWRHPDQ-----------------------------------      200
XP_019367488.1      LTFTSWL--HNIRDINLSLWRHPEQ-----------------------------------      200
XP_019412078.1      LTFTSWL--HNIQDINLSLWRHPEQ-----------------------------------      200
XP_014434314.1      LTFTSWL--HNIRDINISLWRQPEL-----------------------------------      200
XP_007060556.1      LTFTSWL--HNIRDINISLWRQPEL-----------------------------------      194
XP_024072175.1      LTFTSWL--HNIRDINLSLWRQPEL-----------------------------------      179
XP_008170567.1      LTFTSWL--HNIRDINLSLWRQPEL-----------------------------------      200
XP_034610543.1      LTFTSWL--HNIRDINLSLWRQPEL-----------------------------------      200
XP_030394127.1      LTFTSWL--HNIRDINISLWRQPEL-----------------------------------      200
XP_032651960.1      LTFTSWL--HNIHDINISLWRQPEL-----------------------------------      200
XP_025020083.1      LTFTSWL--HNIRDINLSLWRTPEE-----------------------------------      200
XP_026536833.1      LTFTSWL--HNIRDINLSLWRTPEE-----------------------------------      200
XP_026564787.1      LTFTSWL--HNIRDINLSLWRTPEE-----------------------------------      200
XP_032084675.1      LTFTSWL--HNIRDINLSLWRTSEE-----------------------------------      185
XP_029139341.1      LTFTSWL--HNIRDINLSLWRTPEE-----------------------------------      200
XP_034281006.1      LTFTSWL--HNIRDINLSLWRTPEE-----------------------------------      200
XP_020649062.1      LTFTSWL--HNIRDINISLWRSQEE-----------------------------------      200
XP_008117087.1      LTFTSWL--HNIRDINISLWRSPEE-----------------------------------      200
XP_028564324.1      LTFTSWL--HNIRDINISLWRTPEE-----------------------------------      206
XP_033028155.1      LTFTSWL--HNIRDINISLWRTPEE-----------------------------------      205
XP_025963249.1      LTFTSWL--HNIRDLNLSLWRQPER-----------------------------------      187
XP_009668348.1      LTFTSWL--HNIRDLNLSLWRQPEL-----------------------------------      199
XP_013798935.1      LTFTSWL--HNIHDLNLSLWRQPEL-----------------------------------      183
XP_025913685.1      LTFTSWL--HNIHDLNLSLWRQPEL-----------------------------------      208
XP_013042552.1      LTFTSWL--HNIRDINLSLWRTPEL-----------------------------------      206
XP_005030458.2      LTFTSWL--HNIRDINLSLWRTPEL-----------------------------------      199
XP_032057953.1      LTFTSWL--HNIRDINLSLWRTPEL-----------------------------------      182
XP_021232050.1      LTFTSWL--HNIHDINLSLWRQPEL-----------------------------------      201
OXB62403.1          LTFTSWL--HNIHDINLSLWRQPEL-----------------------------------      200
OXB81319.1          LTFTSWL--HNIHDINLSLWRQPEL-----------------------------------      182
XP_010722007.1      LTFTSWL--HNIHDINLSLWRQPEL-----------------------------------      201
XP_015739349.1      LTFTSWL--HNIHDINLSLWRQPEL-----------------------------------      201
XP_031455498.1      LTFTSWL--HNIHDINLSLWRQPEL-----------------------------------      202
POI27435.1          LTFTSWL--HNIHDINLSLWRQPEL-----------------------------------      177
XP_004948120.1      LTFTSWL--HNIHDINLSLWRQPEL-----------------------------------      202
XP_032851190.1      LTFTSWL--HHIRDINLSLWRQPEL-----------------------------------      197
XP_010007255.1      LTFTSWL--HHVHDINLSLWRQPEL-----------------------------------      199
XP_030320702.1      LTFTSWL--HHIHDINLSLWRQPDL-----------------------------------      170
XP_010191940.1      LTFTSWL--HHIRDINLSLWRQPEL-----------------------------------      197
XP_027737112.1      LTFTSWL--HHIRDINLSLWRQPEL-----------------------------------      195
XP_027555032.1      LTFTSWL--HHIRDINLTLWRQPEL-----------------------------------      200
XP_032565370.1      LTFTSWL--HHIRDINLTLWRQPEL-----------------------------------      246
XP_027511217.1      LTFTSWL--HHIRDINLTLWRQPEL-----------------------------------      345
XP_027593499.1      LTFTSWL--HHIRDINLTLWRQPEL-----------------------------------      296
XP_017664924.1      LTFTSWL--HHIRDINLTLWRQPEL-----------------------------------      197
XP_029817938.1      LTFTSWL--HHIRDINLTLWRQPEL-----------------------------------      179
XP_005058641.1      LTFTSWL--HHIRDINLSLWRQPEL-----------------------------------      230
XP_021385804.1      LTFTSWL--HHIRDINLSLWRQPEL-----------------------------------      199
XP_030146687.2      LTFTSWL--HHIRDINLSLWRQPEL-----------------------------------      244
KAF4796420.1        LTFTSWL--HHIRDINLSLWRQPEL-----------------------------------      199
XP_032937581.1      LTFTSWL--HHIRDINLSLWRQPEL-----------------------------------      246
XP_031989659.1      LTFTSWL--HHIRDINLSLWRQPEL-----------------------------------      200
```

```
XP_010402086.1      LTFTSWL--HHIRDINLSLWRQPEL-----------------------------------      199
XP_017594069.1      LTFTSWL--HHIRDINLSLWRQPEL-----------------------------------      170
XP_014115268.1      LTFTSWL--HHIRDINLSLWRQPEL-----------------------------------      224
XP_023797108.1      LTFTSWL--HHIRDINLSLWRQPEL-----------------------------------      170
XP_033375614.1      LTFTSWL--HHIRDINLSLWRQPEL-----------------------------------      170
XP_014740121.1      LTFTSWL--HHIRDINLSLWRQPEL-----------------------------------      344
RLV83430.1          LTFTSWL--HHIRDINLSLWRQPEL-----------------------------------      190
XP_009096098.2      LTFTSWL--HHIRDINLSLWRQPEL-----------------------------------      305
TRZ15870.1          LTFTSWL--HHIRDINLSLWRQPEL-----------------------------------      200
RMB91935.1          LTFTSWL--HHIRDINLSLWRQPEL-----------------------------------      170
XP_030820843.1      LTFTSWL--HHIRDINLSLWRQPEL-----------------------------------      246
XP_014165179.1      LTFTSWL--HHIRDINLSLWRQPEL-----------------------------------      248
XP_026653582.1      LTFTSWL--HHIRDINLSLWRQPEL-----------------------------------      130
PKU35975.1          LTFTSWL--HHIRDINLSLWRQPEL-----------------------------------      202
XP_014805072.1      LTFTSWL--HHIRDINLSLWRQPEL-----------------------------------      196
XP_009818330.1      ------------------------------------------------------------      0
OPJ68307.1          LTFTSWL--HHIRDLNLSLWRQPEL-----------------------------------      199
XP_008936289.1      LTFTSWL--HHIRDINLSLGRQPEL-----------------------------------      200
XP_010287046.1      LTFTSWL--HHIRDINLSLWRQPXXXX---------------------------------      124
XP_005240140.2      LTFTSWL--HHIRDINLSLWRQPEL-----------------------------------      259
XP_005437752.2      LTFTSWL--HHIRDINLSLWRQPEL-----------------------------------      259
KFV74811.1          LTFTSWL--HHIRDINLSLWRQPEL-----------------------------------      164
XP_010018389.1      LTFTSWL--HHIRDINLSLWRQPEL-----------------------------------      207
KQK78711.1          LTFTSWL--HHIRDINLSLWRQPEL-----------------------------------      199
XP_009570162.1      LTFTSWL--HHIRDLNLSLWRQPEL-----------------------------------      198
KFP11268.1          LTFTSWL--HH-------SWSSPEL-----------------------------------      162
KFQ98910.1          LTFTSWL--HH--NINLALWRQPEL-----------------------------------      181
XP_012985202.3      LTFTSWL--HHIRDINLSLWRQPEL-----------------------------------      199
XP_010573388.1      LTFTSWL--HHIRDINLSLWRQPEL-----------------------------------      198
XP_029879496.1      LTFTSWL--HHIRDINLSLWRQPEL-----------------------------------      170
XP_030361086.1      LTFTSWL--HHIRDINLSLWRQPEL-----------------------------------      251
KFM00668.1          LTFTSWL--HHIRDINLSLWRQPGA-----------------------------------      183
KAF1479074.1        LTFTSWL--HHIRDINLSLWRQPEL-----------------------------------      162
KAF1651161.1        LTFTSWL--HHIRDINLSLWRQPEL-----------------------------------      163
KAF1673648.1        LTFTSWL--HHIRDINLSLWRQPEL-----------------------------------      163
KAF1493319.1        LTFTSWL--HHIRDINLSLWRQPEL-----------------------------------      163
KAF1584157.1        LTFTSWL--HHIRDINLSLWRQPEL-----------------------------------      163
KAF1571723.1        LTFTSWL--HHIRDINLSLWRQPEL-----------------------------------      163
KAF1533169.1        LTFTSWL--HHIRDINLSLWRQPEL-----------------------------------      163
KAF1638955.1        LTFTSWL--HHIRDINLSLWRQPEL-----------------------------------      163
KAF1549972.1        LTFTSWL--HHIRDINLSLWRQPEL-----------------------------------      163
KAF1606914.1        LTFTSWL--HHIRDINLSLWRQPEL-----------------------------------      163
KAF1510957.1        LTFTSWL--HHIRDINLSLWRQPEL-----------------------------------      163
KAF1498899.1        LTFTSWL--HHIRDINLSLWRQPEL-----------------------------------      163
KAF1411525.1        LTFTSWL--HHIRDINLSLWRQPEL-----------------------------------      163
KAF1429205.1        LTFTSWL--HHIRDINLSLWRQPEL-----------------------------------      163
XP_005334318.1      LTFNSIL--HTVNDINISLWRLPEK-----------------------------------      189
XP_014395552.1      LTFNSIL--HTVQDINISLWRPPEK-----------------------------------      189
KAB0404854.1        LTFTSWL--HTIQDINISLWRLPEK-----------------------------------      185
XP_007494828.1      LTFTSWL--HTIRDINITLWRLPEK-----------------------------------      200
XP_003764254.1      LTFTSWL--HTIRDINISLWRLPEK-----------------------------------      200
XP_020845489.1      LTFTSWL--HTIQDINISLWRLPEK-----------------------------------      200
XP_027703160.1      LTFTSWL--HTIRDINISLWRLPEK-----------------------------------      200
XP_017523929.1      LTFTSWL--HTIQDINISLWRLPEE-----------------------------------      198
XP_008688428.1      ---LVSS--LSVQDINISLWRLPEK-----------------------------------      152
XP_029812166.1      ---LVSP--RPVQDINISLWRLPEK-----------------------------------      214
XP_025749781.1      LTFTSWL--HTIQDINISLWRLPEK-----------------------------------      200
XP_004416432.1      LTFTSWL--HTIQDINISLWRLPEK-----------------------------------      200
XP_027436262.1      LTFTSWL--HTIQDINISLWRLPEK-----------------------------------      200
XP_032284025.1      LTFTSWL--HTIQDINISLWRLPEK-----------------------------------      200
XP_006735421.1      LTFTSWL--HTIQDINISLWRLPEK-----------------------------------      200
XP_021552166.1      LTFTSWL--HTIQDINISLWRLPEK-----------------------------------      200
XP_026361066.1      LTFTSWL--HTIQDINISLWRLPEK-----------------------------------      200
XP_034523598.1      LTFTSWL--HTIQDINISLWRLPEK-----------------------------------      200
NP_001297113.1      LTFTSWL--HTIQDINISLWRLPEK-----------------------------------      200
XP_032215488.1      LTFTSWL--HTIQDINISLWRLPEK-----------------------------------      200
VCX31483.1          LTFTSWL--HTIQDINISLWRLPEK-----------------------------------      200
XP_022369003.1      LTFTSWL--HTIQDINISLWRLPEK-----------------------------------      200
XP_032694248.1      LTFTSWL--HTIQDINISLWRLPEK-----------------------------------      200
NP_001041584.1      LTFTSWL--HTIQDINISLLRLPEK-----------------------------------      200
XP_025862501.1      LTFTSWL--HTIQDINISLLRLPEK-----------------------------------      200
KAF0873564.1        LTFTSWL--HTIQDINISLWRLPEK-----------------------------------      342
XP_025784751.1      LTFTSWL--HTIQDINISLWRLPEK-----------------------------------      200
XP_007075625.1      LTFTSWL--HTIQDINISLWRLPEK-----------------------------------      336
XP_030189489.1      LTFTSWL--HTIQDINISLWRLPEK-----------------------------------      200
XP_019324655.1      LTFTSWL--HTIQDINISLWRLPEK-----------------------------------      301
XP_023094886.1      LTFTSWL--HTIQDINISLWRLPEK-----------------------------------      336
```

```
XP_026892357.1      LTFTSWL--HTIQDINISLWRLPEK-----------------------------------     200
XP_016004457.1      LTFTSWL--HTIQDINISLWRLPEK-----------------------------------     195
XP_006912860.1      LTFTSWL--HTIQDINISLWRSPEK-----------------------------------     195
XP_011363679.1      LTFTSWL--HTIQDINISLWRSPEK-----------------------------------     195
XP_016076060.1      LTFTSWL--HTIQDINISLWRLPEK-----------------------------------     200
XP_008148371.1      LTFTSWL--HTIQDINISLWRLPEK-----------------------------------     200
XP_006093568.1      LTFTSWL--HTIQDINISLWRPPEK-----------------------------------     200
XP_006761601.1      LTFTSWL--HTIQDINISLWRPPEK-----------------------------------     200
XP_024426743.1      LTFTSWL--HTTQDINISLWRSPEK-----------------------------------     195
XP_019520779.1      LTFTSWL--HTIQDINISLWRLPEK-----------------------------------     200
XP_032976539.1      LTFTSWL--HTIQDINISLWRLPEK-----------------------------------     200
ELW64270.1          LTFTSWL--HTIQDINISLWRLPEK-----------------------------------     204
XP_004427367.1      LTFTSWL--HTIQDINITLWRLPEK-----------------------------------     200
XP_008529353.1      LTFTSWL--HTIQDINITLLRLPEK-----------------------------------     200
NP_001288165.1      LTFTSWL--HTIQDINITLLRLPEK-----------------------------------     200
XP_014711213.1      LTFTSWL--HTIQDINITLLRLPEK-----------------------------------     200
XP_012514621.1      LTFTSWL--HTIQDINVSLWRSPEK-----------------------------------     200
XP_008071525.1      LTFTSWL--HTIQDINVSLWRLPEE-----------------------------------     198
XP_009005082.1      LTFTSWL--HTIQDINISLWRSPEK-----------------------------------     200
XP_010332832.1      LTFTSWL--HTIQDINISLWRSPEK-----------------------------------     195
XP_012326058.1      LTFTSWL--HTIQDINISLWRSPEK-----------------------------------     206
XP_017378796.1      LTFTSWL--HTIQDINISLWRSPEK-----------------------------------     229
XP_032141374.1      LTFTSWL--HTIQDINISLWRSPEK-----------------------------------     229
XP_032024018.1      LTFTSWL--HTIQDINVSLWRLPEK-----------------------------------     195
XP_018891497.2      LTFTSWL--HTIQDINISLWRLPEK-----------------------------------     201
XP_030684894.1      LTFTSWL--HTIQDINISLWRLPEK-----------------------------------     195
PNJ75904.1          LTFTSWL--HTIQDINISLWRLPEK-----------------------------------     201
AAP35868.1          LTFTSWL--HTIQDINISLWRLPEK-----------------------------------     201
XP_001149570.1      LTFTSWL--HTIQDINISLWRLPEK-----------------------------------     201
XP_003805532.1      LTFTSWL--HTIQDINISLWRLPEK-----------------------------------     201
XP_023063823.1      LTFTSWL--HTIQDINISLWRLPEK-----------------------------------     200
XP_033060210.1      LTFTSWL--HTIQDINISLWRLPEK-----------------------------------     200
XP_010370669.1      LTFTSWL--HTIQDINISLWRLPEK-----------------------------------     200
XP_017738496.1      LTFTSWL--HTIQDINISLWRLPEK-----------------------------------     200
XP_011782168.1      LTFTSWL--HTIQDINISLWRLPEK-----------------------------------     200
EHH23440.1          LTFTSWL--HTIQDINISLWRLPEK-----------------------------------     195
XP_003910778.3      LTFTSWL--HTIQDINISLWRLPEK-----------------------------------     200
XP_025212649.1      LTFTSWL--HTIQDINISLWRLPEK-----------------------------------     206
XP_008019132.1      LTFTSWL--HTIQDINISLWRLPEK-----------------------------------     200
XP_011832421.1      LTFTSWL--HTIQDINISLWRLPEK-----------------------------------     185
XP_011759618.1      LTFTSWL--HTIQDINISLWRLPEK-----------------------------------     200
XP_005579749.1      LTFTSWL--HTIQDINISLWRLPEK-----------------------------------     200
XP_011921684.1      ------------------------------------------------------------     0
XP_008259391.1      LTFTSWL--HTIQDINITLWRLPEK-----------------------------------     200
XP_012782438.1      LTFTSWL--HTIQDINISLWRLPEE-----------------------------------     196
VTJ86076.1          LTFTSWL--HTINDINISLWRLPEK-----------------------------------     206
XP_015345533.1      LTFTSWL--HTINDINISLWRLPEK-----------------------------------     206
XP_027786432.1      LTFTSWL--HTINDINISLWRLPEK-----------------------------------     206
XP_026248345.1      LTFTSWL--HTINDINISLWRLPEK-----------------------------------     200
XP_005378209.1      LTFTSWL--HTIQDINISLWRLPEK-----------------------------------     200
XP_004466010.1      LTFTSWL--HTIQDINISLWRLPEK-----------------------------------     200
XP_020035707.1      LTFTSWL--HTIQDINVSLWRLPEK-----------------------------------     195
XP_006181802.1      LTFTSWL--HTIQDINISLLRLPET-----------------------------------     200
XP_006207862.1      LTFTSWL--HTIQDINISLLRLPET-----------------------------------     200
XP_005891968.1      LTFTSWL--HTIQDINISLWRLPEK-----------------------------------     197
XP_010828662.1      LTFTSWL--HTIQDINISLWRLPEK-----------------------------------     197
XP_019830126.1      LTFTSWL--HTIQDINISLWRLPEK-----------------------------------     196
XP_017914486.1      LTFTSWL--HTIQDINISLWRLPEK-----------------------------------     196
XP_006060266.2      LTFTSWL--HTIQDINISLWRLPEK-----------------------------------     201
XP_020728447.1      LTFTSWL--HNIQDINISLWRLPEK-----------------------------------     201
KAF4008892.1        LTFTSWL--HNIQDINISLWRLPEK-----------------------------------     201
KAB0348059.1        LTFTSWL--HNIQDINISLWRLPEK-----------------------------------     184
KAB0371113.1        LTFTSWL--HNIQDINISLWRLPEK-----------------------------------     177
MBV96963.1          LTFTSWL--HTIQDINISLWRLPEK-----------------------------------     200
XP_007172048.1      LTFTSWL--HTIQDINISLWRLPEK-----------------------------------     200
XP_007452270.1      LTFTSWL--HTIQDINISLWRLPEK-----------------------------------     200
XP_023987108.1      LTFTSWL--HTIQDINISLWRLPEK-----------------------------------     195
XP_004273432.1      LTFTSWL--HTIQDINLSLWRLPEK-----------------------------------     195
XP_004328646.1      LTFTSWL--HTIQDINLSLWRLPEK-----------------------------------     195
XP_026937670.1      LTFTSWL--HTIQDINLSLWRLPEK-----------------------------------     195
XP_030691031.1      LTFTSWL--HTIQDINLSLWRLPEK-----------------------------------     195
XP_022414870.1      LTFTSWL--HTIQDINLSLWRLPEK-----------------------------------     200
XP_029064651.1      LTFTSWL--HTIQDINLSLWRLPEK-----------------------------------     200
XP_024607566.1      LTFTSWL--HTIQDINLSLWRLPEK-----------------------------------     200
XP_032496907.1      LTFTSWL--HTIQDINLSLWRLPEK-----------------------------------     200
XP_007535559.1      LTFTSWL--HTIQDINISLWRPPEK-----------------------------------     193
XP_031299536.1      LTFTSWL--HTIQDINISLLRLPET-----------------------------------     199
```

```
XP_004666516.1    LTFTSWL--HTIHDINVSLWRLPEE-----------------------------------   195
XP_008830963.1    LTFTSWL--HTIQDINLSLWRSPED-----------------------------------   200
XP_005347338.1    LTFTSWL--HTIQDINISLWRSPEE-----------------------------------   200
XP_005069548.1    LTFTSWL--HSIQDINISLWRSPEE-----------------------------------   200
XP_027267894.1    LTFTSWL--HSIQDINISLWRSPEE-----------------------------------   200
OBS80992.1        LTFTSWL--HTIQDINISLWRSPEE-----------------------------------   187
XP_006979812.1    LTFTSWL--HTIQDINISLWRSPEE-----------------------------------   206
XP_028720687.1    LTFTSWL--HTIQDINISLWRSPEE-----------------------------------   200
XP_021489005.1    LTFTSWL--HTIQDINISLWRSPEE-----------------------------------   201
XP_031199589.1    LTFTSWL--HTIQDINISLWRSPED-----------------------------------   225
XP_028617944.1    ----------LVQDINISLWRTPED-----------------------------------   15
XP_034347030.1    LTFTSWL--HTIQDINISLWRTPEE-----------------------------------   198
NP_077370.2       LTFTSWL--HTIQDINISLWRTPEE-----------------------------------   200
XP_032766961.1    LTFTSWL--HTIQDINISLWRTPEE-----------------------------------   200
XP_021063186.1    LTFTSWL--HTIQDINISLWRSPEE-----------------------------------   183
NP_001093114.1    LTFTSWL--HTIQDINITLWRSPEE-----------------------------------   200
XP_021028379.1    LTFTSWL--HTIQDINITLWRSPEE-----------------------------------   200
XP_004689305.1    LTFTSWL--HTIQDINISLWRLPEK-----------------------------------   200
XP_004712871.1    LTFTSWL--HTIQDINISLWRNPEK-----------------------------------   200
XP_004382575.1    LTFTSWL--HTIQDINISLWRKPEK-----------------------------------   200
XP_010596188.1    LTFTSWL--HTIQDINISLWRKPEK-----------------------------------   200
XP_006890855.1    LTFTSWL--HTIKDINMSLWRKPEK-----------------------------------   200
XP_006834038.1    LTFTSWL--HTIQDINISLWRKPEK-----------------------------------   200
XP_007934716.1    LTFTSWL--HTIQDINISLWRKPEK-----------------------------------   200
XP_012603491.1    LTFTSWL--HTIQDINISLWRSPEK-----------------------------------   200
XP_012663606.1    LTFTSWL--HTIQDINISLWRSPEK-----------------------------------   195
XP_028372812.1    LTFTSWL--HTIQDINISLWRSPRK-----------------------------------   200
XP_008589608.1    LTFTSWL--HTIQDINITLWRLPEK-----------------------------------   200
XP_012865077.1    LTFTSWL--HTIQDINISLWRLPDK-----------------------------------   200
NP_001166178.1    LTFTSWL--HTIQDINISLWRLPEK-----------------------------------   200
XP_004636553.1    LTFTSWL--HTIQDINISLWRLPEK-----------------------------------   200
XP_004856670.1    LTFTSWL--HNIQDINISLWRLPEK-----------------------------------   200
XP_010627744.1    LTFTSWL--HTIQDVNISLWRLPEK-----------------------------------   200
```

```
                          Loop-F                                  Loop-C
NP_509270.1       -----LDT--FVPKKSKNSEWDIVSFN-----------------ATKMTTKY--GD-TL-   223
VDO93178.1        -----MTSDVFLEEYIENSEWTVESFK-----------------AERVVEMY--KC-CQ-   241
PAV91580.1        ------DPEVSLQSFIKNEEWDIISFN-----------------IYRHEYIY--AC-CP-   233
VDK46997.1        ------DPEVNLASFIPNEEWDVKSFK-----------------IFRHEYKY--AC-CA-   199
VIO86814.1        -----DQQEVFLNDFYNSQEWLLENAT-----------------ISNGTVEY--LE--Q-   258
VDP38785.1        ----SMNTTIMLNKLIENVELAVPELK-----------------LFKDYRIT--V--GG-   280
KOF68401.1        ----PASTTGDTSNYVQNSEWALVRLK-----------------AIRNEVKH--AS----   198
CDJ96026.1        ----LNTDKGDLSSYMNNSEFDLLDMI-----------------AIKEVVSF--PSNPL-   350
EFX76216.1        ----NQAEEGDLSNYVPNGEWDLIDLV-----------------VERNVVFY--SC-CE-   191
KAF7403848.1      ----KQSEKGDVTNYKANGEFDLVNFS-----------------ARRNIEYY--SC-CP-   203
KAF7427032.1      ----KQSEKGDVTNYKANGEFDLVNFS-----------------ARRNIEYY--SC-CP-   218
KAE9417558.1      -RGLNEKHKMDLLYYVPNGEFELVATP-----------------ADRVASVF--N---N-   206
KJH51504.1        --------EFDFSEYLTNGEWIILESK-----------------VNVSVKRY--EC-CP-   206
VDL69795.1        -------NGFDISE-----------TS-----------------AKRNIQHY--EC-CV-   187
VDO32671.1        -ENTNSSHSMDISTYVINGEWDLVSSP-----------------AIREVKYY--KC-CP-   221
VBB32409.1        -------GGFDISEYMPSGEWALPMTT-----------------VSRTVKFY--EC-CP-   198
VDN54565.1        -------GGFDISEYMPSGEWALPMTT-----------------VSRSEKFY--DC-CP-   217
EGT55171.1        -------GGFDISEYLPNGEWALPLTT-----------------VERNEKFY--DC-CP-   228
RCN52111.1        -------KGIDVSEYLPNGEWALPLTT-----------------VSRNEKFY--DC-CP-   233
RMX54856.1        ---VPEALLADTTKFVSNGEWELISFP-----------------AKRNVIYY--VC-CP-   229
XP_022781674.1    ---VPEALIADTSKFVSNGEWELISFP-----------------AKRNVIYY--VC-CP-   491
RNA37099.1        VDIMNRSENIDLSNYVPNGEWDLVRTY-----------------CVRRVVYY--PC-CR-   233
KAA0187152.1      VDVTNRTADVDLTNYISNGEWELLEAR-----------------IIRNVIYY--SC-CP-   406
GAU96593.1        VDVTNRTMEVDLKNYVKNGEWELLKIE-----------------VHRNVKYY--SC-CP-   231
XP_009043980.1    VDIVNRSDRVDLANYVYSGEWELIDVK-----------------VVRHEVYY--AC-CK-   192
VDM43573.1        VDITNRSDNVDLSNYVVSGEFDLVRVH-----------------QKRRVVKY--TC-CP-   310
XP_003140283.1    VDITNHSVNVDLSNYVESGEFDLVRVF-----------------QKRRIVKY--TC-CL-   201
VDN82010.1        VDITNRSINVDLSNYVESGEFDLVRVF-----------------QKRRVVKY--TC-CL-   227
VDO31501.1        VDITNRSINVDLSNYVESGEFDLVRVF-----------------QKRRVVKY--TC-CL-   229
VDP19591.1        ----TQVGTVDLSEYSRNGEFHLSGSS-----------------VRRYAQRY--EC-CD-   223
EDO32053.1        -------SGIDTENFSDNREWFLKGTS-----------------GRRDVKMY--IC-CP-   195
TRY67230.1        -------TSADLSGYVENGEWKLLGFP-----------------GYRHLSSF--D---G-   206
KAF7391312.1      -------KGGDLSDFIMNGEWYLIGMP-----------------GRKNTIVY--KC-CP-   220
GFG30449.1        -------NGGDISSFITNGEWDLLGVP-----------------GKRNEIYY--NC-CP-   230
KQS30083.1        -------AGGDISSFITNGEWDLLGVP-----------------GKRNEIYY--NC-CP-   252
EDW57583.2        -------TGGDISSYVLNGEWELLGVP-----------------GKRNEIYY--NC-CP-   388
KNC22799.1        -------TGGDISSYVLNGEWELLGVP-----------------GKRNEIYY--NC-CP-   198
RZF44856.1        -------GGGDISSFITNGEWDLLGVP-----------------GKRNEIYY--NC-CP-   218
ENN76856.1        -------NGGDISNFITNGEWDLLGVP-----------------GRRNEIFY--NC-CP-   224
KAF5300392.1      -------IGGDISNFITNGEWDLLGVP-----------------GKRNEIYY--NC-CP-   228
```

```
CAB3239999.1      -------GGGDISSFVTNGEWELIGVP-----------------GKRNEIYY--NC-CP-    215
PCG77624.1        -------GGGDISSFVTNGEWELIGVP-----------------GKRNEIYY--NC-CP-    158
PZC79131.1        -------GGGDISSFVTNGEWELIGVP-----------------GKRNEIYY--NC-CP-    225
KAF4083067.1      ------MSVNSKRYLEASGEWELVVIL-----------------GEAGILNF-GI-----    200
XP_009924659.1    ------MMKDSQSYFLTDGEWKFTNLS-----------------IIEYREEL-DD-----    223
XP_010123544.1    ------MMKDSQSYFLTDGEWKFTNLS-----------------IIEYTEEL-DN-----    222
CBN81618.1        ------IADLRHD--FIQYEWLFVNMT-----------------VSKMKDDS-TV-----    218
KAF3704230.1      ------ATEWSREVMRTQYEWLFINMN-----------------VTTN-NAS-DP-----    214
RUS86578.1        -----EDEGVDLDSYTINGEWEIISTS-----------------IKIDTG----------    236
XP_034309618.1    ----------ITEYFAENGEWNLVSSN-----------------MKMDEKN---------    225
PIK58946.1        -----VLASLE------IADWKFSNLT-----------------S----HNY-TIM-KPD    242
VUZ42516.1        ------IDSAMMDTYQPSNEWHIVVYP-----------------AVRRFFKQ--TC-CE-    226
PAA51166.1        TQGLEPNPYIDYTNYIRSQEWRTDGEDEPGIWPSRRRLQIRSVVRCRTVTFQ-SAD-GKQ    264
TGZ55997.1        DEGAVMSPFVDFSDYVPSNEWRTDGELEREINPLERKLQIRSVKRYRRRN-Q-TVG-NET    259
VDP48851.1        SPPIEEMPYLDFSDYVPSNEWRTDGEKEREVHHVNRTLQIRSVKKHRRRS-Q-TVG-NEV    215
PVD38331.1        ------KTAFDLDDYIESNEWEITQNT-----------------AKRHVKRY--TC-CP-    212
VDK73355.1        ENITVVEDGIDLSDYYPSVEWDIISRV-----------------AKRRTKNY--AS-CCP    203
VDD84963.1        DNVTIAEEGIDLSDYYPSVEWDIMSRV-----------------AKRRTKNY--PS-CCP    241
VDK42196.1        DNITIVEDGIDLSDYYPSVEWDIMSRV-----------------AKRRTKNY--PS-CCP    168
KFD58288.1        RMVWIVANGVDLSDFLPSAEWDIMAVP-----------------VKRHEERY--EC-CR-    248
OUC49089.1        RLVWIVPNGVDLSDFLPSTEWDVMAVP-----------------VKRHEERY--EC-CR-    245
EYC26492.1        ESVWIVNEGIDLSDYYPSVEWDILKVP-----------------GKRHSKRY--PC-CE-    271
VDK27218.1        ETIWVVDEGIDLSDYYPSVEWDVLGVP-----------------GKRHLKRY--PC-CE-    240
VDM17286.1        ------QTNIDLSDYVKSGMWDFVEGP-----------------VSSTHRRG--SV-SN-    147
VDL91846.1        TRPIYIDYAADLYEFNSTIEFEVLSMS-----------------ATCQERYY--KY-SH-    218
KAE9548540.1      SDVEFLEDGMDLSFYYKSAEWDLLQLT-----------------SARHSVLY--AS-CCG    238
PDM74087.1        -------KYIDLGEYSKSSIWDIVDAP-----------------AELAQQR---------    212
XP_024504322.1    ------ASFIDLSEYSPSSIWDVLDAP-----------------AALVKKR---------    215
PIO52336.1        ------AEWVDLSEYAPSSIWDVMDAP-----------------ASLVNKR---------    211
TKR73865.1        ------AEWVDVSEYSPSSIWDVVDAP-----------------ASLVNKR---------    216
RLU23395.1        ------KNFVDLSDYWKSGTWDIISVP-----------------AYLNTYQG--D---F-    222
XP_002427906.1    ------KNFVDLSDYWKSGTWDIIEVP-----------------AYLNIYNG--S---A-    223
TMW47392.1        ------KNFVDLSDYWKSGTWDIIEVP-----------------AYLNIYEG--DG-NH-    223
VDD83956.1        NCRSQIDHAIDLSMFTTDVQWDLIGVS-----------------ARRNIEFY--PC-CS-    235
VEL33078.1        SGMVEIDYAVDMRGFSSSVEFDILSVT-----------------GVREEHYP--AW-AN-    165
XP_009019088.1    DD--SIDMGIDLSEFYRSAEWDLMSAP-----------------ATKRNRSY--AG-SE-    227
KAE9536378.1      TN--KVDVGIDLSAYYPSVEWDILGVP-----------------AERHEKYY--SC-CA-    264
KDR23473.1        NN--KVEVGIDLREYYPSVEWDILGVP-----------------AERHEKYY--PC-CA-    213
KAF7266914.1      SN--IIKVGIDLSEFYLSVEWDILAVP-----------------ATRNEEYY--PD-SQ-    246
KAF4519445.1      SN--LVRIGIDLSEFYYSVEWDILDVP-----------------ARRNEEYY--PN-YK-    243
OXU25983.1        SN--LVAMGIDLTDFYLSVEWDILEVP-----------------ASRNEEYY--PC-CK-    231
TGZ32403.1        SN--IVAIGIDLTDFYLSVEWDILEVP-----------------AARNEEYY--PC-CE-    228
ELT94491.1        LD--VVEKGIDLTDYYKSTEWDLMKVP-----------------AKKDVKYY--PC-CA-    231
VVC39575.1        SR--VVDVGVDLSEFYASVEWDILEVP-----------------AIRNEKFY--TC-CE-    322
RVE49089.1        TN--IVELGVDLSEFYTSVEWDILEVP-----------------AVRNEKFY--TC-CD-    226
KAB0800277.1      SD--NIDVGIDLQDYYISVEWHIMRVP-----------------AVRNEKFY--SC-CE-    223
TDG52197.1        SD--NIEVGIDLQDYYISVEWDIMRVP-----------------AVRNEKFY--SC-CE-    209
TMW48669.1        SD--NIEVGIDLQDYYISVEWDIMRVP-----------------AVRNEKFY--SC-CE-    224
XP_025896085.1    -------VKADRSVFMNQGEWELLHVL-----------------SRIQEFSVK----DS-    228
KAF2977017.1      -------VKFDRSVFMNQGEWELLYVL-----------------SHFQEFSVK----SS-    32
XP_009321837.1    -------VKFDRSVFMNQGEWELLYVL-----------------SRFQEFSVK----SS-    207
XP_009979985.1    -------VKFDRSVFMNQGEWELLYVL-----------------SCFREFSVK----SS-    180
XP_028942374.1    -------VKFDRSVFMNQGEWELLYVL-----------------SHFQEFSVK----SS-    203
PKK19633.1        -------VKFDRSIFMNQGEWELLYVL-----------------SCFQEFSVK----SS-    270
XP_009894240.1    -------VKFDRSVFMNQGWWELLYVL-----------------SSFQEFSVK----SS-    323
XP_032820219.1    -------IKEDKSIFLNFGEWELISVP-----------------NELLHPLY-----GE-    256
XP_029429480.1    -------EED------NDGEWELCKVY-----------------HEYNIFEE-----NC-    259
XP_030077572.1    -------GKD------DKGEWEVCNVT-----------------HQYNIFNE-----SN-    206
XP_033774596.1    -------DRD------DKGEWEVCNVT-----------------HQRCEYKE-----SN-    237
ETE72600.1        -------VKNDRSVFMNQGEWELLHVL-----------------SQFREFSVG----DN-    259
XP_018082638.1    -------VKEDKSVFMNKGEWELLSVL-----------------SQYRKFVE-----NE-    439
XP_032905410.1    -------VKYDRSIFMNKGEWELLYIN-----------------TQFNEFNLEREV-TN-    226
XP_020773490.1    -------LREDKSVFMNQGEWELLHIL-----------------SNYKIFSV-DN---D-    232
XP_033833634.1    -------LREDKSVFMNQGEWELLHIL-----------------SNYKIFSV-DN---D-    258
XP_030215795.1    -------LKEDHSLFMNQGEWELLHVL-----------------SAYKSFSL-DN---D-    231
XP_030602980.1    -------LSKDKSVFMNQGEWELLHVL-----------------SKYKNFSV-DN---K-    237
XP_004573543.1    -------LSKDKSVFMNQGEWELLHVL-----------------SKYKNFSV-DK---E-    231
XP_005916159.1    -------LSKDKSVFMNQGEWELLHVL-----------------SKYKNFSV-DK---E-    231
XP_013132089.1    -------LSKDKSVFMNQGEWELLHVL-----------------SKYKNFSV-DK---E-    231
XP_031603488.1    -------LSKDKSVFMNQGEWELLHVL-----------------SKYKNFSV-DK---E-    237
XP_005725536.1    -------LSKDKSVFMNQGEWELLHVL-----------------SKYKNFSV-DK---E-    237
XP_026038075.1    -------LSKDKSVFMNQGEWELLHVL-----------------SKYKNFSV-DK---E-    237
CAF96649.1        -------LREDKSVFMNQGEWELLHIL-----------------SKYKSFSV-DN---D-    200
XP_023818570.1    -------LRDDKSVFMNQGEWELLHIL-----------------SKYKIFSV-DN---D-    227
RVE64289.1        -------LRDDKSVFMNQGEWELLHIL-----------------SKYKIFSV-DN---D-    262
XP_024121971.1    -------LRDDKSVFMNQGEWELLHIL-----------------SKYKIFSV-DN---D-    233
XP_015817559.1    -------LRDDKSVFMNQGEWELLHIL-----------------SNYKIFSV-DN---D-    217
```

```
XP_015225670.1     -------LRDDKSVFMNQGEWELLHIL-----------------SNYKIFSV-DN---D-     216
XP_012722063.1     -------LRDDKSVFMNQGEWELLHIL-----------------SNYKIFSV-DN---D-     231
XP_032431307.1     -------LRDDKSVFMNQGEWELLHIL-----------------SNYKIFSV-DN---D-     231
XP_014328329.1     -------LRDDKSVFMNQGEWELLHIL-----------------SNYKIFSV-DN---D-     231
XP_027886578.1     -------LRDDKSVFMNQGEWELLHIL-----------------SNYKIFSV-DN---D-     231
XP_008426791.1     -------LRDDKSVFMNQGEWELLHIL-----------------SNYKIFSV-DN---D-     231
XP_014838686.1     -------LRDDKSVFMNQGEWELLHIL-----------------SNYKIFSV-DN---D-     231
XP_014885967.1     -------LRDDKSVFMNQGEWELLHIL-----------------SNYKIFSV-DN---D-     231
XP_033954312.1     -------LREDKSVFMNQGEWELLHIL-----------------SNYKIFSV-DN---D-     231
XP_034089244.1     -------LREDKSVFMNQGEWELLHIL-----------------SNYKIFSV-DN---D-     231
XP_010780064.1     -------LREDKSVFMNQGEWELLHIL-----------------SNYKIFSV-DN---D-     247
XP_033970261.1     -------LREDKSVFMNQGEWELLHIL-----------------SNYKIFSV-DN---D-     247
XP_029375575.1     -------LSEDKSVFMNQGEWELLHVL-----------------SNYKSFSV-DD---D-     268
KAF0023022.1       -------LREDKSVFMNQGEWELLHVL-----------------SKYKIFSV-DN---D-     264
XP_019952614.1     -------LREDKSVFMNQGEWELLHIL-----------------SNYKIFSV-DN---D-     220
XP_034463117.1     -------LREDKSVFMNQGEWELLHIL-----------------SNYKIFSV-DN---D-     231
XP_029924656.1     -------LREDKSLFMNQGEWELLHVL-----------------SKYKSFSL-DD---D-     232
XP_028323228.1     -------LREDKSVFMNQGEWELLHIL-----------------SNYKIFSV-DN---D-     231
TNM84480.1         -------LREDKSVFMNQGEWELLHIL-----------------SKYKSFSV-DN---D-     231
XP_029703788.1     -------LREDKSVFMNQGEWELLHIL-----------------SKYKSFSV-DN---D-     231
XP_030010368.1     -------LREDKSVFMNQGEWELLHIL-----------------SKYKSFSV-DN---D-     234
XP_019725056.1     -------LREDKSVFMNQGEWELLHIL-----------------SNYKIFSV-DD---D-     232
XP_034034934.1     -------LREDKSVFMNQGEWELLHIL-----------------SQYKSFSL-DN---D-     220
XP_029956880.1     -------LREDKSVFMNQGEWELLHVL-----------------SNYKNFSV-DY---S-     231
XP_008331307.3     -------LREDKSVFMNQGEWELLHIL-----------------SNYKIFSV-DN---D-     264
XP_031724845.1     -------LREDKSVFMNQGEWELLHIL-----------------SNYKIFSV-DN---D-     232
XP_013859395.1     -------LRDDKSVFMNQGEWELLHIL-----------------SNYKIFSV-DN---B-     231
XP_017275335.1     -------LRDDKSVFMNQGEWELLHIL-----------------SHYKIFSV-DN---D-     231
XP_020496197.1     -------LSEDKSVFMNQGEWELLHIL-----------------SNYKIFSV-DN---D-     231
XP_029030400.1     -------LREDKSVFMNQGEWELLHIL-----------------SNYKIFSV-DN---D-     231
XP_022060538.1     -------LREDKSVFMNQGEWELLHIL-----------------SNYKIFSV-DD---D-     232
XP_023150584.1     -------LREDKSVFMNQGEWELLHIL-----------------SNYKIFSV-DD---D-     232
XP_029303892.1     -------LREDKSVFMNQGEWELLHIL-----------------SNYKIFSV-DN---D-     255
XP_020454172.1     -------LSEDKSVFMNQGEWELLHIL-----------------SKYKIFSV-DN---D-     230
TKS83244.1         -------LREDKRVFMNQGEWELLHIL-----------------SKYSNFSV-DN---D-     218
XP_022616583.1     -------LREDKSVFMNQGEWELLHIL-----------------SNYKSFSV-DD---D-     264
XP_030293172.1     -------LKEDKSVFMNQGEWELLHIL-----------------SNYKIFSV-DN---D-     231
XP_027129539.1     -------LREDKRVFMNQGEWELLHIL-----------------SKYSNFSV-DN---D-     231
XP_034539538.1     -------LSEDKSVFMNQGEWELLHIL-----------------SNYKIFSV-DN---D-     231
XP_008277330.1     -------LREDKSVFMNQGEWELLHIL-----------------SDYKIFSV-DN---D-     231
XP_028276940.1     -------LREDKSVFMNQGEWELLHIL-----------------SNYKIFSV-DN---D-     231
XP_023263433.1     -------LREDKSVFMNQGEWELLHVL-----------------SNYKSFSV-DD---D-     231
XP_034406336.1     -------LREDKSVFMNQGEWELLHIL-----------------SNYKIFSV-DN---D-     231
XP_026184460.1     -------LREDKSVFMNQGEWELLHIL-----------------SNYKIFSV-DN---D-     231
KAF1378228.1       -------LREDKSVFMNQGEWELLHIL-----------------SNYKIFSV-DN---D-     216
XP_028450365.1     -------LREDKSVFMNQGEWELLHIL-----------------SNYKIFSV-DN---D-     231
XP_031163851.1     -------LREDKSVFMNQGEWELLHIL-----------------SNYKIFSV-DN---D-     231
XP_032389084.1     -------LREDKSVFMNQGEWELLHIL-----------------SNYKIFSV-DN---D-     231
XP_033494682.1     -------LREDKSVFMNQGEWELLHIL-----------------SNYKIFSV-DN---D-     231
XP_018544782.1     -------LREDKSVFMNQGEWELLHIL-----------------SNYKIFSV-DN---D-     231
XP_026228189.1     -------LREDKSVFMNQGEWELLHIL-----------------SNYKIFSV-DN---D-     231
XP_028976606.1     -------VKVDKSVFMNQGEWELLHVL-----------------SKYKRFSL-DN---I-     231
CAB1352378.1       -------LKVDKSVFMNQGEWELLHVL-----------------SKYKRFSL-DN---I-     231
XP_023866849.1     -------LKVDKSVFMNQGEWELLHVL-----------------SKYKRFSL-DN---I-     231
XP_013992832.1     -------LKVDKSVFMNQGEWELLHVL-----------------SKYKRFSL-DN---I-     231
XP_029546688.1     -------LKVDKSVFMNQGEWELLHVL-----------------SKYKRFSL-DN---I-     231
XP_021481546.1     -------LKVDKSVFMNQGEWELLHVL-----------------SKYKRFSL-DN---I-     231
XP_020339889.1     -------LKVDKSVFMNQGEWELLHVL-----------------SKYKRFSL-DN---I-     231
XP_029481972.1     -------LKVDKSVFMNQGEWELLHVL-----------------SKYKRFSL-DN---I-     231
KPP68743.1         -------VKFDKSLFMNQGEWELLHIL-----------------SKYKSFSI-DE---N-     208
XP_023669383.1     -------VKVDKSVFMNQGEWELLHIL-----------------SKYKSFSI-DE---N-     230
KAA0720096.1       -------VKEDKNVFMNQGEWELLHVL-----------------SKYKQFSV-DS---N-     136
XP_009293684.1     -------VKTDKSVFMNQGEWELLHVL-----------------STYNAFSI-DN---D-     230
XP_026090784.1     -------VMNDKSVFMNQGEWELLHVL-----------------SVYNEFSI-DN---D-     230
XP_018918715.1     -------VMNDKSVFMNQGEWELLHVL-----------------STYKDFSV-DN---D-     230
KAF4098549.1       -------VMNDKSVFMNQGEWELLHVL-----------------STYKEFSI-DN---D-     230
XP_016084173.1     -------VMNDKSVFMNQGEWELLHVL-----------------STYKDFSI-DN---D-     230
XP_016332759.1     -------VMNDKSVFMNQGEWELLHVL-----------------STYKEFSI-DN---D-     230
XP_016398039.1     -------VMNDKSVFMNQGEWELLHVL-----------------STYKEFSI-DN---D-     230
XP_026989970.1     -------VKEDKSVFMNQGEWELLHVL-----------------SKYKSFSV-DC---K-     230
TSQ12698.1         -------VKEDKSVFMNQGEWELLHVL-----------------SKYKSFSV-DY---D-     230
XP_017347546.1     -------VKDDKSVFMNQGEWELLHVL-----------------SKYKSFSV-DN---D-     225
XP_026794616.2     -------VKEDKYVFMNQGEWELLHVL-----------------SKYKSFSV-DN---N-     230
XP_026861889.1     -------VKVDKTVFMNQGEWELLHIL-----------------SKYKSFSV-DN---D-     230
XP_017575347.1     -------VMVDKSVFMNQGEWELLHVL-----------------SKYKSFSV-DN---D-     230
XP_022531596.1     -------VKVDKSVFMNQGEWELLHVL-----------------SKYKCFSV-DN---D-     230
XP_012691019.2     -------VKEDKSVFMNQGEWELLHVL-----------------SKYNSFSL-DN---D-     213
```

```
XP_028839723.1      -------VKVDKSVFMNQGEWELLHVL-----------------SKYNSFSL-DN---D-      230
XP_030638860.1      -------VKEDKSVFMNQGEWELLHVL-----------------SKYKSFSL-DN---D-      212
XP_007882964.1      -------VMYDRSVFMNQGEWELLYVG-----------------SKYSIFNL-GNK-ES-      208
GCB70244.1          -------VKYDRSVFMNQGEWELLYVD-----------------TQFNEFNL-ERG-VS-      209
GCC26242.1          -------VKYDRSVFMNQGEWELLYVD-----------------TQFNEFNL-ERG-LS-      230
XP_020387034.1      -------VKYDRSVFMNQGEWELLYVD-----------------TQFNEFNL-ERG-LS-      221
XP_028931940.1      -------VKQDKTVFMNQGEWELLCVL-----------------PQFQEFSI-ES---N-      236
XP_031762490.1      -------VKEDKSLFMNNGEWELLSVL-----------------SQYRKFVE-N----E-      224
PIO32240.1          ---------------MNKGEWELLYVL-----------------PQYRMFVE-N----E-      22
XP_018425466.1      -------VKEDKSVFMNKGEWELLYVL-----------------PQYRKFVA-N----E-      225
XP_006642351.1      -------VMSDKNVFMNQGEWELLNVP-----------------SQYIDFSI-DG---T-      247
XP_028665372.1      -------VMFDKSVFMNQGEWELLHVL-----------------SKYSVFSI-DG---N-      230
GCF49814.1          -------VKTDKSVFMNQGEWELLHVL-----------------SYFQEFRV-DEA-SS-      211
XP_015277816.1      -------VKTDKSVFMNQGEWELLHVL-----------------SHFREFRV-EDA-SS-      234
XP_005987340.2      -------VKFDKSVFMNQGEWELLYVL-----------------TEYEAFSI-DS---K-      214
XP_019339534.1      -------VKFDRSVFLNQGEWELLCVL-----------------SHFREFSV-QG---S-      231
XP_025063414.1      -------VKFDRSVFLNQGEWELLCVL-----------------SHFREFSV-QG---S-      231
XP_019367488.1      -------VKFDRSVFLNQGEWELLCVL-----------------SHFREFSV-QG---S-      231
XP_019412078.1      -------VKFDRSVFLNQGEWELLCVL-----------------SHFREFSI-QD---S-      231
XP_014434314.1      -------VKFDKSVFMNQGEWELLYVL-----------------SQFQEFSV-EG---S-      231
XP_007060556.1      -------VKFDKSIFMNQGEWELLYVL-----------------SQFREFSV-EG---S-      225
XP_024072175.1      -------VKFDKSVFMNQGEWELLYVL-----------------SQFREFSV-EG---S-      210
XP_008170567.1      -------VKFDKSVFMNQGEWELLYVL-----------------SQFREFSV-EG---S-      231
XP_034610543.1      -------VKFDKSVFMNQGEWELLYVL-----------------SQFREFSV-EG---S-      231
XP_030394127.1      -------VKFDKSVFMNQGEWELLYVL-----------------SHFQEFSV-KG---S-      231
XP_032651960.1      -------VKFDKSVFMNQGEWELLYVL-----------------SQFQEFSV-KG---S-      231
XP_025020083.1      -------VKNDRSVFMNQGEWELLHVL-----------------SQFREFSV-GD---S-      231
XP_026536833.1      -------VKNDRSVFMNQGEWELLHVL-----------------SQFREFSV-GD---N-      231
XP_026564787.1      -------VKNDRSVFMNQGEWELLHVL-----------------SQFREFSV-GD---N-      231
XP_032084675.1      -------VKNDRSVFMNQGEWELLHVL-----------------SQFREFSV-GD---N-      216
XP_029139341.1      -------VKTDRSVFMNQGEWELLHVL-----------------SQFREFSV-GD---N-      231
XP_034281006.1      -------VKNDRSVFMNQGEWELLHVL-----------------SQFREFSV-GD---N-      231
XP_020649062.1      -------VKHDKSVFMNQGEWELLHVL-----------------PQFREFSV-GD---S-      231
XP_008117087.1      -------VKHDKSVFMNQGEWELLHVL-----------------SQFREFSV-GD---S-      231
XP_028564324.1      -------VKHDKSVFMNQGEWELLHVL-----------------SQFREFSV-GD---S-      237
XP_033028155.1      -------VKHDKSVFMNQGEWELLHVL-----------------SQFREFSV-GD---S-      236
XP_025963249.1      -------VKFDRNVFLNQGEWELLYVL-----------------SHFREFSV-EG---S-      218
XP_009668348.1      -------VKFDKSVFMNQGEWELLYVL-----------------SHFQEFSV-EG---S-      230
XP_013798935.1      -------VKFDKSVFMNQGEWELLYVL-----------------SHFQEFSV-EG---S-      214
XP_025913685.1      -------VKFDKSVFMNQGEWELLYVL-----------------SHFQEFSV-EG---S-      239
XP_013042552.1      -------VKFDRSIFMNQGEWELLYVL-----------------SHFQEFSV-KS---S-      237
XP_005030458.2      -------VKFDRSIFMNQGEWELLYVL-----------------SHFQEFSV-KS---S-      230
XP_032057953.1      -------VKFDRSIFMNQGEWELLYVL-----------------SHFQEFSV-KS---S-      213
XP_021232050.1      -------VKFDRSVFMNQGEWELLYVL-----------------SHFQEFSV-KS---S-      232
OXB62403.1          -------VKFDRSVFMNQGEWELLYVL-----------------SHFQEFSV-KS---S-      231
OXB81319.1          -------VKFDRSVFMNQGEWELLYVL-----------------SHFQEFSV-KS---S-      213
XP_010722007.1      -------VKFDRSVFMNQGEWELLYVL-----------------SHFQEFSV-KS---S-      232
XP_015739349.1      -------VKFDRSVFMNQGEWELLYVL-----------------SHFQEFSV-KS---S-      232
XP_031455498.1      -------VKFDRSVFMNQGEWELLYVL-----------------SHFQEFSV-KS---S-      233
POI27435.1          -------VKFDRSVFMNQGEWELLYVL-----------------SHFQEFSV-KS---S-      208
XP_004948120.1      -------VKFDRSVFMNQGEWELLYVL-----------------SHFQEFSV-KS---S-      233
XP_032851190.1      -------VKFDRSVFMNQGEWELLYVL-----------------SHFQEFSV-KS---S-      228
XP_010007255.1      -------VKFDRSVFMNQGEWELLYVL-----------------SHFQEFSV-KS---S-      230
XP_030320702.1      -------VKFDRSVFMNQGEWELLYVL-----------------THFQEFRV-KS---S-      201
XP_010191940.1      -------VKFDRSVFMNQGEWELLYVL-----------------SHFQEFSV-KS---S-      228
XP_027737112.1      -------VKFDQSVFMNQGEWELLYVL-----------------SHSQEFSV-KS---S-      226
XP_027555032.1      -------VKFDRSVFMNQGEWELLYVL-----------------SHFQEFSV-KS---S-      231
XP_032565370.1      -------VKFDRSVFMNQGEWELLYVL-----------------SHFQEFSV-KS---S-      277
XP_027511217.1      -------VKFDRSVFMNQGEWELLYVL-----------------SRFQEFSV-KS---S-      376
XP_027593499.1      -------VKFDRSVFMNQGEWELLYVL-----------------SHFQEFSV-KS---S-      327
XP_017664924.1      -------VKFDRSVFMNQGEWELLYVL-----------------SHFQEFSV-KS---S-      228
XP_029817938.1      -------VKFDRSVFMNQGEWELLYVL-----------------SHFQEFSV-KS---S-      210
XP_005058641.1      -------VKFDRSVFMNQGEWELLYVL-----------------SHFQEFSV-KS---S-      261
XP_021385804.1      -------VKFDRSVFMNQGEWELLYVL-----------------THFQEFSV-KS---S-      230
XP_030146687.2      -------VKFDRSVFMNQGEWELLYVL-----------------THFQEFSV-KS---S-      275
KAF4796420.1        -------VKFDRSVFMNQGEWELLYVL-----------------SRFQEFSV-KS---S-      230
XP_032937581.1      -------VKFDRSVFMNQGEWELLYVL-----------------SRFREFSV-KS---S-      277
XP_031989659.1      -------VKFDRSVFMNQGEWELLYVL-----------------SRFQEFSV-KS---S-      231
XP_010402086.1      -------VKFDRSVFMNQGEWELLYVL-----------------SRFQEFSV-KS---S-      230
XP_017594069.1      -------VKFDRSVFMNQGEWELLYVL-----------------SRFQEFSV-KS---S-      201
XP_014115268.1      -------VKFDRSVFMNQGEWELLYIL-----------------SHFQEFSV-KS---S-      255
XP_023797108.1      -------VKFDRSVFMNQGEWELLYIL-----------------SHFQEFSV-KS---S-      201
XP_033375614.1      -------VKFDRSVFMNQGEWELLYIL-----------------SHFQEFSV-KS---S-      201
XP_014740121.1      -------VKFDRSIFMNQGEWELLYVL-----------------SRFQEFSV-KS---S-      375
RLV83430.1          -------VKFDRSVFMNQGEWELLYVL-----------------THFQEFSV-KS---S-      221
XP_009096098.2      -------VKFDQSVFMNQGEWELLYVL-----------------SHFQEFSV-KS---S-      336
TRZ15870.1          -------VKFDRSVFMNQGEWELLYVL-----------------SHFQEFSV-KS---S-      231
```

RMB91935.1	-------VKFDRSVFMNQGEWELLYVL-----------------SHFQEFSV-KS---S-	201
XP_030820843.1	-------VKFDQSVFMNQGEWELLYVL-----------------SHFQEFSV-KS---S-	277
XP_014165179.1	-------VKFDQSVFMNQGEWELLYVL-----------------SHFQEFSV-KS---S-	279
XP_026653582.1	-------VKFDRSVFMNQGEWELLYVL-----------------SHFREFSV-KS---S-	161
PKU35975.1	-------VKFDRSVFMNQGEWELLYVL-----------------SSFQEFSV-KS---S-	233
XP_014805072.1	-------VKFDRSVFMNQGEWELLYVL-----------------SSFQEFSV-KS---S-	227
XP_009818330.1	--	0
OPJ68307.1	-------VKFDRSIFMNQGEWELLYVL-----------------SCFQEFSV-KS---S-	230
XP_008936289.1	-------VKFDRSVFMNQGEWELLYVL-----------------SHFQEFSV-KS---S-	231
XP_010287046.1	-XXXXXXKFDRSVFMNQGEWELLYVL-----------------SRFQEFSV-KS---S-	161
XP_005240140.2	-------VKFDRSVFMNQGEWELLYVL-----------------SHFQEFSV-KS---S-	290
XP_005437752.2	-------VKFDRSVFMNQGEWELLYVL-----------------SHFQEFSV-KS---S-	290
KFV74811.1	-------VKFDRSVFMNQGEWELLYVL-----------------SHFQEFSV-KS---S-	195
XP_010018389.1	-------VKFDRSVFMNQGEWELLYIL-----------------THFQEFSV-KS---S-	238
KQK78711.1	-------VKFDRSVFMNQGEWELLYVL-----------------THFQEFSV-KS---S-	230
XP_009570162.1	-------VKFDRSIFMNQGEWELLYIL-----------------SHFQEFSV-KS---S-	229
KFP11268.1	-------VKFDRSVFMNQGEWELLYVL-----------------SRFQEFSV-KS---S-	193
KFQ98910.1	-------VKFDRSVFMNQGEWELLYVL-----------------SCFQEFSV-KS---S-	212
XP_012985202.3	-------VKFDRSVFMNQGEWELLYVL-----------------THFQEFSV-KS---S-	230
XP_010573388.1	-------VKFDRSVFMNQGEWELLYVL-----------------SRFQEFSV-KS---S-	229
XP_029879496.1	-------VKFDRSVFMNQGEWELLYVL-----------------SRFQEFSV-KS---S-	201
XP_030361086.1	-------VKFDRSVFMNQGEWELLYVL-----------------THFQEFSV-KS---S-	282
KFM00668.1	-------GSL-TGVFMNQGEWELLYVL-----------------SRFQEFSV-KS---S-	213
KAF1479074.1	-------VKFDRSVFMNQGEWELLYVL-----------------SRFQEFSV-KS---S-	193
KAF1651161.1	-------VKFDRSVFMNQGEWELLYVL-----------------SRFQEFSV-KS---S-	194
KAF1673648.1	-------VKFDRSVFMNQGEWELLYVL-----------------SRFQEFSV-KS---S-	194
KAF1493319.1	-------VKFDRSVFMNQGEWELLYVL-----------------SRFQEFSV-KS---S-	194
KAF1584157.1	-------VKFDRSVFMNQGEWELLYVL-----------------SRFQEFSV-KS---S-	194
KAF1571723.1	-------VKFDRSVFMNQGEWELLYVL-----------------SRFQEFSV-KS---S-	194
KAF1533169.1	-------VKFDRSVFMNQGEWELLYVL-----------------SRFQEFSV-KS---S-	194
KAF1638955.1	-------VKFDRSVFMNQGEWELLYVL-----------------SRFQEFSV-KS---S-	194
KAF1549972.1	-------VKFDRSVFMNQGEWELLYVL-----------------SRFQEFSV-KS---S-	194
KAF1606914.1	-------VKFDRSVFMNQGEWELLYVL-----------------SRFQEFSV-KS---S-	194
KAF1510957.1	-------VKFDRSVFMNQGEWELLYVL-----------------SRFQEFSV-KS---S-	194
KAF1498899.1	-------VKFDRSVFMNQGEWELLYVL-----------------SRFQEFSV-KS---S-	194
KAF1411525.1	-------VKFDRSVFMNQGEWELLYVL-----------------SRFQEFSV-KS---S-	194
KAF1429205.1	-------VKFDRSVFMNQGEWELLYVL-----------------SRFQEFSV-KS---S-	194
XP_005334318.1	-------VKSDKSIFMNQGEWELLGVL-----------------PQFRKFTM-ES---S-	220
XP_014395552.1	-------VKFDKSVFMNQGEWELLGVL-----------------TQFREFSM-ES---S-	220
KAB0404854.1	-------VKLDRSIFMNQGEWELLGVL-----------------SQFQEFSI-ES---S-	216
XP_007494828.1	-------VKLDKSVFMNQGEWELLYVL-----------------TQFHEFSV-ES---S-	231
XP_003764254.1	-------VKLDKSVFMNQGEWELLYVL-----------------TQFREFSV-ES---S-	231
XP_020845489.1	-------VKLDKSVFMNQGEWELLYVL-----------------TQFREFSV-ES---S-	231
XP_027703160.1	-------VKLDKSVFMNQGEWELLYVL-----------------TQFREFSV-ES---S-	231
XP_017523929.1	-------VKSDKSVFMNQGEWELLGVL-----------------TQFREF-M-ES---S-	228
XP_008688428.1	-------VKLDKTLFMNQGEWELLGVL-----------------TQFREFSL-ED---S-	183
XP_029812166.1	-------VKVDKTIFMNQGEWELLAVL-----------------TQFREFSM-DS---S-	245
XP_025749781.1	-------VKLDKTLFMNQGEWELLGVL-----------------TQFREFSM-ED---S-	231
XP_004416432.1	-------VKLDKTLFMNQGEWELLGVL-----------------TQFREFSM-ED---S-	231
XP_027436262.1	-------VKLDKTLFMNQGEWELLGVL-----------------TQFREFSM-ED---S-	231
XP_032284025.1	-------VKLDKTLFMNQGEWELLGVL-----------------TQFREFSM-ED---S-	231
XP_006735421.1	-------VKLDKTLFMNQGEWELLGVL-----------------TQFREFSM-ED---S-	231
XP_021552166.1	-------VKLDKTLFMNQGEWELLGVL-----------------TQFREFSM-ED---S-	231
XP_026361066.1	-------VKLDKTLFMNQGEWELLGVL-----------------TQFREFSL-ED---S-	231
XP_034523598.1	-------VKLDKTLFMNQGEWELLGVL-----------------TQFREFSM-ED---S-	231
NP_001297113.1	-------VKLDKTVFMNQGEWELLGVL-----------------TQFREFSL-ED---S-	231
XP_032215488.1	-------VKLDKTVFMNQGEWELLGVL-----------------TQFREFSL-ED---S-	231
VCX31483.1	-------VKLDKTVFMNQGEWELLGVL-----------------TQFREFSM-ED---S-	231
XP_022369003.1	-------VKLDKTVFMNQGEWELLGVL-----------------TQFREFSM-ED---S-	231
XP_032694248.1	-------VKLDKTVFMNQGEWELLGVL-----------------TQFREFSM-ED---S-	231
NP_001041584.1	-------VKLDKTIFMNQGEWELLGVL-----------------TQFREFSM-ES---N-	231
XP_025862501.1	-------VKLDKTIFMNQGEWELLGVL-----------------TQFREFSM-ES---N-	231
KAF0873564.1	-------VKVDKTIFMNQGEWELLAVL-----------------TQFREFSM-ES---S-	373
XP_025784751.1	-------VKLDKTIFMNQGEWELLGVL-----------------TQFREFSM-ES---S-	231
XP_007075625.1	-------VKLDKTIFMNQGEWELLGVL-----------------TQFREFSM-ES---S-	367
XP_030189489.1	-------VKLDKTIFMNQGEWELLGVL-----------------TQFREFSM-ES---S-	231
XP_019324655.1	-------VKLDKTIFMNQGEWELLGVL-----------------TQFREFSM-ES---S-	332
XP_023094886.1	-------VKLDKTIFMNQGEWELLGVL-----------------TQFREFSM-ES---S-	367
XP_026892357.1	-------VKLDKTIFMNQGEWELLGVL-----------------TQFREFSM-ES---S-	231
XP_016004457.1	-------VKFDKSIFMNQGEWELLGVL-----------------TQFREFSM-ES---N-	226
XP_006912860.1	-------VKFDKSIFMNQGEWELLGVL-----------------TQFREFSM-ES---S-	226
XP_011363679.1	-------VKFDKSVFMNQGEWELLGVL-----------------TQFREFSM-ES---S-	226
XP_016076060.1	-------VKFDKSVFMNQGEWELLGVL-----------------TQFREFSM-ES---S-	231
XP_008148371.1	-------VKVDKSVFMNQGEWELLGVL-----------------TQFREFSM-ES---S-	231
XP_006093568.1	-------VKFDKSVFMNQGEWELLGVL-----------------TQFREFSM-ES---S-	231
XP_006761601.1	-------VKFDKSVFMNQGEWELLGVL-----------------TQFREFSM-ES---S-	231
XP_024426743.1	-------VKFDKSVFMNQGEWELLGVL-----------------TQFREFSM-ES---S-	226

XP_019520779.1	-------VKFDKSVFMNQGEWELLGVL-----------------TQFREFSI-ES---S-	231
XP_032976539.1	-------VKFDKSVFMNQGEWELLGVL-----------------TQFREFSI-ES---S-	231
ELW64270.1	-------VKFDKSVFMNQGEWELLGVL-----------------TQFREFSI-ES---S-	235
XP_004427367.1	-------VKFDKSVFMNQGEWELLGVL-----------------PQFQKFSL-ES---S-	231
XP_008529353.1	-------VKFDKSVFMNQGEWELLEVL-----------------PQFQKFSL-ES---S-	231
NP_001288165.1	-------VKFDKSVFMNQGEWELLEVL-----------------PQFQKFSL-ES---S-	231
XP_014711213.1	-------VKFDKSVFMNQGEWELLEVL-----------------PQFQKFSL-ES---S-	231
XP_012514621.1	-------VKSDKSVFMNQGEWELLGVL-----------------TQFREFSM-ES---S-	231
XP_008071525.1	-------VKFDKSVFMNQGEWELLGVL-----------------PQFREF-M-ES---S-	228
XP_009005082.1	-------VKFDKSVFMNQGEWELLGVL-----------------PYFREFSM-ES---S-	231
XP_010332832.1	-------VKFDKSVFMNQGEWELLGVL-----------------PYFREFSM-ES---S-	226
XP_012326058.1	-------VKFDKSVFMNQGEWELLVVL-----------------PYFREFSM-ES---S-	237
XP_017378796.1	-------VKFDKSVFMNQGEWELLGVL-----------------PYFREFSM-ES---S-	260
XP_032141374.1	-------VKFDKSVFMNQGEWELLGVL-----------------PYFREFSM-ES---S-	260
XP_032024018.1	-------VKSDRSVFMNQGEWELLGVL-----------------PYFREFSL-ES---S-	226
XP_018891497.2	-------VKSDRSVFMNQGEWELLGVL-----------------PYFREFTM-ES---S-	232
XP_030684894.1	-------VKSDRSVFMNQGEWELLGVL-----------------PYFREFSM-ES---S-	226
PNJ75904.1	-------VKFDRSVFMNQGEWELLGVL-----------------PYFREFSM-ES---S-	232
AAP35868.1	-------VKSDRSVFMNQGEWELLGVL-----------------PYFREFSM-ES---S-	232
XP_001149570.1	-------VKSDRSVFMNQGEWELLGVL-----------------PYFREFSM-ES---S-	232
XP_003805532.1	-------VKSDRSVFMNQGEWELLGVL-----------------PYFREFSM-ES---S-	232
XP_023063823.1	-------VKFDKSVFMNQGEWELLGVL-----------------PDFQEF-M-ES---S-	230
XP_033060210.1	-------VKFDRSVFMNQGEWELLGVL-----------------PDFQEF-M-ES---S-	230
XP_010370669.1	-------VKFDKSVFMNQGEWELLGVL-----------------PDFQEF-M-ES---S-	230
XP_017738496.1	-------VKFDKSVFMNQGEWELLGVL-----------------PDFQEF-M-ES---S-	230
XP_011782168.1	-------VKFDKSVFMNQGEWELLGVL-----------------PDFQEFSM-ES---S-	231
EHH23440.1	-------VKFDKSVFMNQGEWELLGVL-----------------PDFREFSM-ES---S-	226
XP_003910778.3	-------VKFDKSVFMNQGEWELLGVL-----------------PDFREFSM-ES---S-	231
XP_025212649.1	-------VKFDKSVFMNQGEWELLGVL-----------------PDFREFSM-ES---S-	237
XP_008019132.1	-------VKFDKSVFMNQGEWELLGVL-----------------PDFQEFSM-ES---S-	231
XP_011832421.1	-------VKFDKSVFMNQGEWELLGVL-----------------PDFREFSM-ES---S-	216
XP_011759618.1	-------VKFDKSVFMNQGEWELLGVL-----------------PDFREFSM-ES---S-	231
XP_005579749.1	-------VKFDKSVFMNQGEWELLGVL-----------------PDFREFSM-ES---S-	231
XP_011921684.1	---------------MNQGEWELLGVL-----------------PDFREFSM-ES---S-	23
XP_008259391.1	-------VKSDKSVFMNQGEWELLGVL-----------------TQYRRFSM-ES---S-	231
XP_012782438.1	-------VKSDKSVFMNQGEWELLAVL-----------------TQFRRFSM-ES---S-	227
VTJ86076.1	-------VKSDKSIFMNQGEWELLGVL-----------------PQFRKFTM-ES---S-	237
XP_015345533.1	-------VKSDKSIFMNQGEWELLGVL-----------------PQFRKFTM-ES---S-	237
XP_027786432.1	-------VKSDKSIFMNQGEWELLGVL-----------------PQFRKFTL-ES---S-	237
XP_026248345.1	-------VKSDKSIFMNQGEWELLGVL-----------------PQFRKFTM-ES---S-	231
XP_005378209.1	-------VKSDKSVFMNQGEWELLGVL-----------------TQFVQFSDGES---R-	232
XP_004466010.1	-------VKDDKSIFMNQGEWELLGVL-----------------AQFREFSI-ES---S-	231
XP_020035707.1	-------VKSDKSVFMNQGEWELLAVL-----------------TQFQEFS--ES---S-	225
XP_006181802.1	-------VKSDRSVFMNQGEWELLGVL-----------------TQFQEFSI-ES---S-	231
XP_006207862.1	-------VKSDRSVFMNQGEWELLGVL-----------------TRFQEFSI-ES---S-	231
XP_005891968.1	-------VKFDRSVFMNQGEWELLGVL-----------------TQFQEFSI-ES---S-	228
XP_010828662.1	-------VKFDRSVFMNQGEWELLGVL-----------------TQFQEFSI-ES---S-	228
XP_019830126.1	-------VKFDRSVFMNQGEWELLGVL-----------------TQFQEFSI-ES---S-	227
XP_017914486.1	-------VKFDRSVFMNQGEWELLGVL-----------------TQFQEFSI-ES---S-	227
XP_006060266.2	-------VKFDRSVFMNQGEWELLGVL-----------------TQFQEFSI-ES---S-	232
XP_020728447.1	-------VKFDRSVFMNQGEWELLGVL-----------------TQFQEFSI-ES---S-	232
KAF4008892.1	-------VKFDRSVFMNQGEWELLGVL-----------------TQFQEFSI-ES---S-	232
KAB0348059.1	-------VKFDRSVFMNQGEWELLGVL-----------------TQFQEFSI-ES---S-	215
KAB0371113.1	-------VKFDRSVFMNQGEWELLGVL-----------------TQFQEFSI-ES---S-	208
MBV96963.1	-------VKLDRSVFMNQGEWELLGVL-----------------SQFQEFSI-ES---S-	231
XP_007172048.1	-------VKLDRSIFMNQGEWELLGVL-----------------SQFQEFSI-ES---S-	231
XP_007452270.1	-------VKLDRSIFMNQGEWELLGVL-----------------TQFQEFSI-ES---S-	231
XP_023987108.1	-------VKLDRSIFMNQGEWELLGVL-----------------TQFQEFSI-ES---S-	226
XP_004273432.1	-------VKLDRSIFMNQGEWELLGVL-----------------TQFQEFSI-ES---S-	226
XP_004328646.1	-------VKLDRSIFMNQGEWELLGVL-----------------TQFQEFSM-ES---S-	226
XP_026937670.1	-------VKLDRSIFMNQGEWELLGVL-----------------TQFQEFSI-ES---S-	226
XP_030691031.1	-------VKLDRSIFMNQGEWELLGVL-----------------TQFQEFSI-ES---S-	226
XP_022414870.1	-------VKLDRSIFMNQGEWELLGVL-----------------TQFREFSI-ES---S-	231
XP_029064651.1	-------VKLDRSIFMNQGEWELLGVL-----------------TQFREFSI-ES---S-	231
XP_024607566.1	-------VKLDRSIFVNQGEWELLGVL-----------------TQFREFSI-ES---S-	231
XP_032496907.1	-------VKLDRSIFVNQGEWELLGVL-----------------TQFREFSI-ES---S-	231
XP_007535559.1	-------VESDKSIFMNQGEWELLGVL-----------------PQFQEFSI-ES---S-	224
XP_031299536.1	-------VKSDRSVFMNQGEWELLGVL-----------------TQFQEFSI-ES---S-	230
XP_004666516.1	-------VKSDKSVFMNQGEWELLGVL-----------------TQFREFSI-ES---S-	226
XP_008830963.1	-------VKLDHSVFMNQGEWELLGVL-----------------TQFQEFSM-KG---S-	231
XP_005347338.1	-------VRSDKSIFMNQGEWELLGVL-----------------PQFQEFSM-ET---S-	231
XP_005069548.1	-------VRSDKSVFMNQGEWELLGVL-----------------TQFQEFSM-KT---S-	231
XP_027267894.1	-------VRSDKSVFMNQGEWELLGVL-----------------TQFQEFSM-ET---S-	231
OBS80992.1	-------VRSDKXVFMNQGEWELLGVL-----------------TQFQEFSM-EI---S-	218
XP_006979812.1	-------VRSDKSIFMNQGEWELLGVL-----------------PQFQEFSM-ET---S-	237
XP_028720687.1	-------VRSDKSVFMNQGEWELLGVL-----------------TQFQEFSM-ET---S-	231
XP_021489005.1	-------VRSDKSIFMNQGEWELLGVL-----------------TQFQEFSI-ET---S-	232

```
XP_031199589.1      -------VRSDKSVFMNQGEWELLGVF-----------------TKFQEFSI-ET---S-     256
XP_028617944.1      -------VRSDKSIFKNQGEWELVGVF-----------------TQFQEFSL-ET---S-     46
XP_034347030.1      -------VRSDKSVFINQGEWELLGVF-----------------TQFQEFSI-ET---S-     229
NP_077370.2         -------VRSDKSIFINQGEWELLGVF-----------------TKFQEFSI-ET---S-     231
XP_032766961.1      -------VRSDKSVFINQGEWELLGVF-----------------TKFQEFSI-ET---S-     231
XP_021063186.1      -------VRSDKSIFINQGEWELLEVV-----------------PQFQEFSI-DI---S-     214
NP_001093114.1      -------VRSDKSIFINQGEWELLEVF-----------------PQFKEFSI-DI---S-     231
XP_021028379.1      -------VRSDKSIFINQGEWELLEVF-----------------PQFKEFSI-DI---S-     231
XP_004689305.1      -------VKSDKSVFMNQGEWELLAVL-----------------AQYREFSM-ES---S-     231
XP_004712871.1      -------VKYDKSVFMNQGEWELLGVL-----------------PQFQQFSI-EG---S-     231
XP_004382575.1      -------VKYDRSVFMNQGEWELLGVL-----------------TQFQKFSI-EA---S-     231
XP_010596188.1      -------VKYDRSVFMNQGEWELLGVL-----------------TQFQKFSI-EA---S-     231
XP_006890855.1      -------VKYDRSVFMNQGEWELLGVL-----------------TQFQEFSI-EG---S-     231
XP_006834038.1      -------VKYDRSVFMNQGEWELLGVL-----------------TQFQEFSI-EG---S-     231
XP_007934716.1      -------VKYDKSVFMNQGEWELLGVL-----------------TQFHEFSI-EG---S-     231
XP_012603491.1      -------VKSDKSVFMNQGEWELLGVL-----------------TQFQEFSM-ES---S-     231
XP_012663606.1      -------VKSDKSVFMNQGEWELLGVL-----------------TQFQEFSI-DG---S-     226
XP_028372812.1      -------GEFDKSVFMNQGEWELLGVR-----------------PSSGS-SVWKA---V-     231
XP_008589608.1      -------VKSDKSVFMNQGEWELLAVL-----------------TQYREFSI-ES---N-     231
XP_012865077.1      -------VKSDKSIFMNQGEWELLAVL-----------------TQFREFTM-ES---S-     231
NP_001166178.1      -------VKSDKSVFMNQGEWELLGVL-----------------TEFLEFSDRES---R-     232
XP_004636553.1      -------VKSDKSVFMNQGEWELLGVL-----------------TQFLEFSDRDS---S-     232
XP_004856670.1      -------VKSDKSVFMNQGEWELLGVL-----------------TQFLEFCDRES---R-     232
XP_010627744.1      -------VKSDKSVFMNQGEWELLGVL-----------------TKFLEFCDRES---R-     232
```

Loop-C

```
NP_509270.1         --GGFN-------------VYEEIFYYL----------------------EL-RRK----     241
VDO93178.1          --NPFT-------------L---IHADL----------------------II-KRK----     256
PAV91580.1          --EPWV-------------I---LQAHL----------------------VI-QRK----     248
VDK46997.1          --EPWV-------------I---LQASL----------------------VI-QRK----     214
VIO86814.1          --ESFS-------------T---VYMVL----------------------IL-RRE----     273
VDP38785.1          --QNIT-------------Q---INLRI----------------------HI-YRR----     295
KOF68401.1          --GVSS-------------E---LIYVI----------------------SL-ERK----     213
CDJ96026.1          --SKWP-------------T---IVVRI----------------------KM-HRR----     365
EFX76216.1          --EPYP-------------D---ITFHI----------------------VL-RRR----     206
KAF7403848.1        --EPYP-------------D---ITYEI----------------------RL-RRR----     218
KAF7427032.1        --EPYP-------------D---ITYEI----------------------RL-RRR----     233
KAE9417558.1        --EPYV-------------E---LYFRL----------------------HL-KRK----     221
KJH51504.1          --EEFE-------------D---IKFFL----------------------HL-RRR----     221
VDL69795.1          --EPYY-------------D---VVFTF----------------------VI-RRR----     202
VDO32671.1          --EPYP-------------T---VKYFL----------------------HI-RRR----     236
VBB32409.1          --EPYP-------------D---LKFYL----------------------HL-RRR----     213
VDN54565.1          --EPYP-------------D---LTFYL----------------------HM-RRR----     232
EGT55171.1          --EPYP-------------D---VHFYL----------------------HM-RRR----     243
RCN52111.1          --EPYP-------------D---LTFYL----------------------HM-RRR----     248
RMX54856.1          --EPYP-------------D---VTYTL----------------------RI-QRL----     244
XP_022781674.1      --EPYP-------------D---VTYTL----------------------RI-QRL----     506
RNA37099.1          --EPFP-------------D---VTFTL----------------------II-RRK----     248
KAA0187152.1        --EPFP-------------D---VTITI----------------------TI-RRK----     421
GAU96593.1          --EPFV-------------D---VTFRI----------------------HI-RRR----     246
XP_009043980.1      --EPYP-------------D---VRFTI----------------------II-RRK----     207
VDM43573.1          --EPYP-------------D---VTFFI----------------------HI-RRK----     325
XP_003140283.1      --EPYP-------------D---VTFYI----------------------HI-RRK----     216
VDN82010.1          --EPYP-------------D---VTFYI----------------------HI-RRK----     242
VDO31501.1          --EPYP-------------D---VTFYI----------------------HI-RRKVIRY     248
VDP19591.1          --YDFV-------------D---VKIAI----------------------TL-QRR----     238
EDO32053.1          --EPYP-------------T---VTYTI----------------------IL-RRR----     210
TRY67230.1          --SNYT-------------E---AIFKL----------------------NL-RRR----     221
KAF7391312.1        --EPYV-------------D---VTFTI----------------------QI-RRR----     235
GFG30449.1          --EPYI-------------D---ITFII----------------------II-RRR----     245
KQS30083.1          --EPYI-------------D---ITFAI----------------------LI-RRK----     267
EDW57583.2          --EPYI-------------D---ITFAI----------------------II-RRR----     403
KNC22799.1          --EPYI-------------D---ITFAI----------------------II-RRR----     213
RZF44856.1          --EPYI-------------D---ITFII----------------------II-RRR----     233
ENN76856.1          --EPYI-------------D---ITFVI----------------------II-RRR----     239
KAF5300392.1        --EPYI-------------D---ITFYI----------------------II-RRR----     243
CAB3239999.1        --EPYI-------------D---ITFAV----------------------VI-RRK----     230
PCG77624.1          --EPYI-------------D---ITFAV----------------------VI-RRK----     173
PZC79131.1          --EPYI-------------D---ITFAV----------------------VI-RRK----     240
KAF4083067.1        --DEWD-------------I---ITFWV----------------------VI-KRR----     215
XP_009924659.1      --EKFS-------------V---VTYVI----------------------SM-ERR----     238
XP_010123544.1      --GHFS-------------V---VTYVI----------------------SM-ERR----     237
CBN81618.1          --CGQS-------------M---IVYTI----------------------NM-KRR----     233
KAF3704230.1        --LGQD-------------I---VVYTI----------------------TM-KRR----     229
RUS86578.1          --EGLA-------------G---FRVEI----------------------VI-QRR----     251
```

```
XP_034309618.1     -------------------I---IRMCV----------------------VL-RRR----     236
PIK58946.1         FANHLP--------LVNIKI---AAVCL----------------------IL-ERN----     264
VUZ42516.1         --EPFP-------------N---LIFFF----------------------VL-RRN----     241
PAA51166.1         YNRTYK-------------T---LRYRV----------------------LM-RRS----     281
TGZ55997.1         MMREYP-------------V---LCYLI----------------------RL-RRN----     276
VDP48851.1         ITREYP-------------V---LRYLI----------------------RL-RRN----     232
PVD38331.1         --EPYP-------------D---LRFKL----------------------RI-RRR----     227
VDK73355.1         N-ETYI-------------D---IMYYL----------------------EL-RRK----     219
VDD84963.1         DTEAYI-------------D---IQYFL----------------------KL-RRK----     258
VDK42196.1         LSDAYI-------------D---IMYYL----------------------EL-RRK----     185
KFD58288.1         --APYI-------------D---ITFYI----------------------YL-RRR----     263
OUC49089.1         --APYI-------------D---ITFYI----------------------YL-RRR----     260
EYC26492.1         --SPFI-------------D---ITYEI----------------------HL-RRK----     286
VDK27218.1         --SPFI-------------D---LTYEI----------------------RL-RRK----     255
VDM17286.1         --------------RPTTAR---LTFKI----------------------VL-RRK----     163
VDL91846.1         --HAYP-------------E---VTFKL----------------------RM-RRR----     233
KAE9548540.1       P-ETYV-------------D---VTYYF----------------------FL-RRK----     254
PDM74087.1         ------------------SR---IQFNV----------------------II-RRK----     224
XP_024504322.1     ------------------SR---IEYQL----------------------KI-RRK----     227
PIO52336.1         ------------------SR---IEFQV----------------------RI-RRK----     223
TKR73865.1         ------------------SR---VEFQV----------------------RI-RRK----     228
RLU23395.1         ---------------PTETD---ITFYI----------------------II-RRK----     237
XP_002427906.1     ---------------PTETD---ITFYI----------------------II-RRK----     238
TMW47392.1         ---------------PTETD---ITFYI----------------------II-RRK----     238
VDD83956.1         --EPYL-------------D---ITFYI----------------------IL-RRK----     250
VEL33078.1         --VHYP-------------D---VTFRI----------------------RM-RRK----     180
XP_009019088.1     --EPFP-------------E---WTFNV----------------------TL-KRK----     242
KAE9536378.1       --EPYI-------------D---IFFNI----------------------TL-RRR----     279
KDR23473.1         --EPYP-------------D---IFFNI----------------------TL-RRK----     228
KAF7266914.1       --EPFS-------------D---ITFKI----------------------TM-RRK----     261
KAF4519445.1       --EPYS-------------D---ITFKL----------------------IM-RRK----     258
OXU25983.1         --EPYS-------------D---ITFNI----------------------KM-RRK----     246
TGZ32403.1         --EPYSESNLDNHNYRLISD---ITFNI----------------------TM-RRK----     256
ELT94491.1         --EPYP-------------D---IKFNI----------------------TI-RRK----     246
VVC39575.1         --EPYL-------------D---ITFNI----------------------TM-RRK----     337
RVE49089.1         --EPYL-------------D---ITFNI----------------------TM-RRK----     241
KAB0800277.1       --EPYP-------------D---IIFNI----------------------TL-RRK----     238
TDG52197.1         --EPYL-------------D---IVFNL----------------------TL-RRK----     224
TMW48669.1         --EPYL-------------D---IVFNL----------------------TL-RRK----     239
XP_025896085.1     --D-----------------SYAEIKFYV---------------------VI-RRR----     243
KAF2977017.1       --D-----------------SYAEMKFYV---------------------VI-RRR----     47
XP_009321837.1     --D-----------------SYAEMKFYV---------------------VI-RRR----     222
XP_009979985.1     --D-----------------SYAEMKFYV---------------------VI-RRR----     195
XP_028942374.1     --D-----------------SYAEMKFYV---------------------RVFGPH----     219
PKK19633.1         --D-----------------SYAEMKFYV---------------------VI-RRR----     285
XP_009894240.1     --D-----------------SYAEMKFYV---------------------VI-RRR----     338
XP_032820219.1     --N-----------------RFSELKIYV---------------------VI-RRR----     271
XP_029429480.1     --D-----------------SFAQLIYRV---------------------II-RRR----     274
XP_030077572.1     --D-----------------IFAELQFRI---------------------TI-RRR----     221
XP_033774596.1     --D-----------------TFAEMRFMI---------------------LI-RRR----     252
ETE72600.1         --D-----------------YYAEMKFFV---------------------VI-RRR----     274
XP_018082638.1     --D-----------------SFAEMKFHV---------------------VI-KRR----     454
XP_032905410.1     --D-----------------GYAEMRFYL---------------------VI-RRR----     241
XP_020773490.1     --D-----------------YYAEMKFHV---------------------VI-RRR----     247
XP_033833634.1     --D-----------------YYAEMKFHV---------------------VI-RRR----     273
XP_030215795.1     --D-----------------YYAEMKFHV---------------------VI-RRR----     246
XP_030602980.1     --E-----------------YYAEMKFHV---------------------VI-RRR----     252
XP_004573543.1     --E-----------------YYAEMKFHV---------------------VI-RRR----     246
XP_005916159.1     --E-----------------YYAEMKFHV---------------------VI-RRR----     246
XP_013132089.1     --E-----------------YYAEMKFHV---------------------VI-RRR----     246
XP_031603488.1     --E-----------------YYAEMKFHV---------------------VI-RRR----     252
XP_005725536.1     --E-----------------YYAEMKFHV---------------------VI-RRR----     252
XP_026038075.1     --E-----------------YYAEMKFHV---------------------VI-RRR----     252
CAF96649.1         --D-----------------YYAEMKFHVRFRPVPGALIKTRSNLGVRVQVVI-RRR----     237
XP_023818570.1     --D-----------------YYAEMKFHV---------------------VI-RRR----     242
RVE64289.1         --D-----------------YYAEMKFHV---------------------VI-RRR----     277
XP_024121971.1     --D-----------------YYAEMKFHV---------------------VI-RRR----     248
XP_015817559.1     --D-----------------YYAEMKFHV---------------------VI-RRR----     232
XP_015225670.1     --D-----------------YYAEMKFHV---------------------VI-RRR----     231
XP_012722063.1     --D-----------------YYAEMKFHV---------------------VI-RRR----     246
XP_032431307.1     --D-----------------YYAEMKFHV---------------------VI-RRR----     246
XP_014328329.1     --D-----------------YYAEMKFHV---------------------VI-RRR----     246
XP_027886578.1     --D-----------------YYAEMKFHV---------------------VI-RRR----     246
XP_008426791.1     --D-----------------YYAEMKFHV---------------------VI-RRR----     246
XP_014838686.1     --D-----------------YYAEMKFHV---------------------VI-RRR----     246
XP_014885967.1     --D-----------------YYAEMKFHV---------------------VI-RRR----     246
XP_033954312.1     --D-----------------YYAEMKFHV---------------------VI-RRR----     246
```

```
XP_034089244.1    --D----------------YYAEMKFHV----------------------VI-RRR----    246
XP_010780064.1    --D----------------YYAEMKFHV----------------------VI-RRR----    262
XP_033970261.1    --D----------------YYAEMKFHV----------------------VI-RRR----    262
XP_029375575.1    --D----------------YYAEMKFHV----------------------VI-RRR----    283
KAF0023022.1      --D----------------YYAEMKFHV----------------------VI-RRR----    279
XP_019952614.1    --D----------------YYAEMKFHV----------------------VI-RRR----    235
XP_034463117.1    --D----------------YYAEMKFHV----------------------VI-RRR----    246
XP_029924656.1    --D----------------YYAEMKFHV----------------------VI-RRR----    247
XP_028323228.1    --D----------------YYAEMKFHV----------------------VI-RRR----    246
TNM84480.1        --D----------------YYAEMKFHV----------------------VI-RRR----    246
XP_029703788.1    --D----------------YYAEMKFHV----------------------VI-RRR----    246
XP_030010368.1    --D----------------YYAEMKFHV----------------------VI-RRR----    249
XP_019725056.1    --D----------------YYAEMKFHV----------------------VI-RRR----    247
XP_034034934.1    --D----------------YYAEMKFHV----------------------VI-RRR----    235
XP_029956880.1    --D----------------YYAEMKFHV----------------------VI-RRR----    246
XP_008331307.3    --D----------------YYAEMKFHV----------------------VI-RRR----    279
XP_031724845.1    --D----------------YYAEMKFHV----------------------VI-RRR----    247
XP_013859395.1    --D----------------YYAEMKFHV----------------------VI-RRR----    246
XP_017275335.1    --D----------------YYAEMKFHV----------------------VI-RRR----    246
XP_020496197.1    --D----------------YYAEMKFHV----------------------VI-RRR----    246
XP_029030400.1    --D----------------YYAEMKFHV----------------------VI-RRR----    246
XP_022060538.1    --D----------------YYAEMKFHV----------------------VI-RRR----    247
XP_023150584.1    --D----------------YYAEMKFHV----------------------VI-RRR----    247
XP_029303892.1    --D----------------YYAEMKFHV----------------------VI-RRR----    270
XP_020454172.1    --D----------------YYAEMKFHV----------------------VI-RRR----    245
TKS83244.1        --D----------------HYAEMKFHV----------------------VI-RRR----    233
XP_022616583.1    --D----------------YYAEMKFHV----------------------VI-RRR----    279
XP_030293172.1    --D----------------YYAEMKFHV----------------------VI-RRR----    246
XP_027129539.1    --D----------------HYAEMKFHV----------------------VI-RRR----    246
XP_034539538.1    --D----------------YYAEMKFHV----------------------VI-RRR----    246
XP_008277330.1    --D----------------YYAEMKFHV----------------------VI-RRR----    246
XP_028276940.1    --D----------------YYAEMKFHV----------------------VI-RRR----    246
XP_023263433.1    --D----------------YYAEMKFHV----------------------VI-RRR----    246
XP_034406336.1    --D----------------YYAEMKFHV----------------------VI-RRR----    246
XP_026184460.1    --D----------------YYAEMKFHV----------------------VI-RRR----    246
KAF1378228.1      --D----------------YYAEMKFHV----------------------VI-RRR----    231
XP_028450365.1    --D----------------YYAEMKFHV----------------------VI-RRR----    246
XP_031163851.1    --D----------------YYAEMKFHV----------------------VI-RRR----    246
XP_032389084.1    --D----------------YYAEMKFHV----------------------VI-RRR----    246
XP_033494682.1    --D----------------YYAEMKFHV----------------------VI-RRR----    246
XP_018544782.1    --D----------------YYAEMKFHV----------------------VI-RRR----    246
XP_026228189.1    --D----------------YYAEMKFHV----------------------VI-RRR----    246
XP_028976606.1    --D----------------SYAEMKFNV----------------------VI-RRR----    246
CAB1352378.1      --D----------------SYAEMKFNV----------------------VI-RRR----    246
XP_023866849.1    --D----------------SYAEMKFNV----------------------VI-RRR----    246
XP_013992832.1    --D----------------SYAEMKFNV----------------------VI-RRR----    246
XP_029546688.1    --D----------------SYAEMKFNV----------------------VI-RRR----    246
XP_021481546.1    --D----------------SYAEMKFNV----------------------VI-RRR----    246
XP_020339889.1    --D----------------SYAEMKFNV----------------------VI-RRR----    246
XP_029481972.1    --D----------------SYAEMKFNV----------------------VI-RRR----    246
KPP68743.1        --D----------------HYAEMKFHV----------------------VI-RRR----    223
XP_023669383.1    --D----------------HYAEMKFHV----------------------VI-RRR----    245
KAA0720096.1      --E----------------YYAEMKFHV----------------------VI-RRR----    151
XP_009293684.1    --D----------------YYAEMKFHV----------------------VI-RRR----    245
XP_026090784.1    --D----------------YYAEMKFHV----------------------VI-RRR----    245
XP_018918715.1    --D----------------YYAEMKFHV----------------------VI-RRR----    245
KAF4098549.1      --D----------------YYAEMKFHV----------------------VI-RRR----    245
XP_016084173.1    --D----------------YYAEMKFHV----------------------VI-RRR----    245
XP_016332759.1    --D----------------YYAEMKFHV----------------------VI-RRR----    245
XP_016398039.1    --D----------------YYAEMKFHV----------------------VI-RRR----    245
XP_026989970.1    --D----------------YYAEMKFHV----------------------VI-RRR----    245
TSQ12698.1        --N----------------YYAEMKFHV----------------------VI-RRR----    245
XP_017347546.1    --D----------------YYAEMKFHV----------------------VI-RRR----    240
XP_026794616.2    --N----------------YYAEMKFHV----------------------VI-RRR----    245
XP_026861889.1    --D----------------YYAEMKFHV----------------------VI-RRR----    245
XP_017575347.1    --D----------------YYAEMKFHV----------------------VI-RRR----    245
XP_022531596.1    --D----------------YYAEMKFHV----------------------VI-RRR----    245
XP_012691019.2    --D----------------YYAEMKFHV----------------------VI-RRR----    228
XP_028839723.1    --D----------------YYAEMKFNV----------------------VI-RRR----    245
XP_030638860.1    --D----------------YYAEMKFHV----------------------VI-RRR----    227
XP_007882964.1    --D----------------GYSEMRFFL----------------------VI-RRR----    223
GCB70244.1        --D----------------GYAEMRFFL----------------------VI-RRR----    224
GCC26242.1        --V----------------GYAEMRFFL----------------------VI-RRR----    245
XP_020387034.1    --A----------------GYAEMRFFL----------------------VI-RRR----    236
XP_028931940.1    --N----------------SYAEMKFYV----------------------VI-RRR----    251
XP_031762490.1    --D----------------SFAEMKFHV----------------------VI-KRR----    239
PIO32240.1        --D----------------SFAEMKFHV----------------------VI-KRR----    37
```

```
XP_018425466.1      --D----------------SFAEMKFHV----------------------VI-KRR----      240
XP_006642351.1      --D----------------HYAEMKFFV----------------------VI-RRR----      262
XP_028665372.1      --D----------------HYAEMKFYV----------------------VI-RRR----      245
GCF49814.1          --E----------------SYAEMKFFV----------------------VI-RRR----      226
XP_015277816.1      --E----------------SYAEMKFFV----------------------VI-RRR----      249
XP_005987340.2      --D----------------SYAQMIFYV----------------------VI-RRR----      229
XP_019339534.1      --D----------------SYPEMKFYV----------------------VI-RRR----      246
XP_025063414.1      --D----------------SYPEMKFYV----------------------VI-RRR----      246
XP_019367488.1      --D----------------SYPEMKFYV----------------------VI-RRR----      246
XP_019412078.1      --D----------------SYPEMKFYV----------------------VI-RRR----      246
XP_014434314.1      --D----------------SYAEMKFYV----------------------VI-RRR----      246
XP_007060556.1      --D----------------SYAEMKFYV----------------------VI-RRR----      240
XP_024072175.1      --D----------------SYAEMKFYV----------------------VI-RRR----      225
XP_008170567.1      --D----------------SYAEMKFYV----------------------VI-RRR----      246
XP_034610543.1      --D----------------SYAEMKFYV----------------------VI-RRR----      246
XP_030394127.1      --D----------------SYAEMKFYV----------------------VI-RRR----      246
XP_032651960.1      --D----------------SYAEMKFYV----------------------VI-RRR----      246
XP_025020083.1      --D----------------HYAEMKFFV----------------------VI-RRR----      246
XP_026536833.1      --D----------------YYAEMKFFV----------------------VI-RRR----      246
XP_026564787.1      --D----------------YYAEMKFFV----------------------VI-RRR----      246
XP_032084675.1      --D----------------YYAEMKFFV----------------------VI-RRR----      231
XP_029139341.1      --D----------------YYAEMKFFV----------------------VI-RRR----      246
XP_034281006.1      --D----------------YYAEMKFFV----------------------VI-RRR----      246
XP_020649062.1      --D----------------YYAEMKFFV----------------------VI-RRR----      246
XP_008117087.1      --D----------------SYAEMKFFV----------------------VI-RRR----      246
XP_028564324.1      --D----------------FYAEMKFFV----------------------VI-RRR----      252
XP_033028155.1      --E----------------FYAEMKFFV----------------------VI-RRR----      251
XP_025963249.1      --D----------------SYAEMKFYV----------------------VI-RRR----      233
XP_009668348.1      --D----------------SYAEMKFYV----------------------VI-RRR----      245
XP_013798935.1      --D----------------SYAEMKFYV----------------------VI-RRR----      229
XP_025913685.1      --D----------------SYAEMKFYV----------------------VI-RRR----      254
XP_013042552.1      --D----------------SYAEMKFYV----------------------VI-RRR----      252
XP_005030458.2      --D----------------SYAEMKFYV----------------------VI-RRR----      245
XP_032057953.1      --D----------------SYAEMKFYV----------------------VI-RRR----      228
XP_021232050.1      --D----------------SYAEMKFYV----------------------VI-RRR----      247
OXB62403.1          --D----------------NYAEMKFYV----------------------VI-RRR----      246
OXB81319.1          --D----------------NYAEMKFYV----------------------VI-RRR----      228
XP_010722007.1      --D----------------SYAEMKFYV----------------------VI-RRR----      247
XP_015739349.1      --D----------------SYAEMKFYV----------------------VI-RRR----      247
XP_031455498.1      --D----------------SYAEMKFYV----------------------VI-RRR----      248
POI27435.1          --D----------------SYAEMKFYV----------------------VI-RRR----      223
XP_004948120.1      --D----------------SYAEMKFYV----------------------VI-RRR----      248
XP_032851190.1      --D----------------SYAEMKFYV----------------------VI-RRR----      243
XP_010007255.1      --D----------------SYAEMKFYV----------------------VI-RRR----      245
XP_030320702.1      --D----------------SYAEMKFYV----------------------VI-RRR----      216
XP_010191940.1      --D----------------SYAEMKFYV----------------------VI-RRR----      243
XP_027737112.1      --D----------------SYAEMKFYV----------------------VI-RRR----      241
XP_027555032.1      --D----------------HYAEMKFYV----------------------VI-RRR----      246
XP_032565370.1      --D----------------NYAEMKFYV----------------------VI-RRR----      292
XP_027511217.1      --D----------------NYAEMKFYV----------------------VI-RRR----      391
XP_027593499.1      --D----------------NYAEMKFYV----------------------VI-RRR----      342
XP_017664924.1      --D----------------NYAEMKFYV----------------------VI-RRR----      243
XP_029817938.1      --D----------------NYAEMKFYV----------------------VI-RRR----      225
XP_005058641.1      --D----------------SYAEMKFYV----------------------VI-RRR----      276
XP_021385804.1      --D----------------SYAEMKFYV----------------------VI-RRR----      245
XP_030146687.2      --D----------------SYAEMKFYV----------------------VI-RRR----      290
KAF4796420.1        --D----------------SYAEMKFYV----------------------VI-RRR----      245
XP_032937581.1      --D----------------SYAEMKFYV----------------------VI-RRR----      292
XP_031989659.1      --D----------------SYAEMKFYV----------------------VI-RRR----      246
XP_010402086.1      --D----------------SYAEMKFYV----------------------VI-RRR----      245
XP_017594069.1      --D----------------SYAEMKFYV----------------------VI-RRR----      216
XP_014115268.1      --D----------------SYAEMKFYV----------------------VI-RRR----      270
XP_023797108.1      --D----------------SYAEMKFYV----------------------VI-RRR----      216
XP_033375614.1      --D----------------SYAEMKFYV----------------------VI-RRR----      216
XP_014740121.1      --D----------------SYAEMKFYV----------------------VI-RRR----      390
RLV83430.1          --D----------------SYAEMKFYV----------------------VI-RRR----      236
XP_009096098.2      --D----------------SYAEMKFYV----------------------VI-RRR----      351
TRZ15870.1          --D----------------SYAEMKFYV----------------------VI-RRR----      246
RMB91935.1          --D----------------SYAEMKFYV----------------------VI-RRR----      216
XP_030820843.1      --D----------------SYAEMKFYV----------------------VI-RRR----      292
XP_014165179.1      --D----------------SYAEMKFYV----------------------VI-RRR----      294
XP_026653582.1      --D----------------SYAEMKFYV----------------------VI-RRR----      176
PKU35975.1          --D----------------SYAEMKFYV----------------------VV-RRR----      248
XP_014805072.1      --D----------------SYAEMKFYV----------------------VI-RRR----      242
XP_009818330.1      --------------------YAEMKFYV----------------------VI-RRR----      13
OPJ68307.1          --D----------------SYAEMKFYV----------------------VI-RRR----      245
XP_008936289.1      --D----------------SYAEMKFYV----------------------VI-RRR----      246
```

```
XP_010287046.1    --D----------------SYAEMKFYV----------------------VI-RRR----    176
XP_005240140.2    --D----------------SYAEMKFYV----------------------VI-RRR----    305
XP_005437752.2    --D----------------SYAEMKFYV----------------------VI-RRR----    305
KFV74811.1        --D----------------SYAEMKFYV----------------------VI-RRR----    210
XP_010018389.1    --D----------------SYAEMKFYV----------------------VI-RRR----    253
KQK78711.1        --D----------------SYAEXKFYV----------------------VI-RRR----    245
XP_009570162.1    --D----------------SYAEMKFYV----------------------VI-RRR----    244
KFP11268.1        --D----------------SYAEMKFYV----------------------VI-RRR----    208
KFQ98910.1        --D----------------SYAEMKFYV----------------------VI-RRR----    227
XP_012985202.3    --D----------------SYAEMKFYL----------------------VI-RRR----    245
XP_010573388.1    --D----------------SYAEMKFYV----------------------VI-RRR----    244
XP_029879496.1    --D----------------SYAEMKFYV----------------------VI-RRR----    216
XP_030361086.1    --D----------------SYAEMKFYV----------------------VI-RRR----    297
KFM00668.1        --D----------------SYAEMKFYV----------------------VI-RRR----    228
KAF1479074.1      --D----------------SYAEMKFYV----------------------VI-RRR----    208
KAF1651161.1      --D----------------SYAEMKFYV----------------------VI-RRR----    209
KAF1673648.1      --D----------------SYAEMKFYV----------------------VI-RRR----    209
KAF1493319.1      --D----------------SYAEMKFYV----------------------VI-RRR----    209
KAF1584157.1      --D----------------SYAEMKFYV----------------------VI-RRR----    209
KAF1571723.1      --D----------------SYAEMKFYV----------------------VI-RRR----    209
KAF1533169.1      --D----------------SYAEMKFYV----------------------VI-RRR----    209
KAF1638955.1      --D----------------SYAEMKFYV----------------------VI-RRR----    209
KAF1549972.1      --D----------------SYAEMKFYV----------------------VI-RRR----    209
KAF1606914.1      --D----------------SYAEMKFYV----------------------VI-RRR----    209
KAF1510957.1      --D----------------SYAEMKFYV----------------------VI-RRR----    209
KAF1498899.1      --D----------------SYAEMKFYV----------------------VI-RRR----    209
KAF1411525.1      --D----------------SYAEMKFYV----------------------VI-RRR----    209
KAF1429205.1      --D----------------SYAEMKFYV----------------------VI-RRR----    209
XP_005334318.1    --D----------------CYAEMKFYV----------------------VI-RRR----    235
XP_014395552.1    --S----------------CYAEMKFYV----------------------VI-RRR----    235
KAB0404854.1      --D----------------CYAEMKFYV----------------------VI-RRR----    231
XP_007494828.1    --D----------------SYAEMKFYV----------------------VI-RRR----    246
XP_003764254.1    --D----------------SYAEMKFYV----------------------VI-RRR----    246
XP_020845489.1    --E----------------SYAEMKFYV----------------------VM-RRR----    246
XP_027703160.1    --E----------------SYAEMKFYV----------------------VI-RRR----    246
XP_017523929.1    --D----------------CYAEMKFYV----------------------VI-RRR----    243
XP_008688428.1    --S----------------HYAEMKFYV----------------------VI-RRR----    198
XP_029812166.1    --G----------------CYAEMKFFV----------------------VI-RRR----    260
XP_025749781.1    --N----------------HYAEMKFYV----------------------VI-RRR----    246
XP_004416432.1    --N----------------HYAEMKFYV----------------------VI-RRR----    246
XP_027436262.1    --N----------------HYAEMKFYV----------------------VI-RRR----    246
XP_032284025.1    --N----------------HYAEMKFYV----------------------VI-RRR----    246
XP_006735421.1    --N----------------HYAEMKFYV----------------------VI-RRR----    246
XP_021552166.1    --N----------------HYAEMKFYV----------------------VI-RRR----    246
XP_026361066.1    --S----------------HYAEMKFYV----------------------VI-RRR----    246
XP_034523598.1    --S----------------HYAEMKFYV----------------------VI-RRR----    246
NP_001297113.1    --S----------------HYAEMKFYV----------------------VI-RRR----    246
XP_032215488.1    --S----------------HYAEMKFYV----------------------VI-RRR----    246
VCX31483.1        --S----------------HYAEMKFYV----------------------VI-RRR----    246
XP_022369003.1    --S----------------HYAEMKFYV----------------------VI-RRR----    246
XP_032694248.1    --S----------------HYAEMKFYV----------------------VI-RRR----    246
NP_001041584.1    --S----------------CYAEMKFYV----------------------VI-RRR----    246
XP_025862501.1    --S----------------CYAEMKFYV----------------------VI-RRR----    246
KAF0873564.1      --G----------------CYAEMKFFV----------------------VI-RRR----    388
XP_025784751.1    --G----------------CYAEMKFFV----------------------VI-RRR----    246
XP_007075625.1    --G----------------CYAEMKFFV----------------------VI-RRR----    382
XP_030189489.1    --G----------------CYAEMKFFV----------------------VI-RRR----    246
XP_019324655.1    --G----------------CYAEMKFFV----------------------VI-RRR----    347
XP_023094886.1    --G----------------CYAEMKFFV----------------------VI-RRR----    382
XP_026892357.1    --G----------------CYAEMKFFV----------------------VI-RRR----    246
XP_016004457.1    --E----------------CYAEMKFYV----------------------VI-RRR----    241
XP_006912860.1    --D----------------CYAEMKFYV----------------------VI-RRR----    241
XP_011363679.1    --D----------------CYAEMKFYV----------------------VI-RRR----    241
XP_016076060.1    --S----------------CYAEMKFYV----------------------VI-RRR----    246
XP_008148371.1    --S----------------CYAEMKFYV----------------------VI-RRR----    246
XP_006093568.1    --S----------------SYAEMKFYV----------------------VI-RRR----    246
XP_006761601.1    --D----------------CYAEMKFYV----------------------VI-RRR----    246
XP_024426743.1    --D----------------CYAEMKFYV----------------------VI-RRR----    241
XP_019520779.1    --S----------------CYAEMKFYV----------------------VI-RRR----    246
XP_032976539.1    --N----------------CYAEMKFYV----------------------VI-RRR----    246
ELW64270.1        --N----------------HYAEMKFYV----------------------VI-RRR----    250
XP_004427367.1    --N----------------YYAEMKFYV----------------------VI-RRR----    246
XP_008529353.1    --N----------------YYAEMKFYV----------------------VI-RRR----    246
NP_001288165.1    --N----------------YYAEMKFYV----------------------VI-RRR----    246
XP_014711213.1    --N----------------YYAEMKFYV----------------------VI-RRR----    246
XP_012514621.1    --D----------------HYAEMKFYV----------------------VI-RRR----    246
XP_008071525.1    --H----------------CYAEMKFHV----------------------VI-RRR----    243
```

```
XP_009005082.1    --H----------------CYAEMKFYV----------------------VI-RRR----    246
XP_010332832.1    --H----------------CYAEMKFYV----------------------VI-RRR----    241
XP_012326058.1    --D----------------CYAEMKFYV----------------------VI-RRR----    252
XP_017378796.1    --H----------------CYAEMKFYV----------------------VI-RRR----    275
XP_032141374.1    --H----------------CYAEMKFYV----------------------VI-RRR----    275
XP_032024018.1    --D----------------RYAEMKFYM----------------------VI-RRR----    241
XP_018891497.2    --N----------------YYAEMKFYV----------------------VI-RRR----    247
XP_030684894.1    --D----------------YYAEMKFYV----------------------VI-RRR----    241
PNJ75904.1        --D----------------YYAEMKFYV----------------------VI-RRR----    247
AAP35868.1        --N----------------YYAEMKFYV----------------------VI-RRR----    247
XP_001149570.1    --N----------------YYAEMKFYV----------------------VI-RRR----    247
XP_003805532.1    --N----------------YYAEMKFYV----------------------VI-RRR----    247
XP_023063823.1    --H----------------CYAEMKFYV----------------------VI-RRR----    245
XP_033060210.1    --H----------------CYAEMKFYV----------------------VI-RRR----    245
XP_010370669.1    --H----------------CYAEMKFYV----------------------VI-RRR----    245
XP_017738496.1    --H----------------CYAEMKFYV----------------------VI-RRR----    245
XP_011782168.1    --H----------------CYAEMKFYV----------------------VI-RRR----    246
EHH23440.1        --H----------------CYAEMKFYV----------------------VI-RRR----    241
XP_003910778.3    --H----------------YYAEMKFYV----------------------VI-RRR----    246
XP_025212649.1    --H----------------YYAEMKFYV----------------------VI-RRR----    252
XP_008019132.1    --H----------------CYAEMKFYV----------------------VI-RRR----    246
XP_011832421.1    --H----------------CYAEMKFYV----------------------VI-RRR----    231
XP_011759618.1    --H----------------CYAEMKFYV----------------------VI-RRR----    246
XP_005579749.1    --H----------------CYAEMKFYV----------------------VI-RRR----    246
XP_011921684.1    --H----------------CYAEMKFYV----------------------VI-RRR----    38
XP_008259391.1    --D----------------CYAEMKFYV----------------------VI-RRR----    246
XP_012782438.1    --D----------------CYAEMKFYV----------------------VI-RRR----    242
VTJ86076.1        --D----------------CYAEMKFYV----------------------VI-RRR----    252
XP_015345533.1    --D----------------CYAEMKFYV----------------------VI-RRR----    252
XP_027786432.1    --D----------------CYAEMKFYV----------------------VI-RRR----    252
XP_026248345.1    --D----------------CYAEMKFYV----------------------VI-RRR----    246
XP_005378209.1    --G----------------SYAEMKFYV----------------------VI-RRR----    247
XP_004466010.1    --D----------------CYAEMKFYV----------------------VI-RRR----    246
XP_020035707.1    --D----------------SYAEMKFYV----------------------VI-RRR----    240
XP_006181802.1    --H----------------HYAEMKFYV----------------------VI-RRR----    246
XP_006207862.1    --H----------------HYAEMKFYV----------------------VI-RRR----    246
XP_005891968.1    --D----------------SYAEMKFYV----------------------VI-RRR----    243
XP_010828662.1    --D----------------SYAEMKFYV----------------------VI-RRR----    243
XP_019830126.1    --D----------------SYAEMKFYV----------------------VI-RRR----    242
XP_017914486.1    --D----------------SYAEMKFYV----------------------VI-RRR----    242
XP_006060266.2    --D----------------SYAEMKFYV----------------------VI-RRR----    247
XP_020728447.1    --D----------------SYAEMKFYV----------------------VI-RRR----    247
KAF4008892.1      --D----------------SYAEMKFYV----------------------VI-RRR----    247
KAB0348059.1      --D----------------SYAEMKFYV----------------------VI-RRR----    230
KAB0371113.1      --D----------------SYAEMKFYV----------------------VI-RRR----    223
MBV96963.1        --D----------------CYAEMKFYV----------------------VI-RRR----    246
XP_007172048.1    --D----------------CYAEMKFYV----------------------VI-RRR----    246
XP_007452270.1    --D----------------CYAEMKFYV----------------------VI-RRR----    246
XP_023987108.1    --D----------------CYAEMKFYV----------------------VI-RRR----    241
XP_004273432.1    --D----------------CYAEMKFYV----------------------VI-RRR----    241
XP_004328646.1    --D----------------CYAEMKFYV----------------------VI-RRR----    241
XP_026937670.1    --D----------------CYAEMKFYV----------------------VI-RRR----    241
XP_030691031.1    --D----------------CYAEMKFYV----------------------VI-RRR----    241
XP_022414870.1    --D----------------CYAEMKFYV----------------------VI-RRR----    246
XP_029064651.1    --D----------------CYAEMKFYV----------------------VI-RRR----    246
XP_024607566.1    --D----------------CYAEMTFYV----------------------VI-RRR----    246
XP_032496907.1    --D----------------CYAEMKFYV----------------------VI-RRR----    246
XP_007535559.1    --S----------------CYAEMKFHV----------------------VI-RRR----    239
XP_031299536.1    --H----------------HYAEMKFYV----------------------VI-RRR----    245
XP_004666516.1    --D----------------CYAEMKFYV----------------------VI-RRR----    241
XP_008830963.1    --D----------------CYAEMKFYV----------------------VI-RRR----    246
XP_005347338.1    --S----------------SYAEMKFYV----------------------VI-RRR----    246
XP_005069548.1    --Y----------------SYAEMKFYV----------------------VI-RRR----    246
XP_027267894.1    --Y----------------SYAEMKFYV----------------------VI-RRR----    246
OBS80992.1        --S----------------SYAEMKFYV----------------------VI-RRR----    233
XP_006979812.1    --N----------------SYAEMKFYV----------------------VI-RRR----    252
XP_028720687.1    --N----------------SYAEMKFYV----------------------VI-RRR----    246
XP_021489005.1    --S----------------SYAEMKFYV----------------------VI-RRR----    247
XP_031199589.1    --N----------------SYAEMKFYV----------------------VI-RRR----    271
XP_028617944.1    --N----------------SYAEMKFYV----------------------VI-RRR----    61
XP_034347030.1    --S----------------SYAEMKFYV----------------------VI-RRR----    244
NP_077370.2       --N----------------SYAEMKFYV----------------------VI-RRR----    246
XP_032766961.1    --N----------------SYAEMKFYV----------------------VI-RRR----    246
XP_021063186.1    --N----------------SYAEMKFYV----------------------II-RRR----    229
NP_001093114.1    --N----------------SYAEMKFYV----------------------II-RRR----    246
XP_021028379.1    --N----------------SYAEMKFYV----------------------II-RRR----    246
XP_004689305.1    --D----------------SYAEMKFYV----------------------VI-RRR----    246
```

```
XP_004712871.1      --E----------------YYAEMKFHV----------------------VI-RRR----    246
XP_004382575.1      --S----------------CYAEMKFYV----------------------VI-RRR----    246
XP_010596188.1      --N----------------YYAEMKFYV----------------------VI-RRR----    246
XP_006890855.1      --E----------------CYAEMKFYV----------------------VI-RRR----    246
XP_006834038.1      --E----------------CYAEMKFYV----------------------VI-RRR----    246
XP_007934716.1      --E----------------CYAEMKFYV----------------------VI-RRR----    246
XP_012603491.1      --G----------------RYAEMKFYV----------------------VI-RRR----    246
XP_012663606.1      --I----------------YYAEMKFYV----------------------VI-RRR----    241
XP_028372812.1      --T----------------GYAEMKFXV----------------------VI-RRR----    246
XP_008589608.1      --D----------------CFAEMKFSV----------------------VI-RRR----    246
XP_012865077.1      --D----------------CYAEMKFYL----------------------VI-RRR----    246
NP_001166178.1      --G----------------SFAEMKFYV----------------------VI-RRR----    247
XP_004636553.1      --G----------------SFAEMKFYV----------------------VI-RRR----    247
XP_004856670.1      --G----------------CYAEMKFYV----------------------VI-RRR----    247
XP_010627744.1      --G----------------CYAEMKFYV----------------------VI-RRR----    247
                                               .                       :

                              Transmembrane domain 1         Transmembrane domain 2

NP_509270.1         ------PLYYIVVILLPSFLIVTVSNIGLFTPHGVHGDREEHVSLGLTTMLTMAVILDMV    295
VDO93178.1          ------PLFTLVNLVIPTAIINLISLFGFFSPTTTNGERTEKVNLGITTLLAMSILLLMV    310
PAV91580.1          ------PLYYLVNLIIPTSIITIVSITGFFTPASTDDDRTEKINLGITTLLAMSILMLMV    302
VDK46997.1          ------PLYYIVNLIIPTSIITIVSITGFFTPASTDDDRTEKINLGITTLLAMSILMLMV    268
VIO86814.1          ------SFYYVFNFVFPTTLVSIVAVIGFHAPINATGRHENKFRLGIMTLLSMSVMLLVL    327
VDP38785.1          ------AIFYTYTVIAPSILLCILTVFSFWLPSGN----VKKIDMGLTVFLFLYFLQVMI    345
KOF68401.1          ------PFFHILSFIFPCLLISSISLLGFLLPPSS----GEKVSIGVTVLLSLSVFLLVV    263
CDJ96026.1          ------PLFYIFNHIIPCVLISSMAVLGFLMPPET----GEKINMIITTLLSMGVYLQSI    415
EFX76216.1          ------PLFYVFNLILPCVLITGIALMSFYMPSDS----GEKVTLGITTLLSMTVFLMVI    256
KAF7403848.1        ------SMFYVFNLILPCILINGIALLVFYVPSES----GEKVTLGISALLSMTVFLMTI    268
KAF7427032.1        ------SMFYVFNLILPCILINGIALLVFYVPSES----GEKVTLGISALLSMTVFLMTI    283
KAE9417558.1        ------TLYYGLNWIVPSILISISNVLGFTMPPEC----GEKITLQITNLLSVTVFLGMV    271
KJH51504.1          ------TLYYAFNLIMPCFLTMILMVLGFTLSPET----CEKVGLQISVSLAICIFLTIM    271
VDL69795.1          ------VLYYAFNLILPCILITMLTLIGFTLPPDA----GEKMSLQITIMLSICIFQNYV    252
VDO32671.1          ------TLYYGFNLIIPSLLICFMTVLGFSLPPDA----GEKITLEMTILLAIVFFLSMV    286
VBB32409.1          ------TLYYGFNLIMPCILTTMMTLLGFTLPPDA----GEKITLQITVLLSICFFLSVV    263
VDN54565.1          ------TLYYGFNLIMPCILTTMMTLLGFTLPPDA----GEKITLQITVLLSICFFLSIV    282
EGT55171.1          ------TLYYGFNLIMPCILTTLMTLLGFTLPPDA----GEKITLQITVLLSICFFLSIV    293
RCN52111.1          ------TLYYGFNLIMPCILTTLMTLLGFTLPPDA----GEKITLQITVLLSICFFLSIV    298
RMX54856.1          ------ALFYYMNLIIPCLLITVLTVTAFYLPPDS----GERITLVITNLLAMTVFMLLV    294
XP_022781674.1      ------ALFYYMNLMIPCLLITVLTVTAFYLPPDS----GERITLVITNLLAMTVFMLLV    556
RNA37099.1          ------VLYFTVNVILPCLMLSALTVLVFVIPSDS----GDKVTLGITVFLAFSVIMLAV    298
KAA0187152.1        ------TLYYMYNVVLPCMMMSVLTLLVFCLPPDS----GEKIALGVTVLLAFSVFMLAI    471
GAU96593.1          ------VLYYTYNVILPCVMMSALTLLVFLLPPDS----GEKIALGVTVLLAFSVFMLAI    296
XP_009043980.1      ------TLYYLFNIIFPCLWLTILSLLGFWLPPDS----GEKITLGITVLLAFSVFMLLI    257
VDM43573.1          ------TLYYLYNVVFPCMMMSVLTLLVFLLPPDS----GEKIALGITVLLAFSVFVLAI    375
XP_003140283.1      ------TLYYLYNVIFPCLMMSVLTLLVFILPPDS----NEKITLGITVLLAFSVSVLAI    266
VDN82010.1          ------TLYYLYNVVFPCLMMSVLTLLVFVLPPDS----NEKIALGITVLLAFSVSVLAI    292
VDO31501.1          VHKKHMTLYYLYNVVFPCLMMSVLTLLVFVLPPDS----NEKIALGITVLLAFSVSVLAI    304
VDP19591.1          ------ALYYVFNLIVPCLLISGMALMVFMLPPDA----GEKISLGVTILLSLTMFLQLV    288
EDO32053.1          ------AMFYVFNMVLPTGVIALLSLFSFYLPPNS----GERVSFVITVLLAMSVYLIMV    260
TRY67230.1          ------TMYYFSTLILPCVLIASMSIFGFYLPPES----GEKITLQITVLMALTFYMNMV    271
KAF7391312.1        ------TLYYFFNLIVPCVLISSMALLGFTLPPDS----GEKLTLGVTILLSLTVFLNLV    285
GFG30449.1          ------TLYYFFNLIVPCVLIASMAVLGFTLPPDS----GEKLSLGVTILLSLTVFLNMV    295
KQS30083.1          ------TLYYFFNLIVPCVLIASMALLGFTLPPDS----GEKLSLGVTILLSLTVFLNMV    317
EDW57583.2          ------TLYYFFNLIIPCVLIASMALLGFTLPPDS----GEKLSLGVTILLSLTVFLNMV    453
KNC22799.1          ------TLYYFFNLIIPCVLIASMALLGFTLPPDS----GEKLSLGVTILLSLTVFLNMV    263
RZF44856.1          ------TLYYFFNLIVPCVLIASMAVLGFTLPPDS----GEKLSLGVTILLSLTVFLNMV    283
ENN76856.1          ------TLYYFFNLIVPCVLIASMAVLGFTLPPDS----GEKLSLGVTILLSLTVFLNMV    289
KAF5300392.1        ------TLYYFFNLIVPCVLIASMAVLGFTLPPDS----GEKLSLGVTILLSLTVFLNMV    293
CAB3239999.1        ------TLYYFFNLIVPCVLIASMALLGFTLPPDS----GEKLSLGVTILLSLTVFLNMV    280
PCG77624.1          ------TLYYFFNLIVPCVLIASMALLGFTLPPDS----GEKLSLGVTILLSLTVFLNMV    223
PZC79131.1          ------TLYYFFNLIVPCVLIASMALLGFTLPPDS----GEKLSLGVTILLSLTVFLNMV    290
KAF4083067.1        ------PILYVVNLLIPSSFLMIIDILSFYLPPHS----VDRASFKMTLILGYTVFLLIM    265
XP_009924659.1      ------PTLYILNLILPTCALYLLDMAVLFGPSSL----EEKISFQIAIILGSSMLAVIL    288
XP_010123544.1      ------PTLYVLNLILPTCALYLLDMAVLFGPISL----EEKINFQIAIILGSSMLAVIL    287
CBN81618.1          ------SILYIINFLLPVFFFLCLDIASFLISDIG----GEKLSFKVTVLLAVTVMQLIL    283
KAF3704230.1        ------SVLYIVNFLLPVLFFLCLDLASFLISDHG----GEKLSFKVTVLLAVTVLQLIL    279
RUS86578.1          ------SGFFVLNIVMPVVLLSFLNIVVFLIPVDS----GEKISYGITVLLALTVFMSIV    301
XP_034309618.1      ------PLFLTLSLVFPIIALSIMNAFCFLLPIES----GEKMGLSVALFLTFAVFGSIL    286
PIK58946.1          ------PENYILTLMLPSTLLCIMAFATFIAPPDS----GERISLGVSMVLGLTVFQLLV    314
VUZ42516.1          ------AEYYAYLLVLPCILLAVLNLVVFWLPPQN----PARMMLGMNIFSGFCIQLKFL    291
PAA51166.1          ------ASFYLSILVLPCILLSCLTWVIFWLPPES----PAKMQLGMNIFVAFFILMLLL    331
TGZ55997.1          ------PSFYVFMLVIPCVLLSSLTLVVFWLPPES----PAKMMLGMNIFVAFFVLLLLL    326
VDP48851.1          ------PSFYVFMLVIPCVLLSSLTLVVFWLPPES----PAKMMLGMNIFVAFFVLLLLL    282
PVD38331.1          ------VAFYTFILVMPCALLSLLTLVIFWVPPES----PAKLILGMNIFLAFFVLLLLL    277
VDK73355.1          ------PLFYTVNLVFPCVGISFLTVLVFYLPSHS----GEKVTLCISILVTLTVFYLLL    269
VDD84963.1          ------PLFYTVNLVFPCVGISFLTIVVFYLPSDS----GEKVSLCISILVALTVYYLLL    308
```

```
VDK42196.1          ------PLFYTVNLVFPCVGISFLTILVFYLPSDS----GEKITLCISILVALTVFFLLL     235
KFD58288.1          ------TLFYSVNLIIPCIGISFLTILVFYLPSDS----GEKVALSVSILVCLTMFFNLL     313
OUC49089.1          ------TLFYSVNLIIPCIGISFLTILVFYLPSDS----GEKVSLSVSILICLTMFFNLL     310
EYC26492.1          ------TLFYTVNLIFPSVGISFLTALVFYLPSDG----GEKISLCISILISLTVFFLLL     336
VDK27218.1          ------TLFYIVYLIFPIVSINFLTVLVFYLPSDG----GEKISLCLNILISLTIFFLLL     305
VDM17286.1          ------PLFYITNIIIPCVLISILGVCVFCLPADA----GEKITLSISILVTLVVYMILV     213
VDL91846.1          ------TIFYTFNLMMPCVAISALMLTFYLPPES----REKISLSINIFISLTLFFLLI     283
KAE9548540.1        ------TLFFTCNLIIPCFLISFLTTFVFYLS-------DHKITFSISILVTLTVFFLVL     301
PDM74087.1          ------TLFYTVVLIIPTVLMAFLSMAVFFLPTDS----GEKITLTISVLLSIVVFLLLV     274
XP_024504322.1      ------TLFYTVVLIIPTVVMAFISMSVFFLPTES----TEKISLTTSVLLSIVVFLLLV     277
PIO52336.1          ------TLFYTVVLIIPTVLMAFLSMAVFFLPTDS----GEKMTLTISVLLSIVVFLLLV     273
TKR73865.1          ------TLFYTVVLIIPTVLMAFLSMAVFFLPTDS----GEKMTLTISVLLSIVVFLLLV     278
RLU23395.1          ------TLFYTVNLILPTVLISFLCVLVFYLPAEA----GEKVTLGISILLSLVVFLLLV     287
XP_002427906.1      ------TLFYTVNLILPTVLISFLCVLVFYLPAEA----GEKVTLGISILLSLVVFLLLV     288
TMW47392.1          ------TLFYTVNLILPTVLISFLCVLVFYLPAEA----GEKVTLGISILLSLVVFLLLV     288
VDD83956.1          ------PLFFGVNLICPCISISILTILVFYLPADS----REKISLSISTLIALTVFFLLV     300
VEL33078.1          ------TLFFTVNLMMPCVTISFLTVLVFYLPSES----GEKISLSISILVSLTLFFLLL     230
XP_009019088.1      ------FLFYTVNLILPLMSHAFITVLVFYIPAAS----TEKMSLSINILLSLTVFFLML     292
KAE9536378.1        ------TLFYTVNLIVPCVGISYLSVLVFYLPADS----KEKISLCITILLSQTMFFLLI     329
KDR23473.1          ------TLFYTVNLIVPCVGISYLSVLVFYLPADS----GEKIALCISILLSQTMFFLLI     278
KAF7266914.1        ------TLFYTVNLIIPCVGITFLTVLVFYLPSDS----GEKVTLCVSILLSLTVFFLLL     311
KAF4519445.1        ------TLFYTVNLIIPCVGITFLTVLVFYLPSDS----GEKVTLCISILLSLTVFFLLL     308
OXU25983.1          ------TLFYTVNLIIPCVGITFLTVLVFYLPSDS----GEKVSLCSSILLSLTVFFLLL     296
TGZ32403.1          ------TLFYTVNLIIPCVGITFLTVLVFYLPSDS----GEKVSLCSSILLSLTVFFLLL     306
ELT94491.1          ------TLFYTVNLILPCVAICSVTLLVFYIPASS----GEKITMGITILNSLNIFLLLV     296
VVC39575.1          ------TLFYTVNLIIPCMGISFLTILVFYLPSDS----NEKVSLSISILLSLTVFFLLL     387
RVE49089.1          ------TLFYTVNLIIPCMGISFLTVLVFYLPSDS----GEKVSLSISILLSLTVFFLLL     291
KAB0800277.1        ------TLFYTVNLIIPCVGISFLSVLVFYLPSDS----GEKISLSISILLSLTVFFLLL     288
TDG52197.1          ------TLFYTVNLIIPCVGISFLSVLVFYLPSDS----GEKISLCISILLSLTVFFLLL     274
TMW48669.1          ------TLFYTVNLIIPCVGISFLSVLVFYLPSDS----GEKISLCISILLSLTVFFLLL     289
XP_025896085.1      ------PLFYTVSLLLPSIFLMLTDLVGFYLPPNS----GERVSFKITLLLGYSVFLIIV     293
KAF2977017.1        ------PLFYTVSLLLPSIFLMLMDIVGFYLPPHS----GERVSFKITLLLGYSVFLIIV     97
XP_009321837.1      ------PLFYTVSLLLPSIFLMVMDIVGFYLPPNS----GERVSFKITLLLGYSVFLIIV     272
XP_009979985.1      ------PLFYTVSLLLPSIFLMVMDIVGFYLPPNS----GERVSFKITLLLGYSVXXXXA     245
XP_028942374.1      ------PXXXXXXXLLPSIFLMVMDIVGFYLPPHS----GERVSFKITLLLGYSVFLIIV     269
PKK19633.1          ------PLFYTVSLLLPSIFLMVMDIVGFYLPPNS----GERVSFKITLLLGYSVFLIIV     335
XP_009894240.1      ------PLFYTVSLLLPSIFLMLMDIVGFYLPPNS----GERVSFKITLLLGYSVFLIIV     388
XP_032820219.1      ------PLFYVVNLIMPSAFLMVMDIAGFYLPPDS----GERVSFKITLLLGYSVFLIVV     321
XP_029429480.1      ------PLFYVVNLLLPSMFLLLMDVIGFYLPPDC----GERISFKITLLLGYSVFLIIV     324
XP_030077572.1      ------PLFYVVNLLLPSMFLMMMDIFGFYLPPNC----GERVSFKITLLLGYSVFLIIV     271
XP_033774596.1      ------PLFYVVNLLLPSMFLMMMDIVGFYLPPNC----GERVSFKITLLLGYSVFLIIV     302
ETE72600.1          ------PLFYAVSLLLPSIFLMVMDIVGFYLPPDS------------------------     303
XP_018082638.1      ------PLFYAVNLLLPSMFLMVMDIIGFYLPPDS----GERVSFKITLLLGYSVFLIIV     504
XP_032905410.1      ------PLFYAVNLLLPSILLMLMDIIGFNLPPDC----AERVSFKITLLLGYSVFLIIV     291
XP_020773490.1      ------PLFYTVNLLLPSMFLMIMDVVGFYLPPDS----GERVSFKITLLLGYSVFLIIV     297
XP_033833634.1      ------PLFYTVNLLLPSMFLMIMDVVGFYLPPDS----GERVSFKITLLLGYSVFLIIV     323
XP_030215795.1      ------PLFYTVNLLLPSIFLMVMDIVGFYLPPDS----GERVSFKVTLLLGYSVFLIIV     296
XP_030602980.1      ------PLFYTVNLLLPSIFLMVMDIVGFYLPPDS----GERVSFKITLLLGYSVFLIIV     302
XP_004573543.1      ------PLFYTVNLLLPSIFLMVMDIVGFYLPPDS----GERVSFKITLLLGYSVFLIIV     296
XP_005916159.1      ------PLFYTVNLLLPSIFLMVMDIVGFYLPPDS----GERVSFKITLLLGYSVFLIIV     296
XP_013132089.1      ------PLFYTVNLLLPSIFLMVMDIVGFYLPPDS----GERVSFKITLLLGYSVFLIIV     296
XP_031603488.1      ------PLFYTVNLLLPSIFLMVMDIVGFYLPPDS----GERVSFKITLLLGYSVFLIIV     302
XP_005725536.1      ------PLFYTVNLLLPSIFLMVMDIVGFYLPPDS----GERVSFKITLLLGYSVFLIIV     302
XP_026038075.1      ------PLFYTVNLLLPSIFLMVMDIVGFYLPPDS----GERVSFKITLLLGYSVFLIIV     302
CAF96649.1          ------PLFYTVNLLLPSIFLMVMDIVGFYLPPDS----GERVSFKITLLLGYSVFLIIV     287
XP_023818570.1      ------PLFYTVNLLLPSIFLMVMDVVGFYLPPDS----GERVSFKITLLLGYSVFLIIV     292
RVE64289.1          ------PLFYTVNLLLPSIFLMVMDVVGFYLPPDS----GERVSFKITLLLGYSVFLIIV     327
XP_024121971.1      ------PLFYTVNLLLPSIFLMVMDVVGFYLPPDS----GERVSFKITLLLGYSVFLIIV     298
XP_015817559.1      ------PLFYTVNLLLPSVFLMVMDIVGFYLPPDS----GERVSFKITLLLGYSVFLIIV     282
XP_015225670.1      ------PLFYTVNLLLPSIFLMVMDIVGFYLPPDS----GERVSFKITLLLGYSVFLIIV     281
XP_012722063.1      ------PLFYTVNLLLPSIFLMVMDIVGFYLPPDS----GERVSFKITLLLGYSVFLIIV     296
XP_032431307.1      ------PLFYTVNLLLPSVFLMVMDIVGFFLPPDS----GERVSFKITLLLGYSVFLIIV     296
XP_014328329.1      ------PLFYTVNLLLPSVFLMVMDIVGFFLPPDS----GERVSFKITLLLGYSVFLIIV     296
XP_027886578.1      ------PLFYTVNLLLPSVFLMVMDIVGFFLPPDS----GERVSFKITLLLGYSVFLIIV     296
XP_008426791.1      ------PLFYTVNLLLPSIFLMVMDIVGFFLPPDS----GERVSFKITLLLGYSVFLIIV     296
XP_014838686.1      ------PLFYTVNLLLPSVFLMVMDIVGFFLPPDS----GERVSFKITLLLGYSVFLIIV     296
XP_014885967.1      ------PLFYTVNLLLPSVFLMVMDIVGFFLPPDS----GERVSFKITLLLGYSVFLIIV     296
XP_033954312.1      ------PLFYTVNLLLPSMFLMVMDIVGFYLPPDS----GERVSFKITLLLGYSVFLIIV     296
XP_034089244.1      ------PLFYTVNLLLPSMFLMVMDIVGFYLPPDS----GERVSFKITLLLGYSVFLIIV     296
XP_010780064.1      ------PLFYTVNLLLPSMFLMVMDIVGFYLPPDS----GERVSFKITLLLGYSVFLIIV     312
XP_033970261.1      ------PLFYTVNLLLPSMFLMVMDIVGFYLPPDS----GERVSFKITLLLGYSVFLIIV     312
XP_029375575.1      ------PLFYTVNLLLPSIFLMVMDIVGFYLPPDS----GERVSFKITLLLGYSVFLIIV     333
KAF0023022.1        ------PLFYTVNLLLPSMFLMVMDVVGFYLPPDS----GERVSFKITLLLGYSVFLIIV     329
XP_019952614.1      ------PLFYTVNLLLPSIFLMVMDIVGFYLPPDS----GERVSFKITLLLGYSVFLIIV     285
XP_034463117.1      ------PLFYTVNLLLPSIFLMVMDIVGFYLPPDS----GERVSFKITLLLGYSVFLIIV     296
XP_029924656.1      ------PLFYTVNLLLPSIFLMVMDIVGFYLPPDS----GERVSFKVTLLLGYSVFLIIV     297
XP_028323228.1      ------PLFYTVNLLLPSIFLMVIDIVGFYLPPDS----GERVSFKITLLLGYSVFLIIV     296
```

```
TNM84480.1          ------PLFYTVNLLLPSIFLMVMDIVGFYLPPDS----GERVSFKITLLLGYSVFLIIV     296
XP_029703788.1      ------PLFYTVNLLLPSIFLMVMDIVGFYLPPDS----GERVSFKITLLLGYSVFLIIV     296
XP_030010368.1      ------PLFYTVNLLLPSIFLMVMDIVGFYLPPDS----GERVSFKITLLLGYSVFLIIV     299
XP_019725056.1      ------PLFYTVNLLLPSIFLMVMDIVGFYLPPDS----GERVSFKITLLLGYSVFLIIV     297
XP_034034934.1      ------PLFYTVNLLLPSIFLMVMDVVGFYLPPDS----GERVSFKITLLLGYSVFLIIV     285
XP_029956880.1      ------PLFYTVNLLLPSVFLMVMDIVGFYLPPDS----GERVSFKITLLLGYSVFLIIV     296
XP_008331307.3      ------PLFYTVNLLLPSIFLMVMDVVGFYLPPDS----GERVSFKITLLLGYSVFLIIV     329
XP_031724845.1      ------PLFYTVNLLLPSIFLMVMDIVGFYLPPDS----GERVSFKITLLLGYSVFLIIV     297
XP_013859395.1      ------PLFYTVNLLLPSIFLMVMDVVGFYLPPDS----GERVSFKITLLLGYSVFLIIV     296
XP_017275335.1      ------PLFYTVNLLLPSIFLMVMDVVGFYLPPDS----GERVSFKITLLLGYSVFLIIV     296
XP_020496197.1      ------PLFYTVNLLLPSIFLMVMDIVGFYLPPDS----GERVSFKITLLLGYSVFLIIV     296
XP_029030400.1      ------PLFYTVNLLLPSIFLMVMDIVGFYLPPDS----GERVSFKITLLLGYSVFLIIV     296
XP_022060538.1      ------PLFYTVNLLLPSIFLMVMDVVGFYLPPDS----GERVSFKITLLLGYSVFLIIV     297
XP_023150584.1      ------PLFYTVNLLLPSIFLMVMDVMGFYLPPDS----GERVSFKITLLLGYSVFLIIV     297
XP_029303892.1      ------PLFYTVNLLLPSIFLMVMDIVGFYLPPDS----GERVSFKITLLLGYSVFLIIV     320
XP_020454172.1      ------PLFYTVNLLMPSIFLMVMDIVGFYLPPDS----GERVSFKITLLLGYSVFLIIV     295
TKS83244.1          ------PLFYTVNLLLPSIFLMVMDVVGFYLPPDS----GERVSFKITLLLGYSVFLIIV     283
XP_022616583.1      ------PLFYTVNLLLPSIFLMVMDIVGFYLPPDS----GERVSFKITLLLGYSVFLIIV     329
XP_030293172.1      ------PLFYTVNLLLPSIFLMVMDIVGFYLPPDS----GERVSFKITLLLGYSVFLIIV     296
XP_027129539.1      ------PLFYTVNLLLPSIFLMVMDVVGFYLPPDS----GERVSFKITLLLGYSVFLIIV     296
XP_034539538.1      ------PLFYTVNLLLPSIFLMVMDIVGFYLPPDS----GERVSFKITLLLGYSVFLIIV     296
XP_008277330.1      ------PLFYTVNLLLPSIFLMVMDIVGFYLPPDS----GERVSFKITLLLGYSVFLIIV     296
XP_028276940.1      ------PLFYTVNLLLPSIFLMVMDIVGFYLPPDS----GERVSFKITLLLGYSVFLIIV     296
XP_023263433.1      ------PLFYTVNLLLPSIFLMVMDIVGFYLPPDS----GERVSFKITLLLGYSVFLIIV     296
XP_034406336.1      ------PLFYTVNLLLPSIFLMVMDIVGFYLPPDS----GERVSFKITLLLGYSVFLIIV     296
XP_026184460.1      ------PLFYTVNLLLPSIFLMVMDIVGFYLPPDS----GERVSFKITLLLGYSVFLIIV     296
KAF1378228.1        ------PLFYTVNLLLPSIFLMVMDIVGFYLPPDS----GERVSFKITLLLGYSVFLIIV     281
XP_028450365.1      ------PLFYTVNLLLPSIFLMVMDIVGFYLPPDS----GERVSFKITLLLGYSVFLIIV     296
XP_031163851.1      ------PLFYTVNLLLPSIFLMVMDIVGFYLPPDS----GERVSFKITLLLGYSVFLIIV     296
XP_032389084.1      ------PLFYTVNLLLPSIFLMVMDIVGFYLPPDS----GERVSFKITLLLGYSVFLIIV     296
XP_033494682.1      ------PLFYTVNLLLPSIFLMVMDIVGFYLPPDS----GERVSFKITLLLGYSVFLIIV     296
XP_018544782.1      ------PLFYTVNLLLPSIFLMVMDIVGFYLPPDS----GERVSFKITLLLGYSVFLIIV     296
XP_026228189.1      ------PLFYTVNLLLPSIFLMVMDIVGFYLPPDS----GERVSFKITLLLGYSVFLIIV     296
XP_028976606.1      ------PLFYTVNLLLPSIFLMVMDLVGFYLPPDS----GERVSFKITLLLGYSVFLIIV     296
CAB1352378.1        ------PLFYTVNLLLPSIFLMVMDLVGFYLPPDS-------------------------     275
XP_023866849.1      ------PLFYTVNLLLPSIFLMVMDLVGFYLPPDS----GERVSFKITLLLGYSVFLIIV     296
XP_013992832.1      ------PLFYTVNLLLPSIFLMVMDLVGFYLPPDS----GERVSFKITLLLGYSVFLIIV     296
XP_029546688.1      ------PLFYTVNLLLPSIFLMVMDLVGFYLPPDS----GERVSFKITLLLGYSVFLIIV     296
XP_021481546.1      ------PLFYTVNLLLPSIFLMVMDLVGFYLPPDS----GERVSFKITLLLGYSVFLIIV     296
XP_020339889.1      ------PLFYTVNLLLPSIFLMVMDLVGFYLPPDS----GERVSFKITLLLGYSVFLIIV     296
XP_029481972.1      ------PLFYTVNLLLPSIFLMVMDLVGFYLPPDS----GERVSFKITLLLGYSVFLIIV     296
KPP68743.1          ------PLFYTVNLLLPSIFLMLMDIVGFYLPPDS----GERVSFKITLLLGYSVFLIIV     273
XP_023669383.1      ------PLFYTVNLLLPSIFLMLMDIVGFYLPPDS----GERVSFKITLLLGYSVFLIIV     295
KAA0720096.1        ------PLFYTVNLLLPSIFLMVMDIVGFYLPPDS----GERVSFKITLLLGYSVFLIIV     201
XP_009293684.1      ------PLFYTVNLLLPSMFLMVMDIVGFYLPPDS----GERVSFKITLLLGYSVFLIIV     295
XP_026090784.1      ------PLFYTVNLLLPSIFLMVMDIVGFYLPPDS----GERVSFKITLLLGYSVFLIIV     295
XP_018918715.1      ------PLFYTVNLLLPSVFLMVMDIVGFYLPPDS----GERVSFKITLLLGYSVFLIIV     295
KAF4098549.1        ------PLFYTVNLLLPSVFLMVMDIVGFYLPPDS----GERVSFKITLLLGYSVFLIIV     295
XP_016084173.1      ------PLFYTVNLLLPSVFLMVMDIVGFYLPPDS----GERVSFKITLLLGYSVFLIIV     295
XP_016332759.1      ------PLFYTVNLLLPSVFLMVMDIVGFYLPPDS----GERVSFKITLLLGYSVFLIIV     295
XP_016398039.1      ------PLFYTVNLLLPSVFLMVMDIVGFYLPPDS----GERVSFKITLLLGYSVFLIIV     295
XP_026989970.1      ------PLFYTVNLLLPSIFLMVMDIVGFYLPPDS----GERVSFKITLLLGYSVFLIIV     295
TSQ12698.1          ------PLFYTVNLLLPSIFLMVMDIVGFYLPPDS----GERVSFKITLLLGYSVFLIIV     295
XP_017347546.1      ------PLFYTVNLLLPSIFLMVMDIVGFYLPPDS----GERVSFKITLLLGYSVFLIIV     290
XP_026794616.2      ------PLFYTVNLLLPSIFLMVMDIVGFYLPPDS----GERVSFKITLLLGYSVFLIIV     295
XP_026861889.1      ------PLFYTVNLLLPSIFLMVMDIVGFYLPPDS----GERVSFKITLLLGYSVFLIIV     295
XP_017575347.1      ------PLFYTVNLLLPSIFLMVMDIVGFYLPPDS----GERVSFKITLLLGYSVFLIIV     295
XP_022531596.1      ------PLFYTVNLLLPSIFLMVMDIVGFYLPPDS----GERVSFKITLLLGYSVFLIIV     295
XP_012691019.2      ------PLFYTVNLLLPSIFLMVMDVVGFYLPPDS----GERVSFKITLLLGYSVFLIIV     278
XP_028839723.1      ------PLFYTVNLLLPSIFLMVMDIVGFYLPPDS----GERVSFKITLLLGYSVFLIIV     295
XP_030638860.1      ------PLFYTVNLLLPSIFLMVMDIVGFYLPPDS----GERVSFKITLLLGYSVFLIIV     277
XP_007882964.1      ------PLFYAVNLLLPSIFLMVMDIIGFYLPPDS----GERVSFKITLLLGYSVFLIIV     273
GCB70244.1          ------PLFYAVSLLLPSIFLMVMDIIGFYLPPDS----GERVSFKITLLLGYSVFLIIV     274
GCC26242.1          ------PLFYAVSLLLPSIFLMVMDIIGFYLPPDS----GERVSFKITLLLGYSVFLIIV     295
XP_020387034.1      ------PLFYAVSLLLPSIFLMVMDIIGFYLPPDS----GERVSFKITLLLGYSVFLIIV     286
XP_028931940.1      ------PLFYTVSLLLPSIFLMIMDIVGFYLPPDS----GERVSFKITLLLGYSVFLIIV     301
XP_031762490.1      ------PLFYAVNLLLPSMFLMVMDIIGFYLPPDS----GERVSFKITLLLGYSVFLIIV     289
PIO32240.1          ------PLFYAVNLLLPSMFLMVMDIIGFYLPPDS----GERVSFKITLLLGYSVFLIIV     87
XP_018425466.1      ------PLFYAVNLLLPSMFLMVMDIIGFYLPPDS----GERVSFKITLLLGYSVFLIIV     290
XP_006642351.1      ------PLFYTVSLLLPSVFLMLMDIVGFYLPPDS----GERVSFKITLLLGYSVFLIIV     312
XP_028665372.1      ------PLFYAVSLLLPSVFLMVMDIVGFYLPPDS----GERVSFKITLLLGYSVFLIIV     295
GCF49814.1          ------PLFYAVSLLLPSIFLMVMDIVGFYLPPDS----GERVSFKITLLLGYSVFLIIV     276
XP_015277816.1      ------PLFYAVSLLLPSIFLMVMDIVGFYLPPDS----GERVSFKITLLLGYSVFLIIV     299
XP_005987340.2      ------PLFYTVSLLLPSIFLMVMDIVGFYLPPDS----GERVSFKITLLLGYSVFLIIV     279
XP_019339534.1      ------PLFYAVSLLLPSIFLMVMDIVGFYLPPDC----GERVSFKITLLLGYSVFLIIV     296
XP_025063414.1      ------PLFYAVSLLLPSIFLMVMDIVGFYLPPDC----GERVSFKITLLLGYSVFLIIV     296
XP_019367488.1      ------PLFYAVSLLLPSIFLMVMDIVGFYLPPDC----GERVSFKITLLLGYSVFLIIV     296
```

XP_019412078.1	------PLFYAVSLLLPSIFLMVMDIVGFYLPPDC----GERVSFKITLLLGYSVFLIIV	296
XP_014434314.1	------PLFYAVNLLLPSIFLMVMDIVGFYLPPDS----GERVSFKITLLLGYSVFLIIV	296
XP_007060556.1	------PLFYAVNLLLPSIFLMVMDIVGFYLPPDS----GERVSFKITLLLGYSVFLIIV	290
XP_024072175.1	------PLFYAVSLLLPSIFLMVMDIVGFYLPPDS----GERVSFKITLLLGYSVFLIIV	275
XP_008170567.1	------PLFYAVSLLLPSIFLMVMDIVGFYLPPDS----GERVSFKITLLLGYSVFLIIV	296
XP_034610543.1	------PLFYAVSLLLPSIFLMVMDIVGFYLPPDS----GERVSFKITLLLGYSVFLIIV	296
XP_030394127.1	------PLFYAVSLLLPSVFLMVMDIVGFYLPPDS----GERVSFKITLLLGYSVFLIIV	296
XP_032651960.1	------PLFYAVSLLLPSIFLMVMDIVGFYLPPDS----GERVSFKITLLLGYSVFLIIV	296
XP_025020083.1	------PLFYAVSLLLPSIFLMVMDIVGFYLPPDS----GERVSFKITLLLGYSVFLIIV	296
XP_026536833.1	------PLFYAVSLLLPSIFLMVMDIVGFYLPPDS----GERVSFKITLLLGYSVFLIIV	296
XP_026564787.1	------PLFYAVSLLLPSIFLMVMDIVGFYLPPDS----GERVSFKITLLLGYSVFLIIV	296
XP_032084675.1	------PLFYAVSLLLPSIFLMVMDIVGFYLPPDS----GERVSFKITLLLGYSVFLIIV	281
XP_029139341.1	------PLFYAVSLLLPSIFLMVMDIVGFYLPPDS----GERVSFKITLLLGYSVFLIIV	296
XP_034281006.1	------PLFYAVSLLLPSIFLMVMDIVGFYLPPDS----GERVSFKITLLLGYSVFLIIV	296
XP_020649062.1	------PLFYAVSLLLPSIFLMVMDIVGFYLPPDS----GERVSFKITLLLGYSVFLIIV	296
XP_008117087.1	------PLFYAVSLLMPSIFLMVMDIVGFYLPPDS----GERVSFKITLLLGYSVFLIIV	296
XP_028564324.1	------PLFYAVSLLLPSIFLMVMDIVGFYLPPDS----GERVSFKITLLLGYSVFLIIV	302
XP_033028155.1	------PLFYAVSLLLPSIFLMVMDIVGFYLPPDS----GERVSFKITLLLGYSVFLIIV	301
XP_025963249.1	------PLFYAVSLLLPSIFLMVMDIVGFYLPPNS----GERVSFKITLLLGYSVFLIIV	283
XP_009668348.1	------PLFYAVSLLLPSSFLMVMDIVGFYLPPNS----GERVSFKITLLLGYSVFLIIV	295
XP_013798935.1	------PLFYAVSLLLPSIFLMVMDIVGFYLPPNS----GERVSFKITLLLGYSVFLIIV	279
XP_025913685.1	------PLFYAVSLLLPSIFLMVMDIVGFYLPPNS----GERVSFKITLLLGYSVFLIIV	304
XP_013042552.1	------PLFYTVSLLLPSIFLMVMDIVGFYLPPNS----GERVSFKITLLLGYSVFLIIV	302
XP_005030458.2	------PLFYTVSLLLPSIFLMVMDIVGFYLPPNS----GERVSFKITLLLGYSVFLIIV	295
XP_032057953.1	------PLFYTVSLLLPSIFLMVMDIVGFYLPPNS----GERVSFKITLLLGYSVFLIIV	278
XP_021232050.1	------PLFYTINLLLPSIFLMVMDIVGFYLPPNS----GERVSFKITLLLGYSVFLIIV	297
OXB62403.1	------PLFYAINLLLPSIFLMVMDIVGFYLPPNS----GERVSFKITLLLGYSVFLIIV	296
OXB81319.1	------PLFYAINLLLPSIFLMVMDIVGFYLPPNS----GERVSFKITLLLGYSVFLIIV	278
XP_010722007.1	------PLFYTINLLLPSIFLMVMDIVGFYLPPNS----GERVSFKITLLLGYSVFLIIV	297
XP_015739349.1	------PLFYTINLLLPSIFLMVMDIVGFYLPPNS----GERVSFKITLLLGYSVFLIIV	297
XP_031455498.1	------PLFYTINLLLPSIFLMVMDIVGFYLPPNS----GERVSFKITLLLGYSVFLIIV	298
POI27435.1	------PLFYTINLLLPSIFLMVMDIVGFYLPPNS----GERVSFKITLLLGYSVFLIIV	273
XP_004948120.1	------PLFYTINLLLPSIFLMVMDIVGFYLPPNS----GERVSFKITLLLGYSVFLIIV	298
XP_032851190.1	------PLFYTVNLLLPSIFLMVMDIVGFYLPPNS----GERVSFKITLLLGYSVFLIIV	293
XP_010007255.1	------PLFYTVSLLLPSIFLMVMDIVGFYLPPNS----GERVSFKITLLLGYSVFLIIV	295
XP_030320702.1	------PLFYTVSLLLPSIFLMVMDIVGFYLPPNS----GERVSFKITLLLGYSVFLIIV	266
XP_010191940.1	------PLFYTVSLLLPSIFLMVMDIVGFYLPPNS----GERVSFKITLLLGYSVFLIIV	293
XP_027737112.1	------PLFYTVSLLLPSIFLMVMDIVGFYLPPNS----GERVSFKITLLLGYSVFLIIV	291
XP_027555032.1	------PLFYTVSLLLPSIFLMVMDIVGFYLPPNS----GERVSFKITLLLGYSVFLIIV	296
XP_032565370.1	------PLFYTVSLLLPSIFLMVMDIVGFYLPPNS----GERVSFKITLLLGYSVFLIIV	342
XP_027511217.1	------PLFYTVSLLLPSIFLMVMDIVGFYLPPNS----GERVSFKITLLLGYSVFLIIV	441
XP_027593499.1	------PLFYTVSLLLPSIFLMVMDIVGFYLPPNS----GERVSFKITLLLGYSVFLIIV	392
XP_017664924.1	------PLFYTVSLLLPSIFLMVMDIVGFYLPPNS----GERVSFKITLLLGYSVFLIIV	293
XP_029817938.1	------PLFYTVSLLLPSIFLMVMDIVGFYLPPNS----GERVSFKITLLLGYSVFLIIV	275
XP_005058641.1	------PLFYTVSLLLPSIFLMVMDIVGFYLPPHS----GERVSFKITLLLGYSVFLIIV	326
XP_021385804.1	------PLFYTVSLLLPSIFLMLMDIVGFYLPPHS----GERVSFKITLLLGYSVFLIIV	295
XP_030146687.2	------PLFYTVSLLLPSIFLMLMDIVGFYLPPHS----GERVSFKITLLLGYSVFLIIV	340
KAF4796420.1	------PLFYTVSLLLPSIFLMVMDIVGFYLPPHS----GERVSFKITLLLGYSVFLIIV	295
XP_032937581.1	------PLFYTVSLLLPSIFLMVMDIVGFYLPPHS----GERVSFKITLLLGYSVFLIIV	342
XP_031989659.1	------PLFYTVSLLLPSIFLMVMDIVGFYLPPHS----GERVSFKITLLLGYSVFLIIV	296
XP_010402086.1	------PLFYTVSLLLPSIFLMVMDIVGFYLPPHS----GERVSFKITLLLGYSVFLIIV	295
XP_017594069.1	------PLFYTVSLLLPSIFLMVMDIVGFYLPPHS----GERVSFKITLLLGYSVFLIIV	266
XP_014115268.1	------PLFYTVSLLLPSIFLMVMDIVGFYLPPHS----GERVSFKITLLLGYSVFLIIV	320
XP_023797108.1	------PLFYTVSLLLPSIFLMVMDIVGFYLPPHS----GERVSFKITLLLGYSVFLIIV	266
XP_033375614.1	------PLFYTVSLLLPSIFLMVMDIVGFYLPPHS----GERVSFKITLLLGYSVFLIIV	266
XP_014740121.1	------PLFYTVSLLLPSIFLMVMDIVGFYLPPHS----GERVSFKITLLLGYSVFLIIV	440
RLV83430.1	------PLFYTVSLLLPSIFLMLMDIVGFYLPPHS----GERVSFKITLLLGYSVFLIIV	286
XP_009096098.2	------PLFYTVSLLLPSIFLMVMDIVGFYLPPHS----GERVSFKITLLLGYSVFLIIV	401
TRZ15870.1	------PLFYTVSLLLPSIFLMVMDIVGFYLPPHS----GERVSFKITLLLGYSVFLIIV	296
RMB91935.1	------PLFYTVSLLLPSIFLMVMDIVGFYLPPHS----GERVSFKITLLLGYSVFLIIV	266
XP_030820843.1	------PLFYTVSLLLPSIFLMVMDIVGFYLPPHS----GERVSFKITLLLGYSVFLIIV	342
XP_014165179.1	------PLFYTVSLLLPSIFLMVMDIVGFYLPPHS----GERVSFKITLLLGYSVFLIIV	344
XP_026653582.1	------PLFYTVSLLLPSIFLMVMDIVGFYLPPHS----GERVSFKITLLLGYSVFLIIV	226
PKU35975.1	------PLFYTVSLLLPSIFLMLMDIVGFYLPPNS----GERVSFKITLLLGYSVFLIIV	298
XP_014805072.1	------PLFYTVSLLLPSIFLMVMDIVGFYLPPNS----GERVSFKITLLLGYSVFLIIV	292
XP_009818330.1	------PLFYTVSLLLPSIFLMVMDIVGFYLPPNS----GERVSFKITLLLGYSVFLIIV	63
OPJ68307.1	------PLFYTVSLLLPSIFLMVMDIVGFYLPPNS----GERVSFKITLLLGYSVFLIIV	295
XP_008936289.1	------PLFYTVSLLLPSIFLMIMDIVGFYLPPNS----GERVSFKITLLLGYSVFLIIV	296
XP_010287046.1	------PLFYTVSLLLPSIFLMVIDIVGFYLPPNS----GERVSFKITLLLGYSVFLIIV	226
XP_005240140.2	------PLFYTVSLLLPSIFLMVMDIVGFYLPPNS----GERVSFKITLLLGYSVFLIIV	355
XP_005437752.2	------PLFYTVSLLLPSIFLMVMDIVGFYLPPNS----GERVSFKITLLLGYSVFLIIV	355
KFV74811.1	------PLFYTVSLLLPSIFLMVMDIVGFYLPPNS----GERVSFKITLLLGYSVFLIIV	260
XP_010018389.1	------PLFYTVSLLLPSIFLMVMDIVGFYLPPNS----GERVSFKITLLLGYSVFLIIV	303
KQK78711.1	------PLFYTISLLLPSIFLMVMDIVGFYLPPNS----GERVSFKITLLLGYSVFLIIV	295
XP_009570162.1	------PLFYTVSLLLPSIFLMVMDIVGFYLPPNS----GERVSFKITLLLGYSVFLIIV	294
KFP11268.1	------PLFYTVSLLLPSIFLMVMDIVGFYLPPNS----GERVSFKITLLLGYSVFLIIV	258
KFQ98910.1	------PLFYTVSLLLPSIFLMVMDIVGFYLPPNS----GERVSFKITLLLGYSVFLIIV	277

XP_012985202.3	------PLFYTVSLLLPSIFLMVMDIVGFYLPPNS----GERVSFKITLLLGYSVFLIIV	295
XP_010573388.1	------PLFYTVSLLLPSIFLMVMDIVGFYLPPNS----GERVSFKITLLLGYSVFLIIV	294
XP_029879496.1	------PLFYTVSLLLPSIFLMVMDIVGFYLPPNS----GERVSFKITLLLGYSVFLIIV	266
XP_030361086.1	------PLFYAVSLLLPSIFLMVMDIVGFYLPPNS----GERVSFKITLLLGYSVFLIIV	347
KFM00668.1	------PLFYTVSLLLPSIFLMVMDIVGFYLPPNS----GERVSFKITLLLGYSVFLIIV	278
KAF1479074.1	------PLFYTVSLLLPSIFLMVMDIVGFYLPPNS----GERVSFKITLLLGYSVFLIIV	258
KAF1651161.1	------PLFYTVSLLLPSIFLMVMDIVGFYLPPNS----GERVSFKITLLLGYSVFLIIV	259
KAF1673648.1	------PLFYTVSLLLPSIFLMVMDIVGFYLPPNS----GERVSFKITLLLGYSVFLIIV	259
KAF1493319.1	------PLFYTVNLLLPSIFLMVMDIVGFYLPPNS----GERVSFKITLLLGYSVFLIIV	259
KAF1584157.1	------PLFYTVNLLLPSIFMMVMDIVGFYLPPNS----GERVSFKITLLLGYSVFLIIV	259
KAF1571723.1	------PLFYTVNLLLPSIFMMVMDIVGFYLPPNS----GERVSFKITLLLGYSVFLIIV	259
KAF1533169.1	------PLFYTVNLLLPSIFMMVMDIVGFYLPPNS----GERVSFKITLLLGYSVFLIIV	259
KAF1638955.1	------PLFYTVNLLLPSIFMMVMDIVGFYLPPNS----GERVSFKITLLLGYSVFLIIV	259
KAF1549972.1	------PLFYTVNLLLPSIFLMVMDIVGFYLPPNS----GERVSFKITLLLGYSVFLIIV	259
KAF1606914.1	------PLFYTVNLLLPSIFLMVMDIVGFYLPPNS----GERVSFKITLLLGYSVFLIIV	259
KAF1510957.1	------PLFYTVNLLLPSIFLMVMDIVGFYLPPNS----GERVSFKITLLLGYSVFLIIV	259
KAF1498899.1	------PLFYTVNLLLPSIFLMVMDIVGFYLPPNS----GERVSFKITLLLGYSVFLIIV	259
KAF1411525.1	------PLFYTVNLLLPSIFLMVMDIVGFYLPPNS----GERVSFKITLLLGYSVFLIIV	259
KAF1429205.1	------PLFYTVNLLLPSIFLMVMDIVGFYLPPNS----GERVSFKITLLLGYSVFLIIV	259
XP_005334318.1	------PLFYAVSLLLPSIFLMVMDIVGFYLPPDS----GERVSFKITLLLGYSVFLIIV	285
XP_014395552.1	------PLFYAVSLLLPSIFLMVMDIVGFYLPPES----GERVSFKITLLLGYSVFLIIV	285
KAB0404854.1	------PLFYAVSLLLPSIFLMVMDIVGFYLPPDS----GERVSFKITLLLGYSVFLIIV	281
XP_007494828.1	------PLFYTVNLLLPSIFLMVMDIVGFYLPPDS----GERVSFKITLLLGYSVFLIIV	296
XP_003764254.1	------PLFYTVSLLLPSIFLMVMDIVGFYLPPDS----GERVSFKITLLLGYSVFLIIV	296
XP_020845489.1	------PLFYTVSLLLPSIFLMVMDIVGFYLPPDS----GERVSFKITLLLGYSVFLIIV	296
XP_027703160.1	------PLFYTVSLLLPSIFLMVMDIIGFYLPPDS----GERVSFKITLLLGYSVFLIIV	296
XP_017523929.1	------PLFYAVSLLLPSIFLMVMDIVGFYLPPDS----GERVSFKITLLLGYSVFLIIV	293
XP_008688428.1	------PLFYAVSLLLPSIFLMLMDIVGFYLPPDS----GERVSFKITLLLGYSVFLIIX	248
XP_029812166.1	------PLFYTVSLILPSIFLMFMDIVGFYLPPDS----GERVSFKITLLLGYSVFLIIV	310
XP_025749781.1	------PLFYAVSLLLPSIFLMLMDIVGFYLPPDS----GERVSFKITLLLGYSVFLIIV	296
XP_004416432.1	------PLFYAVSLLLPSIFLMLMDIVGFYLPPDS----GERVSFKITLLLGYSVFLIIV	296
XP_027436262.1	------PLFYAVSLLLPSIFLMLMDIVGFYLPPDS----GERVSFKITLLLGYSVFLIIV	296
XP_032284025.1	------PLFYAVSLLLPSIFLMLMDIVGFYLPPDS----GERVSFKITLLLGYSVFLIIV	296
XP_006735421.1	------PLFYAVSLLLPSIFLMLMDIVGFYLPPDS----GERVSFKITLLLGYSVFLIIV	296
XP_021552166.1	------PLFYAVSLLLPSIFLMLMDIVGFYLPPDS----GERVSFKITLLLGYSVFLIIV	296
XP_026361066.1	------PLFYAVSLLLPSIFLMLMDIVGFYLPPDS----GERVSFKITLLLGYSVFLIIV	296
XP_034523598.1	------PLFYAVSLLLPSVFLMLMDIVGFYLPPDS----GERVSFKITLLLGYSVFLIIV	296
NP_001297113.1	------PLFYAVSLLLPSIFLMLMDIVGFYLPPDS----GERVSFKITLLLGYSVFLIIV	296
XP_032215488.1	------PLFYAVSLLLPSIFLMLMDIVGFYLPPDS----GERVSFKITLLLGYSVFLIIV	296
VCX31483.1	------PLFYAVSLLLPSIFLMLMDIVGFYLPPDS----GERVSFKITLLLGYSVFLIIV	296
XP_022369003.1	------PLFYAVSLLLPSIFLMLMDIVGFYLPPDS----GERVSFKITLLLGYSVFLIIV	296
XP_032694248.1	------PLFYAVSLLLPSIFLMLMDIVGFYLPPDS----GERVSFKITLLLGYSVFLIIV	296
NP_001041584.1	------PLFYTVSLLLPSIFLMLMDIVGFYLPPDS----GERVSFKITLLLGYSVFLIIV	296
XP_025862501.1	------PLFYTVSLLLPSIFLMLMDIVGFYLPPDS----GERVSFKITLLLGYSVFLIIV	296
KAF0873564.1	------PLFYTVSLLLPSIFLMFMDIVGFYLPPDS----GERVSFKITLLLGYSVFLIIV	438
XP_025784751.1	------PLFYTVSLLLPSIFLMFMDIVGFYLPPDS----GERVSFKITLLLGYSVFLIIV	296
XP_007075625.1	------PLFYTVSLLLPSIFLMFMDIVGFYLPPDS----GERVSFKITLLLGYSVFLIIV	432
XP_030189489.1	------PLFYTVSLLLPSIFLMFMDIVGFYLPPDS----GERVSFKITLLLGYSVFLIIV	296
XP_019324655.1	------PLFYTVSLLLPSIFLMFLDIVGFYLPPDS----GERVSFKITLLLGYSVFLIIV	397
XP_023094886.1	------PLFYTVSLLLPSIFLMFMDIVGFYLPPDS----GERVSFKITLLLGYSVFLIIV	432
XP_026892357.1	------PLFYTVSLLLPSIFLMFMDIVGFYLPPDS----GERVSFKITLLLGYSVFLIIV	296
XP_016004457.1	------PLFYVVNLLLPSIFLMVMDIVGFYLPPDS----GERVSFKITLLLGYSVFLIIV	291
XP_006912860.1	------PLFYVVSLLLPSIFLMVMDIVGFYLPPDS----GERVSFKITLLLGYSVFLIIV	291
XP_011363679.1	------PLFYVVSLLLPSIFLMVMDIVGFYLPPDS----GERVSFKITLLLGYSVFLIIV	291
XP_016076060.1	------PLFYAVSLLLPSIFLMVMDIVGFYLPPES----GERVSFKITLLLGYSVFLIIV	296
XP_008148371.1	------PLFYAVNLLLPSIFLMVMDIVGFYLPPES----GERVSFKITLLLGYSVFLIIV	296
XP_006093568.1	------PLFYAVSLLLPSIFLMVMDIVGFYLPPES----GERVSFKITLLLGYSVFLIIV	296
XP_006761601.1	------PLFYAVSLLLPSIFLMVMDIVGFYLPPES----GERVSFKITLLLGYSVFLIIV	296
XP_024426743.1	------PLFYAVSLLLPSIFLMVMDIVGFYLPPES----GERVSFKITLLLGYSVFLIIV	291
XP_019520779.1	------PLFYAVSLLLPSIFLMVMDIVGFYLPPDS----GERVSFKITLLLGYSVFLIIV	296
XP_032976539.1	------PLFYAVSLLLPSIFLMVMDIVGFYLPPDS----GERVSFKITLLLGYSVFLIIV	296
ELW64270.1	------PLFYAVSLLLPSIFLMVMDIVGFYLPPDS----GERVSFKITLLLGYSVFLIIV	300
XP_004427367.1	------PLFYAVSLLLPSIFLMVMDIVGFYLPPDS----GERVSFKITLLLGYSVFLIIV	296
XP_008529353.1	------PLFYAVSLLLPSIFLMVMDIVGFYLPPDS----GERVSFKITLLLGYSVFLIIV	296
NP_001288165.1	------PLFYAVSLLLPSIFLMVMDIVGFYLPPDS----GERVSFKITLLLGYSVFLIIV	296
XP_014711213.1	------PLFYAVSLLLPSIFLMVMDIVGFYLPPDS----GERVSFKITLLLGYSVFLIIV	296
XP_012514621.1	------PLFYVVGLLLPSIFLMVMDIMGFYLPPDS----GERVSFKITLLLGYSVFLIIV	296
XP_008071525.1	------PLFYVVGLLLPSVFLMVMDIMGFYLPPDS----GERVSFKITLLLGYSVFLIIV	293
XP_009005082.1	------PLFYVVGLLLPSIFLMIMDIVGFYLPPNS----GERVSFKITLLLGYSVFLIIV	296
XP_010332832.1	------PLFYVVGLLLPSIFLMVMDIVGFYLPPNS----GERVSFKITLLLGYSVFLIIV	291
XP_012326058.1	------PLFYVVGLLLPSIFLMVMDIVGFYLPPNS----GERVSFKITLLLGYSVFLIIV	302
XP_017378796.1	------PLFYVVGLLLPSIFLMVMDIVGFYLPPNS----GERVSFKITLLLGYSVFLIIV	325
XP_032141374.1	------PLFYVVGLLLPSIFLMVMDIVGFYLPPNS----GERVSFKITLLLGYSVFLIIV	325
XP_032024018.1	------PLFYVVSLLLPSIFLMVMDIVGFYLPPNS----GERVSFKITLLLGYSVFLIIV	291
XP_018891497.2	------PLFYVVSLLLPSIFLMVMDIVGFYLPPNS----GERVSFKITLLLGYSVFLIVV	297
XP_030684894.1	------PLFYVVSLLLPSIFLMVMDIVGFYLPPNS----GERVSFKITLLLGYSVFLIIV	291
PNJ75904.1	------PLFYVVSLLLPSIFLMVMDIVGFYLPPNS----GERVSFKITLLLGYSVFLIIV	297

AAP35868.1	------PLFYVVSLLLPSIFLMVMDIVGFYLPPNS----GERVSFKITLLLGYSVFLIIV	297
XP_001149570.1	------PLFYVVSLLLPSIFLMVMDIVGFYLPPNS----GERVSFKITLLLGYSVFLIIV	297
XP_003805532.1	------PLFYVVSLLLPSIFLMVMDIVGFYLPPNS----GERVSFKITLLLGYSVFLIIV	297
XP_023063823.1	------PLFYVVSLLLPSIFLMVMDIVGFYLPPNS----GERVSFKITLLLGYSVFLIIV	295
XP_033060210.1	------PLFYVVSLLLPSIFLMVMDIVGFYLPPNS----GERVSFKITLLLGYSVFLIIV	295
XP_010370669.1	------PLFYVVSLLLPSIFLMVMDIVGFYLPPNS----GERVSFKITLLLGYSVFLIIV	295
XP_017738496.1	------PLFYVVSLLLPSIFLMVMDIVGFYLPPNS----GERVSFKITLLLGYSVFLIIV	295
XP_011782168.1	------PLFYVVSLLLPSIFLMVMDIVGFYLPPNS----GERVSFKITLLLGYSVFLIIV	296
EHH23440.1	------PLFYVVSLLLPSIFLMVMDIVGFYLPPNS----GERVSFKITLLLGYSVFLIIV	291
XP_003910778.3	------PLFYVVSLLLPSIFLMVMDIVGFYLPPNS----GERVSFKITLLLGYSVFLIIV	296
XP_025212649.1	------PLFYVVSLLLPSIFLMVMDIVGFYLPPNS----GERVSFKITLLLGYSVFLIIV	302
XP_008019132.1	------PLFYVVSLLLPSIFLMVMDIVGFYLPPNS----GERVSFKITLLLGYSVFLIIV	296
XP_011832421.1	------PLFYVVSLLLPSIFLMVMDIVGFYLPPNS----GERVSFKITLLLGYSVFLIIV	281
XP_011759618.1	------PLFYVVSLLLPSIFLMVMDIVGFYLPPNS----GERVSFKITLLLGYSVFLIIV	296
XP_005579749.1	------PLFYVVSLLLPSIFLMVMDIVGFYLPPNS----GERVSFKITLLLGYSVFLIIV	296
XP_011921684.1	------PLFYVVSLLLPSIFLMVMDIVGFYLPPNS----GERVSFKITLLLGYSVFLIIV	88
XP_008259391.1	------PLFYAVSLLLPSIFLMVMDIVGFYLPPDS----GERVSFKITLLLGYSVFLIIV	296
XP_012782438.1	------PLFYAVSLLLPSIFLMVMDIVGFYLPPDS----GERVSFKITLLLGYSVFLIIV	292
VTJ86076.1	------PLFYAVSLLLPSIFLMVMDIVGFYLPPDS----GERVSFKITLLLGYSVFLIIV	302
XP_015345533.1	------PLFYAVSLLLPSIFLMVMDIVGFYLPPDS----GERVSFKITLLLGYSVFLIIV	302
XP_027786432.1	------PLFYAVSLLLPSIFLMVMDIVGFYLPPDS----GERVSFKITLLLGYSVFLIIV	302
XP_026248345.1	------PLFYAVSLLLPSIFLMVMDIVGFYLPPDS----GERVSFKITLLLGYSVFLIIV	296
XP_005378209.1	------PLFYAVSLLLPSIFLMIMDIVGFYLPPDS----GERVSFKITLLLGYSVFLIIV	297
XP_004466010.1	------PLFYVVSLLLPSIFLMVMDIVGFYLPPDS----GERVSFKVTLLLGYSVFLIIV	296
XP_020035707.1	------PLFYAVSLLLPSIFLMVVDIVGFYLPPDS----GERVSFKITLLLGYSVFLIIV	290
XP_006181802.1	------PLFYAVSLLLPSIFLMVMDIVGFYLPPDS----GERVSFKITLLLGYSVFLIIV	296
XP_006207862.1	------PLFYAVSLLLPSIFLMVMDIVGFYLPPDS----GERVSFKITLLLGYSVFLIIV	296
XP_005891968.1	------PLFYAVSLLLPSIFLMVVDIVGFYLPPDS----GERVSFKITLLLGYSVFLIIV	293
XP_010828662.1	------PLFYAVSLLLPSIFLMVVDIVGFYLPPDS----GERVSFKITLLLGYSVFLIIV	293
XP_019830126.1	------PLFYAVSLLLPSIFLMVVDIVGFYLPPDS----GERVSFKITLLLGYSVFLIIV	292
XP_017914486.1	------PLFYAVSLLLPSIFLMVVDIVGFYLPPDS----GERVSFKITLLLGYSVFLIIV	292
XP_006060266.2	------PLFYAVSLLLPSIFLMVVDIVGFYLPPDS----GERVSFKITLLLGYSVFLIIV	297
XP_020728447.1	------PLFYAVSLLLPSIFLMVVDIVGFYLPPDS----GERVSFKITLLLGYSVFLIIV	297
KAF4008892.1	------PLFYAVSLLLPSIFLMVVDIVGFYLPPDS----GERVSFKITLLLGYSVFLIIV	297
KAB0348059.1	------PLFYAVSLLLPSIFLMVVDIVGFYLPPDS----GERVSFKITLLLGYSVFLIIV	280
KAB0371113.1	------PLFYAVSLLLPSIFLMVVDIVGFYLPPDS----GERVSFKITLLLGYSVFLIIV	273
MBV96963.1	------PLFYAVSLLLPSIFLMVMDIVGFYLPPDS----GERVSFKITLLLGYSVFLIIV	296
XP_007172048.1	------PLFYAVSLLLPSIFLMVMDIVGFYLPPDS----GERVSFKITLLLGYSVFLIIV	296
XP_007452270.1	------PLFYVVSLLLPSIFLMIMDIVGFYLPPDS----GERVSFKITLLLGYSVFLIIV	296
XP_023987108.1	------PLFYVVSLLLPSIFLMVMDIVGFYLPPDS----GERVSFKITLLLGYSVFLIIV	291
XP_004273432.1	------PLFYAVNLLLPSIFLMVMDIVGFYLPPDS----GERVSFKITLLLGYSVFLIIV	291
XP_004328646.1	------PLFYAVNLLLPSIFLMVTDIVGFYLPPDS----GERVSFKITLLLGYSVFLIIV	291
XP_026937670.1	------PLFYAVNLLLPSIFLMVMDIVGFYLPPDS----GERVSFKITLLLGYSVFLIIV	291
XP_030691031.1	------PLFYAVNLLLPSIFLMVMDIVGFYLPPDS----GERVSFKITLLLGYSVFLIIV	291
XP_022414870.1	------PLFYAVSLLLPSIFLMVMDIVGFYLPPDS----GERVSFKITLLLGYSVFLIIV	296
XP_029064651.1	------PLFYAVSLLLPSIFLMVMDIVGFYLPPDS----GERVSFKITLLLGYSVFLIIV	296
XP_024607566.1	------PLFYVVSLLLPSIFLMVVDIVGFYLPPDS----GERVSFKITLLLGYSVFLIIV	296
XP_032496907.1	------PLFYVVSLLLPSIFLMVMDIVGFYLPPDS----GERVSFKITLLLGYSVFLIIV	296
XP_007535559.1	------PLFYAVSLLLPSIFLMVMDIVGFYLPPDS----GERVSFKITLLLGYSVFLIIV	289
XP_031299536.1	------PLFYAVSLLLPSIFLMVMDIVGFYLPPDS----GERVSFKITLLLGYSVFLIIV	295
XP_004666516.1	------PLFYTVSLLLPSILLMVVDIVGFYLPPDS----GERVSFKITLLLGYSVFLIIV	291
XP_008830963.1	------PLFYAVSLLLPSIFLMVVDIVGFCLPPDS----GERVSFKITLLLGYSVFLIIV	296
XP_005347338.1	------PLFYAVSLLLPSIFLMVVDIVGFCLPPDS----GERVSFKITLLLGYSVFLIIV	296
XP_005069548.1	------PLFYAVSLLLPSIFLMVVDIVGFCLPPDS----GERVSFKITLLLGYSVFLIIV	296
XP_027267894.1	------PLFYAVSLLLPSIFLMVVDIVGFCLPPDS----GERVSFKITLLLGYSVFLIIV	296
OBS80992.1	------PLFYAVSLLLPSIFLMVVDIVGFCLPPDS----GERVSFKITLLLGYSVFLIIV	283
XP_006979812.1	------PLFYAISLLLPSIFLMVVDIVGFCLPPDS----GERVSFKITLLLGYSVFLIIV	302
XP_028720687.1	------PLFYAVSLLLPSIFLMVVDIVGFCLPPDS----GERVSFKITLLLGYSVFLIIV	296
XP_021489005.1	------PLFYAVSLLLPSIFLMVVDIVGFCLPPDS----GERVSFKITLLLGYSVFLIIV	297
XP_031199589.1	------PLFYAVSLLLPSIFLMVVDIVGFCLPPDS----GERVSFKITLLLGYSVFLIIV	321
XP_028617944.1	------PLFYAVSLLLPSIFLMVVDIVGFCLPPDS----GERVSFKITLLLGYSVFLIIV	111
XP_034347030.1	------PLFYAVSLLLPSIFLMVVDIVGFCLPPDS----GERVSFKITLLLGYSVFLIIV	294
NP_077370.2	------PLFYAVSLLLPSIFLMVVDIVGFCLPPDS----GERVSFKITLLLGYSVFLIIV	296
XP_032766961.1	------PLFYAVSLLLPSIFLMVVDIVGFCLPPDS----GERVSFKITLLLGYSVFLIIV	296
XP_021063186.1	------PLFYAVSLLLPSIFLMVVDIVGFCLPPDS----GERVSFKITLLLGYSVFLIIV	279
NP_001093114.1	------PLFYAVSLLLPSIFLMVVDIVGFCLPPDS----GERVSFKITLLLGYSVFLIIV	296
XP_021028379.1	------PLFYAVSLLLPSIFLMVVDIVGFCLPPDS----GERVSFKITLLLGYSVFLIIV	296
XP_004689305.1	------PLFYAVSLLLPSIFLMVMDIVGFYLPPDS----GERVSFKITLLLGYSVFLIIV	296
XP_004712871.1	------PLFYAVSLLLPSIFLMVMDIVGFYLPPES----GERVSFKITLLLGYSVFLIIV	296
XP_004382575.1	------PLFYVVSLLLPSVFLMVMDIVGFYLPPDS----GERVSFKITLLLGYSVFLIIV	296
XP_010596188.1	------PLFYAVSLILPSIFLMVMDIAGFYLPPDS----GERVSFKITLLLGYSVFLIIV	296
XP_006890855.1	------PLFYAVSLLLPSIFLMVMDIVGFYLPPDS----GERVSFKITLLLGYSVFLIIV	296
XP_006834038.1	------PLFYAVSLLLPSIFLMVMDIVGFYLPPDS----GERVSFKITLLLGYSVFLIIV	296
XP_007934716.1	------PLFYAVSLLLPSIFLMVMDIVGFYLPPDS----GERVSFKITLLLGYSVFLIIV	296
XP_012603491.1	------PLFYVVGLLLPSIFLMVMDIMGFYLPPDS----GERVSFKITLLLGYSVFLIIV	296
XP_012663606.1	------PLFYVVSLLLPSIFLMVMDIMGFYLPPDS----GERVSFKITLLLGYSVFLIIV	291
XP_028372812.1	------PLFYVVSLLLPSIFLMVMDIVGFYLPPES----GERVSFKITLLLGYSVFLIIV	296

```
XP_008589608.1        ------PLFYVVSLLLPSIFLMVMDIMGFYLPPDS----GERVSFKITLLLGYSVFLIIV      296
XP_012865077.1        ------PLFYAVSLLLPSIFLMIMDIVGFYLPPDS----GERVSFKITLLLGYSVFLIIV      296
NP_001166178.1        ------PLFYAVTLLLPSIFLMIVDIVGFYLPPDS----GERVSFKITLLLGYSVFLIIV      297
XP_004636553.1        ------PLFYAVSLLLPSIFLMIMDIVGFYLPPDS----GERVSFKITLLLGYSVFLIIV      297
XP_004856670.1        ------PLFYAVSLLLPSIFLMIMDIVGFYLPPDS----GERVSFKITLLLGYSVFLIIV      297
XP_010627744.1        ------PLFYAVNLLLPSIFLMIMDIVGFYLPPDS----GERVSFKITLLLGYSVFLIIV      297
                                    : *           :

NP_509270.1           TGQMPRS----------SEGIPLL------------------------------------      309
VDO93178.1            SEEMPTT----------SDFIPLI------------------------------------      324
PAV91580.1            SDAMPTT----------SEFVPLI------------------------------------      316
VDK46997.1            SDQMPTT----------SEFVPLI------------------------------------      282
VIO86814.1            VDEMKFVLKSVPGQRESFYNVPLL------------------------------------      351
VDP38785.1            AENTPES-----------NSTPLI------------------------------------      358
KOF68401.1            NQTLPAN----------SDEIPYI------------------------------------      277
CDJ96026.1            TESIPPT----------SEAVPLI------------------------------------      429
EFX76216.1            GESMPPT----------SEKLPLI------------------------------------      270
KAF7403848.1          RETLPPT-----------EKTPLI------------------------------------      281
KAF7427032.1          RETLPPT-----------EKTPLI------------------------------------      296
KAE9417558.1          SDITPPT----------SESIPII------------------------------------      285
KJH51504.1            NEMTPHT----------SEAVPLL------------------------------------      285
VDL69795.1            AEMSPPT----------SEALPFL------------------------------------      266
VDO32671.1            AEMTPPT----------SDAVPLI------------------------------------      300
VBB32409.1            SEMSPPT----------SEAVPLL------------------------------------      277
VDN54565.1            SDMSPPT----------SEAVPLL------------------------------------      296
EGT55171.1            SEMSPPT----------SEAVPLL------------------------------------      307
RCN52111.1            SDMSPPT----------SEAVPLL------------------------------------      312
RMX54856.1            ADIMPST----------SEVIPVI------------------------------------      308
XP_022781674.1        ADIMPST----------SEVIPVI------------------------------------      570
RNA37099.1            AENLPET----------SEYVPLI------------------------------------      312
KAA0187152.1          AEKMPET----------SESIPLI------------------------------------      485
GAU96593.1            AEKLPET----------SDYIPLI------------------------------------      310
XP_009043980.1        AESMPAT----------SEFVPLI------------------------------------      271
VDM43573.1            AEKMPET----------SDSMPLI------------------------------------      389
XP_003140283.1        AEKMPET----------SDSIPLI------------------------------------      280
VDN82010.1            AEKMPET----------SDSIPLI------------------------------------      306
VDO31501.1            AEKMPET----------SDSIPLI------------------------------------      318
VDP19591.1            ADKLPQT----------SEAIPLI------------------------------------      302
EDO32053.1            TENIPRS----------T-DIPLV------------------------------------      273
TRY67230.1            TELTPQS----------S-ETPLL------------------------------------      284
KAF7391312.1          AESMPTT----------SDAVPLI------------------------------------      299
GFG30449.1            AETMPAT----------SDAVPLL------------------------------------      309
KQS30083.1            AETMPAT----------SDAVPLL------------------------------------      331
EDW57583.2            AETMPAT----------SDAVPLL------------------------------------      467
KNC22799.1            AETMPAT----------SDAVPLL------------------------------------      277
RZF44856.1            AETMPAT----------SDAVPLL------------------------------------      297
ENN76856.1            AETMPAT----------SEAVPLL------------------------------------      303
KAF5300392.1          AETMPAT----------SDAVPLL------------------------------------      307
CAB3239999.1          AETMPAT----------SDAVPLL------------------------------------      294
PCG77624.1            AETMPAT----------SDAVPLL------------------------------------      237
PZC79131.1            AETMPAT----------SDAVPLL------------------------------------      304
KAF4083067.1          NDLLPST----------ANGTPII------------------------------------      279
XP_009924659.1        NNILPTS----------SNKPPII------------------------------------      302
XP_010123544.1        NNILPTS----------SNKPPVI------------------------------------      301
CBN81618.1            NEILPSS----------SDRAPLI------------------------------------      297
KAF3704230.1          NEILPSS----------SNSIPLI------------------------------------      293
RUS86578.1            GDMLPRR-------VESSDIVPLV------------------------------------      318
XP_034309618.1        SDSMPQN----------SENISLF------------------------------------      300
PIK58946.1            ADILPST----------N-ERPIL------------------------------------      327
VUZ42516.1            TQSTPSA----------SNTIPYL------------------------------------      305
PAA51166.1            AETTPSA----------VKNFPLI------------------------------------      345
TGZ55997.1            AESTPSA----------VRNFPLI------------------------------------      340
VDP48851.1            AESTPSA----------VKNFPLI------------------------------------      296
PVD38331.1            AESTPKA----------AASVPLI------------------------------------      291
VDK73355.1            TEIIPAT----------SISLPLI------------------------------------      283
VDD84963.1            IEIIPAT----------GDNLPLI------------------------------------      322
VDK42196.1            TEIIPAT----------SISLPLI------------------------------------      249
KFD58288.1            VEIIPST----------SLIIPLI------------------------------------      327
OUC49089.1            VEIIPST----------SLIIPLI------------------------------------      324
EYC26492.1            VEIIPST----------SLVIPLI------------------------------------      350
VDK27218.1            VEIIPST----------SLVIPLI------------------------------------      319
VDM17286.1            SKILPAG----------PKSIPLL------------------------------------      227
VDL91846.1            SETIPPT----------SLAVPLL------------------------------------      297
KAE9548540.1          IDIIPPT----------SLVIPMF------------------------------------      315
PDM74087.1            SKILPPT----------SSTIPLM------------------------------------      288
XP_024504322.1        SKILPPT----------SSTIPLM------------------------------------      291
PIO52336.1            SKILPPT----------SSTIPLM------------------------------------      287
```

TKR73865.1	SKILPPT----------SSTIPLM------------------------------------	292
RLU23395.1	SKILPPT----------SLVLPLI------------------------------------	301
XP_002427906.1	SKILPPT----------SLVLPLI------------------------------------	302
TMW47392.1	SKILPPT----------SLVLPLI------------------------------------	302
VDD83956.1	FEISPPT----------SLVVPLI------------------------------------	314
VEL33078.1	AEIIPAT----------NLVVPLI------------------------------------	244
XP_009019088.1	VEIIPPT----------SLVVPLL------------------------------------	306
KAE9536378.1	SEIIPST----------SLSLPLL------------------------------------	343
KDR23473.1	SEIIPST----------SLALPLL------------------------------------	292
KAF7266914.1	AEIIPPT----------SLAVPLL------------------------------------	325
KAF4519445.1	AEIIPPT----------SLAVPLL------------------------------------	322
OXU25983.1	AEIIPPT----------SLAIPLL------------------------------------	310
TGZ32403.1	AEIIPPT----------SLAVPLL------------------------------------	320
ELT94491.1	AEINPPT----------SLATPLI------------------------------------	310
VVC39575.1	AEIIPPT----------SLVVPLL------------------------------------	401
RVE49089.1	AEIIPPT----------SLVVPLL------------------------------------	305
KAB0800277.1	VEIIPPT----------SITVPLL------------------------------------	302
TDG52197.1	AEIIPPT----------SLTVPLL------------------------------------	288
TMW48669.1	AEIIPPT----------SLTVPLL------------------------------------	303
XP_025896085.1	SDTLPAT----------XIGTPLI------------------------------------	307
KAF2977017.1	SDTLPAT----------AVGTPLI------------------------------------	111
XP_009321837.1	SDTLPAT----------AVGTPLI------------------------------------	286
XP_009979985.1	PEPLQAA----------HLADISA------------------------------------	259
XP_028942374.1	SDTLPAT----------AIGTPLI------------------------------------	283
PKK19633.1	SDTLPAT----------AVGTPLI------------------------------------	349
XP_009894240.1	SDTLPAT----------AVGTPLI------------------------------------	402
XP_032820219.1	QDTLPAT----------PIGTPLI------------------------------------	335
XP_029429480.1	SDILPAT----------ATGTPLI------------------------------------	338
XP_030077572.1	SDTLPAT----------ATQTPLI------------------------------------	285
XP_033774596.1	SDTLPAT----------ATQTPLI------------------------------------	316
ETE72600.1	--	303
XP_018082638.1	SDTLPAT----------AIGTPLI------------------------------------	518
XP_032905410.1	SDTLPAT----------AIGTPLI------------------------------------	305
XP_020773490.1	SDTLPAT----------AIGTPLI------------------------------------	311
XP_033833634.1	SDTLPAT----------AIGTPLI------------------------------------	337
XP_030215795.1	SDTLPAT----------AIGTPLI------------------------------------	310
XP_030602980.1	SDTLPAT----------AIGTPLI------------------------------------	316
XP_004573543.1	SDTLPAT----------AIGTPLI------------------------------------	310
XP_005916159.1	SDTLPAT----------AIGTPLI------------------------------------	310
XP_013132089.1	SDTLPAT----------AIGTPLI------------------------------------	310
XP_031603488.1	SDTLPAT----------AIGTPLI------------------------------------	316
XP_005725536.1	SDTLPAT----------AIGTPLI------------------------------------	316
XP_026038075.1	SDTLPAT----------AIGTPLI------------------------------------	316
CAF96649.1	SDTLPAT----------AIGTPLI------------------------------------	301
XP_023818570.1	SDTLPAT----------AIGTPLI------------------------------------	306
RVE64289.1	SDTLPAT----------AIGTPLI------------------------------------	341
XP_024121971.1	SDTLPAT----------AIGTPLI------------------------------------	312
XP_015817559.1	SDTLPAT----------AIGTPLI------------------------------------	296
XP_015225670.1	SDTLPAT----------AIGTPLI------------------------------------	295
XP_012722063.1	SDTLPAT----------AIGTPLI------------------------------------	310
XP_032431307.1	SDTLPAT----------AIGTPLI------------------------------------	310
XP_014328329.1	SDTLPAT----------AIGTPLI------------------------------------	310
XP_027886578.1	SDTLPAT----------AIGTPLI------------------------------------	310
XP_008426791.1	SDTLPAT----------AIGTPLI------------------------------------	310
XP_014838686.1	SDTLPAT----------AIGTPLI------------------------------------	310
XP_014885967.1	SDTLPAT----------AIGTPLI------------------------------------	310
XP_033954312.1	SDTLPAT----------AIGTPLI------------------------------------	310
XP_034089244.1	SDTLPAT----------AIGTPLI------------------------------------	310
XP_010780064.1	SDTLPAT----------AIGTPLI------------------------------------	326
XP_033970261.1	SDTLPAT----------AIGTPLI------------------------------------	326
XP_029375575.1	SDTLPAT----------AIGTPLI------------------------------------	347
KAF0023022.1	SDTLPAT----------AIGTPLI------------------------------------	343
XP_019952614.1	SDTLPAT----------AIGTPLI------------------------------------	299
XP_034463117.1	SDTLPAT----------AIGTPLI------------------------------------	310
XP_029924656.1	SDTLPAT----------AIGTPLI------------------------------------	311
XP_028323228.1	SDTLPAT----------AIGTPLI------------------------------------	310
TNM84480.1	SDTLPGH----------RHRNPVD------------------------------------	310
XP_029703788.1	SDTLPAT----------AIGTPLI------------------------------------	310
XP_030010368.1	SDTLPAT----------AIGTPLI------------------------------------	313
XP_019725056.1	SDTLPAT----------AIGTPLI------------------------------------	311
XP_034034934.1	SDTLPAT----------AIGTPLI------------------------------------	299
XP_029956880.1	SDTLPAT----------AIGTPLI------------------------------------	310
XP_008331307.3	SDTLPAT----------AIGTPLI------------------------------------	343
XP_031724845.1	SDTLPAT----------AIGTPLI------------------------------------	311
XP_013859395.1	SDTLPAT----------AIGTPLI------------------------------------	310
XP_017275335.1	SDTLPAT----------AIGTPLI------------------------------------	310
XP_020496197.1	SDTLPAT----------AIGTPLI------------------------------------	310

XP_029030400.1	SDTLPAT----------AIGTPLI------------------------------------	310
XP_022060538.1	SDTLPAT----------AIGTPLI------------------------------------	311
XP_023150584.1	SDTLPAT----------AIGTPLI------------------------------------	311
XP_029303892.1	SDTLPAT----------AIGTPLI------------------------------------	334
XP_020454172.1	SDTLPAT----------AIGTPLI------------------------------------	309
TKS83244.1	SDTLPAT----------AIGTPLI------------------------------------	297
XP_022616583.1	SDTLPAT----------AIGTPLI------------------------------------	343
XP_030293172.1	SDTLPAT----------AIGTPLI------------------------------------	310
XP_027129539.1	SDTLPAT----------AIGTPLI------------------------------------	310
XP_034539538.1	SDTLPAT----------AIGTPLI------------------------------------	310
XP_008277330.1	SDTLPAT----------AIGTPLI------------------------------------	310
XP_028276940.1	SDTLPAT----------AIGTPLI------------------------------------	310
XP_023263433.1	SDTLPAT----------AIGTPLI------------------------------------	310
XP_034406336.1	SDTLPAT----------AIGTPLI------------------------------------	310
XP_026184460.1	SDTLPAT----------AIGTPLI------------------------------------	310
KAF1378228.1	SDTLPAT----------AIGTPLI------------------------------------	295
XP_028450365.1	SDTLPAT----------AIGTPLI------------------------------------	310
XP_031163851.1	SDTLPAT----------AIGTPLI------------------------------------	310
XP_032389084.1	SDTLPAT----------AIGTPLI------------------------------------	310
XP_033494682.1	SDTLPAT----------AIGTPLI------------------------------------	310
XP_018544782.1	SDTLPAT----------AIGTPLI------------------------------------	310
XP_026228189.1	SDTLPAT----------AIGTPLI------------------------------------	310
XP_028976606.1	SDTLPAT----------AIGTPLIGEMQLAVKREWGVPVFSDYGTPSGVLGXHGSLPLSP	346
CAB1352378.1	--	275
XP_023866849.1	SDTLPAT----------AIGTPLI------------------------------------	310
XP_013992832.1	SDTLPAT----------AIGTPLI------------------------------------	310
XP_029546688.1	SDTLPAT----------AIGTPLI------------------------------------	310
XP_021481546.1	SDTLPAT----------AIGTPLI------------------------------------	310
XP_020339889.1	SDTLPAT----------AIGTPLI------------------------------------	310
XP_029481972.1	SDTLPAT----------AIGTPLI------------------------------------	310
KPP68743.1	SDTLPAT----------AIGTPLI------------------------------------	287
XP_023669383.1	SDTLPAT----------AIGTPLI------------------------------------	309
KAA0720096.1	SDTLPAT----------AIGTPLI------------------------------------	215
XP_009293684.1	SDTLPAT----------AIGTPLI------------------------------------	309
XP_026090784.1	SDTLPAT----------AIGTPLI------------------------------------	309
XP_018918715.1	SDTLPAT----------AIGTPLI------------------------------------	309
KAF4098549.1	SDTLPAT----------AIGTPLI------------------------------------	309
XP_016084173.1	SDTLPAT----------AIGTPLI------------------------------------	309
XP_016332759.1	SDTLPAT----------AIGTPLI------------------------------------	309
XP_016398039.1	SDTLPAT----------AIGTPLI------------------------------------	309
XP_026989970.1	SDTLPAT----------AIGTPLI------------------------------------	309
TSQ12698.1	SDTLPAT----------AIGTPLI------------------------------------	309
XP_017347546.1	SDTLPAT----------AIGTPLI------------------------------------	304
XP_026794616.2	SDTLPAT----------AIGTPLI------------------------------------	309
XP_026861889.1	SDTLPAT----------AIGTPLI------------------------------------	309
XP_017575347.1	SDTLPAT----------AIGTPLI------------------------------------	309
XP_022531596.1	SDTLPAT----------AIGTPLI------------------------------------	309
XP_012691019.2	SDTLPAT----------AIGTPLI------------------------------------	292
XP_028839723.1	SDTLPAT----------AIGTPLI------------------------------------	309
XP_030638860.1	SDTLPAT----------AIGTPLI------------------------------------	291
XP_007882964.1	SDTLPAT----------AIGTPLI------------------------------------	287
GCB70244.1	SDTLPAT----------AIGTPLI------------------------------------	288
GCC26242.1	SDTLPAT----------AIGTPLI------------------------------------	309
XP_020387034.1	SDTLPAT----------AIGTPLI------------------------------------	300
XP_028931940.1	SDTLPAT----------AIGTPLI------------------------------------	315
XP_031762490.1	SDTLPAT----------AIGTPLI------------------------------------	303
PIO32240.1	SDTLPAT----------AIGTPLI------------------------------------	101
XP_018425466.1	SDTLPAT----------AIGTPLI------------------------------------	304
XP_006642351.1	SDTLPAT----------AIGTPLI------------------------------------	326
XP_028665372.1	SDTLPAT----------AIGTPLI------------------------------------	309
GCF49814.1	SDTLPAT----------AIGTPLI------------------------------------	290
XP_015277816.1	SDTLPAT----------AIGTPLI------------------------------------	313
XP_005987340.2	SDTLPAT----------AIGTPLI------------------------------------	293
XP_019339534.1	SDTLPAT----------AIGTPLI------------------------------------	310
XP_025063414.1	SDTLPAT----------AIGTPLI------------------------------------	310
XP_019367488.1	SDTLPAT----------AIGTPLI------------------------------------	310
XP_019412078.1	SDTLPAT----------AIGTPLI------------------------------------	310
XP_014434314.1	SDTLPAT----------AIGTPLI------------------------------------	310
XP_007060556.1	SDTLPAT----------AIGTPLI------------------------------------	304
XP_024072175.1	SDTLPAT----------AIGTPLI------------------------------------	289
XP_008170567.1	SDTLPAT----------AIGTPLI------------------------------------	310
XP_034610543.1	SDTLPAT----------AIGTPLI------------------------------------	310
XP_030394127.1	SDTLPAT----------AIGTPLI------------------------------------	310
XP_032651960.1	SDTLPAT----------AIGTPLI------------------------------------	310
XP_025020083.1	SDTLPAT----------AIGTPLI------------------------------------	310
XP_026536833.1	SDTLPAT----------AIGTPLI------------------------------------	310
XP_026564787.1	SDTLPAT----------AIGTPLI------------------------------------	310

XP_032084675.1	SDTLPAT----------AIGTPLI------------------------------------	295
XP_029139341.1	SDTLPAT----------AIGTPLI------------------------------------	310
XP_034281006.1	SDTLPAT----------AIGTPLI------------------------------------	310
XP_020649062.1	SDTLPAT----------AIGTPLI------------------------------------	310
XP_008117087.1	SDTLPAT----------AIGTPLI------------------------------------	310
XP_028564324.1	SDTLPAT----------AIGTPLI------------------------------------	316
XP_033028155.1	SDTLPAT----------AIGTPLI------------------------------------	315
XP_025963249.1	SDTLPAT----------AIGTPLI------------------------------------	297
XP_009668348.1	SDTLPAT----------AIGTPLI------------------------------------	309
XP_013798935.1	SDTLPAT----------AIGTPLI------------------------------------	293
XP_025913685.1	SDTLPAT----------AIGTPLI------------------------------------	318
XP_013042552.1	SDTLPAT----------AVGTPLI------------------------------------	316
XP_005030458.2	SDTLPAT----------AVGTPLI------------------------------------	309
XP_032057953.1	SDTLPAT----------AVGTPLI------------------------------------	292
XP_021232050.1	SDTLPAT----------AVGTPLI------------------------------------	311
OXB62403.1	SDTLPAT----------AVGTPLI------------------------------------	310
OXB81319.1	SDTLPAT----------AVGTPLI------------------------------------	292
XP_010722007.1	SDTLPAT----------AVGTPLI------------------------------------	311
XP_015739349.1	SDTLPAT----------AVGTPLI------------------------------------	311
XP_031455498.1	SDTLPAT----------AVGTPLI------------------------------------	312
POI27435.1	SDTLPAT----------AVGTPLI------------------------------------	287
XP_004948120.1	SDTLPAT----------AVGTPLI------------------------------------	312
XP_032851190.1	SDTLPAT----------AVGTPLI------------------------------------	307
XP_010007255.1	SDTLPAT----------AVGTPLI------------------------------------	309
XP_030320702.1	SDTLPAT----------AVGTPLI------------------------------------	280
XP_010191940.1	SATLPAT----------HLAGTPT------------------------------------	307
XP_027737112.1	SDTLPAT----------AVGTPLI------------------------------------	305
XP_027555032.1	SDTLPAT----------AVGTPLI------------------------------------	310
XP_032565370.1	SDTLPAT----------AVGTPLI------------------------------------	356
XP_027511217.1	SDTLPAT----------AVGTPLI------------------------------------	455
XP_027593499.1	SDTLPAT----------AVGTPLI------------------------------------	406
XP_017664924.1	SDTLPAT----------AVGTPLI------------------------------------	307
XP_029817938.1	SDTLPAT----------AVGTPLI------------------------------------	289
XP_005058641.1	SDTLPAT----------AVGTPLI------------------------------------	340
XP_021385804.1	SDTLPAT----------AVGTPLI------------------------------------	309
XP_030146687.2	SDTLPAT----------AVGTPLI------------------------------------	354
KAF4796420.1	SDTLPAT----------AVGTPLI------------------------------------	309
XP_032937581.1	SDTLPAT----------AVGTPLI------------------------------------	356
XP_031989659.1	SDTLPAT----------AVGTPLI------------------------------------	310
XP_010402086.1	SDTLPAT----------AVGTPLI------------------------------------	309
XP_017594069.1	SDTLPAT----------AVGTPLI------------------------------------	280
XP_014115268.1	SDTLPAT----------AVGTPLI------------------------------------	334
XP_023797108.1	SDTLPAT----------AVGTPLI------------------------------------	280
XP_033375614.1	SDTLPAT----------AVGTPLI------------------------------------	280
XP_014740121.1	SDTLPAT----------AVGTPLI------------------------------------	454
RLV83430.1	SDTLPAT----------AVGTPLI------------------------------------	300
XP_009096098.2	SDTLPAT----------AVGTPLI------------------------------------	415
TRZ15870.1	SDTLPAT----------AVGTPLI------------------------------------	310
RMB91935.1	SDTLPAT----------AVGTPLI------------------------------------	280
XP_030820843.1	SDTLPAT----------AVGTPLI------------------------------------	356
XP_014165179.1	SDTLPAT----------AVGTPLI------------------------------------	358
XP_026653582.1	SDTLPAT----------AVGTPLI------------------------------------	240
PKU35975.1	SDTLPAT----------AVGTPLI------------------------------------	312
XP_014805072.1	SDTLPAT----------AVGTPLI------------------------------------	306
XP_009818330.1	SDTLPAT----------HLADIST------------------------------------	77
OPJ68307.1	SDTLPAT----------AVGTPLI------------------------------------	309
XP_008936289.1	SDTLPAT----------AVGTPLI------------------------------------	310
XP_010287046.1	SDTLPAT----------AVGTPLI------------------------------------	240
XP_005240140.2	SDTLPAT----------AVGTPLI------------------------------------	369
XP_005437752.2	SDTLPAT----------AVGTPLI------------------------------------	369
KFV74811.1	SDTLPAT----------AVGTPLI------------------------------------	274
XP_010018389.1	SDTLPAT----------AVGTPLI------------------------------------	317
KQK78711.1	SDTLPAT----------AVGTPLI------------------------------------	309
XP_009570162.1	SDTLPAT----------AVGTPLI------------------------------------	308
KFP11268.1	SDTLPAT----------AVGTPLI------------------------------------	272
KFQ98910.1	SDTLPAT----------AVGTPLI------------------------------------	291
XP_012985202.3	SDTLPAT----------AVGTPLI------------------------------------	309
XP_010573388.1	SDTLPAT----------AVGTPLI------------------------------------	308
XP_029879496.1	SDTLPAT----------AVGTPLI------------------------------------	280
XP_030361086.1	SDTLPAT----------AVGTPLI------------------------------------	361
KFM00668.1	SDTLPAT----------AVGTPLI------------------------------------	292
KAF1479074.1	SDTLPAT----------AVGTPLI------------------------------------	272
KAF1651161.1	SDTLPAT----------AVGTPLI------------------------------------	273
KAF1673648.1	SDTLPAT----------AVGTPLI------------------------------------	273
KAF1493319.1	SDTLPAT----------AVGTPLI------------------------------------	273
KAF1584157.1	SDTLPAT----------AVGTPLI------------------------------------	273
KAF1571723.1	SDTLPAT----------AVGTPLI------------------------------------	273

```
KAF1533169.1        SDTLPAT----------AVGTPLI------------------------------------    273
KAF1638955.1        SDTLPAT----------AVGTPLI------------------------------------    273
KAF1549972.1        SDTLPAT----------AVGTPLI------------------------------------    273
KAF1606914.1        SDTLPAT----------AVGTPLI------------------------------------    273
KAF1510957.1        SDTLPAT----------AVGTPLI------------------------------------    273
KAF1498899.1        SDTLPAT----------AVGTPLI------------------------------------    273
KAF1411525.1        SDTLPAT----------AVGTPLI------------------------------------    273
KAF1429205.1        SDTLPAT----------AVGTPLI------------------------------------    273
XP_005334318.1      SDTLPAT----------AIGTPLI------------------------------------    299
XP_014395552.1      SDTLPAT----------AIGTPLI------------------------------------    299
KAB0404854.1        SDTLPAT----------AIGTPLI------------------------------------    295
XP_007494828.1      SDTLPAT----------AIGTPLI------------------------------------    310
XP_003764254.1      SDTLPAT----------AIGTPLI------------------------------------    310
XP_020845489.1      SDTLPAT----------AIGTPLI------------------------------------    310
XP_027703160.1      SDTLPAT----------AIGTPLI------------------------------------    310
XP_017523929.1      SDTLPAT----------AIGTPLI------------------------------------    307
XP_008688428.1      -----------------AIGTPLI------------------------------------    255
XP_029812166.1      SDTLPAT----------AIGTPLI------------------------------------    324
XP_025749781.1      SDTLPAT----------AIGTPLI------------------------------------    310
XP_004416432.1      SDTLPAT----------AIGTPLI------------------------------------    310
XP_027436262.1      SDTLPAT----------AIGTPLI------------------------------------    310
XP_032284025.1      SDTLPAT----------AIGTPLI------------------------------------    310
XP_006735421.1      SDTLPAT----------AIGTPLI------------------------------------    310
XP_021552166.1      SDTLPAT----------AIGTPLI------------------------------------    310
XP_026361066.1      SDTLPAT----------AIGTPLI------------------------------------    310
XP_034523598.1      SDTLPAT----------AIGTPLI------------------------------------    310
NP_001297113.1      SDTLPAT----------AIGTPLI------------------------------------    310
XP_032215488.1      SDTLPAT----------AIGTPLI------------------------------------    310
VCX31483.1          SDTLPAT----------AIGTPLI------------------------------------    310
XP_022369003.1      SDTLPAT----------AIGTPLI------------------------------------    310
XP_032694248.1      SDTLPAT----------AIGTPLI------------------------------------    310
NP_001041584.1      SDTLPAT----------AIGTPLI------------------------------------    310
XP_025862501.1      SDTLPAT----------AIGTPLI------------------------------------    310
KAF0873564.1        SDTLPAT----------AIGTPLI------------------------------------    452
XP_025784751.1      SDTLPAT----------AIGTPLI------------------------------------    310
XP_007075625.1      SDTLPAT----------APGTPLI------------------------------------    446
XP_030189489.1      SDTLPAT----------AIGTPLI------------------------------------    310
XP_019324655.1      SDTLPAT----------AIGTPLI------------------------------------    411
XP_023094886.1      SDTLPAT----------AIGTPLI------------------------------------    446
XP_026892357.1      SDTLPAT----------AIGTPLI------------------------------------    310
XP_016004457.1      SDTLPAT----------AIGTPLI------------------------------------    305
XP_006912860.1      SDTLPAT----------AIGTPLI------------------------------------    305
XP_011363679.1      SDTLPAT----------AIGTPLI------------------------------------    305
XP_016076060.1      SDTLPAT----------AIGTPLI------------------------------------    310
XP_008148371.1      SDTLPAT----------AIGTPLI------------------------------------    310
XP_006093568.1      SDTLPAT----------AIGTPLI------------------------------------    310
XP_006761601.1      SDTLPAT----------AIGTPLI------------------------------------    310
XP_024426743.1      SDTLPAT----------AIGTPLI------------------------------------    305
XP_019520779.1      SDTLPAT----------AIGTPLI------------------------------------    310
XP_032976539.1      SDTLPAT----------AIGTPLI------------------------------------    310
ELW64270.1          SDTLPAT----------AIGTPLI------------------------------------    314
XP_004427367.1      SDTLPAT----------AIGTPLI------------------------------------    310
XP_008529353.1      SDTLPAT----------AIGTPLI------------------------------------    310
NP_001288165.1      SDTLPAT----------AIGTPLI------------------------------------    310
XP_014711213.1      SDTLPAT----------AIGTPLI------------------------------------    310
XP_012514621.1      SDTLPAT----------AIGTPLI------------------------------------    310
XP_008071525.1      SDTLPAT----------AIGTPLI------------------------------------    307
XP_009005082.1      SDTLPAT----------AIGTPLI------------------------------------    310
XP_010332832.1      SDTLPAT----------AIGTPLI------------------------------------    305
XP_012326058.1      SDTLPAT----------AIGTPLI------------------------------------    316
XP_017378796.1      SDTLPAT----------AIGTPLI------------------------------------    339
XP_032141374.1      SDTLPAT----------AIGTPLI------------------------------------    339
XP_032024018.1      SDTLPAT----------AIGTPLI------------------------------------    305
XP_018891497.2      SDTLPAT----------AIGTPLI------------------------------------    311
XP_030684894.1      SDTLPAT----------AIGTPLI------------------------------------    305
PNJ75904.1          SDTLPAT----------AIGTPLI------------------------------------    311
AAP35868.1          SDTLPAT----------AIGTPLI------------------------------------    311
XP_001149570.1      SDTLPAT----------AIGTPLI------------------------------------    311
XP_003805532.1      SDTLPAT----------AIGTPLI------------------------------------    311
XP_023063823.1      SDTLPAT----------AIGTPLI------------------------------------    309
XP_033060210.1      SDTLPAT----------AIGTPLI------------------------------------    309
XP_010370669.1      SDTLPAT----------AIGTPLI------------------------------------    309
XP_017738496.1      SDTLPAT----------AIGTPLI------------------------------------    309
XP_011782168.1      SDTLPAT----------AIGTPLI------------------------------------    310
EHH23440.1          SDTLPAT----------AIGTPLIGKVSPGSRAQSGEKPT----PSHLLYVSLASALDCT    337
XP_003910778.3      SDTLPAT----------AIGTPLI------------------------------------    310
XP_025212649.1      SDTLPAT----------AIGTPLI------------------------------------    316
```

XP_008019132.1	SDTLPAT----------AIGTPLI------------------------------------	310
XP_011832421.1	SDTLPAT----------AIGTPLI------------------------------------	295
XP_011759618.1	SDTLPAT----------AIGTPLI------------------------------------	310
XP_005579749.1	SDTLPAT----------AIGTPLI------------------------------------	310
XP_011921684.1	SDTLPAT----------AIGTPLI------------------------------------	102
XP_008259391.1	SDTLPAT----------AIGTPLI------------------------------------	310
XP_012782438.1	SDTLPAT----------AIGTPLI------------------------------------	306
VTJ86076.1	SDTLPAT----------AIGTPLI------------------------------------	316
XP_015345533.1	SDTLPAT----------AIGTPLI------------------------------------	316
XP_027786432.1	SDTLPAT----------AIGTPLI------------------------------------	316
XP_026248345.1	SDTLPAT----------AIGTPLI------------------------------------	310
XP_005378209.1	SDTLPAT----------AIGTPLI------------------------------------	311
XP_004466010.1	SDTLPAT----------AIGTPLI------------------------------------	310
XP_020035707.1	SDTLPAT----------AIGTPLI------------------------------------	304
XP_006181802.1	SDTLPAT----------AIGTPLI------------------------------------	310
XP_006207862.1	SDTLPAT----------AIGTPLI------------------------------------	310
XP_005891968.1	SDTLPAT----------AIGTPLI------------------------------------	307
XP_010828662.1	SDTLPAT----------AIGTPLI------------------------------------	307
XP_019830126.1	SDTLPAT----------AIGTPLI------------------------------------	306
XP_017914486.1	SDTLPAT----------AIGTPLI------------------------------------	306
XP_006060266.2	SDTLPAT----------AIGTPLI------------------------------------	311
XP_020728447.1	SDTLPAT----------AIGTPLI------------------------------------	311
KAF4008892.1	SDTLPAT----------AIGTPLI------------------------------------	311
KAB0348059.1	SDTLPAT----------AIGTPLI------------------------------------	294
KAB0371113.1	SDTLPAT----------AIGTPLI------------------------------------	287
MBV96963.1	SDTLPAT----------AIGTPLI------------------------------------	310
XP_007172048.1	SDTLPAT----------AIGTPLI------------------------------------	310
XP_007452270.1	SDTLPAT----------AIGTPLI------------------------------------	310
XP_023987108.1	SDTLPAT----------AIGTPLI------------------------------------	305
XP_004273432.1	SDTLPAT----------AIGTPLI------------------------------------	305
XP_004328646.1	SDTLPAT----------AIGTPLI------------------------------------	305
XP_026937670.1	SDTLPAT----------AIGTPLI------------------------------------	305
XP_030691031.1	SDTLPAT----------AIGTPLI------------------------------------	305
XP_022414870.1	SDTLPAT----------AIGTPLI------------------------------------	310
XP_029064651.1	SDTLPAT----------AIGTPLI------------------------------------	310
XP_024607566.1	SDTLPAT----------AIGTPLI------------------------------------	310
XP_032496907.1	SDTLPAT----------AIGTPLI------------------------------------	310
XP_007535559.1	SDTLPAT----------AIGTPLI------------------------------------	303
XP_031299536.1	SDTLPAT----------AIGTPLI------------------------------------	309
XP_004666516.1	SDTLPAT----------AIGTPLI------------------------------------	305
XP_008830963.1	SDTLPAT----------AIGTPLI------------------------------------	310
XP_005347338.1	SDTLPAT----------AIGTPLI------------------------------------	310
XP_005069548.1	SDTLPAT----------AIGTPLI------------------------------------	310
XP_027267894.1	SDTLPAT----------AIGTPLI------------------------------------	310
OBS80992.1	SDTLPAT----------AIGTPLI------------------------------------	297
XP_006979812.1	SDTLPAT----------AIGTPLI------------------------------------	316
XP_028720687.1	SDTLPAT----------AIGTPLI------------------------------------	310
XP_021489005.1	SDTLPAT----------AIGTPLI------------------------------------	311
XP_031199589.1	SDTLPAT----------AIGTPLI------------------------------------	335
XP_028617944.1	SDTLPAT----------AIGTPLI------------------------------------	125
XP_034347030.1	SDTLPAT----------AIGTPLI------------------------------------	308
NP_077370.2	SDTLPAT----------AIGTPLI------------------------------------	310
XP_032766961.1	SDTLPAT----------AIGTPLI------------------------------------	310
XP_021063186.1	SDTLPAT----------AIGTPLI------------------------------------	293
NP_001093114.1	SDTLPAT----------AIGTPLI------------------------------------	310
XP_021028379.1	SDTLPAT----------AIGTPLI------------------------------------	310
XP_004689305.1	SDTLPAT----------AIGTPLI------------------------------------	310
XP_004712871.1	SDTLPAT----------AIGTPLI------------------------------------	310
XP_004382575.1	SDTLPAT----------AIGTPLI------------------------------------	310
XP_010596188.1	SDTLPAT----------AIGTPLI------------------------------------	310
XP_006890855.1	SDTLPAT----------AIGTPLI------------------------------------	310
XP_006834038.1	SDTLPAT----------AIGTPLI------------------------------------	310
XP_007934716.1	SDTLPAT----------AIGTPLI------------------------------------	310
XP_012603491.1	SDTLPAT----------AIGTPLI------------------------------------	310
XP_012663606.1	SDTLPAT----------AIGTPLI------------------------------------	305
XP_028372812.1	SDTLPAT----------AIGTPLI------------------------------------	310
XP_008589608.1	SDTLPAT----------AIGTPLI------------------------------------	310
XP_012865077.1	SDTLPAT----------AIGTPLI------------------------------------	310
NP_001166178.1	SDTLPAT----------AIGTPLI------------------------------------	311
XP_004636553.1	SDTLPAT----------AIGTPLI------------------------------------	311
XP_004856670.1	SDTLPAT----------AIGTPLI------------------------------------	311
XP_010627744.1	SDTLPAT----------AIGTPLI------------------------------------	311

Transmembrane domain 3

NP_509270.1	GMYVLIEFVISVIAVLVSV-VI---IFAHERMLY--LD-ATPPYWVCK-----LFSDD--	355

```
VDO93178.1          GTR---------------------------QGRY--GK-RLSYKWKKF-----IMKTM--     347
PAV91580.1          AWFYLSIIVIVSIGTFLTS-VV---LSVQSRRQY--GK-NPPRYVRYL-----FFIWM--     362
VDK46997.1          AWFYLSIIIIVSIGTFLTS-VV---LSVQSRRQY--GR-NPPLYVRYY-----FFVII--     328
VIO86814.1          AVFHMVQMLIISVATCTSS-VFVYLEKYALRNQY--IE-AIPWWLRFLSAKRLFCCYV--     405
VDP38785.1          GTFLTMVMTLNSISLIFAT-IV---LHIHRRSKNDPCP-TPHPLLWRL-----IIGIF--     406
KOF68401.1          GIYFASCMMLVSLSCVMAV-IT---VNIHYKSSM--GT-TFPAWMEKL-----QQSWL--     323
CDJ96026.1          GMYYVSSLFMVCLATCVNV-IT---LNMHRNGAANQGR-HVPCWMEKW-----VLGYL--     477
EFX76216.1          GLYYGVTISLVSFATGLSV-VT---LNIHHRGMR--GR-GVPPLVKKI-----VFGVL--     316
KAF7403848.1        SLYYGVSICLVSFASGLAV-LT---LNLHHRGTR------VPEVVRSL-----FLHKL--     324
KAF7427032.1        SLYYGVSICLVSFASGLAV-LT---LNLHHRGTR------VPEVVRSL-----FLHKL--     339
KAE9417558.1        AVFFSLSMLILGLSIIATL-VI---INVHFRSPR--TH-QMGEWTQLV-----FLEWL--     331
KJH51504.1          GVFFQSCMVISVLATSFTV-FV---QSYHFRSHNN-TQ-QMTFWIRFI-----LLEWI--     332
VDL69795.1          GAFFAVCMFTCACCVVATT-LA---LNFHHRNMY--SH-EMGDMFRCV-----MLNWL--     312
VDO32671.1          GVFFSCCMLVISASVVFTV-LI---LNLHFRSPD--TH-EMSPIMQKV-----FMEWL--     346
VBB32409.1          GVFFSCCMIVVTASTVFTV-YV---LNLHYRTSE--TH-EMGTLTKTL-----LLYWL--     323
VDN54565.1          GIFFSCCMIVVTASTVFTV-YV---LNLHYRTPE--TH-EMGTLTRTI-----LLYWL--     342
EGT55171.1          GIFFTCCMIVVTASTVFTV-YV---LNLHYRTPE--TH-DMGPWTRNL-----LLYWI--     353
RCN52111.1          GIFFSCCMIVVTASTVFTV-YV---LNLHYRTPE--TH-EMSATMRSI-----LLYWL--     358
RMX54856.1          SIYFSGAIFEVSLALLATC-FI---LKCHFHDPA--SS-DMPVWIRHI-----VFNWM--     354
XP_022781674.1      SIYFSGAIFEVSLALLATC-FI---LKCHFHDPA--SS-DMPVWIRHI-----VLKWM--     616
RNA37099.1          SIYLTVIMFTASLSVICTV-FV---LNLHHRRSA--KR-RVPNWLRRI-----FIGPD--     358
KAA0187152.1        GIYLTAVMTITSISIIMTV-IV---LNCHYRGPI--QK-EVSPWMRRI-----FLDSG--     531
GAU96593.1          GIYLTVVMTMTSISVIMTV-IV---LNFHWRAAE--KY-VVPPWIQKW-----ILGKL--     356
XP_009043980.1      GIYLTVTMAMTSMSIILTV-FV---LQLHHVGPH--QK-PVPRWMKSL-----VFNIL--     317
VDM43573.1          GIYLTVVMAMTSVSVVMTV-MV---LNFHHRGPF--NR-AVPKWVRRF-----VLQRL--     435
XP_003140283.1      GIYLTIIMLLTSISVIMTV-MV---LNFHHRGPF--DR-PVPEWVHIL-----VLQKL--     326
VDN82010.1          GIYLTIIMLLTSISVIMTV-MV---LNFHHRGPF--DR-PIPEWVRVL-----VLQKL--     352
VDO31501.1          GIYLTIIMLLTSISVIMTV-MV---LNFHHRGPF--DR-PIPEWVRVL-----VLQKL--     364
VDP19591.1          GIYFSCTMFMCSLSIVFTV-LV---LNYHHRSAD--CI-AVPAWVTKL-----MCMEP--     348
EDO32053.1          SKFFMASMIQIALSLAATC-VV---IRFDT---T--KK-PMSSVIKLV-----VNDWL--     316
TRY67230.1          GIYFSCIMVMVAASVVDAI-LV---LNFHRQFGT--EN-TMPYIIKFV-----FLQWL--     330
KAF7391312.1        GSYFNCIMFMVASSVVLTV-MV---LNYHHRTPD--RY-VMPTWIKKV-----FLQWL--     345
GFG30449.1          GTYFNCIMFMVASSVVSTI-LI---LNYHHRNAD--TH-EMSPWVSSE-----ALQSP--     355
KQS30083.1          GTYFNCIMFMVASSVVSTI-LI---LNYHHRNPD--TH-EMSEWTRTG-----WLRMS--     377
EDW57583.2          GTYFNCIMFMVASSVVSTI-LI---LNYHHRNAD--TH-EMSEWIRIV-----FLCWL--     513
KNC22799.1          GTYFNCIMFMVASSVVSTI-LI---LNYHHRNAD--TH-EMSEWIRVV-----FLCWL--     323
RZF44856.1          GTYFNCIMFMVASSVVSTI-LI---LNYHHRNAD--TH-EMSPWIKAV-----FLNWM--     343
ENN76856.1          GTYFNCIMFMVASSVVSTI-LI---LNYHHRNAD--TH-IMSPWFKLV-----FLIWL--     349
KAF5300392.1        GTYFNCIMFMVASSVVSTI-LI---LNYHHRNAD--TH-EMSAWIKIV-----FLYWL--     353
CAB3239999.1        GTYFNCIMFMVASSVVSTI-LI---LNYHHRHSD--TH-EMSEWIRCV-----FLYWL--     340
PCG77624.1          GTYFNCIMFMVASSVVSTI-LI---LNYHHRHAD--TH-EMSDWIRCV-----FLYWL--     283
PZC79131.1          GTYFNCIMFMVASSVVSTI-LI---LNYHHRHAD--TH-EMSDWIRCV-----FLYWL--     350
KAF4083067.1        GELQLLH-------MITTL-L----LHSGYVPLC--VP----------------------     303
XP_009924659.1      VVFFLGTFLLMIMAVLDTF-FL---LHQQHKSLR--LD-K--------------------     335
XP_010123544.1      VVFFLGTFLLMIMAVLDTF-FL---LHQKHKSLR--LD-K--------------------     334
CBN81618.1          ALYCIGIFGLMMLSLVETI-VV---MSLRARDSQ--DN-E--------------------     330
KAF3704230.1        AVYCIGIFALMMLSLFETI-FV---MYLMEKDSA--SD-G--------------------     326
RUS86578.1          TIYLFVLLVISVLTVMVAI-II---VWLHHKDEQ--ET-K-RQEATHT-----FRNLF--     363
XP_034309618.1      VVYVTIQIFLSGVSVVMET-VV---LRLYYTEVC--SSLPKPAWLREK-----NILTS--     347
PIK58946.1          SSYLISTFVLACLAVPASL-LN---INIAYGDRS--ILVFKYTFSRKL-----FLEYL--     374
VUZ42516.1          GYYYCLNMVLIAIFTFFSV-IA---VQIHFRKDK--TK-PLPSWLSRF-----VKTSS--     351
PAA51166.1          GYYYCLNMIMITLSTFLAV-IV---VNLHFGADR--RA-PLSPFVRQL-----VIEGI--     391
TGZ55997.1          GVFFCLNMVMITLSTVLAT-LV---IHLYFRGDR--NG-SVPAFLRRI-----VIEGI--     386
VDP48851.1          GVFFCLNMIMITLSTFLAT-MV---IHLYFRGDR--KG-AVPYFLRRA-----YQNRC--     342
PVD38331.1          GVYFCLNMVMITMSTVLTT-VV---ANMFYRGVR--IN-RAPKWLRVL-----MIDII--     337
VDK73355.1          GKYLLFTMVMVTLSVIVTV-IS---LNLHFRRPT--TH-RMPNWVKWF-----FLEIL--     329
VDD84963.1          GKYLLFTMFMVSISIGVTV-IS---LNLHFRKPT--TH-RMPRWIKWL-----FLQRL--     368
VDK42196.1          GKYLLFTMVMVTLSVIVTV-IS---LNLHFRTPT--TH-RMPAWVKWL-----FLKFL--     295
KFD58288.1          GKYLLFTMVLVTLSIAVTV-FT---LNVHYRLPA--TH-RFSKWIRTF-----FLEFL--     373
OUC49089.1          GKYLLFTMVMITLSIAVTV-FT---LNVHYRLPA--TH-RFSVWIRYL-----FLDFL--     370
EYC26492.1          GKYLLFTMVLVTLSVVVTV-VT---LNVHYRSPT--TH-NMPECVRHF-----FIEVL--     396
VDK27218.1          GKYLLFTMVLVTLSVIVTV-IT---LNVHFRSPA--TH-TMPEWAKVF-----FTDLL--     365
VDM17286.1          SQFLLFTFAMTFLALCITAAVI---INLNHCNPK--SHPTVPLWVQRS-----CMDWL--     275
VDL91846.1          GKYLLFTIILITSSIVSTV-LV---LSLHFRSAK--TH-VISAFTRTV-----FLGLL--     343
KAE9548540.1        GRYLVTTMVLVALSTVISV-VT---VNFRYRRGS--AY-KMSPWVRTI-----FLNCI--     361
PDM74087.1          AKYLLLTFVLNVITILVTV-II---INVYFRSPT--TH-RMPNWVRVI-----FLQFM--     334
XP_024504322.1      AKYLLLTFVLNVITILITV-II---INVYFRAPT--TH-RMPKWVRVV-----FLEFL--     337
PIO52336.1          AKYLLLTFVLNVITILVTV-II---INVYFRGPT--TH-RMPKWVRKL-----FLEWM--     333
TKR73865.1          AKYLLLTFVLNVITILVTV-II---INVYFRSPT--TH-RMPNWARAT-----FLVIL--     338
RLU23395.1          AKYLLFTFIMNTVSILVTV-II---INWNFRGPR--TH-RMPQLIRKI-----FLKYL--     347
XP_002427906.1      AKYLLFTFIMNTVSILVTV-II---INWNFRGPR--TH-RMPPWIRAV-----FLYYL--     348
TMW47392.1          AKYLLFTFIMNTVSILVTV-VI---INWNFRGPR--TH-RMPNWIRRA-----FLQYL--     348
VDD83956.1          VKFLLFTMVLIIFSTIATV-IV---LNIHTRTPD--TH-KISPWTRHI-----FIEVL--     360
VEL33078.1          GKYLLFTIILVSLSIIVTV-IV---LNLHHRASE--AQ-NMSGWLRVV-----FLGIL--     290
XP_009019088.1      GKYLLFTLMLVSSSVIITV-LT---YNIHYRSFA--TH-CLPHWVRKV-----FLYWL--     352
KAE9536378.1        GKYLLFTMLLVALCVVVTI-II---INIHYRQPS--TH-KIPSWMRTV-----FIRAL--     389
KDR23473.1          GKYLLFTMILVGLSVVITI-MV---LNVHYRKPS--TH-KMARWVRKV-----FIRRL--     338
```

```
KAF7266914.1      GKYLLFTMILVTSSIWVTV-CV---LNVHFRSPS--TH-KMSPLFRKI-----FLHIM--    371
KAF4519445.1      GKYLLFTMILVTLSICVTV-CV---LNVYFRSPS--TH-KMAPWVKSV-----FLNVM--    368
OXU25983.1        GKYLLFTMILVTLSIWITV-CV---LNVHFRSPT--TH-NMSPWVRSV-----FLNWM--    356
TGZ32403.1        GKYLLFTMILVTLSIWITV-CV---LNVHFRSPT--TH-NMSPWVRHM-----FLNWM--    366
ELT94491.1        GKYLLFTMVLVTCSIIVTV-FV---LNIHFRTPS--TH-VMSPWIREL-----FLNVL--    356
VVC39575.1        GKFVLFTMILDTLSICVTV-VV---LNVHFRSPT--TH-VMSPWVRRV-----FIHIL--    447
RVE49089.1        GKFVLFTMILDTFSICVTV-VV---LNVHFRSPQ--TH-TMAPWVRRV-----FIHVL--    351
KAB0800277.1      GQYLLFTMLLCTLSVVTTI-AV---LNVNFRSPV--TH-KLAPWVRYF-----FIGIL--    348
TDG52197.1        GKYLLFTMMLVTLSVVVTI-AV---LNVNFRSPV--TH-RMAPWVQRV-----FIKIL--    334
TMW48669.1        GKYLLFTMMLVTLSVVVTI-AV---LNVNLGNHY--TF----------------------    335
XP_025896085.1    GVYFVVCMALLVLSLMETV-LI---VRLVHQQDL--QP-HVPAWVRRW-----VLECA--    353
KAF2977017.1      GIYFVVCMALLVISLTETI-LI---VCLVHKQDL--QP-HVPEWLKHL-----LLERA--    157
XP_009321837.1    GIYFVVCMALLVISLMETI-LI---VRLVHKQDL--QP-HVPEWVKRL-----LLEQA--    332
XP_009979985.1    GIYFVVCMALLVISLTETI-LI---VRVVHKQDL--QP-RVPGWVKRL-----LLQRA--    305
XP_028942374.1    GIYFVVCMALLVISLTETI-LI---VRLVHKQDL--QP-HVPDWVKRL-----VLERA--    329
PKK19633.1        GIYFVVCMALLVISLTETI-LI---VRLVHKQDL--QP-HVPQWVKHL-----LLERA--    395
XP_009894240.1    GIYFVVCMALLVISLTETI-LI---VRLVHKQDL--QP-HVPEWVKRL-----LLERA--    448
XP_032820219.1    AVYFVVCMALLVISLMETI-FI---VRLVHKQDV--QS-HVPPWLKKW-----LLSRG--    381
XP_029429480.1    GVYFAVCLALLVLSLTESI-VI---VQLVHKQNL--QP-HVPRWMKRL-----VLEKL--    384
XP_030077572.1    GVYFAVCMALLVTSLTETI-LV---VRLVHNQHL--KS-HVPHWMKYL-----VLEKI--    331
XP_033774596.1    GVYFAVCMALLVISLTETI-LV---VRLVHNQHL--KS-QVPHWMNYL-----VLEKI--    362
ETE72600.1        -IYFVVCMALLVISLTETI-LI---VRLVHKQDL--QP-HVPDWVKRW-----VLERA--    348
XP_018082638.1    GVYFVVCMALLVISLTETI-LI---VRLVHKQDL--QP-HVPEWLRRL-----VLEKI--    564
XP_032905410.1    GVYFVVCMALLVISLTETI-FI---VRLIHKQDL--QP-QVPEWLKHL-----VLEKV--    351
XP_020773490.1    GVYFVVCMALLVISLTETV-LI---VRLVHKQDL--QP-PVPHWLKYL-----VLEKA--    357
XP_033833634.1    GVYFVVCMALLVISLTETV-LI---VRLVHKQDL--QP-PVPHWLKYL-----VLEKA--    383
XP_030215795.1    GVYFVVCMALLVISLAETI-II---VRLVHKQDL--QR-PAPDWLKRL-----VLEKA--    356
XP_030602980.1    GVYFVVCMALLVISLTETI-LI---VRLVHKQDL--QP-PVPHWLKYL-----VLERA--    362
XP_004573543.1    GVYFVVCMALLVISLTETV-LI---VRLVHKQDL--QP-PVPQWLKYL-----VLERA--    356
XP_005916159.1    GVYFVVCMALLVISLTETV-LI---VRLVHKQDL--QP-PVPQWLKYL-----VLERA--    356
XP_013132089.1    GVYFVVCMALLVISLTETV-LI---VRLVHKQDL--QP-PVPQWLKYL-----VLERA--    356
XP_031603488.1    GVYFVVCMALLVISLTETV-LI---VRLVHKQDL--QP-PVPQWLKYL-----VLERA--    362
XP_005725536.1    GVYFVVCMALLVISLTETV-LI---VRLVHKQDL--QP-PVPQWLKYL-----VLERA--    362
XP_026038075.1    GVYFVVCMALLVISLTETV-LI---VRLVHKQDL--QP-PVPQWLKYL-----VLERA--    362
CAF96649.1        GVYFVVCMALLVISLTETV-LI---VRLVHKQDL--RD-AGPRL----------------    338
XP_023818570.1    GVYFVVCMALLVISLTETV-LI---VRLVHKQDL--QP-PVPHWVKYL-----VLERA--    352
RVE64289.1        GVYFVVCMALLVISLTETV-LI---VRLVHKQDL--QP-PVPHWVKYL-----VLERA--    387
XP_024121971.1    GVYFVVCMALLVISLTETV-LI---VRLVHKQDL--QP-PVPHWVKYL-----VLERA--    358
XP_015817559.1    GVYFVVCMALLVISLTETV-LI---VRLVHKQDL--QP-PVPHWVKYL-----VLERA--    342
XP_015225670.1    ------------------------------------------------------------    295
XP_012722063.1    GVYFVVCMALLVISLTETV-LI---VRLVHKQDL--QP-PVPHWVKYL-----VLERA--    356
XP_032431307.1    GVYFVVCMALLVISLTETV-LI---VRLVHKQDL--QP-PVPHWVKYL-----VLERA--    356
XP_014328329.1    GVYFVVCMALLVISLTETV-LI---VRLVHKQDL--QP-PVPHWVKYL-----VLERA--    356
XP_027886578.1    GVYFVVCMALLVISLTETV-LI---VRLVHKQDL--QP-PVPHWVKYL-----VLERA--    356
XP_008426791.1    GVYFVVCMALLVISLTETV-LI---VRLVHKQDL--QP-PVPHWVKYL-----VLERA--    356
XP_014838686.1    GVYFVVCMALLVISLTETV-LI---VRLVHKQDL--QP-PVPHWVKYL-----ILERA--    356
XP_014885967.1    GVYFVVCMALLVISLTETV-LI---VRLVHKQDL--QP-PVPHWVKYL-----ILERA--    356
XP_033954312.1    GVYFVVCMALLVISLTETV-LI---VRLVHKQDL--QP-PVPPWLKYL-----ILERA--    356
XP_034089244.1    GVYFVVCMALLVISLTETV-LI---VRLVHKQDL--QP-PVPPWLKYL-----ILERA--    356
XP_010780064.1    AGHK--------------------------------------------------------    330
XP_033970261.1    GVYFVVCMALLVISLTETV-LI---VRLVHKQDL--QP-PVPPWLKYL-----ILELA--    372
XP_029375575.1    GVYFVVCMALLVISLTETV-LI---VRLVHKQDL--QP-PVPPWVKHL-----VLERA--    393
KAF0023022.1      GVYFVVCMALLVISLTETV-LI---VRLVHKQDL--QP-PVPHWVKYL-----VLERA--    389
XP_019952614.1    GVYFVVCMALLVISLTETV-LI---VRLVHKQDL--QP-PVPHWVKYL-----VLERA--    345
XP_034463117.1    GVYFVVCMALLVISLTETV-LI---VRLVHKQDL--QP-PVPHWLKYL-----VLERA--    356
XP_029924656.1    GVYFVVCMALLVISLTETV-LI---VRLVHKQDM--QP-PVPHWVKHL-----VLERA--    357
XP_028323228.1    GVYFAVCMALLVISLTETV-LI---VRLVHKQDL--QM-PVPHWIKYL-----VLERA--    356
TNM84480.1        R-----------------------------------------------------------    311
XP_029703788.1    GVYFVVCMALLVISLTETV-LI---VRLVHKQDL--QT-PVPDWVKYV-----VLERA--    356
XP_030010368.1    GVYFVVCMALLVISLTETV-LI---VRLVHKQDL--QP-PVPRWVKHL-----ILERA--    359
XP_019725056.1    GVYFVVCMALLVISLTETV-LI---VRLVHKQDL--QP-PVPHWLRYL-----ILERA--    357
XP_034034934.1    GVYFVVCMALLVISLTETV-LI---VRLVHKQDL--QR-PVPQWVKYL-----VLERA--    345
XP_029956880.1    GVYFVVCMALLVISLTETV-LI---VRLVHKQDL--QP-PVPPWVKYL-----VLERA--    356
XP_008331307.3    GVYFVVCMALLVISLTETV-LI---VRLVHKQDL--QP-PVPHWVKYL-----VLERA--    389
XP_031724845.1    GVYFVVCMALLVISLTETV-LI---VRLVHKQDL--QP-PVPHWVKYL-----VLERA--    357
XP_013859395.1    GVYFVVCMALLVISLTETV-LI---VRLVHKQDL--QP-PVPHWVKYL-----ILERA--    356
XP_017275335.1    GVYFVVCMALLVISLTETV-LI---VRLVHKQDL--QP-PVPHWVKYL-----ILERA--    356
XP_020496197.1    GVYFVVCMALLVISLTETV-LI---VRLVHKQDL--QP-PVPHWVKYL-----VLERA--    356
XP_029030400.1    GVYFVVCMALLVISLTETV-LI---VRLVHKQDL--QT-PVPHWVKYL-----VLERA--    356
XP_022060538.1    GVYFVVCMALLVISLTETV-LI---VRLVHKQDL--QP-PVPHWVKYL-----VLERA--    357
XP_023150584.1    GVYFVVCMALLVISLTETV-LI---VRLVHKQDL--QP-PVPHWVKYL-----VLERA--    357
XP_029303892.1    GVYFVVCMALLVISLTETV-LI---VRLVHKQDL--QP-PVPDWVKYL-----VLERA--    380
XP_020454172.1    GVYFVVCMALLVISLTETV-LI---VRLVHKQDL--QP-PVPHWVKYL-----VLERA--    355
TKS83244.1        GVYFVVCMALLVISLTETV-LI---VRLVHKQDL--QT-PVPHWVKYL-----VLERA--    343
XP_022616583.1    GVYFVVCMALLVISLTETV-LI---VRLVHKQDL--QP-PVPHWVKYL-----VLERA--    389
XP_030293172.1    GVYFVVCMALLVISLTETV-LI---VRLVHKQDL--QT-PVPHWVKYV-----VLERA--    356
XP_027129539.1    GVYFVVCMALLVISLTETV-LI---VRLVHKQDL--QT-PVPHWVKYL-----VLERA--    356
```

XP_034539538.1	GVYFVVCMALLVISLTETV-LI---VRLVHKQDL--QP-PVPQWVKYL-----VLERA--	356
XP_008277330.1	GVYFVVCMALLVISLTETV-LI---VRLVHKQDL--QP-PVPHWVKYL-----VLERA--	356
XP_028276940.1	GVYFVVCMALLVISLTETV-LI---VRLVHKQDL--QS-PVPHWVKYL-----VLERA--	356
XP_023263433.1	GVYFVVCMALLVISLTETV-LI---VRLVHKQDL--QP-PVPHWVKYL-----VLERA--	356
XP_034406336.1	GVYFVVCMALLVISLTETV-LI---VRLVHKQDL--QP-PVPHWVKYL-----VLERA--	356
XP_026184460.1	GVYFVVCMALLVISLTETV-LI---VRLVHKQDL--QT-PVPHWVKYL-----VLERA--	356
KAF1378228.1	GVYFVVCMALLVISLTETV-LI---VRLVHKQDL--QP-PVPHWVKYL-----VLERA--	341
XP_028450365.1	GVYFVVCMALLVISLTETV-LI---VRLVHKQDL--QP-PVPHWVKYL-----VLERA--	356
XP_031163851.1	GVYFVVCMALLVISLTETV-LI---VRLVHKQDL--QP-PVPHWLKYL-----VLERA--	356
XP_032389084.1	GVYFVVCMALLVISLTETV-LI---VRLVHKQDL--QP-PVPHWVKYL-----VLERA--	356
XP_033494682.1	GVYFVVCMALLVISLTETV-LI---VRLVHKQDL--QP-PVPHWVRYL-----VLERA--	356
XP_018544782.1	GVYFVVCMALLVISLTETV-LI---VRLVHKQDL--QP-PVPHWVKYL-----VLERA--	356
XP_026228189.1	GVYFVVCMALLVISLTETV-LI---VRLVHKQDL--QT-PVPHWVKYL-----VLERA--	356
XP_028976606.1	GVYFVVCMALLVISLTETV-LI---VRLVHKQDL--QS-HVPQWLRHL-----VLERA--	392
CAB1352378.1	-VYFVVCMALLVISLTETV-LI---VRLVHKQDL--QP-HVPQWLKHL-----VLERA--	320
XP_023866849.1	GVYFVVCMALLVISLTETV-LI---VRLVHKQDL--QP-HVPQWLKHL-----VLERA--	356
XP_013992832.1	GVYFVVCMALLVISLTETV-LI---VRLVHKQDL--QP-HVPQWLKHL-----VLERA--	356
XP_029546688.1	GVYFVVCMALLVISLTETV-LI---VRLVHKQDL--QP-HVPQWLKHL-----VLERA--	356
XP_021481546.1	GVYFVVCMALLVISLTETV-LI---VRLVHKQDL--QP-HVPQWLKHL-----VLERA--	356
XP_020339889.1	GVYFVVCMALLVISLTETV-LI---VRLVHKQDL--QP-HVPQWLKHL-----VLERA--	356
XP_029481972.1	GVYFVVCMALLVISLTETV-LI---VRLVHKQDL--QP-HVPQWLKHL-----VLERA--	356
KPP68743.1	GVYFVVCMALLVISLTETV-LI---VRLVHKQDL--QP-PVPNWLKRL-----VLEKV--	333
XP_023669383.1	GVYFVVCMALLVISLTETV-LI---VRLVHKQDL--QP-PVPDWVKRL-----VLEKA--	355
KAA0720096.1	GVYFVVCMALLVISLTETV-LI---VRLVHKQDL--QS-HVPQWVKHL-----VLEKA--	261
XP_009293684.1	GVYFVVCMALLVISLTESV-LI---VRLVHKQDL--QS-RVPQWVKHL-----VLEKA--	355
XP_026090784.1	GVYFVVCMALLVISLTESV-LI---VRLVHKQDL--QS-RVPQWVKHL-----VLEKI--	355
XP_018918715.1	GVYFVVCMALLVISLTESV-LI---VRLVHKQDL--QS-RVPQWVKHL-----VLEKA--	355
KAF4098549.1	GVYFVVCMALLVISLTESV-LI---VRLVHKQDL--QS-RVPQWVKHL-----VLEKA--	355
XP_016084173.1	GVYFVVCMALLVISLTESV-LI---VRLVHKQDL--QS-RVPQWVKHL-----VLEKA--	355
XP_016332759.1	GVYFVVCMALLVISLTESV-LI---VRLVHKQDL--QS-RVPQWVKHL-----VLEKA--	355
XP_016398039.1	GVYFVVCMALLVISLTESV-LI---VRLVHKQDL--QS-RVPQWVKHL-----VLEKA--	355
XP_026989970.1	GVYFIVCMALLVISLTETV-LI---VRLVHKQDL--QT-HVPQWVKYL-----VLEKA--	355
TSQ12698.1	GVYFIVCMALLVISLTETV-LI---VRLVHKQDL--QT-HVPQWVKYL-----LLEKA--	355
XP_017347546.1	GVYFIVCMALLVISLTETV-LI---VRLVHKQDL--QT-HVPQWVKYL-----VLEKA--	350
XP_026794616.2	GVYFIVCMALLVISLTETV-LI---VRLVHKQDL--QT-HVPQWVKYL-----VLEKA--	355
XP_026861889.1	GVYFVVCMALLVISLTETV-LI---VRLVHKQDL--QS-HVPQWVKHL-----VLDKA--	355
XP_017575347.1	GVYFVVCMALLVISLTETV-LI---VRLVHKQDL--QP-HVPQWVKHL-----VLEKA--	355
XP_022531596.1	GVYFVVCMALLVISLTETV-LI---VRLVHKQDL--QS-HVPQWVKNL-----VLEKA--	355
XP_012691019.2	GVYFVVCMALLVISLTETV-LI---VRLVHQQDL--QP-RVPNWLRHL-----VLERA--	338
XP_028839723.1	GVYFVVCMALLVISLTETV-LI---VRLVHKQDL--QS-HVPQWVKYL-----VLERA--	355
XP_030638860.1	GVYFVVCMALLVISLTETV-LI---VRLVHKQDL--RP-HVPQWVRHL-----VLEKA--	337
XP_007882964.1	GVYFIVCMALLVISLTETI-LI---VRLVHKQDL--QP-HVPDWLRHL-----VLVRA--	333
GCB70244.1	GVYFVVCMALLVISLTETI-LI---VRLVHKQDL--QP-HVPEWVKRL-----VLEKA--	334
GCC26242.1	GVYFVVCMALLVISLTETI-LI---VRLVHKQDL--QP-HVPEWVKSL-----ILDKA--	355
XP_020387034.1	GVYFVVCMALLVISLTETI-LI---VQLVHKQDL--RS-HAPEWVKSL-----ILDKA--	346
XP_028931940.1	GVYFVVCMALLVISLTETI-LI---VRLVHRQEL--QR-PVPPWLRRL-----VLERA--	361
XP_031762490.1	GVYFVVCMALLVISLTETI-LI---VRLVHKQDL--QT-HVPEWLKRL-----VLEKI--	349
PIO32240.1	GVYFVVCMALLVISLTETI-LI---VRLVHKQDL--QP-HVPEWVKRL-----VLEKI--	147
XP_018425466.1	GVYFVVCMALLVISLTETI-LI---VRLVHKQDL--QP-HVPEWVKRL-----VLEKI--	350
XP_006642351.1	GVYFVVCMALLVISLTETI-LI---VRLVHKQDL--QS-HVPEWVKHL-----VLEKA--	372
XP_028665372.1	GVYFVVCMALLVISLMETI-LI---VRLVHKQDL--QP-HVPEWLRHL-----ILERA--	355
GCF49814.1	GVYFVVCMALLVISLTETI-LI---VRLVHKQDL--QP-HVPEWVKRW-----VLGRA--	336
XP_015277816.1	GVYFVVCMALLVISLTETI-LI---VRLVHKQDL--QP-HVPDWVKRW-----VLERA--	359
XP_005987340.2	GVYFVVCMALLVISLTETI-LI---VRLVHKQDL--QP-HVPDWVKYL-----VLEKA--	339
XP_019339534.1	GIYFIVCMALLVLSLTETI-LI---VRLVHKQDL--QP-HVPDWVKWL-----VLDRA--	356
XP_025063414.1	GIYFIVCMALLVLSLTETI-LI---VRLVHKQDL--QP-HVPDWVKWL-----VLDRA--	356
XP_019367488.1	GIYFVVCMALLVLSLTETI-LI---VGLVHKQDL--QS-HVPDWVKWL-----VLDRA--	356
XP_019412078.1	GIYFVVCMALLVLSLTETI-LI---VRLVHKQDL--QS-HVPDWVKWL-----VLDRA--	356
XP_014434314.1	GVYFVVCMALLVISLTETI-LI---VRLVHKQDL--QP-HVPDWVKRL-----VLERA--	356
XP_007060556.1	GVYFVVCMALLVISLTETI-LI---VRLVHKQDL--QP-HVPDWVKHL-----VLERA--	350
XP_024072175.1	GVYFVVCMALLVISLTETI-LI---VRLVHKQDL--QP-HVPDWVKHL-----VLERA--	335
XP_008170567.1	GVYFVVCMALLVISLTETI-LI---VRLVHKQDL--QP-HVPDWVKHL-----VLERA--	356
XP_034610543.1	GVYFVVCMALLVISLTETI-LI---VRLVHKQDL--QP-HVPDWVKHL-----VLERA--	356
XP_030394127.1	GVYFVVCMALLVISLTETI-LI---VRLVHKQDL--QP-HVPNWVKHL-----VLERA--	356
XP_032651960.1	SVYFVVCMALLVISLTETI-LI---VRLVHKQDL--QP-HVPDWVKHL-----VLERA--	356
XP_025020083.1	GIYFVVCMALLVISLTETI-LI---VRLVHKQDL--QP-HVPDWAKRW-----VLERA--	356
XP_026536833.1	GIYFVVCMALLVISLTETI-LI---VRLVHKQDL--QP-HVPDWVKRW-----VLERA--	356
XP_026564787.1	GIYFVVCMALLVISLTETI-LI---VRLVHKQDL--QP-HVPDWVKRW-----VLERA--	356
XP_032084675.1	GIYFVVCMALLVISLTETI-LI---VRLVHKQDL--QP-HVPDWVKRW-----VLERA--	341
XP_029139341.1	GIYFVVCMALLVISLTETI-LI---VRLVHKQDL--QP-HVPDWVKRW-----VLERA--	356
XP_034281006.1	GIYFVVCMALLVISLTETI-LI---VRLVHKQDL--QP-HVPDWVKRW-----VLERA--	356
XP_020649062.1	GVYFVVCMALLVISLTETI-LI---VRLVHKQDL--QP-HVPEWVKRW-----VLERA--	356
XP_008117087.1	GVYFVVCMALLVISLTETI-LI---VRLVHKQDL--QP-HVPEWVKRW-----VLERA--	356
XP_028564324.1	GVYFVVCMALLVISLTETI-LI---VRLVHKQDL--QP-HVPDWVKRW-----VLERA--	362
XP_033028155.1	GVYFVVCMALLVISLTETI-LI---VRLVHKQDL--QP-HVPDWVKRW-----VLERA--	361
XP_025963249.1	GIYFVVCMALLVISLTETT-LI---VHLVHKQDL--QP-HVPDWVKHW-----LLERA--	343
XP_009668348.1	GIYFVVCMALLVISLTETI-LI---VCLVHKQDL--QP-HVPDWVKHL-----LLERA--	355

XP_013798935.1	GVYFVVCMALLVISLTETT-LI---VHLVHKQDL--QP-HVPDWVKHL-----LLERA--	339
XP_025913685.1	GVYFVVCMALLVISLTETT-LI---VHLVHKQDL--QP-HVPDWVKHL-----LLERA--	364
XP_013042552.1	GIYFVVCMALLVISLTETI-LI---VRLIHNQDL--QP-HVPNWVKHL-----LLERA--	362
XP_005030458.2	GIYFVVCMALLVISLTETI-LI---VRLIHNQDL--QP-HVPSWVKHL-----LLERA--	355
XP_032057953.1	GIYFVVCMALLVISLTETI-LI---VRLIHNQDL--QP-HVPSWVKHL-----LLERA--	338
XP_021232050.1	GIYFVVCMALLVISLTETI-FI---VRLVHKQDL--QP-HVPSWVKHL-----LLERA--	357
OXB62403.1	GIYFVVCMALLVISLTETI-FI---VRLVHKQDL--QP-HVPNWVKHL-----LLERA--	356
OXB81319.1	GIYFVVCMALLVISLTETI-FI---VRLVHKQDL--QP-HVPNWVKHL-----LLERA--	338
XP_010722007.1	GIYFVVCMALLVISLTETI-FI---VRLVHKQDL--QP-HVPSWVKHL-----LLERA--	357
XP_015739349.1	GIYFVVCMALLVISLTETI-FI---VRLVHKQDL--QP-HVPSWVKHL-----LLERA--	357
XP_031455498.1	GIYFVVCMALLVISLTETI-FI---VRLVHKQDL--QP-HVPSWVKHL-----LLERA--	358
POI27435.1	GIYFVVCMALLVISLTETI-FI---VRLVHKQDL--QP-HVPSWVKHL-----LLERA--	333
XP_004948120.1	GIYFVVCMALLVISLTETI-FI---VRLVHKQDL--QP-HVPSWVKHL-----LLERA--	358
XP_032851190.1	GIYFVVCMALLVISLTETI-LI---VCLVHKQDL--QP-HVPAWVKRL-----LLERA--	353
XP_010007255.1	GIYFVVCMALLVISLTETI-LI---VRLVHKQDL--QP-QVPHWVKHL-----LLERA--	355
XP_030320702.1	GIYFVVCMALLVISLTETI-LI---VRLVHKQDL--QP-HVPHWVKHL-----LLEQA--	326
XP_010191940.1	GTYFVVCMALLVISLTETI-LI---VRLVHKQDL--QP-HVPEWVKRL-----LLERA--	353
XP_027737112.1	GIYFVVCMALLVISLTETI-LI---VRLVHKQDL--QP-HVPEWVKHL-----LLERA--	351
XP_027555032.1	GTYFVVCMALLVISLTETI-LI---VRLVHKQDL--QP-HVPEWVKHL-----LLERA--	356
XP_032565370.1	GTYFVVCMALLVISLTETI-LI---VRLVHKQDL--QP-HVPEWVKHL-----LLERA--	402
XP_027511217.1	GTYFVVCMALLVISLTETI-LI---VRLVHKQDL--QP-HVPEWVKHL-----LLERA--	501
XP_027593499.1	GTYFVVCMALLVISLTETI-LI---VRLVHKQDL--QP-HVPEWVKHL-----LLERA--	452
XP_017664924.1	GTYFVVCMALLVISLTETI-LI---VRLVHKQDL--QP-HVPEWVKHL-----LLERA--	353
XP_029817938.1	GTYFVVCMALLVISLTETI-LI---VRLVHKQDL--QP-HVPEWVKHL-----LLERA--	335
XP_005058641.1	GIYFVVCMALLVISLTETI-LI---VRLVHKQDL--QP-HVPEWLKHL-----LLEKA--	386
XP_021385804.1	GIYFVVCMALLVISLTETI-LI---VRLVHKQDL--QP-HVPKWVKHL-----LLERA--	355
XP_030146687.2	GIYFVVCMALLVISLTETI-LI---VRLVHKQDL--QP-HVPEWVKHL-----LLERA--	400
KAF4796420.1	GIYFVVCMALLVISLTETI-LI---VRLVHKQDL--QP-HVPEWVKHL-----LLEKA--	355
XP_032937581.1	GIYFVVCMALLVISLTETI-LI---VRLVHKQDL--QP-HVPEWVKHL-----LLEKA--	402
XP_031989659.1	GIYFVVCMALLVISLTETI-LI---VRLVHKQDL--QP-HVPEWVKHL-----LLERA--	356
XP_010402086.1	GIYFVVCMALLVISLTETI-LI---VRLVHKQDL--QP-HVPEWVKHL-----LLERA--	355
XP_017594069.1	GIYFVVCMALLVISLTETI-LI---VRLVHKQDL--QP-HVPEWVKHL-----LLERA--	326
XP_014115268.1	GIYFVVCMALLVISLTETI-LI---VRLVHKQDL--QP-HVPEWVKHL-----LLERA--	380
XP_023797108.1	GIYFVVCMALLVISLTETI-LI---VRLVHKQDL--QP-HVPEWVKHL-----LLERA--	326
XP_033375614.1	GIYFVVCMALLVISLTETI-LI---VRLVHKQDL--QP-HVPEWVKHL-----LLERA--	326
XP_014740121.1	GIYFVVCMALLVISLTETI-LI---VRLVHKQDL--QP-HVPEWVKHL-----LLERA--	500
RLV83430.1	GIYFVVCMALLVISLTETI-LI---VRLVHKQDL--QP-HVPEWVKHL-----LLEKA--	346
XP_009096098.2	GIYFVVCMALLVISLTETI-LI---VCLVHKQDL--QP-HVPGWVKHL-----LLERA--	461
TRZ15870.1	GIYFVVCMALLVISLTETI-LI---VRLVHKQDL--QP-HVPEWMKHL-----LLERA--	356
RMB91935.1	GIYFVVCMALLVISLTETI-LI---VRLVHKQDL--QP-HVPAWVKHL-----LLERA--	326
XP_030820843.1	GIYFVVCMALLVISLTETI-LI---VCLVHKQDL--QP-HVPEWLKHL-----LLERA--	402
XP_014165179.1	GIYFVVCMALLVISLTETI-LI---VCLVHKQDL--QP-HVPEWLKHL-----LLERA--	404
XP_026653582.1	GIYFVVCMALLVISLTETI-LI---VCLVHKQDL--QP-HVPEWLKHL-----LLERA--	286
PKU35975.1	GIYFVVCMALLVISLTETI-LI---VRLVHKQDL--QP-HVPEWVKRL-----LLERA--	358
XP_014805072.1	GIYFVVCMALLVISLTETI-LI---VRLVHRQDL--QP-HVPEWVKHL-----LLERA--	352
XP_009818330.1	GIYFVVCMALLVISLTETI-LI---VRLVHKQDL--QP-HVPEWVKRL-----LLERA--	123
OPJ68307.1	GIYFVVCMALLVISLTETI-LI---VRLVHKQDL--QP-HVPQWVKHL-----LLERA--	355
XP_008936289.1	GIYFVVCMALLVISLTETI-LI---VRLVHKQDL--QP-HVPKWVKRL-----LLERA--	356
XP_010287046.1	GVYFVVCMALLVISLTETI-LI---VRLVHKQDL--QP-HVPDWVKHL-----LLERA--	286
XP_005240140.2	GIYFVVCMALLVISLMETI-LI---VCLVHKQDL--QP-HIPGWVRRL-----LLEQA--	415
XP_005437752.2	GIYFVVCMALLVISLMETI-LI---VCLVHKQDL--QP-HVPGWVRRL-----LLEQA--	415
KFV74811.1	GIYFVVCMALLVISLTETI-LI---VRLVHKQDL--QP-HVPPWVKHL-----LLEQA--	320
XP_010018389.1	GIYFVVCMALLVISLTETI-LI---VRLVHKQDL--QP-HVPAWVKHL-----LLERA--	363
KQK78711.1	GIYFVVCMALLVISLTETI-LI---VRLVHKQDL--QP-HVPAWVKHL-----LLERA--	355
XP_009570162.1	GTYFVVCMALLVISLTETI-LI---VRLVHKQDL--QP-HVPDWVKRL-----LLERA--	354
KFP11268.1	GIYFVVCMALLVISLTETI-LI---VRLVHKQDL--QP-HVPDWVKRL-----LLERA--	318
KFQ98910.1	GIYFVVCMALLVISLTETI-LI---VRLVHKQDL--QP-HVPDWVKRL-----LLERA--	337
XP_012985202.3	GIYFVVCMALLVISLTETI-LI---VRLVHKQDL--QP-HVPAWVKHL-----LLERA--	355
XP_010573388.1	GIYFVVCMALLVISLTETI-LI---VRLVHKQDL--QP-HVPEWVKRL-----LLERA--	354
XP_029879496.1	GIYFVVCMALLVISLTETI-LI---VRLVHKQDL--QP-HVPEWVKRL-----LLERA--	326
XP_030361086.1	GIYFVVCMALLVISLMETI-LI---VRLVHKQDL--QP-HVPAWVKHL-----LLERA--	407
KFM00668.1	GIYFVVCMALLVISLMETI-LI---VRLVHKQDL--QP-HVPEWVKRL-----LLEQA--	338
KAF1479074.1	GIYFVVCMALLVISLMETI-LI---VRLVHKQDL--QP-HVPEWVKRL-----LLEQA--	318
KAF1651161.1	GIYFVVCMALLVISLMETI-LI---VRLVHKQDL--QP-HVPEWVKRL-----LLEQA--	319
KAF1673648.1	GIYFVVCMALLVISLMETI-LI---VRLVHKQDL--QP-HVPEWVKRL-----LLEQA--	319
KAF1493319.1	GTYFVVCMALLVISLMETI-LI---VRLVHKQDL--QP-HVPEWVKRL-----LLEQA--	319
KAF1584157.1	GTYFVVCMALLVISLMETI-LI---VRLVHKQDL--QP-HVPEWVKRL-----LLEQA--	319
KAF1571723.1	GTYFVVCMALLVISLMETI-LI---VRLVHKQDL--QP-HVPEWVKRL-----LLEQA--	319
KAF1533169.1	GTYFVVCMALLVISLMETI-LI---VRLVHKQDL--QP-HVPEWVKRL-----LLEQA--	319
KAF1638955.1	GTYFVVCMALLVISLMETI-LI---VRLVHKQDL--QP-HVPEWVKRL-----LLEQA--	319
KAF1549972.1	GTYFVVCMALLVISLMETI-LI---VRLVHKQDL--QP-HVPEWVKRL-----LLEQA--	319
KAF1606914.1	GTYFVVCMALLVISLMETI-LI---VRLVHKQDL--QP-HVPEWVKRL-----LLEQA--	319
KAF1510957.1	GTYFVVCMALLVISLMETI-LI---VRLVHKQDL--QP-HVPEWVKRL-----LLEQA--	319
KAF1498899.1	GTYFVVCMALLVISLMETI-LI---VRLVHKQDL--QP-HVPEWVKRL-----LLEQA--	319
KAF1411525.1	GTYFVVCMALLVISLMETI-LI---VRLVHKQDL--QP-HVPEWVKRL-----LLEQA--	319
KAF1429205.1	GTYFVVCMALLVISLMETI-LI---VRLVHKQDL--QP-HVPEWVKRL-----LLEQA--	319
XP_005334318.1	GVYFVVCMALLVISLAETI-LI---VRLVHKQDL--QQ-PVPAWLQHL-----VLERI--	345

```
XP_014395552.1      GVYFVVCMALLVISLAETI-LI---VRLVHKQDL--QQ-PVPAWLRRL-----VLERI--      345
KAB0404854.1        GVYFIVCMALLVISLAETI-LI---VRLVHKQDL--QQ-PVPAWLRRL-----VLERV--      341
XP_007494828.1      GVYFVVCMALLVISLTETI-FI---VRLVHRQEL--QP-PVPAWLRHL-----VLERA--      356
XP_003764254.1      GVYFVVCMALLVISLTETI-LI---VRLVHRQEL--QS-PVPAWLRHL-----VLDRA--      356
XP_020845489.1      GVYFVVCMALLVISLTETI-LI---VRLVHRQEL--QP-PVPAWLRHL-----VLDRA--      356
XP_027703160.1      GIYFVVCMALLVISLTETI-LI---VRLVHRQEL--QP-PVPAWLRHL-----VLDRA--      356
XP_017523929.1      GVYFVVCMALLVLSLAETI-LI---VRLVHKQEL--QR-PVPAWLRRL-----APDRL--      353
XP_008688428.1      GVYFVVCMALLVMSLAETI-FI---VRLVHKQDL--QQ-PVPAWLRRL-----VLERG--      301
XP_029812166.1      GVYFIVCMALLVLSLAETI-LI---VRLVHKQDL--QQ-PVPAWLRHL-----VLEWA--      370
XP_025749781.1      GVYFVVCMALLVMSLAETI-FI---VRLVHKQDL--QQ-PVPAWLRRL-----VLGQV--      356
XP_004416432.1      GVYFVVCMALLVMSLAETI-FI---VRLVHKQDL--QQ-PVPAWLRRL-----VLGRV--      356
XP_027436262.1      GVYFVVCMALLVMSLAETI-FI---VRLVHKQDL--QQ-PVPAWLRRL-----VLGQV--      356
XP_032284025.1      GVYFVVCMALLVISLAETI-FI---VRLVHKQDL--QQ-PVPAWLRRL-----LLGRV--      356
XP_006735421.1      GVYFVVCMALLVMSLAETI-FI---VRLVHKQDL--QQ-PVPAWLRRL-----ALGRV--      356
XP_021552166.1      GVYFVVCMALLVMSLAETI-FI---VRLVHKQDL--QQ-PVPAWLRRL-----VLGRV--      356
XP_026361066.1      GVYFVVCMALLVMSLAETI-FI---VRLVHKQDL--QQ-PVPAWLRRL-----VLERG--      356
XP_034523598.1      GVYFVVCMALLVMSLAETI-FI---VRLVHKQDL--QQ-PVPAWLRRL-----VLERG--      356
NP_001297113.1      GVYFVVCMALLVMSLAETI-FI---VRLVHKQDL--QQ-PVPAWLRHL-----VLERV--      356
XP_032215488.1      GVYFVVCMALLVMSLAETI-FI---VRLVHKQDL--QQ-PVPAWLRHL-----VLERV--      356
VCX31483.1          GVYFVVCMALLVLSLAETI-FI---VRLVHKQDL--RQ-PVPAWLRHL-----VLERV--      356
XP_022369003.1      GVYFIVCMALLVMSLAETI-FI---VRLVHKQDL--QQ-PVPAWLRHL-----VLERV--      356
XP_032694248.1      GVYFVVCMALLVMSLAETI-FI---VRLVHKQDL--QQ-PVPAWLRHL-----VLERV--      356
NP_001041584.1      GVYFVVCMALLVMSLAETI-FI---VRLVHKQDL--QQ-PVPAWLRHL-----VLERV--      356
XP_025862501.1      GVYFVVCMALLVMSLAETI-FI---VRLVHKQDL--QQ-PVPAWLRHL-----VLERV--      356
KAF0873564.1        GVYFVVCMALLVVSLAETI-LI---VRLVHKQDL--QQ-PVPAWLRHL-----VLDRV--      498
XP_025784751.1      GVYFVVCMALLVVSLAETI-LI---VRLVHKQDL--QQ-PVPAWLRHL-----VLERV--      356
XP_007075625.1      GVYFVVCMALLVVSLAETI-LI---VRLVHKQDL--QQ-PVPAWLRHL-----VLERV--      492
XP_030189489.1      GVYFVVCMALLVVSLAETI-LI---VRLVHKQDL--QQ-PVPAWLRHL-----VLERV--      356
XP_019324655.1      GVYFVVCMALLVVSLAETI-LI---VRLVHKQDL--QQ-PVPAWLRHL-----VLERV--      457
XP_023094886.1      GVYFIVCMALLVVSLAETI-LI---VRLVHKQDL--QQ-PVPAWLRHL-----VLERV--      492
XP_026892357.1      GVYFVVCMALLVVSLAETI-LI---VRLVHKQDL--QQ-PVPAWLRHL-----VLERV--      356
XP_016004457.1      GVYFVVCMALLLISLAETI-FI---VRLVHKQDL--QQ-PVPAWLRRL-----VLEKV--      351
XP_006912860.1      GIYFVVCMALLVISLAETI-LI---VRLVHKQDL--QQ-PVPAWLRRL-----ILERV--      351
XP_011363679.1      GVYFVVCMALLVISLAETI-LI---VRLVHKQDL--QQ-PVPAWLRRL-----ILERV--      351
XP_016076060.1      GVYFVVCMALLVISLAETI-LI---VRLVHKQDL--QR-PVPAWLRSL-----VLERV--      356
XP_008148371.1      GVYFVVCMALLVISLAETI-LI---VRLVHKQDL--QQ-PVPAWLRRL-----VLERI--      356
XP_006093568.1      GVYFVVCMALLVISLAETI-LI---VRLVHKQDL--QQ-PVPAWLRRL-----VLERI--      356
XP_006761601.1      GVYFVVCMALLVISLAETI-LI---VRLVHKQDL--QQ-PVPAWLRRL-----VLERI--      356
XP_024426743.1      GVYFVVCMALLVISLAETI-LI---VRLVHKQDL--QQ-PVPAWLRLL-----VLEKF--      351
XP_019520779.1      GVYFVVCMALLVISLAETI-FI---VRLVHKQDL--QQ-PVPAWLRHL-----VLEKV--      356
XP_032976539.1      GVYFVVCMALLVISLAETI-LI---VRLVHKQDL--QQ-PVPAWLRHL-----VLERV--      356
ELW64270.1          GVYFVVCMALLVISLAETI-LI---VRLVHKQDL--QQ-PVPAWLRRL-----VLEQL--      360
XP_004427367.1      GVYFVVCMALLVISLAETI-LI---VRLVHKQDL--QQ-PVPAWLRHL-----VLERV--      356
XP_008529353.1      GVYFIVCMALLVISLAETI-LI---VRLVHKQDL--QQ-PVPAWLQHL-----VLERV--      356
NP_001288165.1      GVYFIVCMALLVISLAETI-LI---VRLVHKQDL--QQ-PVPAWLQHL-----VLERV--      356
XP_014711213.1      GVYFIVCMALLVISLAETI-LI---VRLVHKQDL--QQ-PVPAWLQHL-----VLERV--      356
XP_012514621.1      GVYFVVCMALLVISLAETI-FI---VRLVHKQDL--QQ-PVPAWLRHL-----VLERI--      356
XP_008071525.1      GVYFVVCMALLVISLAETI-FI---VRLVHKQDL--QQ-PVPAWLRRL-----VLERL--      353
XP_009005082.1      GVYFVVCMALLVISLAETI-FI---VRLVHKQDL--QQ-PVPAWLRHL-----VLERI--      356
XP_010332832.1      GVYFVVCMALLVISLAETI-FI---VRLVHKQDL--QQ-PVPAWLRHL-----VLERI--      351
XP_012326058.1      GVYFVVCMALLVISLTETI-FI---VRLVHKQDL--QQ-PVPAWLRHL-----VLERI--      362
XP_017378796.1      GVYFVVCMALLVISLAETI-FI---MRLVHKQDL--QQ-PVPAWLRHL-----VLERI--      385
XP_032141374.1      GVYFVVCMALLVISLAETI-FI---MRLVHKQDL--QQ-PVPAWLRHL-----VLERI--      385
XP_032024018.1      GVYFVVCMALLVISLAETI-FI---VRLVHKQDL--QQ-PVPAWLRHL-----VLERI--      351
XP_018891497.2      SVYFVVCMALLVISLAETI-FI---VRLVHKQDL--QQ-PVPAWLRHL-----VLERI--      357
XP_030684894.1      GVYFVVCMALLVISLAETI-FI---VRLVHKQDL--QQ-PVPAWLRHL-----VLERI--      351
PNJ75904.1          GVYFVVCMALLVISLAETI-FI---VRLVHKQDL--QQ-PVPAWLRHL-----VLERI--      357
AAP35868.1          GVYFVVCMALLVISLAETI-FI---VRLVHKQDL--QQ-PVPAWLRHL-----VLERI--      357
XP_001149570.1      SVYFVVCMALLVISLAETI-FI---VRLVHKQDL--QQ-PVPAWLRHL-----VLERI--      357
XP_003805532.1      SVYFVVCMALLVISLAETI-FI---VRLVHKQDL--QQ-PVPAWLRHL-----VLERI--      357
XP_023063823.1      GVYFVVCMALLVISLAETI-FI---VRLVHKQDL--QQ-PVPAWLRHL-----VLERI--      355
XP_033060210.1      GVYFVVCMALLVISLAETI-FI---VRLVHKQDL--QQ-PVPAWLRHL-----VLERI--      355
XP_010370669.1      GVYFVVCMALLVISLAETI-FI---VRLVHKQDL--QQ-PVPAWLRHL-----VLERI--      355
XP_017738496.1      GVYFVVCMALLVISLAETI-FI---VRLVHKQDL--QQ-PVPAWLRHL-----VLERI--      355
XP_011782168.1      GVYFVVCMALLVISLAETI-FI---VRLVHKQDL--QQ-PVPAWLRHL-----VLERI--      356
EHH23440.1          GVYFVVCMALLVISLAETI-FI---VRLVHKQDL--QQ-PVPAWLRHL-----VLERI--      383
XP_003910778.3      GVYFVVCMALLVISLAETI-FI---VRLVHKQDL--QQ-PVPAWLRHL-----VLERI--      356
XP_025212649.1      GVYFVVCMALLVISLAETI-FI---VRLVHKQDL--QQ-PVPAWLRHL-----VLERI--      362
XP_008019132.1      GVYFVVCMALLVISLAETI-FI---VRLVHKQDL--QQ-PVPAWLRHL-----VLERI--      356
XP_011832421.1      GVYFVVCMALLVISLAETI-FI---VRLVHKQDL--QQ-PVPAWLRHL-----VLERI--      341
XP_011759618.1      GVYFVVCMALLVISLAETI-FI---VRLVHKQDL--QQ-PVPAWLRHL-----VLERI--      356
XP_005579749.1      GVYFVVCMALLVISLAETI-FI---VRLVHKQDL--QQ-PVPAWLRHL-----VLERI--      356
XP_011921684.1      GVYFVVCMALLVISLAETI-FI---VRLVHKQDL--QQ-PVPAWLRHL-----VLERI--      148
XP_008259391.1      GVYFVVCMALLVISLAETI-LI---VRLVHKQDL--QQ-PVPPWLRRL-----VLERI--      356
XP_012782438.1      GVYFVVCMALLVISLAETI-LI---VRLVHKQDL--QQ-PVPTWLRHL-----VLERI--      352
VTJ86076.1          GVYFVVCMALLVISLAETI-LI---VRLVHKQDL--QQ-PVPAWLQHL-----VLERI--      362
XP_015345533.1      GVYFVVCMALLVISLAETI-LI---VRLVHKQDL--QQ-PVPTWLQHL-----VLERI--      362
```

```
XP_027786432.1      GVYFVVCMALLVISLAETI-LI---VRLVHKQDL--QQ-PVPAWLQHL-----VLERI--      362
XP_026248345.1      GVYFVVCMALLVISLAETI-LI---VRLVHKQDL--QQ-PVPAWLQHL-----VLERI--      356
XP_005378209.1      GVYFVVCMALLVISLAETI-LI---VRLVHKQDL--QQ-PVPLWLRHL-----VLERI--      357
XP_004466010.1      GVYFVVCMALLVLSLAETI-LI---VRLVHKQDL--QQ-PVPAWLRRL-----VLERV--      356
XP_020035707.1      GVYFVVCMALLVISLAETI-LI---VRLVHKQDL--QK-PVPAWLRHL-----VLERI--      350
XP_006181802.1      GVYFIVCMALLVISLAETI-LI---VRLVHKQDL--QR-PVPAWLRHL-----VLERI--      356
XP_006207862.1      GVYFVVCMALLVISLAETI-LI---VRLVHKQDL--QR-PVPAWLRHL-----VLERI--      356
XP_005891968.1      GVYFVVCMALLVISLAETI-LI---VRLVHKQDL--QQ-PVPAWLRHL-----VLERV--      353
XP_010828662.1      GVYFVVCMALLVISLAETI-LI---VRLVHKQDL--QQ-PVPAWLRHL-----VLERV--      353
XP_019830126.1      GVYFVVCMALLVISLAETI-LI---VRLVHKQDL--QQ-PVPAWLRHL-----VLERV--      352
XP_017914486.1      GVYFVVCMALLVISLAETI-LI---VRLVHKQDL--QQ-PVPAWLRHL-----VLERV--      352
XP_006060266.2      GVYFVVCMALLVISLAETI-LI---VRLVHKQDL--QQ-PVPAWLRHL-----VLERV--      357
XP_020728447.1      GVYFVVCMALLVISLAETI-LI---VRLVHKQDL--QQ-PVPAWLRHL-----VLERV--      357
KAF4008892.1        GVYFVVCMALLVISLAETI-LI---VRLVHKQDL--QQ-PVPAWLRHL-----VLERV--      357
KAB0348059.1        GVYFVVCMALLVISLAETI-LI---VRLVHKQDL--QQ-PVPAWLRHL-----VLERV--      340
KAB0371113.1        GVYFVVCMALLVISLAETI-LI---VRLVHKQDL--QQ-PVPAWLRHL-----VLERV--      333
MBV96963.1          GVYFVVCMALLVISLAETI-LI---VRLVHKQDL--QQ-PVPAWLRRL-----VLERV--      356
XP_007172048.1      GVYFVVCMALLVISLAETI-LI---VRLVRKQDL--QQ-PVPAWLRRL-----VLERV--      356
XP_007452270.1      GVYFVVCMALLVISLAETI-LI---VRLVHKQNL--QQ-PVPAWLRHL-----VLERV--      356
XP_023987108.1      GVYFVVCMALLVISLAETI-LT---VRLVHKQDL--QQ-PVPAWLRHL-----VLERV--      351
XP_004273432.1      GVYFVVCMALLVISLAETI-LI---VRLVHKQNL--QQ-PVPAWLRHL-----VLERV--      351
XP_004328646.1      GVYFVVCMALLVISLAETI-LI---VRLVHKQNL--QQ-PVPAWLRHL-----VLERV--      351
XP_026937670.1      GVYFVVCMALLVISLAETI-LI---VRLVHKQNL--QQ-PVPAWLRHL-----VLERV--      351
XP_030691031.1      GVYFVVCMALLVISLAETI-LI---VRLVHKQNL--QQ-PVPAWLRHL-----VLERV--      351
XP_022414870.1      GVYFVVCMALLVISLAETI-LI---VRLVHKQNL--QQ-PVPAWLRHL-----VLERV--      356
XP_029064651.1      GVYFVVCMALLVISLAETI-FI---VRLVHKQNL--QQ-PVPAWLRHL-----VLERV--      356
XP_024607566.1      GVYFVVCMALLVVSLAETI-LI---VRLVHKQNL--QQ-PVPAWLRHL-----VLERV--      356
XP_032496907.1      GVYFVVCMALLVVSLAETI-LI---VRLVHKQNL--QQ-PVPAWLRHL-----VLERV--      356
XP_007535559.1      GVYFVVCMALLVISLAETI-LI---VRLVHKQDL--QK-PVPAWLRYL-----VLDRI--      349
XP_031299536.1      GVYFIVCMALLVISLAETI-LI---VRLVHKQDL--QR-PVPAWLRHL-----VLERI--      355
XP_004666516.1      GVYFVVCMALLVISLAETI-FI---VRLTHKQDL--QQ-PVPAWLRRL-----VLEKM--      351
XP_008830963.1      GVYFVVCMALLVISLAETI-FI---VRLVHKQDL--QR-PVPLWLQRL-----VLERI--      356
XP_005347338.1      GVYFVVCMALLVISLAETI-FI---VRLVHKQDL--QR-PVPDWLRHL-----VLRKI--      356
XP_005069548.1      GVYFVVCMALLVISLAETI-FI---VRLVHKQDL--QR-PVPAWLRHL-----VLQKM--      356
XP_027267894.1      GVYFVVCMALLVISLAETI-FI---VRLVHKQDL--QR-PVPAWLRHL-----VLQKT--      356
OBS80992.1          GVYFVVCMALLVISLAETI-FI---VRLVHKQDL--QR-PVPAWLRHL-----VLEKT--      343
XP_006979812.1      GVYFVVCMALLVISLAETI-FI---VRLVHKQDL--QR-PVPAWLRHL-----VLEKT--      362
XP_028720687.1      GVYFVVCMALLVISLAETI-FI---VRLVHKQDL--QR-PVPAWLRHL-----VLEKT--      356
XP_021489005.1      GVYFVVCMALLVISLSETI-FI---VRLVHKQDL--QR-PVPAWLRHL-----VLERV--      357
XP_031199589.1      GVYFVVCMALLVISLAETI-FI---VRLVHKQDL--QR-PVPDWLRHL-----VLDRI--      381
XP_028617944.1      GVYFVVCMALLVISLAETI-FI---VRLVHKQDL--QR-PVPDWLRHL-----VLNRI--      171
XP_034347030.1      GVYFVVCMALLVISLAETI-FI---VRLVHKQDL--QR-PVPDWLRHL-----VLDRI--      354
NP_077370.2         GVYFVVCMALLVISLAETI-FI---VQLVHKQDL--QR-PVPDWLRHL-----VLDRI--      356
XP_032766961.1      GVYFVVCMALLVISLAETI-FI---VRLVHKQDL--QR-PVPDWLRHL-----VLDRI--      356
XP_021063186.1      GVYFVVCMALLVISLAETI-FI---VRLVHKQDL--QR-PVPDWLRHL-----VLDRI--      339
NP_001093114.1      GVYFVVCMALLVISLAETI-FI---VRLVHKQDL--QR-PVPDWLRHL-----VLDRI--      356
XP_021028379.1      GVYFVVCMALLVISLAETI-FI---VRLVHKQDL--QR-PVPDWLRHL-----VLDRI--      356
XP_004689305.1      GVYFVVCMALLVLSLAETI-LI---VRLVHKQDL--QQ-PVPAWLRRL-----VLERI--      356
XP_004712871.1      GVYFVVCMALLVISLAETI-LI---VRLVHKQDL--QQ-PVPPWLRRL-----VLQRL--      356
XP_004382575.1      GVYFVVCMALLVISLAETI-LI---VRVVHKQDL--QQ-PVPVWLRRL-----VLDRI--      356
XP_010596188.1      GVYFVVCMALLVISLAETI-LI---VRVVHKQDL--QQ-PVPTWLRRF-----VLERI--      356
XP_006890855.1      GVYFVVCMALLVISLAETI-FI---VRLVHKQDL--QQ-PVPAWLRRL-----VLERI--      356
XP_006834038.1      GVYFVVCMALLVISLAETI-LI---VRLVHKQDL--QQ-PVPAWLRHL-----VLERI--      356
XP_007934716.1      GVYFVVCMALLVISLAETI-LI---VRLVHKQDL--QQ-PLPTWLRRL-----VLERI--      356
XP_012603491.1      GVYFVVCMALLVISLAETI-FI---VRLVHKQDL--QQ-PVPAWLRRL-----VQERI--      356
XP_012663606.1      GIYFVVCMALLVISLAETI-FI---VRLVHKQDL--QQ-PLPAWLRRL-----VLERI--      351
XP_028372812.1      GVYFVVCMALLVISLAETI-LI---VRLVHKQDL--QQ-PVPAWLRRL-----VLEKV--      356
XP_008589608.1      GIYFVVCMALLVISLAETI-LI---VRLVHKQDL--QQ-PVPAWLRRL-----VLERTGT      358
XP_012865077.1      GVYFVVCMALLVISLAETI-LI---VRLVHKQDL--QQ-PVPAWLRRL-----VLERG--      356
NP_001166178.1      SVYFVVCMALLVISLAETI-LI---VRLVHKQDL--QQ-PVPLWLRHL-----VLERI--      357
XP_004636553.1      SVYFVVCMALLVISLAETI-LI---VRLVHKQDL--QR-PVPPWLRHL-----VLERI--      357
XP_004856670.1      GVYFVVCMALLVISLAETI-LI---VRLVHKQDL--QQ-PVPLWLRHL-----VLERI--      357
XP_010627744.1      SVYFVVCMALLVISLVETI-FI---VRLVHKQDL--QQ-PVPLWLRHL-----VLERI--      357

NP_509270.1         CKM-------------------------------------SL-----------EEIEED-      366
VDO93178.1          ANKVGLSL------------------------------PLSLKDAL---LMDQENSQEEP      374
PAV91580.1          TRIMFLHV------------------------------PPTLRILW-------GELDDDP      385
VDK46997.1          PSFLYVSV------------------------------PPALEKLW-------SELDDDP      351
VIO86814.1          PKRFRIASSEEI------------------------KRNQDVTTRF--------------      427
VDP38785.1          GYLAMIPY----------------------------K---KMNQYS--------------      421
KOF68401.1          GNMIGPKTVTK-------------------------NTPETLAE----------------      342
CDJ96026.1          ASLMRMSI----------------------------REPDSISLLK---IAQSK------      500
EFX76216.1          AKLLFIHL----------------------------DEFQQKNQME---M----------      335
KAF7403848.1        ARIVFLNF----------------------------QEENKPGSAE---S----------      343
KAF7427032.1        ARIVFLNF----------------------------QEENKPGSTE---S----------      358
KAE9417558.1        PWFLL------------------------------MSRPGKKFIRRSL-HCS--------      352
```

```
KJH51504.1        AWLLQ------------------------------MKCPDRQNTMTTI-KKS--------   353
VDL69795.1        PWLLM------------------------------MHRPGYKASRGKM-GKD--------   333
VDO32671.1        PWILC------------------------------MSRPGYTFIGGVA-IAD--------   367
VBB32409.1        PYLLR------------------------------INRPGVNLSWK--------------   339
VDN54565.1        PYFLR------------------------------MNRPGIYLTWE--------------   358
EGT55171.1        PWILR------------------------------MKRPGHNLTYA--------------   369
RCN52111.1        PWLLR------------------------------MKRPGVKLTYA--------------   374
RMX54856.1        AQLLRV------------------------------KVARHRKQDIQE-LGD--------   375
XP_022781674.1    AQFLRV------------------------------KVPRHRKQGTQE-LDH--------   637
RNA37099.1        DISFGAKANEL---------------------KVLFNMANHMDKNR------NQRIHDNK   391
KAA0187152.1      LVEKC----------------------------RSFTRNSHTSSNN------NQSSTKET   557
GAU96593.1        AYVMC----------------------------IDTDFNPRDEEKK--------------   374
XP_009043980.1    ARFVC----------------------------MRSVADSTMSMT---------------   334
VDM43573.1        RKALC----------------------------MRLPYSGWNDSGF--------------   453
XP_003140283.1    RHFLK----------------------------VHLQYPSGKRNGL--------------   344
VDN82010.1        RHFLK----------------------------MHIHRL-SNRDGL--------------   369
VDO31501.1        RHFLK----------------------------MHIHRL-SNRDGL--------------   381
VDP19591.1        PKRSQIIEVEDISSSISDNFIENYPNTKKSQNEILIKGGGKGEQQQQE-GGGGEEEDDDN   407
EDO32053.1        ATAL------------------------------LMKK----------------------   324
TRY67230.1        PW------------------------------ILFMKRPGQKITR---------------   345
KAF7391312.1      PF------------------------------ILCMSRPGKKITK---------------   360
GFG30449.1        VLDLSDIR----------YFVNFYLV---GQEVLYFPYIKRRLRL---------------   387
KQS30083.1        AA------------------------------ALFFQFLQFR------------------   389
EDW57583.2        PW------------------------------ILRMSRPGRPLIL---------------   528
KNC22799.1        PW------------------------------ILRMHRPGRPIIL---------------   338
RZF44856.1        PC------------------------------LLRMSRPAGGGNG---------------   358
ENN76856.1        PK------------------------------CLYMHRPGDKENN---------------   364
KAF5300392.1      PW------------------------------VLRMHKPRHN------------------   365
CAB3239999.1      PW------------------------------ILRMSRPGSA------------------   352
PCG77624.1        PW------------------------------VLRMSRPGSA------------------   295
PZC79131.1        PW------------------------------VLRMSRPGSA------------------   362
KAF4083067.1      ------------------------------------------------------------   303
XP_009924659.1    --VL-------------------------------------------G-SFQ-------Q   342
XP_010123544.1    --AL-------------------------------------------R-SFQ-------Q   341
CBN81618.1        --AD-------------------------------------------K--EQ-------S   336
KAF3704230.1      --NE-------------------------------------------A-EGD-------Q   333
RUS86578.1        KKVRLFK------------------------------KATTPSKKVMP-EPSN-----DQ   387
XP_034309618.1    PSGDSFD------------------------------KSLQKMKDSDR-SDS--------   368
PIK58946.1        PRMTVVP------------------------------SYSERIRTLME-ETAQ-----TE   398
VUZ42516.1        -KWLRLQ------------------------------PG-PLRSTKSLQAETN--ISKIE   377
PAA51166.1        GRLYMVR------------------------------QSIPLASHKPP-PQ-------QQ   413
TGZ55997.1        GRLVYVR------------------------------QRIPLPDVKKT-GS-------HS   408
VDP48851.1        KNEQ----------------------------------------NRYF-GG-------GF   354
PVD38331.1        ARILCLR------------------------------DKVADPDYSTS-SEKK---PSRS   363
VDK73355.1        PKILFMR------------------------------RPMNDANDSFY-KSRK---EN--   353
VDD84963.1        PKILFMT------------------------------RPTTDEKTNVY-K----------   387
VDK42196.1        PEVLFMR------------------------------RPVAESDDSHR-RVSQ---R---   318
KFD58288.1        PKVLMMQ------------------------------RPTTTLTTDS---DKR---YK--   395
OUC49089.1        PKLLMMR------------------------------RPEPEPLSVAT-VDKA---PA--   394
EYC26492.1        PKYLLMK------------------------------RPPQPGSKQKK-D-SR---TT--   419
VDK27218.1        PKYLLMR------------------------------RPLPAKISKGS-S----------   384
VDM17286.1        SPVLCLQ------------------------------RFDEIRIRYLR-HPL----ST--   298
VDL91846.1        PRLMRLN------------------------------PPRSEASRNKV-CD-------DH   365
KAE9548540.1      PKLLMMS------------------------------RPEKSPKKEEH-SNSL---VDAS   387
PDM74087.1        PKFLCMR------------------------------RPKATEKRKCN-LK-----NVKK   358
XP_024504322.1    PKFLCMK------------------------------RPKQISA--VK-KKTN---KMAS   361
PIO52336.1        PHAMCMQ------------------------------RPKSQVRKPIA-EPPH-----PE   357
TKR73865.1        PKLLCMQ------------------------------RPKQAKRNVVK-KKPE---VKNG   364
RLU23395.1        PAILFMR------------------------------RPKKTRLRWMM-EIPN---VT--   371
XP_002427906.1    PTILFMK------------------------------RPKKTRLRWMM-EMPG---MS--   372
TMW47392.1        PAILLMK------------------------------RPRKTRLRWMM-EMPG---SS--   372
VDD83956.1        PKVLRIK------------------------------RYEHPGSKQIG-R----------   379
VEL33078.1        PKILLMT------------------------------PARESDESNDE-EKQR---KS--   314
XP_009019088.1    PRCLFMK------------------------------RPEIENHEDV----YL---KHII   375
KAE9536378.1      PKMLLMR------------------------------VPEQLLADSAL-K-------QKH   411
KDR23473.1        PRLLLMK------------------------------VPEQLLDESAA-N-------KNR   360
KAF7266914.1      PKMLIMR------------------------------RTTYTIP-EYD-DSQP---PR--   394
KAF4519445.1      PRILFMR------------------------------RPHYSPRDHYE-EPFS---DN--   392
OXU25983.1        PRMLMMR------------------------------RTPYSTP-EYD-DTYL---DS--   379
TGZ32403.1        PRVLMMR------------------------------RTPYSTP-EYD-DAYM---DS--   389
ELT94491.1        PRLLLMK------------------------------RPQTRRDKYPK----------VA   376
VVC39575.1        PRMLIMR------------------------------RPHYQMERKSL-MGTC---HRIM   473
RVE49089.1        PRLLVMR------------------------------RPHFRVDPHRS------------   369
KAB0800277.1      PKFLFIE------------------------------RPKKDDDDNKG-GDSM---LTDV   374
TDG52197.1        PRLLCIE------------------------------RPKKEDPEEDQ-PPEV---LTDV   360
TMW48669.1        ------------------------------------------------------------   335
XP_025896085.1    AALLCIRHRSELCPGR--------------------------V-----------------   370
```

```
KAF2977017.1        TILLCIRDRKKFSQSR--------------------------M-----------------      174
XP_009321837.1      TVLLCIRDRKKFSRSR--------------------------T-----------------      349
XP_009979985.1      AVLLCIRDREKFSQSR--------------------------T-----------------      322
XP_028942374.1      PVLLCIRDRKKFSQSR--------------------------T-----------------      346
PKK19633.1          TVLLCIGDRKKFSQSR--------------------------T-----------------      412
XP_009894240.1      TVLLCIRDRKKFSQSR--------------------------T-----------------      465
XP_032820219.1      SVILCRKQIPYPR-SV--------------------------T-----------------      397
XP_029429480.1      AVLLCIRAKKGI---Q--------------------------T-----------------      398
XP_030077572.1      AVILCIQDKKRFHMAQ--------------------------T-----------------      348
XP_033774596.1      AVILCIQDKRRFQMAQ--------------------------T-----------------      379
ETE72600.1          TILLCIQDRKAFHPAC--------------------------T-----------------      365
XP_018082638.1      TVILCIRDKKKFAAVR--------------------------V-----------------      581
XP_032905410.1      TVWFCIPNKKPLSPAL--------------------------P-----------------      368
XP_020773490.1      PVLFCIHQKHRLCSRLS------------------SHSQ---------------------      378
XP_033833634.1      PVLFCIHQKHRLCSRLS------------------SHSQ---------------------      404
XP_030215795.1      PVLFCIHTKHRLCSMLS------------------THSSSSSSSTS--------------      384
XP_030602980.1      PVLFCIHKKHRLCSRLS------------------SQ-----------------------      381
XP_004573543.1      PVLFCIHKKHRLCSRLS------------------SQ-----------------------      375
XP_005916159.1      PVLFCIHKKHRLCSRLS------------------SQ-----------------------      375
XP_013132089.1      PVLFCIHKKHRLCSRLS------------------SQ-----------------------      375
XP_031603488.1      PVLFCIHKKHRLCSRLS------------------SQ-----------------------      381
XP_005725536.1      PVLFCIHKKHRLCSRLS------------------SQ-----------------------      381
XP_026038075.1      PVLFCIHKKHRLCSRLS------------------SQ-----------------------      381
CAF96649.1          -----------------------------------AE-----------------------      340
XP_023818570.1      PVLFCIRKKHRLCSRLS------------------SQ-----------------------      371
RVE64289.1          PVLFCIHKKHRLCSRLS------------------SQ-----------------------      406
XP_024121971.1      PVLFCIHKKHRLCSRLS------------------SQ-----------------------      377
XP_015817559.1      PVLFCIHKKHRFCSRLS------------------TH-----------------------      361
XP_015225670.1      ------------------------------------------------------------      295
XP_012722063.1      PVLFCIHKKHRFCARLS------------------SQ-----------------------      375
XP_032431307.1      PVLFCIHKKHRFCARLS------------------SQ-----------------------      375
XP_014328329.1      PVLFCIHKKHRFCARLS------------------SQ-----------------------      375
XP_027886578.1      PVLFCIHKKHRFCARLS------------------SQ-----------------------      375
XP_008426791.1      PVLFCIHKKHRFCARLS------------------SQ-----------------------      375
XP_014838686.1      PVLFCIHKKHRFCARLS------------------SQ-----------------------      375
XP_014885967.1      PVLFCIHKKHRFCARLS------------------SQ-----------------------      375
XP_033954312.1      PVLFCIHRKPRLCSNLT------------------SQ-----------------------      375
XP_034089244.1      PMLFCIHRKPRLCSNLT------------------SQ-----------------------      375
XP_010780064.1      ------------------------------------------------------------      330
XP_033970261.1      PVLFCIHRKPRLCSNLT------------------SQ-----------------------      391
XP_029375575.1      PVLFCIHKKHRLCSQLS------------------SE-----------------------      412
KAF0023022.1        PVLFCIHKKHRLCSRLS------------------SQ-----------------------      408
XP_019952614.1      PVLFCIHKKHRLCSRLS------------------SQ-----------------------      364
XP_034463117.1      PVLFCIHKKHRLCSRLS------------------SQ-----------------------      375
XP_029924656.1      PVLFCIHKKHRLCSRLS------------------SQ-----------------------      376
XP_028323228.1      PVLFCIHQKHRLCSSLS------------------S------------------------      374
TNM84480.1          ------------------------------------------------------------      311
XP_029703788.1      PVLFCIRRKHRLCSRLS------------------SQ-----------------------      375
XP_030010368.1      PVLFCIHQKHRLCSTLS------------------SQ-----------------------      378
XP_019725056.1      PALFCIHKKHRLCSRLS------------------SQ-----------------------      376
XP_034034934.1      TTLFCIHKKHRLCSTLS------------------SQ-----------------------      364
XP_029956880.1      PVLFCIHKKHRLCSRLS------------------SQ-----------------------      375
XP_008331307.3      PVLFCIHKKHRLCSRLS------------------SH-----------------------      408
XP_031724845.1      PVLFCIHKKHSLCPTLS------------------SQ-----------------------      376
XP_013859395.1      PVLFCIHKKHRFCSGLS------------------SQ-----------------------      375
XP_017275335.1      PVLFCIHKKHRFCSRLS------------------SQ-----------------------      375
XP_020496197.1      PVLFCIHKKHRLCSRLS------------------SQ-----------------------      375
XP_029030400.1      PVLFCIHQKHRLCSRLS------------------SR-----------------------      375
XP_022060538.1      PVLFCIHKKHRLCSRLS------------------SG-----------------------      376
XP_023150584.1      PVLFCIHKKHRLCSRLS------------------SQ-----------------------      376
XP_029303892.1      PVLFCIHKKHRLCSTLS------------------SQ-----------------------      399
XP_020454172.1      PVLFCIHKNHRFCSRLS------------------SQ-----------------------      374
TKS83244.1          PVLFCIHKKHRLCSRLS------------------SQ-----------------------      362
XP_022616583.1      PVLFCIHKKHRLCSRLS------------------SE-----------------------      408
XP_030293172.1      PVLFCIHKKHRLCSRLS------------------SQ-----------------------      375
XP_027129539.1      PVLFCIHKKHRLCSRLS------------------SQ-----------------------      375
XP_034539538.1      PVLFCIHKKHRLCSRLS------------------SQ-----------------------      375
XP_008277330.1      PVLFCIHKKHRLCSRLS------------------SQ-----------------------      375
XP_028276940.1      PVLFCIHQKHRFCSRLS------------------SQ-----------------------      375
XP_023263433.1      PVLFCIHKKHRLCSRLS------------------SE-----------------------      375
XP_034406336.1      PALFCIHKKHSLCPTLS------------------SQ-----------------------      375
XP_026184460.1      PVLFCIHKKHHLCSRLS------------------SQ-----------------------      375
KAF1378228.1        PVLFCIHKKHRLCSTLS------------------SQ-----------------------      360
XP_028450365.1      PVLFCIHKKHRLCSTLS------------------SQ-----------------------      375
XP_031163851.1      PVLFCIHKKHRLCSTLS------------------SQ-----------------------      375
XP_032389084.1      PVLFCIHKKHRLCSTLS------------------SQ-----------------------      375
XP_033494682.1      PVLFCIHKKHRLCSSLS------------------SQ-----------------------      375
```

```
XP_018544782.1      PVLFCIHKKHRLCSRLS------------------SQ-----------------------      375
XP_026228189.1      PVLFCIHKKHRLCSRLS------------------SQ-----------------------      375
XP_028976606.1      TVLLCIRNKHSLCSVLS------------------RDSD---------------------      413
CAB1352378.1        TVLLCIRNKHSLCSMLS------------------RDSD---------------------      341
XP_023866849.1      TVLLCIRNKHSLCSMLS------------------RDSD---------------------      377
XP_013992832.1      TVLLCIRNKHSLCSMLS------------------RDSD---------------------      377
XP_029546688.1      TVLLCIRNKHSLCSMLS------------------RDSD---------------------      377
XP_021481546.1      TVLLCIRNKHSLCSMLS------------------RDSD---------------------      377
XP_020339889.1      TVLLCIRNKHSLCSILS------------------RDSD---------------------      377
XP_029481972.1      TVLLCIRNKHSLCSMLS------------------RDSD---------------------      377
KPP68743.1          PLLLCIRDKHKFCPVLS------------------RGSD---------------------      354
XP_023669383.1      PLLLCIRDKQKFCPALS------------------RGSD---------------------      376
KAA0720096.1        TVLLCIRNK-KFCPLLS------------------QGSD---------------------      281
XP_009293684.1      TVLLCIRNK-KICSFRS------------------QGSD---------------------      375
XP_026090784.1      TMLLCIRNK-KFCSNLT------------------QGSD---------------------      375
XP_018918715.1      TVLFCIRNK-KVCSVLS------------------QGSD---------------------      375
KAF4098549.1        TVLLCIRNK-KVCSILS------------------QGSD---------------------      375
XP_016084173.1      TVLLCIRNK-KVCSILS------------------QGSD---------------------      375
XP_016332759.1      TILLCIRNK-KVCSILS------------------QGSD---------------------      375
XP_016398039.1      TVLLCIRNK-KVCSILS------------------QGSD---------------------      375
XP_026989970.1      TILLCIRNK-KFCSLLV------------------HESD---------------------      375
TSQ12698.1          TVLLCIRNK-KFCSLLA------------------HESD---------------------      375
XP_017347546.1      TLLLCIRNK-KFCSLLA------------------HESD---------------------      370
XP_026794616.2      TVLLCIRNK-KFCSLLS------------------HESD---------------------      375
XP_026861889.1      TMLLCIRNR-MSCSLLA------------------RDSD---------------------      375
XP_017575347.1      AVLLCIPNK-KFCSLLA------------------RDSD---------------------      375
XP_022531596.1      TVLLCIRNK-KFCSLLN------------------RESD---------------------      375
XP_012691019.2      ATLLCVRDKHKFCSSLL------------------ASSP---------------------      359
XP_028839723.1      TTLLCMRNKHNLCSILS------------------RDSL---------------------      376
XP_030638860.1      TVLLCIR--HKLCTVLS------------------RDSD---------------------      356
XP_007882964.1      AALLCLRGRHRLSPDP--------------------------H-----------------      350
GCB70244.1          TILLCLRNKKWFHSTS--------------------------Q-----------------      351
GCC26242.1          TVLLCLRNKTQFHSMH--------------------------R-----------------      372
XP_020387034.1      TVLLCLRNKTQFRSMR--------------------------Q-----------------      363
XP_028931940.1      ASLFCLQQPPPRM-----------------------------------------------      374
XP_031762490.1      TALLCIRDKKKFGAVR--------------------------V-----------------      366
PIO32240.1          TFLLCIRDKKKYSVSR--------------------------I-----------------      164
XP_018425466.1      TLLLCIRDKKKFGTSQ--------------------------I-----------------      367
XP_006642351.1      TVLLCIRNKEKFGPIP--------------------------S-----------------      389
XP_028665372.1      TVLFCIRNKHKFGPIH--------------------------S-----------------      372
GCF49814.1          VVLLCIRDRSAFCPVR--------------------------T-----------------      353
XP_015277816.1      VVLLCIRDRKAFCPIR--------------------------T-----------------      376
XP_005987340.2      TALLCIWDKKRFGPIR--------------------------T-----------------      356
XP_019339534.1      TVLLCCRDRKQFSPMW--------------------------A-----------------      373
XP_025063414.1      TVLLCCRDRKQFSPMW--------------------------A-----------------      373
XP_019367488.1      AVLLCCRDRKQVSPAW--------------------------A-----------------      373
XP_019412078.1      AILLCCRDRKQVSPAW--------------------------A-----------------      373
XP_014434314.1      TILLCIRNRKKFYPSR--------------------------T-----------------      373
XP_007060556.1      TILLCIRERQKFYPSR--------------------------T-----------------      367
XP_024072175.1      TILLCIRDRKKFYPSR--------------------------T-----------------      352
XP_008170567.1      TILLCIRDRKKFSPSR--------------------------T-----------------      373
XP_034610543.1      TILLCIRDRKKFSPSR--------------------------T-----------------      373
XP_030394127.1      TILLCIRDRKKFYPSR--------------------------T-----------------      373
XP_032651960.1      TILLCIRDRKQFYPSR--------------------------T-----------------      373
XP_025020083.1      TVLLCIQDRRAFHPVC--------------------------T-----------------      373
XP_026536833.1      TVLLCIQDRKAFRPTC--------------------------T-----------------      373
XP_026564787.1      TVLLCIRDRKGFRPTC--------------------------T-----------------      373
XP_032084675.1      TILLCIHDRKAFHPVC--------------------------T-----------------      358
XP_029139341.1      TILLCIQDRKAFRPIC--------------------------T-----------------      373
XP_034281006.1      TILLCIQDRKAFRPVC--------------------------T-----------------      373
XP_020649062.1      TILLCVRDRSAFCPVR--------------------------T-----------------      373
XP_008117087.1      TVLLCIRDRHTFRPVR--------------------------T-----------------      373
XP_028564324.1      TILLCIRDRNTFCPVH--------------------------T-----------------      379
XP_033028155.1      TILLCIRDRNTFCPVH--------------------------T-----------------      378
XP_025963249.1      AVLLCIRDRNKFSHSR--------------------------T-----------------      360
XP_009668348.1      TVLLCIRDRNQFSHSR--------------------------A-----------------      372
XP_013798935.1      TVLLCIRDRNKFIHSK--------------------------T-----------------      356
XP_025913685.1      TVLLCIRDRNKFIHSK--------------------------T-----------------      381
XP_013042552.1      AVLLCIRDRKKFSQSR--------------------------A-----------------      379
XP_005030458.2      AVLLCIRDRKKLSQSR--------------------------A-----------------      372
XP_032057953.1      AVLLCIRDRKKLSQSR--------------------------A-----------------      355
XP_021232050.1      TALLCIWDRKKFSQSR--------------------------T-----------------      374
OXB62403.1          TALLCIWDRKKFSQSR--------------------------T-----------------      373
OXB81319.1          TALLFIWDRKKFSQSR--------------------------T-----------------      355
XP_010722007.1      TALLCIWDRKKFSQSR--------------------------T-----------------      374
XP_015739349.1      TALLCIWDRKKFSQTR--------------------------T-----------------      374
XP_031455498.1      TALLCIWDRKKFSQSR--------------------------T-----------------      375
```

POI27435.1	TALLCIWDRKKFSQSR--------------------------T-----------------	350
XP_004948120.1	TALLCIWDRKKFSQSR--------------------------T-----------------	375
XP_032851190.1	TVLLCIRDRKKFTQST--------------------------T-----------------	370
XP_010007255.1	TVLLCIRDRKKLSQSR--------------------------T-----------------	372
XP_030320702.1	PILLCIRDKKKFSQSR--------------------------P-----------------	343
XP_010191940.1	TVLLCIRDGKKFSQSR--------------------------T-----------------	370
XP_027737112.1	TVLLCIRDRKKFSQSR--------------------------T-----------------	368
XP_027555032.1	TVLLCIRDRNKFSQSR--------------------------T-----------------	373
XP_032565370.1	TVLLCIRDRKKFSQSR--------------------------T-----------------	419
XP_027511217.1	TVLLCIRDRKKFSQSR--------------------------T-----------------	518
XP_027593499.1	TVLLCIRDRKKFSQSR--------------------------T-----------------	469
XP_017664924.1	TVLLCIRDRKKFSQSR--------------------------T-----------------	370
XP_029817938.1	TVLLCIRDRKKFSQSR--------------------------T-----------------	352
XP_005058641.1	TILLCIRDRKKFRPSR--------------------------M-----------------	403
XP_021385804.1	TILLCIRDRKKFSQSR--------------------------M-----------------	372
XP_030146687.2	TILLCIRDRKKFSQSR--------------------------M-----------------	417
KAF4796420.1	TILLCIRDRKKFSQGR--------------------------T-----------------	372
XP_032937581.1	TILLCIRDRKKFSQSR--------------------------T-----------------	419
XP_031989659.1	TILLCVWDRKKFSQSR--------------------------T-----------------	373
XP_010402086.1	TILLCVWDRKKFSQSR--------------------------T-----------------	372
XP_017594069.1	TILLCVWDRKKFSQSR--------------------------T-----------------	343
XP_014115268.1	TILLCIRDRKTFSQSR--------------------------T-----------------	397
XP_023797108.1	TILLCIRDRKTFSQSR--------------------------T-----------------	343
XP_033375614.1	TILLCIRDRKTLSQSR--------------------------T-----------------	343
XP_014740121.1	TILLCIRDRKKLSQSR--------------------------T-----------------	517
RLV83430.1	TVLLCIRDRKKFSQSR--------------------------M-----------------	363
XP_009096098.2	TILLCVRDRKKFSQSR--------------------------M-----------------	478
TRZ15870.1	TILLCIRDRKKLSQSR--------------------------M-----------------	373
RMB91935.1	TILLCIRDRKKFSQST--------------------------M-----------------	343
XP_030820843.1	TILLCIRDRKKFSQSR--------------------------M-----------------	419
XP_014165179.1	TILLCIRDRKKFSQSR--------------------------M-----------------	421
XP_026653582.1	TILLCIRDRKKFSQSR--------------------------M-----------------	303
PKU35975.1	TILLCIRDRDTFSQSR--------------------------T-----------------	375
XP_014805072.1	TVLLCIRDRDTFSQSR--------------------------T-----------------	369
XP_009818330.1	TILLCIRDRKKFTQSR--------------------------I-----------------	140
OPJ68307.1	TVLLCIGDRKKFSQSR--------------------------T-----------------	372
XP_008936289.1	TVLLCIRDRTQFSQSR--------------------------T-----------------	373
XP_010287046.1	TVLLCIRDRKKFSQSR--------------------------T-----------------	303
XP_005240140.2	TILLCIWDRKKFRQSR--------------------------M-----------------	432
XP_005437752.2	TILLCIWDRKKFRQSR--------------------------M-----------------	432
KFV74811.1	TVLLCIRDRTAFIQSR--------------------------T-----------------	337
XP_010018389.1	TVLLCIRDRKKFSQSR--------------------------T-----------------	380
KQK78711.1	TVLLCIRDRKKFSQTX--------------------------T-----------------	372
XP_009570162.1	TVLLCIRDRKKFSQGR--------------------------T-----------------	371
KFP11268.1	TSLLCIRDRTKISQSR--------------------------T-----------------	335
KFQ98910.1	TVLLCIRDRKKFGQSR--------------------------T-----------------	354
XP_012985202.3	PVLLCIRDRKKFSQTR--------------------------T-----------------	372
XP_010573388.1	TVLLCIQDGRTFSPSR--------------------------T-----------------	371
XP_029879496.1	TVLLCIQDGRTLSPSR--------------------------T-----------------	343
XP_030361086.1	TVLLCIRDRKKFSQSR--------------------------T-----------------	424
KFM00668.1	TVLLCIRDRKKFSQSR--------------------------T-----------------	355
KAF1479074.1	TVLLCIRDRKKFSRSR--------------------------T-----------------	335
KAF1651161.1	TVLLCIRDRKKFSRSR--------------------------T-----------------	336
KAF1673648.1	TVLLCIRDRKKFSRSR--------------------------T-----------------	336
KAF1493319.1	TVLLCIRDRKKFSRSR--------------------------T-----------------	336
KAF1584157.1	TVLLCIRDRKKFSRSR--------------------------T-----------------	336
KAF1571723.1	TVLLCIRDRKKFSRSR--------------------------T-----------------	336
KAF1533169.1	TVLLCIRDRKKFSRSR--------------------------M-----------------	336
KAF1638955.1	TVLLCIRDRKKFSRSR--------------------------T-----------------	336
KAF1549972.1	TVLLCIRDRKKFSRSR--------------------------T-----------------	336
KAF1606914.1	TVLLCIRDRKKFSRSR--------------------------T-----------------	336
KAF1510957.1	TVLLCIRDRKKFSRSR--------------------------T-----------------	336
KAF1498899.1	TVLLCIRDRKKFSRSR--------------------------T-----------------	336
KAF1411525.1	TVLLCIRDRKKFSRSR--------------------------T-----------------	336
KAF1429205.1	TVLLCIRDRKKFSRSR--------------------------T-----------------	336
XP_005334318.1	TWLLCLRE-QSASC-----------------------RPPATT-----------------	364
XP_014395552.1	ALLLCLGE-QPTSH-----------------------RCPAAS-----------------	364
KAB0404854.1	ALLLCPGE-QSTSR-----------------------RPPATS-----------------	360
XP_007494828.1	AVLLCLQD-QPHHSPSA------------------LQRSTVSS-----------------	380
XP_003764254.1	AVLLCLQD-QSYHNPST------------------LQRPTIPS-----------------	380
XP_020845489.1	AVLLCFRN-HPYHSPSA------------------LQRPTVPS-----------------	380
XP_027703160.1	AVLLCLQD-QPYHSSSA------------------LQRPTVPS-----------------	380
XP_017523929.1	VPPLCRRG-QSASP-----------------------RPPTTS-----------------	372
XP_008688428.1	ALLLCLGE-QPASR-----------------------RPPATS-----------------	320
XP_029812166.1	ALLLCLGE-QTASR-----------------------QPPATP-----------------	389
XP_025749781.1	ALLLCLGE-QPASR-----------------------RSPATS-----------------	375
XP_004416432.1	ALLLCLGE-QPASR-----------------------RSPATS-----------------	375

XP_027436262.1	ALLLCLGE-QPASR-----------------------RSPATS-----------------	375
XP_032284025.1	ALLLCLGE-QPAPR-----------------------RSPATS-----------------	375
XP_006735421.1	ALLLCLGE-QPASR-----------------------RSPATS-----------------	375
XP_021552166.1	ALLLCLGE-QPASR-----------------------RSPATS-----------------	375
XP_026361066.1	ALLLCLGE-QPASR-----------------------RPPATS-----------------	375
XP_034523598.1	ALLLCLGE-QPASR-----------------------RPPATS-----------------	375
NP_001297113.1	APLLCLGE-QPASR-----------------------RPLVIS-----------------	375
XP_032215488.1	APLLCLGE-QPASR-----------------------RPLVIS-----------------	375
VCX31483.1	ALLLCLGE-QPASR-----------------------RPLATS-----------------	375
XP_022369003.1	ALLLCLGE-QPASR-----------------------RPLATS-----------------	375
XP_032694248.1	ALLLCLGE-QPASR-----------------------RPLATS-----------------	375
NP_001041584.1	ALLLCLGE-QSASR-----------------------RPPATS-----------------	375
XP_025862501.1	ALLLCLGE-QSASR-----------------------RPPATS-----------------	375
KAF0873564.1	ALLLCLGE-QTASR-----------------------RPPATP-----------------	517
XP_025784751.1	ALLLCLGE------------------------------PPATP-----------------	369
XP_007075625.1	ALLLCLGE-QTASR-----------------------RPPATP-----------------	511
XP_030189489.1	ALLLCLGE-QTASR-----------------------RPPATP-----------------	375
XP_019324655.1	ALLLCLGE-QTASR-----------------------RPPATP-----------------	476
XP_023094886.1	ALLLCLGE-QTASR-----------------------RPPATP-----------------	511
XP_026892357.1	ALLLCLGE-QTASR-----------------------RPPATP-----------------	375
XP_016004457.1	ALFLCLGE-QSASH-----------------------SCPATS-----------------	370
XP_006912860.1	ALLLCLGE-QSTSH-----------------------RCPATS-----------------	370
XP_011363679.1	ALLLCLGE-QSTSH-----------------------RCPATS-----------------	370
XP_016076060.1	SLLLCLGE-QPTSR-----------------------RRPAAP-----------------	375
XP_008148371.1	ALLLCLGE-QPTSH-----------------------RCPAAP-----------------	375
XP_006093568.1	ALLLCLGE-QPTSH-----------------------RCPAAS-----------------	375
XP_006761601.1	ALLLCLGE-QPTSH-----------------------QCPAAS-----------------	375
XP_024426743.1	ALLLCLGE-QPTSY-----------------------RCPATS-----------------	370
XP_019520779.1	ALLLCLGE-QSTSH-----------------------RCPATS-----------------	375
XP_032976539.1	ALLLCLGE-QSTSH-----------------------RCPATS-----------------	375
ELW64270.1	SWLLCLGE-QPTSH-----------------------RPPANS-----------------	379
XP_004427367.1	ALLLCLGE-QPTSR-----------------------RPAATS-----------------	375
XP_008529353.1	TLLLCLGE-QLTSQ-----------------------RPTTTS-----------------	375
NP_001288165.1	TLLLCLGE-QLTSQ-----------------------RPTTTS-----------------	375
XP_014711213.1	TLLLCLGE-QLTSQ-----------------------RPPATS-----------------	375
XP_012514621.1	TRLPCLRE-QSTSH-----------------------RPPAAS-----------------	375
XP_008071525.1	APLLCLRE-QSTSH-----------------------RPPATS-----------------	372
XP_009005082.1	TWLLCLRE-QSTSH-----------------------RSPATS-----------------	375
XP_010332832.1	AWLLCLRE-QSTSH-----------------------RSPATS-----------------	370
XP_012326058.1	AWLLCLRE-QSTSH-----------------------RSPATS-----------------	381
XP_017378796.1	AWLLCLRE-QSTSH-----------------------RPPATS-----------------	404
XP_032141374.1	AWLLCLRE-QSTSH-----------------------RSPATS-----------------	404
XP_032024018.1	AWLLCLRE-QSTSQ-----------------------RPPATS-----------------	370
XP_018891497.2	AWLLCLRE-QSTSQ-----------------------RPPATS-----------------	376
XP_030684894.1	AWLLCLRE-QSTSQ-----------------------RPPATS-----------------	370
PNJ75904.1	AWLLCLRE-QSTSQ-----------------------RPPATS-----------------	376
AAP35868.1	AWLLCLRE-QSTSQ-----------------------RPPATS-----------------	376
XP_001149570.1	AWLLCLRE-QSTSQ-----------------------RPPATS-----------------	376
XP_003805532.1	AWLLCLRE-QSTSQ-----------------------RPPATS-----------------	376
XP_023063823.1	AWLLCLRE-QSTSQ-----------------------RPPATS-----------------	374
XP_033060210.1	AWLLCLRE-QSTSQ-----------------------KPPATS-----------------	374
XP_010370669.1	AWLLCLRE-QSTSQ-----------------------KPPATS-----------------	374
XP_017738496.1	AWLLCLRE-QSTSQ-----------------------KPPATS-----------------	374
XP_011782168.1	AWLLCLRE-QSTSQ-----------------------RPPATS-----------------	375
EHH23440.1	AWLLCLRE-QSTSQ-----------------------RPPATS-----------------	402
XP_003910778.3	AWLLCLRE-QSTSQ-----------------------RPPATS-----------------	375
XP_025212649.1	AWLLCLRE-QSTSQ-----------------------RPPATS-----------------	381
XP_008019132.1	AWLLCLRE-QSTSQ-----------------------RPPATS-----------------	375
XP_011832421.1	AWLLCLRE-QSTSQ-----------------------RPPATS-----------------	360
XP_011759618.1	AWLLCLRE-QSTSQ-----------------------RPPATS-----------------	375
XP_005579749.1	AWLLCLRE-QSTSQ-----------------------RPPATS-----------------	375
XP_011921684.1	AWLLCLRE-QSTSQ-----------------------RPPATS-----------------	167
XP_008259391.1	ARLLCLQE-QVPSQ-----------------------RLPATS-----------------	375
XP_012782438.1	ARLLCLQE-QVTSQ-----------------------RLQATS-----------------	371
VTJ86076.1	TWLLCLRE-QSTSC-----------------------RPPAIT-----------------	381
XP_015345533.1	TWLLCLRE-QSTSC-----------------------RPPATT-----------------	381
XP_027786432.1	TWLLCLRE-QSTSC-----------------------RPPATT-----------------	381
XP_026248345.1	TWLLCLRE-QSASC-----------------------RPPTTT-----------------	375
XP_005378209.1	ARLLCLGE-QLTSH-----------------------RPPATS-----------------	376
XP_004466010.1	AALLCLGE-QSHSH-----------------------RPPAPS-----------------	375
XP_020035707.1	ARLLGQGE-QPTSH-----------------------RPPATS-----------------	369
XP_006181802.1	ALLLCLGE-QSASR-----------------------RPPATS-----------------	375
XP_006207862.1	TLLLCLGE-QSASR-----------------------RPPAPS-----------------	375
XP_005891968.1	TLLLCLGD-QSTAR-----------------------RPAATS-----------------	372
XP_010828662.1	TLLLCLGD-QSTAR-----------------------RPAATS-----------------	372
XP_019830126.1	TLLLCLGD-QSTAR-----------------------RPAATS-----------------	371
XP_017914486.1	TLLLCLGE-QSTAR-----------------------RPAATS-----------------	371

```
XP_006060266.2    TLLLCLGE-QSTAR-----------------------RPAATS-----------------    376
XP_020728447.1    ALLLCIGE-QSTPR-----------------------RPPATS-----------------    376
KAF4008892.1      ALLLCLGE-QSTPR-----------------------RPPATS-----------------    376
KAB0348059.1      ALLLCLGE-QSTPW-----------------------RPPATS-----------------    359
KAB0371113.1      ALLLCLGE-QSTPR-----------------------RPPATS-----------------    352
MBV96963.1        ALLLCPGE-QSTSR-----------------------RPPATS-----------------    375
XP_007172048.1    ALLLCPGE-QSTSR-----------------------RPPATS-----------------    375
XP_007452270.1    SLLLCLGE-QSTFR-----------------------RSPATS-----------------    375
XP_023987108.1    ALLLCLGE-QSTSR-----------------------RPPATS-----------------    370
XP_004273432.1    ALLLCLGE-QSTSR-----------------------RPPATP-----------------    370
XP_004328646.1    ALLLCLGE-QSTSR-----------------------RPPATS-----------------    370
XP_026937670.1    ALLLCLGE-QSTSR-----------------------RPPATS-----------------    370
XP_030691031.1    ALLLCLGE-QSTSR-----------------------RPPATS-----------------    370
XP_022414870.1    ALLLCLGE-QSTSR-----------------------RPPATS-----------------    375
XP_029064651.1    ALLLCLGE-QSTSR-----------------------RPPATS-----------------    375
XP_024607566.1    ALLLCLGE-QSTSR-----------------------RPPATS-----------------    375
XP_032496907.1    ALLLCLGE-QSTSR-----------------------RPPATS-----------------    375
XP_007535559.1    ALLLCLGE-QPTVR-----------------------NPPATP-----------------    368
XP_031299536.1    ALLLCLGE-QSASR-----------------------RPPATS-----------------    374
XP_004666516.1    ARLLCLGE-QPTSQ-----------------------RPTGTF-----------------    370
XP_008830963.1    AWLLCLGE-QPMFR-----------------------RSPGTF-----------------    375
XP_005347338.1    AWLLCLGE-QPMTN-----------------------RPPATF-----------------    375
XP_005069548.1    AWLLCLGE-QPIAH-----------------------RPPATF-----------------    375
XP_027267894.1    AWLLCLGE-QPIAH-----------------------RSPATF-----------------    375
OBS80992.1        AWLLCLGEQQPMAH-----------------------RHPATF-----------------    363
XP_006979812.1    AWLLCLGEQQPMAH-----------------------RHPDTF-----------------    382
XP_028720687.1    AWLLCLGEQQPMAH-----------------------RHPATF-----------------    376
XP_021489005.1    AWLLCLGE-QPRAH-----------------------RSPATF-----------------    376
XP_031199589.1    AWLLCLGE-KPMAH-----------------------RPPATF-----------------    400
XP_028617944.1    ARLLYLGE-QPMAH-----------------------RPPATF-----------------    190
XP_034347030.1    ARLLCLGE-QPMAH-----------------------RPPATF-----------------    373
NP_077370.2       AWLLCLGE-QPMAH-----------------------RPPATF-----------------    375
XP_032766961.1    AWLLCLGE-QPMAQ-----------------------RPPATF-----------------    375
XP_021063186.1    AWILCLGE-QPMAH-----------------------RPPATF-----------------    358
NP_001093114.1    AWILCLGE-QPMAH-----------------------RPPATF-----------------    375
XP_021028379.1    AWILCLGE-QPMAH-----------------------RPPATF-----------------    375
XP_004689305.1    ALLLCLGE-QPPAR-----------------------RSPATP-----------------    375
XP_004712871.1    ATLLCLGE-RPTSP-----------------------RLSVPT-----------------    375
XP_004382575.1    AVLLCLGE-QLNSP-----------------------RLSATS-----------------    375
XP_010596188.1    AVLLCLGE-QSNSP-----------------------RLSATS-----------------    375
XP_006890855.1    TVLLCLGD-QAASP-----------------------RLSASN-----------------    375
XP_006834038.1    AALVCLGE-QSNSP-----------------------RLPAAS-----------------    375
XP_007934716.1    AVLLCLGE-QSNSP-----------------------RLPAGS-----------------    375
XP_012603491.1    ARLPCLRE-QATSH-----------------------RPPAAS-----------------    375
XP_012663606.1    AWLLCLGE-QTTSR-----------------------RAPTTC-----------------    370
XP_028372812.1    ALLLCLGE-QPTSH-----------------------RCPATS-----------------    375
XP_008589608.1    TQLLCLGE-WSTSQ-----------------------RPPAIP-----------------    377
XP_012865077.1    TWLMCLGE-WPTSH-----------------------RPPAAS-----------------    375
NP_001166178.1    AGLLCLGE-QLTSH-----------------------RGPATL-----------------    376
XP_004636553.1    ARLLCLQE-PLTSP-----------------------RPPATW-----------------    376
XP_004856670.1    AQLLCLGE-QLTSH-----------------------RPPPTL-----------------    376
XP_010627744.1    TQLLCLGE-QLTSH-----------------------RPPATL-----------------    376

NP_509270.1       ------------------------------------------------------------    366
VDO93178.1        ---------------------VPL------------------------------------    377
PAV91580.1        ---------------------INIYR-----------K-KKS--IYTP----------RT    400
VDK46997.1        ------------------------------------------------------------    351
VIO86814.1        -VS--SASDSFTFRDII---PAEV---------NMTECHQET------------------    454
VDP38785.1        ---------------------IHLNCNHNNYIDN---DHIS--------Y----------    439
KOF68401.1        ----------------------------------ITRTHSSRVSQFNPKKE---------    359
CDJ96026.1        --------KSTIRRSSI---MRDL---------KRIKNHDHK------------------    522
EFX76216.1        --------------------NERF---------N--------------------------    340
KAF7403848.1      --------------------MRKV---------N--------------------------    348
KAF7427032.1      --------------------MRKV---------N--------------------------    363
KAE9417558.1      ---------------SL---PSNED----------------E------------------    360
KJH51504.1        -----------------------WK----------NRK----------------------    358
VDL69795.1        ---------------DS---D---------------------------------------    336
VDO32671.1        ---------------EL---PPLKS-----------------------------------    374
VBB32409.1        ---------------AL---PSLFPCT-------KPTTHSESL-----------------    357
VDN54565.1        ---------------TL---PPLLTCS-------KPTRHSESL-----------------    376
EGT55171.1        ---------------SL---PPLFT-S-------KPNRHSESL-----------------    386
RCN52111.1        ---------------SL---PSLFN-S-------KLKSHSESL-----------------    391
RMX54856.1        --------VTFLHRNKI---AIEYRVNEKTKIENAS-TYKA-------------------    404
XP_022781674.1    --------VTFLHRNKT---PKEYGVNNKTKIENAS-THIT-------------------    666
RNA37099.1        LNNSIHKMESFLSTE-S---DVDLACSNEPSTVNE--RHKKKLLNVNSNFQRKKDWTNMY    445
KAA0187152.1      LTSP-NDSHSFFGLKWR---NRSLAKPKEPDENNASGRHSNS-NNVAPPKV--MTSS---    607
```

GAU96593.1	-LSP-PM---------------------------RLSRHTQA-SQDDPEFA--LERSNML	402
XP_009043980.1	---R-TI---------------------------KSND-------MCLSTY-------ME	349
VDM43573.1	-CSA-NG---------------------------MTKR-------VSVGFA-------MD	470
XP_003140283.1	-FVS-NG---------------------------QPRE-------ICSRKI-------I-	360
VDN82010.1	-FVS-NG---------------------------QPGR-------TCSRKI-------I-	385
VDO31501.1	-FVS-NG---------------------------QPGR-------TCSRKI-------I-	397
VDP19591.1	DEEGQIGAKTFVGWNYD---TTNMMNS-----------------NINPIYMKK-------	440
EDO32053.1	--	324
TRY67230.1	-------------SSMI---SH--------------------------------------	351
KAF7391312.1	-------------KS---	362
GFG30449.1	-------------WGSE---NP--------------------------------------	393
KQS30083.1	-------------REEA---TP--------------------------------------	395
EDW57583.2	-------------EFPT---TP--------------------------------------	534
KNC22799.1	-------------DFSS---TP--------------------------------------	344
RZF44856.1	-------------GGEA---TS--------------------------------------	364
ENN76856.1	-------------STYS---PP--------------------------------------	370
KAF5300392.1	-------------YEYQ---NP--------------------------------------	371
CAB3239999.1	-------------TTPP---PA--------------------------------------	358
PCG77624.1	-------------TTPP---PA--------------------------------------	301
PZC79131.1	-------------TTPP---PA--------------------------------------	368
KAF4083067.1	--	303
XP_009924659.1	--	342
XP_010123544.1	--	341
CBN81618.1	LSED----------------CGDKR-----------------------------------	345
KAF3704230.1	RATE----------------SGNNQ-----------------------------------	342
RUS86578.1	-------------------------V--DPE-----------------------------	391
XP_034309618.1	--	368
PIK58946.1	L---------R------------------SSSINGIG-----------------------	408
VUZ42516.1	MHLN-QERKLR------------------------------R-----------R--DSFE	393
PAA51166.1	LLSS-QP--QNQQHGSLATS---------P------------------------------	431
TGZ55997.1	SPRG-SAAKSRSQHSMLEKDGNKLFLNYVPYGEDGR-----------------T--GYYG	448
VDP48851.1	FPQC-SE-------SVNQPACGDQYLENVTTNWSGS-----------------N--EFQT	387
PVD38331.1	WNTG--------------RVGGNPKG-QYPYT----------------------------	380
VDK73355.1	LRTN-SKQEECGRK-------RGEQIAI--------------------------------	373
VDD84963.1	-------------I-------GNEKVAL--------------------------------	395
VDK42196.1	-----------RGD-------NCEKVAI--------------------------------	328
KFD58288.1	ADTE-QTE-EI--------------MMT--------------------------------	407
OUC49089.1	FELN-QSENVI--------------MMT--------------------------------	407
EYC26492.1	FSTP-HGNSQFASAL------ADQNLDL--------------------------------	440
VDK27218.1	--	384
VDM17286.1	---------------------GEVQM----------------------------------	303
VDL91846.1	LHCC-LSG----PYGTN----KTAFMKRIPKKLQRLR--ERK-----------R--ESMQ	401
KAE9548540.1	---------------------ALIAMPSLAYPIPSTEIKSKK-----------K--QKFA	413
PDM74087.1	MTAL----------------PGLGNFSSSPAP----------------------------	374
XP_024504322.1	KSKL----------------PGVGEFTISKSS----------------------------	377
PIO52336.1	VHQL----------------PGLGHFTINPAA----------------------------	373
TKR73865.1	LMAL----------------PGLGEFTVNPAH----------------------------	380
RLU23395.1	LPA-------S-TYS-----GSPTEV---PKHLPAS-LAK----------------SKME	398
XP_002427906.1	APP-------H-SY------GSPGEL---PKHFQP----R----------------AKVE	395
TMW47392.1	AQT-------YPTSY-----GSPAEM---PKQISAIGGKQ----------------SKME	401
VDD83956.1	--------------LSR-----------SPPS---LY-V---------------------	389
VEL33078.1	TR-----EDLRVEQISE--ENGGQNIPSLAQS----T-NS----------------G---	343
XP_009019088.1	LCTC-FKNKSHDVNS-------------LQ------------------------------	391
KAE9536378.1	MKTC-KKLNLNLARLNAASVGATLNVPPAPAGSPPAG-HR----------------S---	450
KDR23473.1	LRAA-AKSKLNAAAA-------------APAASSSSG-AS----------------S---	386
KAF7266914.1	------------GYPN-----GDMEYVCLKPF----------------------------	409
KAF4519445.1	------------GFGG-----GN-EF----------------------------------	400
OXU25983.1	------------GYTN-----EM-DFSFR-DS----------------------------	392
TGZ32403.1	------------AYTN-----EI-DYS---------------------------------	398
ELT94491.1	VRTC-DGVEIRDSYANH-NA--------HPPR----------------------------	398
VVC39575.1	VRTC----NGLELRDQD-SADGLL-----QAR----------------------------	495
RVE49089.1	---------RFAEIGFV-PSSGLLGGEPVPWE----------------------------	391
KAB0800277.1	IHIP--GSDKFKAYDNN-SFGGSFDLGILPPS----------------------------	403
TDG52197.1	FHLP-PDVDKFVNYDTK-RFSGDYGIPVLPAS----------------------------	390
TMW48669.1	--	335
XP_025896085.1	-----HSESAGKGGASG----------------SACRAQ----------S----------	389
KAF2977017.1	-----QTLDTSRQVENN----------------DS-------------------------	188
XP_009321837.1	-----QSSDISRQVDNN----------------DS-------------------------	363
XP_009979985.1	-----QSSDISGQAESN----------------DS-------------------------	336
XP_028942374.1	-----QSSDISRQVENH----------------NS-------------------------	360
PKK19633.1	-----QSSDISGEVENN----------------DS-------------------------	426
XP_009894240.1	-----QSSDISRQAESN----------------DS-------------------------	479
XP_032820219.1	-----FPNDGAQNAADN----------------E--------------------------	410
XP_029429480.1	-----QSSELSLQKENS----------------TST------------------------	413
XP_030077572.1	-----RSSEISLQKENS----------------TSTGGMD--------GA----------	369
XP_033774596.1	-----QSSEISLQKENS----------------AST------------------------	394
ETE72600.1	-----QSSNISRDMENN----------------DST------------------------	380

```
XP_018082638.1      -----HSSDISHQMENN----------------S-T------------------------      595
XP_032905410.1      -----YDRKHNP-----------------------A------------------------      376
XP_020773490.1      ------TSDLEHYKDTN----------------YGTAQCT--------------------      396
XP_033833634.1      ------SSDLD-------------------------------------------------      409
XP_030215795.1      -SSSQAASDLERFKEPG----------------YGRGPCS--------------------      407
XP_030602980.1      ------SSDMENYKENN----------------YGTAPCS--------------------      399
XP_004573543.1      ------STDMENYKENN----------------YG-TPCS--------------------      392
XP_005916159.1      ------STDMENYKENN----------------YGSAPCS--------------------      393
XP_013132089.1      ------STDMENYKENN----------------YGTAPCS--------------------      393
XP_031603488.1      ------STDMENYKENN----------------YGTG-----------------------      396
XP_005725536.1      ------STDMENYKENN----------------YGSG-----------------------      396
XP_026038075.1      ------STDMENYKENN----------------YGSAPCS--------------------      399
CAF96649.1          ------GSDLDRYKDSS----------------YGTTQCT--------------------      358
XP_023818570.1      ------ASDLEHYKENN----------------YGTAQCT--------------------      389
RVE64289.1          ------ASDLEHYKENN----------------YGTAQCS--------------------      424
XP_024121971.1      ------ASDLEHYKENN----------------YGTAQCT--------------------      395
XP_015817559.1      ------ASDLEHYKENN----------------YGTAQCS--------------------      379
XP_015225670.1      ------------------------------------AQCS--------------------      299
XP_012722063.1      ------ASELDRYKEND----------------YGTAQCS--------------------      393
XP_032431307.1      ------ASELDHSKENN----------------YGTAQCS--------------------      393
XP_014328329.1      ------ASELDHSKENN----------------YGTAQCS--------------------      393
XP_027886578.1      ------ASELDHSKENN----------------YGTAQCS--------------------      393
XP_008426791.1      ------ASELDRSKEYN----------------YGTAQCS--------------------      393
XP_014838686.1      ------ASELDRSKENN----------------YGTAQCS--------------------      393
XP_014885967.1      ------ASELDRSKENN----------------YGTAQCS--------------------      393
XP_033954312.1      ------PPDLELYKESS----------------YGTAQCP--------------------      393
XP_034089244.1      ------PPDLELYKESS----------------YGTAQCP--------------------      393
XP_010780064.1      ------------------------------------------------------------      330
XP_033970261.1      ------PPDLELYKESS----------------YGTAQCP--------------------      409
XP_029375575.1      ------ASDLEHYKDNN----------------YGTAQCT--------------------      430
KAF0023022.1        ------ASDLEHYKDNS----------------YGTARCG--------------------      426
XP_019952614.1      ------ASDLEHYKDNS----------------YGTARCT--------------------      382
XP_034463117.1      ------ASDLEHYKDNS----------------YGTAARC--------------------      393
XP_029924656.1      ------ASDMEHYKD-N----------------YGTA--Q--------------------      391
XP_028323228.1      --------DLEPYKD-N----------------YGTAAQ---------------------      388
TNM84480.1          ------------------------------------------------------------      311
XP_029703788.1      ------GSDLDHYKDSS----------------YGTTQ----------------------      391
XP_030010368.1      ------ASDLEHCKENN----------------YGSAQ----------------------      394
XP_019725056.1      ------TSDMADYKDDN----------------YGTVQ----------------------      392
XP_034034934.1      ------SSDLEHYKDNT----------------YGTAQ----------------------      380
XP_029956880.1      ------ASDLERYKDGS----------------YGTAQ----------------------      391
XP_008331307.3      ------TSDLEHYKDSS----------------YGTARCS--------------------      426
XP_031724845.1      ------GTDLEHYKDNN----------------YGT-AQ---------------------      392
XP_013859395.1      ------ASELEHYKEND----------------YGTAAQ---------------------      392
XP_017275335.1      ------ASELEHYKENT----------------YRTAAQ---------------------      392
XP_020496197.1      ------ASDMDDLSL-S----------------KPA-AQ---------------------      390
XP_029030400.1      ------ASDLDHHKENN----------------YGT-AR---------------------      391
XP_022060538.1      ------TSDLEHYKDSS----------------YGT-AQ---------------------      392
XP_023150584.1      ------ASDLEPYKDSS----------------YGT-AQ---------------------      392
XP_029303892.1      ------GVDLEHYKDNN----------------YGT-AQ---------------------      415
XP_020454172.1      ------ASDLENYKDSS----------------YGT-AP---------------------      390
TKS83244.1          ------ASDLEHY---N----------------YGT-AQ---------------------      375
XP_022616583.1      ------ASDLEHYKDNS----------------YGT-AQ---------------------      424
XP_030293172.1      ------ASDLDHYKDNS----------------YGT-AQ---------------------      391
XP_027129539.1      ------ASDLEHY---N----------------YGT-AQ---------------------      388
XP_034539538.1      ------ASDMEHYKESN----------------YGT-AQ---------------------      391
XP_008277330.1      ------ASDLEHYKDTS----------------YGT-AQ---------------------      391
XP_028276940.1      ------ASDLEHYKDSN----------------YGT-AQ---------------------      391
XP_023263433.1      ------ASDLEHYKENS----------------YGT-AQ---------------------      391
XP_034406336.1      ------GTDLEHYKDNN----------------YGT-AQ---------------------      391
XP_026184460.1      ------ASDLEHYKDNN----------------YGT-AQ---------------------      391
KAF1378228.1        ------GADLEHYKDNS----------------YGT-AQ---------------------      376
XP_028450365.1      ------GADLEHYKDNS----------------YGT------------------------      389
XP_031163851.1      ------GTDLEHYKDNS----------------YGT-AQ---------------------      391
XP_032389084.1      ------GADLEHYKDNS----------------YGT-AQ---------------------      391
XP_033494682.1      ------GADLEHYKDNN----------------YGT-AQ---------------------      391
XP_018544782.1      ------ASDLEHYKDNN----------------YGT-AR---------------------      391
XP_026228189.1      ------ASDLEHYKDNN----------------YGT-AR---------------------      391
XP_028976606.1      --------HPGQYKENN----------------FSPAGQ---------------------      428
CAB1352378.1        --------HLGHYKENN----------------ISPAR----------------------      355
XP_023866849.1      --------HLGHYKENN----------------ISPAR----------------------      391
XP_013992832.1      --------HLGHYKENN----------------ISPAR----------------------      391
XP_029546688.1      --------HLGHYKENN----------------ISPAR----------------------      391
XP_021481546.1      --------HLGHYKENN----------------ISPAR----------------------      391
XP_020339889.1      --------HLGHYKENN----------------ISPAR----------------------      391
XP_029481972.1      --------HLGHYKENN----------------ISPAR----------------------      391
KPP68743.1          --------LS-RYTEHN----------------MNT-AR---------------------      367
```

```
XP_023669383.1      --------LS-HYADHH----------------LNT-SR---------------------     389
KAA0720096.1        --------LP-LYKENN----------------MNTAVR---------------------     295
XP_009293684.1      --------LP-LYKENN----------------MNTAIH---------------------     389
XP_026090784.1      --------LP-QYKENN----------------MNTAVH---------------------     389
XP_018918715.1      --------LP-LYKENN----------------MNTAVH---------------------     389
KAF4098549.1        --------LP-LYKENN----------------MNTAVH---------------------     389
XP_016084173.1      --------LP-LYKENN----------------MNTAVH---------------------     389
XP_016332759.1      --------LP-LYKENN----------------MNTAVH---------------------     389
XP_016398039.1      --------LP-LYKENN----------------MNTAVH---------------------     389
XP_026989970.1      --------LS-HFRENN----------------MNT-VR---------------------     388
TSQ12698.1          --------LP-RYKENN----------------MNT-VR---------------------     388
XP_017347546.1      --------LP-RYRENN----------------MNT-VR---------------------     383
XP_026794616.2      --------LP-RYTENN----------------MNT-VR---------------------     388
XP_026861889.1      --------LA-RYKENN----------------MNT-VR---------------------     388
XP_017575347.1      --------LP-RYKENN----------------MNT-VR---------------------     388
XP_022531596.1      --------LP-HYKENN----------------MNT-VR---------------------     388
XP_012691019.2      --------LP-PYTENN----------------IHTASH---------------------     373
XP_028839723.1      --------LP-RYKENN----------------MNIATR---------------------     390
XP_030638860.1      --------LP-HYKENN----------------MNT-VR---------------------     369
XP_007882964.1      -----HGHHPQPHTDKA----------------GGS-------------A----------     366
GCB70244.1          -----NSETPENPRDRK----------------GST-------------G----------     367
GCC26242.1          -----HSGTPDNARDRK----------------GST-------------V----------     388
XP_020387034.1      -----HSETSDNARDRK----------------DSM-------------V----------     379
XP_028931940.1      ----------ARQAADD----------------SNS-------------S----------     385
XP_031762490.1      -----HSSDISHQMENS----------------STA------------------------     381
PIO32240.1          -----HSPNMSLQMENS----------------STA------------------------     179
XP_018425466.1      -----HSSNMSRQMENS----------------STA------------------------     382
XP_006642351.1      -----RGCDMSRFKENN----------------MNT-------------G----------     405
XP_028665372.1      -----SRRDVSQYMENN----------------VTA-------------A----------     388
GCF49814.1          -----QSSDVSRSMESN----------------GSTA-----------------------     369
XP_015277816.1      -----QSSDSSRNIENN----------------GSTG-----------------------     392
XP_005987340.2      -----QSADTCQYNENN----------------TST-------------A----------     372
XP_019339534.1      -----QSAESTRHLESN----------------DSS-------------T----------     389
XP_025063414.1      -----QRAESTRHLESN----------------DSS-------------T----------     389
XP_019367488.1      -----QSAESTRHLDSN----------------DSS-------------T----------     389
XP_019412078.1      -----QSAESTRHLESN----------------DSS-------------T----------     389
XP_014434314.1      -----QSSEISRRPEDD----------------DSTGSSL------IPAD----------     396
XP_007060556.1      -----PSSDISRHPENN----------------DST-------------A----------     383
XP_024072175.1      -----QSSDISRHPENN----------------DSTGTSCGT----GKGA----------     377
XP_008170567.1      -----QSSDISRHPENN----------------DST-------------A----------     389
XP_034610543.1      -----QSSDISRHPENN----------------DST-------------A----------     389
XP_030394127.1      -----QSSDISQHPENN----------------DST-------------A----------     389
XP_032651960.1      -----QSSDISRHLENN----------------DST-------------A----------     389
XP_025020083.1      -----QSSDISKNTENN----------------GST-------------A----------     389
XP_026536833.1      -----QSSNISRDMENN----------------DST-------------A----------     389
XP_026564787.1      -----QSSNISRDMENN----------------DST-------------A----------     389
XP_032084675.1      -----QSSSISRNTENN----------------DST-------------A----------     374
XP_029139341.1      -----QSSNISRNIENN----------------DST-------------A----------     389
XP_034281006.1      -----QSSNISRNMENN----------------DST-------------A----------     389
XP_020649062.1      -----QSSDISRNTENN----------------DST-------------A----------     389
XP_008117087.1      -----QSSDISRNMENN----------------DST-------------A----------     389
XP_028564324.1      -----QSSDISGNMENN----------------DST-------------A----------     395
XP_033028155.1      -----QSSDISGNMENN----------------DST-------------A----------     394
XP_025963249.1      -----QSSDISRH---N----------------DST-------------A----------     373
XP_009668348.1      -----QSSDISRHVENN----------------GST-------------A----------     388
XP_013798935.1      -----QSSDISRHMENN----------------DST-------------A----------     372
XP_025913685.1      -----QSSDISRHMENN----------------DST-------------A----------     397
XP_013042552.1      -----QSSDISRHVENN----------------DST-------------A----------     395
XP_005030458.2      -----QSSDISRHVENN----------------DST-------------A----------     388
XP_032057953.1      -----QSSDISRHVENN----------------DST-------------A----------     371
XP_021232050.1      -----QSSDISRHVENN----------------DST-------------G----------     390
OXB62403.1          -----QSSDISRHVENN----------------DST-------------A----------     389
OXB81319.1          -----QSSDISRHVENN----------------DST-------------A----------     371
XP_010722007.1      -----QSSDISRHMENN----------------DST-------------A----------     390
XP_015739349.1      -----QSSDISRHVENN----------------DST-------------A----------     390
XP_031455498.1      -----QSSDISRHVENN----------------DST-------------A----------     391
POI27435.1          -----RSSDISRHVENN----------------DST-------------A----------     366
XP_004948120.1      -----QSSDISRHVENN----------------DST-------------A----------     391
XP_032851190.1      -----QGSDIAGQVENN----------------DST-------------A----------     386
XP_010007255.1      -----QSLDITRQVENT----------------DST-------------A----------     388
XP_030320702.1      -----QSSDISRQVENT----------------DST-------------G----------     359
XP_010191940.1      -----QSLDISGQGENN----------------DST-------------A----------     386
XP_027737112.1      -----QSSDISRQAENN----------------DST-------------A----------     384
XP_027555032.1      -----QSSDISRQAENN----------------DST-------------A----------     389
XP_032565370.1      -----QSSDISRQAENN----------------DST-------------A----------     435
XP_027511217.1      -----QSSDISRQAENN----------------DST-------------A----------     534
XP_027593499.1      -----QSSDISRQAENN----------------DST-------------A----------     485
```

```
XP_017664924.1      -----QSSDISRQAENN----------------DST-------------A----------      386
XP_029817938.1      -----QSSDISRQAENN----------------DST-------------A----------      368
XP_005058641.1      -----QSLDTSRQVENN----------------DST-------------G----------      419
XP_021385804.1      -----QNLDTSRQVENN----------------DST-------------G----------      388
XP_030146687.2      -----QNLDTSRQVENN----------------DST-------------A----------      433
KAF4796420.1        -----QSSDTSRQVENN----------------DST-------------A----------      388
XP_032937581.1      -----PSLDTSRQVENH----------------DST-------------A----------      435
XP_031989659.1      -----QSLDISRQMENN----------------DST-------------A----------      389
XP_010402086.1      -----QSLDISRQVENN----------------DST-------------A----------      388
XP_017594069.1      -----QSLDISRQVENN----------------DST-------------A----------      359
XP_014115268.1      -----QSLDTSRQVENN----------------DST-------------A----------      413
XP_023797108.1      -----QSLETSRQVENN----------------DST-------------A----------      359
XP_033375614.1      -----QSLDTSRQVENN----------------DST-------------A----------      359
XP_014740121.1      -----QSLDTSRQVESN----------------DST-------------A----------      533
RLV83430.1          -----QDLDTSRQVENN----------------DST-------------A----------      379
XP_009096098.2      -----QTLDTSRQVENN----------------DST-------------A----------      494
TRZ15870.1          -----QSLDTSGQVENN----------------DST-------------A----------      389
RMB91935.1          -----ESLDTSRQVENN----------------DST-------------A----------      359
XP_030820843.1      -----QTLDTSRQVENN----------------DST-------------A----------      435
XP_014165179.1      -----QTLDTSRQVENN----------------DST-------------A----------      437
XP_026653582.1      -----QTMDTSRRVENN----------------DST-------------A----------      319
PKU35975.1          -----QSSDISRQTE-N----------------DST-------------A----------      390
XP_014805072.1      -----QSSDISRQTE-N----------------DST-------------A----------      384
XP_009818330.1      -----QNLDISRQVENN----------------DST-------------G----------      156
OPJ68307.1          -----QSSDISGQAE-N----------------DST-------------A----------      387
XP_008936289.1      -----QTSGIPREVENN----------------DST-------------A----------      389
XP_010287046.1      -----QSSDISRQAENN----------------DST-------------A----------      319
XP_005240140.2      -----QSSDISRQVESN----------------DST-------------A----------      448
XP_005437752.2      -----QSSDISRQVESN----------------DST-------------A----------      448
KFV74811.1          -----QSMDISGQAENN----------------DST-------------A----------      353
XP_010018389.1      -----QHSDISRQVENN----------------DST-------------A----------      396
KQK78711.1          -----QSSDISRQEENT----------------DST-------------A----------      388
XP_009570162.1      -----QSSDISRQVENN----------------DST-------------A----------      387
KFP11268.1          -----QSSDISGQVESN----------------DST-------------A----------      351
KFQ98910.1          -----QSSDISRQVENN----------------DST-------------A----------      370
XP_012985202.3      -----QSSDISGQVENN----------------DST-------------A----------      388
XP_010573388.1      -----QSSDISREVENN----------------DSA-------------A----------      387
XP_029879496.1      -----RSSDISREVENN----------------DSA-------------A----------      359
XP_030361086.1      -----QSSDISRQVENN----------------DST-------------A----------      440
KFM00668.1          -----QSSDISRQVDNN----------------DST-------------A----------      371
KAF1479074.1        -----QSSDISRQVDNN----------------DST-------------A----------      351
KAF1651161.1        -----QSSDISRQVDNN----------------DST-------------A----------      352
KAF1673648.1        -----QSSDISRQVDNN----------------DST-------------A----------      352
KAF1493319.1        -----QSSDISRQVDNN----------------DST-------------A----------      352
KAF1584157.1        -----QSLDISRQVDNN----------------DST-------------A----------      352
KAF1571723.1        -----QSSDISRQVDNN----------------DST-------------A----------      352
KAF1533169.1        -----QSSDISRQVDNN----------------DST-------------A----------      352
KAF1638955.1        -----QSSDISRQVDNN----------------DST-------------A----------      352
KAF1549972.1        -----QSSDISRQVDNN----------------DST-------------A----------      352
KAF1606914.1        -----QSSDISRQVDNN----------------DST-------------A----------      352
KAF1510957.1        -----QSSDISRQVDNN----------------DST-------------A----------      352
KAF1498899.1        -----QSSDISRQVDNN----------------DST-------------A----------      352
KAF1411525.1        -----QSSDISRQVDNN----------------DST-------------A----------      352
KAF1429205.1        -----QSSDISRQVDNN----------------DST-------------A----------      352
XP_005334318.1      -----QATKT------N----------------NCS------------------------      373
XP_014395552.1      -----QTSKT------D----------------DCS------------------------      373
KAB0404854.1        -----QAAKT------D----------------DCS------------------------      369
XP_007494828.1      -----YSTEN------D----------------TCS------------------------      389
XP_003764254.1      -----HPTEN------D----------------SCS------------------------      389
XP_020845489.1      -----HPTEN------D----------------TCS------------------------      389
XP_027703160.1      -----HPTEN------D----------------TCS------------------------      389
XP_017523929.1      -----QATKT------D----------------DCS------------------------      381
XP_008688428.1      -----PATKT------D----------------DCS------------------------      329
XP_029812166.1      -----QATKA------D----------------DCS------------------------      398
XP_025749781.1      -----PTPKT------D----------------DCP------------------------      384
XP_004416432.1      -----PAPKT------D----------------DCP------------------------      384
XP_027436262.1      -----PAPKT------D----------------DCP------------------------      384
XP_032284025.1      -----PATKT------D----------------DCP------------------------      384
XP_006735421.1      -----PDTKT------D----------------DCP------------------------      384
XP_021552166.1      -----PATKT------D----------------DCP------------------------      384
XP_026361066.1      -----PATKT------D----------------DCS------------------------      384
XP_034523598.1      -----PATKT------D----------------DCS------------------------      384
NP_001297113.1      -----PATKT------D----------------DCT------------------------      384
XP_032215488.1      -----PATKT------D----------------DCI------------------------      384
VCX31483.1          -----PATKT------D----------------DCT------------------------      384
XP_022369003.1      -----PATKT------D----------------DCT------------------------      384
XP_032694248.1      -----PATKT------D----------------DCT------------------------      384
```

```
NP_001041584.1      -----QTTKT------D----------------DCS------------------------     384
XP_025862501.1      -----QTTKT------D----------------DCS------------------------     384
KAF0873564.1        -----QAAKA------D----------------DCS------------------------     526
XP_025784751.1      -----QAAKT------D----------------DCS------------------------     378
XP_007075625.1      -----QASKT------D----------------DCS------------------------     520
XP_030189489.1      -----RAAKT------D----------------DCS------------------------     384
XP_019324655.1      -----QASKT------D----------------DCS------------------------     485
XP_023094886.1      -----RAAKT------D----------------DCS------------------------     520
XP_026892357.1      -----RAAKT------D----------------DCS------------------------     384
XP_016004457.1      -----QSTKT------D----------------DCS------------------------     379
XP_006912860.1      -----QSTKT------D----------------DCS------------------------     379
XP_011363679.1      -----QSTKT------D----------------DCS------------------------     379
XP_016076060.1      -----QAPKT------D----------------DCS------------------------     384
XP_008148371.1      -----QTSKT------D----------------DCP------------------------     384
XP_006093568.1      -----QTSKT------D----------------DCS------------------------     384
XP_006761601.1      -----QTSKT------D----------------DCS------------------------     384
XP_024426743.1      -----KATKT------D----------------DCS------------------------     379
XP_019520779.1      -----QATKT------D----------------DCS------------------------     384
XP_032976539.1      -----QATKT------N----------------ECS------------------------     384
ELW64270.1          -----QATKT------D----------------DCS------------------------     388
XP_004427367.1      -----QATKA------D----------------DCS------------------------     384
XP_008529353.1      -----QATKTLMTGS-A----------------LLP------------------------     389
NP_001288165.1      -----QATKTH----------------------DCS------------------------     384
XP_014711213.1      -----QATKTH----------------------DCS------------------------     384
XP_012514621.1      -----QATKT------D----------------DCS------------------------     384
XP_008071525.1      -----QATKT------D----------------DCS------------------------     381
XP_009005082.1      -----HATKT------A----------------DCS------------------------     384
XP_010332832.1      -----QATKI------A----------------DCS------------------------     379
XP_012326058.1      -----QATKT------A----------------DCS------------------------     390
XP_017378796.1      -----QATKT------A----------------DCS------------------------     413
XP_032141374.1      -----QATKT------A----------------DCS------------------------     413
XP_032024018.1      -----QATKT------D----------------DCS------------------------     379
XP_018891497.2      -----QATKT------D----------------DCS------------------------     385
XP_030684894.1      -----QATKT------D----------------DCS------------------------     379
PNJ75904.1          -----QATKT------D----------------DCS------------------------     385
AAP35868.1          -----QATKT------D----------------DCS------------------------     385
XP_001149570.1      -----QATKT------D----------------DCS------------------------     385
XP_003805532.1      -----QATKT------D----------------DCS------------------------     385
XP_023063823.1      -----QATKT------D----------------DCS------------------------     383
XP_033060210.1      -----QATKT------D----------------DCS------------------------     383
XP_010370669.1      -----QATKT------D----------------DCS------------------------     383
XP_017738496.1      -----QATKT------D----------------DCS------------------------     383
XP_011782168.1      -----QATKT------D----------------DCS------------------------     384
EHH23440.1          -----QATKT------D----------------DCS------------------------     411
XP_003910778.3      -----QATKT------D----------------DCS------------------------     384
XP_025212649.1      -----QATKT------D----------------DCS------------------------     390
XP_008019132.1      -----QATKT------D----------------DCS------------------------     384
XP_011832421.1      -----QATKT------D----------------DCS------------------------     369
XP_011759618.1      -----QATKT------D----------------DCS------------------------     384
XP_005579749.1      -----QATKT------D----------------DCS------------------------     384
XP_011921684.1      -----QATKT------D----------------DCS------------------------     176
XP_008259391.1      -----QPTKA------D----------------DCS------------------------     384
XP_012782438.1      -----QATKT------D----------------DCS------------------------     380
VTJ86076.1          -----QATKT------N----------------NCS------------------------     390
XP_015345533.1      -----QATKT------N----------------NCS------------------------     390
XP_027786432.1      -----QATKT------N----------------NCS------------------------     390
XP_026248345.1      -----QATKT------N----------------NCS------------------------     384
XP_005378209.1      -----QAPKT------D----------------DLS------------------------     385
XP_004466010.1      -----QATKT------E----------------DWP------------------------     384
XP_020035707.1      -----QATKT------D----------------DCS------------------------     378
XP_006181802.1      -----QAAKM------D----------------DCS------------------------     384
XP_006207862.1      -----QAAKM------D----------------DCS------------------------     384
XP_005891968.1      -----QAAKT------D----------------DCS------------------------     381
XP_010828662.1      -----QAAKT------D----------------DCS------------------------     381
XP_019830126.1      -----QAAKT------D----------------DCS------------------------     380
XP_017914486.1      -----QATKT------D----------------DCS------------------------     380
XP_006060266.2      -----QAAKT------D----------------DCS------------------------     385
XP_020728447.1      -----QAAKT------D----------------DCS------------------------     385
KAF4008892.1        -----QAAKT------D----------------GCS------------------------     385
KAB0348059.1        -----QAAKT------D----------------DCS------------------------     368
KAB0371113.1        -----QAAKT------D----------------DCS------------------------     361
MBV96963.1          -----QAAKT------D----------------DCS------------------------     384
XP_007172048.1      -----QAAKT------D----------------DCS------------------------     384
XP_007452270.1      -----QATKT------D----------------DCS------------------------     384
XP_023987108.1      -----QATKT------D----------------DCS------------------------     379
XP_004273432.1      -----QAAKA------D----------------ACS------------------------     379
XP_004328646.1      -----QAPKT------D----------------ACS------------------------     379
```

```
XP_026937670.1      -----QAPKT------D----------------ACS------------------------     379
XP_030691031.1      -----QAPKT------D----------------AYS------------------------     379
XP_022414870.1      -----QAAKT------D----------------DCS------------------------     384
XP_029064651.1      -----QAAKT------D----------------DCS------------------------     384
XP_024607566.1      -----QAAKT------D----------------DRS------------------------     384
XP_032496907.1      -----QAAKT------D----------------DRS------------------------     384
XP_007535559.1      -----QVPKI------E----------------DCR------------------------     377
XP_031299536.1      -----QAAKM------D----------------DCS------------------------     383
XP_004666516.1      -----KATKT------E----------------DFS------------------------     379
XP_008830963.1      -----QDTKT------D----------------DYP------------------------     384
XP_005347338.1      -----QASKA------D----------------DCS------------------------     384
XP_005069548.1      -----QANKT------D----------------DFS------------------------     384
XP_027267894.1      -----QANKT------D----------------DCS------------------------     384
OBS80992.1          -----QTNKT------D----------------DCS------------------------     372
XP_006979812.1      -----QANKT------E----------------DCS------------------------     391
XP_028720687.1      -----QVNKT------E----------------DCS------------------------     385
XP_021489005.1      -----QASKT------D----------------DCS------------------------     385
XP_031199589.1      -----QANKT------D----------------DCS------------------------     409
XP_028617944.1      -----QANKT------D----------------DCS------------------------     199
XP_034347030.1      -----QANKT------D----------------DCS------------------------     382
NP_077370.2         -----QANKT------D----------------DCS------------------------     384
XP_032766961.1      -----QANKT------D----------------DCS------------------------     384
XP_021063186.1      -----QANKT------D----------------DCS------------------------     367
NP_001093114.1      -----QANKT------D----------------DCS------------------------     384
XP_021028379.1      -----QANKT------D----------------DCS------------------------     384
XP_004689305.1      -----QAAKT------D----------------DCP------------------------     384
XP_004712871.1      -----PAAKM------D----------------EGS------------------------     384
XP_004382575.1      -----QATKI------D----------------DCS------------------------     384
XP_010596188.1      -----QATKI------D----------------DCS------------------------     384
XP_006890855.1      -----QAPNI------N----------------DCS------------------------     384
XP_006834038.1      -----QVTKI------D----------------DCS------------------------     384
XP_007934716.1      -----QATKT------D----------------DCS------------------------     384
XP_012603491.1      -----QATKP------E----------------DCS------------------------     384
XP_012663606.1      -----QASKA------D----------------DCS------------------------     379
XP_028372812.1      -----QATKS------D----------------DCS------------------------     384
XP_008589608.1      -----QSTKT------D----------------DCS------------------------     386
XP_012865077.1      -----QTPKT------D----------------DCS------------------------     384
NP_001166178.1      -----QATKT------D----------------DFSGSTL--------------------     389
XP_004636553.1      -----QAPKT------D----------------DFS------------------------     385
XP_004856670.1      -----QATKI------E----------------DCS------------------------     385
XP_010627744.1      -----QATKT------D----------------DCS------------------------     385


NP_509270.1         ------FCSKPADLV---------------------------------------------     375
VDO93178.1          ----GKLDES------------------------TM----YDSGVHEANHLIEI-NLYKL     404
PAV91580.1          PTKTGSYKAKPGSPV----------------TSLRV----PGSAGLDRDRVNSI-QMIDV     439
VDK46997.1          ------------------------------------------------------------     351
VIO86814.1          ----------------------------------------------------AVSEA---     459
VDP38785.1          KSYKGDYRKS-----------------------------------------------IID     452
KOF68401.1          ------------SSC------------------------------------HRRKGIEGL     371
CDJ96026.1          ---RGGLQQSTECDC------------ITQD------------------------NHLSY     543
EFX76216.1          -----------VD---------------VGD-----------------------------     345
KAF7403848.1        -----------SCPL------------HSKT-----------------------------     356
KAF7427032.1        -----------SCPL------------HSKT-----------------------------     371
KAE9417558.1        ---------------------------------------------------VGRFVA--V     367
KJH51504.1          ---------------------------------------------------QSRSET---     364
VDL69795.1          ----------------------------------------------------------DE     338
VDO32671.1          -----------------------------------------------------KLERLP-     380
VBB32409.1          -----------------------------------------IRNIKEAES-GSRSNSLDV     375
VDN54565.1          -----------------------------------------IRNIKETET-GSRSNSLDV     394
EGT55171.1          -----------------------------------------IRNIKDNEHSLSRANSFDA     405
RCN52111.1          -----------------------------------------IRNIKENESSTSRSNSLEI     410
RMX54856.1          ---------------------------SSPDHSLATRHNDIHSLVKRISHSKVLKGNYKS     437
XP_022781674.1      ---------------------------SSPDHTLATPPKDIQSLVKRISHSNLLNGNYKS     699
RNA37099.1          TSGLTLVDDQHQKSNDNSHYLVIESSNQSEK--------------ENVSKLKLINSSLTV     491
KAA0187152.1        --FLGDFTNNIA-----------------KDHTLKQ-ADTIHLTVEGVHEDGRTDGFVAV     647
GAU96593.1          PLQLFSYNNNTQENHHHDSDKLLGANSSFQDATLLQ-RRQMF------ASSNRMTNTISA     455
XP_009043980.1      NIET--------GNC-----------NGGRP------QNGMV---------------LS-     368
VDM43573.1          ELNG--------DSF-----------DELDTQLLQM-QETEL---------------VNV     495
XP_003140283.1      --GL--------KSR-----------NQFEDQLLWL-QETEF---------------ISV     383
VDN82010.1          --DL--------KLH-----------NKLEDQLVRL-QETEF---------------INI     408
VDO31501.1          --DL--------KLH-----------NKLEDQLVRL-QETEF---------------INI     420
VDP19591.1          ----HLFTNSPICINGNLKTTTLPMINQSMNNIDYQ----LQNTVNERTPKNAIANVIDL     492
EDO32053.1          ------------------------------------------------------------     324
TRY67230.1          ----------HE---------------------MQK----LELHGNSTSSCSLLNNVLNV     376
KAF7391312.1        ---------------------------LLM--GNRM----KELELQERSSKSLLANVLDI     389
GFG30449.1          ----------HT------------VVKRAK--GIIY----H-----DEMELWLMPRLLEG     420
```

```
KQS30083.1          ----------KR------------------------------------------------    397
EDW57583.2          ------------CS---------DTSSERK--HQIL----SDVELKERSSKSLLANVLDI    567
KNC22799.1          ------------CS---------DTSSERK--HQIL----SDVELKERSSKSLLANVLDI    377
RZF44856.1          ----------DSRK--------------SL--QMRE----LDASLKDRSSKSLLANVLDI    394
ENN76856.1          ----------PRPS---------STQADRK--TIHF----PDLEMKERSSKSLLANVLDI    405
KAF5300392.1        ----------NKPV---------PVVADRK--PLNL----QDVELKERSSKSLLANVLDI    406
CAB3239999.1        ----------RA---------------------PPP----PDLELRERSSKSLLANVLDI    383
PCG77624.1          ----------RV---------------------PPP----PDLELRERSSKSLLANVLDI    326
PZC79131.1          ----------RV---------------------PPP----PDLELRERSSKSLLANVLDI    393
KAF4083067.1        ----EL-VEISECVRA----------------------------IR--------------    316
XP_009924659.1      -----D-T--EQAMRA----------------------------MT--------------    352
XP_010123544.1      -----D-VHVELAMRA----------------------------VN--------------    353
CBN81618.1          ----GK-VSFKNCFRD----------------------------VS--------------    358
KAF3704230.1        ----GK-ANFYSCHRE----------------------------LQ--------------    355
RUS86578.1          -------------------------------------------NAD--------------    394
XP_034309618.1      ------------------------------------------------------------    368
PIK58946.1          ------------------------------------------PKLD--------------    412
VUZ42516.1          ------------SHLI--------------------------------------------    397
PAA51166.1          ----AQ-QHQPKLHMPQ-----------------------L-------------------    444
TGZ55997.1          MNNRFQ-PGCIHCGYPQ-----------------------TYGNLAPTGGPSISQMGRLT    484
VDP48851.1          LKEYSG-KSCPHMM----------------------------------------KTSIKL    406
PVD38331.1          -----D-VNFAQVRLL-------------------------DNGAE--------------    395
VDK73355.1          --------NYYEHR----------------------------------------------    379
VDD84963.1          --------NYHDHR----------------------------------------------    401
VDK42196.1          --------NYHEHR----------------------------------------------    334
KFD58288.1          --------LMPSER----------------------------------------------    413
OUC49089.1          --------VLPKL-----------------------------------------------    412
EYC26492.1          --------LSPTFR----------------------------------------------    446
VDK27218.1          ------------------------------------------------------------    384
VDM17286.1          ------------------------------------------------------------    303
VDL91846.1          -----------EIRLDS-----------------------ISRKQS--------------    413
KAE9548540.1        GENLKD-KKLPVTDISN-----------------------LPPSYE--------------    435
PDM74087.1          --------HHPLCPSS--------------------------EEKT--------------    386
XP_024504322.1      --------HHPLCPSV--------------------------IKNQ--------------    389
PIO52336.1          --------HHPFCPSA--------------------------DEKT--------------    385
TKR73865.1          --------HHPLCPSS--------------------------DLND--------------    392
RLU23395.1          VMELSD-LHHPNCKIN--------------------------RKVH--------------    417
XP_002427906.1      VLELSD-LHHPNCKIN--------------------------RKSS--------------    414
TMW47392.1          VMELSD-LHHPNCKIN--------------------------RKVN--------------    420
VDD83956.1          --------QNSDCVL---------------------------------------------    396
VEL33078.1          -------KENIECRIG--------------------------SVVK--------------    356
XP_009019088.1      -----------TSRFG--------------------------SNIK--------------    400
KAE9536378.1        -------PTESLCRFA--------------------------AA----------------    461
KDR23473.1          -------PDS--LRHH--------------------------PL----------------    395
KAF7266914.1        -----------TSDFK--------------------------ITTR--------------    418
KAF4519445.1        ------------------------------------------------------------    400
OXU25983.1          -----------ISDYP--------------------------SESK--------------    401
TGZ32403.1          -----------VSDYP--------------------------LELK--------------    407
ELT94491.1          ------------------------------------------PGGI--------------    402
VVC39575.1          -----------D--------------------------------LE--------------    498
RVE49089.1          -----------DCGSD--------------------------AGAG--------------    400
KAB0800277.1        -------------RFD--------------------------VAAS--------------    410
TDG52197.1          -----------H-RFD--------------------------LAAA--------------    398
TMW48669.1          ------------------------------------------------------------    335
XP_025896085.1      ----SPPTAFAPAAR---------------------------------------------    400
KAF2977017.1        ----TGARPGAGPPE---------------------------------------------    199
XP_009321837.1      ----TAKLNHYSCED---------------------------------------------    374
XP_009979985.1      ----TGKG----CAE---------------------------------------------    343
XP_028942374.1      ----TAKLNPCGCED---------------------------------------------    371
PKK19633.1          ----TAKLNHYSGED---------------------------------------------    437
XP_009894240.1      ----TARLNHYSCED---------------------------------------------    490
XP_032820219.1      -----NPSPFFRCEE---------------------------------------------    420
XP_029429480.1      -----AKVSHYSHED---------------------------------------------    423
XP_030077572.1      ----KSKFSHYSRED---------------------------------------------    380
XP_033774596.1      -----AKFSHYSRED---------------------------------------------    404
ETE72600.1          -----AKLNHYSCDA---------------------------------------------    390
XP_018082638.1      -----AKLAHYSTEY---------------------------------------------    605
XP_032905410.1      -----VEQIGCRNEA---------------------------------------------    386
XP_020773490.1      --------LHHSTDFSR-------------------------------------RRS---    408
XP_033833634.1      ------------TDFSR-------------------------------------RRS---    417
XP_030215795.1      --------LHHTCEMGH-------------------------------------TGLVGL    422
XP_030602980.1      --------LHHTCEIGR-------------------------------------KLS---    411
XP_004573543.1      --------LHHTCEIGR-------------------------------------KLS---    404
XP_005916159.1      --------LHHTCEIGR-------------------------------------KLS---    405
XP_013132089.1      --------LHHTCEIGR-------------------------------------KLS---    405
XP_031603488.1      --------------------------------------------------------S---    397
XP_005725536.1      --------------------------------------------------------S---    397
```

```
XP_026038075.1      --------LHHTCEIGR-------------------------------------KLS---      411
CAF96649.1          --------LHHTCEIGQ-------------------------------------RLS---      370
XP_023818570.1      --------LHHTCEMGQ-------------------------------------RHS---      401
RVE64289.1          --------LHHTCEMGQ-------------------------------------RLG---      436
XP_024121971.1      --------LHHTCEMGQ-------------------------------------RHG---      407
XP_015817559.1      --------HHHTCDIGQ-------------------------------------RLS---      391
XP_015225670.1      --------LHHTCEIGQ-------------------------------------RLS---      311
XP_012722063.1      --------LHHTCEIGQ-------------------------------------RLS---      405
XP_032431307.1      --------LHHTCEIGQ-------------------------------------RLS---      405
XP_014328329.1      --------LHHTCEIGQ-------------------------------------RLS---      405
XP_027886578.1      --------LHHTCEIGQ-------------------------------------RLS---      405
XP_008426791.1      --------LHHTCEIGQ-------------------------------------RLS---      405
XP_014838686.1      --------LHHTCEIGQ-------------------------------------RLS---      405
XP_014885967.1      --------LHHTCEIGQ-------------------------------------RLS---      405
XP_033954312.1      --------LHHTCELGR-------------------------------------RRS---      405
XP_034089244.1      --------LHHTCE----------------------------------------------      399
XP_010780064.1      ------------------------------------------------------------      330
XP_033970261.1      --------LHHTCELGR-------------------------------------RRS---      421
XP_029375575.1      -----LH---HTCEISQ-------------------------------------RLS---      442
KAF0023022.1        ------HRHQHTCELGQ-------------------------------------RLS---      440
XP_019952614.1      -----H-HHHHTCEIGQ-------------------------------------RLS---      396
XP_034463117.1      -----THHHHHTCEIGQ-------------------------------------RLS---      408
XP_029924656.1      -----CT-LHHTCEIGQ-------------------------------------RVS---      405
XP_028323228.1      -----CT-LHHTCDIDQ-------------------------------------RLS---      402
TNM84480.1          ------------------------------------------------------------      311
XP_029703788.1      -----CT-LHHTCEIGQ-------------------------------------RLS---      405
XP_030010368.1      -----CT-LHHTTEIRR-------------------------------------L-----      406
XP_019725056.1      -----CM-LHHTCEIGR-------------------------------------Q-----      404
XP_034034934.1      -----CT-LHHTCEIGQ-------------------------------------RLS---      394
XP_029956880.1      -----CG-LHHTCELGR-------------------------------------RLS---      405
XP_008331307.3      -----QH-LHHTCEIGQ-------------------------------------RLS---      440
XP_031724845.1      -----CP-LHHTCEIGR-------------------------------------RLS---      406
XP_013859395.1      -----CS-IHHTCEIGQ-------------------------------------RLS---      406
XP_017275335.1      -----CS-IHHTCEIGQ-------------------------------------RLS---      406
XP_020496197.1      -----CT-LHHTCEIGQ-------------------------------------RLS---      404
XP_029030400.1      -----CT-LHHTCEMGR-------------------------------------SLS---      405
XP_022060538.1      -----CS-LHHTCEIGR-------------------------------------RLS---      406
XP_023150584.1      -----CS-LHHTCELGR-------------------------------------RLS---      406
XP_029303892.1      -----CS-LHHTCEIGR-------------------------------------RVS---      429
XP_020454172.1      -----CT-LHPTCDRVR-------------------------------------RLS---      404
TKS83244.1          -----CT-LHHTCEIGR-------------------------------------RLS---      389
XP_022616583.1      -----CT-LHHTCEIGQ-------------------------------------R-----      436
XP_030293172.1      -----CT-HHHTCEIGR-------------------------------------RLS---      405
XP_027129539.1      -----CT-LHHTCEIGR-------------------------------------RIS---      402
XP_034539538.1      -----CT-VHHTCEIGR-------------------------------------RLS---      405
XP_008277330.1      -----CS-LHHTCEIGR-------------------------------------RLS---      405
XP_028276940.1      -----CT-IHHTCEIGQ-------------------------------------RHS---      405
XP_023263433.1      -----CT-LHHTCEIGQ-------------------------------------R-----      403
XP_034406336.1      -----CP-LHHTCELGR-------------------------------------RLS---      405
XP_026184460.1      -----CT-LHQNCERGR-------------------------------------RLS---      405
KAF1378228.1        -----CT-LHHTCEIGR-------------------------------------RLS---      390
XP_028450365.1      ---------------GR-------------------------------------RLS---      394
XP_031163851.1      -----CT-LHHTCEIGR-------------------------------------RLS---      405
XP_032389084.1      -----CT-LHHTCEIGR-------------------------------------RSS---      405
XP_033494682.1      -----CT-LHHTCEIGR-------------------------------------RLS---      405
XP_018544782.1      -----CT-LHHTCEIGR-------------------------------------RLS---      405
XP_026228189.1      -----CT-HHHTCEIGR-------------------------------------RLS---      405
XP_028976606.1      -----CPHHPHHHH----------------------------------------------      437
CAB1352378.1        -----C------SH----------------------------------------------      358
XP_023866849.1      -----C------NH----------------------------------------------      394
XP_013992832.1      -----C------NH----------------------------------------------      394
XP_029546688.1      -----C------NH----------------------------------------------      394
XP_021481546.1      -----C------NH----------------------------------------------      394
XP_020339889.1      -----C------NH----------------------------------------------      394
XP_029481972.1      -----C------NH----------------------------------------------      394
KPP68743.1          -----CNHR---------------------------------------------------      371
XP_023669383.1      -----CNHR---------------------------------------------------      393
KAA0720096.1        -----CNHH---------------------------------------------------      299
XP_009293684.1      -----CNHH---------------------------------------------------      393
XP_026090784.1      -----CNHH---------------------------------------------------      393
XP_018918715.1      -----CNHH---------------------------------------------------      393
KAF4098549.1        -----CNHH---------------------------------------------------      393
XP_016084173.1      -----CNHH---------------------------------------------------      393
XP_016332759.1      -----CNHH---------------------------------------------------      393
XP_016398039.1      -----CNHH---------------------------------------------------      393
XP_026989970.1      -----CNH-H--------------------------------------------------      392
TSQ12698.1          -----CNHHH--------------------------------------------------      393
```

```
XP_017347546.1      -----CNH-H--------------------------------------------------      387
XP_026794616.2      -----CNHHH--------------------------------------------------      393
XP_026861889.1      -----CN--H--------------------------------------------------      391
XP_017575347.1      -----CN--H--------------------------------------------------      391
XP_022531596.1      -----CN--H--------------------------------------------------      391
XP_012691019.2      -----CTHHH--------------------------------------------------      378
XP_028839723.1      -----CNHH---------------------------------------------------      394
XP_030638860.1      -----CNHH---------------------------------------------------      373
XP_007882964.1      ----MP--NHFESES---------------------------------------------      375
GCB70244.1          ----WW--NKRQGKQ---------------------------------------------      376
GCC26242.1          ----QL--NHFACEN---------------------------------------------      397
XP_020387034.1      ----QL--NHFACEN---------------------------------------------      388
XP_028931940.1      ----KM--NHYGKGL---------------------------------------------      394
XP_031762490.1      ----KL--AHYSTEY---------------------------------------------      390
PIO32240.1          ----KL--AHCNSEN---------------------------------------------      188
XP_018425466.1      ----KL--AHYNSEN---------------------------------------------      391
XP_006642351.1      ----KF--NHRTCEN---------------------------------------------      414
XP_028665372.1      ----KL--NHVTCEN---------------------------------------------      397
GCF49814.1          ----KL--NHY--SS---------------------------------------------      376
XP_015277816.1      ----ER--ANRLWDL---------------------------------------------      401
XP_005987340.2      ----RL--NHFTCES---------------------------------------------      381
XP_019339534.1      ----KL--NHYSGET---------------------------------------------      398
XP_025063414.1      ----KL--NHYSGEI---------------------------------------------      398
XP_019367488.1      ----KL--NHYSGET---------------------------------------------      398
XP_019412078.1      ----KL--NHYSGET---------------------------------------------      398
XP_014434314.1      ----KQ--NHYGCEN---------------------------------------------      405
XP_007060556.1      ----KL--NHYGCEN---------------------------------------------      392
XP_024072175.1      ----KL--NHYGCEN---------------------------------------------      386
XP_008170567.1      ----KL--NHYGCEN---------------------------------------------      398
XP_034610543.1      ----KL--NHYGCEN---------------------------------------------      398
XP_030394127.1      ----RL--NHYGCEN---------------------------------------------      398
XP_032651960.1      ----KL--NHYGCEN---------------------------------------------      398
XP_025020083.1      ----KL--NHYSCDS---------------------------------------------      398
XP_026536833.1      ----KL--NHYSCDA---------------------------------------------      398
XP_026564787.1      ----KL--NHYSCDA---------------------------------------------      398
XP_032084675.1      ----KL--NHYSCDA---------------------------------------------      383
XP_029139341.1      ----KL--NHYSCEA---------------------------------------------      398
XP_034281006.1      ----KL--NHYSCDA---------------------------------------------      398
XP_020649062.1      ----KL--NHYSCEN---------------------------------------------      398
XP_008117087.1      ----KL--NHYNCEN---------------------------------------------      398
XP_028564324.1      ----KL--NHYSCES---------------------------------------------      404
XP_033028155.1      ----KL--NHYSCES---------------------------------------------      403
XP_025963249.1      ----KL--DRYGCED---------------------------------------------      382
XP_009668348.1      ----KL--NHYSGED---------------------------------------------      397
XP_013798935.1      ----GL--NHYSCED---------------------------------------------      381
XP_025913685.1      ----GL--NHYSCED---------------------------------------------      406
XP_013042552.1      ----KL--NHYGCED---------------------------------------------      404
XP_005030458.2      ----KL--NHYGCED---------------------------------------------      397
XP_032057953.1      ----KL--NHYGCED---------------------------------------------      380
XP_021232050.1      ----KG----WGESS---------------------------------------------      397
OXB62403.1          ----KL--NHYGAED---------------------------------------------      398
OXB81319.1          ----KL--NHYGSED---------------------------------------------      380
XP_010722007.1      ----KL--NHYSSED---------------------------------------------      399
XP_015739349.1      ----KL--NHYSSED---------------------------------------------      399
XP_031455498.1      ----KL--NHYGSED---------------------------------------------      400
POI27435.1          ----KL--NHYGSED---------------------------------------------      375
XP_004948120.1      ----KL--NHYGSED---------------------------------------------      400
XP_032851190.1      ----KL--NHYGCED---------------------------------------------      395
XP_010007255.1      ----KL--NPYSCED---------------------------------------------      397
XP_030320702.1      ----ED--CP--------------------------------------------------      363
XP_010191940.1      ----KL--NHYGCED---------------------------------------------      395
XP_027737112.1      ----KL--TPYGCED---------------------------------------------      393
XP_027555032.1      ----KL--TPYGCED---------------------------------------------      398
XP_032565370.1      ----KL--TPYGCED---------------------------------------------      444
XP_027511217.1      ----KL--TPYGCED---------------------------------------------      543
XP_027593499.1      ----KL--TPYGCED---------------------------------------------      494
XP_017664924.1      ----KL--TPYGCED---------------------------------------------      395
XP_029817938.1      ----KL--TPYGCED---------------------------------------------      377
XP_005058641.1      ----KAKPSPYVCDD---------------------------------------------      430
XP_021385804.1      ----KG--C--TERG---------------------------------------------      395
XP_030146687.2      ----KP--TPYVCEG---------------------------------------------      442
KAF4796420.1        ----KP--TPYVCED---------------------------------------------      397
XP_032937581.1      ----KP--TPYVCED---------------------------------------------      444
XP_031989659.1      ----KL--TPYGCED---------------------------------------------      398
XP_010402086.1      ----KL--TPYGCED---------------------------------------------      397
XP_017594069.1      ----KL--TPYGCED---------------------------------------------      368
XP_014115268.1      ----KP--TPYVCED---------------------------------------------      422
```

```
XP_023797108.1      ----KP--TPYACED---------------------------------------------      368
XP_033375614.1      ----KP--TPYVCED---------------------------------------------      368
XP_014740121.1      ----KL--TPYVCED---------------------------------------------      542
RLV83430.1          ----KP--TPYVCEG---------------------------------------------      388
XP_009096098.2      ----KP-AAPYVCED---------------------------------------------      504
TRZ15870.1          ----KL--TSYVCKD---------------------------------------------      398
RMB91935.1          ----KL--TPYVCED---------------------------------------------      368
XP_030820843.1      ----KP--TPYVCED---------------------------------------------      444
XP_014165179.1      ----KP--TPYVCED---------------------------------------------      446
XP_026653582.1      ----KP--TPYVCED---------------------------------------------      328
PKU35975.1          ----KL--NQYGCED---------------------------------------------      399
XP_014805072.1      ----KL--NQYSCED---------------------------------------------      393
XP_009818330.1      ------------------------------------------------------------      156
OPJ68307.1          ----KL--NHYSGED---------------------------------------------      396
XP_008936289.1      ----KM--NPYSCED---------------------------------------------      398
XP_010287046.1      ----KL--NHYGCED---------------------------------------------      328
XP_005240140.2      ----KL--NNYSCED---------------------------------------------      457
XP_005437752.2      ----KL--NNYSCED---------------------------------------------      457
KFV74811.1          ----KL--NPYSCED---------------------------------------------      362
XP_010018389.1      ----KL--NHYGCED---------------------------------------------      405
KQK78711.1          ----KL--NHYGCED---------------------------------------------      397
XP_009570162.1      ----KL--NNYSCED---------------------------------------------      396
KFP11268.1          ----KL--NHYGCED---------------------------------------------      360
KFQ98910.1          ----KL--NHYGCED---------------------------------------------      379
XP_012985202.3      ----KL--NHYGCED---------------------------------------------      397
XP_010573388.1      ----KL--SHHGCED---------------------------------------------      396
XP_029879496.1      ----KL--SHHGCED---------------------------------------------      368
XP_030361086.1      ----KL--NHYGCED---------------------------------------------      449
KFM00668.1          ----KL--NHYGCED---------------------------------------------      380
KAF1479074.1        ----KL--NHYGCED---------------------------------------------      360
KAF1651161.1        ----KL--NHYGCED---------------------------------------------      361
KAF1673648.1        ----KL--NHYGCED---------------------------------------------      361
KAF1493319.1        ----KL--NHYGCED---------------------------------------------      361
KAF1584157.1        ----KL--NHYGCED---------------------------------------------      361
KAF1571723.1        ----KL--NHYGCED---------------------------------------------      361
KAF1533169.1        ----KL--NHYGCED---------------------------------------------      361
KAF1638955.1        ----KL--NHYGCED---------------------------------------------      361
KAF1549972.1        ----KL--NHYGCED---------------------------------------------      361
KAF1606914.1        ----KL--NHYGCED---------------------------------------------      361
KAF1510957.1        ----KL--NHYGCED---------------------------------------------      361
KAF1498899.1        ----KL--NHYGCED---------------------------------------------      361
KAF1411525.1        ----KL--NHYGCED---------------------------------------------      361
KAF1429205.1        ----KL--NHYGCED---------------------------------------------      361
XP_005334318.1      -------AMGNYCSH---------------------------------------------      381
XP_014395552.1      -------DMGNHCGH---------------------------------------------      381
KAB0404854.1        -------GPDGARSP---------------------------------------------      377
XP_007494828.1      -------ATINHCSR---------------------------------------------      397
XP_003764254.1      -------ATINHCSR---------------------------------------------      397
XP_020845489.1      -------ATINHCSR---------------------------------------------      397
XP_027703160.1      -------ATINHCSR---------------------------------------------      397
XP_017523929.1      -------DMGNRCSP---------------------------------------------      389
XP_008688428.1      -------DMGNHCSH---------------------------------------------      337
XP_029812166.1      -------DVGNHCSH---------------------------------------------      406
XP_025749781.1      -------DVGSPCNH---------------------------------------------      392
XP_004416432.1      -------DMGSPCNH---------------------------------------------      392
XP_027436262.1      -------DMGSPCNH---------------------------------------------      392
XP_032284025.1      -------DMGNPCNH---------------------------------------------      392
XP_006735421.1      -------DMGNPCNH---------------------------------------------      392
XP_021552166.1      -------DMGNPCNH---------------------------------------------      392
XP_026361066.1      -------DMGNHCSH---------------------------------------------      392
XP_034523598.1      -------DMGNHCSH---------------------------------------------      392
NP_001297113.1      -------DVGNHCSH---------------------------------------------      392
XP_032215488.1      -------DVGNHCSH---------------------------------------------      392
VCX31483.1          -------DVGNHCSH---------------------------------------------      392
XP_022369003.1      -------D-GNHCSH---------------------------------------------      391
XP_032694248.1      -------D-RNHCSH---------------------------------------------      391
NP_001041584.1      -------DMGNHCNH---------------------------------------------      392
XP_025862501.1      -------DMGNHCNH---------------------------------------------      392
KAF0873564.1        -------DMGNHCSH---------------------------------------------      534
XP_025784751.1      -------DVGNHCSH---------------------------------------------      386
XP_007075625.1      -------DVGNHCSH---------------------------------------------      528
XP_030189489.1      -------DVGNHCSH---------------------------------------------      392
XP_019324655.1      -------DVGNHCSH---------------------------------------------      493
XP_023094886.1      -------DVGNHCSH---------------------------------------------      528
XP_026892357.1      -------DVGNHCSH---------------------------------------------      392
XP_016004457.1      -------DMGNHCSH---------------------------------------------      387
XP_006912860.1      -------DMENHCSH---------------------------------------------      387
```

```
XP_011363679.1      -------DMENHCSH---------------------------------------------      387
XP_016076060.1      -------DMGNHCGH---------------------------------------------      392
XP_008148371.1      -------DMGNHCGH---------------------------------------------      392
XP_006093568.1      -------DMGNHCGH---------------------------------------------      392
XP_006761601.1      -------DMGNHCGH---------------------------------------------      392
XP_024426743.1      -------DMGNHCSH---------------------------------------------      387
XP_019520779.1      -------DMGNHCSH---------------------------------------------      392
XP_032976539.1      -------DMGNHCSH---------------------------------------------      392
ELW64270.1          -------AMGNHCDH---------------------------------------------      396
XP_004427367.1      -------DMGNHCSH---------------------------------------------      392
XP_008529353.1      -------EVGNHCSH---------------------------------------------      397
NP_001288165.1      -------EVGNHCSH---------------------------------------------      392
XP_014711213.1      -------EVGNHCSH---------------------------------------------      392
XP_012514621.1      -------AMGNHCGH---------------------------------------------      392
XP_008071525.1      -------AMGNHCSH---------------------------------------------      389
XP_009005082.1      -------AMGNHCSH---------------------------------------------      392
XP_010332832.1      -------AMGNHCSH---------------------------------------------      387
XP_012326058.1      -------AMGNHCSY---------------------------------------------      398
XP_017378796.1      -------AMGNHCSH---------------------------------------------      421
XP_032141374.1      -------AMGNHCSH---------------------------------------------      421
XP_032024018.1      -------AMRNHCSH---------------------------------------------      387
XP_018891497.2      -------AMGNHCSH---------------------------------------------      393
XP_030684894.1      -------AMRNHCSH---------------------------------------------      387
PNJ75904.1          -------AMGNHCSH---------------------------------------------      393
AAP35868.1          -------AMGNHCSH---------------------------------------------      393
XP_001149570.1      -------AMGNHCSH---------------------------------------------      393
XP_003805532.1      -------AMGNHCSH---------------------------------------------      393
XP_023063823.1      -------AMRNHCNH---------------------------------------------      391
XP_033060210.1      -------AMRNHCNH---------------------------------------------      391
XP_010370669.1      -------AMRNHCNH---------------------------------------------      391
XP_017738496.1      -------AMRNHCNH---------------------------------------------      391
XP_011782168.1      -------AMRNHCNH---------------------------------------------      392
EHH23440.1          -------AMRNHCNH---------------------------------------------      419
XP_003910778.3      -------AIRNHCNH---------------------------------------------      392
XP_025212649.1      -------AMRNHCNH---------------------------------------------      398
XP_008019132.1      -------AMRNHCNH---------------------------------------------      392
XP_011832421.1      -------AMRNHCNH---------------------------------------------      377
XP_011759618.1      -------AMRNHCNH---------------------------------------------      392
XP_005579749.1      -------AMRNHCNH---------------------------------------------      392
XP_011921684.1      -------AMRNHCNH---------------------------------------------      184
XP_008259391.1      -------AMGNHCNH---------------------------------------------      392
XP_012782438.1      -------AMGNHCSH---------------------------------------------      388
VTJ86076.1          -------AMGNHCSH---------------------------------------------      398
XP_015345533.1      -------AMGNHCSH---------------------------------------------      398
XP_027786432.1      -------AMGNHCSH---------------------------------------------      398
XP_026248345.1      -------AMGNHCSH---------------------------------------------      392
XP_005378209.1      -------AMGNHCSP---------------------------------------------      393
XP_004466010.1      -------AMGNHCSH---------------------------------------------      392
XP_020035707.1      -------AMGNHCSH---------------------------------------------      386
XP_006181802.1      -------DMGNHCSH---------------------------------------------      392
XP_006207862.1      -------DIGNHCSH---------------------------------------------      392
XP_005891968.1      -------DMGNHYSH---------------------------------------------      389
XP_010828662.1      -------DMGNHYSH---------------------------------------------      389
XP_019830126.1      -------DMGNHYSH---------------------------------------------      388
XP_017914486.1      -------DMGNHCSH---------------------------------------------      388
XP_006060266.2      -------DMGNHCSH---------------------------------------------      393
XP_020728447.1      -------DMGNHCSH---------------------------------------------      393
KAF4008892.1        -------DMGNHCSH---------------------------------------------      393
KAB0348059.1        -------DMGNHCSH---------------------------------------------      376
KAB0371113.1        -------DMGNHCSH---------------------------------------------      369
MBV96963.1          -------DMTNHCNH---------------------------------------------      392
XP_007172048.1      -------DMTNHCNH---------------------------------------------      392
XP_007452270.1      -------DMANHCSH---------------------------------------------      392
XP_023987108.1      -------DMANHCSH---------------------------------------------      387
XP_004273432.1      -------DMANHCSH---------------------------------------------      387
XP_004328646.1      -------DMANHCSH---------------------------------------------      387
XP_026937670.1      -------DMANHCSH---------------------------------------------      387
XP_030691031.1      -------DMANHCSH---------------------------------------------      387
XP_022414870.1      -------DMANHCSH---------------------------------------------      392
XP_029064651.1      -------DMANHCSH---------------------------------------------      392
XP_024607566.1      -------DVANHCSH---------------------------------------------      392
XP_032496907.1      -------DVANHCSH---------------------------------------------      392
XP_007535559.1      -------DLGNHCCP---------------------------------------------      385
XP_031299536.1      -------DMGNHCSH---------------------------------------------      391
XP_004666516.1      -------AMGNHCSH---------------------------------------------      387
XP_008830963.1      -------AMGNHCSH---------------------------------------------      392
XP_005347338.1      -------AMGNHVSH---------------------------------------------      392
```

```
XP_005069548.1      -------VMGNHFSH---------------------------------------------      392
XP_027267894.1      -------AMGNHFSH---------------------------------------------      392
OBS80992.1          -------AIGNHVSH---------------------------------------------      380
XP_006979812.1      -------AMGNHFSH---------------------------------------------      399
XP_028720687.1      -------AMGNHFSH---------------------------------------------      393
XP_021489005.1      -------DMGNHCSH---------------------------------------------      393
XP_031199589.1      -------AMGNHCSH---------------------------------------------      417
XP_028617944.1      -------AMGNHCSH---------------------------------------------      207
XP_034347030.1      -------AMGNHCSH---------------------------------------------      390
NP_077370.2         -------AMGNHCSH---------------------------------------------      392
XP_032766961.1      -------AMGNHCSH---------------------------------------------      392
XP_021063186.1      -------AMGNHCSH---------------------------------------------      375
NP_001093114.1      -------AMGNHCSH---------------------------------------------      392
XP_021028379.1      -------AMGNHCSH---------------------------------------------      392
XP_004689305.1      -------GTGNHCSH---------------------------------------------      392
XP_004712871.1      -------AVGNHCSS---------------------------------------------      392
XP_004382575.1      -------AMGNHCSH---------------------------------------------      392
XP_010596188.1      -------AMGNHCSP---------------------------------------------      392
XP_006890855.1      -------AMGNHCSH---------------------------------------------      392
XP_006834038.1      -------AMGNHCNH---------------------------------------------      392
XP_007934716.1      -------AVGNHCSH---------------------------------------------      392
XP_012603491.1      -------AMGNHCGH---------------------------------------------      392
XP_012663606.1      -------ATENHCGH---------------------------------------------      387
XP_028372812.1      -------DMGNHCSH---------------------------------------------      392
XP_008589608.1      -------AMGNHCSH---------------------------------------------      394
XP_012865077.1      -------VLGNHCNH---------------------------------------------      392
NP_001166178.1      -----LPAMGNHCGP---------------------------------------------      399
XP_004636553.1      -------VMGNHCSS---------------------------------------------      393
XP_004856670.1      -------AMGNHCSH---------------------------------------------      393
XP_010627744.1      -------AMGNHCSH---------------------------------------------      393


NP_509270.1         ------------------------------------------------------------      375
VDO93178.1          ----KENSKPRNVQNP--------------------------------------------      416
PAV91580.1          -------STPGSPSSP--------------------------------------------      448
VDK46997.1          ------------------------------------------------------------      351
VIO86814.1          ------------------------------------------------------------      459
VDP38785.1          ERDLNEHERLHNSNDP--------ITDSNNNNNT--------------------------      479
KOF68401.1          A----------------YHNSSF-----------------V-------------------      379
CDJ96026.1          VEAIPQLSSNGTNG----------------------------------------------      557
EFX76216.1          --SLKSD-----------------------------------------------------      350
KAF7403848.1        --DLPQQQ---MTS----------------------------------------------      365
KAF7427032.1        --DLPQQQ---MTS----------------------------------------------      380
KAE9417558.1        ERTKPDFF----------------F-----------------------------------      376
KJH51504.1          -MTAFDYV----------------KSSTL-------------------------------      376
VDL69795.1          E-KEK-------------------------------------------------------      342
VDO32671.1          ------------------------------------------------------------      380
VBB32409.1          DCRVCQYM----------------SGISN-------------------------------      388
VDN54565.1          ERKVYQYM----------------RGISK-------------------------------      407
EGT55171.1          DCRLNQYI----------------MTQSV-------------------------------      418
RCN52111.1          ERRLHFYM----------------SSSGL-------------------------------      423
RMX54856.1          GPGLREQESIPRMA--------------------------V-------------------      452
XP_022781674.1      GTGPREQESISKMT--------------------------I-------------------      714
RNA37099.1          DRKSKNVF----------------DCQCGNQNNKAN--NFK-------------RLKLKK      520
KAA0187152.1        DASSVED------------------LNY--------------------------------      657
GAU96593.1          DEAT-----------------------T--------------------------------      460
XP_009043980.1      ------------------------------------------------------------      368
VDM43573.1          DDGA-----------------------A--------------------------------      500
XP_003140283.1      DRYL-----------------------T--------------------------------      388
VDN82010.1          DRHL-----------------------T--------------------------------      413
VDO31501.1          DRHL-----------------------T--------------------------------      425
VDP19591.1          DDDFRATARFGNTTTTTYTTHTTTHSNTTNNTNNSN--IFINMNEKQTILNNNTKLKMNT      550
EDO32053.1          ------------------------------------------------------------      324
TRY67230.1          DDHLLRKCSTGVHE----------------------------------------------      390
KAF7391312.1        DDDFRHGNSAANPASGYISRS-------A-----------YGTPL---------------      416
GFG30449.1          KPSLDSK--HGGVS---PHINNDVKT--L-----------LNWHLHEL------------      450
KQS30083.1          ------------------------------------------------------------      397
EDW57583.2          DDDFRHNCRPMTPGGTLPHNPAFYRT--V-----------YGQGD-DG------------      601
KNC22799.1          DDDFR-NVRPMTPGGTLPHNPAFYRT--V-----------YGQGD-DG------------      410
RZF44856.1          DDDFRHGGTLQSSS------HSF-------------------------------------      411
ENN76856.1          DDDFRHNYRSGGTPTP-LPAT-FYRT--V-----------F-------------------      431
KAF5300392.1        DDDFRHNHRGGGTPTP-LPSAAFFRT--V-----------YRQNE-DN------------      439
CAB3239999.1        DDDFRHAQSQQ------PPCCRYYR-----------------------------------      402
PCG77624.1          DDDFRHPQAQQ------PQCCRYYR-----------------------------------      345
PZC79131.1          DDDFRHPQAQQ------PQCCRYYRS--L-----------DDLHE-HY------------      421
KAF4083067.1        ------A-----------------------HLTML-------------------------      322
XP_009924659.1      ------KQTTK--------------HLAKGGAQRL-NL----------------------      369
```

```
XP_010123544.1      ------TQTMK--------------HLTKEGTQPL-KL----------------------     370
CBN81618.1          ------DVSP--------------------GVTPSELL----------------------     370
KAF3704230.1        ------KWIPSAC-----------VCAVSAAETPCEPL----------------------     376
RUS86578.1          ------------------------------------------------------------     394
XP_034309618.1      ------------------------------------------------------------     368
PIK58946.1          --------------------------------SAK-------------------------     415
VUZ42516.1          -------QTSRHC-----------YCQQQQSQQQAN------------------------     415
PAA51166.1          --KYNKFDSR-------------------RPSNAAE------------------------     459
TGZ55997.1          GTKYPGYADSAAC-----------GCVGPEPPDGMNELI---------------------     512
VDP48851.1          NHRIPEILQQIM-----------------HNDNNMMNNYPFR------------------     430
PVD38331.1          ---------KSRG-----------TPPPPPPPGSGSVGAFG-------------------     416
VDK73355.1          ------------------------------ASGDGG------------------------     385
VDD84963.1          ------------------------------VSRDAN------------------------     407
VDK42196.1          ------------------------------VSRDSE------------------------     340
KFD58288.1          ---------TGL------------------TDRVEQ------------------------     422
OUC49089.1          ---------GKT------------------AEVAGQ------------------------     421
EYC26492.1          ---------TSF------------------ATGSAE------------------------     455
VDK27218.1          ------------------------------SSTSHD------------------------     390
VDM17286.1          -------ENES-------------------------------------------------     307
VDL91846.1          ------------------------------------------------------------     413
KAE9548540.1        ----EQMENTSFC-----------QPNGSSSSGPS-------------------------     455
PDM74087.1          ---------ELLD-----------AN----------------------------------     392
XP_024504322.1      ---------TGNV-----------VDQWPV---PA-------------------------     401
PIO52336.1          ---------ASPK-----------T-----------------------------------     390
TKR73865.1          ---------TPAI-----------E-----------------------------------     397
RLU23395.1          ---------HT----------------TSSSSGAGA----G-------------------     429
XP_002427906.1      ---------T------------------------------E-------------------     416
TMW47392.1          -------------------------------SGAEL----G-------------------     426
VDD83956.1          ------------------------------------------------------------     396
VEL33078.1          ---------RTG----------------RVPSSAH-------------------------     366
XP_009019088.1      ------------------------------------------------------------     400
KAE9536378.1        ---------TGRT-----------GCNGTAAAGAASRLM---------------------     480
KDR23473.1          ---------QRPG-----------GCNGLYSSGSGATNRFG-------------------     416
KAF7266914.1        ---------E--------------------------------------------------     419
KAF4519445.1        ------------------------------------------------------------     400
OXU25983.1          ---------G--------------------------------------------------     402
TGZ32403.1          ---------G--------------------------------------------------     408
ELT94491.1          ---------GGGG-----------ATCGMRPPGAGG------------------------     418
VVC39575.1          ---------SGVI-----------GCCEIHGP----------------------------     510
RVE49089.1          ---------GGGG-----------GGGGGA------G-----------------------     411
KAB0800277.1        ---------GGI------------GPCFGEPPFPA-M-----------------------     425
TDG52197.1          ---------GGIA-----------AHCFSDPPLPSSL-----------------------     415
TMW48669.1          ------------------------------------------------------------     335
XP_025896085.1      -------------------------------PSPCSV-----------------------     406
KAF2977017.1        -------------------------------PPEPAL-----------------------     205
XP_009321837.1      -------------------------------PRECET-----------------------     380
XP_009979985.1      -------------------------------RSWGSQ-----------------------     349
XP_028942374.1      -------------------------------PRXXXX-----------------------     377
PKK19633.1          -------------------------------PREGEA-----------------------     443
XP_009894240.1      -------------------------------PRDCET-----------------------     496
XP_032820219.1      -------------------------------KVCE-------------------------     424
XP_029429480.1      -------------------------------LKDYEK-----------------------     429
XP_030077572.1      -------------------------------SKDYEK-----------------------     386
XP_033774596.1      -------------------------------SKDYEK-----------------------     410
ETE72600.1          -------------------------------TRDYEG-----------------------     396
XP_018082638.1      -------------------------------PKDYER-----------------------     611
XP_032905410.1      -------------------------------PCNMSD-----------------------     392
XP_020773490.1      ---------------------------H--HDRDGG------------------------     415
XP_033833634.1      ---------------------------H--HDRDGG------------------------     424
XP_030215795.1      ---------DGL-GGPPTHHHHHHNHPH-QTERDGGG-----------------------     448
XP_030602980.1      ---------------------------Q-HERE---------------------------     416
XP_004573543.1      ---------------------------Q-HERE---------------------------     409
XP_005916159.1      ---------------------------Q-HERE---------------------------     410
XP_013132089.1      ---------------------------Q-HERE---------------------------     410
XP_031603488.1      ---------------------------Q-HERE---------------------------     402
XP_005725536.1      ---------------------------Q-HERE---------------------------     402
XP_026038075.1      ---------------------------Q-HERE---------------------------     416
CAF96649.1          ---------------------------Q-HERE---------------------------     375
XP_023818570.1      ---------------------------Q-QERDV--------------------------     407
RVE64289.1          ---------------------------Q-QERDV--------------------------     442
XP_024121971.1      ---------------------------Q-QERDV--------------------------     413
XP_015817559.1      ---------------------------Q-QDRDS--------------------------     397
XP_015225670.1      ---------------------------Q-PDREV--------------------------     317
XP_012722063.1      ---------------------------Q-QDREG--------------------------     411
XP_032431307.1      ---------------------------Q-QDREG--------------------------     411
XP_014328329.1      ---------------------------Q-QDREG--------------------------     411
XP_027886578.1      ---------------------------Q-QDREG--------------------------     411
```

```
XP_008426791.1      ---------------------------H-QDREG--------------------------     411
XP_014838686.1      ---------------------------Q-QDRES--------------------------     411
XP_014885967.1      ---------------------------Q-QDRES--------------------------     411
XP_033954312.1      ---------------------------H-LDREGGGS-----------------------     414
XP_034089244.1      ---------------------------------GGGS-----------------------     403
XP_010780064.1      ------------------------------------------------------------     330
XP_033970261.1      ---------------------------H-LDREGGGS-----------------------     430
XP_029375575.1      ---------------------------H-HD-REA-------------------------     448
KAF0023022.1        ---------------------------Q-HD-REG-------------------------     446
XP_019952614.1      ---------------------------Q-HD-REG-------------------------     402
XP_034463117.1      ---------------------------Q-HD-REG-------------------------     414
XP_029924656.1      ---------------------------H-HSDREG-------------------------     412
XP_028323228.1      ---------------------------Q-HD-REG-------------------------     408
TNM84480.1          ------------------------------------------------------------     311
XP_029703788.1      ---------------------------E-HE-RES-------------------------     411
XP_030010368.1      ---------------------------S-HPERNG-------------------------     413
XP_019725056.1      ------------------------------HEREA-------------------------     409
XP_034034934.1      ---------------------------Q-HE-REG-------------------------     400
XP_029956880.1      ---------------------------Q-HD-REG-------------------------     411
XP_008331307.3      ---------------------------H-HD-REA-------------------------     446
XP_031724845.1      ---------------------------H-HD-REG-------------------------     412
XP_013859395.1      ---------------------------Q-QD-REG-------------------------     412
XP_017275335.1      ---------------------------Q-QD-REG-------------------------     412
XP_020496197.1      ---------------------------H-HD-REG-------------------------     410
XP_029030400.1      ---------------------------Q-HD-REG-------------------------     411
XP_022060538.1      ---------------------------Q-QDHREA-------------------------     413
XP_023150584.1      ---------------------------Q-QDHREA-------------------------     413
XP_029303892.1      ---------------------------H-HD-RES-------------------------     435
XP_020454172.1      ---------------------------Q-HD-REG-------------------------     410
TKS83244.1          ---------------------------Q-HD-REG-------------------------     395
XP_022616583.1      -----------------------------HD-REA-------------------------     441
XP_030293172.1      ---------------------------Q-HD-REA-------------------------     411
XP_027129539.1      ---------------------------Q-HD-REG-------------------------     408
XP_034539538.1      ---------------------------Q-HD-REG-------------------------     411
XP_008277330.1      ---------------------------Q-HD-REA-------------------------     411
XP_028276940.1      ---------------------------Q-HD-REG-------------------------     411
XP_023263433.1      -----------------------------HD-REA-------------------------     408
XP_034406336.1      ---------------------------H-HD-REG-------------------------     411
XP_026184460.1      ---------------------------Q-HD-REG-------------------------     411
KAF1378228.1        ---------------------------H-HD-REG-------------------------     396
XP_028450365.1      ---------------------------H-HD-REG-------------------------     400
XP_031163851.1      ---------------------------H-HD-REG-------------------------     411
XP_032389084.1      ---------------------------H-HD-REG-------------------------     411
XP_033494682.1      ---------------------------H-HD-REG-------------------------     411
XP_018544782.1      ---------------------------Q-HD-REG-------------------------     411
XP_026228189.1      ---------------------------Q-HD-REG-------------------------     411
XP_028976606.1      ----------------HHHLHHTYENSC-GGADSMER-----------------------     457
CAB1352378.1        ----------------HHHLHHT--------GDSMER-----------------------     371
XP_023866849.1      ----------------HHHLHHTCESGR-GGGDIMER-----------------------     414
XP_013992832.1      ----------------HHHLHHTCESGR-GGGDIMER-----------------------     414
XP_029546688.1      ----------------HHHLHHTCESGR-GGGDIMER-----------------------     414
XP_021481546.1      ----------------HQHLHHTCESGR-GGGDIMER-----------------------     414
XP_020339889.1      ----------------HHHLHHTCESGR-GGGDIMER-----------------------     414
XP_029481972.1      ----------------HHHLHHTCESGR-GGGDIMER-----------------------     414
KPP68743.1          --------------------------NC-DSTKDSDR-----------------------     381
XP_023669383.1      --------------------------NC-DGGKDSER-----------------------     403
KAA0720096.1        --------------------------SC-EGSRDTGR-----------------------     309
XP_009293684.1      --------------------------SC-EGSRDSGR-----------------------     403
XP_026090784.1      --------------------------SC-EGPKDPER-----------------------     403
XP_018918715.1      --------------------------SS-EGSRDSGR-----------------------     403
KAF4098549.1        --------------------------SC-EGSRDSGR-----------------------     403
XP_016084173.1      --------------------------SC-EGSRDSGR-----------------------     403
XP_016332759.1      --------------------------SC-EGSRDSGR-----------------------     403
XP_016398039.1      --------------------------SC-EGSRDSGR-----------------------     403
XP_026989970.1      --------------------------HC-DSSRDIDR-----------------------     402
TSQ12698.1          --------------------------HC-DSTRDMDR-----------------------     403
XP_017347546.1      --------------------------HC-DSSRDMDR-----------------------     397
XP_026794616.2      --------------------------HC-DSSRDMDR-----------------------     403
XP_026861889.1      --------------------------HC-ETSRDMER-----------------------     401
XP_017575347.1      --------------------------HC-ESSRDMER-----------------------     401
XP_022531596.1      --------------------------HC-ETSRDMER-----------------------     401
XP_012691019.2      --------------------------TC-DVSRDHGR-----------------------     388
XP_028839723.1      --------------------------SC-ESSRDTDR-----------------------     404
XP_030638860.1      --------------------------SC-EASRDSER-----------------------     383
XP_007882964.1      ---------------------------V----AECDG-----------------------     381
GCB70244.1          ---------------------------E--------------------------------     377
GCC26242.1          ---------------------------G----TDCEK-----------------------     403
```

```
XP_020387034.1      ----------------------------G----TDCEK-----------------------      394
XP_028931940.1      ----------------------------G-TGLREGEK-----------------------      403
XP_031762490.1      ----------------------------P----KDYER-----------------------      396
PIO32240.1          ----------------------------T----KEYDR-----------------------      194
XP_018425466.1      ----------------------------T----KEYDR-----------------------      397
XP_006642351.1      ----------------------------T----RDCEK-----------------------      420
XP_028665372.1      ----------------------------S----KDCEK-----------------------      403
GCF49814.1          ----------------------------D------CD------------------------      379
XP_015277816.1      ----------------------------K------FN------------------------      404
XP_005987340.2      ----------------------------A----KDCEK-----------------------      387
XP_019339534.1      ----------------------------P----QEYEG-----------------------      404
XP_025063414.1      ----------------------------P----QEYEG-----------------------      404
XP_019367488.1      ----------------------------L----QEYEG-----------------------      404
XP_019412078.1      ----------------------------L----QEYEG-----------------------      404
XP_014434314.1      ----------------------------L----REFER-----------------------      411
XP_007060556.1      ----------------------------F----REYER-----------------------      398
XP_024072175.1      ----------------------------L----R---------------------------      388
XP_008170567.1      ----------------------------L----REYER-----------------------      404
XP_034610543.1      ----------------------------L----REYER-----------------------      404
XP_030394127.1      ----------------------------L----REYER-----------------------      404
XP_032651960.1      ----------------------------L----REYER-----------------------      404
XP_025020083.1      ----------------------------A----RDYEG-----------------------      404
XP_026536833.1      ----------------------------T----RDYEG-----------------------      404
XP_026564787.1      ----------------------------T----RDYEG-----------------------      404
XP_032084675.1      ----------------------------P----RDYEG-----------------------      389
XP_029139341.1      ----------------------------T----RDYEG-----------------------      404
XP_034281006.1      ----------------------------P----RDYEG-----------------------      404
XP_020649062.1      ----------------------------T----RDDEG-----------------------      404
XP_008117087.1      ----------------------------I----RDYEG-----------------------      404
XP_028564324.1      ----------------------------T----RDFEG-----------------------      410
XP_033028155.1      ----------------------------T----RDFEG-----------------------      409
XP_025963249.1      ----------------------------P----REREA-----------------------      388
XP_009668348.1      ----------------------------P----RECEV-----------------------      403
XP_013798935.1      ----------------------------L----REGEV-----------------------      387
XP_025913685.1      ----------------------------L----REGEV-----------------------      412
XP_013042552.1      ----------------------------P----REREV-----------------------      410
XP_005030458.2      ----------------------------P----REREV-----------------------      403
XP_032057953.1      ----------------------------P----REREV-----------------------      386
XP_021232050.1      ----------------------------C----RNRRE-----------------------      403
OXB62403.1          ----------------------------P----REREV-----------------------      404
OXB81319.1          ----------------------------P----REREV-----------------------      386
XP_010722007.1      ----------------------------P----REREV-----------------------      405
XP_015739349.1      ----------------------------P----REREV-----------------------      405
XP_031455498.1      ----------------------------P----REREV-----------------------      406
POI27435.1          ----------------------------P----REREV-----------------------      381
XP_004948120.1      ----------------------------P----REREV-----------------------      406
XP_032851190.1      ----------------------------A----RECEA-----------------------      401
XP_010007255.1      ----------------------------P----RESEA-----------------------      403
XP_030320702.1      ---------------------------------PTGTP-----------------------      368
XP_010191940.1      ----------------------------P----RDREV-----------------------      401
XP_027737112.1      ----------------------------P----RECGA-----------------------      399
XP_027555032.1      ----------------------------P----RECGA-----------------------      404
XP_032565370.1      ----------------------------P----RECGA-----------------------      450
XP_027511217.1      ----------------------------P----RECGA-----------------------      549
XP_027593499.1      ----------------------------P----RECGV-----------------------      500
XP_017664924.1      ----------------------------P----RECGA-----------------------      401
XP_029817938.1      ----------------------------P----RECGA-----------------------      383
XP_005058641.1      ----------------------------P----RECEA-----------------------      436
XP_021385804.1      ----------------------------P----RDCGA-----------------------      401
XP_030146687.2      ----------------------------P----WECGA-----------------------      448
KAF4796420.1        ----------------------------L----RECGA-----------------------      403
XP_032937581.1      ----------------------------P----RECGA-----------------------      450
XP_031989659.1      ----------------------------P----RECGS-----------------------      404
XP_010402086.1      ----------------------------P----RECGS-----------------------      403
XP_017594069.1      ----------------------------P----RECGS-----------------------      374
XP_014115268.1      ----------------------------P----RECGA-----------------------      428
XP_023797108.1      ----------------------------P----RECGA-----------------------      374
XP_033375614.1      ----------------------------P----RECGA-----------------------      374
XP_014740121.1      ----------------------------P----RECGA-----------------------      548
RLV83430.1          ----------------------------P----RECGA-----------------------      394
XP_009096098.2      ----------------------------P----RECGA-----------------------      510
TRZ15870.1          ----------------------------P----QECGA-----------------------      404
RMB91935.1          ----------------------------P----RECGA-----------------------      374
XP_030820843.1      ----------------------------P----RECGA-----------------------      450
XP_014165179.1      ----------------------------P----RECGA-----------------------      452
XP_026653582.1      ----------------------------P----RECGA-----------------------      334
PKU35975.1          ----------------------------P----RECET-----------------------      405
```

```
XP_014805072.1      ---------------------------P----RECEM-----------------------      399
XP_009818330.1      ------------------------------------------------------------      156
OPJ68307.1          ---------------------------P----RECGA-----------------------      402
XP_008936289.1      ---------------------------P----RECEA-----------------------      404
XP_010287046.1      ---------------------------P----RECEA-----------------------      334
XP_005240140.2      ---------------------------P----RECEA-----------------------      463
XP_005437752.2      ---------------------------P----RECEA-----------------------      463
KFV74811.1          ---------------------------P----REREA-----------------------      368
XP_010018389.1      ---------------------------P----XRXXX-----------------------      411
KQK78711.1          ---------------------------P----REYEA-----------------------      403
XP_009570162.1      ---------------------------P----REGEV-----------------------      402
KFP11268.1          ---------------------------P----RERER-----------------------      366
KFQ98910.1          ---------------------------P----RECET-----------------------      385
XP_012985202.3      ---------------------------P----RECEA-----------------------      403
XP_010573388.1      ---------------------------P----RECEA-----------------------      402
XP_029879496.1      ---------------------------P----RECEA-----------------------      374
XP_030361086.1      ---------------------------P----RECEA-----------------------      455
KFM00668.1          ---------------------------P----RECET-----------------------      386
KAF1479074.1        ---------------------------P----RECET-----------------------      366
KAF1651161.1        ---------------------------P----RECET-----------------------      367
KAF1673648.1        ---------------------------P----RECET-----------------------      367
KAF1493319.1        ---------------------------P----RECET-----------------------      367
KAF1584157.1        ---------------------------P----RECET-----------------------      367
KAF1571723.1        ---------------------------P----RECET-----------------------      367
KAF1533169.1        ---------------------------P----RECET-----------------------      367
KAF1638955.1        ---------------------------P----RECET-----------------------      367
KAF1549972.1        ---------------------------P----RECET-----------------------      367
KAF1606914.1        ---------------------------P----RECET-----------------------      367
KAF1510957.1        ---------------------------P----RECET-----------------------      367
KAF1498899.1        ---------------------------P----RECET-----------------------      367
KAF1411525.1        ---------------------------P----RECET-----------------------      367
KAF1429205.1        ---------------------------P----RECEM-----------------------      367
XP_005334318.1      ---------------------------L-GGPQDLEK-----------------------      390
XP_014395552.1      ---------------------------L-GGPRDLDK-----------------------      390
KAB0404854.1        ---------------------------E-KSARRVQA-----------------------      386
XP_007494828.1      ---------------------------L-GGHQDLEK-----------------------      406
XP_003764254.1      ---------------------------L-GGLQDLKK-----------------------      406
XP_020845489.1      ---------------------------L-GGLQDLEK-----------------------      406
XP_027703160.1      ---------------------------L-GGLQDLEK-----------------------      406
XP_017523929.1      ---------------------------R-GGPQDSEG-----------------------      398
XP_008688428.1      ---------------------------S-GGPRDLEK-----------------------      346
XP_029812166.1      ---------------------------L-GGPWDSEK-----------------------      415
XP_025749781.1      ---------------------------L-GVPRDLEK-----------------------      401
XP_004416432.1      ---------------------------L-GVPRDLER-----------------------      401
XP_027436262.1      ---------------------------L-GVPRDLEK-----------------------      401
XP_032284025.1      ---------------------------L-GGPRDLEK-----------------------      401
XP_006735421.1      ---------------------------L-GGPRDLEK-----------------------      401
XP_021552166.1      ---------------------------L-GGPRDLEK-----------------------      401
XP_026361066.1      ---------------------------S-GGPRDLEK-----------------------      401
XP_034523598.1      ---------------------------L-GGPQDLEK-----------------------      401
NP_001297113.1      ---------------------------L-GDPWDLEK-----------------------      401
XP_032215488.1      ---------------------------L-GDPWDLEK-----------------------      401
VCX31483.1          ---------------------------L-GDPWDLEK-----------------------      401
XP_022369003.1      ---------------------------L-GDPWDLEK-----------------------      400
XP_032694248.1      ---------------------------L-GDPWDLEK-----------------------      400
NP_001041584.1      ---------------------------L-GVPRDLEK-----------------------      401
XP_025862501.1      ---------------------------L-GVPRDLEK-----------------------      401
KAF0873564.1        ---------------------------L-EGPRDLEK-----------------------      543
XP_025784751.1      ---------------------------L-RGPQDLEK-----------------------      395
XP_007075625.1      ---------------------------L-RGPQDLEN-----------------------      537
XP_030189489.1      ---------------------------L-RGPQDLEK-----------------------      401
XP_019324655.1      ---------------------------L-RGPQDLEN-----------------------      502
XP_023094886.1      ---------------------------L-RGPQDLEK-----------------------      537
XP_026892357.1      ---------------------------L-RGPQDLEK-----------------------      401
XP_016004457.1      ---------------------------L-GEHGDLDK-----------------------      396
XP_006912860.1      ---------------------------L-GARGDLDK-----------------------      396
XP_011363679.1      ---------------------------L-GARGDLDK-----------------------      396
XP_016076060.1      ---------------------------L-GGPRDLDK-----------------------      401
XP_008148371.1      ---------------------------V-GGPRDLDK-----------------------      401
XP_006093568.1      ---------------------------L-GGPRDLDK-----------------------      401
XP_006761601.1      ---------------------------L-GGPRDLDK-----------------------      401
XP_024426743.1      ---------------------------L-GGSRDLDK-----------------------      396
XP_019520779.1      ---------------------------V-GGPVDLDK-----------------------      401
XP_032976539.1      ---------------------------V-GGPVDLDR-----------------------      401
ELW64270.1          ---------------------------L-GGPQDLEK-----------------------      405
XP_004427367.1      ---------------------------L-GGPQDCEK-----------------------      401
XP_008529353.1      ---------------------------L-GGPQDCEK-----------------------      406
```

```
NP_001288165.1      ---------------------------L-GGPQDCEK-----------------------     401
XP_014711213.1      ---------------------------L-GGPQDCEK-----------------------     401
XP_012514621.1      ---------------------------L-GRSLDLEK-----------------------     401
XP_008071525.1      ---------------------------L-GGPQDLEK-----------------------     398
XP_009005082.1      ---------------------------V-GGPQDLEK-----------------------     401
XP_010332832.1      ---------------------------V-GGPQDLEK-----------------------     396
XP_012326058.1      ---------------------------V-GGPQDLEK-----------------------     407
XP_017378796.1      ---------------------------V-GGPQDLEK-----------------------     430
XP_032141374.1      ---------------------------V-GGPQDLEK-----------------------     430
XP_032024018.1      ---------------------------M-GSPQDLEK-----------------------     396
XP_018891497.2      ---------------------------M-GGPQDFEK-----------------------     402
XP_030684894.1      ---------------------------M-GSPQDLEK-----------------------     396
PNJ75904.1          ---------------------------M-GGPQDLEK-----------------------     402
AAP35868.1          ---------------------------M-GGPQDFEK-----------------------     402
XP_001149570.1      ---------------------------M-GGPQDFEK-----------------------     402
XP_003805532.1      ---------------------------M-GGPQDFEK-----------------------     402
XP_023063823.1      ---------------------------M-GGPQDLEK-----------------------     400
XP_033060210.1      ---------------------------M-GGPQDLEK-----------------------     400
XP_010370669.1      ---------------------------M-GGSQDLEK-----------------------     400
XP_017738496.1      ---------------------------M-GGSQDLEK-----------------------     400
XP_011782168.1      ---------------------------M-GGPQDLEK-----------------------     401
EHH23440.1          ---------------------------M-GGPQDLEK-----------------------     428
XP_003910778.3      ---------------------------M-GGPQDLEK-----------------------     401
XP_025212649.1      ---------------------------M-GGPQDLEK-----------------------     407
XP_008019132.1      ---------------------------M-GGPQDLEK-----------------------     401
XP_011832421.1      ---------------------------M-GGPQDLEK-----------------------     386
XP_011759618.1      ---------------------------M-GGPQDLEK-----------------------     401
XP_005579749.1      ---------------------------M-GGPQDLEK-----------------------     401
XP_011921684.1      ---------------------------M-GGPQDLEK-----------------------     193
XP_008259391.1      ---------------------------P-GGPQNLEK-----------------------     401
XP_012782438.1      ---------------------------V-DGPQNSEK-----------------------     397
VTJ86076.1          ---------------------------L-GGPQDLEK-----------------------     407
XP_015345533.1      ---------------------------L-GGPQDLEK-----------------------     407
XP_027786432.1      ---------------------------L-GGPQDLEK-----------------------     407
XP_026248345.1      ---------------------------L-GGPQDLEK-----------------------     401
XP_005378209.1      ---------------------------L-GGPQDLEK-----------------------     402
XP_004466010.1      ---------------------------L-GGPCDSEK-----------------------     401
XP_020035707.1      ---------------------------L-GGPQDLEK-----------------------     395
XP_006181802.1      ---------------------------L-AGPQDLEK-----------------------     401
XP_006207862.1      ---------------------------L-GGTQDLEK-----------------------     401
XP_005891968.1      ---------------------------L-GGPRDLEK-----------------------     398
XP_010828662.1      ---------------------------L-GGPRDLEK-----------------------     398
XP_019830126.1      ---------------------------L-GGPRDLEK-----------------------     397
XP_017914486.1      ---------------------------L-GGPRDLEK-----------------------     397
XP_006060266.2      ---------------------------L-GGPRDLEK-----------------------     402
XP_020728447.1      ---------------------------L-GGPRDLEK-----------------------     402
KAF4008892.1        ---------------------------L-GGPRDLEK-----------------------     402
KAB0348059.1        ---------------------------L-GGPRDLEK-----------------------     385
KAB0371113.1        ---------------------------L-GGPRDLEK-----------------------     378
MBV96963.1          ---------------------------L-GGSRDLEK-----------------------     401
XP_007172048.1      ---------------------------L-GGSRDLEK-----------------------     401
XP_007452270.1      ---------------------------L-GASQDLEK-----------------------     401
XP_023987108.1      ---------------------------L-GGSRDLEK-----------------------     396
XP_004273432.1      ---------------------------L-GGSRDLEK-----------------------     396
XP_004328646.1      ---------------------------L-GGSRDLEK-----------------------     396
XP_026937670.1      ---------------------------L-GGSRDLEK-----------------------     396
XP_030691031.1      ---------------------------L-GGSRDLEK-----------------------     396
XP_022414870.1      ---------------------------L-GGSRDLEK-----------------------     401
XP_029064651.1      ---------------------------L-GGSRDLEK-----------------------     401
XP_024607566.1      ---------------------------L-GGSRDLEK-----------------------     401
XP_032496907.1      ---------------------------L-GGSRDLEK-----------------------     401
XP_007535559.1      ---------------------------L-VAPQNLEK-----------------------     394
XP_031299536.1      ---------------------------L-AGPQDLEK-----------------------     400
XP_004666516.1      ---------------------------V-GGPEDLEK-----------------------     396
XP_008830963.1      ---------------------------A-GGPQDLEK-----------------------     401
XP_005347338.1      ---------------------------V-GGPQDLEK-----------------------     401
XP_005069548.1      ---------------------------V-GEPQDLEK-----------------------     401
XP_027267894.1      ---------------------------A-AGPQDLEK-----------------------     401
OBS80992.1          ---------------------------V-XGPQDLEK-----------------------     389
XP_006979812.1      ---------------------------V-GGPQDLEK-----------------------     408
XP_028720687.1      ---------------------------V-GGPQDLEK-----------------------     402
XP_021489005.1      ---------------------------V-GGLQDLEK-----------------------     402
XP_031199589.1      ---------------------------V-GGPQDLEK-----------------------     426
XP_028617944.1      ---------------------------V-GGPQDLEK-----------------------     216
XP_034347030.1      ---------------------------V-GGPQDLEK-----------------------     399
NP_077370.2         ---------------------------V-GSPQDLEK-----------------------     401
XP_032766961.1      ---------------------------V-GSPQDLEK-----------------------     401
```

```
XP_021063186.1      ---------------------------V-GGPQDLEK-----------------------      384
NP_001093114.1      ---------------------------V-GGPQDLEK-----------------------      401
XP_021028379.1      ---------------------------V-GGPQDLEK-----------------------      401
XP_004689305.1      ---------------------------L-GGTRDFEK-----------------------      401
XP_004712871.1      ---------------------------L-AGPRDLEK-----------------------      401
XP_004382575.1      ---------------------------S-GGPLDLEK-----------------------      401
XP_010596188.1      ---------------------------S-GGPLDLEK-----------------------      401
XP_006890855.1      ---------------------------P-GGPQDFDK-----------------------      401
XP_006834038.1      ---------------------------L-GGPQDFEK-----------------------      401
XP_007934716.1      ---------------------------L-GGPQEFEK-----------------------      401
XP_012603491.1      ---------------------------L-GRSLNLEK-----------------------      401
XP_012663606.1      ---------------------------L-GGSLDLEK-----------------------      396
XP_028372812.1      ---------------------------L-GGPRDLDK-----------------------      401
XP_008589608.1      ---------------------------L-EEPRDLEK-----------------------      403
XP_012865077.1      ---------------------------S-EGPQDLEK-----------------------      401
NP_001166178.1      ---------------------------L-GGPQDLEK-----------------------      408
XP_004636553.1      ---------------------------F-RGPQDLEK-----------------------      402
XP_004856670.1      ---------------------------L-GGPQDLEK-----------------------      402
XP_010627744.1      ---------------------------L-GAPQDLEK-----------------------      402


NP_509270.1         ------------------------------------------------------------      375
VDO93178.1          ----------------------------------NS------------------------      418
PAV91580.1          ----------------------------------TGRTS----------------R---T      455
VDK46997.1          ------------------------------------------------------------      351
VIO86814.1          ------------------------------------------------------------      459
VDP38785.1          ---------------------------------------------D--------------      480
KOF68401.1          -----------------------SDGE----------------CCDDYNDDGISNI---E      397
CDJ96026.1          -----------------GSHSYKQFQSE---SAFLG---------RIVGE----------      578
EFX76216.1          -----------------------SNGSL---PH---------------------------      357
KAF7403848.1        -----------------PKHVKRKQDDD---SS---------------------------      378
KAF7427032.1        -----------------PKHVKRKQDDD---SS---------------------------      393
KAE9417558.1        ----------------------------PSLFLCWQKI----------------------      386
KJH51504.1          -----------------------SSDEWSVENF---LIE---------------------      389
VDL69795.1          ------------------------------QNICLESLL---------------------      351
VDO32671.1          ------------------------------------------------------------      380
VBB32409.1          -----------------------GKSPIS-TVIN-GPTL---------------------      402
VDN54565.1          -----------------------GRGTGQYTTLN-GPNP---------------------      422
EGT55171.1          -----------------------SNGLTSMASIPSTMIS---------------------      434
RCN52111.1          -----------------------MNGVSPPL---TTLQS---------------------      436
RMX54856.1          ----------------------SQVEDWYIQSSDVSLSS---------NEDHGVEQ---H      478
XP_022781674.1      ----------------------TQVEDWCIESSDASLSS---------NEYHGQ-R---H      739
RNA37099.1          N----------------------KLGGP-----------------SDFNESI---R---C      535
KAA0187152.1        --------------------------GW-----------------REYNDSNSFRR---T      671
GAU96593.1          --------------------------G-----------------------NHINRR---S      468
XP_009043980.1      ------------------------------------------------------------      368
VDM43573.1          ------------------------------------------------------------      500
XP_003140283.1      ------------------------------------------------------------      388
VDN82010.1          ------------------------------------------------------------      413
VDO31501.1          ------------------------------------------------------------      425
VDP19591.1          DLLSSVTVTNPSLTVQSPYRLYNSVGNFNSKSVPLTSTSTIEQVQDVYSDDFSDLS---K      607
EDO32053.1          ------------------------------------------------------------      324
TRY67230.1          -------------------------------------------YQG--------FK----      395
KAF7391312.1        ------------------------SGRPA----------TVEET----------------      426
GFG30449.1          -----------------------LLGRRG----------STDL-----------------      460
KQS30083.1          ------------------------------------------------------------      397
EDW57583.2          -----------------------SIGPIG----------STRMPDA--------VT----      616
KNC22799.1          -----------------------SIGPIG----------STRMPDA--------VT----      425
RZF44856.1          -----------------------LRGHH-------------EDGGG--------GV----      423
ENN76856.1          ------------------------------------------------------------      431
KAF5300392.1        -----------------------GNGGR-------------LHESL--------VS----      451
CAB3239999.1        -------------------------GGE-------------ESGAG--------LA----      412
PCG77624.1          -------------------------GGE-------------ENGAG--------LA----      355
PZC79131.1          -----------------------SPGGE-------------ENGAG--------LA----      433
KAF4083067.1        ------------------------------------------------------------      322
XP_009924659.1      -----------------------------------------------------P------      370
XP_010123544.1      -----------------------------------------------------P------      371
CBN81618.1          -----------------------------------------------------P------      371
KAF3704230.1        -----------------------------------------------------P------      377
RUS86578.1          ------------------------------------------------------------      394
XP_034309618.1      ------------------------------------------------------------      368
PIK58946.1          ------------------------------------------------------------      415
VUZ42516.1          -----------------------------------------------------------Q      416
PAA51166.1          ------------------------------------------------------------      459
TGZ55997.1          ------------------------------------------------------------      512
VDP48851.1          ------------------------------------------------------------      430
PVD38331.1          -------------------EMA--DNGFQ------------------------------Q      425
```

```
VDK73355.1          --------------------------------------------------------SLVT    389
VDD84963.1          --------------------------------------------------------KASE    411
VDK42196.1          --------------------------------------------------------CVRS    344
KFD58288.1          --------------------------------------------------------STSS    426
OUC49089.1          --------------------------------------------------------SSSS    425
EYC26492.1          --------------------------------------------------------ETSY    459
VDK27218.1          --------------------------------------------------------GSSF    394
VDM17286.1          ------------------------------------------------------------    307
VDL91846.1          ------------------------------------------------------------    413
KAE9548540.1        -----------------------------------------------------------K    456
PDM74087.1          -----------------------------------------------------K-LSPFA    398
XP_024504322.1      -------------------------------------------------IHIYE-PESID    411
PIO52336.1          ---------------------------------------------------LN--CTTEL    397
TKR73865.1          ---------------------------------------------------LS--A----    400
RLU23395.1          -------------------EGM-------------------------------------G    433
XP_002427906.1      -------------------IGG-------------------------------------G    420
TMW47392.1          -------------------IGD-------------------------------------H    430
VDD83956.1          ---------------------------------------------TDFNQHM--------    403
VEL33078.1          -------------------PLKRDRKKICSE------------------EEVMPA-----    384
XP_009019088.1      -------------------------------------------------------KLVEQ    405
KAE9536378.1        -------------------H--------VAV------------------------SSIDD    489
KDR23473.1          -------------------GLMAGFNGLPSV------------------------VGLDG    433
KAF7266914.1        -----------------------PSF-------V---------LQNVDPDDMNKMKAHSS    440
KAF4519445.1        ------------------------------------------------------------    400
OXU25983.1          -----------------------SPDGFESVTAA---------YKNIRDEEIRH-IPHA-    428
TGZ32403.1          -----------------------SPDAFESVTST---------YKSIRDDDARH-VPHA-    434
ELT94491.1          ----------------------------------------------AYIG-MHDPVIEAE    431
VVC39575.1          ----------------------------------------------IIAL-PSPPEIINQ    523
RVE49089.1          -------------------GGGAGEGGVCRACAC---------RLHDAPS-LCDAL----    438
KAB0800277.1        -------------------PLSGGDDDLYSPASC---------RKNTY-----------D    446
TDG52197.1          -------------------PLPGADDDLFSPSGL---------NGDISPG-CCPAAAAAA    446
TMW48669.1          ------------------------------------------------------------    335
XP_025896085.1      -------------------E----DAQ---------------------------------    410
KAF2977017.1        ------------------------------------------------------------    205
XP_009321837.1      -------------------V----GGARL-------------------------------    386
XP_009979985.1      -------------------S----GPMQP-------------------------------    355
XP_028942374.1      -------------------X----XXXXX-------------------------------    383
PKK19633.1          -------------------A----GGAMP-------------------------------    449
XP_009894240.1      -------------------A----GRT---------------------------------    500
XP_032820219.1      ------------------------HGGE--------------------------------    428
XP_029429480.1      ------------------------------------------------------------    429
XP_030077572.1      -------------------T----------------------------------------    387
XP_033774596.1      -------------------T----------------------------------------    411
ETE72600.1          -------------------R----TGGAT-------------------------------    402
XP_018082638.1      -------------------S----VGVV--------------------------------    616
XP_032905410.1      -------------------E----PG----------------------------------    395
XP_020773490.1      ----------------------------L-------------------------------    416
XP_033833634.1      ----------------------------L-------------------------------    425
XP_030215795.1      -------------------L----LG----------------------------------    451
XP_030602980.1      -------------------------GGLL-------------------------------    420
XP_004573543.1      -------------------------DGLL-------------------------------    413
XP_005916159.1      -------------------------GGLL-------------------------------    414
XP_013132089.1      -------------------------GGLL-------------------------------    414
XP_031603488.1      -------------------------GGLL-------------------------------    406
XP_005725536.1      -------------------------GGLL-------------------------------    406
XP_026038075.1      -------------------------DGLL-------------------------------    420
CAF96649.1          -------------------------GGLL-------------------------------    379
XP_023818570.1      --------------------------GML-------------------------------    410
RVE64289.1          --------------------------GML-------------------------------    445
XP_024121971.1      --------------------------GML-------------------------------    416
XP_015817559.1      --------------------------GML-------------------------------    400
XP_015225670.1      --------------------------GML-------------------------------    320
XP_012722063.1      --------------------------GML-------------------------------    414
XP_032431307.1      --------------------------GML-------------------------------    414
XP_014328329.1      --------------------------GML-------------------------------    414
XP_027886578.1      --------------------------GML-------------------------------    414
XP_008426791.1      --------------------------GML-------------------------------    414
XP_014838686.1      --------------------------GML-------------------------------    414
XP_014885967.1      --------------------------GML-------------------------------    414
XP_033954312.1      -------------------L----EGVLL-------------------------------    420
XP_034089244.1      -------------------L----EGVLL-------------------------------    409
XP_010780064.1      ------------------------------------------------------------    330
XP_033970261.1      -------------------L----EGVLL-------------------------------    436
XP_029375575.1      --------------------------GLL-------------------------------    451
KAF0023022.1        --------------------------GLL-------------------------------    449
XP_019952614.1      --------------------------ALL-------------------------------    405
XP_034463117.1      --------------------------ALL-------------------------------    417
```

```
XP_029924656.1      --------------------------GLL-------------------------------     415
XP_028323228.1      --------------------------GLF-------------------------------     411
TNM84480.1          ------------------------------------------------------------     311
XP_029703788.1      --------------------------GLL-------------------------------     414
XP_030010368.1      --------------------------GLL-------------------------------     416
XP_019725056.1      --------------------------TLL-------------------------------     412
XP_034034934.1      --------------------------RLL-------------------------------     403
XP_029956880.1      --------------------------GLL-------------------------------     414
XP_008331307.3      --------------------------GLL-------------------------------     449
XP_031724845.1      --------------------------RLI-------------------------------     415
XP_013859395.1      --------------------------GML-------------------------------     415
XP_017275335.1      --------------------------GML-------------------------------     415
XP_020496197.1      --------------------------GLL-------------------------------     413
XP_029030400.1      --------------------------GLL-------------------------------     414
XP_022060538.1      --------------------------GLL-------------------------------     416
XP_023150584.1      --------------------------GLL-------------------------------     416
XP_029303892.1      --------------------------GLL-------------------------------     438
XP_020454172.1      --------------------------GLL-------------------------------     413
TKS83244.1          --------------------------GLL-------------------------------     398
XP_022616583.1      --------------------------GLL-------------------------------     444
XP_030293172.1      --------------------------GLI-------------------------------     414
XP_027129539.1      --------------------------GLL-------------------------------     411
XP_034539538.1      --------------------------DLL-------------------------------     414
XP_008277330.1      --------------------------GLL-------------------------------     414
XP_028276940.1      --------------------------GLL-------------------------------     414
XP_023263433.1      --------------------------GLL-------------------------------     411
XP_034406336.1      --------------------------RLL-------------------------------     414
XP_026184460.1      --------------------------RLL-------------------------------     414
KAF1378228.1        --------------------------GLL-------------------------------     399
XP_028450365.1      --------------------------GLL-------------------------------     403
XP_031163851.1      --------------------------GLL-------------------------------     414
XP_032389084.1      --------------------------GLL-------------------------------     414
XP_033494682.1      --------------------------GLL-------------------------------     414
XP_018544782.1      --------------------------GLL-------------------------------     414
XP_026228189.1      --------------------------GLL-------------------------------     414
XP_028976606.1      -------------------G----L-GGL-------------------------------     462
CAB1352378.1        -------------------G----L-GG--------------------------------     375
XP_023866849.1      -------------------G----L-GG--------------------------------     418
XP_013992832.1      -------------------G----L-GG--------------------------------     418
XP_029546688.1      -------------------G----L-GG--------------------------------     418
XP_021481546.1      -------------------G----L-GG--------------------------------     418
XP_020339889.1      -------------------G----L-GG--------------------------------     418
XP_029481972.1      -------------------G----L-GG--------------------------------     418
KPP68743.1          -------------------G--------L-------------------------------     383
XP_023669383.1      -------------------G--------L-------------------------------     405
KAA0720096.1        -------------------N--------L-------------------------------     311
XP_009293684.1      -------------------T--------L-------------------------------     405
XP_026090784.1      -------------------N--------I-------------------------------     405
XP_018918715.1      -------------------N--------L-------------------------------     405
KAF4098549.1        -------------------N--------L-------------------------------     405
XP_016084173.1      -------------------N--------L-------------------------------     405
XP_016332759.1      -------------------N--------L-------------------------------     405
XP_016398039.1      -------------------N--------L-------------------------------     405
XP_026989970.1      -------------------N--------L-------------------------------     404
TSQ12698.1          -------------------N--------L-------------------------------     405
XP_017347546.1      -------------------N--------L-------------------------------     399
XP_026794616.2      -------------------N--------L-------------------------------     405
XP_026861889.1      -------------------G--------L-------------------------------     403
XP_017575347.1      -------------------G--------L-------------------------------     403
XP_022531596.1      -------------------G--------L-------------------------------     403
XP_012691019.2      ------------------------------------------------------------     388
XP_028839723.1      -------------------G--------L-------------------------------     406
XP_030638860.1      -------------------G----R---L-------------------------------     386
XP_007882964.1      -------------------Q----RRSVS-------------------------------     387
GCB70244.1          ------------------------------------------------------------     377
GCC26242.1          -------------------P----ISS---------------------------------     407
XP_020387034.1      -------------------P----LSS---------------------------------     398
XP_028931940.1      -------------------A----PS-ED-------------------------------     408
XP_031762490.1      -------------------S----VGVLV-------------------------------     402
PIO32240.1          -------------------S----IGAIL-------------------------------     200
XP_018425466.1      -------------------S----IGVLL-------------------------------     403
XP_006642351.1      -------------------S----IGMAP-------------------------------     426
XP_028665372.1      -------------------S----LGVTF-------------------------------     409
GCF49814.1          -------------------------GATS-------------------------------     383
XP_015277816.1      -------------------------PRGP-------------------------------     408
XP_005987340.2      -------------------P----QGVA--------------------------------     392
XP_019339534.1      -------------------A----GGTRP-------------------------------     410
```

```
XP_025063414.1      -------------------A----GGMRP-------------------------------      410
XP_019367488.1      -------------------A----GDTRP-------------------------------      410
XP_019412078.1      -------------------A----GDTRP-------------------------------      410
XP_014434314.1      -------------------A----AGARP-------------------------------      417
XP_007060556.1      -------------------T----AGARP-------------------------------      404
XP_024072175.1      -------------------V----AGARP-------------------------------      394
XP_008170567.1      -------------------V----AGARP-------------------------------      410
XP_034610543.1      -------------------V----AGARP-------------------------------      410
XP_030394127.1      -------------------V----AGARP-------------------------------      410
XP_032651960.1      -------------------V----AGARP-------------------------------      410
XP_025020083.1      -------------------R----AGGAT-------------------------------      410
XP_026536833.1      -------------------R----TGGAT-------------------------------      410
XP_026564787.1      -------------------R----TGGAT-------------------------------      410
XP_032084675.1      -------------------R----TGDAT-------------------------------      395
XP_029139341.1      -------------------R----TGAAT-------------------------------      410
XP_034281006.1      -------------------R----TGDAT-------------------------------      410
XP_020649062.1      -------------------A----ESSAM-------------------------------      410
XP_008117087.1      -------------------G----ASGAP-------------------------------      410
XP_028564324.1      -------------------G----ASGTM-------------------------------      416
XP_033028155.1      -------------------G----ASGTM-------------------------------      415
XP_025963249.1      -------------------A----AGPGP-------------------------------      394
XP_009668348.1      -------------------A----AGPRP-------------------------------      409
XP_013798935.1      -------------------A----VGTRS-------------------------------      393
XP_025913685.1      -------------------A----VGTRS-------------------------------      418
XP_013042552.1      -------------------A----GGTRP-------------------------------      416
XP_005030458.2      -------------------A----GGTRP-------------------------------      409
XP_032057953.1      -------------------A----GGTRP-------------------------------      392
XP_021232050.1      -------------------M----NPTQ--------------------------------      408
OXB62403.1          -------------------A----GGTRP-------------------------------      410
OXB81319.1          -------------------A----GGTRP-------------------------------      392
XP_010722007.1      -------------------A----GGTRP-------------------------------      411
XP_015739349.1      -------------------A----GGTRP-------------------------------      411
XP_031455498.1      -------------------A----GGTRP-------------------------------      412
POI27435.1          -------------------A----GGTRP-------------------------------      387
XP_004948120.1      -------------------A----GSTRP-------------------------------      412
XP_032851190.1      -------------------A----EGARP-------------------------------      407
XP_010007255.1      -------------------A----EGVRP-------------------------------      409
XP_030320702.1      -------------------A----PPLGP-------------------------------      374
XP_010191940.1      -------------------A----GGTMP-------------------------------      407
XP_027737112.1      -------------------V----GGTRP-------------------------------      405
XP_027555032.1      -------------------V----GGTRP-------------------------------      410
XP_032565370.1      -------------------V----GGTRP-------------------------------      456
XP_027511217.1      -------------------V----GGTRP-------------------------------      555
XP_027593499.1      -------------------V----GGTRP-------------------------------      506
XP_017664924.1      -------------------V----GGTRP-------------------------------      407
XP_029817938.1      -------------------V----GGTRP-------------------------------      389
XP_005058641.1      -------------------A----GGTRS-------------------------------      442
XP_021385804.1      -------------------V----GSTRS-------------------------------      407
XP_030146687.2      -------------------V----GSTRS-------------------------------      454
KAF4796420.1        -------------------A----GGTRS-------------------------------      409
XP_032937581.1      -------------------A----GGTRS-------------------------------      456
XP_031989659.1      -------------------V----GGTRS-------------------------------      410
XP_010402086.1      -------------------V----GGTRS-------------------------------      409
XP_017594069.1      -------------------V----GGTRS-------------------------------      380
XP_014115268.1      -------------------V----GGTRS-------------------------------      434
XP_023797108.1      -------------------V----GGTRS-------------------------------      380
XP_033375614.1      -------------------V----GGTRS-------------------------------      380
XP_014740121.1      -------------------A----GGMRS-------------------------------      554
RLV83430.1          -------------------V----GSTRS-------------------------------      400
XP_009096098.2      -------------------V----GGTRS-------------------------------      516
TRZ15870.1          -------------------V----GGTRS-------------------------------      410
RMB91935.1          -------------------V----GGTRS-------------------------------      380
XP_030820843.1      -------------------V----GGTRS-------------------------------      456
XP_014165179.1      -------------------V----GGTRS-------------------------------      458
XP_026653582.1      -------------------V----EGTRP-------------------------------      340
PKU35975.1          -------------------A----GGAGP-------------------------------      411
XP_014805072.1      -------------------A----GGAGP-------------------------------      405
XP_009818330.1      ------------------------------------------------------------      156
OPJ68307.1          -------------------A----GGTMP-------------------------------      408
XP_008936289.1      -------------------L----GGARH-------------------------------      410
XP_010287046.1      -------------------A----GGTRP-------------------------------      340
XP_005240140.2      -------------------A----GSX---------------------------------      467
XP_005437752.2      -------------------A----GSERP-------------------------------      469
KFV74811.1          -------------------T----GDTR--------------------------------      373
XP_010018389.1      -------------------X-----XXXX-------------------------------      416
KQK78711.1          -------------------V----GGMRP-------------------------------      409
XP_009570162.1      -------------------V----GSTRS-------------------------------      408
```

```
KFP11268.1          -------------------A----GGTRP-------------------------------      372
KFQ98910.1          -------------------A----GGSRP-------------------------------      391
XP_012985202.3      -------------------A----GAMRP-------------------------------      409
XP_010573388.1      -------------------A----GAVRP-------------------------------      408
XP_029879496.1      -------------------A----GAMRP-------------------------------      380
XP_030361086.1      -------------------V----RGSRP-------------------------------      461
KFM00668.1          -------------------V----GGARL-------------------------------      392
KAF1479074.1        -------------------V----GGVRL-------------------------------      372
KAF1651161.1        -------------------V----GGARL-------------------------------      373
KAF1673648.1        -------------------V----GGARL-------------------------------      373
KAF1493319.1        -------------------V----GGARL-------------------------------      373
KAF1584157.1        -------------------V----GGARL-------------------------------      373
KAF1571723.1        -------------------V----GGARL-------------------------------      373
KAF1533169.1        -------------------V----GGARL-------------------------------      373
KAF1638955.1        -------------------V----GGARL-------------------------------      373
KAF1549972.1        -------------------V----GGARL-------------------------------      373
KAF1606914.1        -------------------V----GGARL-------------------------------      373
KAF1510957.1        -------------------V----RGARL-------------------------------      373
KAF1498899.1        -------------------V----GGARL-------------------------------      373
KAF1411525.1        -------------------V----GGARL-------------------------------      373
KAF1429205.1        -------------------V----GGARL-------------------------------      373
XP_005334318.1      -------------------T----LRGRG-------------------------------      396
XP_014395552.1      -------------------T----PRGKD-------------------------------      396
KAB0404854.1        -------------------AVDTAAPGRL-------------------------------      396
XP_007494828.1      -------------------T----TEERS-------------------------------      412
XP_003764254.1      -------------------T----TPEVG-------------------------------      412
XP_020845489.1      -------------------T----TSERG-------------------------------      412
XP_027703160.1      -------------------N----TSERG-------------------------------      412
XP_017523929.1      -------------------T----PRGRG-------------------------------      404
XP_008688428.1      -------------------T----PRGRG-------------------------------      352
XP_029812166.1      -------------------T----PRGRG-------------------------------      421
XP_025749781.1      -------------------T----PRGRG-------------------------------      407
XP_004416432.1      -------------------T----PRGQG-------------------------------      407
XP_027436262.1      -------------------T----PRGQG-------------------------------      407
XP_032284025.1      -------------------T----PRGPG-------------------------------      407
XP_006735421.1      -------------------T----PRGRG-------------------------------      407
XP_021552166.1      -------------------T----PRGRG-------------------------------      407
XP_026361066.1      -------------------T----PRGRG-------------------------------      407
XP_034523598.1      -------------------T----PRGRG-------------------------------      407
NP_001297113.1      -------------------T----PRGRD-------------------------------      407
XP_032215488.1      -------------------T----PRGRD-------------------------------      407
VCX31483.1          -------------------T----PRGRD-------------------------------      407
XP_022369003.1      -------------------T----PRGRD-------------------------------      406
XP_032694248.1      -------------------T----PRGRD-------------------------------      406
NP_001041584.1      -------------------T----PRSRG-------------------------------      407
XP_025862501.1      -------------------T----PRGRG-------------------------------      407
KAF0873564.1        -------------------T----VRGRG-------------------------------      549
XP_025784751.1      -------------------T----PRGRG-------------------------------      401
XP_007075625.1      -------------------T----PRGRG-------------------------------      543
XP_030189489.1      -------------------T----PRGRG-------------------------------      407
XP_019324655.1      -------------------T----PRGRG-------------------------------      508
XP_023094886.1      -------------------T----PRGRG-------------------------------      543
XP_026892357.1      -------------------T----PRGRG-------------------------------      407
XP_016004457.1      -------------------T----PKGRS-------------------------------      402
XP_006912860.1      -------------------T----PKGRS-------------------------------      402
XP_011363679.1      -------------------T----PKGRS-------------------------------      402
XP_016076060.1      -------------------T----LRGRG-------------------------------      407
XP_008148371.1      -------------------I----PRGKD-------------------------------      407
XP_006093568.1      -------------------T----PRGKD-------------------------------      407
XP_006761601.1      -------------------T----PRGKD-------------------------------      407
XP_024426743.1      -------------------T----LRGRG-------------------------------      402
XP_019520779.1      -------------------T----PRGRG-------------------------------      407
XP_032976539.1      -------------------T----QRGRG-------------------------------      407
ELW64270.1          -------------------I----PRGRG-------------------------------      411
XP_004427367.1      -------------------T----PRGRG-------------------------------      407
XP_008529353.1      -------------------T----PRGRG-------------------------------      412
NP_001288165.1      -------------------T----PRGRG-------------------------------      407
XP_014711213.1      -------------------T----PRGRS-------------------------------      407
XP_012514621.1      -------------------T----PRGRC-------------------------------      407
XP_008071525.1      -------------------T----PRARC-------------------------------      404
XP_009005082.1      -------------------S----TRNRC-------------------------------      407
XP_010332832.1      -------------------S----QRNRC-------------------------------      402
XP_012326058.1      -------------------S----PRNRC-------------------------------      413
XP_017378796.1      -------------------S----PRNRC-------------------------------      436
XP_032141374.1      -------------------S----PRNRC-------------------------------      436
XP_032024018.1      -------------------S----PRDRC-------------------------------      402
XP_018891497.2      -------------------S----PRDRC-------------------------------      408
```

```
XP_030684894.1      -------------------S----PRDRC-------------------------------      402
PNJ75904.1          -------------------S----PRDRC-------------------------------      408
AAP35868.1          -------------------S----PRDRC-------------------------------      408
XP_001149570.1      -------------------S----PRDRC-------------------------------      408
XP_003805532.1      -------------------S----PRDRC-------------------------------      408
XP_023063823.1      -------------------N----PRGRC-------------------------------      406
XP_033060210.1      -------------------N----PRGRC-------------------------------      406
XP_010370669.1      -------------------N----PRGRC-------------------------------      406
XP_017738496.1      -------------------N----PRGRC-------------------------------      406
XP_011782168.1      -------------------S----PRGRC-------------------------------      407
EHH23440.1          -------------------N----PRGRC-------------------------------      434
XP_003910778.3      -------------------N----PRGRC-------------------------------      407
XP_025212649.1      -------------------N----PRGRC-------------------------------      413
XP_008019132.1      -------------------N----PRGRC-------------------------------      407
XP_011832421.1      -------------------N----PRGRC-------------------------------      392
XP_011759618.1      -------------------N----PRGRC-------------------------------      407
XP_005579749.1      -------------------N----PRGRC-------------------------------      407
XP_011921684.1      -------------------N----PRGRC-------------------------------      199
XP_008259391.1      -------------------T----PRGSG-------------------------------      407
XP_012782438.1      -------------------M----VRGSG-------------------------------      403
VTJ86076.1          -------------------T----LRGRG-------------------------------      413
XP_015345533.1      -------------------T----LRGRG-------------------------------      413
XP_027786432.1      -------------------T----LRGRG-------------------------------      413
XP_026248345.1      -------------------T----LRGRG-------------------------------      407
XP_005378209.1      -------------------T----PRGRG-------------------------------      408
XP_004466010.1      -------------------T----PRGRA-------------------------------      407
XP_020035707.1      -------------------A----PRGRG-------------------------------      401
XP_006181802.1      -------------------T----PRGRG-------------------------------      407
XP_006207862.1      -------------------T----PRGRG-------------------------------      407
XP_005891968.1      -------------------T----PRGRG-------------------------------      404
XP_010828662.1      -------------------T----PRGRG-------------------------------      404
XP_019830126.1      -------------------T----PRGRG-------------------------------      403
XP_017914486.1      -------------------T----PRGRG-------------------------------      403
XP_006060266.2      -------------------T----PRGTG-------------------------------      408
XP_020728447.1      -------------------T----PRGRG-------------------------------      408
KAF4008892.1        -------------------T----PRGRG-------------------------------      408
KAB0348059.1        -------------------T----PRGRG-------------------------------      391
KAB0371113.1        -------------------T----PRGRG-------------------------------      384
MBV96963.1          -------------------T----PRGSG-------------------------------      407
XP_007172048.1      -------------------T----PRGSG-------------------------------      407
XP_007452270.1      -------------------T----PRGSG-------------------------------      407
XP_023987108.1      -------------------T----PRGSG-------------------------------      402
XP_004273432.1      -------------------T----PRGSG-------------------------------      402
XP_004328646.1      -------------------T----PRGSG-------------------------------      402
XP_026937670.1      -------------------T----PRGSG-------------------------------      402
XP_030691031.1      -------------------T----PRGSG-------------------------------      402
XP_022414870.1      -------------------T----PRGSG-------------------------------      407
XP_029064651.1      -------------------T----PRGSG-------------------------------      407
XP_024607566.1      -------------------T----PRGSG-------------------------------      407
XP_032496907.1      -------------------T----PRGSG-------------------------------      407
XP_007535559.1      -------------------T----PRARG-------------------------------      400
XP_031299536.1      -------------------T----PRGRG-------------------------------      406
XP_004666516.1      -------------------T----LKGTD-------------------------------      402
XP_008830963.1      -------------------T----LRGRS-------------------------------      407
XP_005347338.1      -------------------T----SRDRG-------------------------------      407
XP_005069548.1      -------------------T----PRGRG-------------------------------      407
XP_027267894.1      -------------------T----PRGRG-------------------------------      407
OBS80992.1          -------------------T----PRGRG-------------------------------      395
XP_006979812.1      -------------------T----LRGRG-------------------------------      414
XP_028720687.1      -------------------T----LRGRG-------------------------------      408
XP_021489005.1      -------------------T----ARGRG-------------------------------      408
XP_031199589.1      -------------------T----PRGRG-------------------------------      432
XP_028617944.1      -------------------T----PRGRG-------------------------------      222
XP_034347030.1      -------------------T----PRGRG-------------------------------      405
NP_077370.2         -------------------T----SRSRD-------------------------------      407
XP_032766961.1      -------------------T----SRSRD-------------------------------      407
XP_021063186.1      -------------------T----PRGRG-------------------------------      390
NP_001093114.1      -------------------T----PRGRG-------------------------------      407
XP_021028379.1      -------------------T----PRGRG-------------------------------      407
XP_004689305.1      -------------------T----LRGRG-------------------------------      407
XP_004712871.1      -------------------T----PRGLG-------------------------------      407
XP_004382575.1      -------------------I----PWGRG-------------------------------      407
XP_010596188.1      -------------------I----PWGRG-------------------------------      407
XP_006890855.1      -------------------S----PRDRG-------------------------------      407
XP_006834038.1      -------------------N----SRGRG-------------------------------      407
XP_007934716.1      -------------------T----PRDRG-------------------------------      407
XP_012603491.1      -------------------T----PRGRC-------------------------------      407
```

```
XP_012663606.1      -------------------N----PRGKC-------------------------------      402
XP_028372812.1      -------------------T----LRGRG-------------------------------      407
XP_008589608.1      -------------------T----LRGRG-------------------------------      409
XP_012865077.1      -------------------S----MRGRG-------------------------------      407
NP_001166178.1      -------------------T----SRGRG-------------------------------      414
XP_004636553.1      -------------------T----PRDRG-------------------------------      408
XP_004856670.1      -------------------T----PRGRG-------------------------------      408
XP_010627744.1      -------------------T----TRGRS-------------------------------      408


NP_509270.1         -----------------------------QELRFCMEEI--KRYLDE----QDSTEKN--      398
VDO93178.1          --------------------VDSSVMEQLHEIRRRIRHL--CESVQD----AEEERKL--      450
PAV91580.1          ASYSKVPT--------LWEGTMSALAENNQQLRRTSQVF--SKEVDE----NRKKRQC--      499
VDK46997.1          ------------------------------------------------------------      351
VIO86814.1          --VADAPP--------------PINTQRIELLVNLMRQF--IQLKEE----AQRKHCL--      495
VDP38785.1          ---LYNP----------------------------------TIVTNE----EYSKEVN--      497
KOF68401.1          KNDKP-------------------NTKEHDGLIETLELFLQYLTTHD----GYKESTV--      432
CDJ96026.1          ---QILPRIS-VTRSVMLSEFDHRFRRILKRIYRSLQQH--EIREEI----IDERQRI--      626
EFX76216.1          --------RV-PSNIGPVDNFERQFLRVLNKVYQHIERS--EARLAD----QDRKDVI--      400
KAF7403848.1        --------SL-NLHFGKDTNLETQWTRILGRVHATIESN--ERRLAE----QDKRERT--      421
KAF7427032.1        --------SP-NLHFGKDTNLETQWTRILGRVHATIENN--ERRLAE----QDKRERT--      436
KAE9417558.1        ---------------------KGKTLVGIDIFKKRLFRTFIQDRFDE----EDRDSDL--      419
KJH51504.1          --MSDKIK---------EDKQTLKRLRILQKIYDRVKMI--RQRSDD----GVEEGRV--      430
VDL69795.1          --SKINPP---------ITQEHIAQLLVLHEIYENLSGI--TTEFRE----KERNKDI--      392
VDO32671.1          --IDEIPA---------RNPVIEAQLALLHKVYEEISEVCICDYLEK----EEREERI--      423
VBB32409.1          --SQTNPS---------IDIGQQATLIILQRIYQELKMI--TKRMID----AEKDDAK--      443
VDN54565.1          ----QINQ---------VDIGQQATILVLQRIYQELKTI--TKRMIE----ADREGTQ--      461
EGT55171.1          --SANGTS---------TDVSQQATLLILHRIYHELKIV--TKRMIE----GDKEEQA--      475
RCN52111.1          --SQITAP---------IDLGQQATLLILQRIYHELKVV--TKRMVD----TDREEQA--      477
RMX54856.1          NSE-TGTDGEVRKPLNNGDRRMEEMLKMQEKLLECVQIL--TKEVAK----NEDVQEK--      529
XP_022781674.1      NSE-TGTDGEVRKSLNDGDRSTEEILKMQEKLLECVQIL--TKEVAK----NEDMQEK--      790
RNA37099.1          RSVKP-NCD-----------KHNENCCLYDIVLNSMKNI--HDIKSM----EESERVI--      575
KAA0187152.1        NSISHSPV-------------VAGGCRLQEEILMALRAL--LSRQLR----QDTDLKR--      710
GAU96593.1          KNVGVGPTV-----------RGRSRDCLQDEIVRALSLL--TTRQEV----DDNILAV--      509
XP_009043980.1      ------RDR-----------THERKDDHCDKVTNHLRVL--VGRQDS----EDEHQDI--      403
VDM43573.1          ------VRK-----------KKRKHNELHMRLLKTLQVL--IRRQEM----EDHCQTL--      535
XP_003140283.1      ------ARR-----------TKR-STDLEFRLHKLLQTF--LKRQET----NELYQML--      422
VDN82010.1          ------ARR-----------TKR-SIDLQVRLHMLLQTF--LKRQET----NELYQML--      447
VDO31501.1          ------ARR-----------TKR-SIDLQVRLHMLLQTF--LKRQET----NELYQML--      459
VDP19591.1          NNVSFNPQME-SNPLTISIDLLNLYKSDLQIIINELHFI--TKKLRD----NEIESLI--      658
EDO32053.1          ------------------------------------------------------------      324
TRY67230.1          ------------IHACPCGRSGNIVEKELKLILTELQVI--TNKIHE----KDRSAQF--      435
KAF7391312.1        ------------SASLP----LGGMQRELHTILKELQFI--TSRMRK----ADENDEV--      462
GFG30449.1          --------------------------------------------LI--------------      462
KQS30083.1          ------------------------------------------------------------      397
EDW57583.2          ------------HHTCI----KSSTEYELGLILKEIRFI--TDQLRK----EDEDNDI--      652
KNC22799.1          ------------HHTCI----KTQTEYELSLILKEIRFI--TDQLRK----EDEENDI--      461
RZF44856.1          ------------LHSCL----G--PHRELTLILKELRII--TDKLRK----EDEASEV--      457
ENN76856.1          ------------------------------------------------------------      431
KAF5300392.1        ------------NHTCL----G--ADYELALILKEIRFI--TDQLRK----EDEHADV--      485
CAB3239999.1        ------------AHSCF----G--VDYELSLILKELRVI--TDQMRK----DDEDADI--      446
PCG77624.1          ------------AHSCF----G--VDYELSLILKEIRVI--TDQMRK----DDEDADI--      389
PZC79131.1          ------------AHSCF----G--VDYELSLILKEIRVI--TDQMRK----DDEDADI--      467
KAF4083067.1        --------------------------------------------QRE--------EAL--      328
XP_009924659.1      KKAQE---------------------------------------NRQ--------PAK--      381
XP_010123544.1      NKAPG---------------------------------------KGQ--------PAQ--      382
CBN81618.1          MAKEVSYLK-----I---------------------------------------------      381
KAF3704230.1        VAEEDNRAK-----LMEECQALEKLSGELKAIEKTLALL--LNNRKE--------EEK--      420
RUS86578.1          ----PNPGL-----KIDKGMSLMKAVQSVTSPEKALPPV--VDTE---------------      428
XP_034309618.1      ---------------------------------------------------------MFN      371
PIK58946.1          ------------------QISLES--EG--------KHI--SKAEK-----------A--      432
VUZ42516.1          QKDPFSNLT-----STTAGKQLEASVKEIK---RALRNL--LHKFNE----KDKDSRL--      460
PAA51166.1          ----DSPIL-----AGGHAGAVEKDVREVK---RCLRMY--LSRLKE----KERKNVL--      499
TGZ55997.1          ----FSPD------LLQSTATLEHDVREVK---RYVKMF--VNRQKE----IHRKNLV--      551
VDP48851.1          MINYDESE------SFNSTTTLERDVRELK---KYVKIV--VNRQKE----TTHKSLI--      473
PVD38331.1          G----GDDF-----SSPGVAPLMEEVRAI-------RDI--LEKVRDKKNKMDEKEKF--      465
VDK73355.1          -TESDQRIQ-----KLYHSPHMIKAFENI-------CFI--AELLKK----KDRDARV--      428
VDD84963.1          NEYGDERIQ-----KLYSSPVVIKAFENI-------CFI--AELLKR----KDNDDKV--      451
VDK42196.1          MAPVDERIQ-----KLYYSPAVVKAFENI-------CFI--AELLKK----KDRDDKV--      384
KFD58288.1          VSA-HHMFF-----DEEESMAIATAVNNT-------CFI--ANHFRK----KTAEDEV--      465
OUC49089.1          TSP-QQTWF-----DEEENAAIASAVRST-------CFI--ANHFRK----KAAEDEV--      464
EYC26492.1          ------SSF------QREISPVRSAVESV-------AYI--ADHLKN----EEDDKQV--      492
VDK27218.1          ------TSF------QKEFTPAMSAVDSV-------SFI--ADQMKD----DKDGQQV--      427
VDM17286.1          --ESETSAL-----EDESTPSLNTIIKSV-------KRI--TKHVMA----EEREKQV--      345
VDL91846.1          -------------------------IR---------------------------------      415
KAE9548540.1        KKLDPKNVK-----EKVNDLIFLNLIRQV-------KFI--AEHFRR----NEEESEE--      496
PDM74087.1          LPKDPSVSM-----YYPLSAQAIDAIDAI-------EYI--TEYIRK----DEELKMF--      438
```

```
XP_024504322.1    TGKDIFTAQ-----FYPLTAEASKAIDAI-------EYI--TDYIKQ----DEEYKMC--    451
PIO52336.1        TTRDPLLLS-----YYPLSADALQAIDAI-------DYI--TDHLKH----EEEHKMY--    437
TKR73865.1        SDFDSATSE-----FYPLSPEALNAIDAI-------EYI--TDHLKR----DEELKMY--    440
RLU23395.1        DRRGSESSD-----SVLLSPEASKATEAV-------EFI--AEHLRN----EDLYIQT--    473
XP_002427906.1    GRRESESSD-----SLLLSPEASKATEAV-------EFI--AEHLRN----EDQYIQI--    460
TMW47392.1        GRRESESSD-----SILLSPEASKATEA-----------------------------T--    454
VDD83956.1        --------M-----EGLSIDQIEASLDKV-------RHT--VLHFRQ----QEKRNKI--    435
VEL33078.1        --KNHVHSY-----PWKLERDEDIKTMGE--------LI--SNRQKR----IKIQNEQVR    423
XP_009019088.1    FNKDSHFIS-----TSNLSPSVQDAVRGA-------LYI--ANHLKR----QDEFSRVFR    447
KAE9536378.1      ALNDVPAAI-----RKKYPFELEKAIHNV-------KFI--QHHLQR----QDEYNTE--    529
KDR23473.1        SLSD--VGT-----RKKYPFELEKAIHNV-------MFI--QHHMQR----QDEFDAE--    471
KAF7266914.1      MGGSSTNIT-----PKNLSANVLAALEGV-------RFI--AQHIRD----ADKDNEI--    480
KAF4519445.1      ------------------------------------------------------------    400
OXU25983.1        SVTDSENTM-----PRHLTPDIVTALQGV-------RFI--AQHIKD----ADKDNEV--    468
TGZ32403.1        SVTDSENIM-----PKHLSPDVISALKGV-------RFI--AQHIKD----ADKDNEV--    474
ELT94491.1        M-AAAPGQR-----ARTLPPTVMQAMIGV-------AFI--SDHMKQ----QDEFNRI--    470
VVC39575.1        NVPLPKGKK-----HWHDCPELHKAAEGA-------AFI--ADYIRK----EEEEKKV--    563
RVE49089.1        --------R-----RWHRCPELHKAIDGI-------NYI--ADQTRK----EEESTRV--    470
KAB0800277.1      GANEPSPIF-----EKLGIHDIEKTIADS-------RFI--AQHVKN----KDTYENV--    486
TDG52197.1        AAADLSPTF-----ERPYAREMEKTIEGS-------RFI--AQHVKN----KDKFESV--    486
TMW48669.1        ------------------------------------------------------------    335
XP_025896085.1    -EPETP-------GEAGG---SGWAR----ALLREVAAV--RELLRR----REELQAA--    447
KAF2977017.1      -------------RRL----------------------S--RHLLAH----YQKGTRP--    222
XP_009321837.1    -AAAFA-------GQAEG---SPLMH----GVLREIPAI--RQFLEK----REEFRDV--    423
XP_009979985.1    -PTS-M-------XPXXX---XXXXX----XXXGDPTAT--RRFLEK----REEFRDV--    391
XP_028942374.1    -XXXXX-------XXXXX---XXXXX----XX----XXX--XXXLEK----REEFRDV--    416
PKK19633.1        -APAFA-------EQKEG---SSL-------------CR--RPVLVT----EEQIGQ---    476
XP_009894240.1    ------------------------------------------------------------    500
XP_032820219.1    --------------GQMP---TSTIR----RVLREISGL--RRHLEK----QEEEKQV--    459
XP_029429480.1    -------------PQQEG---TLVVD----HILQEILAI--RQQLRR----QWEDQSM--    461
XP_030077572.1    -----L-------PQREG---ALIED----NILQEIMAI--HQQLKK----QEEHPAI--    420
XP_033774596.1    -----L-------PQQEG---AHVVD----NILQEITAI--RQQLKR----Q-ENPDT--    443
ETE72600.1        ----SP-------LQGQS---PESVA----HILQEIAAI--RQFLEK----RDEFRDI--    436
XP_018082638.1    -----V-------PSKEN---SLVVD----NILHEIASI--RQYLEK----RDQCRDI--    649
XP_032905410.1    -----P-------PVSDS---LQGVD----CILHEVSAI--RHFLEK----RERFRDI--    428
XP_020773490.1    -LLGFPSSR---D-PPAP---PPVMN----NILQEVTAI--RHFLEK----RDRCREV--    456
XP_033833634.1    -LLGFPSSR---D-PPPP---PPVMN----NILQEVTAI--RHFLEK----RDRCREV--    465
XP_030215795.1    --------------GLGV---PPVVD----NILQEVTSI--RHFLEK----RDKCREV--    482
XP_030602980.1    -GLHLTPS------KENA---PPLMD----NILQEVTTI--RHFLEK----RDKCREI--    458
XP_004573543.1    -GLHLTPA------KDNV---PPLMD----NILQEVTTI--RHFLEK----RDKCREI--    451
XP_005916159.1    -GLHLTPA------KDNV---PPLMD----NILQEVTTI--RHFLEK----RDKCREI--    452
XP_013132089.1    -GLHLTPA------KDNV---PPLMD----NILQEVTTI--RHFLEK----RDKCREI--    452
XP_031603488.1    -GLHLTPA------KDNV---PPLMD----NILQEVTTI--RHFLEK----RDKCREI--    444
XP_005725536.1    -GLHLTPA------KDNV---PPLMD----NILQEVTTI--RHFLEK----RDKCREI--    444
XP_026038075.1    -GLHLTPA------KDNV---PPLMD----NILQEVTTI--RHFLEK----RDKCREI--    458
CAF96649.1        -RLGLPP------HRDPT---PPVMS----NILREVTAI--RSFLEK----RDRCREV--    417
XP_023818570.1    -GLRPPS------SRDIN---PPVMD----NILQEVTAI--RGFLEK----RDRCREI--    448
RVE64289.1        -GLRPAL------SRDSS---PPVMD----NILQEVTAI--RGFLEK----RDRCREV--    483
XP_024121971.1    -GLRPPS------SRDSS---PPVMD----NILQEVTAI--RGFLEK----RDRCREI--    454
XP_015817559.1    -GLRLPP------PRDNT---PPVMD----NILQEVTAI--RHFLEK----RDRCREI--    438
XP_015225670.1    -GLRLPP------SRDNT---PPVMD----NILQEVTAI--RQFLEK----RDRCREI--    358
XP_012722063.1    -GFRLAP------PRDNT---PPVMD----NILQEVTAI--RQFLEK----RDRCREI--    452
XP_032431307.1    -GLRLAP------SRDNT---PPVMD----NILQEVTAI--RQFLEK----RDRCREI--    452
XP_014328329.1    -GLRLAP------SRDNT---PPVMD----NILQEVTAI--RQFLEK----RDRCREI--    452
XP_027886578.1    -GLRLAP------SRDNT---PPVMD----NILQEVTAI--RQFLEK----RDRCREI--    452
XP_008426791.1    -GLRLAP------SRDNT---PPVMD----NILQEVTAI--RQFLEK----RDRCREI--    452
XP_014838686.1    -GLRLAP------SRDNT---PPVMD----NILQEVTAI--RHFLEK----RDRCREI--    452
XP_014885967.1    -GLRLAP------SRDNT---PPVMD----NILQEVMAI--RHFLEK----RDRCREI--    452
XP_033954312.1    -GLGRPL------PRDPT---PPVMD----NILQEVTAI--RHFLEK----RDRCREV--    458
XP_034089244.1    -GLGRPL------PRDPT---PPVMD----NILQEVTAI--RHFLEK----RDRCREV--    447
XP_010780064.1    ------------------------------------------------------------    330
XP_033970261.1    -GLGRPL------PRDPT---PPVMD----NILQEVTAI--RHFLEK----RDRCREV--    474
XP_029375575.1    -GLGLPPSR---DPT------PPVMD----NILHEVTAI--RHFLEK----RDRCREV--    489
KAF0023022.1      -GLGLPPSR---EPAPPA---PPVMD----NILHEVTAI--RHFLEK----RDRCREV--    490
XP_019952614.1    -GLGLPPSR---DPT------PPVMD----NILHEVTAI--RHFLEK----RDRCREV--    443
XP_034463117.1    -GLGLPPSR---DPT------PPVMD----NILHEVTAI--RHFLEK----RDRCREV--    455
XP_029924656.1    -GLGLPP-----S-RDPT---PPVVD----NILQEVTSI--RHFLEK----KDRCRDV--    453
XP_028323228.1    -GLHLPP-----S-RDSS---PPVMD----NILQEVTAI--RTFLEK----RDRCREV--    449
TNM84480.1        ------------------------------------------------------------    311
XP_029703788.1    -RLGLPP-----R-RDPT---PPVMN----NILQEVMAI--RHFLEK----RDRCREV--    452
XP_030010368.1    -GLGLPPS----R-EPPT---PPVMD----NILQEVTAI--RHFLEK----RDRCREV--    455
XP_019725056.1    -GRGLPP-----S-RDPS---PPVVS----NILQEVTAI--RNFLEK----RDRCNEV--    450
XP_034034934.1    -GLGLPP-----S-RDST---PPVMD----NILQEVTAI--RHFLEK----RDRCREV--    441
XP_029956880.1    -GLHLPP-----P-RDAA---PPVMD----SILQEVTAI--RSFLEK----RDRCREV--    452
XP_008331307.3    -GMNRPL-----P-REPT---PPVMD----NILQEVTAI--RHFLEK----RDRCREV--    487
XP_031724845.1    -GLGLPP-----S-RDPT---PPVMD----NILHEVTAI--RHFLEK----RDRCREV--    453
XP_013859395.1    -GLRLPP-----S-RDST---PPIMD----NILQEVTAI--RHFLEK----RDKCREV--    453
```

```
XP_017275335.1      -GLRLPP-----S-RDSN---PPVMD----NILQEVTAI--RHFLEK----RDKCREV--      453
XP_020496197.1      -GLGLPP-----S-REPT---PPVMD----NILQEVTAI--RHFLEK----RDKCREV--      451
XP_029030400.1      -GLGRPL-----A-RDAG---PPVMD----HILQEVTAI--RHFLEK----RDRCREV--      452
XP_022060538.1      -GL--HRTP---P-RDPG---PPVMD----SILQEVTAI--RHFLEK----RDRCREV--      454
XP_023150584.1      -GL--HRPP---S-RDPG---PPVMD----NILQEVTAI--RHFLEK----RDRCREV--      454
XP_029303892.1      -GLGLPP-----S-RDPT---PPVMD----NILQEVTAI--RHFLEK----RDRCREV--      476
XP_020454172.1      -GLGLPP-----S-RDPT---PPVMD----NILQEVTAI--RHFLEK----RDRCREV--      451
TKS83244.1          -GLGLPP-----S-RDPT---PPVMD----NILQEVTAI--RRFLEK----RDRCREV--      436
XP_022616583.1      -GLGLPP-----S-RDPT---PPVMD----NILHEVTAI--RHFLEK----RDRCREV--      482
XP_030293172.1      -RMGLPPVP---S-RDPT---PPVMD----NILQEVTAI--RHFLEK----RDRAREV--      454
XP_027129539.1      -GLGLPP-----S-RDPT---PPVMD----NILQEVTAI--RHFLEK----RDRCREV--      449
XP_034539538.1      -RLGLPP-----S-RDPT---PPVMD----NILQEVTAI--RHFLEK----RDRCREV--      452
XP_008277330.1      -GLHLPP-----S-RDSS---PPVMD----NILQEVTAI--RHFLEK----RDRCREV--      452
XP_028276940.1      -GLHLPP-----S-RDST---PPVMD----NILQEVTAI--RHFLEK----RDRCREV--      452
XP_023263433.1      -GLGLPP-----S-RDPT---PPVMD----NILHEVTAI--RHFLEK----RDRCREV--      449
XP_034406336.1      -GLGLPP-----S-RDHA---PPVMD----NILHEVTAI--RHFLEK----RDRCREV--      452
XP_026184460.1      -GLGLPP-----S-RDST---PPVMD----NILQEVTAI--RHFLEK----RDRCREV--      452
KAF1378228.1        -GLGLPP-----S-RDHA---SPVMD----NILQEVTAI--RHFLEK----RDRCREV--      437
XP_028450365.1      -GLGLPP-----S-RDHT---PPVMD----NILQEVTAI--RHFLEK----RDRCREV--      441
XP_031163851.1      -GLGLAP-----S-RDHT---PPVMD----NILQEVTAI--RHFLEK----RDRCREV--      452
XP_032389084.1      -GLGLAP-----S-RDHT---PPVMD----NILQEVTAI--RHFLEK----RDRCREV--      452
XP_033494682.1      -GLGLPP-----S-RDPT---PPVMD----NILQEVTAI--RHFLEK----RDRCREV--      452
XP_018544782.1      -GLGLPP-----S-RDPT---PPVMD----NILHEVTAI--RHFLEK----RDRCREV--      452
XP_026228189.1      -GLGLPP-----S-RDNT---PPVMD----NILQEVTAI--RHFLEK----RDRCREV--      452
XP_028976606.1      -VGLPV-------VRDNI---PPVME----NILHELSSI--RGYLEK----KDESRDI--      499
CAB1352378.1        ---LSV-------VRDNT---PPVMD----NILHEISSI--RGFLEK----RDESRDI--      410
XP_023866849.1      ---LSV-------VRDNT---PPVMD----NILQEISSI--RGFLEK----RDESRDI--      453
XP_013992832.1      ---LSV-------VRDNT---PPVMD----NILQEISSI--RGFLEK----RDESRDI--      453
XP_029546688.1      ---LSV-------VRDNT---PPVMD----NILQEISSI--RGFLEK----RDESRDI--      453
XP_021481546.1      ---LSV-------VRDNT---PPVMD----NILQEISSI--RGFLEK----RDESRDI--      453
XP_020339889.1      ---LSV-------VRDNT---PPVMD----NILQEISSI--RGFLEK----RDESRDI--      453
XP_029481972.1      ---LSV-------VRDNT---PPVMD----NILQEISSI--RGFLEK----RDESRDI--      453
KPP68743.1          -GLVPP-------SRDS----APVMD----SILLEICSI--RQFLDK----RDECRDI--      419
XP_023669383.1      -GLLLP-------ARDS----TPVMD----SILLEISSI--RRFLDR----RDESRDI--      441
KAA0720096.1        -SLGMT-------VRDTS---PPVMD----SILHEITSI--RQFMEK----KDESREI--      348
XP_009293684.1      -GLGLS-------AHESG---PPVMD----GILREITTI--RQFLEK----KDESREI--      442
XP_026090784.1      -GLGLS-------IRDSG---SPVMD----GILREITTI--RQFMEK----KNESREI--      442
XP_018918715.1      -GLGLS-------TRDSG---SPVMD----GILREITTI--RQFLEK----KDESREI--      442
KAF4098549.1        -GLGLS-------TRDSG---SPMMD----GILREITTI--RQFLEK----KDESREI--      442
XP_016084173.1      -GLGLS-------MRDSG---SPVMD----GILREITTI--RQFLEK----KDESREI--      442
XP_016332759.1      -GLGLS-------TRDSG---SPVMD----GILREITTI--RQFLEK----KDESREI--      442
XP_016398039.1      -GLALS-------TRDSG---SPVMD----GILREITTI--RQFLEK----KDESREI--      442
XP_026989970.1      -IMGLP-------IRDST---PPVMD----NILHEVSSI--RQYLEK----RQACRDI--      441
TSQ12698.1          -IMGLP-------IRDTT---PPVMD----NILKEVSFI--RQYLEK----RQTCRDI--      442
XP_017347546.1      -IMGLP-------IRDST---PPVMD----NILHEVSSI--RQYLEK----RQACRDI--      436
XP_026794616.2      -VMGLP-------IRDST---PPVMD----NILHEVSSI--RQYLEK----RQACRDI--      442
XP_026861889.1      -GVGLP-------VRDTT---PPVMD----NILHEVSSI--RQFLEK----REACRDI--      440
XP_017575347.1      -GIGLP-------VRDST---PPVMD----NILHEVSSI--RQFLEK----REASREI--      440
XP_022531596.1      -GMGMP-------VRDST---PPVMD----NILHEVSSI--RQFLEK----REASREI--      440
XP_012691019.2      -SLGLP-------VRDPT---PPVMD----NILQEVSCI--RQYLDR----RDESRDV--      425
XP_028839723.1      -GLSLP-------IRDNT---PPVMD----NILHEITSI--RQFLER----RDESRDI--      443
XP_030638860.1      -GLALP-------LRENT---PPIMD----NILQEISSI--RQFLEK----RDESRDI--      423
XP_007882964.1      ----GV-------APGDP---GAPET----SILQEIVAI--RRFLEK----RDEHRDI--      421
GCB70244.1          ------------------------------------------------------------      377
GCC26242.1          ----SF-------PQADS---LLTVD----NILHEISAI--RQFLEK----RDECRDI--      441
XP_020387034.1      ----SL-------PQADT---HLTVD----NILHEISAI--RQFLEK----RDEYRDI--      432
XP_028931940.1      -DSPPP-------PPRES---SLAVR----GMLQELAAI--RRFLER----REECRDI--      445
XP_031762490.1      -------------PSKEN---PLVVD----NILHEIASI--RQYLEK----RDQCRDI--      434
PIO32240.1          -------------PTQES---SIVVD----NILHEIASI--RQYLEK----RDEYRDI--      232
XP_018425466.1      -------------PTKES---SVVVD----SILHEIASI--RQYLEK----RDEYRDI--      435
XP_006642351.1      -------------PLRDN---APVVD----NILQEISSI--RHFLER----REQYREI--      458
XP_028665372.1      -------------PLKEN---APVVE----SILQEISSI--RQYLEK----RDEYRDI--      441
GCF49814.1          -GMVPQ-------AQGGG---SHVFE----SILQEIASI--RQFLEK----RDEFRDI--      420
XP_015277816.1      -GPPPP-------PPSGG---SHIFK----SILQEIAAI--HQFLEK----RDEFRDI--      445
XP_005987340.2      -----L-------PQRDN---AAVVD----NILHEISSI--RQYLEK----RDEYRDI--      425
XP_019339534.1      -MLTTL-------ARGES---PAMMH----SILQEIISI--RHFLEK----RDASRAI--      447
XP_025063414.1      -MLTTL-------ARGES---PAMMH----SILQEIISI--RHFLEK----RDASRAI--      447
XP_019367488.1      -MLATL-------ARGES---PAMMH----SILQEIIAI--RHFLEK----RDASRTI--      447
XP_019412078.1      -MLAPL-------ARGES---PAMMH----SILQEITAI--RHFLEK----RDASRAI--      447
XP_014434314.1      -APGPP--------PGES---SPVAD----SILQEIAAI--RQFLEK----RDEFRDI--      453
XP_007060556.1      -LPAPP-------AQGES---CPVVE----SILQEIAAI--RQFLEK----RDEFRDI--      441
XP_024072175.1      -LPAPP-------AQGDS---SPVVA----SILQEISAI--RQFLEK----RDEFRDI--      431
XP_008170567.1      -LPAPP-------AQGDS---SPVVA----SILQEISAI--RQFLEK----RDEFRDI--      447
XP_034610543.1      -LPALP-------AQGDS---SPVVA----SILQEISAI--RQFLEK----RDEFRDI--      447
XP_030394127.1      -LP----------AQGES---SPVVA----SILQEISAI--RQFLEK----RDEFRDI--      444
XP_032651960.1      -LPAPP-------AQGES---SPVMA----SILQEISAI--RQFLEK----RDEFRDI--      447
XP_025020083.1      -A---Q-------LQGES---PLSVE----NILQEIAAI--RQFLEK----RDEFRDI--      444
```

```
XP_026536833.1      -S---P-------LQGQS---PESLA----HILQEIAAI--RQFLEK----RDEFRDI--      444
XP_026564787.1      -S---P-------LQAQS---PESLA----HILQEITAI--RQFLEK----RDEFRDI--      444
XP_032084675.1      -A---P-------PQGQS---PESVA----HILHEIAAI--RQFLEK----RDEFRDI--      429
XP_029139341.1      -D---P-------LQGES---PESVA----HILQEIAAI--RQFLEK----RDEFRDI--      444
XP_034281006.1      -A---P-------LQGQS---PELVA----HILHEIAAI--RQFLEK----RDEFRDI--      444
XP_020649062.1      -H---R-------TQGEN---SQVVE----NILQEIMAI--RQFLEK----RDEFRDI--      444
XP_008117087.1      -A---T-------SQGEG---SLVVE----NILQEIASI--RQFLEK----RDEFRDI--      444
XP_028564324.1      -T---Q-------PQGES---SLVVE----SILQEIAAI--RQFLEK----RDEFRDI--      450
XP_033028155.1      -T---Q-------PQGES---SLVVE----NILQEIAAI--RQFLEK----RDEFRDI--      449
XP_025963249.1      -APAFP-------TEAEG---SPLLH----SLLREITTI--RQFLEK----REEFHSV--      431
XP_009668348.1      -MPAFP-------TEAEG---SPLMH----SILREITTI--RQFLEK----QEEFHDV--      446
XP_013798935.1      -MPAFP-------AEAEG---SPLMH----SLLREITTI--RQFLEK----REEFHHV--      430
XP_025913685.1      -MPAFP-------AEAEG---SPLMH----SLLREITTI--RQFLEK----REEFHHV--      455
XP_013042552.1      -TPAFG-------TLAEG---SPLTH----SILHEITTI--RQFLEK----RDEFRDV--      453
XP_005030458.2      -APAFG-------SVAEG---SPLMP----SILHEISTI--RQFLEK----RDEFRDV--      446
XP_032057953.1      -APAFG-------SLAEG---SPLMP----SILHEISTI--RQFLEK----RDEFRDV--      429
XP_021232050.1      ------------------------------------------------------------      408
OXB62403.1          -TPAFA-------TPAEG---SLLVH----SILHEITTI--RQFLEK----RDEFRDV--      447
OXB81319.1          -TPAFA-------TPAEG---SLLVH----SILHEISTI--RQFLEK----RDEFRDV--      429
XP_010722007.1      -TPAFA-------TPAEG---SLLIH----SILHEITTI--RQFLEK----RDEFRDV--      448
XP_015739349.1      -TPAFA-------TPAEG---SLLIH----SILHEITTI--RQFLEK----RDEFRDV--      448
XP_031455498.1      -TPAFA-------TPAEG---SLLIH----SILHEITTI--RQFLEK----RDEFRDV--      449
POI27435.1          -TPASA-------TPAEG---SLLIH----SILHEITTI--RQFLEK----RDEFRDV--      424
XP_004948120.1      -TPASA-------TPAEG---SLLIH----SILHEITTI--RQFLEK----RDEFRDV--      449
XP_032851190.1      -TAAFA-------GPAQL---QHRGQ----RPRHRRPPG--GPA------------GP--      436
XP_010007255.1      -T-------------SEG---SLLAH----GILREVTAI--RQFLEK----REEFRDV--      440
XP_030320702.1      -P-------------GEG---SPLAH----GLLREVTAI--RQFLEK----REEFRDV--      405
XP_010191940.1      -ALAAA-------GPVEG------------------------------------------      417
XP_027737112.1      -APTFA-------SRAEG---SAALQ----GVLRETTAI--RQFLEK----REEFRDV--      442
XP_027555032.1      -ALAFG-------SRAEG---SAVLQ----EVLRETTAI--RQFLEK----REEFRDV--      447
XP_032565370.1      -TLAFG-------SRAEG---SAVLQ----EVLRETTAI--RQFLEK----REEFRDV--      493
XP_027511217.1      -ALAFG-------SRAEG---SAVLQ----EVLRETTAI--RQFLEK----REEFRDV--      592
XP_027593499.1      -ALAFG-------SRAEG---SAVLQ----EVLRETMAI--RQFLEK----REEFRDV--      543
XP_017664924.1      -ALAFG-------SRAEG---SAVLQ----EVLRETMAI--RQFLEK----REEFRDV--      444
XP_029817938.1      -ALAFG-------SRAEG---SAVLQ----EVLRETMAI--RQFLEK----REEFRDV--      426
XP_005058641.1      -ALAFA-------GRTEG---SAALQ----EVLREATAI--RQLLEK----REEFRDV--      479
XP_021385804.1      -ALAFA-------GQTEG---SAALQ----EILCETTAI--RQLLEK----REEFREA--      444
XP_030146687.2      -ALAFA-------GQTEG---SAALQ----EILRETTAI--RQLLEK----REEFRDA--      491
KAF4796420.1        -ALAFA-------GRMDG---SAALQ----EVLRETTAI--RQLLEK----REEFRDV--      446
XP_032937581.1      -ALAFA-------GRAEG---SAALQ----EVLRETTAI--RQLLEK----REEFRDV--      493
XP_031989659.1      -TLAFA-------GRTEG---LAALQ----EVLRETTAI--RQLLEK----REEFRDV--      447
XP_010402086.1      -TLAFA-------GRTEG---LAALQ----EVLRETTAI--RQLLEK----REEFRDV--      446
XP_017594069.1      -TLAFA-------GRTEG---LAALQ----EVLRETTAI--RQLLEK----REEFRDV--      417
XP_014115268.1      -ALAFT-------GRPEG---SGVLQ----EVLHETTAI--RQLLEK----REEFRDV--      471
XP_023797108.1      -ALAFT-------GRPEG---SGALQ----EILHETTAI--RQLLEK----REEFRDV--      417
XP_033375614.1      -AVAFT-------GRPEG---SGALQ----EVLHETTAI--RQLLEK----REEFRDV--      417
XP_014740121.1      -ALAFA-------GRTEG---SAALQ----EMLRETTAI--RQLLEK----REEFRDV--      591
RLV83430.1          -ALAFA-------GQTEG---SAALQ----EILRETTAI--RQLLEK----REEFRDA--      437
XP_009096098.2      -ALAFA-------GRAEG---SAALQ----EVLRETMAI--RQLLEK----REEFRDL--      553
TRZ15870.1          -ALAFA-------GRTEG---SAALQ----EVLRETMAI--RQLLEK----REEFRDV--      447
RMB91935.1          -ALAFA-------GRAEG---SAALQ----EVLRETTAI--RQLLEK----REEFRDV--      417
XP_030820843.1      -APAFA-------GRPEG---SAALQ----EVLRETTAI--RQLLEK----REEFRDL--      493
XP_014165179.1      -APAFA-------GRPEG---SAALQ----EVLRETTAI--RQLLEK----REEFRDL--      495
XP_026653582.1      -ALALA-------GRPEG---SAALQ----EVLRETTAI--RQLLEK----REEFRDL--      377
PKU35975.1          -APASA-------GPAEG---SPLLP----GVLREITAI--RQFLEK----REEFRDV--      448
XP_014805072.1      -AAA---------SGAEG---SPLLP----GVLREITAI--RQFLEK----REEFRDV--      440
XP_009818330.1      --------------------------------------------------------DI--      158
OPJ68307.1          -APAFA-------KQTEG---SSLAQ----GVLREVAAI--RRFLEK----REEFRDV--      445
XP_008936289.1      -TPAFA-------GPSEG---SVLML----GILREVTAI--RQVLGK----REDFREV--      447
XP_010287046.1      -APAFA-------SQAEG---SLLMH----AILREITAI--RQFLEK----REEF-----      374
XP_005240140.2      -----D-------GQAEG---SGLVH----GVLREITAI--RQLLEK----REEFREV--      500
XP_005437752.2      -TPTSA-------GQAEG---SGLVH----GVLREITAI--RQLLEK----REEFREV--      506
KFV74811.1          -----A-------RLAEG---SPLVL----GVLREITAI--RRFLEK----REEFRDV--      406
XP_010018389.1      -XPAFA-------GQAEG---SLVMH----GVLREIPAI--RQFLEK----REEFRDV--      453
KQK78711.1          -APAFA-------SQADG---SLLMH----DVLREIXAI--RQFLQK----HEEFRDV--      446
XP_009570162.1      -TPGFA-------GHAEG---SLLMQ----GVLREITAI--RQFLEK----REEFRGA--      445
KFP11268.1          -TPALA-------GQAEG---SPLMH----GVLREIAAI--RQFLEK----REEFRDV--      409
KFQ98910.1          -APAFA-------GQAEG---SPLTH----GLLREIAAI--RQFLEK----REEFRDV--      428
XP_012985202.3      -ALGCA-------SQADG---SLLMH----GVLHEITAI--RQFLEK----REEFRDV--      446
XP_010573388.1      -APAFA-------GQAEG---SPLMH----SILREITAI--RQFLEK----REEFRDV--      445
XP_029879496.1      -APAFA-------GQAEG---SPLMH----SVLREITAI--RQFLEK----REEFRDV--      417
XP_030361086.1      -APAIA-------GHVEG---SLLMH----GVLREITAI--RQFLEK----REEFRDV--      498
KFM00668.1          -AAAFA-------GQAEG---SPLMH----GVLREITAI--RQFLEK----REEFRDV--      429
KAF1479074.1        -AAAFA-------GQAEG---SPLMH----GVLREITAI--RQFLEK----REEFRDV--      409
KAF1651161.1        -AAAFA-------GQAEG---SPLMH----GVLREITAI--RQFLEK----REEFRDV--      410
KAF1673648.1        -AAAFA-------GQAEG---SPLMH----GVLREITAI--RQFLEK----REEFRDV--      410
KAF1493319.1        -APAFA-------GQAEG---CLLMH----GVLREITAI--RQFLEK----REEFRDV--      410
```

KAF1584157.1	-APAFA-------GQAEG---SPLMH----GVLREITAI--RQFLEK----REEFRDV--	410
KAF1571723.1	-APAFA-------GQAEG---SPLMH----GVLREITAI--RQFLEK----REEFRDV--	410
KAF1533169.1	-APAFA-------GQAEG---SPLMH----GVLREITAI--RQFLEK----REEFRDV--	410
KAF1638955.1	-APAFA-------GQAEG---SPLMH----GVLREITAI--RQFLEK----REEFRDV--	410
KAF1549972.1	-APAFA-------GQAEG---SPLMH----GVLREITAI--RQFLEK----REEFRDV--	410
KAF1606914.1	-APAFA-------GQVEG---SPLMH----GVLREITAI--RQFLEK----REEFRDV--	410
KAF1510957.1	-APAFA-------GQAEG---SPLMH----GVLREITAI--RQFLEK----REEFRDV--	410
KAF1498899.1	-APAFA-------GQAEG---SPLMH----GVLREITAI--RQFLEK----REEFRDI--	410
KAF1411525.1	-APAFA-------GQAEG---SPLMH----GVLREITTI--RQFLEK----REEFRDV--	410
KAF1429205.1	-APAFA-------GQAEG---SPLMH----GVLREITTI--RQFLEK----REEFRDV--	410
XP_005334318.1	-GPLPP--------PREA---SLAVR----GLLQELSSI--RHYLEK----RDEIREV--	432
XP_014395552.1	-SPPPP--------PREA---SLAMR----GLLQELASI--RHFLEK----REESREV--	432
KAB0404854.1	-TYPAV--------LPE----LIAKCNRRHPAISPFPQH--KRLLTQ----AGGPEPL--	435
XP_007494828.1	-NPSPL--------SQEN---SSVVH----GLLQELATI--KHFLEK----QDERRDI--	448
XP_003764254.1	-NPSPP--------FQEN---SLVVH----GLLQELATI--RHFLEK----QDECRDI--	448
XP_020845489.1	-NPSPP--------PQEN---SLIVH----GLLQELVTI--RHFLEK----QDECRDI--	448
XP_027703160.1	-NHSPP--------PQEN---SLVVH----GLLQELATI--RHFLEK----QDECRDI--	448
XP_017523929.1	-SPPPA--------PREA---SLATC----AVLQELSSV--RHLLER----REEGREV--	440
XP_008688428.1	-SPPPP--------PREA---SLAMR----GLLQELASI--RRFLEK----RDESREV--	388
XP_029812166.1	-SPPPP--------PREA---SLAVR----GLLQELSSI--RHFLEK----RDESREV--	457
XP_025749781.1	-SPPPP--------PREA---SLAVR----GLLQELVSI--RRFLEK----RDESREV--	443
XP_004416432.1	-SPPPP--------PREA---SLAVR----GLLQELVSI--RRFLEK----RDETREV--	443
XP_027436262.1	-SPPPP--------PREA---SLAVR----GLLQELVSI--RRFLEK----RDESREV--	443
XP_032284025.1	-SPPPP--------PREA---SLAVR----GLLQELVSI--RRFLEK----RDESREV--	443
XP_006735421.1	-SPPPP--------PREA---SLAVR----GLLQELVSI--RRFLEK----RDESREV--	443
XP_021552166.1	-TPPPP--------PREA---SLAVR----GLLQELVSI--RRFLEK----RDESREV--	443
XP_026361066.1	-SPPPP--------PREA---SLAMR----GLLQELASI--RRFLEK----RDESREV--	443
XP_034523598.1	-SPPPP--------PREA---SLAMR----GLLQELASI--RRFLEK----RDENREV--	443
NP_001297113.1	-SPPPP--------PREA---SLAVR----GLLQELASI--RRFLEK----RDESREV--	443
XP_032215488.1	-SPPPP--------PREA---SLAVR----GLLQELASI--RRFLEK----RDESREV--	443
VCX31483.1	-SPPPP--------PREA---SLAVR----GLLQELASI--RRFLEK----RDESREV--	443
XP_022369003.1	-SPPPP--------PREA---SLAVQ----GLLQELASI--RRFLEK----RDESREV--	442
XP_032694248.1	-SPPPP--------PREA---SLAVR----GLLQELASI--RRFLEK----RDESREL--	442
NP_001041584.1	-SPPPP--------PRES---SLAVR----GLLKELSSI--RHFLEK----RDESREV--	443
XP_025862501.1	-SPPPP--------PRES---SLAVR----GLLKELSSI--RHFLEK----RDESREV--	443
KAF0873564.1	-SPPPP--------PREA---SLAVR----GLLQELSSI--RLFLEK----RDESREV--	585
XP_025784751.1	-SPPPP--------PREA---SLAVC----GLLQELSSI--RHFLEK----RDESREV--	437
XP_007075625.1	-SPPPP--------PREA---SLAVC----GLLQELSSI--RHFLEK----RDESREV--	579
XP_030189489.1	-SPPSP--------PRES---SLAVC----GLLQELSSI--RHFLEK----RDESREV--	443
XP_019324655.1	-SPPPP--------PREA---SLAVC----GLLQELSSI--RHFLEK----RDESREV--	544
XP_023094886.1	-SPPSP--------PREA---SLAVC----GLLQELSSI--RHFLEK----RDESREV--	579
XP_026892357.1	-SPPPP--------PREA---SLAVC----GLLQELSSI--RHFLEK----RDESREV--	443
XP_016004457.1	-SPPPP--------PREA---SLAVH----GLLQELASI--RHFLEK----REESREV--	438
XP_006912860.1	-GPPPP--------PREA---SLAVH----GLLQELASM--RHFLEK----QEESREV--	438
XP_011363679.1	-GPPPP--------PREA---SLAVH----GLLQELASM--RHFLEK----QEESREV--	438
XP_016076060.1	-SPPPP--------PREA---SLAVR----GLLQELASI--RHLLEK----REDSREV--	443
XP_008148371.1	-SPPPP--------PREA---SLAVR----GLLQELASI--RHFLEK----REESREV--	443
XP_006093568.1	-SPPPP--------PREA---SLAMR----GLLQELASI--RHFLEK----REESREV--	443
XP_006761601.1	-SPPPP--------PREA---SLAMR----GLLQELASI--RHFLEK----REESREV--	443
XP_024426743.1	-SPPPP--------PREA---SLAVH----GLLQELASI--RHFLEK----REESREV--	438
XP_019520779.1	-SPPPP--------VREA---SLAVR----GLLQELASI--RHFLEK----REESREV--	443
XP_032976539.1	-SPPPP--------VREA---SLAVR----GLLQELASI--RHFLEK----REESREV--	443
ELW64270.1	-SPPPP--------PREA---SLAVR----GLLQELSSI--RRFLEK----RDEIREV--	447
XP_004427367.1	-SPPPP--------PREA---SLAVR----GLLQELASI--RHFLEK----REESREV--	443
XP_008529353.1	-SPPPP--------PREA---SLAAR----GLLQELASI--RHFLEK----RDESREV--	448
NP_001288165.1	-SPPPP--------PREA---SLAAR----GLLQELASI--RHFLEK----RDESREV--	443
XP_014711213.1	-SPPPP--------PGEA---SLAAR----GLLQELASI--RHFLEK----RDESREV--	443
XP_012514621.1	-SPPPP--------PRGA---SLAVH----GLLQELSSI--RHFLEK----RDEIREV--	443
XP_008071525.1	-SPPPP--------PREA---SLAVR----GLLQELASI--RHFLEK----RDEIREV--	440
XP_009005082.1	-GPPPP--------PQEA---SLAVS----ELLQELSSI--RHFLEK----RDEIREV--	443
XP_010332832.1	-GPPPP--------PREA---SLAVC----GLLQELSSI--RHFLEK----RDEIREV--	438
XP_012326058.1	-GPPPP--------PREA---SLAVS----GLLQELSSI--RHFLEK----RDEIREV--	449
XP_017378796.1	-GPPPP--------PREA---SLAVS----GLLQELSSI--RHFLEK----RDEIREV--	472
XP_032141374.1	-GPPPP--------PREA---SLAVS----GLLQELSSI--RHFLEK----RDEIREV--	472
XP_032024018.1	-SPPPP--------PREA---SLAVC----GLLQELSSI--RQFLEK----RDEMREV--	438
XP_018891497.2	-SPPPP--------PREA---SLAVC----GLLQELSSI--RQFLEK----RDEIREV--	444
XP_030684894.1	-SPPPP--------PREA---SLAVC----GLLQELSSI--RQFLEK----RDEIREV--	438
PNJ75904.1	-SPPPP--------PREA---SLAVC----GLLQELSSI--RQFLEK----RDEIREV--	444
AAP35868.1	-SPPPP--------PREA---SLAVC----GLLQELSSI--RQFLEK----RDEIREV--	444
XP_001149570.1	-SPPPP--------PREA---SLAVC----GLLQELSSI--RQFLEK----RDEIREV--	444
XP_003805532.1	-SPPPP--------PREA---SLAVC----GLLQELSSI--RQFLEK----RDEIREV--	444
XP_023063823.1	-SPPPP--------PREA---SLAVC----GLLQELSSI--RHFLEK----RDEIREV--	442
XP_033060210.1	-SPPPP--------PREA---SLAVC----GLLQELSSI--RHFLEK----RDEIREV--	442
XP_010370669.1	-SPPPP--------PREG---SLAVC----GLLQELSSI--RHFLEK----RDEIREV--	442
XP_017738496.1	-SPPPP--------PREA---SLAVC----GLLQELSSI--RHFLEK----RDEIREV--	442
XP_011782168.1	-SPPPP--------PREA---SLAVC----GLLQELSSI--RHFLEK----RDEIREV--	443
EHH23440.1	-SPPPP--------PREA---SLAVR----GLLQELSSI--RHFLEK----RDEIREV--	470

XP_003910778.3	-SPPPP--------PREA---SLAVR----GLLQELSSI--RHFLEK----RDEIREV--	443
XP_025212649.1	-SPPPP--------PREA---SLAVR----GLLQELSSI--RHFLEK----RDEIREV--	449
XP_008019132.1	-SPPPP--------PREA---SLAVR----GLLQELSSI--RHFLEK----RDEIREV--	443
XP_011832421.1	-SPPPP--------PREA---SLAVR----GLLQELSSI--RHFLEK----RDEIREV--	428
XP_011759618.1	-SPPPP--------PREA---SLAVR----GLLQELSSI--RHFLEK----RDEIREV--	443
XP_005579749.1	-SPPPP--------PREA---SLAVR----GLLQELSSI--RHFLEK----RDEIREV--	443
XP_011921684.1	-SPPPP--------PREA---SLAVR----GLLQELSSI--RHFLEK----RDEIREV--	235
XP_008259391.1	-SPPPP--------PREA---SLAVR----GLLQELASI--RHFLEK----RDEIREV--	443
XP_012782438.1	-SPPPP--------PREA---SLAMR----GLLQELASI--RHFLEK----RDEIREV--	439
VTJ86076.1	-GPPPP--------PREA---SLAVR----GLLQELSSI--RHYLEK----RDEIREV--	449
XP_015345533.1	-GPPPP--------PREA---SLAVR----GLLQELSSI--RHYLEK----RDEIREV--	449
XP_027786432.1	-GPPPP--------PREA---SLAVR----GLLQELSSI--RHYLEK----RDEIREV--	449
XP_026248345.1	-GPPPP--------PREA---SLAVR----GLLQELSSI--RHYLEK----RDEIREM--	443
XP_005378209.1	-SPPPP--------PREA---SLAVR----GLLQELSSI--RHFLEK----RDEIREV--	444
XP_004466010.1	-SPPPP--------PREA---SLAVR----GLLQELAAI--RHFLEK----RDESRQV--	443
XP_020035707.1	-SPPPP--------PRET---SLAVR----GLLQELSSI--RHYLEK----REEIREV--	437
XP_006181802.1	-SPPPP--------PREA---SLAMR----GLLQELTSI--RHFLEK----RDESREV--	443
XP_006207862.1	-SPPPP--------PREA---SLAMR----GLLQELTSI--RHFLEK----RDESREV--	443
XP_005891968.1	-SPPPP--------PREA---SLAVR----GLLQELTSI--RHFLEK----REGSREV--	440
XP_010828662.1	-SPPPP--------PREA---SLAVR----GLLQELTSI--RHFLEK----REGSREV--	440
XP_019830126.1	-SPPPP--------PREA---SLAVR----GLLQELTSI--RHFLEK----REGSREV--	439
XP_017914486.1	-SPPPP--------PREA---SLAVR----GLLQELTSI--RHFLEK----REGSREV--	439
XP_006060266.2	-SPPPP--------PREA---SLAVR----GLLQELTSI--RHFLEK----REGSREV--	444
XP_020728447.1	-SPPPP--------PREA---SLAVR----GLLQELTSI--RHFLER----REGSREV--	444
KAF4008892.1	-SPPPP--------PREA---SLAVR----GLLQELTSI--RHFLEK----REGSREV--	444
KAB0348059.1	-SPPPP--------TREA---SLAVR----GLLQELTSI--RHFLER----REGSREV--	427
KAB0371113.1	-SPPPP--------PREA---SLAVR----GLLQELTSI--RHFLER----REGSREV--	420
MBV96963.1	-SPPPP--------PREA---SLAVR----GLLQELTSI--RHFLEK----REESREV--	443
XP_007172048.1	-SPPPP--------PREA---SLAVR----GLLQELTSI--RHFLEK----REESREV--	443
XP_007452270.1	-SPPPR--------PREA---SLAVR----GLLRELTSI--RHFLET----REESREV--	443
XP_023987108.1	-SPPPP--------PRDA---SLAVR----GLLQELTSI--RHFLEK----REESREL--	438
XP_004273432.1	-SPPPP--------PREA---SLAVR----GLLQELTSI--RHFLEK----QEESREV--	438
XP_004328646.1	-SPPPP--------PREA---SLAVH----GLLQELTSI--RHFLEK----REESREV--	438
XP_026937670.1	-SPPPP--------PREA---SLAVR----GLLQELTSI--RHFLEK----REESREV--	438
XP_030691031.1	-SPPPP--------PREA---SLAVR----GLLQELTSI--RHFLEK----REESREV--	438
XP_022414870.1	-SPPPP--------PREA---SLAVR----GLLQELTSI--RHFLEK----REESREV--	443
XP_029064651.1	-SPPPP--------PREA---SLAVR----GLLQELTSI--RHFLEK----REESREV--	443
XP_024607566.1	-SPPPP--------PREA---SLAVR----GLLQELTSI--RHFLEK----REESREV--	443
XP_032496907.1	-SPPPP--------PREA---SLAVR----GLLQELTSI--RHFLEK----REESREV--	443
XP_007535559.1	-SPVPP--------PREA---SLAVR----GLLQELAAI--RHFLEK----REESREV--	436
XP_031299536.1	-SPPPP--------PREA---SLAMR----GLLQELTSI--RHFLEK----RDESREV--	442
XP_004666516.1	-GPPPP-------PRREA---SLAVR----GLLQELSSI--RHILEK----RDDIREV--	439
XP_008830963.1	-SPPPP--------PRET---SLAVR----GLLQELSSI--RHFLEK----RDEMREV--	443
XP_005347338.1	-SPPPP--------PREA---SLAVR----GLLQELASI--RHFLEK----RDEMREV--	443
XP_005069548.1	-SPPPP--------PREA---SLAVR----GLLQELSSI--RHFLEK----RDEMREV--	443
XP_027267894.1	-SPPPP--------PREA---SLAVR----GLLQELSSI--RHFLEK----RDEMREV--	443
OBS80992.1	-SPPPP--------PREA---SLAVR----GLLQELSSI--RHFLEK----RDEMREV--	431
XP_006979812.1	-SPPPP--------PREA---SLAVR----GLLQELSSI--RHFLEK----RDEMREV--	450
XP_028720687.1	-SPPPP--------PREA---SLAVR----GLLQELASI--RHFLEK----RDEMREV--	444
XP_021489005.1	-SPPPP--------PREA---SLAVR----GLLQELSSI--RHFLEK----RDEMREV--	444
XP_031199589.1	-SPLPP--------PREA---SLAVR----GLLQELSSI--RHFLEK----RDEMREV--	468
XP_028617944.1	-SPLPP--------PREA---SLAVR----GLLQELSSI--RHFLEK----RDEMREV--	258
XP_034347030.1	-SPLPP--------PREA---SLAVR----GLLQELSSI--RHFLEK----RDEMREV--	441
NP_077370.2	-SPLPP--------PREA---SLAVR----GLLQELSSI--RHSLEK----RDEMREV--	443
XP_032766961.1	-SPLPP--------PREA---SLAVR----GLLQELSSI--RHSLEK----RDEMREV--	443
XP_021063186.1	-SPLPP--------PRDA---SLAVR----GLLQELSSI--RHFLEK----RDEMREV--	426
NP_001093114.1	-SPLPP--------PREA---SLAVR----GLLQELSSI--RHFLEK----RDEMREV--	443
XP_021028379.1	-SPLPP--------PREA---SLAVR----GLLQELSSI--RHFLEK----RDEMREV--	443
XP_004689305.1	-SPPPP--------PREA---SLAVR----GLLQELSSI--RHYLEK----REESREV--	443
XP_004712871.1	-SPPPP--------PRDA---SLAVR----GLLQELSTI--RHFLEK----RDESREV--	443
XP_004382575.1	-SPPPP--------PRGA---SLAVR----GLLQELSNI--RHFLEK----RDESREV--	443
XP_010596188.1	-SPPPP--------PRGA---SLAVR----GLLQELSTI--RHFLEK----RDESREV--	443
XP_006890855.1	-SPPPP--------PREA---SLAVR----GLLHELSTI--RHFLEK----RDESREV--	443
XP_006834038.1	-SPPPP--------PREA---SFAVR----GVLRELSSI--RHFLEK----RDESREV--	443
XP_007934716.1	-CPPPP--------PREA---SLAVR----GLLQELSTI--RHFLEK----RDESREV--	443
XP_012603491.1	-SPPPP--------PRGA---SLAMR----GLLQELSSI--RHFLEK----RDEIREV--	443
XP_012663606.1	-SPAPP--------PQEA---SLAVT----GLLQELSSI--RRFLEK----RDEIREV--	438
XP_028372812.1	-SPPPP--------PREA---SLAVR----GLLQELASI--RHFLEK----REESREV--	443
XP_008589608.1	-SPPPP--------PREA---SLAVR----GLLQELSSI--RHFLEK----RDEIREV--	445
XP_012865077.1	-SPPPP--------PREA---SLAVR----GLLQELSSI--RHFLEK----REEIREM--	443
NP_001166178.1	-SPPPP--------PREA---SLAMC----GLLQELASI--RHFLEK----REETREV--	450
XP_004636553.1	-SPPPP--------PREG---SLAVR----GLLQELSSI--RHFLEK----REEIREV--	444
XP_004856670.1	-SPPPP--------PQEA---SLAVR----GLLQELSSI--RHFLEK----RDEIREV--	444
XP_010627744.1	-SPPPP--------PREA---SLAVR----GLLQELSSI--RHFLEK----RDEIREV--	444

Transmembrane domain 4

NP_509270.1	------RIIWQRFF----SWT-DIIFSIFFFV----VNCL-VT-FYMFMEFMF-------	434
VDO93178.1	------LLEWEFVA----TVL-DRVFLIMFTT----LSIT-TIAGILLMGFLGR-QVI--	491
PAV91580.1	------SLEWEFLA----TVL-DRFLLIIFIL----AVMI-VTSGLIVVGKMAQ-YSYDH	542
VDK46997.1	--	351
VIO86814.1	------PAYWKRII----RRL-ENISLTTYLF----LIIT-NVAMFMCHDLWY-------	532
VDP38785.1	------IRRWLYIA----RVV-DRLLFQVYLI----TTII-SIFVFLIYLPNSYDIKLNA	541
KOF68401.1	------GNKWKLFA----LFL-DRTFMFAHII----TFIL-TTAALLILCAYF-------	469
CDJ96026.1	------QWQWQQLA----SVV-DRLLLVLFSL----ATLF-TIFFFLLLPVG--LRDEDE	668
EFX76216.1	------KLEWQQVA----LVI-DRFLLWIFII----STVA-ATFGILYMSPHSRLFSM--	442
KAF7403848.1	------ELEWKQIA----LVS-DRILLGIFFL----MTVV-STAVILYGSPPTTESKNDD	465
KAF7427032.1	------ELEWKQIA----LVS-DRILLGIFFL----MTVV-STAVILYGSPPTTESKNDD	480
KAE9417558.1	------QADWKFMA----MVI-DRLSLFLFTV----LIVA-TTSLIFLSTPRMFSGSP--	461
KJH51504.1	------ALEWRFAA----MVV-DRLGLLAFST----LFTT-TTLIICLRAPYLVA-----	469
VDL69795.1	------EDDWKFAA----MVV-DRFCLFVFSF----FIVF-STLALFLSVPASKAFR---	433
VDO32671.1	------QADWKFAA----MAV-DRACLIMFSV----FIVL-SAIAILLSAPHIVA-----	462
VBB32409.1	------ANNWKFAA----IVV-DRLCLYIFTI----FIIA-SSCGILLSAPYFIA-----	482
VDN54565.1	------ANNWKFAA----MVV-DRLCLYIFTI----FIIA-SSCGILLSAPYIIA-----	500
EGT55171.1	------SNNWKFAA----MVV-DRLCLYVFTI----FIIA-STIGIFWSAPYLVA-----	514
RCN52111.1	------SNNWKFAA----MVV-DRLCLYVFTM----FILA-STIGIFSSAPYLVA-----	516
RMX54856.1	------KDEWNMVV----AIL-DRAFRMLFLI----MFFL-STLTIFYFSMV--------	565
XP_022781674.1	------KDEWNTAV----AIL-DRAFRMLFLL----MFFL-STLTIFYFSMV--------	826
RNA37099.1	------LEEWKQVA----SKV-DKLLFWIFLI----VTFV-FSLVCLVIVPSYQNSKLYE	619
KAA0187152.1	------LNEWRTVA----IAV-DRILFWVFFV----VTTV-SSVVFLLVLPLVKRAEYVR	754
GAU96593.1	------RKQWRQVA----QVL-DRCLFWIFTV----ATVS-STFILLVIVPLFGDMGIDE	553
XP_009043980.1	------VQEWQFVA----HVM-DRLLFWIFLF----VAFV-SSIVILVIKPLMKPDL---	444
VDM43573.1	------ANEWRQVA----QVI-DRLLFWVFLI----CTVI-ITLILLIIIPTVHRSMESD	579
XP_003140283.1	------TSEWRQVA----HVV-DRLLFWIFLI----CTVM-ITVILLVIIPIRYRSFDES	466
VDN82010.1	------TSEWRQVA----HVI-DRLLFWVFLT----CTLM-ITVILLVIIPIRYRSSDDS	491
VDO31501.1	------TSEWRQVA----HVI-DCLLFWVFLT----CTLM-ITVILLVIIPIRYRSSDDS	503
VDP19591.1	------SLEWKFAA----RVI-DRFCLVIFSV----CNIV-VTFAILCSAPNLIASFMP-	701
EDO32053.1	--	324
TRY67230.1	------ENDWKYAA----MVL-DRLSLVVFTF----LTLL-LSGACLVSAPQILVY----	475
KAF7391312.1	------INDWKFAA----MVV-DRLCLIIFTL----FTVL-ATVVILSSAPHIIVH----	502
GFG30449.1	--	462
KQS30083.1	--	397
EDW57583.2	------ANDWKFAA----MVV-DRLCLIIFTM----FTIL-ATIAVLLSAPHIIVS----	692
KNC22799.1	------ANDWKFAA----MVV-DRLCLIIFTM----FTIL-ATIAVLLSAPHIIVS----	501
RZF44856.1	------TNDWKFAA----MVV-DRLCLIVFTL----FTVI-ATIAVLFSAPHIIVT----	497
ENN76856.1	--	431
KAF5300392.1	------TRDWKFAA----MVV-DRLCLIIFTL----FTII-ATLAVLFSAPHIIVS----	525
CAB3239999.1	------SRDWKFAA----MVV-DRLCLIIFTL----FTII-ATLAVLLSAPHIMVS----	486
PCG77624.1	------SRDWKFAA----MVV-DRLCLIIFTL----FTII-ATLAVLLSAPHIMVS----	429
PZC79131.1	------SRDWKFAA----MVV-DRLCLIIFTL----FTII-ATLAVLLSAPHIMVS----	507
KAF4083067.1	------QDEWCHVG----YVL-DFLLFRVYLM----LITFYAFVIIFMWCIWMYQ-----	368
XP_009924659.1	------PHWKPQEQDPFLLVL-EKVLLYSHFF----LSLFFFATISVKWSS---------	421
XP_010123544.1	------PHRQP--QDPFLPVL-EKVLLYSHFF----LSLFFFAIISIKWSS---------	420
CBN81618.1	--	381
KAF3704230.1	------PGYWTRVA----KRF-NKVFFIGYVT----VVSMFLVFIFLKWNIV--------	457
RUS86578.1	----PQVNKYKLIG----RHI-DTIAFFIFLF----MWVS-VTLAFTIKLNS--------	466
XP_034309618.1	GTQMNPGEKWKTRA----RRL-DKILLLLNIL----TNIV-TI-CIF--ASFLA------	412
PIK58946.1	------KIQARTVA----LVM-DRLVSLVFIT----VFAV-LVIKTLIDFS--GNSGAQS	474
VUZ42516.1	------ASEWDAVG----LVV-DRYCFWVYLI----LIVVTGMITLIPPMPYEVQEDTPG	505
PAA51166.1	------AMEWRTFA----LIL-DRVFFSAYVM----AILI-AVISLI---PRTHPK----	536
TGZ55997.1	------AMEWRTLA----LIL-DRLFFFLYIA----TIGI-AVLVSV---PRSTEPVLKE	592
VDP48851.1	------A---	474
PVD38331.1	------VREWRIIS----CVT-DRVIFITYLL----INFI-GLTVIFLWQFNRKDLSPPE	509
VDK73355.1	------DEDWNYVA----MVL-DRLFLLLFSI----ACFA-GTITILLQVNLKQNLXXXX	472
VDD84963.1	------DEDWKYVA----TVF-DRLFLIIFSL----ACFI-GTVTIFIQAPTLYDTRVPV	495
VDK42196.1	------DEDWKYVA----MVL-DRLFLMIFSF----ACFL-GTVTILLQAPTLYDSREAI	428
KFD58288.1	------KNDWRYIA----NVL-DRLFLWTFSL----TFIS-GTCLIVLQAPSIHDSRSSL	509
OUC49089.1	------KDDWRYIA----NVL-DRLFLWTFSL----TFIS-GTCLIMLQAPSIHDSRHPI	508
EYC26492.1	------IEDWKYVS----VVM-DRVFLLLFTF----ACAL-GTVLIIARAPSIYDTTVPL	536
VDK27218.1	------IEDWKYIS----VVM-DRLFLILFTT----ACVI-GSIVIILRAPSIYDTTVAL	471
VDM17286.1	------RENWKHVT----SAI-DRVQLIIFTC----VTVS-GTLIILFSAPYIFAEIDQA	389
VDL91846.1	--	415
KAE9548540.1	------ANDWMFLS----MVL-DRLFLIIFSI----L-NA-GAFLVILQAPSLYDTREPL	539
PDM74087.1	------RDDWKYVA----MIL-DRLLLYVFFG----ITLG-GTIGILASSPYVFSSTNET	482
XP_024504322.1	------RDDWKYVG----MVI-DRLLLFVFFG----VTLG-GTFGVLLSSPTVLEHVNQK	495
PIO52336.1	------RDDWKYVA----MII-DRLLLYVFFG----ITVG-GTCGILFSAPYVFQGVNQR	481
TKR73865.1	------KDDWKYVA----MVI-DRLLLYVFFG----ITVG-GSCGILFSAPYMFKPLNQT	484
RLU23395.1	------REDWKYVA----MVI-DRLQLYIFFL----VTTA-GTVGILMDAPHIFEYVDQD	517
XP_002427906.1	------REDWKYVA----MVI-DRLQLFIFFI----VTTA-GTVGIIMDAPHIFDYVDQD	504
TMW47392.1	------REDWKYVA----MVI-DRLQLYLFFI----VTTA-GTIGILMDAPHIFEYVDQD	498
VDD83956.1	------RSEWRYVA----FVI-DRLLLWMFAG----ATMA-GIVSIFMQAPTLHTKERAL	479
VEL33078.1	K---	424
XP_009019088.1	RQRYPTIEDWKYMA----LVI-DRIFLWIYSL----VCLS-GTLIIICQAPMLYDTRVPV	497

```
KAE9536378.1        ------DQDWGFVA----MVL-DRLFLWIFTV----ASIM-GTILILCEAPALYDDTKPI    573
KDR23473.1          ------DQDWGFVA----MVL-DRLFLWIFTI----ASIV-GTFAILCEAPALYDDTKPI    515
KAF7266914.1        ------IEDWKFVS----MVL-DRFFLWVFTL----ACIT-GTCGIICQAPSLYDTRIPV    524
KAF4519445.1        ------------------------------------------------------------    400
OXU25983.1          ------IEDWKFVA----MVL-DRFFLWIFTL----ACII-GTAGIICQAPSLYDTREPL    512
TGZ32403.1          ------IEDWKFVA----MVL-DRFFLWVFTF----ACIG-GTLSIICQAPSLYDTREPV    518
ELT94491.1          ------KQDWQFVA----MVL-DRLFLWIFTA----ACIV-GTFGIIVQAPTLYDNKEPL    514
VVC39575.1          ------LE----------------------------------------------------    565
RVE49089.1          ------KEDWKYVA----MVL-DRLFLWIFTL----AVVV-GSAGIILQAPTLYDERAPI    514
KAB0800277.1        ------EEDWKYVA----MVL-DRLFLWLFTI----ACVT-GTALIIFKAPSLYDNTRPI    530
TDG52197.1          ------EEDWKYVA----MVL-DRMFLWIFAI----ACVV-GTALIILQAPSLYDQSQPI    530
TMW48669.1          ------------------------------------------------------------    335
XP_025896085.1      ------AHEWLQVA----YVV-DVLLFRAYLA----AVAAYGATLGALWAAWRCA-----    487
KAF2977017.1        ------VRDWRTTT----TVAIDLMVYAILSVDEKDQVLTTYIWYRQHWTDEFLKWDPAH    272
XP_009321837.1      ------AHEWLQVG----YVM-EVLLFRAYLA----AVLAYSITLGTLWSVWRDAWAAPA    468
XP_009979985.1      ------AREWLQVG----YVL-DVLLFRVYLV----AILAYSITLGSLWSVWRDA-----    431
XP_028942374.1      ------AREWLQVG----YVL-DVLLFRAYLA----AVLAYSLTLGILWSVWRDA-----    456
PKK19633.1          ------------------------------------------------------------    476
XP_009894240.1      ------------------------------------------------------------    500
XP_032820219.1      ------TGEWLQVG----HVL-DSLLFRLYLL----VIVMYTLTLGILWSIWSYSETL--    502
XP_029429480.1      ------GNEWLQVG----YVL-DVLLFRMYLV----TLLACGMTLGLLWAFWQQDLHDCT    506
XP_030077572.1      ------GKEWLEVG----YVL-DVLFFRMYLV----TLLANGIILGLMWSLGQQELRCS-    464
XP_033774596.1      ------GKKWLEVG----YVL-DVLFFWIYLV----ALLASGISLSLLWTLWQKELAAPK    488
ETE72600.1          ------ACEWLQVG----YVL-DVLLFRAYLV----AVLAYSITLGTLWSIWQYA-----    476
XP_018082638.1      ------AREWLQVG----YVL-DILLFRIYLV----AVLAYTVTLATMWSYWQQA-----    689
XP_032905410.1      ------AKEWLEVG----YVL-DVLLYRVYLL----VVAVYVIILSILWSVWQHS-----    468
XP_020773490.1      ------AKEWLQVG----YVL-DVLLFRVYLV----AMVTYSITLGTLWAVWQGA-----    496
XP_033833634.1      ------AKEWLQVG----YVL-DVLLFRVYLV----AMVTYSITLGTLWAVWQGA-----    505
XP_030215795.1      ------AKEWLQIG----YVL-DVLLFRVYLV----AMVAYSITLGTLWSVWQIV-----    522
XP_030602980.1      ------AKEWLQVG----YVL-DVLLFRVYLV----AVVAYSITLGTLWSVWQVA-----    498
XP_004573543.1      ------AKEWLQVG----YVL-DVLLFRVYLV----AVVAYSITLGTLWSVWQAA-----    491
XP_005916159.1      ------AKEWLQVG----YVL-DVLLFRVYLV----AVVAYSITLGTLWSVWQAA-----    492
XP_013132089.1      ------AKEWLQVG----YVL-DVLLFRVYLV----AVVAYSITLGTLWSVWQAA-----    492
XP_031603488.1      ------AKEWLQVG----YVL-DVLLFRVYLV----AVVAYSITLGTLWSVWQAA-----    484
XP_005725536.1      ------AKEWLQVG----YVL-DVLLFRVYLV----AVVAYSITLGTLWSVWQAA-----    484
XP_026038075.1      ------AKEWLQVG----YVL-DVLLFRVYLV----AVVAYSITLGTLWSVWQAA-----    498
CAF96649.1          ------AKEWLQVG----YVL-DVLLFRVYLV----AVVTYSITLGTLWSVWQVA-----    457
XP_023818570.1      ------AKEWLQVG----YVL-DVLLFRVYLV----AIVAYSITLGTLWSVWQVA-----    488
RVE64289.1          ------AKEWLQVG----YVL-DVLLFRVYLV----AMVAYSITLGTLWSVWQVA-----    523
XP_024121971.1      ------AKEWLQVG----YVL-DVLLFRVYLV----AMVAYSITLGTLWSVWQVA-----    494
XP_015817559.1      ------AKEWLQVG----YIL-DVLLFRVYLV----AVVAYSITLGTLWSVWQVA-----    478
XP_015225670.1      ------AKEWLQVG----YVL-DVLLFRVYLV----AVVAYSITLGTLWSVWQVA-----    398
XP_012722063.1      ------AKEWLQVG----YVL-DVLLFRVYLV----AVIAYSITLGTLWSVWQVA-----    492
XP_032431307.1      ------AKEWLQVG----YVL-DVLLFRVYLV----AVIAYSITLGTLWSVWQVA-----    492
XP_014328329.1      ------AKEWLQVG----YVL-DVLLFRVYLV----AVIAYSITLGTLWSVWQVA-----    492
XP_027886578.1      ------AKEWLQVG----YVL-DVLLFRVYLV----AVIAYSITLGTLWSVWQVA-----    492
XP_008426791.1      ------AKEWLQVG----YVL-DVLLFRVYLV----AVIAYSITLGTLWSVWQVA-----    492
XP_014838686.1      ------AKEWLQVG----YVL-DVLLFRVYLV----AVIAYSITLGTLWSVWQVA-----    492
XP_014885967.1      ------AKEWLQVG----YVL-DVLLFRVYLV----AVIAYSITLGTLWSVWQVA-----    492
XP_033954312.1      ------AKEWLQVG----YVL-DVLLFRVYLV----AVVAYSITLGTLWSVWQVA-----    498
XP_034089244.1      ------AKEWLQVG----YVL-DVLLFRVYLV----AVVAYSITLGTLWSVWQVA-----    487
XP_010780064.1      ------------------------------------------------------------    330
XP_033970261.1      ------AKEWLQVG----YVL-DVLLFRVYLV----AVVAYSITLGTLWSVWQVA-----    514
XP_029375575.1      ------AKEWLQVG----YVL-DVLLFRVYLV----AVVAYSITLGTLWSVWQVA-----    529
KAF0023022.1        ------AKEWLQVG----YVL-DVLLFRVYLV----AVVAYSITLGTLWSVWQVA-----    530
XP_019952614.1      ------AKEWLQVG----YVL-DVLLFRVYLV----AVVAYSITLGTLWSVWQVA-----    483
XP_034463117.1      ------AKEWLQVG----YVL-DVLLFRVYLV----AVVAYSITLGTLWSVWQVA-----    495
XP_029924656.1      ------AKEWLQVG----YVL-DVLLFRVYLV----AMVAYSITLGTLWSVWQIA-----    493
XP_028323228.1      ------AKEWLQVG----YVL-DVLLFRVYLL----VLVAYSITLGTLWCVWQGA-----    489
TNM84480.1          ------------------------------------------------------------    311
XP_029703788.1      ------AKEWLQVG----YVL-DVLLFRVYLV----AVVTYSITLGTLWSVWQVA-----    492
XP_030010368.1      ------AKEWLQVG----YVL-DVLLFRVYLV----SMVAYSITLGTLWSVWQVA-----    495
XP_019725056.1      ------AKEWLQVG----YVL-DVLLFRVYLV----AVVAYSITLGTLWSVWQVA-----    490
XP_034034934.1      ------AKEWLQVG----YVL-DVLLFRVYLV----AMVAYSITLGTLWSVWQGA-----    481
XP_029956880.1      ------AKEWLQVG----YVL-DVLLFRVYLV----AVVAYSITLGTLWSVWQVA-----    492
XP_008331307.3      ------AKEWLQVG----YVL-DVLLFRVYLV----AMVAYSITLGTLWSVWQVA-----    527
XP_031724845.1      ------AKEWLQVG----YVL-DVLLFRVYLV----AVVAYSITLGTLWSVWQVA-----    493
XP_013859395.1      ------AKEWLQVG----YVL-DVLLFRVYLV----AMVAYSITLGTLWSVWQVA-----    493
XP_017275335.1      ------AKEWLQVG----YVL-DILLFRVYLV----AMVAYSITLGTLWSVWQVA-----    493
XP_020496197.1      ------AKEWLQVG----YVL-DVLLFRVYLV----AVVAYSITLGTLWSVWQVA-----    491
XP_029030400.1      ------AKEWLQVG----YVL-DVLLFRVYLV----AVVAYSITLGTLWSVWQVA-----    492
XP_022060538.1      ------AKEWLQVG----YVL-DVLLFRVYLV----AMVAYSITLGTLWSVWQVA-----    494
XP_023150584.1      ------AKEWLQVG----YVL-DVLLFRVYLV----AMVAYSITLGTLWSIWQVA-----    494
XP_029303892.1      ------AKEWLQVG----YVL-DVLLFRVYLV----AVVAYSITLGTLWSVWQVA-----    516
XP_020454172.1      ------AKEWLQVG----YVL-DVLLFRVYLV----AMVAYSITLGTLWSVWQVA-----    491
TKS83244.1          ------AKEWLQVG----YVL-DVLLFRVYLV----AIVAYSITLGTLWSVWQVA-----    476
XP_022616583.1      ------AKEWLQVG----YVL-DVLLFRVYLV----AMVAYSITLGTLWSVWQVA-----    522
```

XP_030293172.1	------AKEWLQVG----YVL-DVLLFRVYLV----AMVAYSITLGTLWSVWQVA-----	494
XP_027129539.1	------AKEWLQVG----YVL-DVLLFRVYLV----AIVAYSITLGTLWSVWQVA-----	489
XP_034539538.1	------AKEWLQVG----YVL-DVLLFRVYLV----AVVAYSITLGTLWSVWQVA-----	492
XP_008277330.1	------AKEWLQVG----YVL-DVLLFRVYLV----AMVAYSITLGTLWSVWQVA-----	492
XP_028276940.1	------AKEWLQVG----YVL-DVLLFRVYLV----AMVAYSITLGTLWSVWQVA-----	492
XP_023263433.1	------AKEWLQVG----YVL-DVLLFRVYLV----AMVAYSITLGTLWSVWQVA-----	489
XP_034406336.1	------AKEWLQVG----YVL-DVLLFRVYLV----AVVAYSITLGTLWSVWQVA-----	492
XP_026184460.1	------AKEWLQVG----YVL-DVLLFRVYLV----AMVAYSITLGTLWSVWQVA-----	492
KAF1378228.1	------AKEWLQVG----YVL-DVLLFRVYLV----AVVAYSITLGTLWSVWQVA-----	477
XP_028450365.1	------AKEWLQVG----YVL-DVLLFRVYLV----AVVAYSITLGTLWSVWQVA-----	481
XP_031163851.1	------AKEWLQVG----YVL-DVLLFRVYLV----AVVAYSITLGTLWSVWQVA-----	492
XP_032389084.1	------AKEWLQVG----YVL-DVLLFRVYLV----AVVAYSITLGTLWSVWQVA-----	492
XP_033494682.1	------AKEWLQVG----YVL-DVLLFRVYLV----AVVAYSITLGTLWSVWQVA-----	492
XP_018544782.1	------AKEWLQVG----YVL-DVLLFRVYLV----AMVAYSITLGTLWSVWQVA-----	492
XP_026228189.1	------AKEWLQVG----YVL-DVLLFRVYLV----AMVAYSITLGTLWSVWQVA-----	492
XP_028976606.1	------AKEWLQVG----YVL-DVLLFRVYLV----AMLAYSITLGTLWSVWQYA-----	539
CAB1352378.1	------AKEWLQVG----YVL-DVLLFRVYLV----AMLAYSITLGTLWSVWQYA-----	450
XP_023866849.1	------AKEWLQVG----YVL-DVLLFRVYLV----AMLAYSITLGTLWSMYQYA-----	493
XP_013992832.1	------AKEWLQVG----YVL-DVLLFRVYLV----AMLAYSITLGTLWSVWQYA-----	493
XP_029546688.1	------AKEWLQVG----YVL-DVLLFRVYLV----AMLAYSITLGTLWSVWQYA-----	493
XP_021481546.1	------AKEWLQVG----YVL-DVLLFRVYLV----AMLAYSITLGTLWSVWQYA-----	493
XP_020339889.1	------AKEWLQVG----YVL-DVLLFRVYLV----AMLAYSITLGTLWSVWQYA-----	493
XP_029481972.1	------AKEWLQVG----YVL-DVLLFRVYLV----AMLAYSITLGTLWSVWQYA-----	493
KPP68743.1	------AKEWLQVG----YVL-DVLLFRVYLV----AVLAYSITLGTLWSVWQYA-----	459
XP_023669383.1	------AKEWLQVG----YVL-DVLLFRVYLV----AVLAYSITLGTLWSVWQYA-----	481
KAA0720096.1	------AKEWLKVG----YVL-DVLLFRVYMV----TVLAYTITLGTLWSVWQNA-----	388
XP_009293684.1	------AKEWLQVG----YVL-DILLFRVYLL----TVLAYGITLGSLWSIWQNV-----	482
XP_026090784.1	------AKEWLQVG----YVL-DILLFRVYLL----TVLAYSITLGTLWSVWQNV-----	482
XP_018918715.1	------AKEWLQVG----YVL-DVLLFRVYLL----TVLAYSITLGSLWSVWQNV-----	482
KAF4098549.1	------AKEWLQVG----YVL-DVLLFRVYLL----TVLAYSITLGTLWSVWQNV-----	482
XP_016084173.1	------AKEWLQVG----YVL-DVLLFRVYLL----TVLAYSITLGTLWSVWQNV-----	482
XP_016332759.1	------AKEWLQVG----YVL-DVLLFRVYLL----TVLAYSITLGTLWSVWQNV-----	482
XP_016398039.1	------AKEWLQVG----YVL-DVLLFRVYLL----TVLAYSITLGTLWSVWQNV-----	482
XP_026989970.1	------AKEWLKVG----YVL-DVLLFRVYLL----AMLAYTITLGTLWSVWQYEKSREM	486
TSQ12698.1	------AKEWLQVG----YVL-DILLFRVYLL----AMLAYTITLGTLWSVWQYA-----	482
XP_017347546.1	------AKEWLQVG----YVL-DVLLFRVYLL----AMLAYSITLGTLWSVWQYA-----	476
XP_026794616.2	------AKEWLQVG----YVL-DVLLFRVYLL----AMLAYSITLGTLWSVWQYA-----	482
XP_026861889.1	------AKDWLQVG----YVL-DVLLFRVYLL----AMLAYSITLGTLWSVWQYA-----	480
XP_017575347.1	------AKEWLQVG----YVL-DVLLFRVYLL----AMLAYSITLGTLWSVWQYA-----	480
XP_022531596.1	------AKEWLQVG----YVL-DVLLFRVYLL----AMLAYSITLGALWSVWQYA-----	480
XP_012691019.2	------AKEWLQVG----YVL-DVLLFRVYLV----AMLTYSITLGTLWSVWQYA-----	465
XP_028839723.1	------AKEWLQVG----YVL-DILLFRVYLV----AMLAYTITLGTLWSVWQYA-----	483
XP_030638860.1	------AKEWLQVG----YVL-DVLLFRIYLL----AMLTYSITLGTLWSVWQYA-----	463
XP_007882964.1	------AKEWLQVG----YVL-DTLLFRVYLL----GVLAYAITLGTLWAVWHYA-----	461
GCB70244.1	--	377
GCC26242.1	------AKEWLQIG----YVL-DVLLFRVYLL----AVLAYGISLGTLWSIWQYS-----	481
XP_020387034.1	------AKEWLQIG----YVL-DVLLFRVYLV----AVLAYAISLGTLWSIWQYS-----	472
XP_028931940.1	------SRDWLRVG----SVL-DGLLFRVYLF----AVLAYGVTLGALWSVWQYA-----	485
XP_031762490.1	------AKEWLQVG----YVL-DILLFRIYLV----AMLAYTVTLATMWSCWQQA-----	474
PIO32240.1	------AREWLQVG----YVL-DVLLFRIYLV----AVLAYTVTLATMWSYWQQA-----	272
XP_018425466.1	------AKEWLQVG----YVL-DVLLFRIYLV----AVLAYTVTLATMWSYWQHA-----	475
XP_006642351.1	------AKEWLQVG----YVL-DVLLFRVYLV----AVLAYSITLGTLWSVWQYA-----	498
XP_028665372.1	------AKEWLQVG----YVL-DVLLFRFYLV----AVLAYSITLGSLWSVWQYA-----	481
GCF49814.1	------AREWLQVG----YVL-DVLLFRAYLV----AVLAYSITLGTLWSIWQYA-----	460
XP_015277816.1	------AREWLQVG----YVL-DVLLFRAYLV----AVLAYSVTLGTLWSIWQYA-----	485
XP_005987340.2	------AREWLQVG----YVL-DVLLFRVYLV----AVLAYGITLGTLWSVWQYA-----	465
XP_019339534.1	------ACEWLQVG----YVL-DALLFRAYLV----AVLAYSVTLGTLWSIWQYA-----	487
XP_025063414.1	------ACEWLQVG----YVL-DALLFRAYLV----AVLAYSVTLGTLWSIWQYA-----	487
XP_019367488.1	------ACEWLQVG----YVL-DALLFRAYLV----AVLAYSVTLGMLWSIWQYA-----	487
XP_019412078.1	------ACEWLQVG----YVL-DALLFRAYLV----AVLAYSVTLGVLWSIWQYA-----	487
XP_014434314.1	------AREWLQVG----YVL-DVLLFRAYLV----AVLAYSITLGTLWSVWQYA-----	493
XP_007060556.1	------AREWLQVG----YVL-DVLLFRVYLV----AVLAYSITLGTLWSIWQYA-----	481
XP_024072175.1	------AREWLQVG----YVL-DVLLFRVYLV----AVLAYSITLGTLWSVWQYA-----	471
XP_008170567.1	------AREWLQVG----YVL-DVLLFRVYLV----AVLAYSITLGTLWSVWQYA-----	487
XP_034610543.1	------AREWLQVG----YVL-DVLLFRVYLV----AVLAYSITLGTLWSVWQYA-----	487
XP_030394127.1	------AREWLQVG----YVL-DVLLFRVYLV----AVLAYSITLGTLWSVWQYA-----	484
XP_032651960.1	------AREWLQVG----YVL-DVLLFRVYLV----AVLAYSITLGTLWSVWQYA-----	487
XP_025020083.1	------AREWLQVG----YVL-DVLLFRAYLV----AVLAYSITLGTLWSIWQYA-----	484
XP_026536833.1	------ACEWLQVG----YIL-DVLLFRAYLV----AVLAYSITLGILWSIWQYA-----	484
XP_026564787.1	------ACEWLQVG----YIL-DVLLFRAYLV----AVLAYSITLGILWSIWQYA-----	484
XP_032084675.1	------AREWLQVG----YVL-DVLLFRAYLV----AVLAYSITLGTLWSIWQYA-----	469
XP_029139341.1	------ACEWLQVG----YVL-DVLLFRAYLV----AVLAYSITLGTLWSIWQYA-----	484
XP_034281006.1	------ACEWLQVG----YVL-DVLLFRAYLV----AVLAYSITLGTLWSIWQYA-----	484
XP_020649062.1	------AREWLQVG----YVL-DVLLFRTYLV----AVLAYSVTLGTLWSIWQYA-----	484
XP_008117087.1	------AREWLQVG----YVL-DVLLFRAYLV----AVLAYSLTLGTLWSIWQYA-----	484
XP_028564324.1	------AREWLQVG----YVL-DVLLFRAYLV----AVLAYSITLGTLWSIWQYA-----	490
XP_033028155.1	------AREWLQVG----YIL-DVLLFRAYLV----AVLAYSITLGTLWSIWQYA-----	489

```
XP_025963249.1      ------AHEWLQVG----YVL-DALLFRAYLL----AVVAYSVTLGTLWSVWQYA-----     471
XP_009668348.1      ------AREWLQVG----YVL-DVLLFRAYLV----AVLAYSVTLGTLWSVWQHA-----     486
XP_013798935.1      ------AHEWLQVG----YVL-DVLLFRAYLV----AILAYSITLGTLWSVWQYA-----     470
XP_025913685.1      ------AHEWLQVG----YVL-DVLLFRAYLV----AILAYSITLGTLWSVWQYA-----     495
XP_013042552.1      ------AREWLQVG----YVL-DVLLFRAYLA----AVLAYTITLGALWSVWQYA-----     493
XP_005030458.2      ------AREWLQVG----YVL-DVLLFRAYLL----AVLAYTITLGALWSVWQYA-----     486
XP_032057953.1      ------AREWLQVG----YVL-DVLLFRAYLL----AVLAYTITLGALWSVWQYA-----     469
XP_021232050.1      ------------------------------------------------------------     408
OXB62403.1          ------AREWLQVG----YVL-DVLLFRAYLV----AVLAYTITLGTLWSVWQYA-----     487
OXB81319.1          ------AREWLQVG----YVL-DVLLFRAYLV----AVLAYTITLGTLWSVWQYA-----     469
XP_010722007.1      ------AREWLQVG----YVL-DVLLFRAYLV----AVLAYTVTLGTLWSVWQYA-----     488
XP_015739349.1      ------AREWLQVG----YVL-DVLLFRAYLV----AVLAYTITLGTLWSVWQYA-----     488
XP_031455498.1      ------AREWLQVG----YVL-DVLLFRAYLV----AVLAYTVTLGTLWSVWQYA-----     489
POI27435.1          ------AREWLQVG----YVL-DVLLFRAYLV----AVLAYTITLGTLWSVWQYA-----     464
XP_004948120.1      ------AREWLQVG----YVL-DVLLFRAYLV----AVLAYTITLGTLWSVWQYA-----     489
XP_032851190.1      ------GGSPITPG----GTP---------------------------------------     447
XP_010007255.1      ------AREWLQVG----YVL-DVLLFRAYMV----AVLAYSITLGTLWSVWRDA-----     480
XP_030320702.1      ------AREWLQVG----YVL-DILLFRAYLL----AVLAYTITLGTLWSVWRDA-----     445
XP_010191940.1      ------------------------------------------------------------     417
XP_027737112.1      ------AREWLQVG----YVL-DVLLFRAYLV----AVLAYSITLATLWSVWRDV-----     482
XP_027555032.1      ------AREWLQVG----YVL-DVLLFRAYLV----AVLAYSITLGTLWSVWRDA-----     487
XP_032565370.1      ------AREWLQVG----YVL-DVLLFRAYLV----AVLAYSITLGTLWSVWRDA-----     533
XP_027511217.1      ------AREWLQVG----YVL-DVLLFRAYLV----AVLAYSITLGTLWSVWRDA-----     632
XP_027593499.1      ------AREWLQVG----YVL-DVLLFRAYLV----AVLAYSITLGTLWSVWRDA-----     583
XP_017664924.1      ------AREWLQVG----YVL-DVLLFRAYLV----AVLAYSITLGTLWSVWRDA-----     484
XP_029817938.1      ------AREWLQVG----YVL-DVLLFRAYLV----AVLAYSITLGTLWSVWRDA-----     466
XP_005058641.1      ------AREWLQVG----YVL-DILLFRVYLA----AVLAYSITLGTLWSVWRDA-----     519
XP_021385804.1      ------AREWLQVG----YVL-DVLLFRVYLA----AVLAYSITLGTLWSVWRDA-----     484
XP_030146687.2      ------AREWLQVG----YVL-DVLLFRVYLA----AVLAYSITLGTLWSVWRDA-----     531
KAF4796420.1        ------AREWLQVG----YVL-DILLFRVYLA----AVLAYSITLGTLWSVWRDA-----     486
XP_032937581.1      ------AREWLQVG----YVL-DILLFRVYLA----AVLAYSITLGTLWSVWRDA-----     533
XP_031989659.1      ------AREWLQVG----YVL-DVLLFRVYLA----AVLAYSITLGTLWSVWRDA-----     487
XP_010402086.1      ------AREWLQVG----YVL-DVLLFRVYLA----AVLAYSITLGTLWSVWRDA-----     486
XP_017594069.1      ------AREWLQVG----YVL-DVLLFRVYLA----AVLAYSITLGTLWSVWRDA-----     457
XP_014115268.1      ------AREWLQVG----YVL-DVLLFRVYLA----AVLAYSITLGTLWSVWRDA-----     511
XP_023797108.1      ------AREWLQVG----YVL-DVLLFRVYLA----AVLAYSITLGTLWSVWRDA-----     457
XP_033375614.1      ------AREWLQVG----YVL-DVLLFRVYLA----AVLAYSITLGTLWSVWRDA-----     457
XP_014740121.1      ------AREWLQVG----YVL-DVLLFRVYLA----AVLAYSITLGTLWSVWRDA-----     631
RLV83430.1          ------AREWLQVG----YVL-DVLLFRVYLA----AVLAYSITLGTLWSVWRDA-----     477
XP_009096098.2      ------AREWLQVG----YVL-DVLLFRVYLA----AVLAYSITLGTLWSVWRDA-----     593
TRZ15870.1          ------AREWLQVG----YVL-DVLLFRVYLA----AVLAYSITLGTLWSVWRDA-----     487
RMB91935.1          ------AREWLQVG----YVL-DVLLFRAYLA----AVLAYSITLGTLWSVWRDA-----     457
XP_030820843.1      ------AREWLQVG----YVL-DVLLFRVYLA----AVLAYSITLGTLWSVWRDA-----     533
XP_014165179.1      ------AREWLQVG----YVL-DVLLFRVYLA----AVLAYSITLGTLWSVWRDA-----     535
XP_026653582.1      ------ARDWLQVG----YVL-DVLLFRVYLA----AVLAYSITLGTLWSVWRDA-----     417
PKU35975.1          ------AREWLQVG----YVL-DVLLFRAYLV----AVLAYTITLGTLWSVWRDA-----     488
XP_014805072.1      ------AREWLQVG----YVL-DVLLFRAYLV----AVLAYTITLGTLWSVWRDA-----     480
XP_009818330.1      ------AREWLQVG----YVL-DVLLFRAYLA----AVLAYSITLGTLWSVWRDA-----     198
OPJ68307.1          ------ARDWLQVG----YVL-DVLLFRAYLA----AVLAYSVTLGTLWSVWRDA-----     485
XP_008936289.1      ------AHEWLQVG----YVL-DVLLFRVYLV----AVLAYSITLGTLWSVWRDA-----     487
XP_010287046.1      ------------------------------------------------------------     374
XP_005240140.2      ------AREWLQVG----YVL-DVLLFRAYLV----AVLAYSITLGTLWSVWRDV-----     540
XP_005437752.2      ------AREWLQVG----YVL-DVLLFRAYLV----AVLAYSITLGTLWSVWRDV-----     546
KFV74811.1          ------AREWLQVG----YVL-DVLLFRAYLV----AVLAYSITLGTLWSVWRDA-----     446
XP_010018389.1      ------AREWLQVG----YVL-DVLLFRAYLA----AVLAYTITLGTLWSVWRDA-----     493
KQK78711.1          ------ACEWLQVG----YVL-DVLLFRAYLV----AILAYSITLGTLWSVWRDA-----     486
XP_009570162.1      ------AHEWLQVG----YVL-DVLLFRAYLV----AVLSYSVTLGTLWSVWRDA-----     485
KFP11268.1          ------ARE---------------------------------------------------     412
KFQ98910.1          ------AREWLQVG----YVL-DVLLFRAYLA----AVLAYSVTLGTLWSVWRDA-----     468
XP_012985202.3      ------AREWLQVG----YVL-DVLLFRAYLV----AVLAYTITLGTLWSVWRDA-----     486
XP_010573388.1      ------AREWLQVG----YVL-DVLLFRAYLA----AVLAYSITLGTLWSVWRDA-----     485
XP_029879496.1      ------AREWLQVG----YVL-DVLLFRAYLA----AVLAYSITLGTLWSVWRDA-----     457
XP_030361086.1      ------AREWLQVG----YVL-DVLLFRAYLV----AVLAYTITLGTLWSVWRDA-----     538
KFM00668.1          ------AHEWLQVG----YVL-DVLLFRAYLA----AVLAYSITLGTLWSVWRDA-----     469
KAF1479074.1        ------AHEWLQVG----YVL-DVLLFRAYLA----AVLAYSITLGTLWSVWRDA-----     449
KAF1651161.1        ------AHEWLQVG----YVL-DVLLFRAYLA----AVLAYSITLGTLWSVWRDA-----     450
KAF1673648.1        ------AHEWLQVG----YVL-DVLLFRAYLA----AVLAYSITLGTLWSVWRDA-----     450
KAF1493319.1        ------AHEWLQVG----YVL-DVLLFRAYLA----AVLAYSITLGTLWSVWRDA-----     450
KAF1584157.1        ------AHEWLQVG----YVL-DVLLFRAYLA----AVLAYSITLGTLWSVWRDA-----     450
KAF1571723.1        ------AHEWLQVG----YVL-DVLLFRAYLA----AVLAYSITLGTLWSVWRDA-----     450
KAF1533169.1        ------AHEWLQVG----YVL-DVLLFRAYLA----AVLAYSITLGTLWSVWRDA-----     450
KAF1638955.1        ------AHEWLQVG----YVL-DVLLFRAYLA----AVLAYSITLGTLWSVWRDA-----     450
KAF1549972.1        ------AHEWLQVG----YVL-DVLLFRAYLV----AVLAYSITLGTLWSVWRDA-----     450
KAF1606914.1        ------AHEWLQVG----YVL-DVLLFRAYLV----AVLAYSITLGTLWSVWRDA-----     450
KAF1510957.1        ------AHEWLQVG----YVL-DVLLFRAYLA----AVLAYSITLGTLWSVWRDA-----     450
KAF1498899.1        ------AHEWLQVG----YVL-DVLLFRAYLA----AVLAYSITLGTLWSVWRDA-----     450
KAF1411525.1        ------AHEWLQVG----YVL-DVLLFRAYLA----AVLAYSITLGTLWSVWRDA-----     450
```

KAF1429205.1	------AHEWLQVG----YVL-DVLLFRAYLA----AVLAYSITLGTLWSVWRDA-----	450
XP_005334318.1	------ARDWLRVG----SVL-DKLLFRIYLL----AVLAYSITLVTLWSIWHYS-----	472
XP_014395552.1	------ARDWLRVG----SVL-DRLLFRIYLL----AVLAYSVTLVTLWSIWQYS-----	472
KAB0404854.1	------CKAKENNA----TGL-GR------------------------------------	448
XP_007494828.1	------ARDWLRVG----SVL-DVLLFRIYLL----AVLAYSITLGTLWSIWQYA-----	488
XP_003764254.1	------ARDWLRVG----SVL-DVLLFRIYLL----AVLAYSITLGTLWSIWQYA-----	488
XP_020845489.1	------ARDWLLVG----SVL-DVLLFRIYLL----AVLAYSITLGTLWSIWQYA-----	488
XP_027703160.1	------ARDWLRVG----SVL-DVLLFRIYLL----AVLTYSITLGTLWSIWQYA-----	488
XP_017523929.1	------ARDWLRVG----SAL-DRLLFRIYLL----VVLVYGATLVALWSIWQCS-----	480
XP_008688428.1	------AREWLRVG----SVL-DRLLFRIYLV----AVLAYSVTLITLWSIWQYS-----	428
XP_029812166.1	------AREWLHVG----SVL-DRLLFRIYLV----AMLAYGVTLVTLWSVWQCS-----	497
XP_025749781.1	------AREWLRVG----SVL-DRLLFRIYLV----AVLAYSITLIIILWSIWQYS-----	483
XP_004416432.1	------AREWLRVG----SVL-DRLLFRIYLV----AVLAYSITLIIILWSIWQYS-----	483
XP_027436262.1	------AREWLRVG----SVL-DRLLFRIYLV----AVLAYSITLIIILWSIWQYS-----	483
XP_032284025.1	------AREWLRVG----SVL-DRLLFRIYLV----AVLAYSITLIVLWSVWQDS-----	483
XP_006735421.1	------AREWLRVG----SVL-DRLLFRIYLV----AVLAYSITLIIILWSVWQYS-----	483
XP_021552166.1	------AREWLRVG----SVL-DRLLFRIYLV----AVLAYSITLIIILWSVWQYS-----	483
XP_026361066.1	------AREWLRVG----SVL-DRLLFRIYLV----AVLAYSVTLVTLWSIWQYS-----	483
XP_034523598.1	------AREWLRVG----SVL-DRLLFRIYLV----AVLAYSITLITLWSIWQYS-----	483
NP_001297113.1	------AREWLRVG----SVL-DRLLFRVYLV----AVLAYSVTLIALWSIWQYS-----	483
XP_032215488.1	------AREWLRVG----SVL-DRLLFRVYLV----AVLAYSVTLIALWSIWQYS-----	483
VCX31483.1	------AREWLRVG----SVL-DRLLFRVYLV----AVLAYSVTLITLWSIWQYS-----	483
XP_022369003.1	------AREWLRVG----SVL-DRLLFRVYLV----AVLAYSITLITLWSVWQYS-----	482
XP_032694248.1	------AREWLRVG----SVL-DRLLFRVYLV----AVLAYSVTLITLWSIWQYS-----	482
NP_001041584.1	------AREWLHVG----SVL-DRLLFRIYLV----AVLAYSITLITLWSIWQYS-----	483
XP_025862501.1	------AREWLHVG----SVL-DRLLFRIYLV----AVLAYSITLITLWSIWQYS-----	483
KAF0873564.1	------AREWLHVG----SVL-DRLLFRIYLV----AVLAYSITLVTLWSIWQYS-----	625
XP_025784751.1	------AREWLHVG----SVL-DGLLFRIYLV----AVLAYSITLVTLWSIWQYS-----	477
XP_007075625.1	------AREWLHVG----SVL-DGLLFRIYLV----AVLAYSITLVTLWSIWQYS-----	619
XP_030189489.1	------AREWLHVG----SVL-DGLLFRIYLV----AVLAYSITLVTLWSIWQYS-----	483
XP_019324655.1	------AREWLHVG----SVL-DGLLFRIYLV----AVLAYSITLVTLWSIWQYS-----	584
XP_023094886.1	------AREWLHVG----SVL-DRLLFRIYLV----AVLAYSITLVTLWSIWQYS-----	619
XP_026892357.1	------AREWLHVG----SVL-DGLLFRIYLV----AVLAYSITLVTLWSIWQYS-----	483
XP_016004457.1	------ARDWLHVG----CVL-DRLLFRIYLL----AVLAYGVTLVTLWSIWQHS-----	478
XP_006912860.1	------ARDWLRVG----TML-DGLLFRIYLL----AVLAYGVTLVTLWSIWQYS-----	478
XP_011363679.1	------ARDWLRVG----TVL-DGLLFRIYLL----AVLAYGVTLVMLWSIWQYS-----	478
XP_016076060.1	------ARDWLRVG----SVL-DRLLFRIYLL----AVLAYSVTLVTLWSIWQYS-----	483
XP_008148371.1	------ARDWLRVG----SVL-DRLLFRIYLL----AVLAYSLTLVTLWSIWQYS-----	483
XP_006093568.1	------ARDWLRVG----SVL-DRLLFRIYLL----AVLAYSVTLVTLWSIWQYS-----	483
XP_006761601.1	------ARDWLRVG----SVL-DRLLFRIYLL----AVLAYSATLVTLWSIWKYS-----	483
XP_024426743.1	------ARDWLRVG----SVL-DRLLFRIYLL----AVLAYSVTLVTLWSIWQYS-----	478
XP_019520779.1	------ARDWLRVG----SVL-DRLLFRIYLL----AVLAYSVTLVTLWSIWQYS-----	483
XP_032976539.1	------ARDWLRVG----SVL-DRLLFRIYLL----AVLAYSITLVTLWSIWQYS-----	483
ELW64270.1	------ARDWLRVG----SVL-DRLLFRIYLL----AVLAYSATLVTLWSIWQYS-----	487
XP_004427367.1	------ARDWLYVG----SVL-DRLLFRLYLL----AVLAYSVTLVTLWSIWQYS-----	483
XP_008529353.1	------ARDWLHVG----SVL-DRLLFRIYLL----AMLAYSVTLVTLWSIWRYS-----	488
NP_001288165.1	------ARDWLHVG----SVL-DRLLFRIYLL----AMLAYSVTLVTLWSIWRYS-----	483
XP_014711213.1	------ARDWLHVG----SVL-DRLLFRIYLL----AMLAYSVTLVTLWSIWQYS-----	483
XP_012514621.1	------ARDWLQVG----SVL-DKLLFRIYLM----VVLTYSVTLVTLWSIWHYS-----	483
XP_008071525.1	------ARDWLRVG----SVL-DKLLFRTYLL----AVLAYSITLVTLWSIWQYS-----	480
XP_009005082.1	------ARDWLHVG----SVL-DKLLFRIYLL----AVLAYSITLVTLWSVWQYA-----	483
XP_010332832.1	------ARDWLHVG----SVL-DKLLFRIYLL----AVLAYSITLVTLWFIWQYA-----	478
XP_012326058.1	------ARDWLHVG----SVL-DKLLFRIYLL----AVLAYSITLVTLWSIWQYA-----	489
XP_017378796.1	------ARDWLHVG----SVL-DKLLFRIYLL----AVLAYSITLVTLWSIWQYA-----	512
XP_032141374.1	------ARDWLHVG----SVL-DKLLFRIYLL----AVLAYSITLVTLWSIWQYA-----	512
XP_032024018.1	------ARDWLRVG----SVL-DKLLFHIYLL----AVLAYSITLVMLWSIWQYA-----	478
XP_018891497.2	------ARDWLRVG----SVL-DKLLFHIYLL----AVLAYSITLVMLWSIWQYA-----	484
XP_030684894.1	------ARDWLRVG----SVL-DKLLFHIYLL----AVLAYSITLVMLWSIWQCA-----	478
PNJ75904.1	------ARDWLRVG----SVL-DKLLFHIYLL----AVLAYSITLVMLWSIWQYA-----	484
AAP35868.1	------ARDWLRVG----SVL-DKLLFHIYLL----AVLAYSITLVMLWSIWQYA-----	484
XP_001149570.1	------ARDWLRVG----SVL-DKLLFHIYLL----AVLAYSITLVILWSIWQYA-----	484
XP_003805532.1	------ARDWLRVG----SVL-DKLLFHIYLL----AVLAYSITLVILWSIWQYA-----	484
XP_023063823.1	------ARDWLRVG----SVL-DKLLFHIYLL----AVLAYGITLVMLWSIWQYA-----	482
XP_033060210.1	------ARDWLRVG----SVL-DKLLFHIYLL----AVLAYSITLVMLWSIWQYA-----	482
XP_010370669.1	------ARDWLRVG----SVL-DRLLFHIYLL----AVLAYSITLVMLWSIWQYA-----	482
XP_017738496.1	------ARDWLRVG----SVL-DRLLFHIYLL----AVLAYSITLVMLWSIWQYA-----	482
XP_011782168.1	------ARDWLRVG----SVM-DKLLFHIYLL----AVLAYGITLVMLWSIWQYA-----	483
EHH23440.1	------ARDWLRVG----SVL-DKLLFHIYLL----AVLAYSITLVMLWSIWQYA-----	510
XP_003910778.3	------ARDWLRVG----SVL-DKLLFHIYLL----AVLAYSITLVMLWSIWQYA-----	483
XP_025212649.1	------ARDWLHVG----SVL-DKLLFHIYLL----AVLAYSITLVMLWSIWQYA-----	489
XP_008019132.1	------ARDWLRVG----SVL-DKLLFHIYLL----AVLAYSITLVMLWSIWQYA-----	483
XP_011832421.1	------ARDWLRVG----SVL-DKLLFHIYLL----AVLAYSITLVMLWSIWQYA-----	468
XP_011759618.1	------ARDWLRVG----SVL-DKLLFHIYLL----AVLAYSITLVMLWSIWQYA-----	483
XP_005579749.1	------ARDWLRVG----SVL-DKLLFHIYLL----AVLAYSITLVMLWSIWQYA-----	483
XP_011921684.1	------ARDWLRVG----SVL-DKLLFHIYLL----AVLAYSITLVMLWSIWQYA-----	275
XP_008259391.1	------ARDWLRVG----SVL-DKLLFRIYLL----AVLAYTITLVSLWSIWQYS-----	483
XP_012782438.1	------ARDWLRVG----SVL-DKLLFRIYLL----AVLAYTITLVSLWSVWQYS-----	479

```
VTJ86076.1          ------ARDWLRVG----SVL-DKLLFRIYLL----AVLAYSITLVTLWSIWHYS-----     489
XP_015345533.1      ------ARDWLRVG----SVL-DKLLFRIYLL----AVLAYSITLVTLWSIWHYS-----     489
XP_027786432.1      ------ARDWLRVG----SVL-DKLLFRIYLL----AVLAYSITLVTLWSIWHYS-----     489
XP_026248345.1      ------ARDWLRVG----SVL-DKLLFRIYLL----AVLAYSITLVTLWSVWHYS-----     483
XP_005378209.1      ------ARDWLRVG----SVL-DKLLFRIYLL----AVLAYSVTLVTLWSVWHYS-----     484
XP_004466010.1      ------ARDWLRVG----SVL-DRLLFRIYLL----AVLAYTATLLTLWSIWQYS-----     483
XP_020035707.1      ------ARDWLRVG----SVL-DKLLFRIYLL----AVLAYSITLVTLWSIWNYS-----     477
XP_006181802.1      ------ARDWLRVG----SLL-DRLLFRIYLL----AVLAYSVTLVTLWSIWQYS-----     483
XP_006207862.1      ------ARDWLHVG----SVL-DKLLFRIYLL----AVLAYSVTLVTLWSIWQYS-----     483
XP_005891968.1      ------ARDWLRVG----SVL-DQLLFRIYLL----AVLAYSITLVTLWSIWQYS-----     480
XP_010828662.1      ------ARDWLRVG----SVL-DRLLFRIYLL----AVLAYSITLVTLWSIWQYS-----     480
XP_019830126.1      ------ARDWLRVG----SVL-DRLLFRIYLL----AVLAYSITLVTLWSIWQYS-----     479
XP_017914486.1      ------ARDWLRVG----SVL-DRLLFRIYLL----AVLAYSVTLVTLWSIWQYS-----     479
XP_006060266.2      ------ARDWLRVG----SVL-DRLLFRIYLL----AVLAYSITLVTLWSIWQYS-----     484
XP_020728447.1      ------ARDWLRVG----SVL-DRLLFRIYLL----AVLAYSITLVTLWSIWQYS-----     484
KAF4008892.1        ------ARDWLRVG----SVL-DRLLFRIYLL----AVLAYSITLVTLWSIWQYS-----     484
KAB0348059.1        ------ARDWLRVG----SVL-DRLLFRIYLL----AVLAYSITLVTLWSIWQYS-----     467
KAB0371113.1        ------ARDWLRVG----SVL-DRLLFRIYLL----AVLAYSITLVTLWSIWQYS-----     460
MBV96963.1          ------ARDWLRVG----SVL-DKLLFRIYLL----AVLAYSVTLVTLWSIWQYS-----     483
XP_007172048.1      ------ARDWLRVG----FVL-DKLLFRIYLL----AVLAYSVTLVTLWSVWQYS-----     483
XP_007452270.1      ------ARDWLRVG----SVL-DRLLFRIYLL----AVLAYSVTLVTLWSIWQYS-----     483
XP_023987108.1      ------ARDWLRVG----SVL-DRLLFRIYLL----ALLAYSVTLVTLWSIWQYS-----     478
XP_004273432.1      ------AREWLRVG----SVL-DRLLFRIYLL----AVLAYGVTLVTLWSVWQYS-----     478
XP_004328646.1      ------AREWLRVG----SVL-DRLLFRIYLL----AVLAYGVTLVTLWSVWQYS-----     478
XP_026937670.1      ------AREWLHVG----SVL-DRLLFRIYLL----AVLAYGVTLVTLWSVWQYS-----     478
XP_030691031.1      ------AREWLRVG----SVL-DRLLFRIYLL----AVLAYGVTLVTLWSVWQYS-----     478
XP_022414870.1      ------AREWLRVG----SVL-DRLLFRIYLL----AVLAYSVTLVTLWSIWQYS-----     483
XP_029064651.1      ------AREWLRVG----SVL-DRLLFRIYLL----AVLAYSVTLVTLWSIWQYS-----     483
XP_024607566.1      ------AREWLRVG----SVL-DRLLFRIYLL----AVLAYSVTLVMLWSIWQYS-----     483
XP_032496907.1      ------AREWLRVG----SVL-DRLLFRIYLL----AVLAYSVTLVMLWSIWQYS-----     483
XP_007535559.1      ------ARDWLRVG----SVL-DRLLFRIYLL----AVLAYSVTLVTLWSIWQYS-----     476
XP_031299536.1      ------ARDWLRVG----SLL-DRLLFRIYLL----AVLAYSVTLVTLWSIWQYS-----     482
XP_004666516.1      ------ARDWLRVG----SVL-DRLLFRIYLL----AVLAYSITLVTLWSIWHYS-----     479
XP_008830963.1      ------ARDWLRVG----YVL-DRLLFRIYLL----AVLAYSITLVTLWSIWHYS-----     483
XP_005347338.1      ------ARDWLRVG----YVL-DRLLFRIYLL----AVLVYSITLVTLWSVWHYSRVNTT     488
XP_005069548.1      ------ARDWLQVG----YVL-DRLLFRIYLL----AVLAYSITLITLWSIWHYS-----     483
XP_027267894.1      ------ARDWLRVG----YVL-DRLLFRIYLL----AVLAYSITLITLWSIWHYS-----     483
OBS80992.1          ------ARDWLRVG----YVL-DRLXFRIYLL----AVLAYSITLVTLWSIWHYS-----     471
XP_006979812.1      ------ARDWLRVG----YVL-DRLLFRIYLL----AVLAYSITLVTLWSIWHYS-----     490
XP_028720687.1      ------ARDWLRVG----YVL-DRLLFRIYLL----AVLAYSITLVTLWSIWHYS-----     484
XP_021489005.1      ------ARDWLRVG----YVL-DRLLFRIYLL----AVLAYSITLVTLWSIWRYS-----     484
XP_031199589.1      ------AKDWLRVG----YLL-DRLLFRIYLL----AVLAYSITLVTLWSIWHYS-----     508
XP_028617944.1      ------AKDWLRVG----YVL-DKLLFRIYLL----AVLAYSITLVTLWSIWHYS-----     298
XP_034347030.1      ------ARDWLRVG----YVL-DRLLFRIYLL----AVLAYSVTLVTLWSIWHYS-----     481
NP_077370.2         ------ARDWLRVG----YVL-DRLLFRIYLL----AVLAYSITLVTLWSIWHYS-----     483
XP_032766961.1      ------ARDWLRVG----YVL-DRLLFRIYLL----AVLAYSITLVTLWSIWHYS-----     483
XP_021063186.1      ------ARDWLRVG----YVL-DRLLFRIYLL----AVLAYGITLVMLWSIWHYS-----     466
NP_001093114.1      ------ARDWLRVG----YVL-DRLLFRIYLL----AVLAYSITLVTLWSIWHYS-----     483
XP_021028379.1      ------ARDWLRVG----YVL-DRLLFRIYLL----AVLAYSITLVTLWSIWHYS-----     483
XP_004689305.1      ------ARDWLQVG----AVL-DRLLFCIYLL----AVLAYSVTLVTLWSVWQYS-----     483
XP_004712871.1      ------ARDWLRVG----SVL-DRLLFRIYLL----AVLAYSITLVTLWSIWQYS-----     483
XP_004382575.1      ------ARDWLRVG----SVL-DRLLFRIYLL----AVLAYTITLVTLWSIWQYS-----     483
XP_010596188.1      ------ARDWLRVG----SVL-DRLLFRIYLL----AVLAYTITLVTLWSIWQYS-----     483
XP_006890855.1      ------ARDWLRVG----SVL-DRLLFRIYLL----AVLAYSITLVTLWSIWQYS-----     483
XP_006834038.1      ------ARDWLRVG----SVL-DRLLFRVYLL----AVLAYSITLVTLWSIWKYS-----     483
XP_007934716.1      ------ARDWLRVG----SVL-DRLLFRIYLL----AVLTYSITLITLWSIWQYS-----     483
XP_012603491.1      ------ARDWLQVG----CVL-DKLLFRIYLM----VVLAYSVTLVTLWSIWQYS-----     483
XP_012663606.1      ------ARDWLQVG----SVL-DKLLFRIYLL----VVLAYSVTLVMLWSIWQYS-----     478
XP_028372812.1      ------ARDWLRVG----SVL-DRLLFRVYLL----AVLAYSITLVTLWSIWQYS-----     483
XP_008589608.1      ------ARDWLHVG----SVL-DKLLFRVYVL----AVLAYSITLVMLWSIWQYA-----     485
XP_012865077.1      ------ARDWLHVG----SVL-DKLLFRIYLL----AVLAYSITLVTLWSIWHYS-----     483
NP_001166178.1      ------ARDWLRVG----SVL-DKLLFRVYLL----AVLAYSITLVTLWSVWHYA-----     490
XP_004636553.1      ------ARDWLRVG----SVL-DKLLFRIYLL----AVLAYSITLVTLWSVWHYS-----     484
XP_004856670.1      ------ARDWLRVG----SVL-DKLLFRVYLL----AVLTYSITLVTLWSIWHYS-----     484
XP_010627744.1      ------ARDWLRVG----SVL-DKLLFRIYLL----AVLAYSITLVTLWSIWHYS-----     484

NP_509270.1         ---------------------------------    434
VDO93178.1          ---------------------------------    491
PAV91580.1          PEDPYFFI-------------------------    550
VDK46997.1          ---------------------------------    351
VIO86814.1          ---------------------------------    532
VDP38785.1          VVALPILVFTSASEP-RCSS--------MM---    562
KOF68401.1          ---------------------------------    469
CDJ96026.1          HLRTYN---------------------------    674
EFX76216.1          ---------------------------------    442
KAF7403848.1        ---------------------------------    465
```

```
KAF7427032.1        ---------------------------------    480
KAE9417558.1        ---------------------------------    461
KJH51504.1          ---------------------------------    469
VDL69795.1          ---------------------------------    433
VDO32671.1          ---------------------------------    462
VBB32409.1          ---------------------------------    482
VDN54565.1          ---------------------------------    500
EGT55171.1          ---------------------------------    514
RCN52111.1          ---------------------------------    516
RMX54856.1          ---------------------------------    565
XP_022781674.1      ---------------------------------    826
RNA37099.1          M--------------------------------    620
KAA0187152.1        ST-------------------------------    756
GAU96593.1          PIENTL---------------------------    559
XP_009043980.1      ---------------------------------    444
VDM43573.1          VFDESLYGLH-----------------------    589
XP_003140283.1      LEENFDWNL------------------------    475
VDN82010.1          LDENSDWNV------------------------    500
VDO31501.1          LDENSDWNV------------------------    512
VDP19591.1          ---------------------------------    701
EDO32053.1          ---------------------------------    324
TRY67230.1          ---------------------------------    475
KAF7391312.1        ---------------------------------    502
GFG30449.1          ---------------------------------    462
KQS30083.1          ---------------------------------    397
EDW57583.2          ---------------------------------    692
KNC22799.1          ---------------------------------    501
RZF44856.1          ---------------------------------    497
ENN76856.1          ---------------------------------    431
KAF5300392.1        ---------------------------------    525
CAB3239999.1        ---------------------------------    486
PCG77624.1          ---------------------------------    429
PZC79131.1          ---------------------------------    507
KAF4083067.1        ---------------------------------    368
XP_009924659.1      ---------------------------------    421
XP_010123544.1      ---------------------------------    420
CBN81618.1          ---------------------------------    381
KAF3704230.1        ---------------------------------    457
RUS86578.1          ---------------------------------    466
XP_034309618.1      ---------------------------------    412
PIK58946.1          DI-------CQSFDKMM----------------    484
VUZ42516.1          EFLIKRFKELSKYVD------PI----------    522
PAA51166.1          -LE--YSNEDQEFCR------------------    548
TGZ55997.1          EFL--YSEE------------------------    599
VDP48851.1          ---------------------------------    474
PVD38331.1          DD-------------------------------    511
VDK73355.1          XXXXXXXXXXXXXA-------------------    486
VDD84963.1          SSS------------------------------    498
VDK42196.1          DLQYRPTNISSPIVQ------------------    443
KFD58288.1          V--------------------------------    510
OUC49089.1          V--------------------------------    509
EYC26492.1          A--------------------------------    537
VDK27218.1          A--------------------------------    472
VDM17286.1          SIIRKFSYNRRDLQ-------------------    403
VDL91846.1          ---------------------------------    415
KAE9548540.1        NITAPTKPLGQANI----HSLRMKF--------    560
PDM74087.1          EALWRITEEFKKFRDKKEMVDKGDFL-------    508
XP_024504322.1      VELERLREIYNSYKKGP----------------    512
PIO52336.1          AVLDRLIDLYKSGGNRD----------------    498
TKR73865.1          MELERLKDLYKNAKLNLG---------------    502
RLU23395.1          RIIEIYRG-K-----------------------    526
XP_002427906.1      KIIEIYRG-K-----------------------    513
TMW47392.1          RIIEIYRG-K-----------------------    507
VDD83956.1          TALTAPSVDAINVTRLSCE-------TKLV---    502
VEL33078.1          ---------------------------------    424
XP_009019088.1      IFS------------------------------    500
KAE9536378.1        DRDLSFIA-KKQFSPMSDME-------------    592
KDR23473.1          DMELSSVA-KQQFLRDIQNSD------------    535
KAF7266914.1        DQKLSEIP-LSKIFQNQLPPLPPGLPPV-----    551
KAF4519445.1        ---------------------------------    400
OXU25983.1          DRQVSSIP-LRKGTYMLPP---QSFFDY----D    537
TGZ32403.1          DEKHTSIS-IRNY--MYPP---PGHNGSLMNED    545
ELT94491.1          ENNDY----------------------------    519
VVC39575.1          ---------------------------------    565
RVE49089.1          DVRLSEIA-YATAKPRPPPPR------------    534
KAB0800277.1        DILISKVA-KKKMALLKMEPEEL----------    552
TDG52197.1          DILYSKIA-KKKFELLKMGSDNS-----L----    553
```

```
TMW48669.1          ---------------------------------    335
XP_025896085.1      ---------------------------------    487
KAF2977017.1        FDNLTQ-----ISLPVESIWVPDILINEL----    296
XP_009321837.1      FGGRRVP--ARANALMVCKWLPIDEVSS-----    494
XP_009979985.1      ---------------------------------    431
XP_028942374.1      ---------------------------------    456
PKK19633.1          ---------------------------------    476
XP_009894240.1      ---------------------------------    500
XP_032820219.1      ---------------------------------    502
XP_029429480.1      KDPP------KNITTYEREW-------------    520
XP_030077572.1      ---------------------------------    464
XP_033774596.1      CQME------S----------------------    493
ETE72600.1          ---------------------------------    476
XP_018082638.1      ---------------------------------    689
XP_032905410.1      ---------------------------------    468
XP_020773490.1      ---------------------------------    496
XP_033833634.1      ---------------------------------    505
XP_030215795.1      ---------------------------------    522
XP_030602980.1      ---------------------------------    498
XP_004573543.1      ---------------------------------    491
XP_005916159.1      ---------------------------------    492
XP_013132089.1      ---------------------------------    492
XP_031603488.1      ---------------------------------    484
XP_005725536.1      ---------------------------------    484
XP_026038075.1      ---------------------------------    498
CAF96649.1          ---------------------------------    457
XP_023818570.1      ---------------------------------    488
RVE64289.1          ---------------------------------    523
XP_024121971.1      ---------------------------------    494
XP_015817559.1      ---------------------------------    478
XP_015225670.1      ---------------------------------    398
XP_012722063.1      ---------------------------------    492
XP_032431307.1      ---------------------------------    492
XP_014328329.1      ---------------------------------    492
XP_027886578.1      ---------------------------------    492
XP_008426791.1      ---------------------------------    492
XP_014838686.1      ---------------------------------    492
XP_014885967.1      ---------------------------------    492
XP_033954312.1      ---------------------------------    498
XP_034089244.1      ---------------------------------    487
XP_010780064.1      ---------------------------------    330
XP_033970261.1      ---------------------------------    514
XP_029375575.1      ---------------------------------    529
KAF0023022.1        ---------------------------------    530
XP_019952614.1      ---------------------------------    483
XP_034463117.1      ---------------------------------    495
XP_029924656.1      ---------------------------------    493
XP_028323228.1      ---------------------------------    489
TNM84480.1          ---------------------------------    311
XP_029703788.1      ---------------------------------    492
XP_030010368.1      ---------------------------------    495
XP_019725056.1      ---------------------------------    490
XP_034034934.1      ---------------------------------    481
XP_029956880.1      ---------------------------------    492
XP_008331307.3      ---------------------------------    527
XP_031724845.1      ---------------------------------    493
XP_013859395.1      ---------------------------------    493
XP_017275335.1      ---------------------------------    493
XP_020496197.1      ---------------------------------    491
XP_029030400.1      ---------------------------------    492
XP_022060538.1      ---------------------------------    494
XP_023150584.1      ---------------------------------    494
XP_029303892.1      ---------------------------------    516
XP_020454172.1      ---------------------------------    491
TKS83244.1          ---------------------------------    476
XP_022616583.1      ---------------------------------    522
XP_030293172.1      ---------------------------------    494
XP_027129539.1      ---------------------------------    489
XP_034539538.1      ---------------------------------    492
XP_008277330.1      ---------------------------------    492
XP_028276940.1      ---------------------------------    492
XP_023263433.1      ---------------------------------    489
XP_034406336.1      ---------------------------------    492
XP_026184460.1      ---------------------------------    492
KAF1378228.1        ---------------------------------    477
XP_028450365.1      ---------------------------------    481
XP_031163851.1      ---------------------------------    492
```

XP_032389084.1	---------------------------------	492
XP_033494682.1	---------------------------------	492
XP_018544782.1	---------------------------------	492
XP_026228189.1	---------------------------------	492
XP_028976606.1	---------------------------------	539
CAB1352378.1	---------------------------------	450
XP_023866849.1	---------------------------------	493
XP_013992832.1	---------------------------------	493
XP_029546688.1	---------------------------------	493
XP_021481546.1	---------------------------------	493
XP_020339889.1	---------------------------------	493
XP_029481972.1	---------------------------------	493
KPP68743.1	---------------------------------	459
XP_023669383.1	---------------------------------	481
KAA0720096.1	---------------------------------	388
XP_009293684.1	---------------------------------	482
XP_026090784.1	---------------------------------	482
XP_018918715.1	---------------------------------	482
KAF4098549.1	---------------------------------	482
XP_016084173.1	---------------------------------	482
XP_016332759.1	---------------------------------	482
XP_016398039.1	---------------------------------	482
XP_026989970.1	KEQRN-V--SLCHISY-----------------	499
TSQ12698.1	---------------------------------	482
XP_017347546.1	---------------------------------	476
XP_026794616.2	---------------------------------	482
XP_026861889.1	---------------------------------	480
XP_017575347.1	---------------------------------	480
XP_022531596.1	---------------------------------	480
XP_012691019.2	---------------------------------	465
XP_028839723.1	---------------------------------	483
XP_030638860.1	---------------------------------	463
XP_007882964.1	---------------------------------	461
GCB70244.1	---------------------------------	377
GCC26242.1	---------------------------------	481
XP_020387034.1	---------------------------------	472
XP_028931940.1	---------------------------------	485
XP_031762490.1	---------------------------------	474
PIO32240.1	---------------------------------	272
XP_018425466.1	---------------------------------	475
XP_006642351.1	---------------------------------	498
XP_028665372.1	---------------------------------	481
GCF49814.1	---------------------------------	460
XP_015277816.1	---------------------------------	485
XP_005987340.2	---------------------------------	465
XP_019339534.1	---------------------------------	487
XP_025063414.1	---------------------------------	487
XP_019367488.1	---------------------------------	487
XP_019412078.1	---------------------------------	487
XP_014434314.1	---------------------------------	493
XP_007060556.1	---------------------------------	481
XP_024072175.1	---------------------------------	471
XP_008170567.1	---------------------------------	487
XP_034610543.1	---------------------------------	487
XP_030394127.1	---------------------------------	484
XP_032651960.1	---------------------------------	487
XP_025020083.1	---------------------------------	484
XP_026536833.1	---------------------------------	484
XP_026564787.1	---------------------------------	484
XP_032084675.1	---------------------------------	469
XP_029139341.1	---------------------------------	484
XP_034281006.1	---------------------------------	484
XP_020649062.1	---------------------------------	484
XP_008117087.1	---------------------------------	484
XP_028564324.1	---------------------------------	490
XP_033028155.1	---------------------------------	489
XP_025963249.1	---------------------------------	471
XP_009668348.1	---------------------------------	486
XP_013798935.1	---------------------------------	470
XP_025913685.1	---------------------------------	495
XP_013042552.1	---------------------------------	493
XP_005030458.2	---------------------------------	486
XP_032057953.1	---------------------------------	469
XP_021232050.1	---------------------------------	408
OXB62403.1	---------------------------------	487
OXB81319.1	---------------------------------	469
XP_010722007.1	---------------------------------	488

```
XP_015739349.1      ---------------------------------     488
XP_031455498.1      ---------------------------------     489
POI27435.1          ---------------------------------     464
XP_004948120.1      ---------------------------------     489
XP_032851190.1      ---------------------------------     447
XP_010007255.1      ---------------------------------     480
XP_030320702.1      ---------------------------------     445
XP_010191940.1      ---------------------------------     417
XP_027737112.1      ---------------------------------     482
XP_027555032.1      ---------------------------------     487
XP_032565370.1      ---------------------------------     533
XP_027511217.1      ---------------------------------     632
XP_027593499.1      ---------------------------------     583
XP_017664924.1      ---------------------------------     484
XP_029817938.1      ---------------------------------     466
XP_005058641.1      ---------------------------------     519
XP_021385804.1      ---------------------------------     484
XP_030146687.2      ---------------------------------     531
KAF4796420.1        ---------------------------------     486
XP_032937581.1      ---------------------------------     533
XP_031989659.1      ---------------------------------     487
XP_010402086.1      ---------------------------------     486
XP_017594069.1      ---------------------------------     457
XP_014115268.1      ---------------------------------     511
XP_023797108.1      ---------------------------------     457
XP_033375614.1      ---------------------------------     457
XP_014740121.1      ---------------------------------     631
RLV83430.1          ---------------------------------     477
XP_009096098.2      ---------------------------------     593
TRZ15870.1          ---------------------------------     487
RMB91935.1          ---------------------------------     457
XP_030820843.1      ---------------------------------     533
XP_014165179.1      ---------------------------------     535
XP_026653582.1      ---------------------------------     417
PKU35975.1          ---------------------------------     488
XP_014805072.1      ---------------------------------     480
XP_009818330.1      ---------------------------------     198
OPJ68307.1          ---------------------------------     485
XP_008936289.1      ---------------------------------     487
XP_010287046.1      ---------------------------------     374
XP_005240140.2      ---------------------------------     540
XP_005437752.2      ---------------------------------     546
KFV74811.1          ---------------------------------     446
XP_010018389.1      ---------------------------------     493
KQK78711.1          ---------------------------------     486
XP_009570162.1      ---------------------------------     485
KFP11268.1          ---------------------------------     412
KFQ98910.1          ---------------------------------     468
XP_012985202.3      ---------------------------------     486
XP_010573388.1      ---------------------------------     485
XP_029879496.1      ---------------------------------     457
XP_030361086.1      ---------------------------------     538
KFM00668.1          ---------------------------------     469
KAF1479074.1        ---------------------------------     449
KAF1651161.1        ---------------------------------     450
KAF1673648.1        ---------------------------------     450
KAF1493319.1        ---------------------------------     450
KAF1584157.1        ---------------------------------     450
KAF1571723.1        ---------------------------------     450
KAF1533169.1        ---------------------------------     450
KAF1638955.1        ---------------------------------     450
KAF1549972.1        ---------------------------------     450
KAF1606914.1        ---------------------------------     450
KAF1510957.1        ---------------------------------     450
KAF1498899.1        ---------------------------------     450
KAF1411525.1        ---------------------------------     450
KAF1429205.1        ---------------------------------     450
XP_005334318.1      ---------------------------------     472
XP_014395552.1      ---------------------------------     472
KAB0404854.1        ---------------------------------     448
XP_007494828.1      ---------------------------------     488
XP_003764254.1      ---------------------------------     488
XP_020845489.1      ---------------------------------     488
XP_027703160.1      ---------------------------------     488
XP_017523929.1      ---------------------------------     480
XP_008688428.1      ---------------------------------     428
XP_029812166.1      ---------------------------------     497
```

XP_025749781.1	---------------------------------	483
XP_004416432.1	---------------------------------	483
XP_027436262.1	---------------------------------	483
XP_032284025.1	---------------------------------	483
XP_006735421.1	---------------------------------	483
XP_021552166.1	---------------------------------	483
XP_026361066.1	---------------------------------	483
XP_034523598.1	---------------------------------	483
NP_001297113.1	---------------------------------	483
XP_032215488.1	---------------------------------	483
VCX31483.1	---------------------------------	483
XP_022369003.1	---------------------------------	482
XP_032694248.1	---------------------------------	482
NP_001041584.1	---------------------------------	483
XP_025862501.1	---------------------------------	483
KAF0873564.1	---------------------------------	625
XP_025784751.1	---------------------------------	477
XP_007075625.1	---------------------------------	619
XP_030189489.1	---------------------------------	483
XP_019324655.1	---------------------------------	584
XP_023094886.1	---------------------------------	619
XP_026892357.1	---------------------------------	483
XP_016004457.1	---------------------------------	478
XP_006912860.1	---------------------------------	478
XP_011363679.1	---------------------------------	478
XP_016076060.1	---------------------------------	483
XP_008148371.1	---------------------------------	483
XP_006093568.1	---------------------------------	483
XP_006761601.1	---------------------------------	483
XP_024426743.1	---------------------------------	478
XP_019520779.1	---------------------------------	483
XP_032976539.1	---------------------------------	483
ELW64270.1	---------------------------------	487
XP_004427367.1	---------------------------------	483
XP_008529353.1	---------------------------------	488
NP_001288165.1	---------------------------------	483
XP_014711213.1	---------------------------------	483
XP_012514621.1	---------------------------------	483
XP_008071525.1	---------------------------------	480
XP_009005082.1	---------------------------------	483
XP_010332832.1	---------------------------------	478
XP_012326058.1	---------------------------------	489
XP_017378796.1	---------------------------------	512
XP_032141374.1	---------------------------------	512
XP_032024018.1	---------------------------------	478
XP_018891497.2	---------------------------------	484
XP_030684894.1	---------------------------------	478
PNJ75904.1	---------------------------------	484
AAP35868.1	---------------------------------	484
XP_001149570.1	---------------------------------	484
XP_003805532.1	---------------------------------	484
XP_023063823.1	---------------------------------	482
XP_033060210.1	---------------------------------	482
XP_010370669.1	---------------------------------	482
XP_017738496.1	---------------------------------	482
XP_011782168.1	---------------------------------	483
EHH23440.1	---------------------------------	510
XP_003910778.3	---------------------------------	483
XP_025212649.1	---------------------------------	489
XP_008019132.1	---------------------------------	483
XP_011832421.1	---------------------------------	468
XP_011759618.1	---------------------------------	483
XP_005579749.1	---------------------------------	483
XP_011921684.1	---------------------------------	275
XP_008259391.1	---------------------------------	483
XP_012782438.1	---------------------------------	479
VTJ86076.1	---------------------------------	489
XP_015345533.1	---------------------------------	489
XP_027786432.1	---------------------------------	489
XP_026248345.1	---------------------------------	483
XP_005378209.1	---------------------------------	484
XP_004466010.1	---------------------------------	483
XP_020035707.1	---------------------------------	477
XP_006181802.1	---------------------------------	483
XP_006207862.1	---------------------------------	483
XP_005891968.1	---------------------------------	480
XP_010828662.1	---------------------------------	480

```
XP_019830126.1      ---------------------------------    479
XP_017914486.1      ---------------------------------    479
XP_006060266.2      ---------------------------------    484
XP_020728447.1      ---------------------------------    484
KAF4008892.1        ---------------------------------    484
KAB0348059.1        ---------------------------------    467
KAB0371113.1        ---------------------------------    460
MBV96963.1          ---------------------------------    483
XP_007172048.1      ---------------------------------    483
XP_007452270.1      ---------------------------------    483
XP_023987108.1      ---------------------------------    478
XP_004273432.1      ---------------------------------    478
XP_004328646.1      ---------------------------------    478
XP_026937670.1      ---------------------------------    478
XP_030691031.1      ---------------------------------    478
XP_022414870.1      ---------------------------------    483
XP_029064651.1      ---------------------------------    483
XP_024607566.1      ---------------------------------    483
XP_032496907.1      ---------------------------------    483
XP_007535559.1      ---------------------------------    476
XP_031299536.1      ---------------------------------    482
XP_004666516.1      ---------------------------------    479
XP_008830963.1      ---------------------------------    483
XP_005347338.1      QQGTRGW--GISP--------------------    499
XP_005069548.1      ---------------------------------    483
XP_027267894.1      ---------------------------------    483
OBS80992.1          ---------------------------------    471
XP_006979812.1      ---------------------------------    490
XP_028720687.1      ---------------------------------    484
XP_021489005.1      ---------------------------------    484
XP_031199589.1      ---------------------------------    508
XP_028617944.1      ---------------------------------    298
XP_034347030.1      ---------------------------------    481
NP_077370.2         ---------------------------------    483
XP_032766961.1      ---------------------------------    483
XP_021063186.1      ---------------------------------    466
NP_001093114.1      ---------------------------------    483
XP_021028379.1      ---------------------------------    483
XP_004689305.1      ---------------------------------    483
XP_004712871.1      ---------------------------------    483
XP_004382575.1      ---------------------------------    483
XP_010596188.1      ---------------------------------    483
XP_006890855.1      ---------------------------------    483
XP_006834038.1      ---------------------------------    483
XP_007934716.1      ---------------------------------    483
XP_012603491.1      ---------------------------------    483
XP_012663606.1      ---------------------------------    478
XP_028372812.1      ---------------------------------    483
XP_008589608.1      ---------------------------------    485
XP_012865077.1      ---------------------------------    483
NP_001166178.1      ---------------------------------    490
XP_004636553.1      ---------------------------------    484
XP_004856670.1      ---------------------------------    484
XP_010627744.1      ---------------------------------    484
```

S6 Fig. Multiple sequence alignment of 449 sequences of 5HT3A subunit homologs. ClustalW alignment of sequences whose details can be found in S1 Table. Accession number represents the species. The colours of the accession numbers in the alignment correspond to the colours of the phyla: Chordata (green with the human sequence highlighted with yellow background), Nematoda (dark red), Arthropoda (orange), Platyhelminthes (dark purple), Mollusca (cyan), Rotifera (yellow), Tardigrada (maroon), Echinodermata (teal), Annelida (blue) and Cnidaria (grey). The Cys-loop and transmembrane (TM) domains are highlighted in yellow and A to E loops in the ligand binding region are highlighted in grey. The symbols asterisk (*), colon (:), and dot (.) indicate identical amino acid residues, conserved substitutions, and semi-conserved substitutions in all sequences used in the alignment respectively are present on pages 83 and 89.
