# Supplementary figures and images for: Phylogenetic analyses of 5-hydroxytryptamine 3 (5-HT3) receptors in Metazoa
Source: PLoS One. 2023 Mar 1;18(3):e0281507. doi: 10.1371/journal.pone.0281507 (PMC9977066; doi:10.1371/journal.pone.0281507)

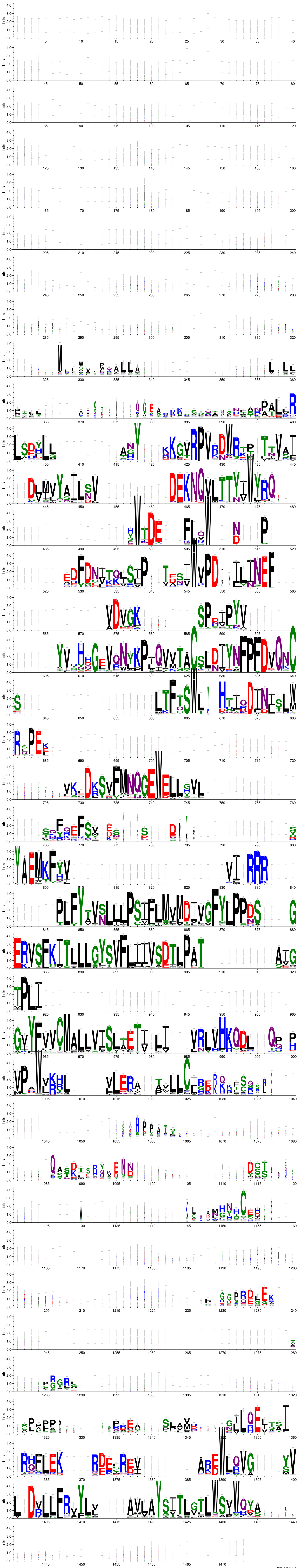

Supplement: S7 Fig — The height of each letter is proportional to the observed frequency of the corresponding amino acid. The overall height of each stack is proportional to the sequence conservation, measured in bits from 1 to 4 units, at that position. The frequency and the position of the amino acids are represented on the y and x axis, respectively. Amino acids are coloured according to their chemical properties; polar amino acids G, S, T, Y and C in green, neutral amino acids Q or N in purple, basic amino acids K, R and H in blue, acidic amino acids D and E in red, hydrophobic amino acids A, V, L, I, P, W, F, and M in black. (PDF) [file pone.0281507.s011.pdf]
