# Supplementary material for: Phylogenetic analyses of 5-hydroxytryptamine 3 (5-HT3) receptors in Metazoa
Source: PLoS One. 2023 Mar 1;18(3):e0281507. doi: 10.1371/journal.pone.0281507 (PMC9977066; doi:10.1371/journal.pone.0281507)
Supplement: S8 Fig — ClustalW alignment of sequences from phylum Chordata (human—Homo sapiens (AAP35868.1), cat—Felis catus (XP_023094886.1), dog—Canis lupus familiaris (NP_001041584.1), pig—Sus scrofa (XP_003357349.2)), parasitic round worm from Nematoda—Toxocara canis (VDM43573.1), and flat worms from Platyhelminthes: flatworm1—Schistosoma bovis (RTG88761.1) and flatworm2—Sparganum proliferum (VZH96180.1). Extracellular loops are highlighted in grey in order of appearance D, A, E, B, F and C; Cys-loop in blue; and transmembrane (TM) domains TM1, TM2, TM3 and TM4 labelled in orange. The asterisk (*), colon (:), and dot (.) indicate identical amino acid residues, conserved substitutions, and semi-conserved substitutions in all sequences used in the alignment respectively. (PDF) [file pone.0281507.s012.pdf]

```
Flatworm1       MYQFPTSIPTAFKSYDASDSVSVDELGISLNTSGPRDQLLETAVSRSELNEELNLDDTMT 60
Flatworm2       ------------------------------------------------------------ 0
Roundworm       ------------------------------------------------------------ 0
Human           ------------------------------------------------------------ 0
Pig             ------------------------------------------------------------ 0
Dog             ------------------------------------------------------------ 0
Cat             ------------------------------------------------------------ 0

Flatworm1       KNSSGNIKLKLQVIYLYLLIDWQIYIEVNSTIKFHNLLSCVYEVEMYQHTLTIRPLPRNC 120
Flatworm2       ------------------------------------------------------------ 0
Roundworm       ------------------------------------------------------------ 0
Human           ------------------------------------------------------------ 0
Pig             ------------------------------------------------------------ 0
Dog             ------------------------------------------------------------ 0
Cat             ------------------------------------------------------------ 0

Flatworm1       PVSLIKDLFPSSVNVRIPQKCNSRFGFVKFRNHDELVAAQKSVSGKLLNGKQIKIEICSL 180
Flatworm2       ------------------------------------------------------------ 0
Roundworm       ------------------------------------------------------------ 0
Human           ------------------------------------------------------------ 0
Pig             ------------------------------------------------------------ 0
Dog             ------------------------------------------------------------ 0
Cat             ---------------------------------------------------------MPF 3

Flatworm1       NGPDNVDKSKWTEPLQRKLSDFDMRSLHVLHLPRATTRFDLAQVFPKAVGIRMPTYDDGS 240
Flatworm2       ------------------------------------------------------------ 0
Roundworm       ------------------------------------------------------------ 0
Human           ------------------------------------------------------------ 0
Pig             ------------------------------------------------------------ 0
Dog             ------------------------------------------------------------ 0
Cat             LSLSPFKTSAWTP------------------------------------GDSLVLLALSC 27

Flatworm1       CRGYCTLTYHSRHIALKDFELRHGTFIHGESIYVMFLLKNLKKISMRNAKRHTSDIYQMD 300
Flatworm2       ------------------------------------------------------------ 0
Roundworm       -------------------------------------MPNSAL------------RSVMA 11
Human           ------------------------------------------------------------ 0
Pig             ------------------------------------------------------------ 0
Dog             ------------------------------------------------------------ 0
Cat             CQAWMYSFWSGV--------RKHMTSEKGSPISLSWTKPSGTK------------LWLIA 67

Flatworm1       VDSTSLPHKEKCAKAIVDHPIPMSIDSF--DPEFKVAKSPATSGKPVKKKSKVATVQRDI 358
Flatworm2       ------------------------------------------------------------ 0
Roundworm       -AKQ---TKRVCVV---AGRLPRDRERRLVRLVQQCSGRAPALGRAGRQF---------- 54
Human           ------------------------------------------------------------ 0
Pig             ------------------------------------------------------------ 0
Dog             ------------------------------------------------------------ 0
Cat             IQRDSIRTWRLSSA---GG--PSAVGG---CSLRGVEGGPEEAGRLGREA---------- 109

Flatworm1       PDPLINKKYQLKEKSVKRKSSSTSIFDSLFQSSTGANDKKQKKKTKILENLFELTI--NT 416
Flatworm2       -----------------------------------------MGFMGVLL-------VLLV 12
Roundworm       ------------ERSLERCLTPPVL--PMMAT-------RRRCFL-ILLQLFHKLVAV-- 90
Human           ----------------------------------------MLGKLAMLLWVQQALLALLL 20
Pig             ----------------------------------------------MLLWVRQALLALLL 14
Dog             ----------------------------------------------MLLWVPQALLALLL 14
Cat             ------------GRVWAACLSARLTLRPLQGS-------SRPGKVTMLSWVPRVLLALLL 150
                                                              :*

Flatworm1       TVLMAQTNV---DHAETESSMEKSLIRV--ILERYKQNGVVGRPVNNSRVKMVVQYGLQM 471
Flatworm2       GYVAGY----------------KEASRLYDVLMNQQPYNKIIRPVRNFNDTLTVFFGLGL 56
Roundworm       --VLADVTITDISDIE---GAATDEQKLLYHL--LRQYEKAVRPVRNASHTVTVKLGMTM 143
Human           PTLLAQGEARR-----SRNTTRPALLRLSDYL--LTNYRKGVRPVRDWRKPTTVSIDVIV 73
Pig             PMFLAQGEVMHRGDPQARNTSRPAMLRLSNHL--LANYKKGVRPVRDWRTPTTVSIDVIV 72
Dog             PTLLVQGEARHWRHLQAQNTSRPALLRLSNYL--LANYQKGVRPVRDWRKPTTVSIDVIV 72
Cat             PTLLAHGQARRREHPQAQNTSRPALLRLSDYL--LANYQKGVRPVRDWRKPTTVSIDVIV 208
                  .                       ::   *          ***.:     .*  .: :
```

```
Flatworm1      IQ----------LLGLDENKQVLRTNCWAVYRWSDSLLKWNASQYGGIKELRIFPHQIWT 521
Flatworm2      LQ----------LMDVDEVNQVITSNVLVSLEWTDIKLTWRPEDFGGVTNLFIPSELLWL 106
Roundworm      TNIFEMVIASQIVTFQDEKNQVLTINVWLDQEWKDELLRWDPKQFGGIESIRIPCDLIWL 203
Human          YA----------ILNVDEKNQVLTTYIWYRQYWTDEFLQWNPEDFDNITKLSIPTDSIWV 123
Pig            YA----------ILSVDEKNQVLTTYIWYRQYWTDEFLQWNPEDFDNITKLSLPTDSIWV 122
Dog            YA----------ILSVDEKNQVLTTYIWYRQYWTDEFLQWNPEDFDNITKLSIPTESIWV 122
Cat            YA----------ILSVDEKNQVLTTYIWYRQYWTDEFLQWNPEDFDNITKLSIPTESIWV 258
                           :   ** :**:         *.*  * *  .::..: .: :  . :*

Flatworm1      PDIKLYNFADERLQEFREGRLVVDSSGNILWIQQALFRSTCQVEITYFPFDSQLRSVKRY 581
Flatworm2      PDLLLYNNADGNYVIDIMTKATVFYNGTVRWTPPAIFKSSCHINVEYFPYDIQECSMKFG 166
Roundworm      PDIVLYNNADDYTAGYMRSRAMVFYDGTVFWPPPTQLRSTCKIDVTYFPFDSQHCALKFG 263
Human          PDILINEFVDVGKSPN-IPYVYIRHQGEVQNYKPLQVVTACSLDIYNFPFDVQNCSLTFT 182
Pig            PDILINEFVDVGKSPN-IPYVYVRHHGEVQNYKPLQVVTACSLDIYNFPFDVQNCSLTFT 181
Dog            PDILINEFVDVGKSPS-IPYVYVGHHGEVQNYKPLQVVTACSLDIYNFPFDVQNCSLTFT 181
Cat            PDILINEFVDVGKSPS-IPYVYVGHHGEVQNYKPLQVVTACSLDIYNFPFDVQNCSLTFT 317
               **: : : .*            :   * :       . ::* :::  **:* *  ::.

Flatworm1      R--------------VREH-------------------------L---------IGSTKH 593
Flatworm2      TWTHNGDQVSLKHMTQKHVPDHDGNVHIDYAISLKDFYPSTEFELLKLSAIRRFQFYPCC 226
Roundworm      SWTYHGFQVDITN--R--S----------DNVDLSNYVVSGEFDLVRVHQKRRVVKYTCC 309
Human          SWLHTIQDINISLWRLPEK----------VKSDRSVFMNQGEWELLGVLPYFREFSM-ES 231
Pig            SWLHTIQDINISLLRLPEN----------VKFDRSVFMNQGEWELLGVLTQFQEFSI-ES 230
Dog            SWLHTIQDINISLLRLPEK----------VKLDKTIFMNQGEWELLGVLTQFREFSM-ES 230
Cat            SWLHTIQDINISLWRLPEK----------VKLDKTIFMNQGEWELLGVLTQFREFSM-ES 366
                                                           *

Flatworm1      ERYYPVLRYLIRIYRNPSFHLFILIVPCLLLSLLSLVVFWLPPDSAAKMM---------- 643
Flatworm2      IQPFVDVTFNISMRRKTLFYTINLILPCVGIAFLTILVFYLPSQSGAKIALSINILLSLT 286
Roundworm      PEPYPDVTFFIHIRRKTLYYLYNVVFPCMMMSVLTLLVFLLPPDSGEKIALGITVLLAFS 369
Human          SNYYAEMKFYVVIRRRPLFYVVSLLLPSIFLMVMDIVGFYLPPNSGERVSFKITLLLGYS 291
Pig            SSSYAEMKFYVVIRRRPLFYAVSLLLPSIFLMVMDIVGFYLPPDSGERVSFKITLLLGYS 290
Dog            NSCYAEMKFYVVIRRRPLFYTVSLLLPSIFLMLMDIVGFYLPPDSGERVSFKITLLLGYS 290
Cat            SGCYAEMKFFVVIRRRPLFYTVSLLLPSIFLMFMDIVGFYLPPDSGERVSFKITLLLGYS 426
                  :  : : : : *.  ::   ::.*.: : .: :: * ** :*. ::

Flatworm1      -------------------LGIFFCLNMVMVTLSIFMATWVVNLFYKGNEGQAVPFWIRR 684
Flatworm2      VFLLLLTESIPPTGLVIPLIGKYLLFTMVLVTLSILKTIFVLNLSNRTP-NAPVPVLFRE 345
Roundworm      VFVLAIAEKMPETSDSMPLIGIYLTVVMAMTSVSVVMTVMVLNFHHRGPFNRAVPKWVRR 429
Human          VFLIIVSDTLPATAIGTPLIGVYFVVCMALLVISLAETIFIVRLVHKQDLQQPVPAWLRH 351
Pig            VFLIIVSDTLPATAIGTPLIGVYFVVCMALLVISLAETIFIVRLVHKQDLQQPVPAWLRH 350
Dog            VFLIIVSDTLPATAIGTPLIGVYFVVCMALLVMSLAETIFIVRLVHKQDLQQPVPAWLRH 350
Cat            VFLIIVSDTLPATAIGTPLIGVYFIVCMALLVVSLAETILIVRLVHKQDLQQPVPAWLRH 486
                                  :* :: . *.:  :*:  :  ::.:  :      **  .*.

Flatworm1      F-IIDGLGRMLGIRQIIPLIDKSQRLDPKMSSLNISI-NETNSIQMNYHDNYSMINDDDN 742
Flatworm2      NSKVIRLLRLTGLETPQAP-----RRDSIYQPSAIQSAIHGKTPPKSVF--LPETTDPSV 398
Roundworm      F-VLQRLRKALCMRLPYSG-----WNDSGFCSANGM----TKRVSVGFA--MDELNGDSF 477
Human          L-VLERIAWLLCLREQSTS-----QRPPATSQATKT----DDCSAMGNH--CSHMGGPQD 399
Pig            L-VLERVALLLCLGEQSTS-----WRPPATSQATKT----DDCSDVGNH--CSHVGGPRD 398
Dog            L-VLERVALLLCLGEQSAS-----RRPPATSQTTKT----DDCSDMGNH--CNHLGVPRD 398
Cat            L-VLERVALLLCLGEQTAS-----RRPPATPRAAKT----DDCSDVGNH--CSHLRGPQD 534
                  :  :     :                            .    .

Flatworm1      NQINNFSNKKQLNHNIHLINE-IASNHINEMKHNELKQYSYHIKEF---LQHINIKQIKS 798
Flatworm2      SSSTSSPWTSSTAH-PSDTNR-KKSSVW-----KENLHRNLRILRFIQNLQILAEHLSEL 451
Roundworm      DELDTQLLQ--MQE-TELVNVDDGAAVR-----KKKRKHNELHMRLLKTLQVLIRRQEME 529
Human          ---------------FEKSPRDRCSPP------PPPREASLAVCGLLQELSSIRQFLEKR 438
Pig            ---------------LEKTPRGRGSPP------PPPREASLAVRGLLQELTSIRHFLEKR 437
Dog            ---------------LEKTPRSRGSPP------PPPRESSLAVRGLLKELSSIRHFLEKR 437
Cat            ---------------LEKTPRGRGSPP------SPPREASLAVCGLLQELSSIRHFLEKR 573
                                       :            . .     :   *  :

Flatworm1      SHKNSLGTEWRILALIIDRLFFIIYLITLITIAGIVILKTD-LPDTTLQLINRNVVDLQ- 856
Flatworm2      QRRKSAQDDWIYLSMIFDRLFLCFFVAACLTGALTIIFQAPALYDTTEALTSANVPKFIH 511
Roundworm      DHCQTLANEWRQVAQVIDRLLFWVFLICTVIITLILLIIIPTVHRSMESDV--------- 580
Human          DEIREVARDWLRVGSVLDKLLFHIYLLAVLAYSITLVMLWSIWQYA-------------- 484
Pig            EESREVARDWLRVGSVLDRLLFRIYLLAVLAYSVTLVTLWSIWQYS-------------- 483
Dog            DESREVAREWLHVGSVLDRLLFRIYLVAVLAYSITLITLWSIWQYS-------------- 483
Cat            DESREVAREWLHVGSVLDRLLFRIYLVAVLAYSITLVTLWSIWQYS-------------- 619
               .. .    :*  :. ::*:*:: .::   :  :  ::        :
```

```
Flatworm1       ------------------------------------------------------------ 856
Flatworm2       LTLKSAANNSDIAETRYSTFGRELLAIYLAVKHFRHFLEGRDFTVSTDHKPLTFAPRSHS 571
Roundworm       -----------FDESLY---GLH------------------------------------- 589
Human           ------------------------------------------------------------ 484
Pig             ------------------------------------------------------------ 483
Dog             ------------------------------------------------------------ 483
Cat             ------------------------------------------------------------ 619

Flatworm1       ------------------------------------------------------------ 856
Flatworm2       DKYNPREIAHLDYISQFTTDIRHIDGTKNEVADTLSRPSLSSLQLSHGIDLCAMEAEQQR 631
Roundworm       ------------------------------------------------------------ 589
Human           ------------------------------------------------------------ 484
Pig             ------------------------------------------------------------ 483
Dog             ------------------------------------------------------------ 483
Cat             ------------------------------------------------------------ 619

Flatworm1       ------------------------------------------------------------ 856
Flatworm2       VGCPGDESVSGLLLKDVPLTTGSGTILCDVSTPFHCPFVPASMRQAVFQTLHGLSHPGIR 691
Roundworm       ------------------------------------------------------------ 589
Human           ------------------------------------------------------------ 484
Pig             ------------------------------------------------------------ 483
Dog             ------------------------------------------------------------ 483
Cat             ------------------------------------------------------------ 619

Flatworm1       ------------------------------------------------------------ 856
Flatworm2       ASQKLLTERFVWPGMNKDVKAWARSCLSCQRNKVQRHNKSPPGTFPSPDARFSHVHLDLV 751
Roundworm       ------------------------------------------------------------ 589
Human           ------------------------------------------------------------ 484
Pig             ------------------------------------------------------------ 483
Dog             ------------------------------------------------------------ 483
Cat             ------------------------------------------------------------ 619

Flatworm1       ---------------------      856
Flatworm2       GPLPPSNGFTHLLTCVDRYTR      772
Roundworm       ---------------------      589
Human           ---------------------      484
Pig             ---------------------      483
Dog             ---------------------      483
Cat             ---------------------      619
```

S8 Fig. Multiple sequence alignment of $5HT_3A$ subunit proteins from domestic animals and parasites. ClustalW alignment of sequences from phylum Chordata (human - *Homo sapiens* (AAP35868.1), cat - *Felis catus* (XP_023094886.1), dog - *Canis lupus familiaris* (NP_001041584.1), pig - *Sus scrofa* (XP_003357349.2)), parasitic round worm from Nematoda - *Toxocara canis* (VDM43573.1), and flat worms from Platyhelminthes: flatworm1 - *Schistosoma bovis* (RTG88761.1) and flatworm2 - *Sparganum proliferum* (VZH96180.1). Extracellular loops are highlighted in grey in order of appearance D, A, E, B, F and C; Cys-loop in blue; and transmembrane (TM) domains TM1, TM2, TM3 and TM4 labelled in orange. The asterisk (*), colon (:), and dot (.) indicate identical amino acid residues, conserved substitutions, and semi-conserved substitutions in all sequences used in the alignment respectively.
