# Supplementary material for: Phylogenetic analyses of 5-hydroxytryptamine 3 (5-HT3) receptors in Metazoa
Source: PLoS One. 2023 Mar 1;18(3):e0281507. doi: 10.1371/journal.pone.0281507 (PMC9977066; doi:10.1371/journal.pone.0281507)
Supplement: S1 Fig — Phylogenetic tree of 5HT3 A, B, C, D and E subunit proteins from phylum Chordata. The subunits are coloured according to the legend (A (blue), B (crimson), C (teal), D (purple) and E (grey)) and the human sequence is on an amber background. This analysis involved 85 amino acid sequences. There was a total of 234 alignment positions in the final dataset. Evolutionary analyses were conducted in MEGA X and tree editing was performed in iTOL. Tree scale represents the number of differences between sequences. The species details are listed in S3 Table. (PDF) [file pone.0281507.s005.pdf]

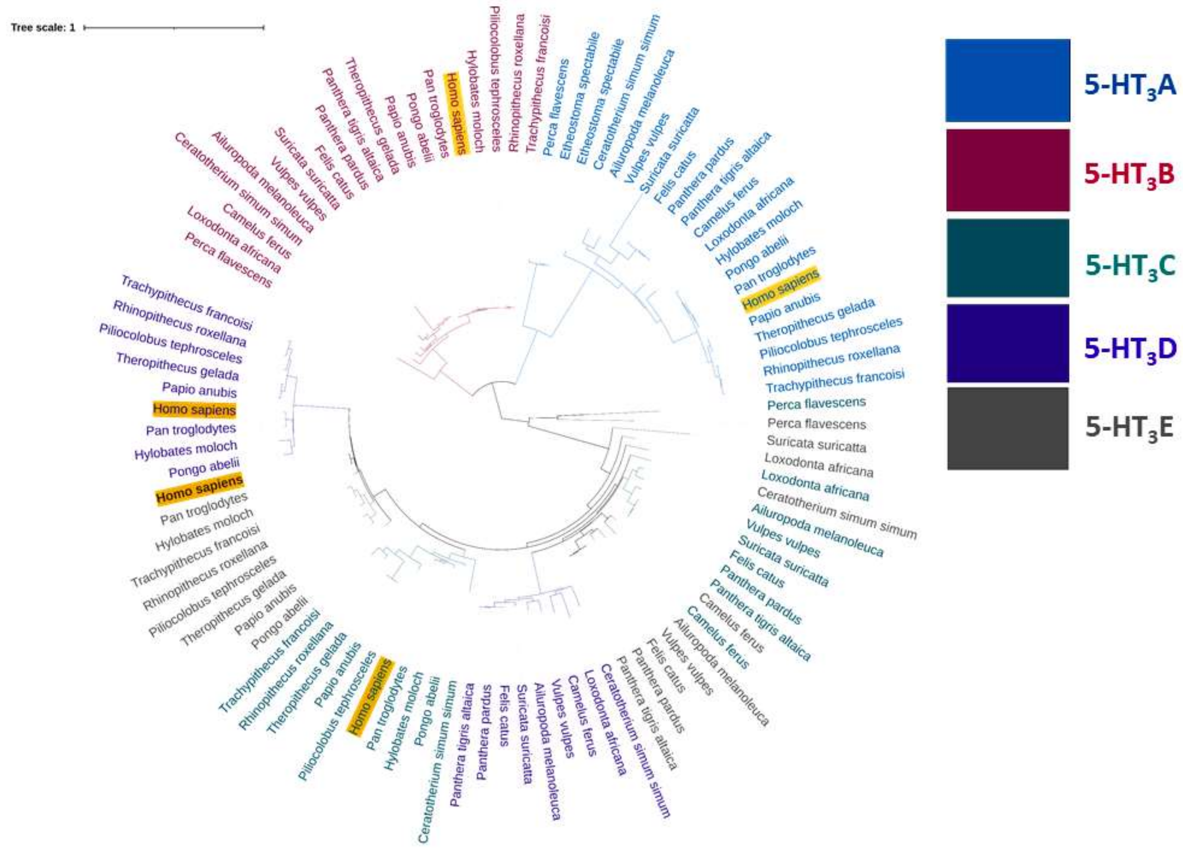

S1 Fig. Phylogenetic tree of 5HT3 A, B, C, D and E subunit proteins from phylum Chordata. The subunits are coloured according to the legend (A (blue), B (crimson), C (teal), D (purple) and E (grey)) and the human sequence is on an amber background. This analysis involved 85 amino acid sequences. There was a total of 234 alignment positions in the final dataset. Evolutionary analyses were conducted in MEGA X and tree editing was performed in iTOL. Tree scale represents the number of differences between sequences. The species details are listed in S3 Table.
