# Supplementary material for: Phylogenetic analyses of 5-hydroxytryptamine 3 (5-HT3) receptors in Metazoa
Source: PLoS One. 2023 Mar 1;18(3):e0281507. doi: 10.1371/journal.pone.0281507 (PMC9977066; doi:10.1371/journal.pone.0281507)
Supplement: S2 Fig — The subunits are coloured according to the legend (Chordata (green), Nematoda (dark red), Arthropoda (orange), Platyhelminthes (dark purple), Mollusca (cyan), Hemichordata (purple), Annelida (blue) and Cnidaria (grey)), where the human sequence is highlighted with amber background. There was a total of 324 alignment positions in the final dataset. Evolutionary analyses were conducted in MEGA X and tree editing was performed in iTOL. Tree scale represents the number of differences between sequences. (PDF) [file pone.0281507.s006.pdf]

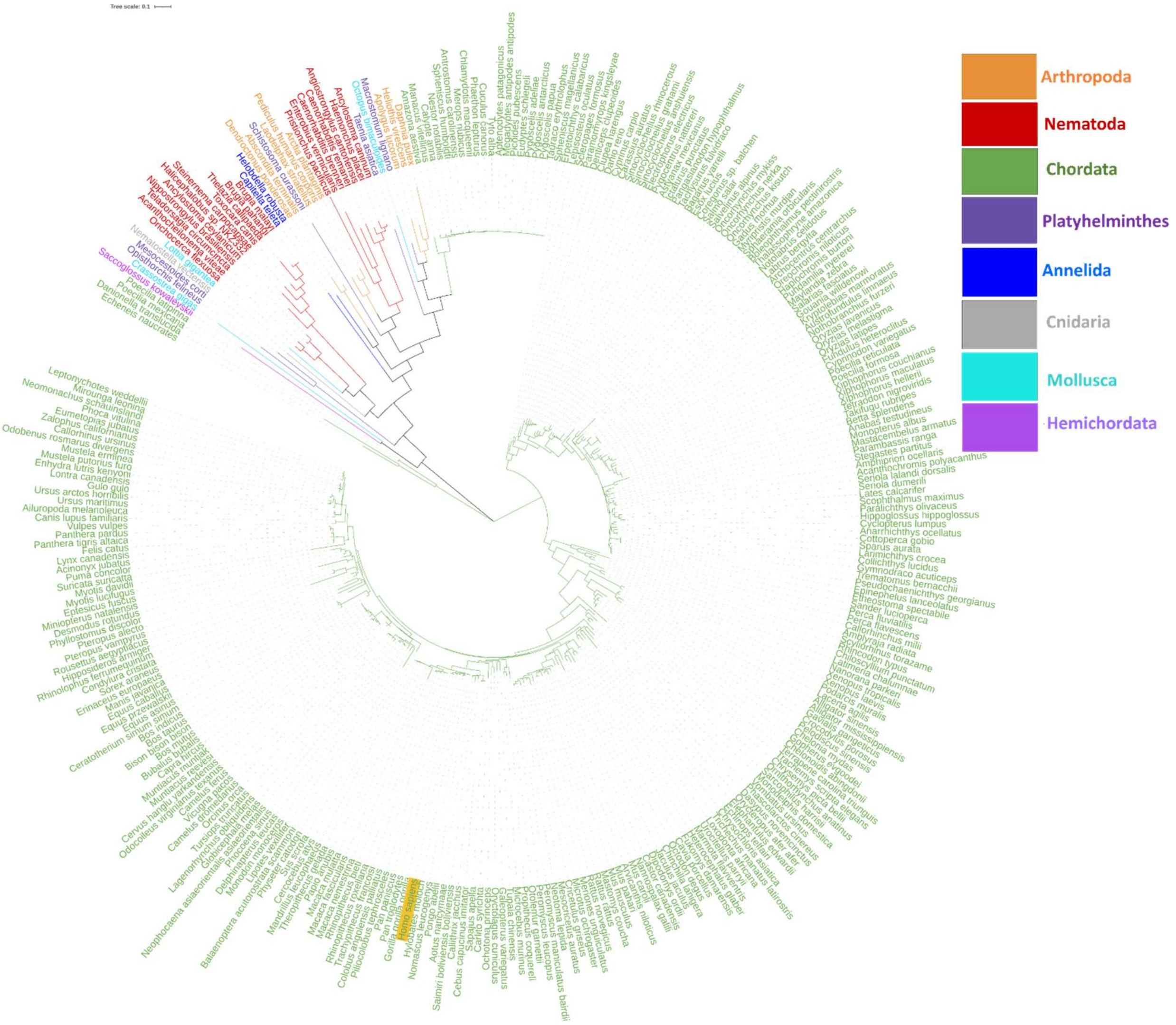

S2 Fig. Phylogenetic tree of 5-HT$_3$ receptor B subunit homologs. The subunits are coloured according to the legend (Chordata (green), Nematoda (dark red), Arthropoda (orange), Platyhelminthes (dark purple), Mollusca (cyan), Hemichordata (purple), Annelida (blue) and Cnidaria (grey)), where the human sequence is highlighted with amber background. There was a total of 324 alignment positions in the final dataset. Evolutionary analyses were conducted in MEGA X and tree editing was performed in iTOL. Tree scale represents the number of differences between sequences.
