# Supplementary material for: Phylogenetic analyses of 5-hydroxytryptamine 3 (5-HT3) receptors in Metazoa
Source: PLoS One. 2023 Mar 1;18(3):e0281507. doi: 10.1371/journal.pone.0281507 (PMC9977066; doi:10.1371/journal.pone.0281507)
Supplement: S3 Fig — The subunits are coloured according to the legend (Chordata (green), Nematoda (dark red), Arthropoda (orange), Platyhelminthes (dark purple), Mollusca (cyan), Annelida (blue) and Cnidaria (grey)) with the human sequence highlighted with amber background. This analysis involved 203 amino acid sequences and there was a total of 277 alignment positions in the final dataset. Evolutionary analyses were conducted in MEGA X and tree editing was performed in iTOL. Tree scale which represents the number of differences between sequences. (PDF) [file pone.0281507.s007.pdf]

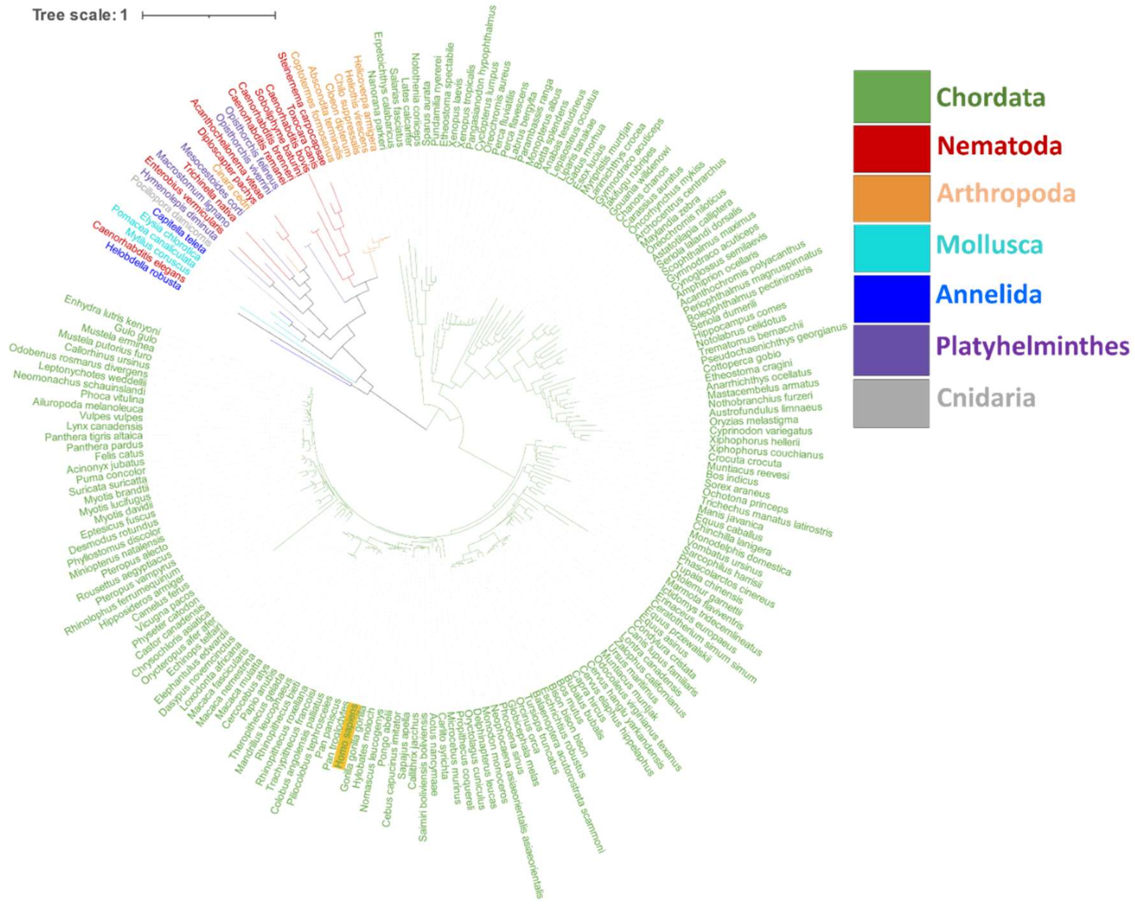

S3 Fig. Phylogenetic tree of 5-HT$_3$ receptor C subunit homologs. The subunits are coloured according to the legend (Chordata (green), Nematoda (dark red), Arthropoda (orange), Platyhelminthes (dark purple), Mollusca (cyan), Annelida (blue) and Cnidaria (grey)) with the human sequence highlighted with amber background. This analysis involved 203 amino acid sequences and there was a total of 277 alignment positions in the final dataset. Evolutionary analyses were conducted in MEGA X and tree editing was performed in iTOL. Tree scale which represents the number of differences between sequences.
