# Supplementary material for: Phylogenetic analyses of 5-hydroxytryptamine 3 (5-HT3) receptors in Metazoa
Source: PLoS One. 2023 Mar 1;18(3):e0281507. doi: 10.1371/journal.pone.0281507 (PMC9977066; doi:10.1371/journal.pone.0281507)
Supplement: S5 Fig — The subunits are coloured according to the legend (Chordata (green with human sequence highlighted with amber background), Nematoda (dark red), Arthropoda (orange), Platyhelminthes (dark purple), Mollusca (cyan), Annelida (blue), Cnidaria (grey)) Orthonectida (pale green) and Echinodermata (teal)). This analysis involved 147 amino acid sequences and 3 sequences were removed due to their placement inaccuracy. There was a total of 164 alignment positions in the final dataset. Evolutionary analyses were conducted in MEGA X and tree editing was performed in iTOL. Tree scale which represents the number of differences between sequences. (PDF) [file pone.0281507.s009.pdf]

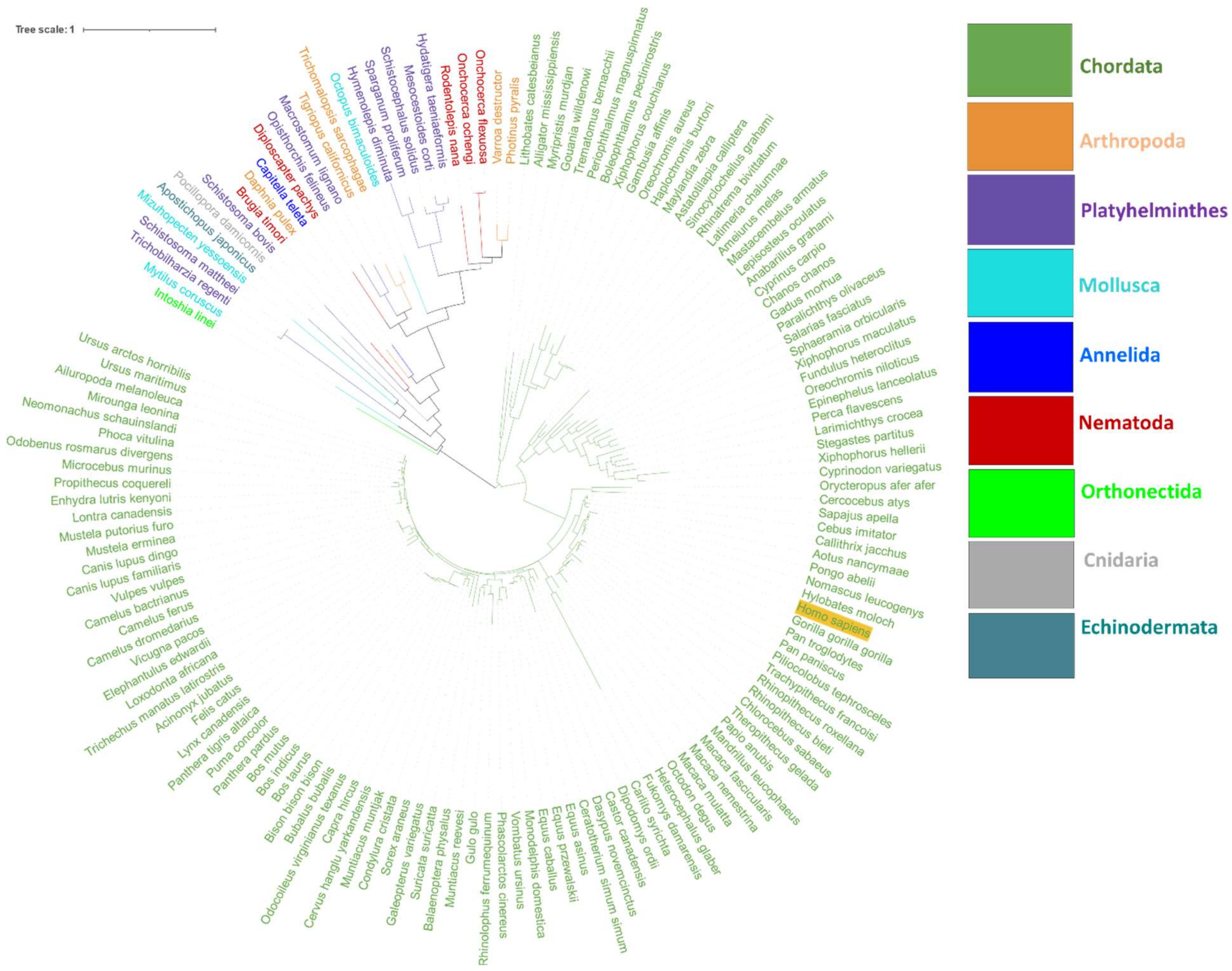

S5 Fig. Phylogenetic tree of 5-HT$_3$ receptor E subunit homologs. The subunits are coloured according to the legend (Chordata (green with human sequence highlighted with amber background), Nematoda (dark red), Arthropoda (orange), Platyhelminthes (dark purple), Mollusca (cyan), Annelida (blue), Cnidaria (grey)) Orthonectida (pale green) and Echinodermata (teal)). This analysis involved 147 amino acid sequences and 3 sequences were removed due to their placement inaccuracy. There was a total of 164 alignment positions in the final dataset. Evolutionary analyses were conducted in MEGA X and tree editing was performed in iTOL. Tree scale which represents the number of differences between sequences.
